# Individual-specific changes in the human gut microbiota after challenge with enterotoxigenic *Escherichia coli* and subsequent ciprofloxacin treatment

**DOI:** 10.1186/s12864-016-2777-0

**Published:** 2016-06-08

**Authors:** Mihai Pop, Joseph N. Paulson, Subhra Chakraborty, Irina Astrovskaya, Brianna R. Lindsay, Shan Li, Héctor Corrada Bravo, Clayton Harro, Julian Parkhill, Alan W. Walker, Richard I. Walker, David A. Sack, O. Colin Stine

**Affiliations:** Center for Bioinformatics and Computational Biology, University of Maryland, College Park, MD USA; Department of Computer Science, University of Maryland, College Park, MD USA; Graduate Program in Applied Mathematics & Scientific Computation, University of Maryland, College Park, MD USA; Department of Biostatistics and Computational Biology, Dana Farber Cancer Institute, Boston, MA USA; Department of Biostatistics, Harvard School of Public Health, Boston, MA USA; Johns Hopkins Bloomberg School of Public Health, Baltimore, MD USA; School of Medicine, University of Maryland, Baltimore, MD USA; Merck & Co. Inc, North Wales, PA USA; Pathogen Genomics Group, Wellcome Trust Sanger Institute, Hinxton, Cambridgeshire, UK; Microbiology Group, Rowett Institute of Nutrition and Health, University of Aberdeen, Aberdeen, UK; PATH, Washington, DC USA

**Keywords:** Diarrhea, Enterotoxigenic *Escherichia coli*, 16S rRNA gene survey, Microbiota, Antibiotic treatment

## Abstract

**Background:**

Enterotoxigenic *Escherichia coli* (ETEC) is a major cause of diarrhea in inhabitants from low-income countries and in visitors to these countries. The impact of the human intestinal microbiota on the initiation and progression of ETEC diarrhea is not yet well understood.

**Results:**

We used 16S rRNA (ribosomal RNA) gene sequencing to study changes in the fecal microbiota of 12 volunteers during a human challenge study with ETEC (H10407) and subsequent treatment with ciprofloxacin.

Five subjects developed severe diarrhea and seven experienced few or no symptoms. Diarrheal symptoms were associated with high concentrations of fecal *E. coli* as measured by quantitative culture, quantitative PCR, and normalized number of 16S rRNA gene sequences. Large changes in other members of the microbiota varied greatly from individual to individual, whether or not diarrhea occurred. Nonetheless the variation within an individual was small compared to variation between individuals. Ciprofloxacin treatment reorganized microbiota populations; however, the original structure was largely restored at one and three month follow-up visits.

**Conclusion:**

Symptomatic ETEC infections, but not asymptomatic infections, were associated with high fecal concentrations of *E. coli*. Both infection and ciprofloxacin treatment caused variable changes in other bacteria that generally reverted to baseline levels after three months.

**Electronic supplementary material:**

The online version of this article (doi:10.1186/s12864-016-2777-0) contains supplementary material, which is available to authorized users.

## Background

Intestinal infection with enterotoxigenic *Escherichia coli* (ETEC) has two expected outcomes: watery diarrhea, or the host remains asymptomatic [1]. The impact of the human intestinal microbiota on the initiation and progression of ETEC diarrhea and subsequent antibiotic treatment is not yet well understood. Although infections with ETEC are rare in high-income countries, they are very common in both children and adults in low-income countries, contributing to an estimated 131,000 deaths per year, as well as being the most common cause of travelers’ diarrhea with an estimated 10 million cases per year [2]. The WHO estimated that ETEC alone contributed to over 86 million cases of foodborne illness and over 26 thousand deaths in 2010 [3]. Because of the public health burden from ETEC diarrhea, efforts are being made to develop vaccines to protect against these pathogens [4]. Candidate vaccines are often evaluated on volunteer subjects during clinical development. The ETEC strain used most frequently in volunteer studies is H10407. About 70 % of naïve volunteers given H10407 following an overnight fast, at a dose of 2x10^7^ colony forming units (CFU), develop moderate or severe watery diarrhea [5]. The utility of candidate vaccines can be assessed by whether immunization reduces the frequency and/or severity of diarrhea following the challenge. For this particular strain, it is important to determine clinical responses to doses lower than 2x10^7^ CFU, therefore we used specimens from volunteers given lower doses to explore questions about changes to the overall gut microbiota occurring during an ETEC infection and to address questions about further changes subsequent to treatment with ciprofloxacin.

The intestinal microbial community is a complex ecosystem that influences human health in numerous ways: regulating metabolism and nutrition [6–8], developing the immune system [9–11], and preventing colonization and invasion by enteropathogens [12–15]. Dietary habits [16, 17], natural physiological changes such as pregnancy [18] or aging [19], infections [20] and medical treatments [21] may alter or disrupt beneficial co-evolved interactions between humans and their microbiota. Specifically, antibiotic treatments for enteric infections such as ETEC may cause significant collateral damage to gut microbiota. The extent of damage is dependent on the type of antibiotics used and the length of their administration [22]. Even a short course of antibiotics may lead to immediate and drastic shifts in gut microbiota, resulting in loss of beneficial species, an increase in drug-resistant strains and predisposition to systematic infections by pathogens [23–25]. Generally, the intestinal ecosystem is able to recover from such insults, but recovery is often incomplete; long-term observational studies have shown what appear to be permanent losses among certain species [21, 24, 26–28]. The vast majority of these are strict anaerobes important in maintaining a healthy gut (e.g., by producing short chain fatty acids); alterations in their abundance in the intestinal microbial community seem to correlate with the most negative health effects. Disruption of commensal microbiota may contribute to a range of significant morbidities such as antibiotic-associated diarrhea [29], inflammatory bowel disease [30], irritable bowel syndrome [31], pseudomembranous colitis [32], and cancer [33–35].

In this study we followed the changes in the microbiota of volunteers during ETEC challenge and subsequent treatment with ciprofloxacin.

## Methods

### Regulatory approval

Use of H10407 was approved under BB-IND#12234.

### Study description

We assessed changes in gut microbiota during volunteer challenge studies to determine the dose response with ETEC H10407 [5]. The volunteer challenge described here used doses of 1x10^5^ and 1x10^6^ (ClinicalTrials.gov, NCT00844493). Each subject received a three-day course of ciprofloxacin whether or not they exhibited symptoms and were followed up on days 28 and 84.

Thirty healthy adult participants (8 females and 22 males, average age 33) were challenged with either 1x10^5^ CFU or 1x10^6^ CFU dose of H10407 with bicarbonate buffer. The health status of subjects was assessed before challenge (for exclusion criteria, see [5]). Starting on day 5, all subjects received a three-day ciprofloxacin treatment (500 mg twice daily). Early antibiotic treatment was given to the patients with severe or moderate diarrhea that lasted for two days or mild/moderate diarrhea experiencing at least two symptoms such as fever (≥38 °C), vomiting, or severe constitutional symptoms (abdominal pain/cramping, headache, myalgias, nausea). Diarrhea was defined as 1 loose/liquid stool of 300 g or ≥2 loose/liquid stools totaling ≥200 g during any 48-h period within 120 h of the challenge. Stool specimens were collected prior to ETEC infection (day -1, 0) and on the days 1–7, 9, 28, 84 after the infection. We sequenced stool specimens from 5 subjects (54 specimens) who developed moderate to severe diarrhea and 7 subjects (78 specimens) who did not. The 12 were chosen based on 1a) presence or 1b) the absence of symptoms and 2) on the maximum number of stools available. Often our subjects did not have a bowel movement and no sample was collected.

### Challenge strain

H10407 is a wild-type virulent ETEC, serotype O78:H11 that produces both heat-labile and heat-stable toxins and expresses colonization factor I. In comparison to the other ETEC strains, H10407 induces a higher proportion of severe diarrhea, with mild fever and vomiting being reported in a relatively higher proportion of subjects [5].

### Fecal shedding/microbiology

Up to two stool specimens were collected on days 0–4. Colonization was defined by detecting H10407 in two stool specimens collected from the same subject at least 24 h after challenge. For quantitative culture, fecal samples were diluted 10-fold up to 10^−5^ in phosphate buffered saline pH 7.0; 0.1 ml aliquots were spread onto MacConkey agar. After overnight incubation, the proportion of 5 *E. coli* colonies that agglutinated with polyclonal rabbit anti-H10407 antiserum (International Centre for Diarrhoeal Disease Research, Bangladesh) was recorded. Quantities of H10407 were expressed as CFUs per gram of stool.

### DNA isolation and quantitative polymerase chain reaction

DNA was isolated from frozen stools using a bead beater with 3 mm-diameter solid-glass beads (Sigma-Aldrich, St. Louis MO, USA) followed by 0.1 mm zirconium beads (BIO-SPEC Inc., Bartlesville, OK, USA) to disrupt cells. Cell slurry was centrifuged at 16,000 g for 1 min, then the supernatant processed using the Qiagen QIAamp DNA stool extraction kit (Hilden, Germany). Extracted DNA was precipitated with ethanol.

Quantitative PCR (qPCR) was performed on 301 samples using the Applied Biosystems 7500/700 Fast Real-Time PCR System with software V2.0.5 (ABI, Foster City, CA, USA) and SYBR Green-Based fluorescent dye, as described previously [36]. The spectrophotometrically determined (Nanodrop 1000, ThermoScientific, Waltham, MA, USA) concentration of H10407 DNA was used to estimate the number of the heat-labile toxin (LT) copies per 100 ng of total stool DNA: one nanogram of purified DNA was estimated to contain 2x10^5^ copies of LT [36].

### Amplification and sequencing

Bacterial DNA was amplified using primers targeting the V1-V2 region of the 16S rRNA gene [20]. Both forward and reverse primers had a 5’ portion specific for use with 454 GS-FLX Titanium sequencing technology. The forward primers contained a barcode between the Titanium and gene specific regions, so samples could be pooled to a multiplex level of 132 samples.

After sequencing, 124 samples passed quality controls, corresponding to data from 50 samples from 5 volunteers with diarrhea and 74 samples from 7 volunteers without diarrhea. Raw data have been submitted to NCBI under project ID: PRJNA298336.

### Analysis pipeline

Data analysis was performed as in [20]. Sequenced reads were filtered for quality, first using the default parameters of the 454 software, then by removing reads containing ambiguous characters (N), as well as reads that were too short (in terms of the number of cycles of the 454 instrument) after trimming. They were then clustered with DNAclust [37] to 99 % identity into clusters for further annotation using reference data from the Ribosomal Database Project [38] (rdp.cme.msu.edu, release 10.4). Sequences without a nearly identical match to the Ribosomal Database (>100 bp perfect match and > 97 % identity by BLAST) were marked as having “no genus match” and assigned an operational taxonomic unit (OTU) identifier. Chimera checking was done with Perseus/UCHIME [39].

### Manual taxonomic classification

Selected OTUs (e.g., OTUs belonging to the pre-infection biomarker) were aligned using MegaBLAST against the NCBI nucleotide database. Manual inspection of the results based on percent identity and query sequence coverage was used to refine automatically determined taxonomic classifications. If the best BLAST hit differed by more than 3 % from the query sequence, or covered less than 95 % of the query sequence, we restricted classification to the genus level.

### Data normalization

We used a Cumulative Sum Scaling (CSS) approach for normalization, which scales counts by dividing the sum of each sample’s counts up to and including the *p*^th^ quantile (i.e., for all samples *j*, ⋅ *S*_*p*_ = ∑_*i*_(*c*_*ij*_|*c*_*ij*_ ≤ *q*_*pj*_), where *q*_*pj*_ is the *p*^th^ quantile of sample *j*). Normalized counts are given by $\frac{c_{ij}}{s_{pj}}1000$. A complete description is in [40].

### Statistical analysis and diversity estimations

To estimate the changes in the abundance of specific taxa associated with disease, we fit a linear mixed model for each OTU independently to account for individual patient variability while controlling for sampling day. We also included the Cumulative Sum Scaling normalization factor as a covariate in the linear mixed models. We retained only OTUs where the boundaries of the estimated Wald-type 95 % confidence intervals agreed in sign with each other. These analyses were performed with the R package lme4 version 1.1–8 [41].

To calculate Shannon diversity and Bray-Curtis dissimilarity we used the R package Vegan, version 2.3-0 [42].

### Analyzing the effect of ciprofloxacin on the gut microbiota

Samples from 7 patients without diarrhea were analyzed separately to identify OTUs with the largest change in abundance immediately after the initiation of antibiotic treatment. A baseline level of change in the normalized abundance of OTUs was determined by comparing OTU abundances between days 4 and 5 (prior to ciprofloxacin treatment), using the formulae: $diff(OTU)=\frac{abundance\left(OTU, day5\right)- abundance\left(OTU, day4\right)}{abundance\left(OTU, day4\right)+1}$. The upper and lower 1 % of the relative difference distribution were used as cutoffs for identifying significant changes in OTUs between days 5 and 6 (after ciprofloxacin treatment). The OTUs found to have significantly changed were aggregated at the species level. For the top 30 species (15 that increased in abundance and 15 that decreased in abundance after antibiotic treatment) log-transformed normalized values were visualized using the R ggplot package.

### Predictive model of diarrheal disease

We developed and trained a model for predicting disease based on microbiota features. For feature set selection we only used samples prior to infection. For each log_2_-transformed OTU we fit a standard case/control linear model: ${y}_i={b}_o+{b}_1\ast {k}_j+\eta { \log}_2\left(\frac{n{f}_j}{1000}+1\right)+{e}_j$. Here, *y*_*j*_ is the observed log transformed count, *k*_*j*_ is a samples’ categorical phenotype (1 if the patient developed diarrhea, and 0 otherwise), *η* is an OTU-specific estimate of biases in PCR amplification that is fit for each OTU independently as an effect on the samples’ normalization scaling factor, *nf*_*j*_ is the samples’ normalization scaling factor achieved through CSS [40]. We retained OTUs with an absolute log_2_ fold-change greater than 1 for further analysis.

We further reduced the number of OTUs using a linear kernel support vector machine (SVM). Using solely features with an absolute weight greater than 0.03 in the linear kernel (resulting in 32 OTUs) we trained a radial basis kernel SVM as implemented in R package, kernlab and caret on samples at time point -1 (prior to infection) with 10-fold cross validation. We then tested the ability of the model developed on the pre-infection microbiota to predict whether post-infection samples belonged to the patients who developed disease.

## Results

Three males and two females had diarrheal symptoms while four males and three females did not. Subjects who experienced diarrhea were of similar age (33.2 ± 1.4 years) to those without diarrhea (34.3 ± 2.8 years); four of five received the higher ETEC dose. Thus, the symptomatic rates were 27 % for 1x10^6^ CFU and 7 % for 1x10^5^ CFU.

Our sequencing produced 796,915 reads that passed the quality checks. The reads clustered into 148,147 OTUs, of which only 6,423 were detected in more than five samples, or were represented by at least 20 sequences in a single sample, and were included in further analysis. After removing low abundance and low prevalence OTUs, each of the 124 stool samples we analyzed comprised at least 605 reads, with an average of 4,657 reads per sample (median 4,349). The number of OTUs per sample ranged from 58 to 928, with a median of 542 and an average of 522. Each OTU on average contained 90 sequences, ranging from 5, by definition, to 20,996 with a median of 16 sequences.

### Overview of microbiota profile

**Fig. 1:**
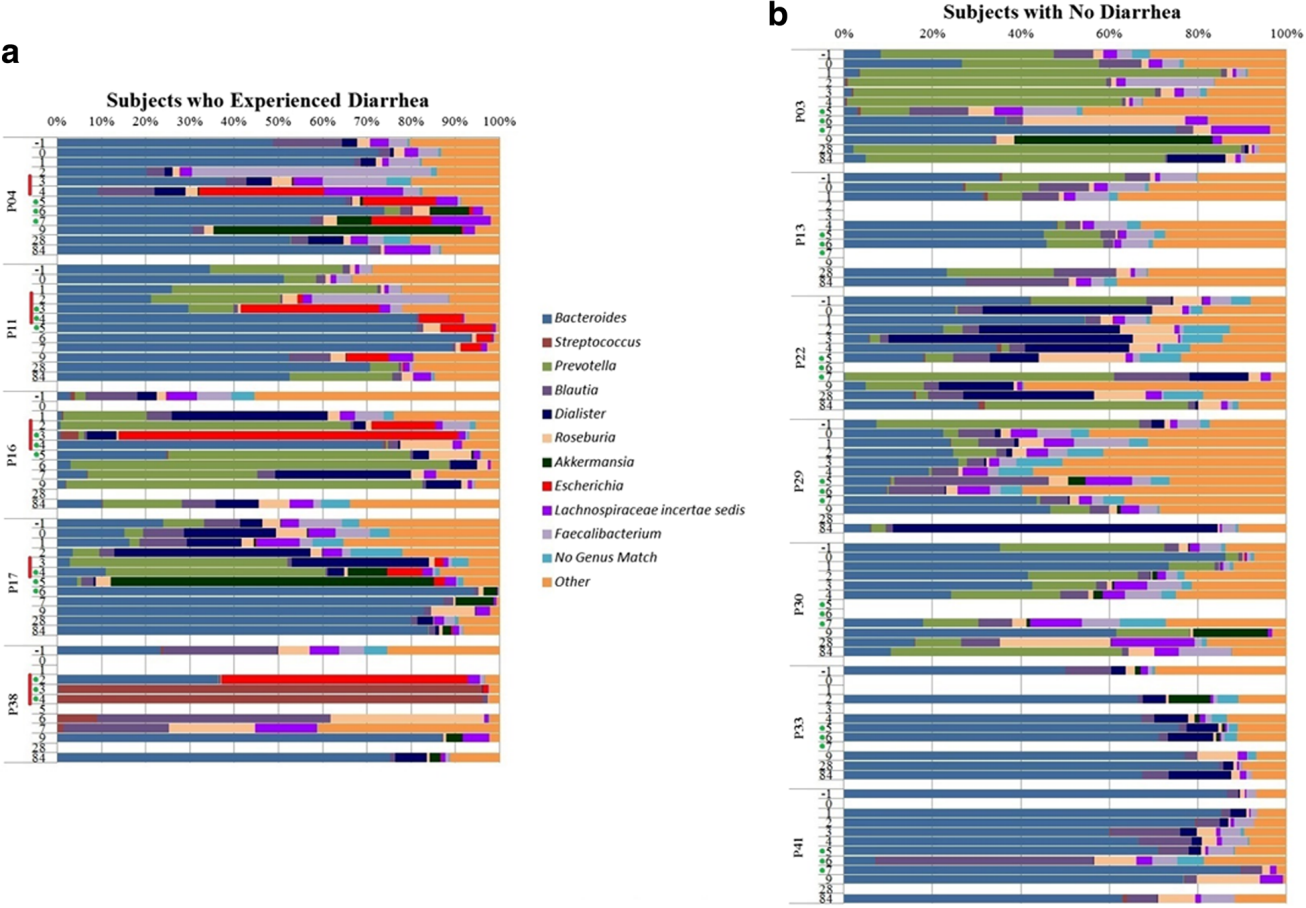
Proportional abundance of the ten most dominant genera in specimens collected from subjects who developed diarrhea (**a**) and subjects who did not (**b**) after challenge with ETEC H10407. Each color represents a different genus. Red marks along the y-axis indicate diarrheal symptoms. Green dots indicate administration of ciprofloxacin

The majority of sequences belonged to the *Bacteroidetes* and *Firmicutes* phyla; lower abundance organisms included *Proteobacteria*, *Actinobacteria*, *Fusobacteria* and *Verrucomicrobia*. The abundance profile of the top 10 genera is shown in Fig. 1 and in Additional file 1: Figures S1 and Additional file 2: Figure S2. Further information about all OTUs is provided in Additional file 3: Table S2 and in .biom format in Additional file 4: File 1.

The number of sequences assigned to the genus *Escherichia* varied substantially across time and was linked to the clinical observations (Fig. 1). Among diarrheal specimens, the maximum proportion of the 16S rRNA gene sequences mapping to *Escherichia* was 76 %. Each of the 17 samples containing more than 1 % *Escherichia* sequences were collected during diarrheal episodes or on the days following ciprofloxacin treatment in patients with diarrhea. After three days of ciprofloxacin treatment, *Escherichia* could not be detected in three of the patients who had diarrhea, but was detected in the other two at 14 % and 9 % of the total bacterial population.

### Assessment of *Escherichia* abundance by quantitative culture, quantitative PCR, and 16S rRNA gene sequencing

Quantitative culture and qPCR targeting the heat-labile toxin (LT) gene were performed using stool specimens collected between days 0 and 4 of the study, before the administration of ciprofloxacin (see Additional file 5: Table S1). Quantitative culture data was available for 44 stools surveyed by 16S rRNA gene sequencing; eight originated from diarrheal specimens. The presence of ETEC H10407 was detected by quantitative culture in eight of eight (100 %) diarrheal stools and thirteen of 36 (36 %) non-diarrheal stools. The maximum abundance was 3.1x10^9^ CFU per gram of stool. Quantitative PCR for the LT gene was available for 46 samples, 9 of which originated from diarrheal samples. The challenge strain was detected by qPCR in all diarrheal stools and in 21 of 37 (57 %) non-diarrheal stools. Thirteen samples (9 symptomatic and 4 asymptomatic) exceeded the 1.4x10^4^ copies of the LT gene threshold, proposed by Lindsay et al. [36].

**Fig. 2:**
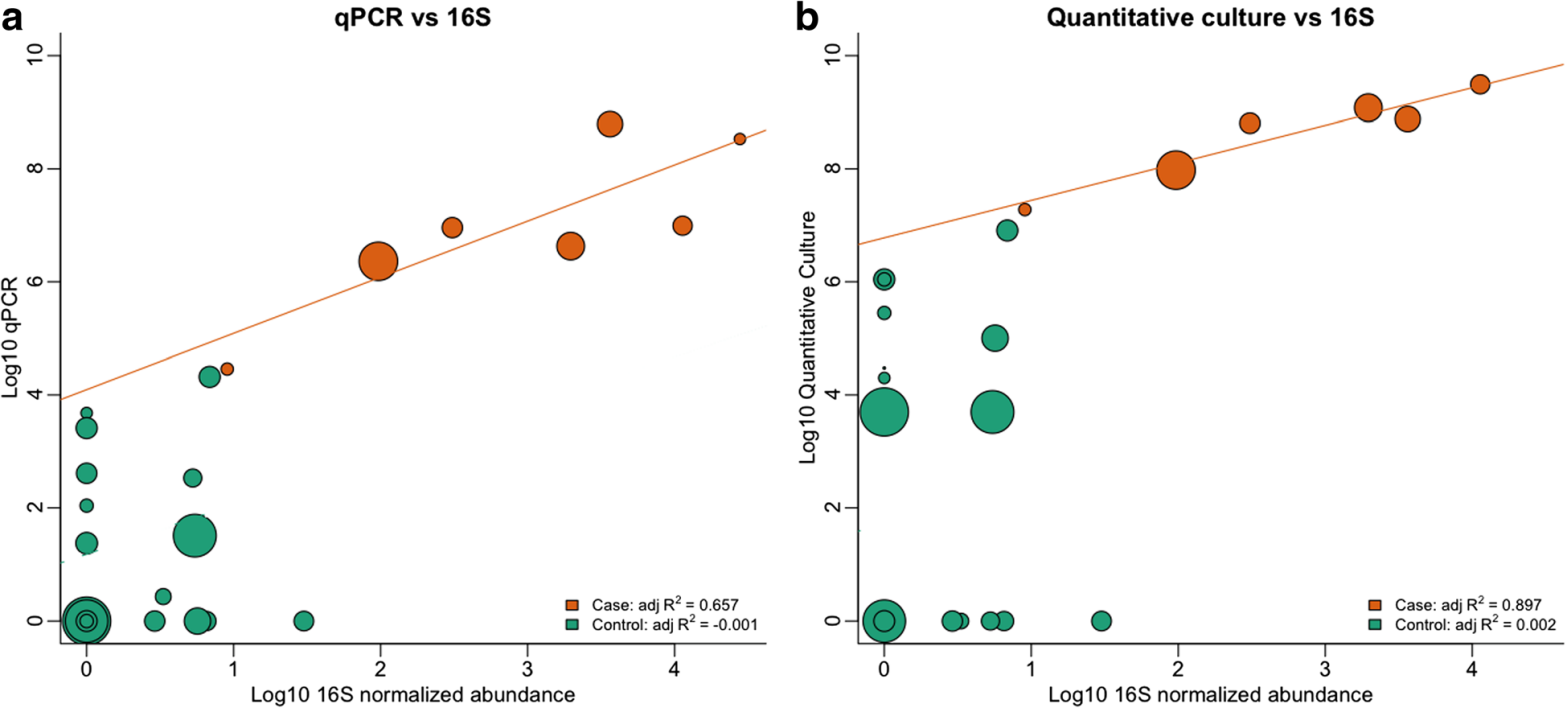
Spearman correlation between 16S rRNA normalized gene count abundances assigned to *Escherichia* (x-axis) and either (**a**) qPCR LT or (**b**) quantitative culture. All axes are on log_10_ scale. The size of each of the circles is proportional to the total number of reads in the sample. Orange and green circles represent diarrheal and non-diarrheal samples, respectively. Orange lines are the linear correlation in stools from patients with diarrhea. Correlation between 16S rRNA gene counts of *Escherichia* and quantitative culture estimates and correlation between 16S rRNA gene counts and qPCR LT estimates are high in diarrheal samples, when *E. coli* is abundant. In non-diarrheal samples, the correlation is low

The proportional abundance of *Escherichia* estimated through 16S rRNA gene sequencing correlated with the abundance estimated by culture and qPCR. The proportional abundance of 16S rRNA gene reads from *Escherichia* OTUs ranged from 0 to 76 %. In diarrheal samples, the correlation between 16S rRNA gene counts of *Escherichia* and quantitative culture was adjusted R^2^ = 0.89, *p* = 0.0002 and for qPCR, was adjusted R^2^ = 0.66, *p* = 0.005, suggesting the OTUs identified in these samples corresponded to the challenge strain (Fig. 2). In non-diarrheal samples, the 16S rRNA gene counts were not correlated with either measure. The limit of detection of the 16S rRNA gene survey assay was estimated to be 10^7^ CFU/g of stool for an average sequencing depth of 6,000 reads.

### Inter-patient Bray Curtis distances were significantly larger than intra-patient distances

**Fig. 3:**
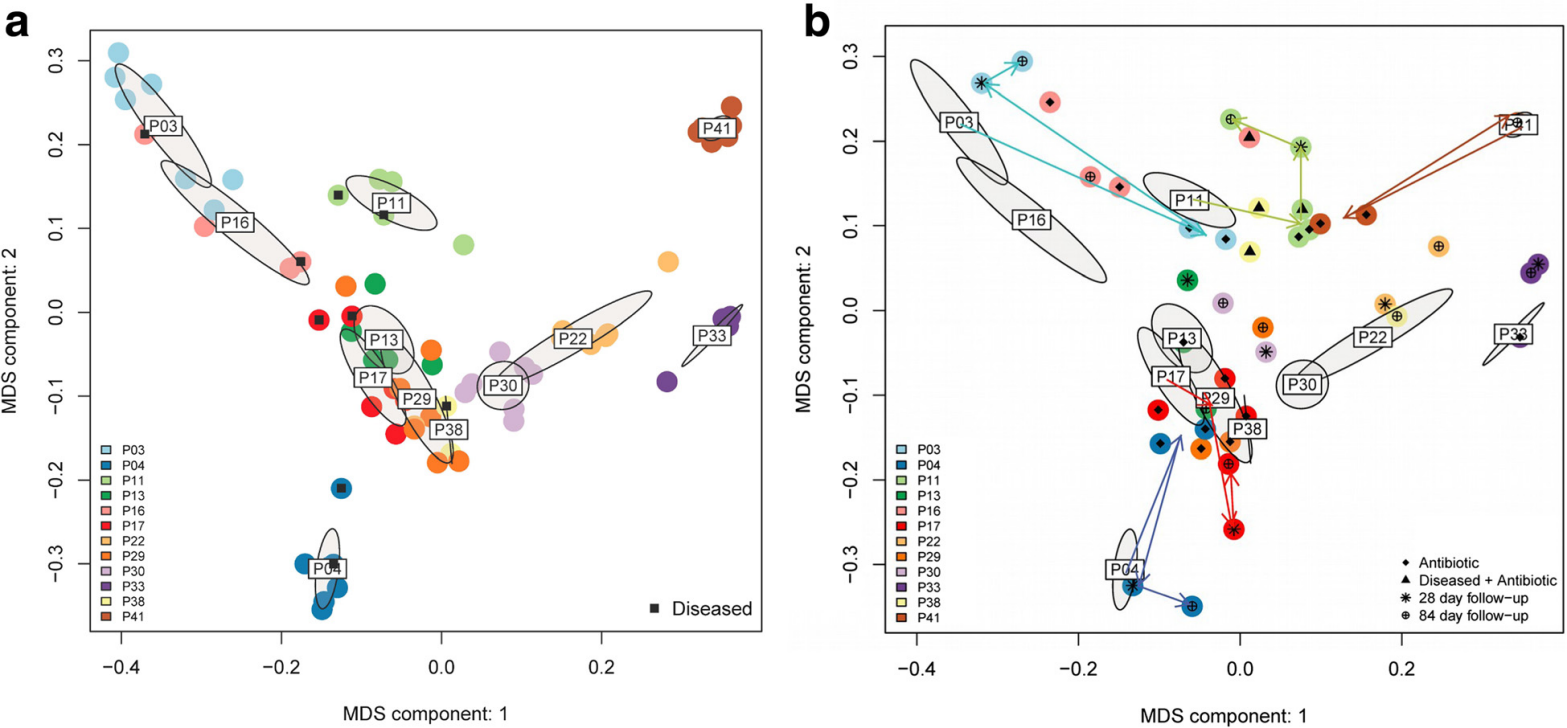
Samples cluster by patient not phenotype in PCoA plots. **a** Samples pre-ciprofloxacin treatment. All data points are shown, with black squares highlighting samples obtained during diarrheal episodes. **b** Samples after antibiotic treatment. Pre-treatment samples summarized as 95 % confidence ellipses (ordiellipse function from the R vegan package). Post-treatment samples shown as points with symbols indicating whether they were derived immediately post-antibiotic treatment, or at 1 and 3 month follow ups. Shifts introduced by antibiotic treatment, and transition to post study microbiota highlighted for selected patients with colored arrows

The inter-personal variability in the distal gut microbiota exceeded the effect of perturbations (Fig. 3), as evidenced by smaller distances found between the microbiota of samples from the same patient versus samples compared across different patients (Bray Curtis distance, *p* = 8x10^−26^). Diarrheal episodes generally did not induce large shifts (labeled points in Fig. 3a). In contrast, in certain patients antibiotic treatment by itself or combined with disease induced large shifts in the microbiota, but this was not consistent, and even the highly perturbed microbiota profiles tended to return to close to the pre-treatment state at 28 and 84 day follow-ups (arrows Fig. 3b).

### Changes in microbial diversity during the challenge study and subsequent ciprofloxacin treatment

**Fig. 4:**
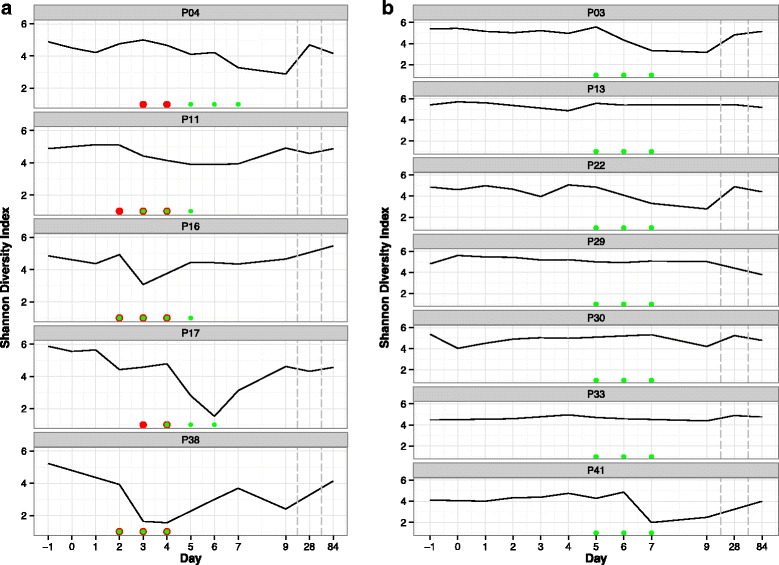
Shannon measure of observed bacterial diversity across time for each of the individuals with diarrheal episodes (**a**) and without (**b**). Red circles indicate diarrheal episodes. Green circles indicate antibiotic treatment

The diversity of the stool microbiota (Fig. 4), as measured by the Shannon diversity index, was highly variable across all individuals. Both diarrhea [20] and ciprofloxacin treatment [21, 24] led to significant decreases in overall diversity (*p* < 0.05, rank-sum Wilcoxon test) but neither the onset of symptoms nor administration of antibiotics produced the same consequences in all individuals. Overall diversity of the gut microbiota was largely restored in all patients by follow-up visits at days 28 and 84. Ciprofloxacin treatment appeared to have a more severe impact on overall diversity than diarrheal disease. Excluding *Escherichia,* six of the top seven most frequently occurring genera (*Bacteroides, Streptococcus, Prevotella, Dialister, Akkermansia, Blautia)* had changes of over 30 % of normalized sequences in different individuals (see Additional file 1: Figures S1 and Additional file 2: Figure S2), most of these extreme changes occurred after the administration of the antibiotic (10 increases pre and 23 increases post initial antibiotic, binomial *p* = 0.016). However the effects of diarrhea and ciprofloxacin cannot be easily disentangled given that patients were treated after they developed severe diarrheal symptoms. All five cases and five of seven controls exhibited large fluctuations in abundance within individual genera.

### Fine-level reorganization of specific taxonomic groups upon ciprofloxacin treatment

**Fig. 5:**
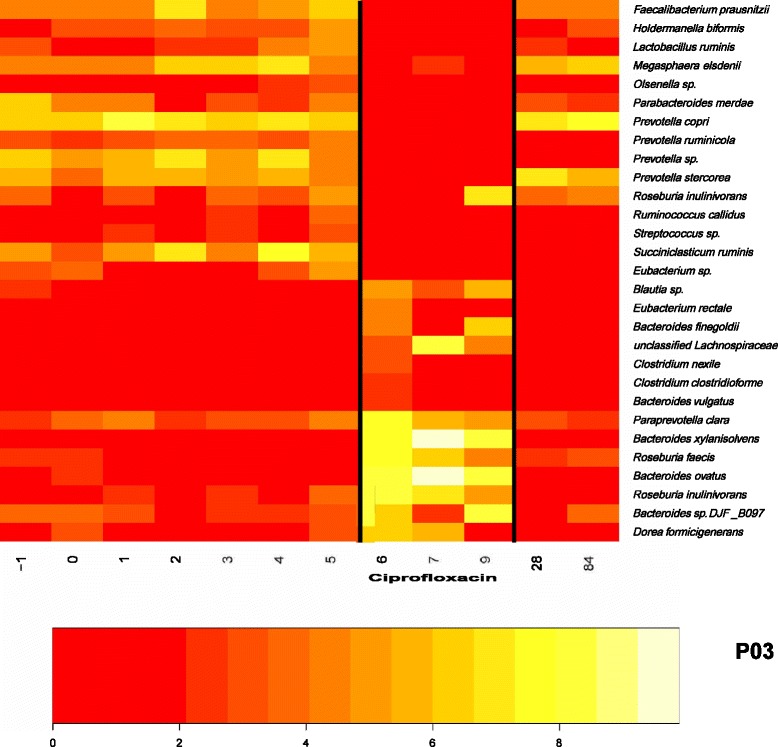
Shift of microbial community structure after ciprofloxacin treatment (administered on days 5, 6, 7 [labels on the x-axis]) for subject P03. Several OTUs, primarily *Prevotella* and *Faecalibacterium*, decreased below the limit of detection after antibiotic treatment but regained their abundance by the 1 month follow-up. The scale is the log of the normalized abundance

Our OTU-level analysis of changes in the microbiota after ciprofloxacin treatment revealed a striking reorganization of certain taxonomic groups. Some taxa were detected before and after but not during ciprofloxacin treatment, while other taxa were detected only during ciprofloxacin treatment. Figure 5 shows sets of OTUs from one individual (results from other controls are shown in Additional file 6: Figure S3). The first panel (largely clustered towards the top of the figure) are OTUs present in the pre-infection (days -1, 0) and pre-treatment (days 1–5) microbiota but which disappear below the level of detection upon antibiotic treatment, then reemerge at days 28 and 84. A second group of OTUs (bottom of the figure) is most abundant after antibiotic treatment.

### Specific taxonomic changes associated with diarrheal disease

Patients who eventually developed diarrheal disease had a higher proportional abundance of OTUs from the genus *Escherichia* as well as *Bacteroides dorei, Bacteroides ovatus,* and *Barnesiella intestinihominis*. In contrast, the microbiota of controls were enriched in OTUs from the species *Bacteroides vulgatus, Bacteroides xylanisolvens,* and *Parabacteroides distasonis*. By restricting our analysis to just the time-points prior to the development of disease, *Bacteroides dorei* and to a lesser extent *Barnesiella intestinihominis* remained associated with the cases, while in controls only *Bacteroides vulgatus* remained significantly enriched. See Additional file 3: Tables S2 and Additional file 7: Table S3 for full details.

### The pre-infection microbiota – a predictor of disease onset?

**Fig. 6:**
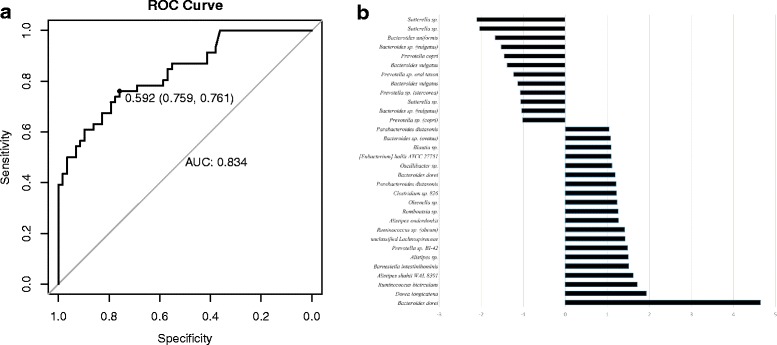
Composition of biomarker that predicts eventual disease. **a** Receiver operating characteristic (ROC) curve for the SVM model. The highlighted point represents the best trade-off between sensitivity and specificity. **b** X-axis represents log2 fold change of the abundance of individual OTUs between health and disease, negative fold changes indicating association with health (patients with asymptomatic infection)

To further explore whether pre-disease microbiota could be predictive of the eventual outcomes, we identified a 'biomarker' comprising 32 bacterial OTUs (See Fig. 6). A radial basis kernel SVM model with an AUROC of 0.83 based on this biomarker achieved 76 % accuracy in predicting whether a sample originated from a patient who eventually developed diarrhea. The most robust predictors of disease development (as judged by a visual inspection of their prevalence and abundance in the pre-infection microbiota) included *Bacteroides dorei*, *Prevotella* sp., *Alistipes onderdonkii, Bacteroides* sp. (ovatus), and *Blautia* sp., while the predictors of resistance against diarrheal disease included *Sutterella* sp., *Prevotella copri*, and *Bacteroides vulgatus*. Full information is provided in Additional file 8: Table S4.

## Discussion

Challenging the intestinal microbiota with ETEC has two expected outcomes: either invasion is successful, resulting in colonization and induction of disease, or the invaders are repelled, and the health of the human host is maintained. Our study focused on changes in microbiota during an ETEC challenge. Despite the small sample size and the large variations among individuals, novel trends emerge that may lead to firmer conclusions. We demonstrated that the proportional abundance of 9 to 76 % *E. coli* 16S rRNA gene sequences in diarrheal samples was associated with successful invasion by ETEC, confirmed by both quantitative culture and qPCR.

There is growing interest in bolstering resistance to infections by altering the microbiota. Previous studies have attempted to correlate microbiota patterns with prevention or development of gastrointestinal pathogens such as *Shigella* [43], *Clostridium difficile* [44] and vancomycin resistant enterococci [45], and have identified candidate species that could be used to enhance defense against these pathogens [44, 46]. Similarly, our study revealed associations between the pre-challenge microbiota and the risk of symptoms following challenge with ETEC. Our biomarker of 32 OTUs predicted with reasonable accuracy whether an individual would develop diarrhea or not. This observation warrants further study, since the 12 OTUs that were associated with not developing diarrheal disease may be taxa that prevent the growth of ETEC; if so, these might have potential therapeutic uses as novel probiotics. Although little is currently known about the impact of our candidate taxa on pathogenic microbes, *Prevotella copri* was recently anti-correlated with diarrheal disease in infant cohorts from low income countries [20] and *Bacteroides* spp. have been linked to resolution of *C. difficile* diarrheal disease following fecal microbiota transplant [47].

After the ETEC challenge, the patients were treated with ciprofloxacin. After treatment, antibiotic resistant strains, which may already be present at low abundance under normal conditions, replace the susceptible organisms killed by the drug. However, the sensitive strains may not be entirely eradicated, allowing their numbers to recover post-treatment. Such dynamics were apparent in some of the samples. Co-existence of sensitive and resistant strains in the same sample in the face of opposite primary selective pressure has potential implications for understanding ecological balance and treatments for disease. It may be that rare forms (species, taxa) have a selective advantage simply because they are rare (frequency-dependent selection) even in the face of strong directional selection such as that imposed by antibiotics.

Our results confirm those of Dethlefsen et al. [21] and others: inter-individual variation is greater than intra-individual variation following ciprofloxacin treatment, and the microbiota tends to recover within a few weeks. Impact on the gut microbiota depends on the duration of the course and degree of resistance in the community, consistent with a previous observation that the structure of the gut microbiota is determined by long-term dietary habits and is less affected by short term perturbations [17]. These observations suggest that short-term, single-course use of ciprofloxacin for infections is unlikely to result in long-term and large-scale microbiota perturbations.

## Conclusions

One of the clearest conclusions from our study is that, despite the major measurable perturbations, each individual’s microbiota maintained its overall uniqueness and its general community structure. As such, our study leads to hypotheses about the microbiota rather than firm conclusions. Nonetheless, we believe the relatively large amount of variation between the microbiota of individuals should be seen as an asset rather than a limitation. Keystone species, and critical interactions between community members, can only be teased out from possible confounding factors if there are sufficient inter-personal differences between microbiota communities. Our study, therefore, provides a first indication of the microbial taxa that may prevent the colonization of the human intestinal tract by ETEC in American volunteers not previously exposed to the pathogen. Our results suggest a candidate list of potential probiotics that may be successful in the prophylaxis of traveler's diarrhea.

## Abbreviations

BLAST, basic alignment search tool; CFU, colony forming units; DNA; deoxyribonucleic acid; ETEC, enterotoxigenic *Escherichia coli;* LT, heat-labile toxin; NCBI, National center for Biotechnology Information; OTU, operational taxonomic unit; PCR, polymerase chain reaction; qPCR, quantitative polymerase chain reaction; RNA, ribonucleic acid; rRNA, ribosomal RNA.

## Additional files

Additional file 1: Figure S1. Proportional abundance of the 9 most abundant genera within patients who developed diarrhea. (JPG 282 kb) Additional file 2: Figure S2. Proportional abundance of the 9 most abundant genera within patients who did not develop diarrhea. (JPG 299 kb) Additional file 3: Table S2. Summary information about OTUs: number of sequences, number of samples, taxonomic annotation. (DOCX 1050 kb) Additional file 4: BIOM formatted data underlying the results of our study. (BIOM 7422 kb) Additional file 5: Table S1. Quantitative measurements of *E. coli* presence in the samples (quantitative culture, qPCR, 16S rRNA gene). (DOCX 27 kb) Additional file 6: Figure S3. Heatmap of the 30 OTUs most impacted by antibiotic treatment for each of the 7 patients who did not develop diarrhea. (PDF 196 kb) Additional file 7: Table S3. Differentially abundant OTUs across multiple conditions. (DOCX 344 kb) Additional file 8: Table S4. Composition of pre-infection biomarker. (XLSX 23 kb)
