# Supplementary figures and images for: Individual-specific changes in the human gut microbiota after challenge with enterotoxigenic Escherichia coli and subsequent ciprofloxacin treatment
Source: BMC Genomics. 2016 Jun 8;17:440. doi: 10.1186/s12864-016-2777-0 (PMC4898365; doi:10.1186/s12864-016-2777-0)

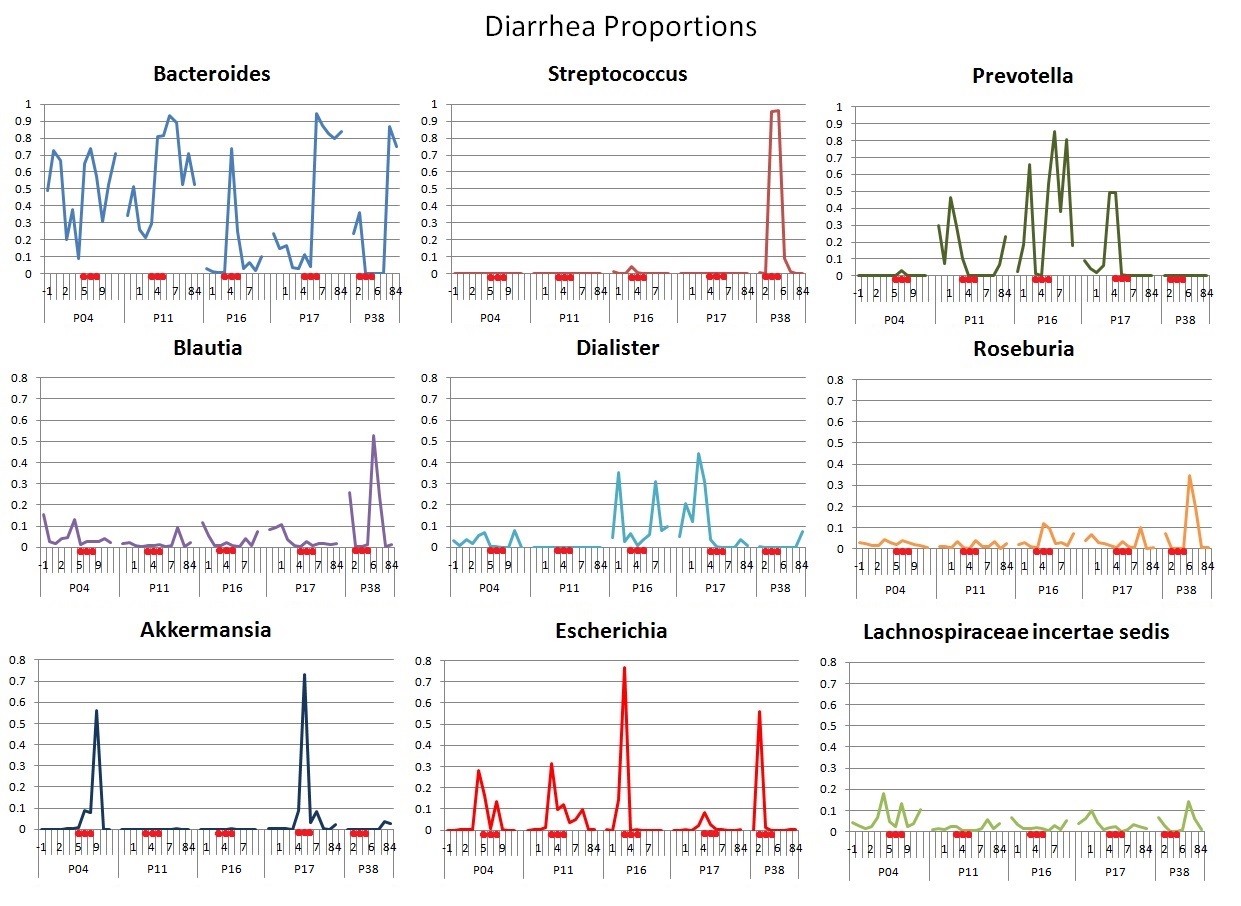

Supplement: Additional file 1: Figure S1. — Proportional abundance of the 9 most abundant genera within patients who developed diarrhea. (JPG 282 kb) [file 12864_2016_2777_MOESM1_ESM.jpg]

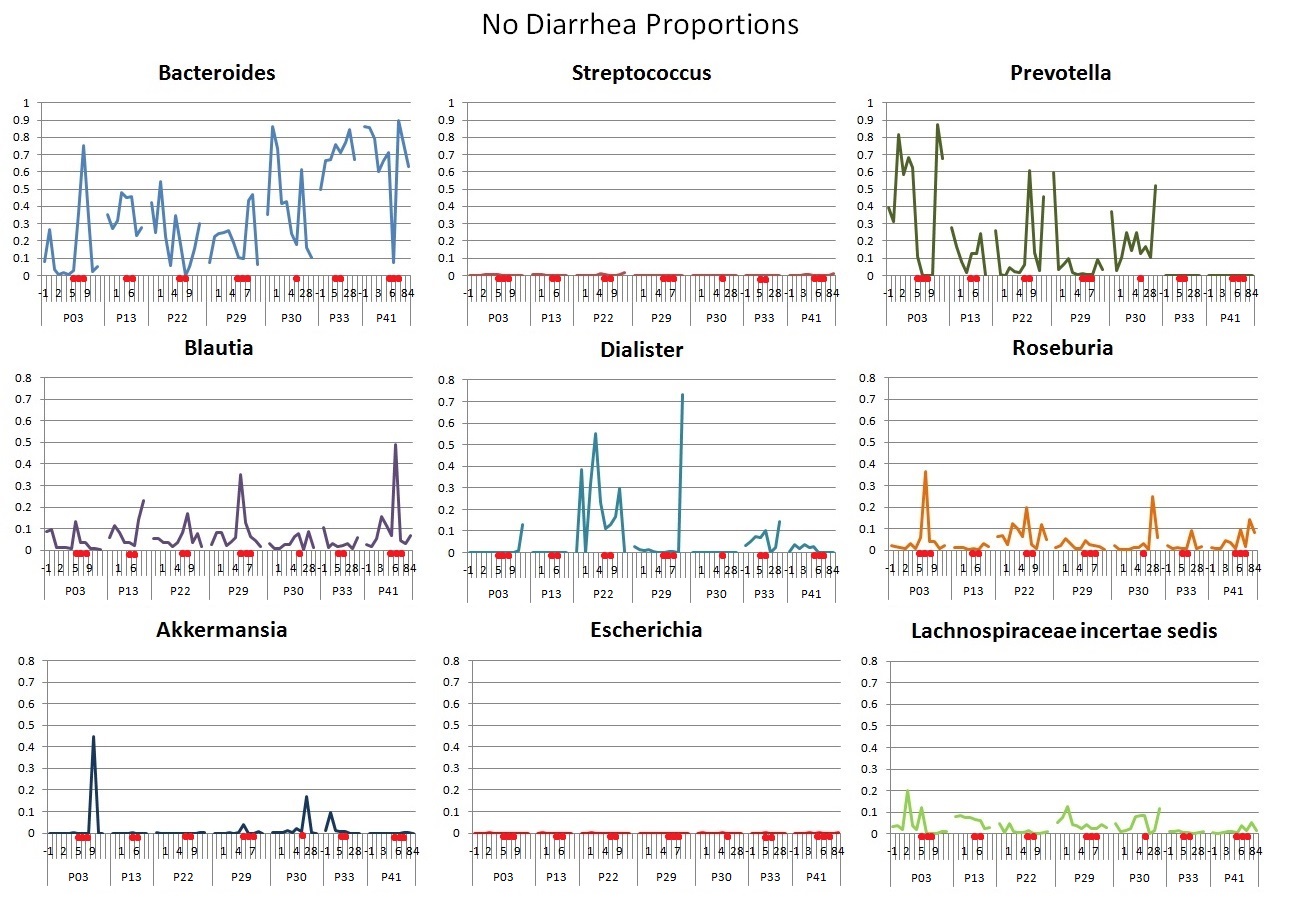

Supplement: Additional file 2: Figure S2. — Proportional abundance of the 9 most abundant genera within patients who did not develop diarrhea. (JPG 299 kb) [file 12864_2016_2777_MOESM2_ESM.jpg]

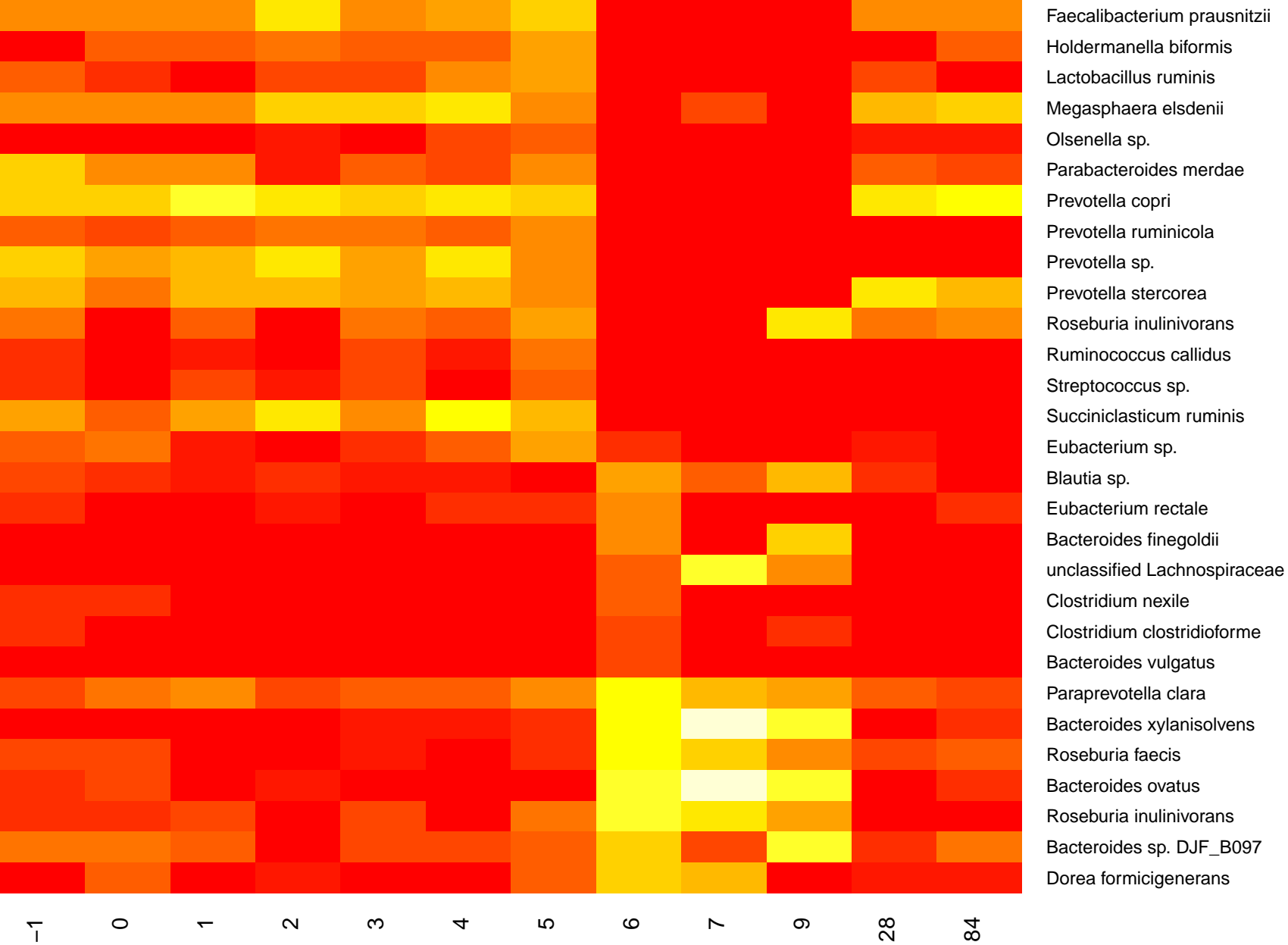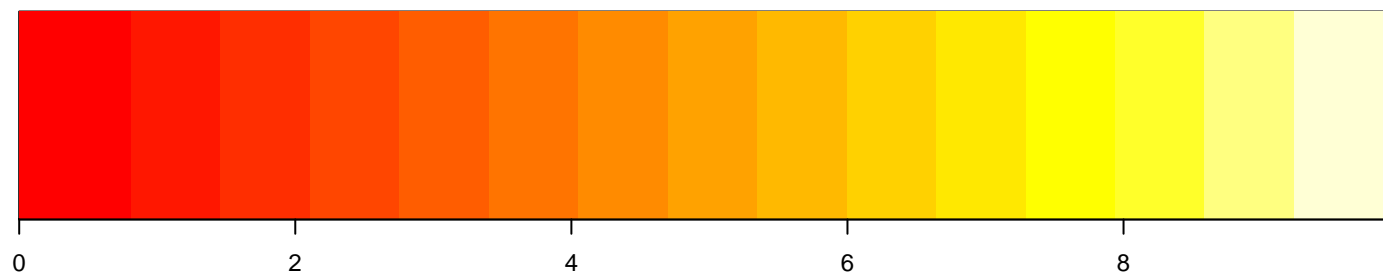

**P03**

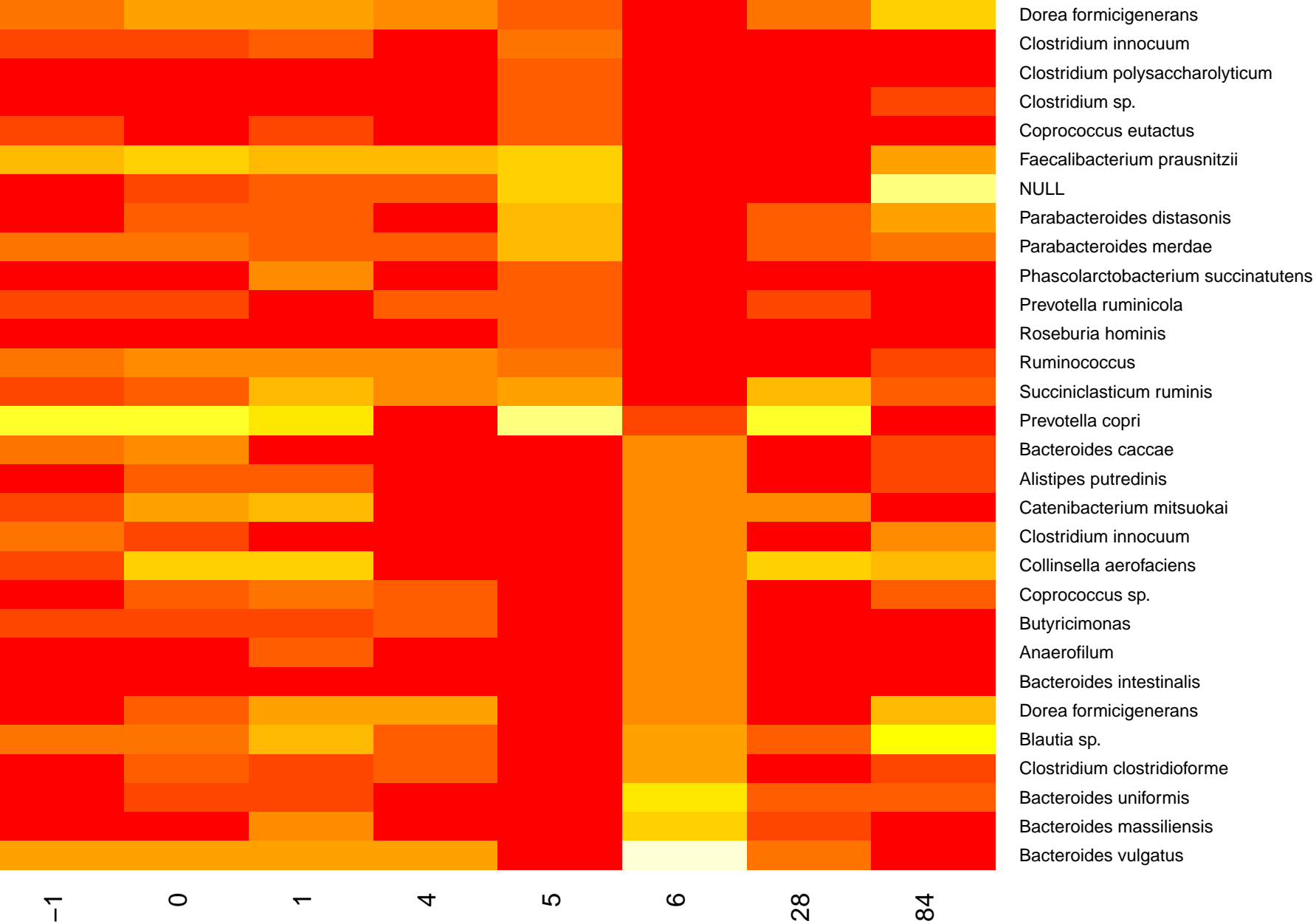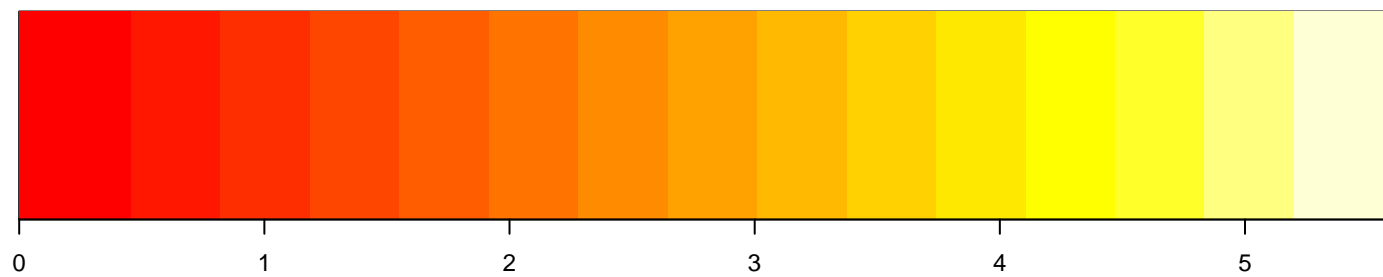

**P13**

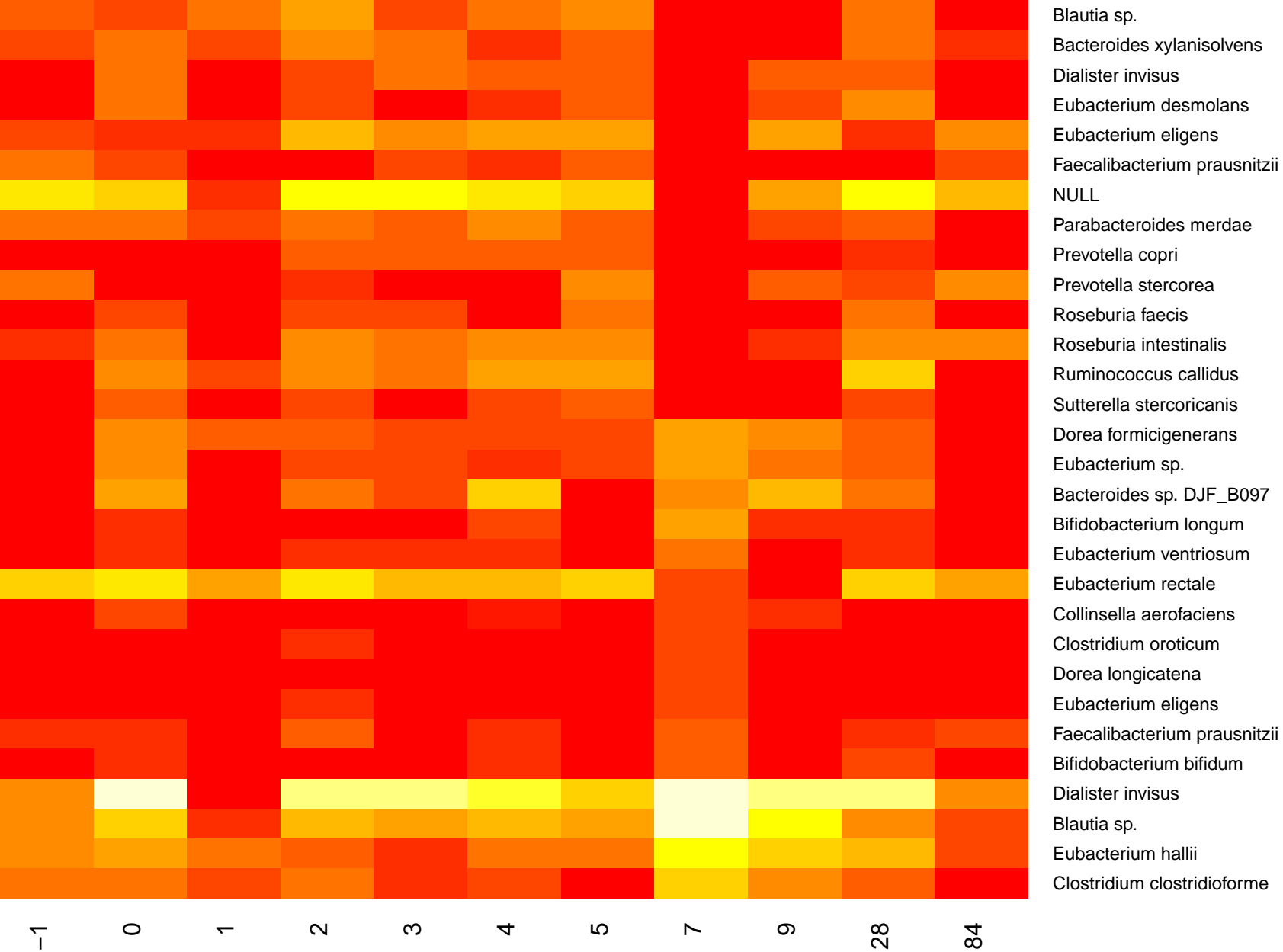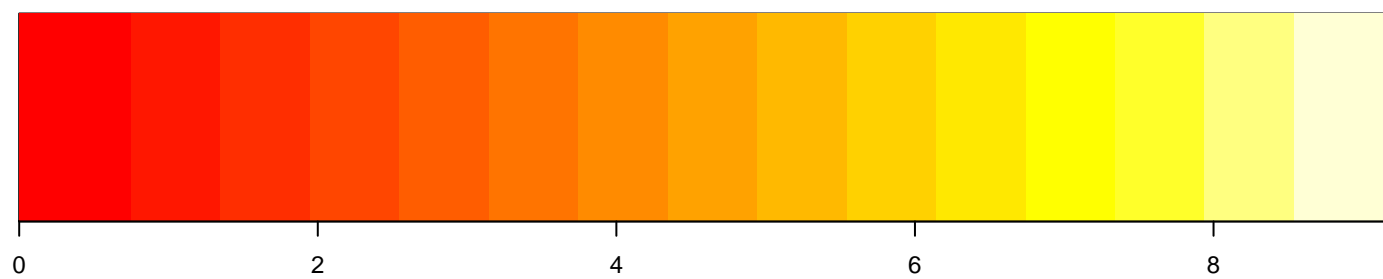

**P22**

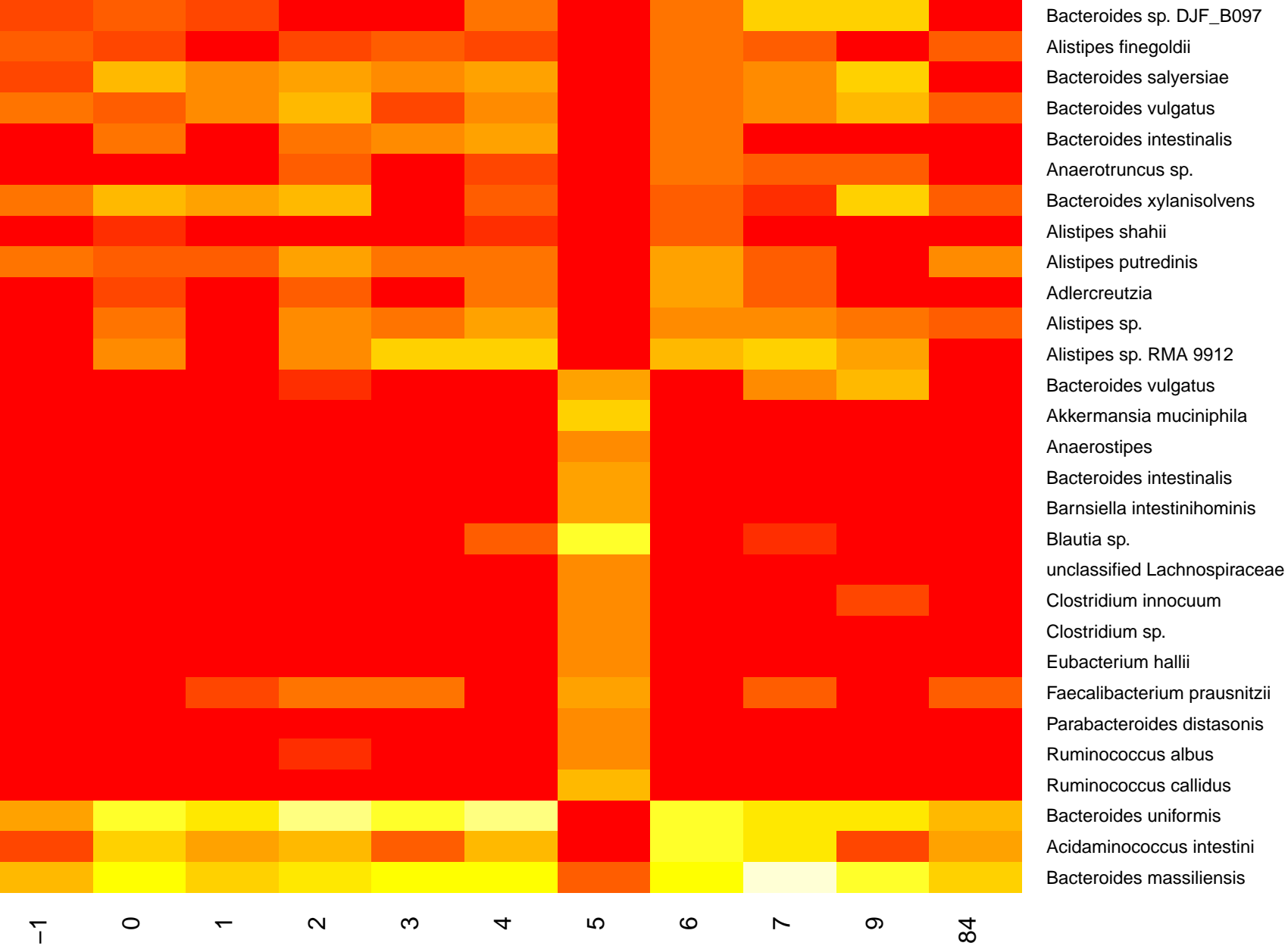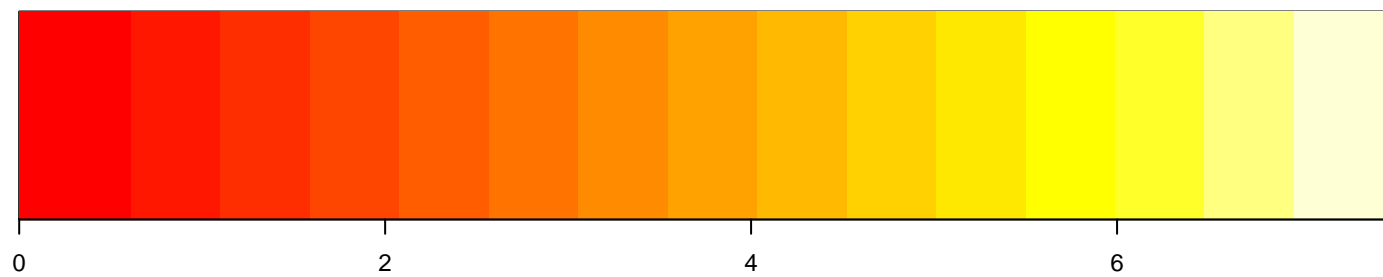

**P29**

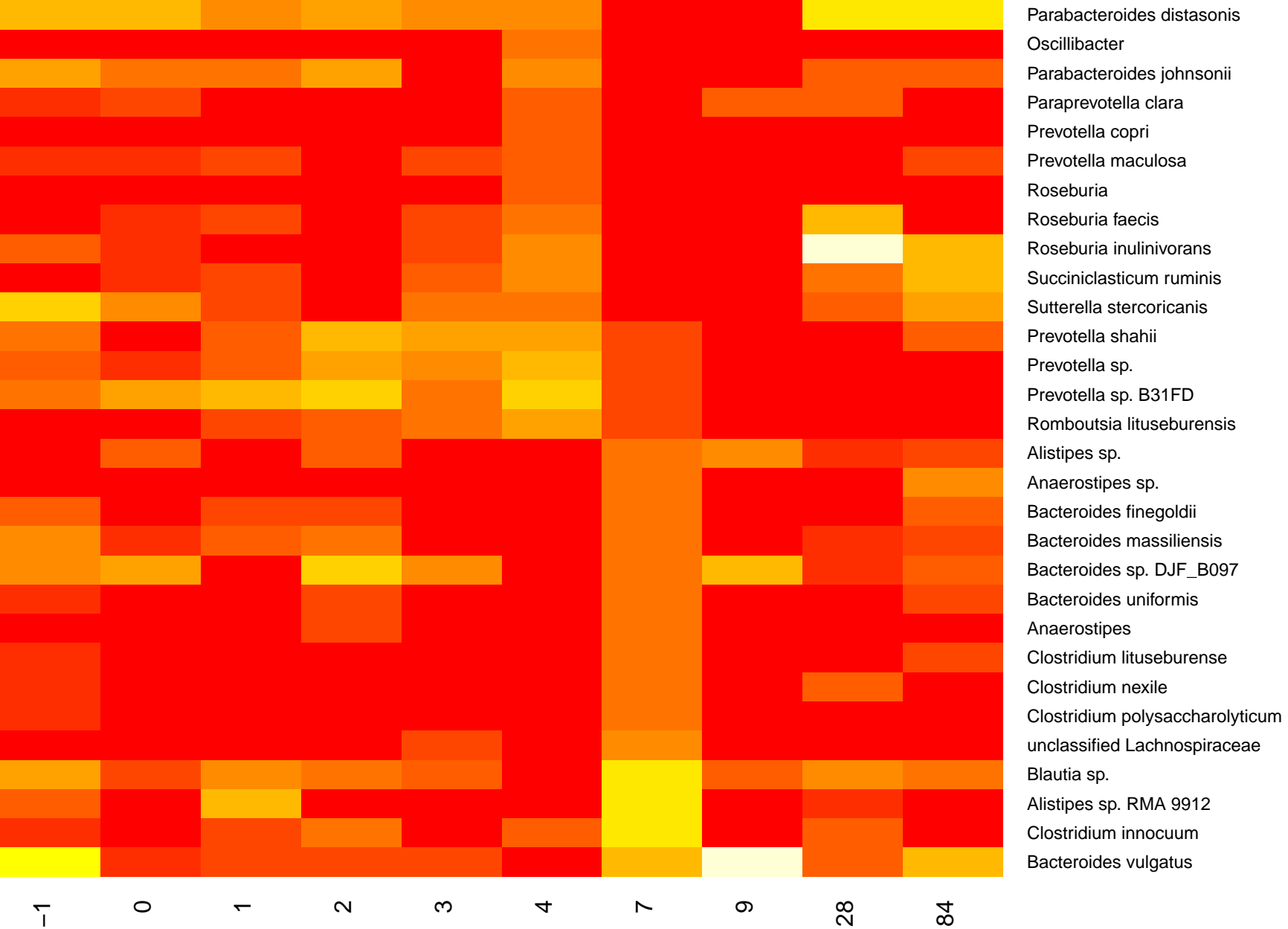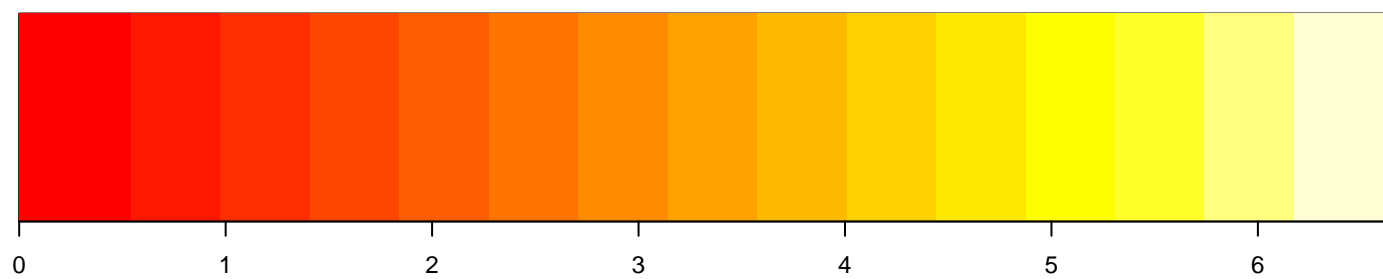

**P30**

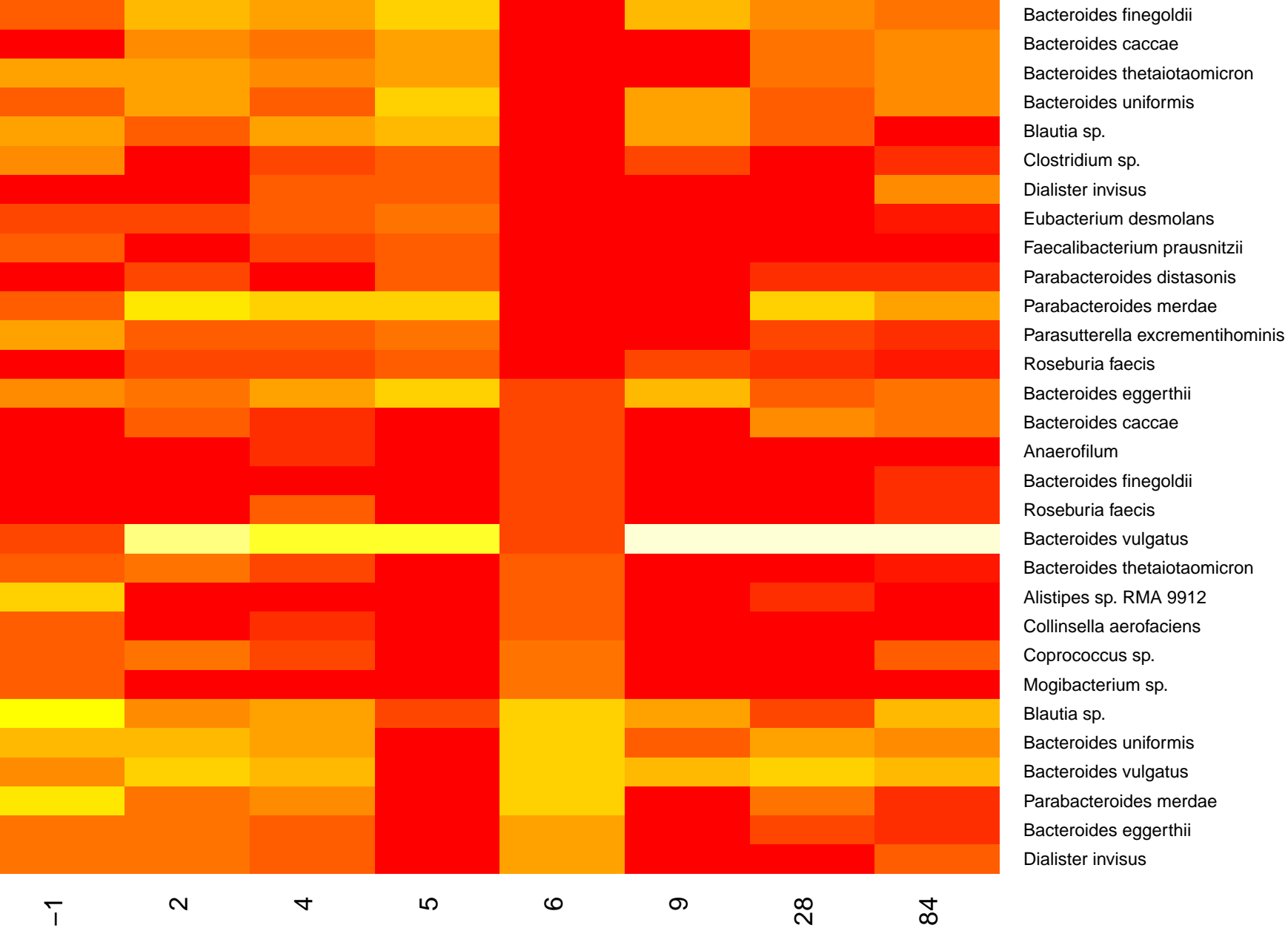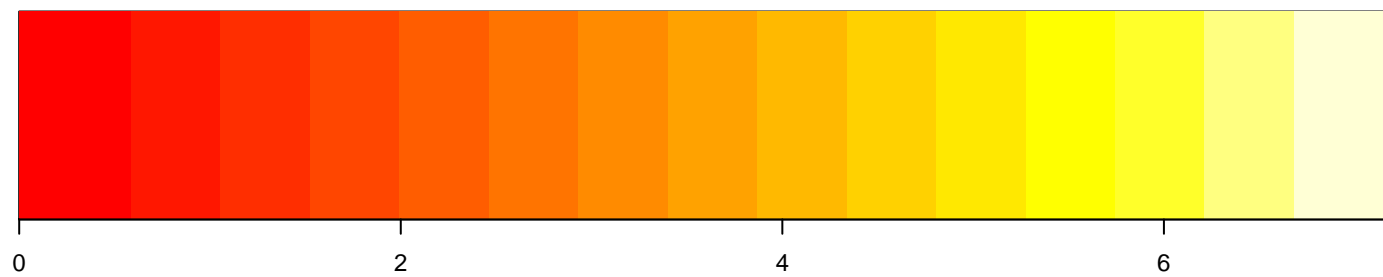

**P33**

Supplement: Additional file 6: Figure S3. — Heatmap of the 30 OTUs most impacted by antibiotic treatment for each of the 7 patients who did not develop diarrhea. (PDF 196 kb) [file 12864_2016_2777_MOESM6_ESM.pdf]
