# Supplementary material for: Individual-specific changes in the human gut microbiota after challenge with enterotoxigenic Escherichia coli and subsequent ciprofloxacin treatment
Source: BMC Genomics. 2016 Jun 8;17:440. doi: 10.1186/s12864-016-2777-0 (PMC4898365; doi:10.1186/s12864-016-2777-0)
Supplement: Additional file 3: Table S2. — Summary information about OTUs: number of sequences, number of samples, taxonomic annotation. (DOCX 1050 kb) [file 12864_2016_2777_MOESM3_ESM.docx]

Table s2

| **OTU ID** | **Center sequence ID** | **Number of sequences** | **Number of samples** | **Phylum** | **Class** | **Order** | **Family** | **Genus** | **Strain** |
| --- | --- | --- | --- | --- | --- | --- | --- | --- | --- |
| 3 | 130_6133 | 20 | 12 | Bacteroidetes | Bacteroidia | Bacteroidales | Bacteroidaceae | Bacteroides | Bacteroides vulgatus |
| 5 | 14_1391 | 9 | 7 | Bacteroidetes | Bacteroidia | Bacteroidales | Bacteroidaceae | Bacteroides | Bacteroides uniformis |
| 10 | 111_7283 | 13 | 6 | Bacteroidetes | Bacteroidia | Bacteroidales | Bacteroidaceae | Bacteroides | Bacteroides uniformis |
| 11 | 130_1817 | 9 | 8 | Bacteroidetes | Bacteroidia | Bacteroidales | Bacteroidaceae | Bacteroides | Bacteroides vulgatus |
| 13 | 82_8248 | 32 | 10 | Bacteroidetes | Bacteroidia | Bacteroidales | Bacteroidaceae | Bacteroides | Bacteroides ovatus |
| 14 | 131_3769 | 10 | 5 | Verrucomicrobia | Verrucomicrobiae | Verrucomicrobiales | Akkermansiaceae | Akkermansia | Akkermansia muciniphila |
| 21 | 105_4167 | 18 | 5 | Bacteroidetes | Bacteroidia | Bacteroidales | Bacteroidaceae | Bacteroides | Bacteroides ovatus |
| 40 | 6_3606 | 8 | 7 | Firmicutes | Clostridia | Clostridiales | Lachnospiraceae | Blautia | Blautia |
| 42 | 32_610 | 6 | 6 | Firmicutes | Clostridia | Clostridiales | Lachnospiraceae | Dorea | Dorea longicatena |
| 59 | 68_10741 | 65 | 11 | Proteobacteria | Gammaproteobacteria | Enterobacteriales | Enterobacteriaceae | Escherichia/Shigella | Escherichia/Shigella |
| 65 | 1_2768 | 16 | 15 | Bacteroidetes | Bacteroidia | Bacteroidales | Porphyromonadaceae | Parabacteroides | Parabacteroides merdae |
| 69 | 55_3816 | 5 | 5 | Firmicutes | Clostridia | Clostridiales | Lachnospiraceae | Lachnospiraceae incertae sedis | Lachnospiraceae incertae sedis |
| 71 | 105_5541 | 10 | 5 | Bacteroidetes | Bacteroidia | Bacteroidales | Bacteroidaceae | Bacteroides | Bacteroides ovatus |
| 81 | 82_4269 | 50 | 6 | Bacteroidetes | Bacteroidia | Bacteroidales | Bacteroidaceae | Bacteroides | Bacteroides ovatus |
| 82 | 11_772 | 15 | 5 | Firmicutes | Clostridia | Clostridiales | Lachnospiraceae | Blautia | Blautia |
| 83 | 71_2231 | 5 | 5 | Firmicutes | Negativicutes | Selenomonadales | Veillonellaceae | Dialister | Dialister invisus |
| 84 | 32_6019 | 26 | 16 | Firmicutes | Clostridia | Clostridiales | Ruminococcaceae | Faecalibacterium | Faecalibacterium prausnitzii |
| 100 | 81_966 | 10 | 5 | Bacteroidetes | Bacteroidia | Bacteroidales | Prevotellaceae | Prevotella | Prevotella copri |
| 101 | 12_931 | 37 | 15 | Firmicutes | Clostridia | Clostridiales | Lachnospiraceae | Roseburia | Roseburia intestinalis |
| 117 | 54_6086 | 9 | 6 | Bacteroidetes | Bacteroidia | Bacteroidales | Prevotellaceae | Prevotella | Prevotella copri |
| 118 | 104_271 | 6 | 6 | Bacteroidetes | Bacteroidia | Bacteroidales | Prevotellaceae | Prevotella | Prevotella copri |
| 142 | 6_793 | 8 | 6 | Firmicutes | Clostridia | Clostridiales | Ruminococcaceae | Ruminococcus | Ruminococcus faecis |
| 157 | 112_4690 | 7 | 5 | Bacteroidetes | Bacteroidia | Bacteroidales | Prevotellaceae | Prevotella | Prevotella copri |
| 159 | 128_3532 | 6 | 5 | Firmicutes | Negativicutes | Selenomonadales | Veillonellaceae | Dialister | Dialister invisus |
| 172 | 125_5159 | 6 | 5 | Firmicutes | Clostridia | Clostridiales | Ruminococcaceae | Ruminococcus | Ruminococcus albus |
| 222 | 88_902 | 10 | 5 | Firmicutes | Clostridia | Clostridiales | Lachnospiraceae | Blautia | Blautia |
| 237 | 104_178 | 5 | 5 | Bacteroidetes | Bacteroidia | Bacteroidales | Prevotellaceae | Prevotella | Prevotella copri |
| 240 | 95_984 | 37 | 6 | Firmicutes | Clostridia | Clostridiales | Ruminococcaceae | Ruminococcus | Ruminococcus bromii |
| 272 | 117_499 | 11 | 8 | Firmicutes | Negativicutes | Selenomonadales | Veillonellaceae | Dialister | Dialister invisus |
| 331 | 1_2303 | 5 | 5 | Firmicutes | Clostridia | Clostridiales | Lachnospiraceae | Blautia | Blautia |
| 364 | 23_94 | 13 | 10 | Bacteroidetes | Bacteroidia | Bacteroidales | Bacteroidaceae | Bacteroides | Bacteroides vulgatus |
| 365 | 91_5120 | 7 | 6 | Bacteroidetes | Bacteroidia | Bacteroidales | Bacteroidaceae | Bacteroides | Bacteroides ovatus |
| 368 | 111_2522 | 11 | 7 | Firmicutes | Clostridia | Clostridiales | Lachnospiraceae | Lachnospiraceae incertae sedis | Lachnospiraceae incertae sedis |
| 388 | 18_2021 | 10 | 7 | Firmicutes | Clostridia | Clostridiales | Ruminococcaceae | Subdoligranulum | Subdoligranulum |
| 393 | 107_3840 | 8 | 6 | Bacteroidetes | Bacteroidia | Bacteroidales | Bacteroidaceae | Bacteroides | Bacteroides massiliensis |
| 447 | 87_1513 | 7 | 6 | Firmicutes | Clostridia | Clostridiales | Lachnospiraceae | Blautia | Blautia |
| 474 | 32_934 | 9 | 5 | Bacteroidetes | Bacteroidia | Bacteroidales | Bacteroidaceae | Bacteroides | Bacteroides dorei |
| 479 | 106_66 | 11 | 5 | Bacteroidetes | Bacteroidia | Bacteroidales | Prevotellaceae | Prevotella | Prevotella stercorea |
| 480 | 129_4171 | 6 | 5 | Bacteroidetes | Bacteroidia | Bacteroidales | Prevotellaceae | Prevotella | Prevotella copri |
| 496 | 63_3345 | 6 | 5 | Bacteroidetes | Bacteroidia | Bacteroidales | Bacteroidaceae | Bacteroides | Bacteroides vulgatus |
| 514 | 100_1414 | 8 | 6 | Bacteroidetes | Bacteroidia | Bacteroidales | Porphyromonadaceae | Parabacteroides | Parabacteroides distasonis |
| 540 | 12_3745 | 57 | 14 | Bacteroidetes | Bacteroidia | Bacteroidales | Bacteroidaceae | Bacteroides | Bacteroides vulgatus |
| 569 | 122_660 | 7 | 5 | Bacteroidetes | Bacteroidia | Bacteroidales | Bacteroidaceae | Bacteroides | Bacteroides dorei |
| 596 | 42_5177 | 19 | 11 | Bacteroidetes | Bacteroidia | Bacteroidales | Bacteroidaceae | Bacteroides | Bacteroides vulgatus |
| 628 | 121_3653 | 9 | 5 | Firmicutes | Negativicutes | Selenomonadales | Veillonellaceae | Megasphaera | Megasphaera elsdenii |
| 647 | 122_970 | 8 | 5 | Bacteroidetes | Bacteroidia | Bacteroidales | Bacteroidaceae | Bacteroides | Bacteroides dorei |
| 661 | 127_3727 | 8 | 6 | Firmicutes | Clostridia | Clostridiales | Lachnospiraceae | Roseburia | Eubacterium rectale |
| 665 | 106_2192 | 36 | 1 | Firmicutes | Clostridia | Clostridiales | Lactobacillaceae | Lactobacillus | Lactobacillus gasseri |
| 666 | 43_2962 | 13 | 6 | Firmicutes | Clostridia | Clostridiales | Ruminococcaceae | Faecalibacterium | Faecalibacterium prausnitzii |
| 838 | 47_982 | 7 | 6 | Firmicutes | Clostridia | Clostridiales | Ruminococcaceae | Faecalibacterium | Faecalibacterium prausnitzii |
| 886 | 64_9831 | 135 | 4 | Firmicutes | Bacilli | Lactobacillales | Streptococcaceae | Streptococcus | Streptococcus sp. |
| 1323 | 63_2344 | 8 | 6 | Firmicutes | Clostridia | Clostridiales | Ruminococcaceae | Subdoligranulum | Subdoligranulum |
| 1400 | 9_6578 | 10 | 5 | Bacteroidetes | Bacteroidia | Bacteroidales | Porphyromonadaceae | Butyricimonas | Butyricimonas virosa |
| 1458 | 106_2581 | 9 | 5 | Firmicutes | Clostridia | Clostridiales | Ruminococcaceae | Subdoligranulum | Subdoligranulum |
| 1459 | 117_2356 | 7 | 5 | Firmicutes | Clostridia | Clostridiales | Ruminococcaceae | Subdoligranulum | Subdoligranulum |
| 1529 | 117_2276 | 8 | 5 | Firmicutes | Clostridia | Clostridiales | Ruminococcaceae | Subdoligranulum | Subdoligranulum |
| 1667 | 130_2192 | 12 | 5 | Firmicutes | Clostridia | Clostridiales | Ruminococcaceae | Subdoligranulum | Subdoligranulum |
| 1669 | 130_1260 | 10 | 6 | Firmicutes | Clostridia | Clostridiales | Ruminococcaceae | Subdoligranulum | Subdoligranulum |
| 1859 | 1_1432 | 5 | 5 | Firmicutes | Erysipelotrichia | Erysipelotrichales | Erysipelotrichaceae | Catenibacterium | Catenibacterium mitsuokai |
| 2138 | 100_10004 | 61 | 3 | Bacteroidetes | Bacteroidia | Bacteroidales | Bacteroidaceae | Bacteroides | Bacteroides xylanisolvens |
| 2564 | 37_3056 | 6 | 6 | Firmicutes | Clostridia | Clostridiales | Ruminococcaceae | Faecalibacterium | Faecalibacterium prausnitzii |
| 2575 | 89_6728 | 56 | 3 | Bacteroidetes | Bacteroidia | Bacteroidales | Bacteroidaceae | Bacteroides | Bacteroides xylanisolvens |
| 2612 | 128_1408 | 39 | 4 | Firmicutes | Negativicutes | Selenomonadales | Veillonellaceae | Dialister | Dialister invisus |
| 2616 | 121_5961 | 23 | 3 | Firmicutes | Negativicutes | Selenomonadales | Veillonellaceae | Dialister | Dialister invisus |
| 2725 | 95_1837 | 6 | 5 | Firmicutes | Negativicutes | Selenomonadales | Veillonellaceae | Dialister | Dialister invisus |
| 2745 | 33_5408 | 8 | 5 | Firmicutes | Clostridia | Clostridiales | Ruminococcaceae | Faecalibacterium | Faecalibacterium prausnitzii |
| 2768 | 89_7389 | 1114 | 22 | Bacteroidetes | Bacteroidia | Bacteroidales | Bacteroidaceae | Bacteroides | Bacteroides xylanisolvens |
| 2794 | 114_7557 | 742 | 30 | Bacteroidetes | Bacteroidia | Bacteroidales | Prevotellaceae | Paraprevotella | Paraprevotella clara |
| 2831 | 130_3840 | 114 | 21 | Firmicutes | Negativicutes | Selenomonadales | Veillonellaceae | Dialister | Dialister invisus |
| 2843 | 49_4623 | 20 | 6 | Firmicutes | Negativicutes | Selenomonadales | Veillonellaceae | Dialister | Dialister invisus |
| 2854 | 76_2588 | 9 | 5 | Firmicutes | Clostridia | Clostridiales | Ruminococcaceae | Ruminiclostridium | Clostridium leptum |
| 3084 | 49_1996 | 4185 | 45 | Firmicutes | Negativicutes | Selenomonadales | Veillonellaceae | Dialister | Dialister invisus |
| 3092 | 128_1293 | 13 | 6 | Firmicutes | Negativicutes | Selenomonadales | Veillonellaceae | Dialister | Dialister invisus |
| 3102 | 76_5308 | 50 | 7 | Firmicutes | Clostridia | Clostridiales | Ruminococcaceae | Faecalibacterium | Faecalibacterium prausnitzii |
| 3115 | 107_210 | 440 | 41 | Firmicutes | Clostridia | Clostridiales | Ruminococcaceae | Ruminiclostridium | Clostridium leptum |
| 3137 | 49_3163 | 6 | 5 | Firmicutes | Negativicutes | Selenomonadales | Veillonellaceae | Dialister | Dialister invisus |
| 3194 | 49_1670 | 192 | 23 | Firmicutes | Negativicutes | Selenomonadales | Veillonellaceae | Dialister | Dialister invisus |
| 3200 | 49_2211 | 4108 | 43 | Firmicutes | Negativicutes | Selenomonadales | Veillonellaceae | Dialister | Dialister invisus |
| 3209 | 49_2293 | 119 | 20 | Firmicutes | Negativicutes | Selenomonadales | Veillonellaceae | Dialister | Dialister invisus |
| 3214 | 48_3233 | 11 | 5 | Firmicutes | Negativicutes | Selenomonadales | Veillonellaceae | Dialister | Dialister invisus |
| 3218 | 71_3542 | 63 | 7 | Firmicutes | Negativicutes | Selenomonadales | Veillonellaceae | Allisonella | Allisonella histaminiformans |
| 3239 | 53_5179 | 81 | 14 | Firmicutes | Clostridia | Clostridiales | Ruminococcaceae | Faecalibacterium | Faecalibacterium prausnitzii |
| 3247 | 87_577 | 54 | 5 | Firmicutes | Clostridia | Clostridiales | Lachnospiraceae | Blautia | Blautia |
| 3271 | 100_4260 | 943 | 8 | Bacteroidetes | Bacteroidia | Bacteroidales | Bacteroidaceae | Bacteroides | Bacteroides xylanisolvens |
| 3296 | 100_9612 | 82 | 19 | Bacteroidetes | Bacteroidia | Bacteroidales | Bacteroidaceae | Bacteroides | Bacteroides xylanisolvens |
| 3321 | 58_2493 | 553 | 30 | Bacteroidetes | Bacteroidia | Bacteroidales | Prevotellaceae | Paraprevotella | Paraprevotella clara |
| 3349 | 65_7250 | 11 | 7 | Firmicutes | Negativicutes | Selenomonadales | Veillonellaceae | Megasphaera | Megasphaera elsdenii |
| 3376 | 128_3933 | 764 | 16 | Firmicutes | Negativicutes | Selenomonadales | Veillonellaceae | Dialister | Dialister invisus |
| 3380 | 49_1476 | 4621 | 38 | Firmicutes | Negativicutes | Selenomonadales | Veillonellaceae | Dialister | Dialister invisus |
| 3381 | 49_4310 | 227 | 30 | Firmicutes | Negativicutes | Selenomonadales | Veillonellaceae | Dialister | Dialister invisus |
| 3399 | 128_4889 | 677 | 34 | Firmicutes | Negativicutes | Selenomonadales | Veillonellaceae | Dialister | Dialister invisus |
| 3403 | 130_3227 | 109 | 15 | Firmicutes | Negativicutes | Selenomonadales | Veillonellaceae | Dialister | Dialister invisus |
| 3408 | 49_4656 | 138 | 10 | Firmicutes | Negativicutes | Selenomonadales | Veillonellaceae | Dialister | Dialister invisus |
| 3409 | 49_2316 | 21 | 10 | Firmicutes | Negativicutes | Selenomonadales | Veillonellaceae | Dialister | Dialister invisus |
| 3415 | 49_2980 | 62 | 12 | Firmicutes | Negativicutes | Selenomonadales | Veillonellaceae | Dialister | Dialister invisus |
| 3420 | 71_3486 | 450 | 10 | Firmicutes | Negativicutes | Selenomonadales | Acidaminococcaceae | Succiniclasticum | Succiniclasticum ruminis |
| 3421 | 49_291 | 105 | 10 | Firmicutes | Negativicutes | Selenomonadales | Veillonellaceae | Dialister | Dialister invisus |
| 3442 | 106_1867 | 1681 | 40 | Firmicutes | Clostridia | Clostridiales | Lachnospiraceae | Blautia | Blautia |
| 3467 | 89_1299 | 1806 | 49 | Bacteroidetes | Bacteroidia | Bacteroidales | Bacteroidaceae | Bacteroides | Bacteroides xylanisolvens |
| 3482 | 111_4700 | 125 | 21 | Bacteroidetes | Bacteroidia | Bacteroidales | Bacteroidaceae | Bacteroides | Bacteroides uniformis |
| 3531 | 93_4140 | 29 | 5 | Bacteroidetes | Bacteroidia | Bacteroidales | Prevotellaceae | Paraprevotella | Paraprevotella clara |
| 3544 | 128_2595 | 59 | 8 | Firmicutes | Negativicutes | Selenomonadales | Veillonellaceae | Dialister | Dialister invisus |
| 3546 | 49_4508 | 2316 | 44 | Firmicutes | Negativicutes | Selenomonadales | Veillonellaceae | Dialister | Dialister invisus |
| 3579 | 130_1418 | 6 | 6 | Firmicutes | Negativicutes | Selenomonadales | Veillonellaceae | Dialister | Dialister invisus |
| 3589 | 49_4306 | 22 | 10 | Firmicutes | Negativicutes | Selenomonadales | Veillonellaceae | Dialister | Dialister invisus |
| 3591 | 60_2497 | 9 | 7 | Firmicutes | Clostridia | Clostridiales | Ruminococcaceae | Ruminiclostridium | Clostridium leptum |
| 3597 | 49_1450 | 19 | 10 | Firmicutes | Negativicutes | Selenomonadales | Veillonellaceae | Dialister | Dialister invisus |
| 3598 | 49_4453 | 8 | 5 | Firmicutes | Negativicutes | Selenomonadales | Veillonellaceae | Dialister | Dialister invisus |
| 3599 | 49_4032 | 106 | 19 | Firmicutes | Negativicutes | Selenomonadales | Veillonellaceae | Dialister | Dialister invisus |
| 3600 | 49_4547 | 10 | 6 | Firmicutes | Negativicutes | Selenomonadales | Veillonellaceae | Dialister | Dialister invisus |
| 3609 | 49_1043 | 24 | 8 | Firmicutes | Negativicutes | Selenomonadales | Veillonellaceae | Dialister | Dialister invisus |
| 3617 | 49_4612 | 53 | 10 | Firmicutes | Negativicutes | Selenomonadales | Veillonellaceae | Dialister | Dialister invisus |
| 3619 | 49_4405 | 7 | 5 | Firmicutes | Negativicutes | Selenomonadales | Veillonellaceae | Dialister | Dialister invisus |
| 3623 | 60_1347 | 13 | 6 | Firmicutes | Negativicutes | Selenomonadales | Veillonellaceae | Dialister | Dialister invisus |
| 3630 | 60_1777 | 41 | 8 | Firmicutes | Negativicutes | Selenomonadales | Veillonellaceae | Dialister | Dialister invisus |
| 3631 | 49_1446 | 19 | 7 | Firmicutes | Negativicutes | Selenomonadales | Veillonellaceae | Dialister | Dialister invisus |
| 3635 | 49_1643 | 54 | 9 | Firmicutes | Negativicutes | Selenomonadales | Veillonellaceae | Dialister | Dialister invisus |
| 3636 | 60_6870 | 22 | 9 | Firmicutes | Negativicutes | Selenomonadales | Veillonellaceae | Dialister | Dialister invisus |
| 3658 | 18_6715 | 29 | 3 | Actinobacteria | Coriobacteriia | Coriobacteriales | Coriobacteriaceae | Collinsella | Collinsella aerofaciens |
| 3678 | 2_1537 | 20 | 4 | Firmicutes | Clostridia | Clostridiales | Lachnospiraceae | Blautia | Blautia |
| 3708 | 89_1338 | 75 | 6 | Bacteroidetes | Bacteroidia | Bacteroidales | Bacteroidaceae | Bacteroides | Bacteroides xylanisolvens |
| 3713 | 89_5429 | 62 | 3 | Bacteroidetes | Bacteroidia | Bacteroidales | Bacteroidaceae | Bacteroides | Bacteroides xylanisolvens |
| 3719 | 89_9667 | 214 | 9 | Bacteroidetes | Bacteroidia | Bacteroidales | Bacteroidaceae | Bacteroides | Bacteroides xylanisolvens |
| 3759 | 128_5932 | 17 | 7 | Firmicutes | Negativicutes | Selenomonadales | Veillonellaceae | Dialister | Dialister invisus |
| 3763 | 104_762 | 13 | 5 | Firmicutes | Negativicutes | Selenomonadales | Veillonellaceae | Dialister | Dialister invisus |
| 3768 | 56_4232 | 46 | 6 | Bacteroidetes | Bacteroidia | Bacteroidales | Prevotellaceae | Paraprevotella | Paraprevotella clara |
| 3769 | 58_7690 | 237 | 13 | Bacteroidetes | Bacteroidia | Bacteroidales | Bacteroidaceae | Bacteroides | Bacteroides ovatus |
| 3784 | 63_4696 | 97 | 13 | Firmicutes | Negativicutes | Selenomonadales | Veillonellaceae | Dialister | Dialister invisus |
| 3785 | 71_8677 | 193 | 35 | Firmicutes | Negativicutes | Selenomonadales | Veillonellaceae | Allisonella | Allisonella histaminiformans |
| 3786 | 102_4837 | 193 | 19 | Firmicutes | Negativicutes | Selenomonadales | Veillonellaceae | Megasphaera | Megasphaera elsdenii |
| 3831 | 49_1808 | 13 | 8 | Firmicutes | Negativicutes | Selenomonadales | Veillonellaceae | Dialister | Dialister invisus |
| 3836 | 128_2787 | 22 | 4 | Firmicutes | Negativicutes | Selenomonadales | Veillonellaceae | Dialister | Dialister invisus |
| 3837 | 128_986 | 10 | 7 | Firmicutes | Negativicutes | Selenomonadales | Veillonellaceae | Dialister | Dialister invisus |
| 3848 | 131_2601 | 5 | 5 | Firmicutes | Negativicutes | Selenomonadales | Veillonellaceae | Dialister | Dialister invisus |
| 3857 | 128_3201 | 13 | 5 | Firmicutes | Negativicutes | Selenomonadales | Veillonellaceae | Dialister | Dialister invisus |
| 3858 | 128_6077 | 935 | 39 | Firmicutes | Negativicutes | Selenomonadales | Veillonellaceae | Dialister | Dialister invisus |
| 3872 | 49_4195 | 9 | 5 | Firmicutes | Negativicutes | Selenomonadales | Veillonellaceae | Dialister | Dialister invisus |
| 3873 | 49_174 | 17 | 9 | Firmicutes | Negativicutes | Selenomonadales | Veillonellaceae | Dialister | Dialister invisus |
| 3876 | 71_2525 | 34 | 17 | Firmicutes | Negativicutes | Selenomonadales | Veillonellaceae | Allisonella | Allisonella histaminiformans |
| 3877 | 50_1231 | 66 | 6 | Firmicutes | Negativicutes | Selenomonadales | Acidaminococcaceae | Succiniclasticum | Succiniclasticum ruminis |
| 3902 | 32_6799 | 9 | 8 | Firmicutes | Clostridia | Clostridiales | Ruminococcaceae | Faecalibacterium | Faecalibacterium prausnitzii |
| 3931 | 95_5699 | 26 | 5 | Firmicutes | Clostridia | Clostridiales | Lachnospiraceae | Blautia | Blautia |
| 3932 | 72_634 | 8 | 6 | Firmicutes | Clostridia | Clostridiales | Lachnospiraceae | Blautia | Blautia |
| 3947 | 49_3306 | 7 | 5 | Firmicutes | Negativicutes | Selenomonadales | Veillonellaceae | Dialister | Dialister invisus |
| 4060 | 63_4943 | 306 | 20 | Firmicutes | Negativicutes | Selenomonadales | Veillonellaceae | Megasphaera | Megasphaera elsdenii |
| 4088 | 128_2240 | 70 | 3 | Firmicutes | Negativicutes | Selenomonadales | Veillonellaceae | Dialister | Dialister invisus |
| 4095 | 63_4799 | 5 | 5 | Firmicutes | Negativicutes | Selenomonadales | Veillonellaceae | Dialister | Dialister invisus |
| 4096 | 49_1974 | 7 | 5 | Firmicutes | Negativicutes | Selenomonadales | Veillonellaceae | Dialister | Dialister invisus |
| 4097 | 130_2053 | 12 | 9 | Firmicutes | Negativicutes | Selenomonadales | Veillonellaceae | Dialister | Dialister invisus |
| 4104 | 128_5666 | 18 | 10 | Firmicutes | Negativicutes | Selenomonadales | Veillonellaceae | Dialister | Dialister invisus |
| 4117 | 49_1896 | 11 | 5 | Firmicutes | Negativicutes | Selenomonadales | Veillonellaceae | Dialister | Dialister invisus |
| 4118 | 128_6269 | 35 | 10 | Firmicutes | Negativicutes | Selenomonadales | Veillonellaceae | Dialister | Dialister invisus |
| 4119 | 42_2538 | 15 | 10 | Firmicutes | Negativicutes | Selenomonadales | Veillonellaceae | Dialister | Dialister invisus |
| 4129 | 71_6349 | 5 | 5 | Firmicutes | Negativicutes | Selenomonadales | Veillonellaceae | Dialister | Dialister invisus |
| 4134 | 49_2745 | 7 | 5 | Firmicutes | Negativicutes | Selenomonadales | Veillonellaceae | Dialister | Dialister invisus |
| 4139 | 49_4259 | 15 | 9 | Firmicutes | Negativicutes | Selenomonadales | Veillonellaceae | Dialister | Dialister invisus |
| 4171 | 106_651 | 20 | 1 | Firmicutes | Negativicutes | Selenomonadales | Veillonellaceae | Dialister | Dialister invisus |
| 4191 | 132_1599 | 238 | 34 | Firmicutes | Clostridia | Clostridiales | Ruminococcaceae | Faecalibacterium | Faecalibacterium prausnitzii |
| 4192 | 16_5874 | 560 | 53 | Firmicutes | Clostridia | Clostridiales | Ruminococcaceae | Faecalibacterium | Faecalibacterium prausnitzii |
| 4196 | 67_8959 | 28 | 4 | Proteobacteria | Betaproteobacteria | Burkholderiales | Sutterellaceae | Parasutterella | Parasutterella excrementihominis |
| 4203 | 68_3619 | 121 | 21 | Firmicutes | Clostridia | Clostridiales | Lachnospiraceae | Lachnospiraceae incertae sedis | Lachnospiraceae incertae sedis |
| 4235 | 102_659 | 66 | 11 | Bacteroidetes | Bacteroidia | Bacteroidales | Bacteroidaceae | Bacteroides | Bacteroides ovatus |
| 4257 | 89_9712 | 105 | 24 | Bacteroidetes | Bacteroidia | Bacteroidales | Bacteroidaceae | Bacteroides | Bacteroides xylanisolvens |
| 4308 | 63_744 | 24 | 13 | Firmicutes | Negativicutes | Selenomonadales | Veillonellaceae | Dialister | Dialister invisus |
| 4319 | 49_220 | 27 | 6 | Firmicutes | Negativicutes | Selenomonadales | Veillonellaceae | Dialister | Dialister invisus |
| 4341 | 96_7590 | 99 | 6 | Bacteroidetes | Bacteroidia | Bacteroidales | Prevotellaceae | Paraprevotella | Paraprevotella clara |
| 4343 | 108_1178 | 370 | 26 | Bacteroidetes | Bacteroidia | Bacteroidales | Prevotellaceae | Paraprevotella | Paraprevotella clara |
| 4344 | 56_4297 | 338 | 33 | Bacteroidetes | Bacteroidia | Bacteroidales | Prevotellaceae | Paraprevotella | Paraprevotella clara |
| 4366 | 60_6823 | 649 | 25 | Firmicutes | Negativicutes | Selenomonadales | Acidaminococcaceae | Acidaminococcus | Acidaminococcus intestini |
| 4451 | 49_292 | 16 | 8 | Firmicutes | Negativicutes | Selenomonadales | Veillonellaceae | Dialister | Dialister invisus |
| 4487 | 49_3417 | 76 | 11 | Firmicutes | Negativicutes | Selenomonadales | Veillonellaceae | Dialister | Dialister invisus |
| 4491 | 128_1751 | 7 | 6 | Firmicutes | Negativicutes | Selenomonadales | Veillonellaceae | Dialister | Dialister invisus |
| 4493 | 63_2943 | 82 | 22 | Firmicutes | Negativicutes | Selenomonadales | Veillonellaceae | Dialister | Dialister invisus |
| 4495 | 106_3695 | 12 | 8 | Firmicutes | Negativicutes | Selenomonadales | Veillonellaceae | Dialister | Dialister invisus |
| 4516 | 71_2386 | 30 | 18 | Firmicutes | Negativicutes | Selenomonadales | Veillonellaceae | Allisonella | Allisonella histaminiformans |
| 4524 | 50_1143 | 75 | 7 | NULL | NULL | NULL | NULL | NULL | NULL |
| 4525 | 50_3051 | 106 | 8 | NULL | NULL | NULL | NULL | NULL | NULL |
| 4539 | 125_2447 | 23 | 4 | Firmicutes | Negativicutes | Selenomonadales | Acidaminococcaceae | Acidaminococcus | Acidaminococcus fermentans |
| 4577 | 71_2861 | 226 | 44 | Actinobacteria | Coriobacteriia | Coriobacteriales | Coriobacteriaceae | Collinsella | Collinsella aerofaciens |
| 4591 | 2_4770 | 335 | 7 | Firmicutes | Clostridia | Clostridiales | Lachnospiraceae | Blautia | Blautia |
| 4638 | 15_4919 | 23 | 8 | Bacteroidetes | Bacteroidia | Bacteroidales | Prevotellaceae | Paraprevotella | Paraprevotella clara |
| 4675 | 79_7280 | 534 | 17 | Bacteroidetes | Bacteroidia | Bacteroidales | Bacteroidaceae | Bacteroides | Bacteroides ovatus |
| 4678 | 68_1255 | 447 | 28 | Bacteroidetes | Bacteroidia | Bacteroidales | Bacteroidaceae | Bacteroides | Bacteroides ovatus |
| 4704 | 49_368 | 13 | 6 | Firmicutes | Negativicutes | Selenomonadales | Veillonellaceae | Dialister | Dialister invisus |
| 4739 | 79_2148 | 72 | 7 | Bacteroidetes | Bacteroidia | Bacteroidales | Bacteroidaceae | Bacteroides | Bacteroides ovatus |
| 4746 | 25_2217 | 21 | 4 | Firmicutes | Negativicutes | Selenomonadales | Veillonellaceae | Allisonella | Allisonella histaminiformans |
| 4778 | 102_3962 | 46 | 7 | Firmicutes | Negativicutes | Selenomonadales | Veillonellaceae | Megasphaera | Megasphaera elsdenii |
| 4792 | 128_4584 | 1183 | 23 | Firmicutes | Negativicutes | Selenomonadales | Veillonellaceae | Megasphaera | Megasphaera elsdenii |
| 4821 | 17_1822 | 6 | 5 | Firmicutes | Clostridia | Clostridiales | Lachnospiraceae | Blautia | Blautia |
| 4833 | 128_6274 | 35 | 14 | Firmicutes | Negativicutes | Selenomonadales | Veillonellaceae | Dialister | Dialister invisus |
| 4840 | 106_7005 | 17 | 10 | Firmicutes | Negativicutes | Selenomonadales | Veillonellaceae | Dialister | Dialister invisus |
| 4854 | 106_6715 | 29 | 7 | Firmicutes | Negativicutes | Selenomonadales | Veillonellaceae | Dialister | Dialister invisus |
| 4877 | 55_3537 | 19 | 7 | Firmicutes | Negativicutes | Selenomonadales | Veillonellaceae | Dialister | Dialister invisus |
| 4879 | 50_1193 | 81 | 7 | Firmicutes | Negativicutes | Selenomonadales | Acidaminococcaceae | Succiniclasticum | Succiniclasticum ruminis |
| 4885 | 117_4946 | 32 | 11 | Firmicutes | Negativicutes | Selenomonadales | Acidaminococcaceae | Acidaminococcus | Acidaminococcus intestini |
| 4926 | 129_3854 | 8 | 6 | Firmicutes | Clostridia | Clostridiales | Ruminococcaceae | Faecalibacterium | Faecalibacterium prausnitzii |
| 4927 | 57_482 | 215 | 33 | Firmicutes | Clostridia | Clostridiales | Ruminococcaceae | Faecalibacterium | Faecalibacterium prausnitzii |
| 4937 | 25_814 | 5 | 5 | Firmicutes | Clostridia | Clostridiales | Clostridiales Family XIII | Clostridiales Family XIII incertae sedis | Clostridiales Family XIII incertae sedis |
| 4942 | 67_5773 | 15 | 11 | Firmicutes | Clostridia | Clostridiales | Lachnospiraceae | Lachnospiraceae incertae sedis | Lachnospiraceae incertae sedis |
| 4952 | 131_4764 | 8 | 5 | Firmicutes | Clostridia | Clostridiales | Ruminococcaceae | Ruminiclostridium | Clostridium leptum |
| 4955 | 61_4771 | 18 | 9 | Firmicutes | Clostridia | Clostridiales | Ruminococcaceae | Anaerotruncus | Anaerotruncus sp. |
| 4958 | 95_5927 | 52 | 12 | Firmicutes | Clostridia | Clostridiales | Lachnospiraceae | Blautia | Blautia |
| 4967 | 45_6926 | 29 | 12 | Proteobacteria | Deltaproteobacteria | Desulfovibrionales | Desulfovibrionaceae | Bilophila | Bilophila wadsworthia |
| 4971 | 108_3494 | 181 | 8 | Bacteroidetes | Bacteroidia | Bacteroidales | Porphyromonadaceae | Barnesiella | Barnesiella |
| 5001 | 91_5657 | 29 | 8 | Bacteroidetes | Bacteroidia | Bacteroidales | Bacteroidaceae | Bacteroides | Bacteroides ovatus |
| 5048 | 102_991 | 17 | 6 | Bacteroidetes | Bacteroidia | Bacteroidales | Bacteroidaceae | Bacteroides | Bacteroides vulgatus |
| 5059 | 63_1105 | 12 | 6 | Firmicutes | Negativicutes | Selenomonadales | Veillonellaceae | Dialister | Dialister invisus |
| 5109 | 125_3543 | 1825 | 12 | Firmicutes | Negativicutes | Selenomonadales | Veillonellaceae | Dialister | Dialister invisus |
| 5128 | 128_5052 | 8 | 6 | Firmicutes | Negativicutes | Selenomonadales | Veillonellaceae | Dialister | Dialister invisus |
| 5129 | 38_6018 | 24 | 6 | Firmicutes | Negativicutes | Selenomonadales | Acidaminococcaceae | Succiniclasticum | Succiniclasticum ruminis |
| 5153 | 102_5708 | 27 | 9 | Firmicutes | Negativicutes | Selenomonadales | Veillonellaceae | Megasphaera | Megasphaera elsdenii |
| 5167 | 125_1316 | 77 | 11 | Firmicutes | Negativicutes | Selenomonadales | Veillonellaceae | Megasphaera | Megasphaera elsdenii |
| 5170 | 1_1001 | 650 | 26 | Firmicutes | Negativicutes | Selenomonadales | Veillonellaceae | Megasphaera | Megasphaera elsdenii |
| 5187 | 106_1185 | 10 | 5 | Bacteroidetes | Bacteroidia | Bacteroidales | Bacteroidaceae | Bacteroides | Bacteroides caccae |
| 5197 | 49_1782 | 13 | 9 | Firmicutes | Clostridia | Clostridiales | Lachnospiraceae | Blautia | Blautia |
| 5209 | 128_1365 | 15 | 7 | Firmicutes | Negativicutes | Selenomonadales | Veillonellaceae | Dialister | Dialister invisus |
| 5216 | 49_3404 | 7 | 5 | Firmicutes | Negativicutes | Selenomonadales | Veillonellaceae | Dialister | Dialister invisus |
| 5219 | 49_3984 | 16 | 9 | Firmicutes | Negativicutes | Selenomonadales | Veillonellaceae | Dialister | Dialister invisus |
| 5236 | 50_913 | 38 | 5 | Firmicutes | Negativicutes | Selenomonadales | Acidaminococcaceae | Succiniclasticum | Succiniclasticum ruminis |
| 5238 | 50_3151 | 15 | 5 | Firmicutes | Negativicutes | Selenomonadales | Acidaminococcaceae | Succiniclasticum | Succiniclasticum ruminis |
| 5243 | 49_3217 | 25 | 7 | Firmicutes | Negativicutes | Selenomonadales | Veillonellaceae | Dialister | Dialister invisus |
| 5273 | 33_1780 | 72 | 22 | Firmicutes | Clostridia | Clostridiales | Ruminococcaceae | Faecalibacterium | Faecalibacterium prausnitzii |
| 5281 | 20_822 | 9 | 7 | Firmicutes | Clostridia | Clostridiales | Ruminococcaceae | Faecalibacterium | Faecalibacterium prausnitzii |
| 5284 | 76_2672 | 354 | 30 | Firmicutes | Clostridia | Clostridiales | Ruminococcaceae | Faecalibacterium | Faecalibacterium prausnitzii |
| 5286 | 97_4049 | 31 | 18 | Firmicutes | Clostridia | Clostridiales | Ruminococcaceae | Faecalibacterium | Faecalibacterium prausnitzii |
| 5299 | 63_6209 | 156 | 19 | Proteobacteria | Betaproteobacteria | Burkholderiales | Sutterellaceae | Parasutterella | Parasutterella excrementihominis |
| 5340 | 6_439 | 12 | 7 | Proteobacteria | Deltaproteobacteria | Desulfovibrionales | Desulfovibrionaceae | Bilophila | Bilophila wadsworthia |
| 5341 | 123_6924 | 40 | 17 | Proteobacteria | Deltaproteobacteria | Desulfovibrionales | Desulfovibrionaceae | Bilophila | Bilophila wadsworthia |
| 5342 | 50_1433 | 33 | 16 | Proteobacteria | Deltaproteobacteria | Desulfovibrionales | Desulfovibrionaceae | Bilophila | Bilophila wadsworthia |
| 5351 | 60_4999 | 6 | 5 | Bacteroidetes | Bacteroidia | Bacteroidales | Porphyromonadaceae | Parabacteroides | Parabacteroides merdae |
| 5365 | 89_7653 | 15 | 5 | Bacteroidetes | Bacteroidia | Bacteroidales | Bacteroidaceae | Bacteroides | Bacteroides ovatus |
| 5372 | 79_8329 | 35 | 6 | Bacteroidetes | Bacteroidia | Bacteroidales | Bacteroidaceae | Bacteroides | Bacteroides ovatus |
| 5409 | 49_1824 | 43 | 17 | Firmicutes | Negativicutes | Selenomonadales | Veillonellaceae | Dialister | Dialister invisus |
| 5415 | 99_4208 | 8 | 7 | Bacteroidetes | Bacteroidia | Bacteroidales | Bacteroidaceae | Bacteroides | Bacteroides uniformis |
| 5471 | 39_5393 | 7 | 5 | Bacteroidetes | Bacteroidia | Bacteroidales | Prevotellaceae | Prevotella | Prevotella sp. |
| 5506 | 17_659 | 157 | 5 | Bacteroidetes | Bacteroidia | Bacteroidales | Prevotellaceae | Prevotella | Prevotella copri |
| 5541 | 60_3399 | 289 | 10 | Firmicutes | Negativicutes | Selenomonadales | Acidaminococcaceae | Acidaminococcus | Acidaminococcus intestini |
| 5545 | 49_3040 | 31 | 13 | Firmicutes | Negativicutes | Selenomonadales | Veillonellaceae | Dialister | Dialister invisus |
| 5575 | 102_1189 | 40 | 6 | Firmicutes | Negativicutes | Selenomonadales | Veillonellaceae | Megasphaera | Megasphaera elsdenii |
| 5579 | 130_3160 | 10 | 5 | Firmicutes | Negativicutes | Selenomonadales | Veillonellaceae | Megasphaera | Megasphaera elsdenii |
| 5581 | 102_6220 | 21 | 5 | Firmicutes | Negativicutes | Selenomonadales | Veillonellaceae | Megasphaera | Megasphaera elsdenii |
| 5586 | 63_5836 | 22 | 5 | Firmicutes | Negativicutes | Selenomonadales | Veillonellaceae | Megasphaera | Megasphaera elsdenii |
| 5600 | 107_4461 | 62 | 7 | Firmicutes | Negativicutes | Selenomonadales | Veillonellaceae | Megasphaera | Megasphaera elsdenii |
| 5622 | 125_3334 | 62 | 6 | Firmicutes | Negativicutes | Selenomonadales | Veillonellaceae | Megasphaera | Megasphaera elsdenii |
| 5703 | 50_2224 | 40 | 6 | NULL | NULL | NULL | NULL | NULL | NULL |
| 5715 | 60_3157 | 152 | 17 | Firmicutes | Negativicutes | Selenomonadales | Acidaminococcaceae | Acidaminococcus | Acidaminococcus intestini |
| 5717 | 49_4693 | 88 | 8 | Firmicutes | Negativicutes | Selenomonadales | Acidaminococcaceae | Acidaminococcus | Acidaminococcus intestini |
| 5747 | 5_673 | 878 | 64 | Firmicutes | Clostridia | Clostridiales | Ruminococcaceae | Faecalibacterium | Faecalibacterium prausnitzii |
| 5754 | 16_2906 | 73 | 23 | Firmicutes | Clostridia | Clostridiales | Ruminococcaceae | Faecalibacterium | Faecalibacterium prausnitzii |
| 5755 | 129_3044 | 115 | 15 | Firmicutes | Clostridia | Clostridiales | Ruminococcaceae | Faecalibacterium | Faecalibacterium prausnitzii |
| 5756 | 76_4080 | 6 | 5 | Firmicutes | Clostridia | Clostridiales | Ruminococcaceae | Faecalibacterium | Faecalibacterium prausnitzii |
| 5758 | 75_1167 | 63 | 17 | Proteobacteria | Betaproteobacteria | Burkholderiales | Sutterellaceae | Parasutterella | Parasutterella excrementihominis |
| 5760 | 2_2880 | 101 | 6 | Proteobacteria | Betaproteobacteria | Burkholderiales | Sutterellaceae | Parasutterella | Parasutterella excrementihominis |
| 5762 | 55_5599 | 549 | 35 | Proteobacteria | Betaproteobacteria | Burkholderiales | Sutterellaceae | Parasutterella | Parasutterella excrementihominis |
| 5763 | 55_6521 | 6 | 5 | Proteobacteria | Betaproteobacteria | Burkholderiales | Sutterellaceae | Parasutterella | Parasutterella excrementihominis |
| 5816 | 61_3470 | 17 | 11 | Proteobacteria | Deltaproteobacteria | Desulfovibrionales | Desulfovibrionaceae | Bilophila | Bilophila wadsworthia |
| 5818 | 23_5356 | 7 | 7 | Proteobacteria | Deltaproteobacteria | Desulfovibrionales | Desulfovibrionaceae | Bilophila | Bilophila wadsworthia |
| 5822 | 55_5313 | 30 | 8 | Proteobacteria | Deltaproteobacteria | Desulfovibrionales | Desulfovibrionaceae | Bilophila | Bilophila wadsworthia |
| 5828 | 91_5546 | 619 | 33 | Bacteroidetes | Bacteroidia | Bacteroidales | Porphyromonadaceae | Barnesiella | Barnsiella intestinihominis |
| 5844 | 58_7889 | 20 | 2 | Bacteroidetes | Bacteroidia | Bacteroidales | Bacteroidaceae | Bacteroides | Bacteroides ovatus |
| 5855 | 89_5272 | 1830 | 18 | Bacteroidetes | Bacteroidia | Bacteroidales | Bacteroidaceae | Bacteroides | Bacteroides ovatus |
| 5865 | 91_2679 | 15 | 5 | Bacteroidetes | Bacteroidia | Bacteroidales | Bacteroidaceae | Bacteroides | Bacteroides ovatus |
| 5868 | 79_8406 | 31 | 4 | Bacteroidetes | Bacteroidia | Bacteroidales | Bacteroidaceae | Bacteroides | Bacteroides ovatus |
| 5869 | 79_3650 | 23 | 6 | Bacteroidetes | Bacteroidia | Bacteroidales | Bacteroidaceae | Bacteroides | Bacteroides ovatus |
| 5872 | 58_6244 | 1677 | 65 | Bacteroidetes | Bacteroidia | Bacteroidales | Bacteroidaceae | Bacteroides | Bacteroides ovatus |
| 5897 | 77_184 | 20 | 6 | Bacteroidetes | Bacteroidia | Bacteroidales | Bacteroidaceae | Bacteroides | Bacteroides xylanisolvens |
| 5918 | 117_1859 | 11 | 5 | Firmicutes | Negativicutes | Selenomonadales | Veillonellaceae | Dialister | Dialister invisus |
| 5958 | 99_6686 | 56 | 10 | Bacteroidetes | Bacteroidia | Bacteroidales | Bacteroidaceae | Bacteroides | Bacteroides uniformis |
| 5978 | 12_3524 | 1573 | 48 | Bacteroidetes | Bacteroidia | Bacteroidales | Bacteroidaceae | Bacteroides | Bacteroides vulgatus |
| 6007 | 21_7184 | 1201 | 7 | Bacteroidetes | Bacteroidia | Bacteroidales | Prevotellaceae | Prevotella | Prevotella sp. |
| 6080 | 96_4925 | 550 | 10 | Bacteroidetes | Bacteroidia | Bacteroidales | Prevotellaceae | Paraprevotella | Paraprevotella clara |
| 6085 | 131_167 | 14 | 7 | Firmicutes | Negativicutes | Selenomonadales | Veillonellaceae | Dialister | Dialister invisus |
| 6157 | 1_350 | 16 | 5 | Bacteroidetes | Bacteroidia | Bacteroidales | Prevotellaceae | Prevotella | Prevotella copri |
| 6167 | 112_6048 | 10 | 5 | Firmicutes | Negativicutes | Selenomonadales | Veillonellaceae | Megasphaera | Megasphaera elsdenii |
| 6171 | 128_2990 | 41 | 10 | Firmicutes | Negativicutes | Selenomonadales | Veillonellaceae | Megasphaera | Megasphaera elsdenii |
| 6196 | 48_5447 | 22 | 6 | Bacteroidetes | Bacteroidia | Bacteroidales | Prevotellaceae | Prevotella | Prevotella copri |
| 6203 | 63_2483 | 24 | 8 | Bacteroidetes | Bacteroidia | Bacteroidales | Bacteroidaceae | Bacteroides | Bacteroides uniformis |
| 6204 | 121_3094 | 21 | 6 | Bacteroidetes | Bacteroidia | Bacteroidales | Prevotellaceae | Prevotella | Prevotella copri |
| 6233 | 18_6728 | 7 | 6 | Firmicutes | Negativicutes | Selenomonadales | Veillonellaceae | Dialister | Dialister invisus |
| 6251 | 81_6838 | 1820 | 13 | Firmicutes | Negativicutes | Selenomonadales | Veillonellaceae | Dialister | Dialister invisus |
| 6255 | 55_6891 | 210 | 8 | Firmicutes | Negativicutes | Selenomonadales | Veillonellaceae | Dialister | Dialister invisus |
| 6266 | 50_1318 | 11 | 5 | Firmicutes | Negativicutes | Selenomonadales | Acidaminococcaceae | Succiniclasticum | Succiniclasticum ruminis |
| 6277 | 49_3342 | 157 | 15 | Firmicutes | Negativicutes | Selenomonadales | Acidaminococcaceae | Acidaminococcus | Acidaminococcus intestini |
| 6289 | 6_4318 | 6 | 5 | Bacteroidetes | Bacteroidia | Bacteroidales | Prevotellaceae | Prevotella | Prevotella copri |
| 6306 | 129_2345 | 5 | 5 | Firmicutes | Clostridia | Clostridiales | Ruminococcaceae | Faecalibacterium | Faecalibacterium prausnitzii |
| 6308 | 33_6508 | 8 | 8 | Firmicutes | Clostridia | Clostridiales | Ruminococcaceae | Faecalibacterium | Faecalibacterium prausnitzii |
| 6309 | 86_3415 | 14 | 11 | Firmicutes | Clostridia | Clostridiales | Ruminococcaceae | Faecalibacterium | Faecalibacterium prausnitzii |
| 6329 | 41_6047 | 135 | 31 | Proteobacteria | Betaproteobacteria | Burkholderiales | Sutterellaceae | Parasutterella | Parasutterella excrementihominis |
| 6333 | 55_5468 | 75 | 13 | Proteobacteria | Betaproteobacteria | Burkholderiales | Sutterellaceae | Parasutterella | Parasutterella excrementihominis |
| 6334 | 115_3567 | 11 | 8 | Proteobacteria | Betaproteobacteria | Burkholderiales | Sutterellaceae | Parasutterella | Parasutterella excrementihominis |
| 6341 | 17_395 | 29 | 15 | Firmicutes | Negativicutes | Selenomonadales | Veillonellaceae | Dialister | Dialister invisus |
| 6358 | 11_517 | 8 | 5 | NULL | NULL | NULL | NULL | NULL | NULL |
| 6362 | 95_2099 | 80 | 24 | Firmicutes | Clostridia | Clostridiales | Lachnospiraceae | Blautia | Blautia |
| 6385 | 22_3793 | 8 | 5 | Proteobacteria | Deltaproteobacteria | Desulfovibrionales | Desulfovibrionaceae | Bilophila | Bilophila wadsworthia |
| 6405 | 49_2669 | 1532 | 16 | NULL | NULL | NULL | NULL | NULL | NULL |
| 6421 | 1_3621 | 475 | 31 | Bacteroidetes | Bacteroidia | Bacteroidales | Porphyromonadaceae | Parabacteroides | Parabacteroides merdae |
| 6438 | 20_3412 | 1261 | 24 | Bacteroidetes | Bacteroidia | Bacteroidales | Bacteroidaceae | Bacteroides | Bacteroides intestinalis |
| 6441 | 68_12259 | 80 | 9 | Bacteroidetes | Bacteroidia | Bacteroidales | Bacteroidaceae | Bacteroides | Bacteroides ovatus |
| 6443 | 89_5254 | 61 | 7 | Bacteroidetes | Bacteroidia | Bacteroidales | Bacteroidaceae | Bacteroides | Bacteroides ovatus |
| 6450 | 79_7794 | 63 | 7 | Bacteroidetes | Bacteroidia | Bacteroidales | Bacteroidaceae | Bacteroides | Bacteroides ovatus |
| 6469 | 82_11603 | 31 | 3 | Bacteroidetes | Bacteroidia | Bacteroidales | Bacteroidaceae | Bacteroides | Bacteroides ovatus |
| 6472 | 82_5062 | 19409 | 63 | Bacteroidetes | Bacteroidia | Bacteroidales | Bacteroidaceae | Bacteroides | Bacteroides ovatus |
| 6474 | 82_4198 | 5273 | 45 | Bacteroidetes | Bacteroidia | Bacteroidales | Bacteroidaceae | Bacteroides | Bacteroides ovatus |
| 6500 | 89_10142 | 214 | 5 | Bacteroidetes | Bacteroidia | Bacteroidales | Bacteroidaceae | Bacteroides | Bacteroides ovatus |
| 6532 | 63_2002 | 217 | 17 | Bacteroidetes | Bacteroidia | Bacteroidales | Bacteroidaceae | Bacteroides | Bacteroides eggerthii |
| 6550 | 20_8382 | 110 | 29 | Bacteroidetes | Bacteroidia | Bacteroidales | Bacteroidaceae | Bacteroides | Bacteroides uniformis |
| 6552 | 99_4257 | 6 | 5 | Bacteroidetes | Bacteroidia | Bacteroidales | Bacteroidaceae | Bacteroides | Bacteroides uniformis |
| 6554 | 131_4561 | 54 | 23 | Bacteroidetes | Bacteroidia | Bacteroidales | Bacteroidaceae | Bacteroides | Bacteroides uniformis |
| 6559 | 99_9972 | 12 | 8 | Bacteroidetes | Bacteroidia | Bacteroidales | Bacteroidaceae | Bacteroides | Bacteroides uniformis |
| 6564 | 107_3829 | 80 | 22 | Bacteroidetes | Bacteroidia | Bacteroidales | Bacteroidaceae | Bacteroides | Bacteroides uniformis |
| 6586 | 102_24 | 29 | 12 | Bacteroidetes | Bacteroidia | Bacteroidales | Bacteroidaceae | Bacteroides | Bacteroides uniformis |
| 6588 | 4_1987 | 31 | 15 | Bacteroidetes | Bacteroidia | Bacteroidales | Bacteroidaceae | Bacteroides | Bacteroides caccae |
| 6713 | 121_4671 | 10 | 5 | Firmicutes | Negativicutes | Selenomonadales | Veillonellaceae | Dialister | Dialister invisus |
| 6752 | 82_11304 | 30 | 6 | Bacteroidetes | Bacteroidia | Bacteroidales | Bacteroidaceae | Bacteroides | Bacteroides ovatus |
| 6770 | 60_5764 | 15 | 8 | Firmicutes | Negativicutes | Selenomonadales | Acidaminococcaceae | Acidaminococcus | Acidaminococcus intestini |
| 6778 | 60_2398 | 400 | 22 | Firmicutes | Negativicutes | Selenomonadales | Acidaminococcaceae | Acidaminococcus | Acidaminococcus intestini |
| 6810 | 115_4018 | 11 | 8 | Bacteroidetes | Bacteroidia | Bacteroidales | Prevotellaceae | Prevotella | Prevotella copri |
| 6872 | 121_736 | 22 | 5 | Bacteroidetes | Bacteroidia | Bacteroidales | Prevotellaceae | Prevotella | Prevotella copri |
| 6873 | 121_2274 | 7 | 5 | Bacteroidetes | Bacteroidia | Bacteroidales | Prevotellaceae | Prevotella | Prevotella copri |
| 6925 | 130_6733 | 19 | 8 | Bacteroidetes | Bacteroidia | Bacteroidales | Bacteroidaceae | Bacteroides | Bacteroides uniformis |
| 6927 | 49_2248 | 11 | 7 | Firmicutes | Negativicutes | Selenomonadales | Veillonellaceae | Dialister | Dialister invisus |
| 6945 | 49_1572 | 26 | 12 | Firmicutes | Negativicutes | Selenomonadales | Veillonellaceae | Dialister | Dialister invisus |
| 6984 | 49_2883 | 20 | 9 | Firmicutes | Negativicutes | Selenomonadales | Acidaminococcaceae | Acidaminococcus | Acidaminococcus intestini |
| 7022 | 49_4666 | 16 | 10 | Firmicutes | Negativicutes | Selenomonadales | Acidaminococcaceae | Acidaminococcus | Acidaminococcus intestini |
| 7023 | 60_862 | 22 | 10 | Firmicutes | Negativicutes | Selenomonadales | Acidaminococcaceae | Acidaminococcus | Acidaminococcus intestini |
| 7071 | 9_515 | 116 | 36 | Firmicutes | Clostridia | Clostridiales | Ruminococcaceae | Faecalibacterium | Faecalibacterium prausnitzii |
| 7114 | 11_2268 | 28 | 10 | Actinobacteria | Coriobacteriia | Eggerthellales | Eggerthellaceae | Adlercreutzia | Adlercreutzia equolifaciens |
| 7119 | 2_5949 | 77 | 6 | Firmicutes | Clostridia | Clostridiales | Lachnospiraceae | Blautia | Blautia |
| 7127 | 102_2731 | 33 | 6 | Bacteroidetes | Bacteroidia | Bacteroidales | Bacteroidaceae | Bacteroides | Bacteroides ovatus |
| 7152 | 45_8288 | 21 | 6 | Bacteroidetes | Bacteroidia | Bacteroidales | Porphyromonadaceae | Barnesiella | Barnsiella intestinihominis |
| 7154 | 20_8200 | 198 | 28 | Bacteroidetes | Bacteroidia | Bacteroidales | Porphyromonadaceae | Barnesiella | Barnsiella intestinihominis |
| 7159 | 39_2592 | 21 | 14 | Bacteroidetes | Bacteroidia | Bacteroidales | Porphyromonadaceae | Barnesiella | Barnsiella intestinihominis |
| 7161 | 102_1958 | 1107 | 40 | Bacteroidetes | Bacteroidia | Bacteroidales | Porphyromonadaceae | Barnesiella | Barnsiella intestinihominis |
| 7179 | 124_5960 | 10 | 9 | Bacteroidetes | Bacteroidia | Bacteroidales | Porphyromonadaceae | Parabacteroides | Parabacteroides merdae |
| 7183 | 124_3379 | 1498 | 78 | Bacteroidetes | Bacteroidia | Bacteroidales | Porphyromonadaceae | Parabacteroides | Parabacteroides merdae |
| 7192 | 91_10750 | 30 | 6 | Bacteroidetes | Bacteroidia | Bacteroidales | Bacteroidaceae | Bacteroides | Bacteroides ovatus |
| 7203 | 68_5728 | 1609 | 9 | Bacteroidetes | Bacteroidia | Bacteroidales | Bacteroidaceae | Bacteroides | Bacteroides ovatus |
| 7210 | 89_5169 | 704 | 10 | Bacteroidetes | Bacteroidia | Bacteroidales | Bacteroidaceae | Bacteroides | Bacteroides ovatus |
| 7225 | 68_12249 | 9 | 5 | Bacteroidetes | Bacteroidia | Bacteroidales | Bacteroidaceae | Bacteroides | Bacteroides ovatus |
| 7227 | 79_4463 | 51 | 7 | Bacteroidetes | Bacteroidia | Bacteroidales | Bacteroidaceae | Bacteroides | Bacteroides ovatus |
| 7228 | 68_4794 | 744 | 12 | Bacteroidetes | Bacteroidia | Bacteroidales | Bacteroidaceae | Bacteroides | Bacteroides ovatus |
| 7229 | 56_11073 | 1428 | 7 | Bacteroidetes | Bacteroidia | Bacteroidales | Bacteroidaceae | Bacteroides | Bacteroides ovatus |
| 7234 | 79_7953 | 11 | 5 | Bacteroidetes | Bacteroidia | Bacteroidales | Bacteroidaceae | Bacteroides | Bacteroides thetaiotaomicron |
| 7237 | 68_5057 | 54 | 7 | Bacteroidetes | Bacteroidia | Bacteroidales | Bacteroidaceae | Bacteroides | Bacteroides ovatus |
| 7258 | 58_6272 | 638 | 42 | Bacteroidetes | Bacteroidia | Bacteroidales | Bacteroidaceae | Bacteroides | Bacteroides ovatus |
| 7263 | 18_3638 | 6 | 5 | Bacteroidetes | Bacteroidia | Bacteroidales | Bacteroidaceae | Bacteroides | Bacteroides ovatus |
| 7270 | 105_3365 | 6096 | 36 | Bacteroidetes | Bacteroidia | Bacteroidales | Bacteroidaceae | Bacteroides | Bacteroides ovatus |
| 7289 | 68_5810 | 36 | 3 | Bacteroidetes | Bacteroidia | Bacteroidales | Bacteroidaceae | Bacteroides | Bacteroides ovatus |
| 7294 | 124_7357 | 875 | 43 | Bacteroidetes | Bacteroidia | Bacteroidales | Bacteroidaceae | Bacteroides | Bacteroides caccae |
| 7296 | 94_10927 | 37 | 4 | Bacteroidetes | Bacteroidia | Bacteroidales | Bacteroidaceae | Bacteroides | Bacteroides ovatus |
| 7333 | 110_1812 | 27 | 4 | Bacteroidetes | Bacteroidia | Bacteroidales | Bacteroidaceae | Bacteroides | Bacteroides eggerthii |
| 7347 | 99_11860 | 47 | 9 | Bacteroidetes | Bacteroidia | Bacteroidales | Bacteroidaceae | Bacteroides | Bacteroides uniformis |
| 7372 | 111_2603 | 8 | 5 | Bacteroidetes | Bacteroidia | Bacteroidales | Bacteroidaceae | Bacteroides | Bacteroides uniformis |
| 7377 | 62_6312 | 20 | 11 | Bacteroidetes | Bacteroidia | Bacteroidales | Bacteroidaceae | Bacteroides | Bacteroides uniformis |
| 7380 | 90_5102 | 16 | 8 | Bacteroidetes | Bacteroidia | Bacteroidales | Bacteroidaceae | Bacteroides | Bacteroides uniformis |
| 7388 | 101_4670 | 19 | 9 | Bacteroidetes | Bacteroidia | Bacteroidales | Bacteroidaceae | Bacteroides | Bacteroides uniformis |
| 7411 | 100_4115 | 9 | 6 | Bacteroidetes | Bacteroidia | Bacteroidales | Bacteroidaceae | Bacteroides | Bacteroides sp. |
| 7468 | 62_2733 | 8 | 7 | Bacteroidetes | Bacteroidia | Bacteroidales | Bacteroidaceae | Bacteroides | Bacteroides uniformis |
| 7470 | 7_144 | 10 | 5 | Bacteroidetes | Bacteroidia | Bacteroidales | Bacteroidaceae | Bacteroides | Bacteroides ovatus |
| 7471 | 12_3573 | 49 | 7 | Bacteroidetes | Bacteroidia | Bacteroidales | Bacteroidaceae | Bacteroides | Bacteroides fragilis |
| 7488 | 53_5055 | 105 | 12 | Bacteroidetes | Bacteroidia | Bacteroidales | Bacteroidaceae | Bacteroides | Bacteroides vulgatus |
| 7495 | 76_2626 | 15 | 6 | Bacteroidetes | Bacteroidia | Bacteroidales | Bacteroidaceae | Bacteroides | Bacteroides fragilis |
| 7516 | 70_3962 | 108 | 6 | Bacteroidetes | Bacteroidia | Bacteroidales | Prevotellaceae | Prevotella | Prevotella copri |
| 7532 | 16_2470 | 62 | 6 | Bacteroidetes | Bacteroidia | Bacteroidales | Prevotellaceae | Prevotella | Prevotella ruminicola |
| 7539 | 81_6971 | 110 | 7 | Bacteroidetes | Bacteroidia | Bacteroidales | Prevotellaceae | Prevotella | Prevotella copri |
| 7545 | 112_3153 | 59 | 11 | Bacteroidetes | Bacteroidia | Bacteroidales | Prevotellaceae | Prevotella | Prevotella copri |
| 7548 | 128_4067 | 17 | 5 | Firmicutes | Negativicutes | Selenomonadales | Veillonellaceae | Dialister | Dialister invisus |
| 7560 | 123_7652 | 295 | 15 | Bacteroidetes | Bacteroidia | Bacteroidales | Prevotellaceae | Prevotella | Prevotella copri |
| 7577 | 54_3709 | 37 | 4 | Bacteroidetes | Bacteroidia | Bacteroidales | Prevotellaceae | Prevotella | Prevotella copri |
| 7578 | 81_6438 | 313 | 12 | Bacteroidetes | Bacteroidia | Bacteroidales | Prevotellaceae | Prevotella | Prevotella copri |
| 7583 | 112_5348 | 13 | 5 | Bacteroidetes | Bacteroidia | Bacteroidales | Prevotellaceae | Prevotella | Prevotella copri |
| 7598 | 81_6907 | 43 | 9 | Bacteroidetes | Bacteroidia | Bacteroidales | Prevotellaceae | Prevotella | Prevotella copri |
| 7602 | 81_1975 | 72 | 6 | Bacteroidetes | Bacteroidia | Bacteroidales | Prevotellaceae | Prevotella | Prevotella copri |
| 7634 | 71_6177 | 45 | 11 | Verrucomicrobia | Verrucomicrobiae | Verrucomicrobiales | Akkermansiaceae | Akkermansia | Akkermansia muciniphila |
| 7658 | 1_3737 | 28 | 4 | Bacteroidetes | Bacteroidia | Bacteroidales | Prevotellaceae | Prevotella | Prevotella copri |
| 7687 | 55_6976 | 153 | 8 | Firmicutes | Negativicutes | Selenomonadales | Veillonellaceae | Dialister | Dialister invisus |
| 7700 | 63_226 | 5 | 5 | Bacteroidetes | Bacteroidia | Bacteroidales | Bacteroidaceae | Bacteroides | Bacteroides uniformis |
| 7725 | 2_6796 | 7 | 5 | Firmicutes | Clostridia | Clostridiales | Lachnospiraceae | Blautia | Blautia |
| 7807 | 128_6212 | 49 | 7 | Firmicutes | Negativicutes | Selenomonadales | Veillonellaceae | Megasphaera | Megasphaera elsdenii |
| 7837 | 48_2872 | 14 | 5 | Bacteroidetes | Bacteroidia | Bacteroidales | Prevotellaceae | Prevotella | Prevotella copri |
| 7848 | 49_3797 | 115 | 13 | Firmicutes | Negativicutes | Selenomonadales | Acidaminococcaceae | Acidaminococcus | Acidaminococcus intestini |
| 7885 | 63_3155 | 13 | 6 | Bacteroidetes | Bacteroidia | Bacteroidales | Bacteroidaceae | Bacteroides | Bacteroides uniformis |
| 7887 | 121_3288 | 8 | 6 | Firmicutes | Clostridia | Clostridiales | Eubacteriaceae | Eubacterium | Eubacterium sp. |
| 7894 | 60_6572 | 46 | 9 | Firmicutes | Negativicutes | Selenomonadales | Acidaminococcaceae | Acidaminococcus | Acidaminococcus intestini |
| 7900 | 76_5359 | 10 | 5 | Firmicutes | Negativicutes | Selenomonadales | Veillonellaceae | Allisonella | Allisonella histaminiformans |
| 7936 | 37_3010 | 37 | 12 | Firmicutes | Negativicutes | Selenomonadales | Acidaminococcaceae | Acidaminococcus | Acidaminococcus intestini |
| 8020 | 122_3868 | 148 | 13 | Proteobacteria | Betaproteobacteria | Burkholderiales | Sutterellaceae | Parasutterella | Parasutterella excrementihominis |
| 8027 | 44_2052 | 8 | 7 | Proteobacteria | Betaproteobacteria | Burkholderiales | Sutterellaceae | Parasutterella | Parasutterella excrementihominis |
| 8030 | 67_6864 | 34 | 10 | Proteobacteria | Betaproteobacteria | Burkholderiales | Sutterellaceae | Parasutterella | Parasutterella excrementihominis |
| 8033 | 44_3216 | 22 | 4 | Proteobacteria | Betaproteobacteria | Burkholderiales | Sutterellaceae | Parasutterella | Parasutterella excrementihominis |
| 8063 | 106_4250 | 110 | 17 | Firmicutes | Clostridia | Clostridiales | Lachnospiraceae | Blautia | Blautia |
| 8106 | 32_3302 | 125 | 11 | Bacteroidetes | Bacteroidia | Bacteroidales | Porphyromonadaceae | Barnesiella | Barnsiella intestinihominis |
| 8108 | 27_568 | 29 | 2 | Bacteroidetes | Bacteroidia | Bacteroidales | Porphyromonadaceae | Barnesiella | Barnsiella intestinihominis |
| 8109 | 16_1855 | 91 | 18 | Bacteroidetes | Bacteroidia | Bacteroidales | Porphyromonadaceae | Barnesiella | Barnsiella intestinihominis |
| 8133 | 2_1347 | 7 | 6 | Bacteroidetes | Bacteroidia | Bacteroidales | Porphyromonadaceae | Parabacteroides | Parabacteroides merdae |
| 8138 | 101_3123 | 1259 | 80 | Bacteroidetes | Bacteroidia | Bacteroidales | Porphyromonadaceae | Parabacteroides | Parabacteroides merdae |
| 8141 | 1_3725 | 11 | 10 | Bacteroidetes | Bacteroidia | Bacteroidales | Porphyromonadaceae | Parabacteroides | Parabacteroides merdae |
| 8179 | 68_12196 | 39 | 8 | Bacteroidetes | Bacteroidia | Bacteroidales | Bacteroidaceae | Bacteroides | Bacteroides ovatus |
| 8181 | 79_1470 | 32 | 5 | Bacteroidetes | Bacteroidia | Bacteroidales | Prevotellaceae | Paraprevotella | Paraprevotella clara |
| 8184 | 68_6125 | 25 | 4 | Bacteroidetes | Bacteroidia | Bacteroidales | Bacteroidaceae | Bacteroides | Bacteroides ovatus |
| 8185 | 91_4557 | 23 | 6 | Bacteroidetes | Bacteroidia | Bacteroidales | Bacteroidaceae | Bacteroides | Bacteroides ovatus |
| 8188 | 91_5119 | 132 | 7 | Bacteroidetes | Bacteroidia | Bacteroidales | Bacteroidaceae | Bacteroides | Bacteroides ovatus |
| 8198 | 89_5167 | 388 | 9 | Bacteroidetes | Bacteroidia | Bacteroidales | Bacteroidaceae | Bacteroides | Bacteroides ovatus |
| 8242 | 82_10781 | 15 | 5 | Bacteroidetes | Bacteroidia | Bacteroidales | Bacteroidaceae | Bacteroides | Bacteroides ovatus |
| 8250 | 56_4927 | 6 | 5 | Bacteroidetes | Bacteroidia | Bacteroidales | Bacteroidaceae | Bacteroides | Bacteroides ovatus |
| 8259 | 63_6402 | 44 | 14 | Bacteroidetes | Bacteroidia | Bacteroidales | Bacteroidaceae | Bacteroides | Bacteroides thetaiotaomicron |
| 8264 | 105_1609 | 38 | 5 | Bacteroidetes | Bacteroidia | Bacteroidales | Bacteroidaceae | Bacteroides | Bacteroides thetaiotaomicron |
| 8266 | 77_4235 | 47 | 5 | Bacteroidetes | Bacteroidia | Bacteroidales | Bacteroidaceae | Bacteroides | Bacteroides ovatus |
| 8282 | 108_5099 | 197 | 15 | Bacteroidetes | Bacteroidia | Bacteroidales | Bacteroidaceae | Bacteroides | Bacteroides faecis |
| 8314 | 110_5286 | 3152 | 27 | Bacteroidetes | Bacteroidia | Bacteroidales | Bacteroidaceae | Bacteroides | Bacteroides eggerthii |
| 8322 | 110_2329 | 302 | 9 | Bacteroidetes | Bacteroidia | Bacteroidales | Bacteroidaceae | Bacteroides | Bacteroides eggerthii |
| 8333 | 108_2155 | 71 | 5 | Bacteroidetes | Bacteroidia | Bacteroidales | Bacteroidaceae | Bacteroides | Bacteroides finegoldii |
| 8371 | 111_5963 | 22 | 14 | Bacteroidetes | Bacteroidia | Bacteroidales | Bacteroidaceae | Bacteroides | Bacteroides uniformis |
| 8377 | 99_10123 | 8243 | 96 | Bacteroidetes | Bacteroidia | Bacteroidales | Bacteroidaceae | Bacteroides | Bacteroides uniformis |
| 8378 | 10_6335 | 170 | 33 | Bacteroidetes | Bacteroidia | Bacteroidales | Bacteroidaceae | Bacteroides | Bacteroides uniformis |
| 8421 | 119_1347 | 561 | 51 | Bacteroidetes | Bacteroidia | Bacteroidales | Porphyromonadaceae | Parabacteroides | Parabacteroides distasonis |
| 8425 | 91_8011 | 36 | 3 | Bacteroidetes | Bacteroidia | Bacteroidales | Bacteroidaceae | Bacteroides | Bacteroides ovatus |
| 8448 | 114_6947 | 177 | 24 | Bacteroidetes | Bacteroidia | Bacteroidales | Bacteroidaceae | Bacteroides | Bacteroides ovatus |
| 8469 | 50_1535 | 22 | 10 | Bacteroidetes | Bacteroidia | Bacteroidales | Prevotellaceae | Prevotella | Prevotella copri |
| 8489 | 23_191 | 78 | 19 | Bacteroidetes | Bacteroidia | Bacteroidales | Prevotellaceae | Paraprevotella | Paraprevotella clara |
| 8497 | 50_2739 | 260 | 37 | Bacteroidetes | Bacteroidia | Bacteroidales | Rikenellaceae | Alistipes | Alistipes sp. |
| 8510 | 31_1834 | 9 | 5 | Bacteroidetes | Bacteroidia | Bacteroidales | Prevotellaceae | Prevotella | Prevotella copri |
| 8516 | 104_1585 | 31 | 5 | Bacteroidetes | Bacteroidia | Bacteroidales | Prevotellaceae | Prevotella | Prevotella sp. |
| 8521 | 9_2795 | 16 | 5 | NULL | NULL | NULL | NULL | NULL | NULL |
| 8529 | 16_2922 | 551 | 23 | Bacteroidetes | Bacteroidia | Bacteroidales | Prevotellaceae | Prevotella | Prevotella ruminicola |
| 8530 | 115_1629 | 70 | 11 | Bacteroidetes | Bacteroidia | Bacteroidales | Prevotellaceae | Prevotella | Prevotella ruminicola |
| 8532 | 35_6572 | 11 | 5 | Bacteroidetes | Bacteroidia | Bacteroidales | Prevotellaceae | Prevotella | Prevotella sp. |
| 8536 | 108_2288 | 636 | 7 | Bacteroidetes | Bacteroidia | Bacteroidales | Prevotellaceae | Prevotella | Prevotella copri |
| 8537 | 9_4972 | 35 | 4 | Bacteroidetes | Bacteroidia | Bacteroidales | Prevotellaceae | Prevotella | Prevotella copri |
| 8538 | 129_5998 | 557 | 13 | Bacteroidetes | Bacteroidia | Bacteroidales | Prevotellaceae | Prevotella | Prevotella copri |
| 8541 | 108_7268 | 178 | 8 | Bacteroidetes | Bacteroidia | Bacteroidales | Prevotellaceae | Prevotella | Prevotella copri |
| 8542 | 81_6928 | 30 | 5 | Bacteroidetes | Bacteroidia | Bacteroidales | Prevotellaceae | Prevotella | Prevotella copri |
| 8549 | 43_2229 | 15 | 6 | Bacteroidetes | Bacteroidia | Bacteroidales | Prevotellaceae | Prevotella | Prevotella sp. |
| 8569 | 123_7792 | 692 | 21 | Bacteroidetes | Bacteroidia | Bacteroidales | Prevotellaceae | Prevotella | Prevotella copri |
| 8574 | 16_2855 | 11 | 6 | Bacteroidetes | Bacteroidia | Bacteroidales | Prevotellaceae | Prevotella | Prevotella copri |
| 8588 | 21_7538 | 218 | 11 | Bacteroidetes | Bacteroidia | Bacteroidales | Prevotellaceae | Prevotella | Prevotella copri |
| 8591 | 70_2608 | 389 | 9 | Bacteroidetes | Bacteroidia | Bacteroidales | Prevotellaceae | Prevotella | Prevotella copri |
| 8595 | 121_5868 | 180 | 9 | Bacteroidetes | Bacteroidia | Bacteroidales | Prevotellaceae | Prevotella | Prevotella copri |
| 8597 | 1_3517 | 296 | 8 | Bacteroidetes | Bacteroidia | Bacteroidales | Prevotellaceae | Prevotella | Prevotella copri |
| 8600 | 33_6371 | 8 | 5 | Bacteroidetes | Bacteroidia | Bacteroidales | Prevotellaceae | Prevotella | Prevotella copri |
| 8605 | 125_3352 | 63 | 7 | Bacteroidetes | Bacteroidia | Bacteroidales | Prevotellaceae | Prevotella | Prevotella copri |
| 8612 | 23_4918 | 8 | 5 | Bacteroidetes | Bacteroidia | Bacteroidales | Prevotellaceae | Prevotella | Prevotella sp. |
| 8649 | 101_4978 | 264 | 17 | Bacteroidetes | Bacteroidia | Bacteroidales | Bacteroidaceae | Bacteroides | Bacteroides thetaiotaomicron |
| 8671 | 4_773 | 34 | 22 | Bacteroidetes | Bacteroidia | Bacteroidales | Porphyromonadaceae | Parabacteroides | Parabacteroides merdae |
| 8691 | 43_2036 | 326 | 10 | Bacteroidetes | Bacteroidia | Bacteroidales | Prevotellaceae | Prevotella | Prevotella copri |
| 8692 | 131_194 | 86 | 9 | Bacteroidetes | Bacteroidia | Bacteroidales | Bacteroidaceae | Bacteroides | Bacteroides ovatus |
| 8713 | 66_2328 | 29 | 9 | Firmicutes | Clostridia | Clostridiales | Lactobacillaceae | Lactobacillus | Lactobacillus ruminis |
| 8847 | 112_443 | 19 | 5 | Bacteroidetes | Bacteroidia | Bacteroidales | Prevotellaceae | Prevotella | Prevotella copri |
| 8850 | 60_4131 | 17 | 5 | Firmicutes | Clostridia | Clostridiales | Lachnospiraceae | Blautia | Blautia |
| 8902 | 95_2010 | 36 | 16 | Firmicutes | Negativicutes | Selenomonadales | Veillonellaceae | Dialister | Dialister invisus |
| 8924 | 55_5105 | 28 | 11 | Firmicutes | Negativicutes | Selenomonadales | Veillonellaceae | Allisonella | Allisonella histaminiformans |
| 8925 | 30_8516 | 8 | 7 | Firmicutes | Negativicutes | Selenomonadales | Veillonellaceae | Allisonella | Allisonella histaminiformans |
| 8940 | 55_6804 | 85 | 7 | Firmicutes | Negativicutes | Selenomonadales | Veillonellaceae | Dialister | Dialister invisus |
| 9008 | 32_3490 | 78 | 34 | Firmicutes | Clostridia | Clostridiales | Ruminococcaceae | Faecalibacterium | Faecalibacterium prausnitzii |
| 9018 | 132_1003 | 160 | 26 | Firmicutes | Clostridia | Clostridiales | Ruminococcaceae | Faecalibacterium | Faecalibacterium prausnitzii |
| 9033 | 23_2128 | 22 | 8 | Proteobacteria | Betaproteobacteria | Burkholderiales | Sutterellaceae | Parasutterella | Parasutterella excrementihominis |
| 9048 | 55_5325 | 15 | 5 | Proteobacteria | Betaproteobacteria | Burkholderiales | Sutterellaceae | Parasutterella | Parasutterella excrementihominis |
| 9057 | 53_2653 | 238 | 43 | Firmicutes | Clostridia | Clostridiales | Lachnospiraceae | Blautia | Blautia |
| 9118 | 41_3813 | 6 | 6 | Proteobacteria | Gammaproteobacteria | Enterobacteriales | Enterobacteriaceae | Escherichia/Shigella | Escherichia/Shigella |
| 9183 | 125_3531 | 718 | 86 | Bacteroidetes | Bacteroidia | Bacteroidales | Porphyromonadaceae | Parabacteroides | Parabacteroides merdae |
| 9224 | 79_8700 | 44 | 6 | Bacteroidetes | Bacteroidia | Bacteroidales | Bacteroidaceae | Bacteroides | Bacteroides sp. |
| 9231 | 100_8490 | 519 | 11 | Bacteroidetes | Bacteroidia | Bacteroidales | Bacteroidaceae | Bacteroides | Bacteroides ovatus |
| 9235 | 100_9469 | 203 | 10 | Bacteroidetes | Bacteroidia | Bacteroidales | Bacteroidaceae | Bacteroides | Bacteroides ovatus |
| 9244 | 79_8792 | 50 | 8 | Bacteroidetes | Bacteroidia | Bacteroidales | Bacteroidaceae | Bacteroides | Bacteroides ovatus |
| 9246 | 15_2671 | 193 | 12 | Bacteroidetes | Bacteroidia | Bacteroidales | Bacteroidaceae | Bacteroides | Bacteroides ovatus |
| 9259 | 91_8163 | 20 | 4 | Bacteroidetes | Bacteroidia | Bacteroidales | Bacteroidaceae | Bacteroides | Bacteroides ovatus |
| 9260 | 79_4371 | 14 | 5 | Bacteroidetes | Bacteroidia | Bacteroidales | Bacteroidaceae | Bacteroides | Bacteroides ovatus |
| 9276 | 89_9882 | 21 | 2 | Bacteroidetes | Bacteroidia | Bacteroidales | Bacteroidaceae | Bacteroides | Bacteroides ovatus |
| 9298 | 105_7315 | 20 | 4 | Bacteroidetes | Bacteroidia | Bacteroidales | Bacteroidaceae | Bacteroides | Bacteroides caccae |
| 9299 | 105_4739 | 21 | 3 | Bacteroidetes | Bacteroidia | Bacteroidales | Bacteroidaceae | Bacteroides | Bacteroides ovatus |
| 9339 | 82_5759 | 62 | 9 | Bacteroidetes | Bacteroidia | Bacteroidales | Bacteroidaceae | Bacteroides | Bacteroides ovatus |
| 9343 | 68_5626 | 17 | 5 | Bacteroidetes | Bacteroidia | Bacteroidales | Bacteroidaceae | Bacteroides | Bacteroides ovatus |
| 9347 | 68_11361 | 67 | 12 | Bacteroidetes | Bacteroidia | Bacteroidales | Bacteroidaceae | Bacteroides | Bacteroides ovatus |
| 9355 | 101_4709 | 7 | 5 | Bacteroidetes | Bacteroidia | Bacteroidales | Bacteroidaceae | Bacteroides | Bacteroides thetaiotaomicron |
| 9357 | 63_3372 | 42 | 6 | Bacteroidetes | Bacteroidia | Bacteroidales | Bacteroidaceae | Bacteroides | Bacteroides thetaiotaomicron |
| 9359 | 122_3442 | 256 | 26 | Bacteroidetes | Bacteroidia | Bacteroidales | Bacteroidaceae | Bacteroides | Bacteroides thetaiotaomicron |
| 9360 | 68_3325 | 38 | 4 | Bacteroidetes | Bacteroidia | Bacteroidales | Bacteroidaceae | Bacteroides | Bacteroides ovatus |
| 9381 | 105_3722 | 17 | 9 | Bacteroidetes | Bacteroidia | Bacteroidales | Bacteroidaceae | Bacteroides | Bacteroides caccae |
| 9394 | 30_10403 | 123 | 6 | Bacteroidetes | Bacteroidia | Bacteroidales | Bacteroidaceae | Bacteroides | Bacteroides fragilis |
| 9399 | 94_4482 | 71 | 5 | Bacteroidetes | Bacteroidia | Bacteroidales | Bacteroidaceae | Bacteroides | Bacteroides ovatus |
| 9409 | 94_8070 | 21 | 2 | Bacteroidetes | Bacteroidia | Bacteroidales | Bacteroidaceae | Bacteroides | Bacteroides ovatus |
| 9421 | 130_4909 | 3913 | 31 | Bacteroidetes | Bacteroidia | Bacteroidales | Bacteroidaceae | Bacteroides | Bacteroides eggerthii |
| 9423 | 110_5453 | 75 | 9 | Bacteroidetes | Bacteroidia | Bacteroidales | Bacteroidaceae | Bacteroides | Bacteroides eggerthii |
| 9428 | 110_5460 | 991 | 13 | Bacteroidetes | Bacteroidia | Bacteroidales | Bacteroidaceae | Bacteroides | Bacteroides eggerthii |
| 9435 | 63_3031 | 255 | 13 | Bacteroidetes | Bacteroidia | Bacteroidales | Bacteroidaceae | Bacteroides | Bacteroides uniformis |
| 9458 | 99_13153 | 12029 | 61 | Bacteroidetes | Bacteroidia | Bacteroidales | Bacteroidaceae | Bacteroides | Bacteroides uniformis |
| 9459 | 99_3362 | 36 | 7 | Bacteroidetes | Bacteroidia | Bacteroidales | Bacteroidaceae | Bacteroides | Bacteroides uniformis |
| 9461 | 131_4804 | 145 | 31 | Bacteroidetes | Bacteroidia | Bacteroidales | Bacteroidaceae | Bacteroides | Bacteroides uniformis |
| 9465 | 111_8924 | 29 | 11 | Bacteroidetes | Bacteroidia | Bacteroidales | Bacteroidaceae | Bacteroides | Bacteroides uniformis |
| 9468 | 99_13130 | 15 | 5 | Bacteroidetes | Bacteroidia | Bacteroidales | Bacteroidaceae | Bacteroides | Bacteroides uniformis |
| 9482 | 40_2377 | 52 | 19 | Bacteroidetes | Bacteroidia | Bacteroidales | Bacteroidaceae | Bacteroides | Bacteroides uniformis |
| 9494 | 99_6209 | 20996 | 105 | Bacteroidetes | Bacteroidia | Bacteroidales | Bacteroidaceae | Bacteroides | Bacteroides uniformis |
| 9498 | 2_6170 | 59 | 21 | Bacteroidetes | Bacteroidia | Bacteroidales | Bacteroidaceae | Bacteroides | Bacteroides uniformis |
| 9499 | 67_8828 | 6 | 5 | Bacteroidetes | Bacteroidia | Bacteroidales | Bacteroidaceae | Bacteroides | Bacteroides uniformis |
| 9500 | 101_4734 | 12 | 6 | Bacteroidetes | Bacteroidia | Bacteroidales | Bacteroidaceae | Bacteroides | Bacteroides uniformis |
| 9509 | 65_4259 | 7 | 5 | Bacteroidetes | Bacteroidia | Bacteroidales | Bacteroidaceae | Bacteroides | Bacteroides uniformis |
| 9520 | 101_2502 | 5 | 5 | Firmicutes | Clostridia | Clostridiales | Lachnospiraceae | Blautia | Blautia |
| 9537 | 79_8458 | 12 | 10 | Bacteroidetes | Bacteroidia | Bacteroidales | Bacteroidaceae | Bacteroides | Bacteroides sp. |
| 9538 | 68_5878 | 12 | 6 | Bacteroidetes | Bacteroidia | Bacteroidales | Bacteroidaceae | Bacteroides | Bacteroides finegoldii |
| 9539 | 68_7489 | 328 | 33 | Bacteroidetes | Bacteroidia | Bacteroidales | Bacteroidaceae | Bacteroides | Bacteroides sp. |
| 9552 | 100_4394 | 2270 | 65 | Bacteroidetes | Bacteroidia | Bacteroidales | Bacteroidaceae | Bacteroides | Bacteroides sp. |
| 9566 | 96_9946 | 35 | 17 | Bacteroidetes | Bacteroidia | Bacteroidales | Bacteroidaceae | Bacteroides | Bacteroides sp. |
| 9575 | 79_2850 | 41 | 4 | Bacteroidetes | Bacteroidia | Bacteroidales | Bacteroidaceae | Bacteroides | Bacteroides finegoldii |
| 9625 | 91_10982 | 47 | 9 | Bacteroidetes | Bacteroidia | Bacteroidales | Bacteroidaceae | Bacteroides | Bacteroides ovatus |
| 9644 | 33_3199 | 11 | 8 | Bacteroidetes | Bacteroidia | Bacteroidales | Prevotellaceae | Prevotella | Prevotella copri |
| 9666 | 102_6159 | 9 | 6 | Bacteroidetes | Bacteroidia | Bacteroidales | Bacteroidaceae | Bacteroides | Bacteroides ovatus |
| 9682 | 31_7123 | 39 | 6 | Bacteroidetes | Bacteroidia | Bacteroidales | Prevotellaceae | Prevotella | Prevotella copri |
| 9705 | 20_4529 | 11 | 5 | NULL | NULL | NULL | NULL | NULL | NULL |
| 9707 | 62_482 | 9 | 5 | NULL | NULL | NULL | NULL | NULL | NULL |
| 9714 | 20_1017 | 74 | 6 | Bacteroidetes | Bacteroidia | Bacteroidales | Prevotellaceae | Prevotella | Prevotella sp. |
| 9715 | 97_353 | 2171 | 10 | Bacteroidetes | Bacteroidia | Bacteroidales | Prevotellaceae | Prevotella | Prevotella sp. |
| 9725 | 37_6230 | 72 | 17 | Bacteroidetes | Bacteroidia | Bacteroidales | Prevotellaceae | Prevotella | Prevotella ruminicola |
| 9726 | 21_4182 | 6 | 6 | Bacteroidetes | Bacteroidia | Bacteroidales | Prevotellaceae | Prevotella | Prevotella ruminicola |
| 9728 | 117_2450 | 11 | 7 | Bacteroidetes | Bacteroidia | Bacteroidales | Prevotellaceae | Prevotella | Prevotella ruminicola |
| 9754 | 81_1932 | 27 | 6 | Bacteroidetes | Bacteroidia | Bacteroidales | Prevotellaceae | Prevotella | Prevotella copri |
| 9760 | 115_4165 | 232 | 17 | Bacteroidetes | Bacteroidia | Bacteroidales | Prevotellaceae | Prevotella | Prevotella copri |
| 9767 | 21_7383 | 111 | 7 | Bacteroidetes | Bacteroidia | Bacteroidales | Prevotellaceae | Prevotella | Prevotella copri |
| 9794 | 123_2981 | 1804 | 25 | Bacteroidetes | Bacteroidia | Bacteroidales | Prevotellaceae | Prevotella | Prevotella copri |
| 9798 | 33_5788 | 110 | 11 | Bacteroidetes | Bacteroidia | Bacteroidales | Prevotellaceae | Prevotella | Prevotella copri |
| 9804 | 81_6445 | 30 | 4 | Bacteroidetes | Bacteroidia | Bacteroidales | Prevotellaceae | Prevotella | Prevotella copri |
| 9805 | 43_2053 | 734 | 15 | Bacteroidetes | Bacteroidia | Bacteroidales | Prevotellaceae | Prevotella | Prevotella copri |
| 9806 | 93_6123 | 805 | 15 | Bacteroidetes | Bacteroidia | Bacteroidales | Prevotellaceae | Prevotella | Prevotella copri |
| 9810 | 81_2895 | 55 | 7 | Bacteroidetes | Bacteroidia | Bacteroidales | Prevotellaceae | Prevotella | Prevotella copri |
| 9814 | 54_6912 | 176 | 15 | Bacteroidetes | Bacteroidia | Bacteroidales | Prevotellaceae | Prevotella | Prevotella copri |
| 9815 | 81_3495 | 321 | 13 | Bacteroidetes | Bacteroidia | Bacteroidales | Prevotellaceae | Prevotella | Prevotella copri |
| 9817 | 43_3851 | 29 | 9 | Bacteroidetes | Bacteroidia | Bacteroidales | Prevotellaceae | Prevotella | Prevotella copri |
| 9818 | 43_2168 | 32 | 6 | Bacteroidetes | Bacteroidia | Bacteroidales | Prevotellaceae | Prevotella | Prevotella copri |
| 9820 | 54_6431 | 132 | 4 | Bacteroidetes | Bacteroidia | Bacteroidales | Prevotellaceae | Prevotella | Prevotella copri |
| 9823 | 81_2838 | 280 | 12 | Bacteroidetes | Bacteroidia | Bacteroidales | Prevotellaceae | Prevotella | Prevotella copri |
| 9830 | 81_5851 | 338 | 14 | Bacteroidetes | Bacteroidia | Bacteroidales | Prevotellaceae | Prevotella | Prevotella copri |
| 9836 | 121_379 | 32 | 12 | Bacteroidetes | Bacteroidia | Bacteroidales | Prevotellaceae | Prevotella | Prevotella copri |
| 9838 | 21_1913 | 41 | 4 | Bacteroidetes | Bacteroidia | Bacteroidales | Prevotellaceae | Prevotella | Prevotella copri |
| 9843 | 121_6655 | 20 | 6 | Bacteroidetes | Bacteroidia | Bacteroidales | Prevotellaceae | Prevotella | Prevotella copri |
| 9844 | 121_759 | 38 | 4 | Bacteroidetes | Bacteroidia | Bacteroidales | Prevotellaceae | Prevotella | Prevotella copri |
| 9845 | 121_1280 | 38 | 6 | Bacteroidetes | Bacteroidia | Bacteroidales | Prevotellaceae | Prevotella | Prevotella copri |
| 9853 | 1_1922 | 42 | 5 | Bacteroidetes | Bacteroidia | Bacteroidales | Prevotellaceae | Prevotella | Prevotella copri |
| 9857 | 125_6828 | 247 | 19 | Bacteroidetes | Bacteroidia | Bacteroidales | Prevotellaceae | Prevotella | Prevotella copri |
| 9861 | 81_2239 | 22 | 5 | Bacteroidetes | Bacteroidia | Bacteroidales | Prevotellaceae | Prevotella | Prevotella copri |
| 9867 | 121_6614 | 141 | 9 | Bacteroidetes | Bacteroidia | Bacteroidales | Prevotellaceae | Prevotella | Prevotella copri |
| 9874 | 121_6911 | 82 | 8 | Bacteroidetes | Bacteroidia | Bacteroidales | Prevotellaceae | Prevotella | Prevotella sp. |
| 9910 | 125_5289 | 27 | 5 | Firmicutes | Clostridia | Clostridiales | Peptococcaceae | Peptococcus | Peptococcus sp. |
| 9923 | 79_6252 | 86 | 13 | Bacteroidetes | Bacteroidia | Bacteroidales | Bacteroidaceae | Bacteroides | Bacteroides finegoldii |
| 9940 | 89_10094 | 28 | 6 | Bacteroidetes | Bacteroidia | Bacteroidales | Bacteroidaceae | Bacteroides | Bacteroides ovatus |
| 9960 | 108_5887 | 84 | 16 | Bacteroidetes | Bacteroidia | Bacteroidales | Bacteroidaceae | Bacteroides | Bacteroides faecis |
| 9973 | 71_8900 | 8 | 5 | Bacteroidetes | Bacteroidia | Bacteroidales | Prevotellaceae | Prevotella | Prevotella ruminicola |
| 10053 | 1_2335 | 27 | 7 | Firmicutes | Negativicutes | Selenomonadales | Acidaminococcaceae | Acidaminococcus | Acidaminococcus fermentans |
| 10058 | 1_615 | 7 | 6 | Bacteroidetes | Bacteroidia | Bacteroidales | Prevotellaceae | Prevotella | Prevotella stercorea |
| 10114 | 49_1447 | 25 | 5 | Firmicutes | Negativicutes | Selenomonadales | Acidaminococcaceae | Acidaminococcus | Acidaminococcus intestini |
| 10140 | 117_4111 | 11 | 5 | Firmicutes | Clostridia | Clostridiales | Lachnospiraceae | Blautia | Blautia |
| 10148 | 128_5333 | 30 | 6 | Firmicutes | Negativicutes | Selenomonadales | Veillonellaceae | Dialister | Dialister invisus |
| 10175 | 49_1427 | 47 | 10 | Firmicutes | Negativicutes | Selenomonadales | Acidaminococcaceae | Acidaminococcus | Acidaminococcus intestini |
| 10195 | 49_4672 | 30 | 4 | Firmicutes | Negativicutes | Selenomonadales | Veillonellaceae | Dialister | Dialister invisus |
| 10232 | 55_5691 | 357 | 8 | Firmicutes | Negativicutes | Selenomonadales | Veillonellaceae | Dialister | Dialister invisus |
| 10248 | 49_1871 | 7 | 5 | Bacteroidetes | Bacteroidia | Bacteroidales | Bacteroidaceae | Bacteroides | Bacteroides uniformis |
| 10297 | 130_1373 | 12 | 8 | Bacteroidetes | Bacteroidia | Bacteroidales | Bacteroidaceae | Bacteroides | Bacteroides uniformis |
| 10322 | 52_8555 | 10 | 7 | Firmicutes | Bacilli | Lactobacillales | Streptococcaceae | Lactococcus | Lactococcus lactis |
| 10328 | 55_4017 | 289 | 31 | Firmicutes | Clostridia | Clostridiales | Ruminococcaceae | Faecalibacterium | Faecalibacterium prausnitzii |
| 10340 | 80_4547 | 43 | 14 | Firmicutes | Clostridia | Clostridiales | Ruminococcaceae | Faecalibacterium | Faecalibacterium prausnitzii |
| 10341 | 132_2057 | 172 | 39 | Firmicutes | Clostridia | Clostridiales | Ruminococcaceae | Faecalibacterium | Faecalibacterium prausnitzii |
| 10373 | 71_1962 | 174 | 16 | NULL | NULL | NULL | NULL | NULL | NULL |
| 10387 | 22_4729 | 39 | 20 | Firmicutes | Clostridia | Clostridiales | Lachnospiraceae | Blautia | Blautia |
| 10389 | 19_5621 | 55 | 10 | NULL | NULL | NULL | NULL | NULL | NULL |
| 10396 | 110_3668 | 6 | 5 | Firmicutes | Clostridia | Clostridiales | Ruminococcaceae | Ruminiclostridium | Clostridium leptum |
| 10398 | 18_5199 | 6 | 5 | Firmicutes | Negativicutes | Selenomonadales | Veillonellaceae | Dialister | Dialister invisus |
| 10414 | 101_1841 | 22 | 4 | Bacteroidetes | Bacteroidia | Bacteroidales | Bacteroidaceae | Bacteroides | Bacteroides uniformis |
| 10415 | 68_5924 | 8 | 5 | Bacteroidetes | Bacteroidia | Bacteroidales | Bacteroidaceae | Bacteroides | Bacteroides ovatus |
| 10439 | 94_4115 | 92 | 6 | Bacteroidetes | Bacteroidia | Bacteroidales | Bacteroidaceae | Bacteroides | Bacteroides thetaiotaomicron |
| 10444 | 3_1520 | 27 | 7 | Bacteroidetes | Bacteroidia | Bacteroidales | Prevotellaceae | Prevotella | Prevotella copri |
| 10490 | 101_2622 | 76 | 32 | Bacteroidetes | Bacteroidia | Bacteroidales | Porphyromonadaceae | Parabacteroides | Parabacteroides merdae |
| 10495 | 122_1964 | 11 | 9 | Bacteroidetes | Bacteroidia | Bacteroidales | Porphyromonadaceae | Parabacteroides | Parabacteroides merdae |
| 10497 | 38_5990 | 35 | 7 | Bacteroidetes | Bacteroidia | Bacteroidales | Porphyromonadaceae | Parabacteroides | Parabacteroides merdae |
| 10499 | 16_3454 | 27 | 18 | Bacteroidetes | Bacteroidia | Bacteroidales | Porphyromonadaceae | Parabacteroides | Parabacteroides merdae |
| 10503 | 7_3791 | 16 | 13 | Bacteroidetes | Bacteroidia | Bacteroidales | Porphyromonadaceae | Parabacteroides | Parabacteroides merdae |
| 10506 | 1_1118 | 7 | 7 | Bacteroidetes | Bacteroidia | Bacteroidales | Porphyromonadaceae | Parabacteroides | Parabacteroides merdae |
| 10509 | 60_6867 | 8 | 8 | Bacteroidetes | Bacteroidia | Bacteroidales | Porphyromonadaceae | Parabacteroides | Parabacteroides merdae |
| 10510 | 13_1368 | 10 | 6 | Bacteroidetes | Bacteroidia | Bacteroidales | Porphyromonadaceae | Parabacteroides | Parabacteroides merdae |
| 10515 | 107_2098 | 55 | 30 | Bacteroidetes | Bacteroidia | Bacteroidales | Porphyromonadaceae | Parabacteroides | Parabacteroides merdae |
| 10522 | 69_842 | 14 | 10 | Bacteroidetes | Bacteroidia | Bacteroidales | Prevotellaceae | Prevotella | Prevotella copri |
| 10528 | 58_8137 | 42 | 2 | Bacteroidetes | Bacteroidia | Bacteroidales | Bacteroidaceae | Bacteroides | Bacteroides ovatus |
| 10530 | 81_3352 | 43 | 4 | Bacteroidetes | Bacteroidia | Bacteroidales | Prevotellaceae | Prevotella | Prevotella copri |
| 10541 | 68_3271 | 20 | 4 | Bacteroidetes | Bacteroidia | Bacteroidales | Bacteroidaceae | Bacteroides | Bacteroides ovatus |
| 10549 | 105_2075 | 927 | 46 | Bacteroidetes | Bacteroidia | Bacteroidales | Bacteroidaceae | Bacteroides | Bacteroides sp. |
| 10563 | 82_4728 | 31 | 3 | Bacteroidetes | Bacteroidia | Bacteroidales | Bacteroidaceae | Bacteroides | Bacteroides ovatus |
| 10568 | 89_10393 | 8 | 6 | Bacteroidetes | Bacteroidia | Bacteroidales | Bacteroidaceae | Bacteroides | Bacteroides ovatus |
| 10569 | 68_1831 | 25 | 6 | Bacteroidetes | Bacteroidia | Bacteroidales | Bacteroidaceae | Bacteroides | Bacteroides ovatus |
| 10598 | 131_9512 | 20 | 6 | Bacteroidetes | Bacteroidia | Bacteroidales | Bacteroidaceae | Bacteroides | Bacteroides ovatus |
| 10608 | 79_7203 | 30 | 4 | Bacteroidetes | Bacteroidia | Bacteroidales | Bacteroidaceae | Bacteroides | Bacteroides ovatus |
| 10620 | 91_11009 | 14 | 5 | Bacteroidetes | Bacteroidia | Bacteroidales | Bacteroidaceae | Bacteroides | Bacteroides ovatus |
| 10640 | 91_10779 | 39 | 7 | Bacteroidetes | Bacteroidia | Bacteroidales | Bacteroidaceae | Bacteroides | Bacteroides ovatus |
| 10645 | 89_8293 | 13 | 5 | Bacteroidetes | Bacteroidia | Bacteroidales | Bacteroidaceae | Bacteroides | Bacteroides ovatus |
| 10657 | 82_6600 | 48 | 6 | Bacteroidetes | Bacteroidia | Bacteroidales | Bacteroidaceae | Bacteroides | Bacteroides ovatus |
| 10668 | 70_3429 | 14 | 9 | Bacteroidetes | Bacteroidia | Bacteroidales | Bacteroidaceae | Bacteroides | Bacteroides thetaiotaomicron |
| 10669 | 82_9953 | 2684 | 12 | Bacteroidetes | Bacteroidia | Bacteroidales | Bacteroidaceae | Bacteroides | Bacteroides ovatus |
| 10682 | 94_5556 | 19 | 7 | Bacteroidetes | Bacteroidia | Bacteroidales | Bacteroidaceae | Bacteroides | Bacteroides ovatus |
| 10709 | 56_5546 | 60 | 8 | Bacteroidetes | Bacteroidia | Bacteroidales | Prevotellaceae | Paraprevotella | Paraprevotella clara |
| 10714 | 91_5464 | 14 | 6 | Bacteroidetes | Bacteroidia | Bacteroidales | Bacteroidaceae | Bacteroides | Bacteroides thetaiotaomicron |
| 10715 | 79_8757 | 163 | 7 | Bacteroidetes | Bacteroidia | Bacteroidales | Bacteroidaceae | Bacteroides | Bacteroides ovatus |
| 10716 | 79_788 | 329 | 24 | Bacteroidetes | Bacteroidia | Bacteroidales | Bacteroidaceae | Bacteroides | Bacteroides thetaiotaomicron |
| 10717 | 68_5990 | 259 | 21 | Bacteroidetes | Bacteroidia | Bacteroidales | Bacteroidaceae | Bacteroides | Bacteroides thetaiotaomicron |
| 10718 | 79_4468 | 135 | 26 | Bacteroidetes | Bacteroidia | Bacteroidales | Bacteroidaceae | Bacteroides | Bacteroides thetaiotaomicron |
| 10719 | 101_398 | 35 | 10 | Bacteroidetes | Bacteroidia | Bacteroidales | Bacteroidaceae | Bacteroides | Bacteroides thetaiotaomicron |
| 10725 | 130_7109 | 7 | 7 | Bacteroidetes | Bacteroidia | Bacteroidales | Bacteroidaceae | Bacteroides | Bacteroides thetaiotaomicron |
| 10726 | 81_3413 | 11 | 8 | Bacteroidetes | Bacteroidia | Bacteroidales | Bacteroidaceae | Bacteroides | Bacteroides thetaiotaomicron |
| 10741 | 101_4950 | 32 | 8 | Bacteroidetes | Bacteroidia | Bacteroidales | Bacteroidaceae | Bacteroides | Bacteroides xylanisolvens |
| 10742 | 67_244 | 56 | 12 | Bacteroidetes | Bacteroidia | Bacteroidales | Bacteroidaceae | Bacteroides | Bacteroides xylanisolvens |
| 10743 | 78_9926 | 26 | 5 | Bacteroidetes | Bacteroidia | Bacteroidales | Bacteroidaceae | Bacteroides | Bacteroides xylanisolvens |
| 10750 | 91_1595 | 10 | 5 | Bacteroidetes | Bacteroidia | Bacteroidales | Bacteroidaceae | Bacteroides | Bacteroides ovatus |
| 10753 | 68_6163 | 58 | 9 | Bacteroidetes | Bacteroidia | Bacteroidales | Bacteroidaceae | Bacteroides | Bacteroides ovatus |
| 10757 | 32_7085 | 8 | 7 | Bacteroidetes | Bacteroidia | Bacteroidales | Bacteroidaceae | Bacteroides | Bacteroides ovatus |
| 10763 | 94_4882 | 68 | 1 | Bacteroidetes | Bacteroidia | Bacteroidales | Bacteroidaceae | Bacteroides | Bacteroides ovatus |
| 10769 | 130_5709 | 8 | 5 | Bacteroidetes | Bacteroidia | Bacteroidales | Bacteroidaceae | Bacteroides | Bacteroides caccae |
| 10770 | 120_1538 | 574 | 63 | Bacteroidetes | Bacteroidia | Bacteroidales | Bacteroidaceae | Bacteroides | Bacteroides caccae |
| 10771 | 16_1646 | 77 | 24 | Bacteroidetes | Bacteroidia | Bacteroidales | Bacteroidaceae | Bacteroides | Bacteroides caccae |
| 10781 | 105_3817 | 27 | 2 | Bacteroidetes | Bacteroidia | Bacteroidales | Bacteroidaceae | Bacteroides | Bacteroides finegoldii |
| 10787 | 9_4704 | 15 | 7 | Bacteroidetes | Bacteroidia | Bacteroidales | Bacteroidaceae | Bacteroides | Bacteroides faecis |
| 10791 | 105_7099 | 34 | 12 | Bacteroidetes | Bacteroidia | Bacteroidales | Bacteroidaceae | Bacteroides | Bacteroides massiliensis |
| 10808 | 107_4633 | 130 | 17 | Bacteroidetes | Bacteroidia | Bacteroidales | Bacteroidaceae | Bacteroides | Bacteroides massiliensis |
| 10819 | 94_4309 | 35 | 4 | Bacteroidetes | Bacteroidia | Bacteroidales | Bacteroidaceae | Bacteroides | Bacteroides ovatus |
| 10825 | 120_1671 | 39 | 10 | Bacteroidetes | Bacteroidia | Bacteroidales | Bacteroidaceae | Bacteroides | Bacteroides uniformis |
| 10836 | 110_5334 | 1460 | 27 | Bacteroidetes | Bacteroidia | Bacteroidales | Bacteroidaceae | Bacteroides | Bacteroides eggerthii |
| 10837 | 131_7799 | 18 | 5 | Bacteroidetes | Bacteroidia | Bacteroidales | Bacteroidaceae | Bacteroides | Bacteroides eggerthii |
| 10841 | 120_1159 | 19 | 6 | Bacteroidetes | Bacteroidia | Bacteroidales | Bacteroidaceae | Bacteroides | Bacteroides eggerthii |
| 10845 | 131_9460 | 34 | 4 | Bacteroidetes | Bacteroidia | Bacteroidales | Bacteroidaceae | Bacteroides | Bacteroides finegoldii |
| 10846 | 20_2270 | 241 | 3 | Bacteroidetes | Bacteroidia | Bacteroidales | Bacteroidaceae | Bacteroides | Bacteroides intestinalis |
| 10853 | 108_7187 | 361 | 9 | Bacteroidetes | Bacteroidia | Bacteroidales | Bacteroidaceae | Bacteroides | Bacteroides finegoldii |
| 10854 | 63_1225 | 333 | 25 | Bacteroidetes | Bacteroidia | Bacteroidales | Bacteroidaceae | Bacteroides | Bacteroides finegoldii |
| 10855 | 108_3125 | 20 | 3 | Bacteroidetes | Bacteroidia | Bacteroidales | Prevotellaceae | Prevotella | Prevotella copri |
| 10858 | 130_7068 | 12 | 7 | Bacteroidetes | Bacteroidia | Bacteroidales | Bacteroidaceae | Bacteroides | Bacteroides thetaiotaomicron |
| 10860 | 91_5076 | 26 | 6 | Bacteroidetes | Bacteroidia | Bacteroidales | Bacteroidaceae | Bacteroides | Bacteroides ovatus |
| 10863 | 68_1351 | 20 | 5 | Bacteroidetes | Bacteroidia | Bacteroidales | Bacteroidaceae | Bacteroides | Bacteroides ovatus |
| 10873 | 91_10141 | 42 | 7 | Bacteroidetes | Bacteroidia | Bacteroidales | Bacteroidaceae | Bacteroides | Bacteroides finegoldii |
| 10883 | 102_1708 | 12 | 8 | Bacteroidetes | Bacteroidia | Bacteroidales | Bacteroidaceae | Bacteroides | Bacteroides finegoldii |
| 10889 | 101_4694 | 14 | 6 | Bacteroidetes | Bacteroidia | Bacteroidales | Bacteroidaceae | Bacteroides | Bacteroides thetaiotaomicron |
| 10896 | 90_9941 | 25 | 10 | Bacteroidetes | Bacteroidia | Bacteroidales | Bacteroidaceae | Bacteroides | Bacteroides finegoldii |
| 10918 | 2_2598 | 133 | 24 | Bacteroidetes | Bacteroidia | Bacteroidales | Bacteroidaceae | Bacteroides | Bacteroides finegoldii |
| 10921 | 130_7131 | 9 | 5 | Bacteroidetes | Bacteroidia | Bacteroidales | Bacteroidaceae | Bacteroides | Bacteroides finegoldii |
| 10925 | 18_7523 | 17 | 10 | Bacteroidetes | Bacteroidia | Bacteroidales | Bacteroidaceae | Bacteroides | Bacteroides uniformis |
| 10927 | 50_3144 | 24 | 11 | Bacteroidetes | Bacteroidia | Bacteroidales | Bacteroidaceae | Bacteroides | Bacteroides thetaiotaomicron |
| 10943 | 105_3449 | 72 | 4 | Bacteroidetes | Bacteroidia | Bacteroidales | Bacteroidaceae | Bacteroides | Bacteroides sp. |
| 10944 | 79_8290 | 30 | 8 | Bacteroidetes | Bacteroidia | Bacteroidales | Bacteroidaceae | Bacteroides | Bacteroides finegoldii |
| 10945 | 37_3820 | 32 | 20 | Bacteroidetes | Bacteroidia | Bacteroidales | Bacteroidaceae | Bacteroides | Bacteroides sp. |
| 10956 | 100_8921 | 309 | 44 | Bacteroidetes | Bacteroidia | Bacteroidales | Bacteroidaceae | Bacteroides | Bacteroides sp. |
| 10958 | 68_6057 | 1912 | 45 | Bacteroidetes | Bacteroidia | Bacteroidales | Bacteroidaceae | Bacteroides | Bacteroides sp. |
| 10963 | 105_4609 | 12 | 7 | Bacteroidetes | Bacteroidia | Bacteroidales | Bacteroidaceae | Bacteroides | Bacteroides caccae |
| 10990 | 79_3101 | 11 | 8 | Bacteroidetes | Bacteroidia | Bacteroidales | Bacteroidaceae | Bacteroides | Bacteroides sp. |
| 10991 | 91_4347 | 15 | 5 | Bacteroidetes | Bacteroidia | Bacteroidales | Bacteroidaceae | Bacteroides | Bacteroides finegoldii |
| 10995 | 49_3943 | 94 | 22 | Bacteroidetes | Bacteroidia | Bacteroidales | Bacteroidaceae | Bacteroides | Bacteroides finegoldii |
| 10996 | 4_2141 | 31 | 16 | Bacteroidetes | Bacteroidia | Bacteroidales | Prevotellaceae | Prevotella | Prevotella copri |
| 11016 | 100_7397 | 549 | 28 | Bacteroidetes | Bacteroidia | Bacteroidales | Porphyromonadaceae | Parabacteroides | Parabacteroides distasonis |
| 11038 | 102_3108 | 27 | 6 | Bacteroidetes | Bacteroidia | Bacteroidales | Bacteroidaceae | Bacteroides | Bacteroides ovatus |
| 11052 | 14_2328 | 340 | 34 | Bacteroidetes | Bacteroidia | Bacteroidales | Bacteroidaceae | Bacteroides | Bacteroides ovatus |
| 11057 | 75_1697 | 65 | 17 | Bacteroidetes | Bacteroidia | Bacteroidales | Bacteroidaceae | Bacteroides | Bacteroides eggerthii |
| 11060 | 112_2827 | 35 | 18 | Bacteroidetes | Bacteroidia | Bacteroidales | Bacteroidaceae | Bacteroides | Bacteroides caccae |
| 11064 | 79_8182 | 87 | 12 | Bacteroidetes | Bacteroidia | Bacteroidales | Bacteroidaceae | Bacteroides | Bacteroides ovatus |
| 11066 | 91_624 | 10 | 7 | Bacteroidetes | Bacteroidia | Bacteroidales | Bacteroidaceae | Bacteroides | Bacteroides thetaiotaomicron |
| 11082 | 112_3930 | 88 | 25 | Bacteroidetes | Bacteroidia | Bacteroidales | Prevotellaceae | Prevotella | Prevotella copri |
| 11083 | 69_1581 | 83 | 19 | Bacteroidetes | Bacteroidia | Bacteroidales | Prevotellaceae | Prevotella | Prevotella copri |
| 11085 | 9_6203 | 61 | 3 | Bacteroidetes | Bacteroidia | Bacteroidales | Prevotellaceae | Prevotella | Prevotella copri |
| 11086 | 107_4436 | 86 | 26 | Bacteroidetes | Bacteroidia | Bacteroidales | Prevotellaceae | Prevotella | Prevotella copri |
| 11087 | 123_6518 | 36 | 9 | Bacteroidetes | Bacteroidia | Bacteroidales | Prevotellaceae | Prevotella | Prevotella copri |
| 11091 | 60_6187 | 47 | 7 | Bacteroidetes | Bacteroidia | Bacteroidales | Bacteroidaceae | Bacteroides | Bacteroides vulgatus |
| 11096 | 91_11066 | 41 | 11 | Bacteroidetes | Bacteroidia | Bacteroidales | Bacteroidaceae | Bacteroides | Bacteroides ovatus |
| 11103 | 120_2321 | 1917 | 73 | Bacteroidetes | Bacteroidia | Bacteroidales | Bacteroidaceae | Bacteroides | Bacteroides uniformis |
| 11104 | 123_7833 | 16 | 10 | Bacteroidetes | Bacteroidia | Bacteroidales | Prevotellaceae | Prevotella | Prevotella copri |
| 11115 | 30_4599 | 34 | 13 | Bacteroidetes | Bacteroidia | Bacteroidales | Bacteroidaceae | Bacteroides | Bacteroides uniformis |
| 11128 | 123_7622 | 11 | 6 | Bacteroidetes | Bacteroidia | Bacteroidales | Prevotellaceae | Prevotella | Prevotella copri |
| 11129 | 15_1599 | 15 | 5 | Bacteroidetes | Bacteroidia | Bacteroidales | Prevotellaceae | Prevotella | Prevotella copri |
| 11132 | 123_3659 | 47 | 13 | Bacteroidetes | Bacteroidia | Bacteroidales | Prevotellaceae | Prevotella | Prevotella copri |
| 11149 | 31_6722 | 23 | 5 | Bacteroidetes | Bacteroidia | Bacteroidales | Prevotellaceae | Prevotella | Prevotella denticola |
| 11151 | 54_1156 | 24 | 4 | Bacteroidetes | Bacteroidia | Bacteroidales | Prevotellaceae | Prevotella | Prevotella copri |
| 11159 | 81_1648 | 20 | 5 | Bacteroidetes | Bacteroidia | Bacteroidales | Prevotellaceae | Prevotella | Prevotella copri |
| 11165 | 81_3431 | 178 | 9 | Bacteroidetes | Bacteroidia | Bacteroidales | Prevotellaceae | Prevotella | Prevotella sp. |
| 11173 | 81_6791 | 285 | 7 | Bacteroidetes | Bacteroidia | Bacteroidales | Prevotellaceae | Prevotella | Prevotella sp. |
| 11174 | 62_3151 | 51 | 7 | Bacteroidetes | Bacteroidia | Bacteroidales | Prevotellaceae | Prevotella | Prevotella sp. |
| 11180 | 62_1371 | 372 | 8 | Bacteroidetes | Bacteroidia | Bacteroidales | Prevotellaceae | Prevotella | Prevotella sp. |
| 11189 | 1_1739 | 81 | 12 | Bacteroidetes | Bacteroidia | Bacteroidales | Prevotellaceae | Prevotella | Prevotella ruminicola |
| 11190 | 71_8902 | 33 | 8 | Bacteroidetes | Bacteroidia | Bacteroidales | Prevotellaceae | Prevotella | Prevotella copri |
| 11191 | 54_7187 | 60 | 18 | Bacteroidetes | Bacteroidia | Bacteroidales | Prevotellaceae | Prevotella | Prevotella copri |
| 11193 | 37_6178 | 18 | 9 | Bacteroidetes | Bacteroidia | Bacteroidales | Prevotellaceae | Prevotella | Prevotella ruminicola |
| 11202 | 49_4676 | 32 | 6 | Firmicutes | Negativicutes | Selenomonadales | Veillonellaceae | Dialister | Dialister invisus |
| 11208 | 129_2674 | 30 | 3 | Bacteroidetes | Bacteroidia | Bacteroidales | Prevotellaceae | Prevotella | Prevotella copri |
| 11213 | 108_3212 | 21 | 3 | Bacteroidetes | Bacteroidia | Bacteroidales | Prevotellaceae | Prevotella | Prevotella copri |
| 11224 | 23_4163 | 61 | 5 | Bacteroidetes | Bacteroidia | Bacteroidales | Prevotellaceae | Prevotella | Prevotella copri |
| 11226 | 43_4091 | 103 | 12 | Bacteroidetes | Bacteroidia | Bacteroidales | Prevotellaceae | Prevotella | Prevotella copri |
| 11228 | 21_2934 | 26 | 7 | Bacteroidetes | Bacteroidia | Bacteroidales | Prevotellaceae | Prevotella | Prevotella copri |
| 11229 | 54_7338 | 882 | 11 | Bacteroidetes | Bacteroidia | Bacteroidales | Prevotellaceae | Prevotella | Prevotella copri |
| 11232 | 121_5689 | 981 | 38 | Bacteroidetes | Bacteroidia | Bacteroidales | Prevotellaceae | Prevotella | Prevotella copri |
| 11234 | 54_7010 | 440 | 16 | Bacteroidetes | Bacteroidia | Bacteroidales | Prevotellaceae | Prevotella | Prevotella copri |
| 11235 | 125_6633 | 99 | 11 | Bacteroidetes | Bacteroidia | Bacteroidales | Prevotellaceae | Prevotella | Prevotella copri |
| 11240 | 104_1593 | 36 | 13 | Bacteroidetes | Bacteroidia | Bacteroidales | Prevotellaceae | Prevotella | Prevotella copri |
| 11247 | 112_1294 | 72 | 9 | Bacteroidetes | Bacteroidia | Bacteroidales | Prevotellaceae | Prevotella | Prevotella copri |
| 11248 | 15_5412 | 15 | 6 | Bacteroidetes | Bacteroidia | Bacteroidales | Bacteroidaceae | Bacteroides | Bacteroides ovatus |
| 11255 | 23_1999 | 9 | 5 | Bacteroidetes | Bacteroidia | Bacteroidales | Prevotellaceae | Prevotella | Prevotella copri |
| 11259 | 123_3988 | 6 | 5 | Bacteroidetes | Bacteroidia | Bacteroidales | Porphyromonadaceae | Barnesiella | Barnsiella intestinihominis |
| 11263 | 25_2740 | 112 | 13 | Bacteroidetes | Bacteroidia | Bacteroidales | Prevotellaceae | Prevotella | Prevotella copri |
| 11269 | 81_1650 | 157 | 9 | Bacteroidetes | Bacteroidia | Bacteroidales | Prevotellaceae | Prevotella | Prevotella copri |
| 11275 | 70_2232 | 24 | 6 | Bacteroidetes | Bacteroidia | Bacteroidales | Bacteroidaceae | Bacteroides | Bacteroides ovatus |
| 11279 | 81_3241 | 408 | 13 | Bacteroidetes | Bacteroidia | Bacteroidales | Prevotellaceae | Prevotella | Prevotella copri |
| 11283 | 81_3019 | 24 | 6 | Bacteroidetes | Bacteroidia | Bacteroidales | Prevotellaceae | Prevotella | Prevotella copri |
| 11285 | 81_3149 | 485 | 18 | Bacteroidetes | Bacteroidia | Bacteroidales | Prevotellaceae | Prevotella | Prevotella copri |
| 11287 | 112_921 | 37 | 7 | Bacteroidetes | Bacteroidia | Bacteroidales | Prevotellaceae | Prevotella | Prevotella copri |
| 11291 | 104_770 | 43 | 5 | Bacteroidetes | Bacteroidia | Bacteroidales | Prevotellaceae | Prevotella | Prevotella copri |
| 11295 | 21_93 | 11 | 6 | Bacteroidetes | Bacteroidia | Bacteroidales | Prevotellaceae | Prevotella | Prevotella copri |
| 11299 | 123_7713 | 74 | 12 | Bacteroidetes | Bacteroidia | Bacteroidales | Prevotellaceae | Prevotella | Prevotella copri |
| 11305 | 121_6709 | 114 | 10 | Bacteroidetes | Bacteroidia | Bacteroidales | Prevotellaceae | Prevotella | Prevotella copri |
| 11306 | 112_2284 | 181 | 9 | Bacteroidetes | Bacteroidia | Bacteroidales | Prevotellaceae | Prevotella | Prevotella copri |
| 11313 | 1_3221 | 93 | 9 | Bacteroidetes | Bacteroidia | Bacteroidales | Prevotellaceae | Prevotella | Prevotella copri |
| 11316 | 112_1582 | 22 | 5 | Bacteroidetes | Bacteroidia | Bacteroidales | Prevotellaceae | Prevotella | Prevotella copri |
| 11318 | 123_3963 | 294 | 7 | Bacteroidetes | Bacteroidia | Bacteroidales | Prevotellaceae | Prevotella | Prevotella copri |
| 11324 | 8_2765 | 31 | 10 | Bacteroidetes | Bacteroidia | Bacteroidales | Prevotellaceae | Prevotella | Prevotella copri |
| 11325 | 81_2925 | 27 | 7 | Bacteroidetes | Bacteroidia | Bacteroidales | Prevotellaceae | Prevotella | Prevotella copri |
| 11327 | 121_2909 | 91 | 7 | Bacteroidetes | Bacteroidia | Bacteroidales | Prevotellaceae | Prevotella | Prevotella copri |
| 11331 | 104_1451 | 32 | 6 | Bacteroidetes | Bacteroidia | Bacteroidales | Prevotellaceae | Prevotella | Prevotella copri |
| 11335 | 121_6546 | 11 | 6 | Bacteroidetes | Bacteroidia | Bacteroidales | Prevotellaceae | Prevotella | Prevotella copri |
| 11340 | 48_5017 | 219 | 8 | Bacteroidetes | Bacteroidia | Bacteroidales | Prevotellaceae | Prevotella | Prevotella copri |
| 11345 | 48_3129 | 37 | 6 | Bacteroidetes | Bacteroidia | Bacteroidales | Prevotellaceae | Prevotella | Prevotella copri |
| 11346 | 48_6340 | 66 | 4 | Bacteroidetes | Bacteroidia | Bacteroidales | Prevotellaceae | Prevotella | Prevotella copri |
| 11348 | 48_2162 | 102 | 4 | Bacteroidetes | Bacteroidia | Bacteroidales | Prevotellaceae | Prevotella | Prevotella copri |
| 11349 | 112_3022 | 55 | 9 | Bacteroidetes | Bacteroidia | Bacteroidales | Prevotellaceae | Prevotella | Prevotella copri |
| 11352 | 121_5484 | 20 | 4 | Bacteroidetes | Bacteroidia | Bacteroidales | Prevotellaceae | Prevotella | Prevotella ruminicola |
| 11362 | 89_4300 | 85 | 7 | Bacteroidetes | Bacteroidia | Bacteroidales | Prevotellaceae | Paraprevotella | Paraprevotella clara |
| 11380 | 107_1740 | 25 | 6 | Bacteroidetes | Bacteroidia | Bacteroidales | Prevotellaceae | Prevotella | Prevotella copri |
| 11390 | 62_2875 | 8 | 5 | Bacteroidetes | Bacteroidia | Bacteroidales | Prevotellaceae | Paraprevotella | Paraprevotella clara |
| 11394 | 5_654 | 13 | 8 | Firmicutes | Clostridia | Clostridiales | Peptococcaceae | Peptococcus | Peptococcus sp. |
| 11480 | 67_3476 | 13 | 7 | Bacteroidetes | Bacteroidia | Bacteroidales | Porphyromonadaceae | Barnesiella | Barnesiella intestinihominis |
| 11498 | 99_2901 | 210 | 25 | Bacteroidetes | Bacteroidia | Bacteroidales | Bacteroidaceae | Bacteroides | Bacteroides uniformis |
| 11515 | 45_1338 | 6 | 6 | Firmicutes | Clostridia | Clostridiales | Lachnospiraceae | Lachnospiraceae incertae sedis | Lachnospiraceae incertae sedis |
| 11526 | 66_3813 | 86 | 11 | Firmicutes | Clostridia | Clostridiales | Lactobacillaceae | Lactobacillus | Lactobacillus ruminis |
| 11617 | 130_7074 | 6 | 5 | Firmicutes | Negativicutes | Selenomonadales | Veillonellaceae | Dialister | Dialister invisus |
| 11619 | 63_1663 | 11 | 6 | Bacteroidetes | Bacteroidia | Bacteroidales | Rikenellaceae | Alistipes | Alistipes sp. |
| 11629 | 106_4315 | 17 | 7 | Firmicutes | Negativicutes | Selenomonadales | Acidaminococcaceae | Acidaminococcus | Acidaminococcus intestini |
| 11755 | 55_5647 | 6 | 5 | Bacteroidetes | Bacteroidia | Bacteroidales | Bacteroidaceae | Bacteroides | Bacteroides uniformis |
| 11790 | 37_3713 | 6 | 5 | Firmicutes | Negativicutes | Selenomonadales | Veillonellaceae | Dialister | Dialister invisus |
| 11801 | 121_6023 | 13 | 6 | Firmicutes | Negativicutes | Selenomonadales | Veillonellaceae | Mitsuokella | Mitsuokella |
| 11845 | 48_6408 | 9 | 6 | Firmicutes | Negativicutes | Selenomonadales | Veillonellaceae | Dialister | Dialister invisus |
| 11865 | 17_2495 | 1169 | 70 | Firmicutes | Clostridia | Clostridiales | Ruminococcaceae | Faecalibacterium | Faecalibacterium prausnitzii |
| 11868 | 55_4339 | 48 | 18 | Firmicutes | Clostridia | Clostridiales | Ruminococcaceae | Faecalibacterium | Faecalibacterium prausnitzii |
| 11919 | 16_1718 | 465 | 61 | Actinobacteria | Coriobacteriia | Coriobacteriales | Coriobacteriaceae | Collinsella | Collinsella aerofaciens |
| 11920 | 16_5696 | 8 | 6 | Actinobacteria | Coriobacteriia | Coriobacteriales | Coriobacteriaceae | Collinsella | Collinsella aerofaciens |
| 11929 | 2_5799 | 7 | 6 | Firmicutes | Clostridia | Clostridiales | Lachnospiraceae | Blautia | Blautia |
| 11971 | 130_321 | 8 | 5 | Firmicutes | Negativicutes | Selenomonadales | Veillonellaceae | Dialister | Dialister invisus |
| 12018 | 47_11916 | 13 | 5 | Firmicutes | Clostridia | Clostridiales | Peptococcaceae | Peptococcus | Peptococcus sp. |
| 12053 | 114_7375 | 37 | 13 | Bacteroidetes | Bacteroidia | Bacteroidales | Prevotellaceae | Prevotella | Prevotella copri |
| 12072 | 124_3277 | 8 | 8 | Bacteroidetes | Bacteroidia | Bacteroidales | Porphyromonadaceae | Parabacteroides | Parabacteroides merdae |
| 12075 | 115_2108 | 9 | 7 | Bacteroidetes | Bacteroidia | Bacteroidales | Prevotellaceae | Prevotella | Prevotella copri |
| 12080 | 115_1081 | 11 | 8 | Bacteroidetes | Bacteroidia | Bacteroidales | Porphyromonadaceae | Parabacteroides | Parabacteroides merdae |
| 12085 | 4_3989 | 11 | 8 | Bacteroidetes | Bacteroidia | Bacteroidales | Porphyromonadaceae | Parabacteroides | Parabacteroides merdae |
| 12099 | 121_3149 | 13 | 8 | Bacteroidetes | Bacteroidia | Bacteroidales | Porphyromonadaceae | Parabacteroides | Parabacteroides merdae |
| 12107 | 80_4988 | 13 | 8 | Bacteroidetes | Bacteroidia | Bacteroidales | Prevotellaceae | Prevotella | Prevotella copri |
| 12108 | 70_4660 | 10 | 5 | Bacteroidetes | Bacteroidia | Bacteroidales | Bacteroidaceae | Bacteroides | Bacteroides thetaiotaomicron |
| 12109 | 10_6462 | 22 | 11 | Bacteroidetes | Bacteroidia | Bacteroidales | Bacteroidaceae | Bacteroides | Bacteroides uniformis |
| 12110 | 43_1664 | 18 | 7 | Bacteroidetes | Bacteroidia | Bacteroidales | Prevotellaceae | Prevotella | Prevotella copri |
| 12112 | 27_2021 | 41 | 24 | Bacteroidetes | Bacteroidia | Bacteroidales | Porphyromonadaceae | Parabacteroides | Parabacteroides merdae |
| 12122 | 58_7268 | 41 | 4 | Bacteroidetes | Bacteroidia | Bacteroidales | Bacteroidaceae | Bacteroides | Bacteroides ovatus |
| 12125 | 58_8045 | 28 | 2 | Bacteroidetes | Bacteroidia | Bacteroidales | Bacteroidaceae | Bacteroides | Bacteroides ovatus |
| 12128 | 58_871 | 538 | 7 | Bacteroidetes | Bacteroidia | Bacteroidales | Bacteroidaceae | Bacteroides | Bacteroides plebeius |
| 12146 | 91_5307 | 13 | 7 | Bacteroidetes | Bacteroidia | Bacteroidales | Bacteroidaceae | Bacteroides | Bacteroides cellulosilyticus |
| 12153 | 88_4731 | 17 | 10 | Bacteroidetes | Bacteroidia | Bacteroidales | Bacteroidaceae | Bacteroides | Bacteroides uniformis |
| 12185 | 100_5251 | 38 | 5 | Bacteroidetes | Bacteroidia | Bacteroidales | Bacteroidaceae | Bacteroides | Bacteroides ovatus |
| 12202 | 131_9651 | 35 | 13 | Bacteroidetes | Bacteroidia | Bacteroidales | Bacteroidaceae | Bacteroides | Bacteroides ovatus |
| 12204 | 131_9588 | 73 | 13 | Bacteroidetes | Bacteroidia | Bacteroidales | Bacteroidaceae | Bacteroides | Bacteroides uniformis |
| 12206 | 77_6974 | 21 | 3 | Bacteroidetes | Bacteroidia | Bacteroidales | Prevotellaceae | Paraprevotella | Paraprevotella clara |
| 12207 | 77_7951 | 42 | 4 | Bacteroidetes | Bacteroidia | Bacteroidales | Prevotellaceae | Paraprevotella | Paraprevotella clara |
| 12231 | 91_10738 | 41 | 5 | Bacteroidetes | Bacteroidia | Bacteroidales | Bacteroidaceae | Bacteroides | Bacteroides ovatus |
| 12232 | 68_5318 | 52 | 4 | Bacteroidetes | Bacteroidia | Bacteroidales | Bacteroidaceae | Bacteroides | Bacteroides ovatus |
| 12234 | 91_5632 | 45 | 5 | Bacteroidetes | Bacteroidia | Bacteroidales | Bacteroidaceae | Bacteroides | Bacteroides ovatus |
| 12241 | 91_10406 | 23 | 4 | Bacteroidetes | Bacteroidia | Bacteroidales | Bacteroidaceae | Bacteroides | Bacteroides ovatus |
| 12242 | 79_8175 | 52 | 6 | Bacteroidetes | Bacteroidia | Bacteroidales | Bacteroidaceae | Bacteroides | Bacteroides thetaiotaomicron |
| 12243 | 102_5945 | 136 | 5 | Bacteroidetes | Bacteroidia | Bacteroidales | Bacteroidaceae | Bacteroides | Bacteroides thetaiotaomicron |
| 12248 | 56_2292 | 33 | 5 | Bacteroidetes | Bacteroidia | Bacteroidales | Bacteroidaceae | Bacteroides | Bacteroides ovatus |
| 12249 | 91_5140 | 72 | 9 | Bacteroidetes | Bacteroidia | Bacteroidales | Bacteroidaceae | Bacteroides | Bacteroides ovatus |
| 12253 | 100_11761 | 100 | 8 | Bacteroidetes | Bacteroidia | Bacteroidales | Bacteroidaceae | Bacteroides | Bacteroides sp. |
| 12260 | 68_1819 | 7 | 5 | Bacteroidetes | Bacteroidia | Bacteroidales | Bacteroidaceae | Bacteroides | Bacteroides ovatus |
| 12261 | 100_7261 | 16 | 5 | Bacteroidetes | Bacteroidia | Bacteroidales | Bacteroidaceae | Bacteroides | Bacteroides ovatus |
| 12262 | 100_4536 | 228 | 18 | Bacteroidetes | Bacteroidia | Bacteroidales | Bacteroidaceae | Bacteroides | Bacteroides ovatus |
| 12265 | 68_10323 | 20 | 6 | Bacteroidetes | Bacteroidia | Bacteroidales | Bacteroidaceae | Bacteroides | Bacteroides ovatus |
| 12267 | 89_7062 | 148 | 11 | Bacteroidetes | Bacteroidia | Bacteroidales | Bacteroidaceae | Bacteroides | Bacteroides ovatus |
| 12273 | 91_5690 | 14 | 6 | Bacteroidetes | Bacteroidia | Bacteroidales | Bacteroidaceae | Bacteroides | Bacteroides ovatus |
| 12274 | 131_4436 | 27 | 7 | Bacteroidetes | Bacteroidia | Bacteroidales | Bacteroidaceae | Bacteroides | Bacteroides ovatus |
| 12291 | 121_6505 | 6 | 6 | Bacteroidetes | Bacteroidia | Bacteroidales | Prevotellaceae | Prevotella | Prevotella copri |
| 12293 | 105_3810 | 30 | 6 | Bacteroidetes | Bacteroidia | Bacteroidales | Bacteroidaceae | Bacteroides | Bacteroides caccae |
| 12299 | 94_3394 | 41 | 9 | Bacteroidetes | Bacteroidia | Bacteroidales | Bacteroidaceae | Bacteroides | Bacteroides caccae |
| 12318 | 94_9558 | 20 | 2 | Bacteroidetes | Bacteroidia | Bacteroidales | Bacteroidaceae | Bacteroides | Bacteroides ovatus |
| 12335 | 68_11008 | 6 | 5 | Bacteroidetes | Bacteroidia | Bacteroidales | Bacteroidaceae | Bacteroides | Bacteroides ovatus |
| 12340 | 123_6702 | 7 | 6 | Bacteroidetes | Bacteroidia | Bacteroidales | Bacteroidaceae | Bacteroides | Bacteroides ovatus |
| 12355 | 81_433 | 23 | 6 | Bacteroidetes | Bacteroidia | Bacteroidales | Prevotellaceae | Prevotella | Prevotella copri |
| 12358 | 79_8256 | 30 | 7 | Bacteroidetes | Bacteroidia | Bacteroidales | Bacteroidaceae | Bacteroides | Bacteroides ovatus |
| 12361 | 101_6384 | 17 | 6 | Bacteroidetes | Bacteroidia | Bacteroidales | Bacteroidaceae | Bacteroides | Bacteroides thetaiotaomicron |
| 12362 | 41_4603 | 20 | 6 | Bacteroidetes | Bacteroidia | Bacteroidales | Bacteroidaceae | Bacteroides | Bacteroides thetaiotaomicron |
| 12365 | 68_10857 | 8 | 6 | Bacteroidetes | Bacteroidia | Bacteroidales | Bacteroidaceae | Bacteroides | Bacteroides ovatus |
| 12366 | 79_4453 | 18 | 6 | Bacteroidetes | Bacteroidia | Bacteroidales | Bacteroidaceae | Bacteroides | Bacteroides thetaiotaomicron |
| 12369 | 131_2736 | 218 | 37 | Bacteroidetes | Bacteroidia | Bacteroidales | Bacteroidaceae | Bacteroides | Bacteroides uniformis |
| 12372 | 91_9420 | 28 | 7 | Bacteroidetes | Bacteroidia | Bacteroidales | Bacteroidaceae | Bacteroides | Bacteroides ovatus |
| 12387 | 102_4297 | 6 | 5 | Bacteroidetes | Bacteroidia | Bacteroidales | Bacteroidaceae | Bacteroides | Bacteroides thetaiotaomicron |
| 12397 | 89_9878 | 22 | 5 | Bacteroidetes | Bacteroidia | Bacteroidales | Bacteroidaceae | Bacteroides | Bacteroides ovatus |
| 12403 | 77_3840 | 45 | 3 | Bacteroidetes | Bacteroidia | Bacteroidales | Prevotellaceae | Paraprevotella | Paraprevotella clara |
| 12411 | 15_6213 | 10 | 6 | Bacteroidetes | Bacteroidia | Bacteroidales | Bacteroidaceae | Bacteroides | Bacteroides ovatus |
| 12412 | 91_1450 | 30 | 10 | Bacteroidetes | Bacteroidia | Bacteroidales | Bacteroidaceae | Bacteroides | Bacteroides ovatus |
| 12413 | 91_9516 | 45 | 6 | Bacteroidetes | Bacteroidia | Bacteroidales | Bacteroidaceae | Bacteroides | Bacteroides ovatus |
| 12414 | 122_1298 | 15 | 11 | Bacteroidetes | Bacteroidia | Bacteroidales | Bacteroidaceae | Bacteroides | Bacteroides caccae |
| 12422 | 91_3620 | 14 | 5 | Bacteroidetes | Bacteroidia | Bacteroidales | Bacteroidaceae | Bacteroides | Bacteroides ovatus |
| 12432 | 94_10638 | 24 | 2 | Bacteroidetes | Bacteroidia | Bacteroidales | Bacteroidaceae | Bacteroides | Bacteroides ovatus |
| 12437 | 94_4659 | 58 | 3 | Bacteroidetes | Bacteroidia | Bacteroidales | Bacteroidaceae | Bacteroides | Bacteroides ovatus |
| 12441 | 105_3833 | 21 | 6 | Bacteroidetes | Bacteroidia | Bacteroidales | Bacteroidaceae | Bacteroides | Bacteroides faecis |
| 12442 | 105_6923 | 863 | 21 | Bacteroidetes | Bacteroidia | Bacteroidales | Bacteroidaceae | Bacteroides | Bacteroides thetaiotaomicron |
| 12446 | 16_4393 | 255 | 52 | Bacteroidetes | Bacteroidia | Bacteroidales | Bacteroidaceae | Bacteroides | Bacteroides caccae |
| 12458 | 76_1554 | 120 | 7 | Bacteroidetes | Bacteroidia | Bacteroidales | Bacteroidaceae | Bacteroides | Bacteroides fragilis |
| 12481 | 78_9809 | 64 | 12 | Bacteroidetes | Bacteroidia | Bacteroidales | Bacteroidaceae | Bacteroides | Bacteroides uniformis |
| 12482 | 107_3919 | 22 | 11 | Bacteroidetes | Bacteroidia | Bacteroidales | Bacteroidaceae | Bacteroides | Bacteroides massiliensis |
| 12484 | 71_4616 | 133 | 18 | Bacteroidetes | Bacteroidia | Bacteroidales | Bacteroidaceae | Bacteroides | Bacteroides massiliensis |
| 12504 | 94_5002 | 34 | 11 | Bacteroidetes | Bacteroidia | Bacteroidales | Bacteroidaceae | Bacteroides | Bacteroides caccae |
| 12505 | 126_10 | 11 | 5 | Bacteroidetes | Bacteroidia | Bacteroidales | Bacteroidaceae | Bacteroides | Bacteroides sp. |
| 12511 | 4_362 | 39 | 12 | Bacteroidetes | Bacteroidia | Bacteroidales | Prevotellaceae | Prevotella | Prevotella copri |
| 12514 | 94_3532 | 20 | 4 | Bacteroidetes | Bacteroidia | Bacteroidales | Bacteroidaceae | Bacteroides | Bacteroides ovatus |
| 12518 | 110_4793 | 37 | 5 | Bacteroidetes | Bacteroidia | Bacteroidales | Bacteroidaceae | Bacteroides | Bacteroides eggerthii |
| 12525 | 110_5346 | 11 | 5 | Bacteroidetes | Bacteroidia | Bacteroidales | Bacteroidaceae | Bacteroides | Bacteroides eggerthii |
| 12526 | 110_1334 | 9 | 6 | Bacteroidetes | Bacteroidia | Bacteroidales | Bacteroidaceae | Bacteroides | Bacteroides eggerthii |
| 12530 | 110_882 | 60 | 6 | Bacteroidetes | Bacteroidia | Bacteroidales | Bacteroidaceae | Bacteroides | Bacteroides eggerthii |
| 12531 | 131_3088 | 24 | 7 | Bacteroidetes | Bacteroidia | Bacteroidales | Bacteroidaceae | Bacteroides | Bacteroides eggerthii |
| 12532 | 110_4787 | 87 | 9 | Bacteroidetes | Bacteroidia | Bacteroidales | Bacteroidaceae | Bacteroides | Bacteroides eggerthii |
| 12535 | 110_2490 | 39 | 7 | Bacteroidetes | Bacteroidia | Bacteroidales | Bacteroidaceae | Bacteroides | Bacteroides eggerthii |
| 12536 | 63_2527 | 86 | 13 | Bacteroidetes | Bacteroidia | Bacteroidales | Bacteroidaceae | Bacteroides | Bacteroides uniformis |
| 12537 | 63_3187 | 83 | 11 | Bacteroidetes | Bacteroidia | Bacteroidales | Bacteroidaceae | Bacteroides | Bacteroides eggerthii |
| 12547 | 131_9391 | 80 | 12 | Bacteroidetes | Bacteroidia | Bacteroidales | Bacteroidaceae | Bacteroides | Bacteroides eggerthii |
| 12549 | 75_2875 | 27 | 8 | Bacteroidetes | Bacteroidia | Bacteroidales | Bacteroidaceae | Bacteroides | Bacteroides caccae |
| 12553 | 131_7663 | 25 | 5 | Bacteroidetes | Bacteroidia | Bacteroidales | Bacteroidaceae | Bacteroides | Bacteroides xylanisolvens |
| 12560 | 63_5696 | 41 | 10 | Bacteroidetes | Bacteroidia | Bacteroidales | Bacteroidaceae | Bacteroides | Bacteroides finegoldii |
| 12578 | 24_2330 | 21 | 15 | Bacteroidetes | Bacteroidia | Bacteroidales | Bacteroidaceae | Bacteroides | Bacteroides uniformis |
| 12580 | 131_8721 | 85 | 13 | Bacteroidetes | Bacteroidia | Bacteroidales | Bacteroidaceae | Bacteroides | Bacteroides uniformis |
| 12582 | 79_6913 | 72 | 14 | Bacteroidetes | Bacteroidia | Bacteroidales | Bacteroidaceae | Bacteroides | Bacteroides ovatus |
| 12605 | 94_9790 | 73 | 19 | Bacteroidetes | Bacteroidia | Bacteroidales | Bacteroidaceae | Bacteroides | Bacteroides ovatus |
| 12615 | 60_6580 | 25 | 12 | Bacteroidetes | Bacteroidia | Bacteroidales | Bacteroidaceae | Bacteroides | Bacteroides sp. |
| 12617 | 49_2244 | 26 | 11 | Bacteroidetes | Bacteroidia | Bacteroidales | Bacteroidaceae | Bacteroides | Bacteroides uniformis |
| 12661 | 130_3680 | 18 | 7 | Bacteroidetes | Bacteroidia | Bacteroidales | Bacteroidaceae | Bacteroides | Bacteroides caccae |
| 12668 | 114_1034 | 323 | 14 | Bacteroidetes | Bacteroidia | Bacteroidales | Bacteroidaceae | Bacteroides | Bacteroides uniformis |
| 12672 | 111_600 | 9 | 5 | Bacteroidetes | Bacteroidia | Bacteroidales | Bacteroidaceae | Bacteroides | Bacteroides uniformis |
| 12685 | 60_6205 | 263 | 15 | Bacteroidetes | Bacteroidia | Bacteroidales | Bacteroidaceae | Bacteroides | Bacteroides sp. |
| 12691 | 60_6892 | 122 | 15 | Bacteroidetes | Bacteroidia | Bacteroidales | Bacteroidaceae | Bacteroides | Bacteroides sp. |
| 12692 | 56_11088 | 17 | 7 | Bacteroidetes | Bacteroidia | Bacteroidales | Bacteroidaceae | Bacteroides | Bacteroides finegoldii |
| 12697 | 68_11888 | 54 | 9 | Bacteroidetes | Bacteroidia | Bacteroidales | Bacteroidaceae | Bacteroides | Bacteroides ovatus |
| 12706 | 105_3824 | 27 | 12 | Bacteroidetes | Bacteroidia | Bacteroidales | Bacteroidaceae | Bacteroides | Bacteroides caccae |
| 12711 | 116_970 | 92 | 12 | Bacteroidetes | Bacteroidia | Bacteroidales | Bacteroidaceae | Bacteroides | Bacteroides finegoldii |
| 12717 | 102_4152 | 18 | 5 | Bacteroidetes | Bacteroidia | Bacteroidales | Bacteroidaceae | Bacteroides | Bacteroides sp. |
| 12732 | 117_2375 | 27 | 12 | Bacteroidetes | Bacteroidia | Bacteroidales | Bacteroidaceae | Bacteroides | Bacteroides finegoldii |
| 12737 | 131_9413 | 184 | 15 | Bacteroidetes | Bacteroidia | Bacteroidales | Bacteroidaceae | Bacteroides | Bacteroides eggerthii |
| 12738 | 131_9205 | 60 | 15 | Bacteroidetes | Bacteroidia | Bacteroidales | Bacteroidaceae | Bacteroides | Bacteroides uniformis |
| 12745 | 40_4919 | 39 | 12 | Bacteroidetes | Bacteroidia | Bacteroidales | Bacteroidaceae | Bacteroides | Bacteroides caccae |
| 12758 | 75_1761 | 22 | 6 | Bacteroidetes | Bacteroidia | Bacteroidales | Bacteroidaceae | Bacteroides | Bacteroides eggerthii |
| 12765 | 91_5252 | 76 | 18 | Bacteroidetes | Bacteroidia | Bacteroidales | Bacteroidaceae | Bacteroides | Bacteroides sp. |
| 12767 | 68_11171 | 151 | 11 | Bacteroidetes | Bacteroidia | Bacteroidales | Bacteroidaceae | Bacteroides | Bacteroides sp. |
| 12768 | 79_4417 | 52 | 6 | Bacteroidetes | Bacteroidia | Bacteroidales | Bacteroidaceae | Bacteroides | Bacteroides finegoldii |
| 12770 | 79_3869 | 21 | 5 | Bacteroidetes | Bacteroidia | Bacteroidales | Bacteroidaceae | Bacteroides | Bacteroides finegoldii |
| 12773 | 105_7001 | 16 | 7 | Bacteroidetes | Bacteroidia | Bacteroidales | Bacteroidaceae | Bacteroides | Bacteroides caccae |
| 12787 | 69_4508 | 15 | 8 | Bacteroidetes | Bacteroidia | Bacteroidales | Porphyromonadaceae | Parabacteroides | Parabacteroides merdae |
| 12791 | 17_466 | 137 | 17 | Bacteroidetes | Bacteroidia | Bacteroidales | Porphyromonadaceae | Parabacteroides | Parabacteroides distasonis |
| 12792 | 17_1973 | 19 | 13 | Bacteroidetes | Bacteroidia | Bacteroidales | Bacteroidaceae | Bacteroides | Bacteroides massiliensis |
| 12796 | 100_8391 | 66 | 7 | Bacteroidetes | Bacteroidia | Bacteroidales | Bacteroidaceae | Bacteroides | Bacteroides ovatus |
| 12802 | 100_3670 | 23 | 4 | Bacteroidetes | Bacteroidia | Bacteroidales | Porphyromonadaceae | Parabacteroides | Parabacteroides distasonis |
| 12803 | 114_7774 | 11 | 6 | Bacteroidetes | Bacteroidia | Bacteroidales | Porphyromonadaceae | Barnesiella | Barnesiella intestinihominis |
| 12807 | 23_689 | 22 | 10 | Bacteroidetes | Bacteroidia | Bacteroidales | Prevotellaceae | Prevotella | Prevotella copri |
| 12811 | 15_1978 | 5 | 5 | Bacteroidetes | Bacteroidia | Bacteroidales | Porphyromonadaceae | Barnesiella | Barnesiella intestinihominis |
| 12813 | 75_1467 | 6 | 5 | Bacteroidetes | Bacteroidia | Bacteroidales | Bacteroidaceae | Bacteroides | Bacteroides uniformis |
| 12817 | 50_1813 | 19 | 10 | Bacteroidetes | Bacteroidia | Bacteroidales | Porphyromonadaceae | Parabacteroides | Parabacteroides merdae |
| 12830 | 70_1524 | 8 | 6 | Bacteroidetes | Bacteroidia | Bacteroidales | Bacteroidaceae | Bacteroides | Bacteroides ovatus |
| 12835 | 23_2457 | 37 | 11 | Bacteroidetes | Bacteroidia | Bacteroidales | Prevotellaceae | Prevotella | Prevotella copri |
| 12838 | 20_7015 | 90 | 16 | Bacteroidetes | Bacteroidia | Bacteroidales | Bacteroidaceae | Bacteroides | Bacteroides eggerthii |
| 12860 | 32_5121 | 51 | 18 | Bacteroidetes | Bacteroidia | Bacteroidales | Bacteroidaceae | Bacteroides | Bacteroides ovatus |
| 12867 | 41_8786 | 9 | 7 | Bacteroidetes | Bacteroidia | Bacteroidales | Bacteroidaceae | Bacteroides | Bacteroides xylanisolvens |
| 12869 | 114_7997 | 8 | 6 | Bacteroidetes | Bacteroidia | Bacteroidales | Porphyromonadaceae | Barnesiella | Barnsiella intestinihominis |
| 12872 | 4_1177 | 12 | 6 | Bacteroidetes | Bacteroidia | Bacteroidales | Prevotellaceae | Prevotella | Prevotella copri |
| 12881 | 56_9923 | 69 | 10 | Bacteroidetes | Bacteroidia | Bacteroidales | Bacteroidaceae | Bacteroides | Bacteroides ovatus |
| 12882 | 68_1519 | 9 | 5 | Bacteroidetes | Bacteroidia | Bacteroidales | Bacteroidaceae | Bacteroides | Bacteroides ovatus |
| 12883 | 68_11367 | 28 | 8 | Bacteroidetes | Bacteroidia | Bacteroidales | Bacteroidaceae | Bacteroides | Bacteroides ovatus |
| 12886 | 91_4754 | 26 | 8 | Bacteroidetes | Bacteroidia | Bacteroidales | Bacteroidaceae | Bacteroides | Bacteroides ovatus |
| 12903 | 80_2251 | 9 | 6 | Bacteroidetes | Bacteroidia | Bacteroidales | Bacteroidaceae | Bacteroides | Bacteroides caccae |
| 12904 | 30_7341 | 951 | 13 | Bacteroidetes | Bacteroidia | Bacteroidales | Bacteroidaceae | Bacteroides | Bacteroides vulgatus |
| 12906 | 56_5475 | 24 | 9 | Bacteroidetes | Bacteroidia | Bacteroidales | Bacteroidaceae | Bacteroides | Bacteroides ovatus |
| 12910 | 4_3925 | 62 | 19 | Bacteroidetes | Bacteroidia | Bacteroidales | Prevotellaceae | Prevotella | Prevotella copri |
| 12912 | 121_6606 | 32 | 12 | Bacteroidetes | Bacteroidia | Bacteroidales | Prevotellaceae | Prevotella | Prevotella copri |
| 12915 | 45_2019 | 28 | 9 | Bacteroidetes | Bacteroidia | Bacteroidales | Prevotellaceae | Prevotella | Prevotella copri |
| 12919 | 7_4728 | 22 | 13 | Bacteroidetes | Bacteroidia | Bacteroidales | Porphyromonadaceae | Parabacteroides | Parabacteroides merdae |
| 12953 | 123_4824 | 14 | 9 | Bacteroidetes | Bacteroidia | Bacteroidales | Prevotellaceae | Prevotella | Prevotella copri |
| 12954 | 114_5158 | 6 | 5 | Bacteroidetes | Bacteroidia | Bacteroidales | Bacteroidaceae | Bacteroides | Bacteroides ovatus |
| 12955 | 56_5792 | 35 | 8 | Bacteroidetes | Bacteroidia | Bacteroidales | Bacteroidaceae | Bacteroides | Bacteroides ovatus |
| 12967 | 119_6291 | 8 | 5 | Bacteroidetes | Bacteroidia | Bacteroidales | Bacteroidaceae | Bacteroides | Bacteroides thetaiotaomicron |
| 12971 | 1_3087 | 36 | 24 | Bacteroidetes | Bacteroidia | Bacteroidales | Porphyromonadaceae | Parabacteroides | Parabacteroides merdae |
| 12976 | 54_2319 | 8 | 5 | Bacteroidetes | Bacteroidia | Bacteroidales | Prevotellaceae | Prevotella | Prevotella copri |
| 12984 | 61_5388 | 9 | 7 | Bacteroidetes | Bacteroidia | Bacteroidales | Rikenellaceae | Alistipes | Alistipes indistinctus |
| 12986 | 84_3506 | 12 | 8 | Bacteroidetes | Bacteroidia | Bacteroidales | Rikenellaceae | Alistipes | Alistipes putredinis |
| 12987 | 115_4207 | 236 | 36 | Bacteroidetes | Bacteroidia | Bacteroidales | Rikenellaceae | Alistipes | Alistipes onderdonkii |
| 12991 | 54_4791 | 10 | 6 | Bacteroidetes | Bacteroidia | Bacteroidales | Prevotellaceae | Prevotella | Prevotella copri |
| 12994 | 21_3619 | 28 | 4 | Bacteroidetes | Bacteroidia | Bacteroidales | Prevotellaceae | Prevotella | Prevotella copri |
| 12995 | 43_3718 | 13 | 6 | Bacteroidetes | Bacteroidia | Bacteroidales | Prevotellaceae | Prevotella | Prevotella copri |
| 13001 | 1_3317 | 39 | 6 | Bacteroidetes | Bacteroidia | Bacteroidales | Prevotellaceae | Prevotella | Prevotella copri |
| 13018 | 31_5432 | 21 | 5 | Bacteroidetes | Bacteroidia | Bacteroidales | Prevotellaceae | Prevotella | Prevotella copri |
| 13032 | 81_6593 | 776 | 10 | Bacteroidetes | Bacteroidia | Bacteroidales | Prevotellaceae | Prevotella | Prevotella sp. |
| 13033 | 35_1783 | 16 | 5 | Bacteroidetes | Bacteroidia | Bacteroidales | Prevotellaceae | Prevotella | Prevotella copri |
| 13042 | 81_6809 | 32 | 4 | Bacteroidetes | Bacteroidia | Bacteroidales | Prevotellaceae | Prevotella | Prevotella sp. |
| 13047 | 1_30 | 9 | 5 | Bacteroidetes | Bacteroidia | Bacteroidales | Prevotellaceae | Prevotella | Prevotella denticola |
| 13053 | 39_3195 | 9 | 5 | Bacteroidetes | Bacteroidia | Bacteroidales | Porphyromonadaceae | Barnesiella | Barnsiella intestinihominis |
| 13055 | 62_3082 | 15 | 7 | Bacteroidetes | Bacteroidia | Bacteroidales | Prevotellaceae | Prevotella | Prevotella sp. |
| 13056 | 129_2999 | 241 | 10 | Bacteroidetes | Bacteroidia | Bacteroidales | Prevotellaceae | Prevotella | Prevotella sp. |
| 13063 | 39_3039 | 22 | 7 | Bacteroidetes | Bacteroidia | Bacteroidales | Prevotellaceae | Prevotella | Prevotella shahii |
| 13097 | 69_2062 | 6 | 5 | Bacteroidetes | Bacteroidia | Bacteroidales | Prevotellaceae | Prevotella | Prevotella ruminicola |
| 13103 | 104_1527 | 201 | 12 | Bacteroidetes | Bacteroidia | Bacteroidales | Prevotellaceae | Prevotella | Prevotella copri |
| 13112 | 93_3070 | 15 | 5 | Bacteroidetes | Bacteroidia | Bacteroidales | Prevotellaceae | Prevotella | Prevotella stercorea |
| 13137 | 81_3217 | 42 | 5 | Bacteroidetes | Bacteroidia | Bacteroidales | Prevotellaceae | Prevotella | Prevotella copri |
| 13138 | 81_2544 | 362 | 14 | Bacteroidetes | Bacteroidia | Bacteroidales | Prevotellaceae | Prevotella | Prevotella copri |
| 13139 | 9_5355 | 15 | 5 | Bacteroidetes | Bacteroidia | Bacteroidales | Prevotellaceae | Prevotella | Prevotella copri |
| 13141 | 81_1033 | 24 | 4 | Bacteroidetes | Bacteroidia | Bacteroidales | Prevotellaceae | Prevotella | Prevotella copri |
| 13142 | 81_6726 | 306 | 13 | Bacteroidetes | Bacteroidia | Bacteroidales | Prevotellaceae | Prevotella | Prevotella copri |
| 13153 | 121_2706 | 174 | 26 | Bacteroidetes | Bacteroidia | Bacteroidales | Prevotellaceae | Prevotella | Prevotella copri |
| 13154 | 54_3412 | 950 | 32 | Bacteroidetes | Bacteroidia | Bacteroidales | Prevotellaceae | Prevotella | Prevotella copri |
| 13155 | 1_3661 | 25 | 5 | Bacteroidetes | Bacteroidia | Bacteroidales | Prevotellaceae | Prevotella | Prevotella sp. |
| 13158 | 21_7867 | 31 | 5 | Bacteroidetes | Bacteroidia | Bacteroidales | Prevotellaceae | Prevotella | Prevotella copri |
| 13160 | 121_1022 | 1494 | 45 | Bacteroidetes | Bacteroidia | Bacteroidales | Prevotellaceae | Prevotella | Prevotella copri |
| 13161 | 121_3397 | 462 | 15 | Bacteroidetes | Bacteroidia | Bacteroidales | Prevotellaceae | Prevotella | Prevotella copri |
| 13163 | 112_1468 | 68 | 6 | Bacteroidetes | Bacteroidia | Bacteroidales | Prevotellaceae | Prevotella | Prevotella copri |
| 13164 | 121_4471 | 1987 | 45 | Bacteroidetes | Bacteroidia | Bacteroidales | Prevotellaceae | Prevotella | Prevotella copri |
| 13166 | 54_5416 | 18 | 5 | Bacteroidetes | Bacteroidia | Bacteroidales | Prevotellaceae | Prevotella | Prevotella copri |
| 13170 | 43_1868 | 65 | 8 | Bacteroidetes | Bacteroidia | Bacteroidales | Prevotellaceae | Prevotella | Prevotella copri |
| 13172 | 25_1426 | 116 | 14 | Bacteroidetes | Bacteroidia | Bacteroidales | Prevotellaceae | Prevotella | Prevotella copri |
| 13180 | 121_1793 | 30 | 9 | Bacteroidetes | Bacteroidia | Bacteroidales | Prevotellaceae | Prevotella | Prevotella copri |
| 13186 | 81_3112 | 98 | 5 | Bacteroidetes | Bacteroidia | Bacteroidales | Prevotellaceae | Prevotella | Prevotella copri |
| 13190 | 48_5302 | 11 | 5 | Bacteroidetes | Bacteroidia | Bacteroidales | Prevotellaceae | Prevotella | Prevotella copri |
| 13193 | 112_6353 | 64 | 13 | Bacteroidetes | Bacteroidia | Bacteroidales | Prevotellaceae | Prevotella | Prevotella copri |
| 13194 | 112_4403 | 1017 | 34 | Bacteroidetes | Bacteroidia | Bacteroidales | Prevotellaceae | Prevotella | Prevotella copri |
| 13204 | 4_4304 | 16 | 7 | Bacteroidetes | Bacteroidia | Bacteroidales | Prevotellaceae | Prevotella | Prevotella copri |
| 13205 | 59_4158 | 61 | 8 | Bacteroidetes | Bacteroidia | Bacteroidales | Prevotellaceae | Prevotella | Prevotella copri |
| 13209 | 112_1661 | 7 | 5 | Bacteroidetes | Bacteroidia | Bacteroidales | Prevotellaceae | Prevotella | Prevotella copri |
| 13216 | 3_297 | 12 | 8 | Bacteroidetes | Bacteroidia | Bacteroidales | Bacteroidaceae | Bacteroides | Bacteroides ovatus |
| 13225 | 104_613 | 661 | 32 | Bacteroidetes | Bacteroidia | Bacteroidales | Prevotellaceae | Prevotella | Prevotella copri |
| 13226 | 48_3808 | 69 | 7 | Bacteroidetes | Bacteroidia | Bacteroidales | Prevotellaceae | Prevotella | Prevotella copri |
| 13240 | 81_2839 | 39 | 6 | Bacteroidetes | Bacteroidia | Bacteroidales | Prevotellaceae | Prevotella | Prevotella copri |
| 13244 | 121_3483 | 980 | 20 | Bacteroidetes | Bacteroidia | Bacteroidales | Prevotellaceae | Prevotella | Prevotella copri |
| 13245 | 21_2712 | 74 | 4 | Bacteroidetes | Bacteroidia | Bacteroidales | Prevotellaceae | Prevotella | Prevotella copri |
| 13246 | 54_5540 | 27 | 5 | Bacteroidetes | Bacteroidia | Bacteroidales | Prevotellaceae | Prevotella | Prevotella copri |
| 13250 | 66_3690 | 82 | 10 | Bacteroidetes | Bacteroidia | Bacteroidales | Prevotellaceae | Prevotella | Prevotella copri |
| 13252 | 81_2397 | 30 | 8 | Bacteroidetes | Bacteroidia | Bacteroidales | Prevotellaceae | Prevotella | Prevotella copri |
| 13254 | 112_1207 | 10 | 7 | Bacteroidetes | Bacteroidia | Bacteroidales | Prevotellaceae | Prevotella | Prevotella copri |
| 13256 | 104_1560 | 19 | 6 | Bacteroidetes | Bacteroidia | Bacteroidales | Prevotellaceae | Prevotella | Prevotella copri |
| 13262 | 21_2390 | 80 | 13 | Bacteroidetes | Bacteroidia | Bacteroidales | Prevotellaceae | Prevotella | Prevotella copri |
| 13265 | 43_3373 | 46 | 10 | Bacteroidetes | Bacteroidia | Bacteroidales | Prevotellaceae | Prevotella | Prevotella copri |
| 13270 | 81_2498 | 39 | 4 | Bacteroidetes | Bacteroidia | Bacteroidales | Prevotellaceae | Prevotella | Prevotella copri |
| 13271 | 104_725 | 70 | 9 | Bacteroidetes | Bacteroidia | Bacteroidales | Prevotellaceae | Prevotella | Prevotella copri |
| 13272 | 25_863 | 35 | 8 | Bacteroidetes | Bacteroidia | Bacteroidales | Prevotellaceae | Prevotella | Prevotella copri |
| 13273 | 104_1282 | 22 | 7 | Bacteroidetes | Bacteroidia | Bacteroidales | Prevotellaceae | Prevotella | Prevotella copri |
| 13301 | 48_6265 | 15 | 5 | Bacteroidetes | Bacteroidia | Bacteroidales | Prevotellaceae | Prevotella | Prevotella copri |
| 13302 | 104_583 | 620 | 15 | Bacteroidetes | Bacteroidia | Bacteroidales | Prevotellaceae | Prevotella | Prevotella copri |
| 13303 | 4_4012 | 17 | 8 | Bacteroidetes | Bacteroidia | Bacteroidales | Bacteroidaceae | Bacteroides | Bacteroides caccae |
| 13306 | 125_5568 | 23 | 7 | Bacteroidetes | Bacteroidia | Bacteroidales | Prevotellaceae | Prevotella | Prevotella copri |
| 13317 | 43_1811 | 24 | 5 | Bacteroidetes | Bacteroidia | Bacteroidales | Prevotellaceae | Prevotella | Prevotella copri |
| 13321 | 21_4093 | 204 | 7 | Bacteroidetes | Bacteroidia | Bacteroidales | Prevotellaceae | Prevotella | Prevotella copri |
| 13322 | 1_3697 | 995 | 20 | Bacteroidetes | Bacteroidia | Bacteroidales | Prevotellaceae | Prevotella | Prevotella copri |
| 13331 | 1_1838 | 180 | 10 | Bacteroidetes | Bacteroidia | Bacteroidales | Prevotellaceae | Prevotella | Prevotella copri |
| 13335 | 121_438 | 39 | 8 | Bacteroidetes | Bacteroidia | Bacteroidales | Prevotellaceae | Prevotella | Prevotella copri |
| 13337 | 66_2174 | 58 | 8 | Bacteroidetes | Bacteroidia | Bacteroidales | Prevotellaceae | Prevotella | Prevotella copri |
| 13340 | 112_5460 | 19 | 5 | Bacteroidetes | Bacteroidia | Bacteroidales | Prevotellaceae | Prevotella | Prevotella copri |
| 13343 | 112_6038 | 21 | 5 | Bacteroidetes | Bacteroidia | Bacteroidales | Prevotellaceae | Prevotella | Prevotella copri |
| 13347 | 123_2214 | 78 | 8 | Bacteroidetes | Bacteroidia | Bacteroidales | Prevotellaceae | Prevotella | Prevotella copri |
| 13349 | 123_470 | 8 | 5 | Bacteroidetes | Bacteroidia | Bacteroidales | Prevotellaceae | Prevotella | Prevotella copri |
| 13350 | 123_5940 | 122 | 8 | Bacteroidetes | Bacteroidia | Bacteroidales | Prevotellaceae | Prevotella | Prevotella copri |
| 13354 | 23_4129 | 82 | 10 | Bacteroidetes | Bacteroidia | Bacteroidales | Prevotellaceae | Prevotella | Prevotella copri |
| 13355 | 33_1282 | 74 | 11 | Bacteroidetes | Bacteroidia | Bacteroidales | Prevotellaceae | Prevotella | Prevotella copri |
| 13359 | 107_2346 | 38 | 8 | Bacteroidetes | Bacteroidia | Bacteroidales | Prevotellaceae | Prevotella | Prevotella copri |
| 13361 | 104_148 | 77 | 10 | Bacteroidetes | Bacteroidia | Bacteroidales | Prevotellaceae | Prevotella | Prevotella copri |
| 13364 | 123_5860 | 9 | 5 | Bacteroidetes | Bacteroidia | Bacteroidales | Prevotellaceae | Prevotella | Prevotella copri |
| 13366 | 112_6356 | 15 | 7 | Bacteroidetes | Bacteroidia | Bacteroidales | Prevotellaceae | Prevotella | Prevotella copri |
| 13369 | 112_2907 | 27 | 8 | Bacteroidetes | Bacteroidia | Bacteroidales | Prevotellaceae | Prevotella | Prevotella copri |
| 13373 | 16_6627 | 44 | 8 | Bacteroidetes | Bacteroidia | Bacteroidales | Prevotellaceae | Prevotella | Prevotella copri |
| 13375 | 54_7210 | 15 | 7 | Bacteroidetes | Bacteroidia | Bacteroidales | Prevotellaceae | Prevotella | Prevotella copri |
| 13378 | 21_3940 | 25 | 4 | Bacteroidetes | Bacteroidia | Bacteroidales | Prevotellaceae | Prevotella | Prevotella copri |
| 13382 | 1_3710 | 86 | 9 | Bacteroidetes | Bacteroidia | Bacteroidales | Prevotellaceae | Prevotella | Prevotella copri |
| 13388 | 6_4402 | 139 | 6 | Bacteroidetes | Bacteroidia | Bacteroidales | Prevotellaceae | Prevotella | Prevotella copri |
| 13392 | 48_2321 | 88 | 4 | Bacteroidetes | Bacteroidia | Bacteroidales | Prevotellaceae | Prevotella | Prevotella copri |
| 13398 | 121_3518 | 18 | 5 | Bacteroidetes | Bacteroidia | Bacteroidales | Prevotellaceae | Prevotella | Prevotella copri |
| 13414 | 81_3094 | 5 | 5 | Bacteroidetes | Bacteroidia | Bacteroidales | Prevotellaceae | Prevotella | Prevotella copri |
| 13415 | 56_284 | 44 | 10 | Bacteroidetes | Bacteroidia | Bacteroidales | Bacteroidaceae | Bacteroides | Bacteroides ovatus |
| 13417 | 66_2438 | 17 | 5 | Bacteroidetes | Bacteroidia | Bacteroidales | Prevotellaceae | Paraprevotella | Paraprevotella clara |
| 13418 | 56_9738 | 29 | 6 | Bacteroidetes | Bacteroidia | Bacteroidales | Bacteroidaceae | Bacteroides | Bacteroides ovatus |
| 13440 | 128_4806 | 7 | 5 | Bacteroidetes | Bacteroidia | Bacteroidales | Prevotellaceae | Prevotella | Prevotella copri |
| 13513 | 131_9523 | 33 | 12 | Bacteroidetes | Bacteroidia | Bacteroidales | Bacteroidaceae | Bacteroides | Bacteroides eggerthii |
| 13526 | 68_3840 | 20 | 6 | Bacteroidetes | Bacteroidia | Bacteroidales | Bacteroidaceae | Bacteroides | Bacteroides ovatus |
| 13537 | 68_192 | 41 | 4 | Bacteroidetes | Bacteroidia | Bacteroidales | Bacteroidaceae | Bacteroides | Bacteroides ovatus |
| 13540 | 54_3256 | 25 | 6 | Bacteroidetes | Bacteroidia | Bacteroidales | Prevotellaceae | Prevotella | Prevotella copri |
| 13548 | 123_6488 | 20 | 8 | Bacteroidetes | Bacteroidia | Bacteroidales | Prevotellaceae | Prevotella | Prevotella copri |
| 13587 | 77_4097 | 43 | 3 | Bacteroidetes | Bacteroidia | Bacteroidales | Bacteroidaceae | Bacteroides | Bacteroides ovatus |
| 13594 | 125_1112 | 1486 | 40 | Bacteroidetes | Bacteroidia | Bacteroidales | Prevotellaceae | Prevotella | Prevotella copri |
| 13648 | 112_3162 | 6 | 5 | Firmicutes | Erysipelotrichia | Erysipelotrichales | Erysipelotrichaceae | Catenibacterium | Catenibacterium mitsuokai |
| 13754 | 49_340 | 14 | 5 | Firmicutes | Negativicutes | Selenomonadales | Acidaminococcaceae | Acidaminococcus | Acidaminococcus intestini |
| 13777 | 49_377 | 9 | 5 | Firmicutes | Negativicutes | Selenomonadales | Acidaminococcaceae | Acidaminococcus | Acidaminococcus intestini |
| 13782 | 106_3853 | 13 | 5 | Bacteroidetes | Bacteroidia | Bacteroidales | Bacteroidaceae | Bacteroides | Bacteroides sp. |
| 13785 | 106_2861 | 29 | 4 | Bacteroidetes | Bacteroidia | Bacteroidales | Prevotellaceae | Prevotella | Prevotella stercorea |
| 13789 | 117_352 | 11 | 5 | Firmicutes | Clostridia | Clostridiales | Lachnospiraceae | Blautia | Blautia |
| 13810 | 95_7016 | 17 | 10 | Firmicutes | Negativicutes | Selenomonadales | Veillonellaceae | Dialister | Dialister invisus |
| 13853 | 60_1125 | 5 | 5 | Firmicutes | Clostridia | Clostridiales | Lachnospiraceae | Roseburia | Roseburia intestinalis |
| 13900 | 84_4218 | 10 | 6 | Firmicutes | Negativicutes | Selenomonadales | Acidaminococcaceae | Acidaminococcus | Acidaminococcus intestini |
| 13917 | 84_4831 | 8 | 6 | Firmicutes | Negativicutes | Selenomonadales | Acidaminococcaceae | Acidaminococcus | Acidaminococcus intestini |
| 13919 | 84_2035 | 5 | 5 | Firmicutes | Negativicutes | Selenomonadales | Acidaminococcaceae | Acidaminococcus | Acidaminococcus intestini |
| 13920 | 49_4390 | 7 | 5 | Firmicutes | Negativicutes | Selenomonadales | Acidaminococcaceae | Acidaminococcus | Acidaminococcus intestini |
| 13990 | 106_3093 | 41 | 2 | Firmicutes | Clostridia | Clostridiales | Lactobacillaceae | Lactobacillus | Lactobacillus oris |
| 14002 | 4_167 | 10 | 8 | Bacteroidetes | Bacteroidia | Bacteroidales | Prevotellaceae | Prevotella | Prevotella copri |
| 14037 | 52_12848 | 13 | 9 | Firmicutes | Bacilli | Lactobacillales | Streptococcaceae | Lactococcus | Lactococcus lactis |
| 14061 | 57_526 | 11 | 9 | Firmicutes | Clostridia | Clostridiales | Ruminococcaceae | incertae sedis | Ruminococcaceae incertae sedis |
| 14095 | 19_6257 | 58 | 17 | Firmicutes | Clostridia | Clostridiales | Clostridiales Family XIII | Clostridiales Family XIII incertae sedis | Clostridiales Family XIII incertae sedis |
| 14108 | 88_1461 | 12 | 7 | Firmicutes | Clostridia | Clostridiales | Lachnospiraceae | Roseburia | Eubacterium rectale |
| 14109 | 99_12437 | 613 | 63 | Firmicutes | Clostridia | Clostridiales | Lachnospiraceae | Blautia | Blautia |
| 14118 | 71_3048 | 161 | 32 | Firmicutes | Clostridia | Clostridiales | Lachnospiraceae | Lachnospiraceae incertae sedis | Lachnospiraceae incertae sedis |
| 14142 | 49_843 | 10 | 6 | Firmicutes | Negativicutes | Selenomonadales | Veillonellaceae | Dialister | Dialister invisus |
| 14152 | 89_10316 | 8 | 5 | Bacteroidetes | Bacteroidia | Bacteroidales | Bacteroidaceae | Bacteroides | Bacteroides xylanisolvens |
| 14154 | 102_3215 | 32 | 10 | Bacteroidetes | Bacteroidia | Bacteroidales | Bacteroidaceae | Bacteroides | Bacteroides finegoldii |
| 14172 | 45_499 | 10336 | 32 | Proteobacteria | Gammaproteobacteria | Enterobacteriales | Enterobacteriaceae | Escherichia/Shigella | Escherichia/Shigella |
| 14190 | 81_4499 | 22 | 5 | Bacteroidetes | Bacteroidia | Bacteroidales | Prevotellaceae | Prevotella | Prevotella copri |
| 14195 | 68_5565 | 18 | 5 | Bacteroidetes | Bacteroidia | Bacteroidales | Bacteroidaceae | Bacteroides | Bacteroides ovatus |
| 14221 | 102_2894 | 12 | 7 | Bacteroidetes | Bacteroidia | Bacteroidales | Porphyromonadaceae | Barnesiella | Barnsiella intestinihominis |
| 14227 | 33_6055 | 8 | 5 | Bacteroidetes | Bacteroidia | Bacteroidales | Prevotellaceae | Prevotella | Prevotella copri |
| 14228 | 71_2442 | 6 | 5 | Bacteroidetes | Bacteroidia | Bacteroidales | Bacteroidaceae | Bacteroides | Bacteroides caccae |
| 14251 | 98_2592 | 48 | 4 | Bacteroidetes | Bacteroidia | Bacteroidales | Porphyromonadaceae | Barnesiella | Barnesiella sp. |
| 14254 | 9_2186 | 40 | 9 | Bacteroidetes | Bacteroidia | Bacteroidales | Porphyromonadaceae | Parabacteroides | Parabacteroides johnsonii |
| 14258 | 1_1957 | 82 | 36 | Bacteroidetes | Bacteroidia | Bacteroidales | Porphyromonadaceae | Parabacteroides | Parabacteroides merdae |
| 14259 | 119_4854 | 8 | 7 | Bacteroidetes | Bacteroidia | Bacteroidales | Bacteroidaceae | Bacteroides | Bacteroides massiliensis |
| 14266 | 44_5701 | 9 | 6 | Bacteroidetes | Bacteroidia | Bacteroidales | Bacteroidaceae | Bacteroides | Bacteroides ovatus |
| 14275 | 62_6061 | 7 | 6 | Bacteroidetes | Bacteroidia | Bacteroidales | Porphyromonadaceae | Parabacteroides | Parabacteroides merdae |
| 14280 | 1_2957 | 13 | 12 | Bacteroidetes | Bacteroidia | Bacteroidales | Porphyromonadaceae | Parabacteroides | Parabacteroides merdae |
| 14284 | 28_820 | 17 | 15 | Bacteroidetes | Bacteroidia | Bacteroidales | Porphyromonadaceae | Parabacteroides | Parabacteroides merdae |
| 14286 | 119_2456 | 55 | 10 | Bacteroidetes | Bacteroidia | Bacteroidales | Porphyromonadaceae | Parabacteroides | Parabacteroides merdae |
| 14288 | 9_2544 | 11 | 6 | Bacteroidetes | Bacteroidia | Bacteroidales | Porphyromonadaceae | Parabacteroides | Parabacteroides merdae |
| 14289 | 124_6097 | 7 | 6 | Bacteroidetes | Bacteroidia | Bacteroidales | Porphyromonadaceae | Parabacteroides | Parabacteroides merdae |
| 14291 | 13_2565 | 8 | 6 | Bacteroidetes | Bacteroidia | Bacteroidales | Prevotellaceae | Prevotella | Prevotella copri |
| 14293 | 101_9859 | 14 | 8 | Bacteroidetes | Bacteroidia | Bacteroidales | Porphyromonadaceae | Parabacteroides | Parabacteroides merdae |
| 14297 | 33_1795 | 5 | 5 | Bacteroidetes | Bacteroidia | Bacteroidales | Prevotellaceae | Prevotella | Prevotella copri |
| 14315 | 54_7320 | 6 | 5 | Bacteroidetes | Bacteroidia | Bacteroidales | Prevotellaceae | Prevotella | Prevotella copri |
| 14319 | 81_2755 | 15 | 6 | Bacteroidetes | Bacteroidia | Bacteroidales | Prevotellaceae | Prevotella | Prevotella copri |
| 14356 | 48_2044 | 14 | 7 | Bacteroidetes | Bacteroidia | Bacteroidales | Prevotellaceae | Prevotella | Prevotella copri |
| 14367 | 22_1875 | 9 | 5 | Bacteroidetes | Bacteroidia | Bacteroidales | Bacteroidaceae | Bacteroides | Bacteroides ovatus |
| 14368 | 122_757 | 20 | 9 | Bacteroidetes | Bacteroidia | Bacteroidales | Bacteroidaceae | Bacteroides | Bacteroides ovatus |
| 14379 | 20_6234 | 25 | 3 | Bacteroidetes | Bacteroidia | Bacteroidales | Bacteroidaceae | Bacteroides | Bacteroides intestinalis |
| 14381 | 91_9222 | 8 | 7 | Bacteroidetes | Bacteroidia | Bacteroidales | Prevotellaceae | Paraprevotella | Paraprevotella clara |
| 14383 | 91_2697 | 9 | 5 | Bacteroidetes | Bacteroidia | Bacteroidales | Bacteroidaceae | Bacteroides | Bacteroides ovatus |
| 14392 | 90_2699 | 7 | 5 | Bacteroidetes | Bacteroidia | Bacteroidales | Porphyromonadaceae | Barnesiella | Barnsiella intestinihominis |
| 14398 | 122_2164 | 15 | 8 | Bacteroidetes | Bacteroidia | Bacteroidales | Rikenellaceae | Alistipes | Alistipes sp. |
| 14399 | 130_6701 | 261 | 28 | Bacteroidetes | Bacteroidia | Bacteroidales | Rikenellaceae | Alistipes | Alistipes sp. |
| 14418 | 56_2693 | 11 | 5 | Bacteroidetes | Bacteroidia | Bacteroidales | Bacteroidaceae | Bacteroides | Bacteroides finegoldii |
| 14439 | 89_5027 | 31 | 13 | Bacteroidetes | Bacteroidia | Bacteroidales | Bacteroidaceae | Bacteroides | Bacteroides ovatus |
| 14442 | 100_5930 | 64 | 9 | Bacteroidetes | Bacteroidia | Bacteroidales | Bacteroidaceae | Bacteroides | Bacteroides sp. |
| 14444 | 77_2544 | 27 | 3 | Bacteroidetes | Bacteroidia | Bacteroidales | Bacteroidaceae | Bacteroides | Bacteroides ovatus |
| 14447 | 131_7538 | 9 | 6 | Bacteroidetes | Bacteroidia | Bacteroidales | Bacteroidaceae | Bacteroides | Bacteroides ovatus |
| 14470 | 91_3789 | 49 | 7 | Bacteroidetes | Bacteroidia | Bacteroidales | Bacteroidaceae | Bacteroides | Bacteroides ovatus |
| 14480 | 102_2289 | 176 | 20 | Bacteroidetes | Bacteroidia | Bacteroidales | Bacteroidaceae | Bacteroides | Bacteroides ovatus |
| 14482 | 79_1764 | 10 | 5 | Bacteroidetes | Bacteroidia | Bacteroidales | Bacteroidaceae | Bacteroides | Bacteroides ovatus |
| 14487 | 79_8773 | 38 | 5 | Bacteroidetes | Bacteroidia | Bacteroidales | Bacteroidaceae | Bacteroides | Bacteroides ovatus |
| 14499 | 56_9951 | 16 | 7 | Bacteroidetes | Bacteroidia | Bacteroidales | Bacteroidaceae | Bacteroides | Bacteroides ovatus |
| 14508 | 102_5410 | 27 | 4 | Bacteroidetes | Bacteroidia | Bacteroidales | Bacteroidaceae | Bacteroides | Bacteroides thetaiotaomicron |
| 14509 | 91_1760 | 8 | 5 | Bacteroidetes | Bacteroidia | Bacteroidales | Bacteroidaceae | Bacteroides | Bacteroides ovatus |
| 14514 | 123_5877 | 10 | 6 | Bacteroidetes | Bacteroidia | Bacteroidales | Porphyromonadaceae | Barnesiella | Barnsiella intestinihominis |
| 14544 | 78_1178 | 12 | 8 | Bacteroidetes | Bacteroidia | Bacteroidales | Bacteroidaceae | Bacteroides | Bacteroides ovatus |
| 14558 | 58_7851 | 12 | 6 | Bacteroidetes | Bacteroidia | Bacteroidales | Bacteroidaceae | Bacteroides | Bacteroides ovatus |
| 14571 | 105_6161 | 9 | 5 | Bacteroidetes | Bacteroidia | Bacteroidales | Bacteroidaceae | Bacteroides | Bacteroides ovatus |
| 14583 | 94_4993 | 43 | 6 | Bacteroidetes | Bacteroidia | Bacteroidales | Bacteroidaceae | Bacteroides | Bacteroides ovatus |
| 14590 | 94_5426 | 31 | 4 | Bacteroidetes | Bacteroidia | Bacteroidales | Bacteroidaceae | Bacteroides | Bacteroides ovatus |
| 14592 | 90_3253 | 113 | 19 | Bacteroidetes | Bacteroidia | Bacteroidales | Bacteroidaceae | Bacteroides | Bacteroides uniformis |
| 14593 | 81_2325 | 17 | 6 | Bacteroidetes | Bacteroidia | Bacteroidales | Prevotellaceae | Prevotella | Prevotella copri |
| 14616 | 56_4466 | 116 | 19 | Bacteroidetes | Bacteroidia | Bacteroidales | Bacteroidaceae | Bacteroides | Bacteroides ovatus |
| 14623 | 33_5885 | 26 | 6 | Bacteroidetes | Bacteroidia | Bacteroidales | Prevotellaceae | Prevotella | Prevotella copri |
| 14628 | 68_9126 | 8 | 7 | Bacteroidetes | Bacteroidia | Bacteroidales | Bacteroidaceae | Bacteroides | Bacteroides ovatus |
| 14643 | 68_5248 | 29 | 6 | Bacteroidetes | Bacteroidia | Bacteroidales | Bacteroidaceae | Bacteroides | Bacteroides ovatus |
| 14645 | 23_5487 | 20 | 9 | Bacteroidetes | Bacteroidia | Bacteroidales | Bacteroidaceae | Bacteroides | Bacteroides ovatus |
| 14650 | 2_6378 | 28 | 7 | Bacteroidetes | Bacteroidia | Bacteroidales | Bacteroidaceae | Bacteroides | Bacteroides thetaiotaomicron |
| 14653 | 91_9992 | 81 | 7 | Bacteroidetes | Bacteroidia | Bacteroidales | Bacteroidaceae | Bacteroides | Bacteroides ovatus |
| 14655 | 101_4848 | 46 | 11 | Bacteroidetes | Bacteroidia | Bacteroidales | Bacteroidaceae | Bacteroides | Bacteroides uniformis |
| 14664 | 91_8182 | 8 | 5 | Bacteroidetes | Bacteroidia | Bacteroidales | Bacteroidaceae | Bacteroides | Bacteroides ovatus |
| 14667 | 81_5994 | 11 | 5 | Bacteroidetes | Bacteroidia | Bacteroidales | Prevotellaceae | Prevotella | Prevotella copri |
| 14702 | 114_2084 | 8 | 7 | Bacteroidetes | Bacteroidia | Bacteroidales | Bacteroidaceae | Bacteroides | Bacteroides caccae |
| 14706 | 55_5485 | 126 | 37 | Bacteroidetes | Bacteroidia | Bacteroidales | Bacteroidaceae | Bacteroides | Bacteroides uniformis |
| 14709 | 67_3442 | 48 | 21 | Bacteroidetes | Bacteroidia | Bacteroidales | Bacteroidaceae | Bacteroides | Bacteroides caccae |
| 14710 | 102_5893 | 8 | 6 | Bacteroidetes | Bacteroidia | Bacteroidales | Bacteroidaceae | Bacteroides | Bacteroides caccae |
| 14722 | 89_2917 | 11 | 7 | Bacteroidetes | Bacteroidia | Bacteroidales | Bacteroidaceae | Bacteroides | Bacteroides ovatus |
| 14729 | 105_6765 | 26 | 4 | Bacteroidetes | Bacteroidia | Bacteroidales | Bacteroidaceae | Bacteroides | Bacteroides faecis |
| 14742 | 94_811 | 44 | 4 | Bacteroidetes | Bacteroidia | Bacteroidales | Bacteroidaceae | Bacteroides | Bacteroides finegoldii |
| 14747 | 75_1723 | 32 | 8 | Bacteroidetes | Bacteroidia | Bacteroidales | Bacteroidaceae | Bacteroides | Bacteroides caccae |
| 14759 | 108_6923 | 28 | 7 | Bacteroidetes | Bacteroidia | Bacteroidales | Bacteroidaceae | Bacteroides | Bacteroides thetaiotaomicron |
| 14763 | 63_2030 | 20 | 12 | Bacteroidetes | Bacteroidia | Bacteroidales | Bacteroidaceae | Bacteroides | Bacteroides caccae |
| 14777 | 106_5770 | 9 | 7 | Bacteroidetes | Bacteroidia | Bacteroidales | Bacteroidaceae | Bacteroides | Bacteroides caccae |
| 14779 | 77_6741 | 13 | 5 | Bacteroidetes | Bacteroidia | Bacteroidales | Prevotellaceae | Paraprevotella | Paraprevotella clara |
| 14784 | 130_3696 | 26 | 11 | Bacteroidetes | Bacteroidia | Bacteroidales | Bacteroidaceae | Bacteroides | Bacteroides caccae |
| 14785 | 63_2716 | 10 | 5 | Bacteroidetes | Bacteroidia | Bacteroidales | Bacteroidaceae | Bacteroides | Bacteroides uniformis |
| 14789 | 68_6662 | 22 | 6 | Bacteroidetes | Bacteroidia | Bacteroidales | Bacteroidaceae | Bacteroides | Bacteroides ovatus |
| 14794 | 13_3050 | 201 | 9 | Bacteroidetes | Bacteroidia | Bacteroidales | Bacteroidaceae | Bacteroides | Bacteroides fragilis |
| 14797 | 30_5231 | 9 | 5 | Bacteroidetes | Bacteroidia | Bacteroidales | Bacteroidaceae | Bacteroides | Bacteroides fragilis |
| 14798 | 53_2880 | 165 | 9 | Bacteroidetes | Bacteroidia | Bacteroidales | Bacteroidaceae | Bacteroides | Bacteroides fragilis |
| 14799 | 1_1711 | 82 | 11 | Bacteroidetes | Bacteroidia | Bacteroidales | Bacteroidaceae | Bacteroides | Bacteroides fragilis |
| 14800 | 65_2596 | 43 | 6 | Bacteroidetes | Bacteroidia | Bacteroidales | Bacteroidaceae | Bacteroides | Bacteroides fragilis |
| 14804 | 69_3735 | 24 | 10 | Bacteroidetes | Bacteroidia | Bacteroidales | Bacteroidaceae | Bacteroides | Bacteroides massiliensis |
| 14808 | 9_3149 | 49 | 23 | Bacteroidetes | Bacteroidia | Bacteroidales | Bacteroidaceae | Bacteroides | Bacteroides massiliensis |
| 14810 | 82_3931 | 49 | 4 | Bacteroidetes | Bacteroidia | Bacteroidales | Bacteroidaceae | Bacteroides | Bacteroides ovatus |
| 14812 | 116_1133 | 25 | 5 | Bacteroidetes | Bacteroidia | Bacteroidales | Bacteroidaceae | Bacteroides | Bacteroides sp. |
| 14827 | 107_2095 | 22 | 5 | Bacteroidetes | Bacteroidia | Bacteroidales | Bacteroidaceae | Bacteroides | Bacteroides sp. |
| 14833 | 101_4679 | 13 | 7 | Bacteroidetes | Bacteroidia | Bacteroidales | Bacteroidaceae | Bacteroides | Bacteroides ovatus |
| 14836 | 107_4615 | 30 | 6 | Bacteroidetes | Bacteroidia | Bacteroidales | Bacteroidaceae | Bacteroides | Bacteroides massiliensis |
| 14839 | 9_65 | 27 | 11 | Bacteroidetes | Bacteroidia | Bacteroidales | Bacteroidaceae | Bacteroides | Bacteroides massiliensis |
| 14853 | 69_2681 | 8 | 5 | Bacteroidetes | Bacteroidia | Bacteroidales | Prevotellaceae | Prevotella | Prevotella copri |
| 14855 | 4_1318 | 29 | 12 | Bacteroidetes | Bacteroidia | Bacteroidales | Prevotellaceae | Prevotella | Prevotella copri |
| 14869 | 105_1706 | 30 | 11 | Bacteroidetes | Bacteroidia | Bacteroidales | Bacteroidaceae | Bacteroides | Bacteroides uniformis |
| 14884 | 20_8001 | 24 | 9 | Bacteroidetes | Bacteroidia | Bacteroidales | Bacteroidaceae | Bacteroides | Bacteroides eggerthii |
| 14885 | 131_4952 | 55 | 8 | Bacteroidetes | Bacteroidia | Bacteroidales | Bacteroidaceae | Bacteroides | Bacteroides uniformis |
| 14893 | 20_8497 | 13 | 7 | Bacteroidetes | Bacteroidia | Bacteroidales | Bacteroidaceae | Bacteroides | Bacteroides eggerthii |
| 14899 | 131_3653 | 29 | 8 | Bacteroidetes | Bacteroidia | Bacteroidales | Bacteroidaceae | Bacteroides | Bacteroides eggerthii |
| 14902 | 11_1454 | 19 | 9 | Bacteroidetes | Bacteroidia | Bacteroidales | Bacteroidaceae | Bacteroides | Bacteroides eggerthii |
| 14910 | 110_5167 | 26 | 3 | Bacteroidetes | Bacteroidia | Bacteroidales | Bacteroidaceae | Bacteroides | Bacteroides eggerthii |
| 14920 | 75_1188 | 27 | 7 | Bacteroidetes | Bacteroidia | Bacteroidales | Bacteroidaceae | Bacteroides | Bacteroides uniformis |
| 14923 | 110_878 | 15 | 5 | Bacteroidetes | Bacteroidia | Bacteroidales | Bacteroidaceae | Bacteroides | Bacteroides eggerthii |
| 14946 | 108_5350 | 61 | 5 | Bacteroidetes | Bacteroidia | Bacteroidales | Prevotellaceae | Prevotella | Prevotella copri |
| 14948 | 51_1215 | 30 | 9 | Bacteroidetes | Bacteroidia | Bacteroidales | Bacteroidaceae | Bacteroides | Bacteroides uniformis |
| 14957 | 109_2649 | 17 | 10 | Bacteroidetes | Bacteroidia | Bacteroidales | Bacteroidaceae | Bacteroides | Bacteroides finegoldii |
| 14960 | 54_5703 | 22 | 10 | Bacteroidetes | Bacteroidia | Bacteroidales | Prevotellaceae | Prevotella | Prevotella copri |
| 14962 | 108_7200 | 21 | 1 | Bacteroidetes | Bacteroidia | Bacteroidales | Bacteroidaceae | Bacteroides | Bacteroides finegoldii |
| 14974 | 102_3617 | 15 | 6 | Bacteroidetes | Bacteroidia | Bacteroidales | Bacteroidaceae | Bacteroides | Bacteroides ovatus |
| 14975 | 42_3454 | 62 | 19 | Bacteroidetes | Bacteroidia | Bacteroidales | Bacteroidaceae | Bacteroides | Bacteroides uniformis |
| 14978 | 131_8359 | 21 | 3 | Bacteroidetes | Bacteroidia | Bacteroidales | Bacteroidaceae | Bacteroides | Bacteroides xylanisolvens |
| 14994 | 131_2130 | 46 | 11 | Bacteroidetes | Bacteroidia | Bacteroidales | Bacteroidaceae | Bacteroides | Bacteroides xylanisolvens |
| 14995 | 91_7013 | 13 | 6 | Bacteroidetes | Bacteroidia | Bacteroidales | Bacteroidaceae | Bacteroides | Bacteroides uniformis |
| 14997 | 111_12088 | 54 | 10 | Bacteroidetes | Bacteroidia | Bacteroidales | Bacteroidaceae | Bacteroides | Bacteroides uniformis |
| 15004 | 16_3433 | 38 | 10 | Bacteroidetes | Bacteroidia | Bacteroidales | Prevotellaceae | Prevotella | Prevotella copri |
| 15006 | 99_12360 | 115 | 9 | Bacteroidetes | Bacteroidia | Bacteroidales | Bacteroidaceae | Bacteroides | Bacteroides uniformis |
| 15018 | 18_3308 | 29 | 13 | Bacteroidetes | Bacteroidia | Bacteroidales | Bacteroidaceae | Bacteroides | Bacteroides ovatus |
| 15020 | 108_1962 | 12 | 5 | Bacteroidetes | Bacteroidia | Bacteroidales | Prevotellaceae | Prevotella | Prevotella copri |
| 15027 | 131_3587 | 15 | 6 | Bacteroidetes | Bacteroidia | Bacteroidales | Bacteroidaceae | Bacteroides | Bacteroides xylanisolvens |
| 15029 | 108_2845 | 63 | 18 | Bacteroidetes | Bacteroidia | Bacteroidales | Bacteroidaceae | Bacteroides | Bacteroides uniformis |
| 15034 | 20_1582 | 153 | 14 | Bacteroidetes | Bacteroidia | Bacteroidales | Bacteroidaceae | Bacteroides | Bacteroides eggerthii |
| 15036 | 91_5617 | 7 | 5 | Bacteroidetes | Bacteroidia | Bacteroidales | Bacteroidaceae | Bacteroides | Bacteroides finegoldii |
| 15037 | 94_4254 | 9 | 6 | Bacteroidetes | Bacteroidia | Bacteroidales | Bacteroidaceae | Bacteroides | Bacteroides finegoldii |
| 15038 | 101_9589 | 6 | 5 | Bacteroidetes | Bacteroidia | Bacteroidales | Bacteroidaceae | Bacteroides | Bacteroides uniformis |
| 15039 | 19_5985 | 9 | 7 | Bacteroidetes | Bacteroidia | Bacteroidales | Bacteroidaceae | Bacteroides | Bacteroides uniformis |
| 15045 | 109_296 | 106 | 26 | Bacteroidetes | Bacteroidia | Bacteroidales | Bacteroidaceae | Bacteroides | Bacteroides uniformis |
| 15047 | 62_3049 | 376 | 60 | Bacteroidetes | Bacteroidia | Bacteroidales | Bacteroidaceae | Bacteroides | Bacteroides uniformis |
| 15049 | 39_2668 | 33 | 14 | Bacteroidetes | Bacteroidia | Bacteroidales | Bacteroidaceae | Bacteroides | Bacteroides uniformis |
| 15053 | 90_8410 | 14 | 7 | Bacteroidetes | Bacteroidia | Bacteroidales | Bacteroidaceae | Bacteroides | Bacteroides uniformis |
| 15055 | 37_6104 | 6 | 5 | Bacteroidetes | Bacteroidia | Bacteroidales | Bacteroidaceae | Bacteroides | Bacteroides uniformis |
| 15064 | 63_3055 | 328 | 19 | Bacteroidetes | Bacteroidia | Bacteroidales | Bacteroidaceae | Bacteroides | Bacteroides uniformis |
| 15073 | 107_2311 | 93 | 31 | Bacteroidetes | Bacteroidia | Bacteroidales | Prevotellaceae | Prevotella | Prevotella copri |
| 15076 | 9_2088 | 23 | 6 | Bacteroidetes | Bacteroidia | Bacteroidales | Prevotellaceae | Prevotella | Prevotella sp. |
| 15093 | 20_4397 | 76 | 13 | Bacteroidetes | Bacteroidia | Bacteroidales | Bacteroidaceae | Bacteroides | Bacteroides uniformis |
| 15099 | 49_2851 | 41 | 20 | Bacteroidetes | Bacteroidia | Bacteroidales | Bacteroidaceae | Bacteroides | Bacteroides uniformis |
| 15112 | 23_4612 | 54 | 17 | Bacteroidetes | Bacteroidia | Bacteroidales | Prevotellaceae | Prevotella | Prevotella copri |
| 15118 | 7_2211 | 6 | 6 | Bacteroidetes | Bacteroidia | Bacteroidales | Porphyromonadaceae | Parabacteroides | Parabacteroides merdae |
| 15125 | 130_4219 | 10 | 9 | Bacteroidetes | Bacteroidia | Bacteroidales | Bacteroidaceae | Bacteroides | Bacteroides caccae |
| 15131 | 107_908 | 12 | 11 | Bacteroidetes | Bacteroidia | Bacteroidales | Prevotellaceae | Prevotella | Prevotella copri |
| 15132 | 62_5220 | 19 | 5 | Bacteroidetes | Bacteroidia | Bacteroidales | Prevotellaceae | Prevotella | Prevotella shahii |
| 15140 | 107_4463 | 14 | 9 | Bacteroidetes | Bacteroidia | Bacteroidales | Bacteroidaceae | Bacteroides | Bacteroides sp. |
| 15143 | 60_7073 | 85 | 20 | Bacteroidetes | Bacteroidia | Bacteroidales | Bacteroidaceae | Bacteroides | Bacteroides sp. |
| 15149 | 56_10685 | 134 | 25 | Bacteroidetes | Bacteroidia | Bacteroidales | Bacteroidaceae | Bacteroides | Bacteroides sp. |
| 15150 | 18_3028 | 34 | 9 | Bacteroidetes | Bacteroidia | Bacteroidales | Bacteroidaceae | Bacteroides | Bacteroides finegoldii |
| 15153 | 18_5397 | 6 | 5 | Bacteroidetes | Bacteroidia | Bacteroidales | Bacteroidaceae | Bacteroides | Bacteroides ovatus |
| 15154 | 9_2016 | 8 | 5 | Bacteroidetes | Bacteroidia | Bacteroidales | Bacteroidaceae | Bacteroides | Bacteroides finegoldii |
| 15159 | 20_4078 | 119 | 9 | Bacteroidetes | Bacteroidia | Bacteroidales | Bacteroidaceae | Bacteroides | Bacteroides eggerthii |
| 15163 | 123_1202 | 11 | 7 | Bacteroidetes | Bacteroidia | Bacteroidales | Prevotellaceae | Prevotella | Prevotella copri |
| 15171 | 104_715 | 10 | 7 | Bacteroidetes | Bacteroidia | Bacteroidales | Prevotellaceae | Prevotella | Prevotella copri |
| 15179 | 100_11979 | 33 | 6 | Bacteroidetes | Bacteroidia | Bacteroidales | Bacteroidaceae | Bacteroides | Bacteroides finegoldii |
| 15182 | 27_1140 | 15 | 6 | Bacteroidetes | Bacteroidia | Bacteroidales | Bacteroidaceae | Bacteroides | Bacteroides sp. |
| 15183 | 68_11445 | 12 | 5 | Bacteroidetes | Bacteroidia | Bacteroidales | Bacteroidaceae | Bacteroides | Bacteroides finegoldii |
| 15188 | 56_3405 | 33 | 9 | Bacteroidetes | Bacteroidia | Bacteroidales | Bacteroidaceae | Bacteroides | Bacteroides finegoldii |
| 15189 | 56_3901 | 23 | 5 | Bacteroidetes | Bacteroidia | Bacteroidales | Bacteroidaceae | Bacteroides | Bacteroides finegoldii |
| 15191 | 68_4093 | 25 | 5 | Bacteroidetes | Bacteroidia | Bacteroidales | Bacteroidaceae | Bacteroides | Bacteroides finegoldii |
| 15208 | 105_2342 | 8 | 6 | Bacteroidetes | Bacteroidia | Bacteroidales | Bacteroidaceae | Bacteroides | Bacteroides finegoldii |
| 15212 | 105_3671 | 8 | 5 | Bacteroidetes | Bacteroidia | Bacteroidales | Bacteroidaceae | Bacteroides | Bacteroides finegoldii |
| 15233 | 63_6178 | 10 | 8 | Bacteroidetes | Bacteroidia | Bacteroidales | Porphyromonadaceae | Parabacteroides | Parabacteroides merdae |
| 15235 | 75_597 | 30 | 7 | Bacteroidetes | Bacteroidia | Bacteroidales | Bacteroidaceae | Bacteroides | Bacteroides uniformis |
| 15242 | 86_2831 | 10 | 7 | Bacteroidetes | Bacteroidia | Bacteroidales | Bacteroidaceae | Bacteroides | Bacteroides caccae |
| 15244 | 120_3486 | 21 | 6 | Bacteroidetes | Bacteroidia | Bacteroidales | Bacteroidaceae | Bacteroides | Bacteroides finegoldii |
| 15245 | 120_1527 | 26 | 8 | Bacteroidetes | Bacteroidia | Bacteroidales | Bacteroidaceae | Bacteroides | Bacteroides finegoldii |
| 15263 | 24_1208 | 19 | 15 | Bacteroidetes | Bacteroidia | Bacteroidales | Prevotellaceae | Prevotella | Prevotella copri |
| 15289 | 125_5034 | 5 | 5 | Bacteroidetes | Bacteroidia | Bacteroidales | Prevotellaceae | Prevotella | Prevotella copri |
| 15302 | 100_5256 | 28 | 1 | Bacteroidetes | Bacteroidia | Bacteroidales | Bacteroidaceae | Bacteroides | Bacteroides xylanisolvens |
| 15304 | 114_17 | 65 | 7 | Bacteroidetes | Bacteroidia | Bacteroidales | Bacteroidaceae | Bacteroides | Bacteroides sp. |
| 15308 | 123_2523 | 882 | 35 | Bacteroidetes | Bacteroidia | Bacteroidales | Porphyromonadaceae | Parabacteroides | Parabacteroides distasonis |
| 15313 | 119_2843 | 33 | 22 | Bacteroidetes | Bacteroidia | Bacteroidales | Porphyromonadaceae | Parabacteroides | Parabacteroides merdae |
| 15318 | 102_3672 | 17 | 5 | Bacteroidetes | Bacteroidia | Bacteroidales | Bacteroidaceae | Bacteroides | Bacteroides ovatus |
| 15319 | 102_1901 | 31 | 10 | Bacteroidetes | Bacteroidia | Bacteroidales | Bacteroidaceae | Bacteroides | Bacteroides ovatus |
| 15328 | 23_485 | 7 | 5 | Bacteroidetes | Bacteroidia | Bacteroidales | Prevotellaceae | Prevotella | Prevotella copri |
| 15332 | 123_5669 | 10 | 7 | Bacteroidetes | Bacteroidia | Bacteroidales | Prevotellaceae | Prevotella | Prevotella copri |
| 15338 | 120_1216 | 10 | 7 | Bacteroidetes | Bacteroidia | Bacteroidales | Porphyromonadaceae | Parabacteroides | Parabacteroides merdae |
| 15340 | 69_1704 | 15 | 12 | Bacteroidetes | Bacteroidia | Bacteroidales | Porphyromonadaceae | Parabacteroides | Parabacteroides merdae |
| 15343 | 124_3736 | 8 | 7 | Bacteroidetes | Bacteroidia | Bacteroidales | Porphyromonadaceae | Parabacteroides | Parabacteroides distasonis |
| 15346 | 69_3927 | 9 | 6 | Bacteroidetes | Bacteroidia | Bacteroidales | Porphyromonadaceae | Parabacteroides | Parabacteroides merdae |
| 15366 | 108_2658 | 23 | 6 | Bacteroidetes | Bacteroidia | Bacteroidales | Prevotellaceae | Prevotella | Prevotella copri |
| 15370 | 63_5767 | 23 | 7 | Bacteroidetes | Bacteroidia | Bacteroidales | Bacteroidaceae | Bacteroides | Bacteroides finegoldii |
| 15388 | 122_1422 | 20 | 8 | Bacteroidetes | Bacteroidia | Bacteroidales | Bacteroidaceae | Bacteroides | Bacteroides caccae |
| 15389 | 67_2729 | 114 | 16 | Bacteroidetes | Bacteroidia | Bacteroidales | Bacteroidaceae | Bacteroides | Bacteroides uniformis |
| 15391 | 63_1476 | 11 | 7 | Bacteroidetes | Bacteroidia | Bacteroidales | Bacteroidaceae | Bacteroides | Bacteroides uniformis |
| 15393 | 125_3201 | 20 | 10 | Bacteroidetes | Bacteroidia | Bacteroidales | Porphyromonadaceae | Parabacteroides | Parabacteroides merdae |
| 15394 | 107_1263 | 7 | 6 | Bacteroidetes | Bacteroidia | Bacteroidales | Prevotellaceae | Prevotella | Prevotella copri |
| 15395 | 125_2647 | 19 | 12 | Bacteroidetes | Bacteroidia | Bacteroidales | Prevotellaceae | Prevotella | Prevotella copri |
| 15398 | 21_7483 | 5 | 5 | Bacteroidetes | Bacteroidia | Bacteroidales | Prevotellaceae | Paraprevotella | Paraprevotella clara |
| 15400 | 23_2753 | 28 | 12 | Bacteroidetes | Bacteroidia | Bacteroidales | Prevotellaceae | Prevotella | Prevotella copri |
| 15403 | 16_2564 | 7 | 5 | Bacteroidetes | Bacteroidia | Bacteroidales | Prevotellaceae | Prevotella | Prevotella copri |
| 15407 | 123_3359 | 9 | 6 | Bacteroidetes | Bacteroidia | Bacteroidales | Porphyromonadaceae | Parabacteroides | Parabacteroides johnsonii |
| 15410 | 123_3881 | 20 | 12 | Bacteroidetes | Bacteroidia | Bacteroidales | Prevotellaceae | Prevotella | Prevotella copri |
| 15411 | 60_3036 | 17 | 10 | Bacteroidetes | Bacteroidia | Bacteroidales | Bacteroidaceae | Bacteroides | Bacteroides sp. |
| 15413 | 114_3802 | 21 | 7 | Bacteroidetes | Bacteroidia | Bacteroidales | Porphyromonadaceae | Barnesiella | Barnsiella intestinihominis |
| 15416 | 58_7121 | 8 | 5 | Bacteroidetes | Bacteroidia | Bacteroidales | Bacteroidaceae | Bacteroides | Bacteroides ovatus |
| 15417 | 68_11584 | 31 | 15 | Bacteroidetes | Bacteroidia | Bacteroidales | Bacteroidaceae | Bacteroides | Bacteroides sp. |
| 15420 | 114_7012 | 39 | 15 | Bacteroidetes | Bacteroidia | Bacteroidales | Bacteroidaceae | Bacteroides | Bacteroides thetaiotaomicron |
| 15440 | 42_5718 | 56 | 18 | Bacteroidetes | Bacteroidia | Bacteroidales | Bacteroidaceae | Bacteroides | Bacteroides uniformis |
| 15441 | 91_2164 | 18 | 8 | Bacteroidetes | Bacteroidia | Bacteroidales | Bacteroidaceae | Bacteroides | Bacteroides ovatus |
| 15445 | 79_7365 | 10 | 5 | Bacteroidetes | Bacteroidia | Bacteroidales | Bacteroidaceae | Bacteroides | Bacteroides ovatus |
| 15452 | 54_1485 | 16 | 8 | Bacteroidetes | Bacteroidia | Bacteroidales | Prevotellaceae | Prevotella | Prevotella copri |
| 15453 | 123_3316 | 9 | 5 | Bacteroidetes | Bacteroidia | Bacteroidales | Prevotellaceae | Prevotella | Prevotella copri |
| 15458 | 4_2100 | 9 | 5 | Bacteroidetes | Bacteroidia | Bacteroidales | Prevotellaceae | Prevotella | Prevotella copri |
| 15473 | 20_8842 | 27 | 12 | Bacteroidetes | Bacteroidia | Bacteroidales | Bacteroidaceae | Bacteroides | Bacteroides eggerthii |
| 15475 | 124_3419 | 21 | 10 | Bacteroidetes | Bacteroidia | Bacteroidales | Bacteroidaceae | Bacteroides | Bacteroides vulgatus |
| 15477 | 56_11291 | 31 | 14 | Bacteroidetes | Bacteroidia | Bacteroidales | Bacteroidaceae | Bacteroides | Bacteroides sp. |
| 15480 | 108_3117 | 60 | 1 | Bacteroidetes | Bacteroidia | Bacteroidales | Bacteroidaceae | Bacteroides | Bacteroides finegoldii |
| 15481 | 108_6717 | 14 | 9 | Bacteroidetes | Bacteroidia | Bacteroidales | Bacteroidaceae | Bacteroides | Bacteroides finegoldii |
| 15483 | 60_6305 | 14 | 9 | Bacteroidetes | Bacteroidia | Bacteroidales | Bacteroidaceae | Bacteroides | Bacteroides vulgatus |
| 15484 | 4_184 | 12 | 9 | Bacteroidetes | Bacteroidia | Bacteroidales | Prevotellaceae | Prevotella | Prevotella copri |
| 15496 | 65_8064 | 7 | 5 | Bacteroidetes | Bacteroidia | Bacteroidales | Bacteroidaceae | Bacteroides | Bacteroides uniformis |
| 15529 | 69_4287 | 26 | 9 | Bacteroidetes | Bacteroidia | Bacteroidales | Prevotellaceae | Prevotella | Prevotella copri |
| 15532 | 56_1762 | 20 | 9 | Bacteroidetes | Bacteroidia | Bacteroidales | Bacteroidaceae | Bacteroides | Bacteroides ovatus |
| 15540 | 114_7995 | 16 | 11 | Bacteroidetes | Bacteroidia | Bacteroidales | Prevotellaceae | Prevotella | Prevotella copri |
| 15543 | 123_6072 | 21 | 9 | Bacteroidetes | Bacteroidia | Bacteroidales | Prevotellaceae | Prevotella | Prevotella copri |
| 15548 | 59_6913 | 30 | 13 | Bacteroidetes | Bacteroidia | Bacteroidales | Bacteroidaceae | Bacteroides | Bacteroides sp. |
| 15550 | 63_4099 | 8 | 7 | Bacteroidetes | Bacteroidia | Bacteroidales | Porphyromonadaceae | Parabacteroides | Parabacteroides merdae |
| 15551 | 4_3752 | 8 | 5 | Bacteroidetes | Bacteroidia | Bacteroidales | Prevotellaceae | Prevotella | Prevotella copri |
| 15562 | 79_7335 | 31 | 6 | Bacteroidetes | Bacteroidia | Bacteroidales | Bacteroidaceae | Bacteroides | Bacteroides ovatus |
| 15565 | 1_1644 | 7 | 6 | Bacteroidetes | Bacteroidia | Bacteroidales | Rikenellaceae | Alistipes | Alistipes onderdonkii |
| 15569 | 54_1189 | 8 | 5 | Bacteroidetes | Bacteroidia | Bacteroidales | Prevotellaceae | Prevotella | Prevotella copri |
| 15572 | 31_3037 | 23 | 5 | Bacteroidetes | Bacteroidia | Bacteroidales | Prevotellaceae | Prevotella | Prevotella copri |
| 15580 | 54_2731 | 57 | 6 | Bacteroidetes | Bacteroidia | Bacteroidales | Prevotellaceae | Prevotella | Prevotella copri |
| 15613 | 1_1544 | 9 | 5 | Bacteroidetes | Bacteroidia | Bacteroidales | Prevotellaceae | Prevotella | Prevotella copri |
| 15617 | 21_2637 | 11 | 6 | Bacteroidetes | Bacteroidia | Bacteroidales | Prevotellaceae | Prevotella | Prevotella copri |
| 15623 | 125_3785 | 18 | 5 | Bacteroidetes | Bacteroidia | Bacteroidales | Prevotellaceae | Prevotella | Prevotella sp. |
| 15633 | 70_2268 | 13 | 5 | Bacteroidetes | Bacteroidia | Bacteroidales | Prevotellaceae | Prevotella | Prevotella copri |
| 15634 | 81_3347 | 36 | 5 | Bacteroidetes | Bacteroidia | Bacteroidales | Prevotellaceae | Prevotella | Prevotella sp. |
| 15641 | 81_1718 | 48 | 3 | Bacteroidetes | Bacteroidia | Bacteroidales | Prevotellaceae | Prevotella | Prevotella sp. |
| 15648 | 70_6396 | 77 | 4 | Bacteroidetes | Bacteroidia | Bacteroidales | Prevotellaceae | Prevotella | Prevotella sp. |
| 15667 | 112_6272 | 6 | 5 | Bacteroidetes | Bacteroidia | Bacteroidales | Prevotellaceae | Prevotella | Prevotella copri |
| 15695 | 97_2284 | 22 | 5 | Bacteroidetes | Bacteroidia | Bacteroidales | Prevotellaceae | Prevotella | Prevotella sp. |
| 15700 | 39_3274 | 10 | 6 | Bacteroidetes | Bacteroidia | Bacteroidales | Bacteroidaceae | Bacteroides | Bacteroides sp. |
| 15753 | 97_180 | 10 | 5 | Bacteroidetes | Bacteroidia | Bacteroidales | Prevotellaceae | Paraprevotella | Paraprevotella clara |
| 15759 | 81_1125 | 45 | 4 | Bacteroidetes | Bacteroidia | Bacteroidales | Prevotellaceae | Prevotella | Prevotella copri |
| 15760 | 81_1631 | 17 | 5 | Bacteroidetes | Bacteroidia | Bacteroidales | Prevotellaceae | Prevotella | Prevotella copri |
| 15761 | 129_2792 | 22 | 3 | Bacteroidetes | Bacteroidia | Bacteroidales | Prevotellaceae | Prevotella | Prevotella copri |
| 15765 | 9_1942 | 29 | 4 | Bacteroidetes | Bacteroidia | Bacteroidales | Prevotellaceae | Prevotella | Prevotella copri |
| 15769 | 108_7330 | 30 | 7 | Bacteroidetes | Bacteroidia | Bacteroidales | Prevotellaceae | Prevotella | Prevotella copri |
| 15772 | 108_3110 | 143 | 4 | Bacteroidetes | Bacteroidia | Bacteroidales | Prevotellaceae | Prevotella | Prevotella copri |
| 15793 | 43_4125 | 29 | 9 | Bacteroidetes | Bacteroidia | Bacteroidales | Prevotellaceae | Prevotella | Prevotella copri |
| 15795 | 1_1851 | 14 | 6 | Bacteroidetes | Bacteroidia | Bacteroidales | Prevotellaceae | Prevotella | Prevotella copri |
| 15800 | 81_2744 | 30 | 4 | Bacteroidetes | Bacteroidia | Bacteroidales | Prevotellaceae | Prevotella | Prevotella copri |
| 15801 | 121_5725 | 6 | 5 | Bacteroidetes | Bacteroidia | Bacteroidales | Bacteroidaceae | Bacteroides | Bacteroides massiliensis |
| 15807 | 123_7535 | 239 | 8 | Bacteroidetes | Bacteroidia | Bacteroidales | Prevotellaceae | Prevotella | Prevotella copri |
| 15811 | 123_6884 | 217 | 21 | Bacteroidetes | Bacteroidia | Bacteroidales | Prevotellaceae | Prevotella | Prevotella copri |
| 15817 | 31_2900 | 7 | 5 | Bacteroidetes | Bacteroidia | Bacteroidales | Prevotellaceae | Prevotella | Prevotella copri |
| 15825 | 123_2156 | 34 | 6 | Bacteroidetes | Bacteroidia | Bacteroidales | Prevotellaceae | Prevotella | Prevotella copri |
| 15829 | 112_592 | 154 | 16 | Bacteroidetes | Bacteroidia | Bacteroidales | Prevotellaceae | Prevotella | Prevotella copri |
| 15830 | 121_6941 | 54 | 6 | Bacteroidetes | Bacteroidia | Bacteroidales | Prevotellaceae | Prevotella | Prevotella copri |
| 15832 | 43_2241 | 36 | 5 | Bacteroidetes | Bacteroidia | Bacteroidales | Prevotellaceae | Prevotella | Prevotella copri |
| 15834 | 43_410 | 36 | 4 | Bacteroidetes | Bacteroidia | Bacteroidales | Prevotellaceae | Prevotella | Prevotella copri |
| 15837 | 125_2739 | 381 | 31 | Bacteroidetes | Bacteroidia | Bacteroidales | Prevotellaceae | Prevotella | Prevotella copri |
| 15840 | 8_1040 | 9 | 6 | Bacteroidetes | Bacteroidia | Bacteroidales | Prevotellaceae | Paraprevotella | Paraprevotella clara |
| 15843 | 25_717 | 72 | 18 | Bacteroidetes | Bacteroidia | Bacteroidales | Prevotellaceae | Prevotella | Prevotella copri |
| 15847 | 59_5961 | 13 | 5 | Bacteroidetes | Bacteroidia | Bacteroidales | Prevotellaceae | Prevotella | Prevotella copri |
| 15848 | 33_2741 | 136 | 13 | Bacteroidetes | Bacteroidia | Bacteroidales | Prevotellaceae | Prevotella | Prevotella copri |
| 15853 | 4_4305 | 160 | 24 | Bacteroidetes | Bacteroidia | Bacteroidales | Prevotellaceae | Prevotella | Prevotella copri |
| 15856 | 112_3064 | 366 | 10 | Bacteroidetes | Bacteroidia | Bacteroidales | Prevotellaceae | Prevotella | Prevotella copri |
| 15873 | 112_4918 | 138 | 20 | Bacteroidetes | Bacteroidia | Bacteroidales | Prevotellaceae | Prevotella | Prevotella copri |
| 15878 | 23_5345 | 21 | 9 | Bacteroidetes | Bacteroidia | Bacteroidales | Bacteroidaceae | Bacteroides | Bacteroides ovatus |
| 15880 | 16_2878 | 40 | 7 | Bacteroidetes | Bacteroidia | Bacteroidales | Bacteroidaceae | Bacteroides | Bacteroides uniformis |
| 15891 | 54_3247 | 12 | 7 | Bacteroidetes | Bacteroidia | Bacteroidales | Prevotellaceae | Prevotella | Prevotella copri |
| 15893 | 104_758 | 24 | 8 | Bacteroidetes | Bacteroidia | Bacteroidales | Prevotellaceae | Prevotella | Prevotella copri |
| 15897 | 69_1633 | 7 | 7 | Bacteroidetes | Bacteroidia | Bacteroidales | Bacteroidaceae | Bacteroides | Bacteroides caccae |
| 15908 | 123_3553 | 22 | 8 | Bacteroidetes | Bacteroidia | Bacteroidales | Prevotellaceae | Prevotella | Prevotella copri |
| 15909 | 71_8390 | 7 | 6 | Bacteroidetes | Bacteroidia | Bacteroidales | Prevotellaceae | Prevotella | Prevotella copri |
| 15912 | 33_4535 | 15 | 5 | Bacteroidetes | Bacteroidia | Bacteroidales | Prevotellaceae | Prevotella | Prevotella copri |
| 15915 | 71_1316 | 17 | 6 | Bacteroidetes | Bacteroidia | Bacteroidales | Prevotellaceae | Prevotella | Prevotella copri |
| 15917 | 4_1073 | 5 | 5 | Bacteroidetes | Bacteroidia | Bacteroidales | Prevotellaceae | Prevotella | Prevotella copri |
| 15927 | 104_543 | 20 | 4 | Bacteroidetes | Bacteroidia | Bacteroidales | Prevotellaceae | Prevotella | Prevotella copri |
| 15928 | 104_1356 | 8 | 5 | Bacteroidetes | Bacteroidia | Bacteroidales | Prevotellaceae | Prevotella | Prevotella copri |
| 15943 | 81_6556 | 32 | 5 | Bacteroidetes | Bacteroidia | Bacteroidales | Prevotellaceae | Prevotella | Prevotella copri |
| 15944 | 81_1396 | 33 | 7 | Bacteroidetes | Bacteroidia | Bacteroidales | Prevotellaceae | Prevotella | Prevotella copri |
| 15948 | 81_4679 | 36 | 4 | Bacteroidetes | Bacteroidia | Bacteroidales | Prevotellaceae | Prevotella | Prevotella copri |
| 15963 | 104_1464 | 75 | 12 | Bacteroidetes | Bacteroidia | Bacteroidales | Prevotellaceae | Prevotella | Prevotella copri |
| 15964 | 104_569 | 41 | 10 | Bacteroidetes | Bacteroidia | Bacteroidales | Prevotellaceae | Prevotella | Prevotella copri |
| 16006 | 121_5959 | 157 | 4 | Bacteroidetes | Bacteroidia | Bacteroidales | Prevotellaceae | Prevotella | Prevotella copri |
| 16007 | 1_2266 | 11 | 7 | Bacteroidetes | Bacteroidia | Bacteroidales | Prevotellaceae | Prevotella | Prevotella copri |
| 16009 | 112_5887 | 35 | 9 | Bacteroidetes | Bacteroidia | Bacteroidales | Prevotellaceae | Prevotella | Prevotella copri |
| 16013 | 21_3087 | 22 | 5 | Bacteroidetes | Bacteroidia | Bacteroidales | Prevotellaceae | Prevotella | Prevotella copri |
| 16021 | 96_4693 | 50 | 8 | Bacteroidetes | Bacteroidia | Bacteroidales | Prevotellaceae | Prevotella | Prevotella copri |
| 16023 | 1_1861 | 13 | 6 | Bacteroidetes | Bacteroidia | Bacteroidales | Prevotellaceae | Prevotella | Prevotella copri |
| 16026 | 104_446 | 136 | 12 | Bacteroidetes | Bacteroidia | Bacteroidales | Prevotellaceae | Prevotella | Prevotella copri |
| 16036 | 121_3292 | 17 | 6 | Bacteroidetes | Bacteroidia | Bacteroidales | Prevotellaceae | Prevotella | Prevotella copri |
| 16041 | 112_5319 | 68 | 7 | Bacteroidetes | Bacteroidia | Bacteroidales | Prevotellaceae | Prevotella | Prevotella copri |
| 16045 | 123_3895 | 13 | 5 | Bacteroidetes | Bacteroidia | Bacteroidales | Bacteroidaceae | Bacteroides | Bacteroides ovatus |
| 16046 | 123_2583 | 25 | 6 | Bacteroidetes | Bacteroidia | Bacteroidales | Prevotellaceae | Prevotella | Prevotella copri |
| 16054 | 123_6929 | 22 | 11 | Bacteroidetes | Bacteroidia | Bacteroidales | Prevotellaceae | Prevotella | Prevotella copri |
| 16057 | 123_2162 | 7 | 5 | Bacteroidetes | Bacteroidia | Bacteroidales | Prevotellaceae | Prevotella | Prevotella copri |
| 16059 | 104_923 | 15 | 6 | Bacteroidetes | Bacteroidia | Bacteroidales | Prevotellaceae | Prevotella | Prevotella copri |
| 16070 | 112_3141 | 41 | 13 | Bacteroidetes | Bacteroidia | Bacteroidales | Prevotellaceae | Prevotella | Prevotella copri |
| 16082 | 8_1969 | 7 | 6 | Bacteroidetes | Bacteroidia | Bacteroidales | Bacteroidaceae | Bacteroides | Bacteroides uniformis |
| 16099 | 48_6260 | 51 | 4 | Bacteroidetes | Bacteroidia | Bacteroidales | Prevotellaceae | Prevotella | Prevotella copri |
| 16105 | 48_6344 | 33 | 3 | Bacteroidetes | Bacteroidia | Bacteroidales | Prevotellaceae | Prevotella | Prevotella copri |
| 16107 | 48_1131 | 23 | 2 | Bacteroidetes | Bacteroidia | Bacteroidales | Prevotellaceae | Prevotella | Prevotella copri |
| 16119 | 66_2527 | 11 | 6 | Bacteroidetes | Bacteroidia | Bacteroidales | Prevotellaceae | Prevotella | Prevotella copri |
| 16145 | 58_8528 | 18 | 6 | Bacteroidetes | Bacteroidia | Bacteroidales | Bacteroidaceae | Bacteroides | Bacteroides ovatus |
| 16150 | 33_3015 | 15 | 7 | Bacteroidetes | Bacteroidia | Bacteroidales | Prevotellaceae | Prevotella | Prevotella copri |
| 16155 | 77_5005 | 43 | 3 | Bacteroidetes | Bacteroidia | Bacteroidales | Bacteroidaceae | Bacteroides | Bacteroides xylanisolvens |
| 16156 | 102_2629 | 26 | 9 | Bacteroidetes | Bacteroidia | Bacteroidales | Bacteroidaceae | Bacteroides | Bacteroides vulgatus |
| 16157 | 43_3647 | 17 | 9 | Bacteroidetes | Bacteroidia | Bacteroidales | Prevotellaceae | Prevotella | Prevotella copri |
| 16175 | 58_8221 | 52 | 15 | Bacteroidetes | Bacteroidia | Bacteroidales | Bacteroidaceae | Bacteroides | Bacteroides vulgatus |
| 16187 | 5_774 | 5 | 5 | Bacteroidetes | Bacteroidia | Bacteroidales | Prevotellaceae | Prevotella | Prevotella copri |
| 16191 | 62_1786 | 5 | 5 | Bacteroidetes | Bacteroidia | Bacteroidales | Porphyromonadaceae | Parabacteroides | Parabacteroides merdae |
| 16226 | 71_733 | 98 | 12 | Verrucomicrobia | Verrucomicrobiae | Verrucomicrobiales | Akkermansiaceae | Akkermansia | Akkermansia muciniphila |
| 16237 | 102_723 | 7 | 5 | Bacteroidetes | Bacteroidia | Bacteroidales | Prevotellaceae | Prevotella | Prevotella copri |
| 16259 | 105_3713 | 22 | 4 | Bacteroidetes | Bacteroidia | Bacteroidales | Bacteroidaceae | Bacteroides | Bacteroides massiliensis |
| 16265 | 50_1545 | 80 | 21 | Bacteroidetes | Bacteroidia | Bacteroidales | Prevotellaceae | Prevotella | Prevotella copri |
| 16290 | 112_2647 | 9 | 5 | Bacteroidetes | Bacteroidia | Bacteroidales | Prevotellaceae | Prevotella | Prevotella copri |
| 16297 | 17_3172 | 10 | 6 | Bacteroidetes | Bacteroidia | Bacteroidales | Prevotellaceae | Prevotella | Prevotella copri |
| 16308 | 99_3728 | 37 | 9 | Bacteroidetes | Bacteroidia | Bacteroidales | Bacteroidaceae | Bacteroides | Bacteroides uniformis |
| 16321 | 24_3032 | 6 | 5 | Bacteroidetes | Bacteroidia | Bacteroidales | Bacteroidaceae | Bacteroides | Bacteroides uniformis |
| 16336 | 105_5756 | 6 | 5 | Bacteroidetes | Bacteroidia | Bacteroidales | Bacteroidaceae | Bacteroides | Bacteroides finegoldii |
| 16353 | 126_234 | 6 | 6 | Bacteroidetes | Bacteroidia | Bacteroidales | Bacteroidaceae | Bacteroides | Bacteroides finegoldii |
| 16363 | 115_2075 | 117 | 11 | Bacteroidetes | Bacteroidia | Bacteroidales | Prevotellaceae | Prevotella | Prevotella copri |
| 16365 | 69_4738 | 16 | 10 | Bacteroidetes | Bacteroidia | Bacteroidales | Porphyromonadaceae | Parabacteroides | Parabacteroides merdae |
| 16371 | 56_2460 | 39 | 2 | Bacteroidetes | Bacteroidia | Bacteroidales | Bacteroidaceae | Bacteroides | Bacteroides ovatus |
| 16378 | 71_4840 | 25 | 6 | Verrucomicrobia | Verrucomicrobiae | Verrucomicrobiales | Akkermansiaceae | Akkermansia | Akkermansia muciniphila |
| 16388 | 60_3272 | 10 | 5 | Firmicutes | Negativicutes | Selenomonadales | Veillonellaceae | Dialister | Dialister invisus |
| 16415 | 1_1560 | 15 | 6 | Firmicutes | Clostridia | Clostridiales | Lactobacillaceae | Lactobacillus | Lactobacillus ruminis |
| 16435 | 66_5027 | 6 | 5 | Firmicutes | Clostridia | Clostridiales | Ruminococcaceae | Faecalibacterium | Faecalibacterium prausnitzii |
| 16445 | 115_1289 | 16 | 7 | Firmicutes | Erysipelotrichia | Erysipelotrichales | Erysipelotrichaceae | Holdemanella | Eubacterium biforme |
| 16662 | 55_6150 | 447 | 8 | Firmicutes | Negativicutes | Selenomonadales | Veillonellaceae | Dialister | Dialister invisus |
| 16666 | 104_398 | 13 | 5 | Bacteroidetes | Bacteroidia | Bacteroidales | Prevotellaceae | Prevotella | Prevotella copri |
| 16671 | 81_5917 | 32 | 7 | Firmicutes | Negativicutes | Selenomonadales | Veillonellaceae | Dialister | Dialister invisus |
| 16683 | 2_2014 | 6 | 5 | Firmicutes | Negativicutes | Selenomonadales | Veillonellaceae | Dialister | Dialister invisus |
| 16709 | 117_2443 | 10 | 7 | Bacteroidetes | Bacteroidia | Bacteroidales | Bacteroidaceae | Bacteroides | Bacteroides massiliensis |
| 16714 | 72_461 | 6 | 5 | Firmicutes | Negativicutes | Selenomonadales | Acidaminococcaceae | Acidaminococcus | Acidaminococcus intestini |
| 16774 | 37_2708 | 7 | 5 | Firmicutes | Negativicutes | Selenomonadales | Veillonellaceae | Dialister | Dialister invisus |
| 16813 | 12_3971 | 26 | 10 | Firmicutes | Clostridia | Clostridiales | Lactobacillaceae | Lactobacillus | Lactobacillus salivarius |
| 16841 | 1_2215 | 17 | 12 | Firmicutes | Clostridia | Clostridiales | Ruminococcaceae | Faecalibacterium | Faecalibacterium prausnitzii |
| 16842 | 76_4790 | 406 | 38 | Firmicutes | Clostridia | Clostridiales | Ruminococcaceae | Faecalibacterium | Faecalibacterium prausnitzii |
| 16849 | 51_810 | 109 | 24 | Firmicutes | Clostridia | Clostridiales | Ruminococcaceae | Faecalibacterium | Faecalibacterium prausnitzii |
| 16867 | 120_811 | 5 | 5 | Bacteroidetes | Bacteroidia | Bacteroidales | Bacteroidaceae | Bacteroides | Bacteroides vulgatus |
| 16887 | 18_3147 | 576 | 38 | Actinobacteria | Coriobacteriia | Coriobacteriales | Coriobacteriaceae | Collinsella | Collinsella aerofaciens |
| 16916 | 99_2005 | 8 | 5 | Firmicutes | Clostridia | Clostridiales | Lachnospiraceae | Blautia | Blautia |
| 16930 | 18_5079 | 49 | 16 | Firmicutes | Negativicutes | Selenomonadales | Veillonellaceae | Dialister | Dialister invisus |
| 16941 | 122_2984 | 65 | 20 | Firmicutes | Clostridia | Clostridiales | Clostridiaceae | Clostridium | Clostridium sp. |
| 16961 | 95_7283 | 8 | 5 | Firmicutes | Negativicutes | Selenomonadales | Veillonellaceae | Dialister | Dialister invisus |
| 16991 | 106_4062 | 11 | 6 | Bacteroidetes | Bacteroidia | Bacteroidales | Bacteroidaceae | Bacteroides | Bacteroides caccae |
| 17052 | 125_2896 | 30 | 5 | Bacteroidetes | Bacteroidia | Bacteroidales | Prevotellaceae | Prevotella | Prevotella copri |
| 17054 | 4_960 | 11 | 7 | Bacteroidetes | Bacteroidia | Bacteroidales | Porphyromonadaceae | Parabacteroides | Parabacteroides merdae |
| 17069 | 78_4750 | 14 | 11 | Bacteroidetes | Bacteroidia | Bacteroidales | Bacteroidaceae | Bacteroides | Bacteroides uniformis |
| 17132 | 25_2516 | 29 | 5 | NULL | NULL | NULL | NULL | NULL | NULL |
| 17146 | 110_5487 | 24 | 2 | Bacteroidetes | Bacteroidia | Bacteroidales | Porphyromonadaceae | Barnesiella | Barnesiella sp. |
| 17151 | 123_7771 | 69 | 13 | Bacteroidetes | Bacteroidia | Bacteroidales | Porphyromonadaceae | Parabacteroides | Parabacteroides johnsonii |
| 17168 | 124_7363 | 29 | 20 | Bacteroidetes | Bacteroidia | Bacteroidales | Porphyromonadaceae | Parabacteroides | Parabacteroides merdae |
| 17175 | 8_2079 | 6 | 5 | Bacteroidetes | Bacteroidia | Bacteroidales | Prevotellaceae | Prevotella | Prevotella copri |
| 17187 | 91_4109 | 6 | 5 | Bacteroidetes | Bacteroidia | Bacteroidales | Bacteroidaceae | Bacteroides | Bacteroides ovatus |
| 17188 | 69_4283 | 32 | 18 | Bacteroidetes | Bacteroidia | Bacteroidales | Bacteroidaceae | Bacteroides | Bacteroides uniformis |
| 17198 | 50_2497 | 27 | 14 | Bacteroidetes | Bacteroidia | Bacteroidales | Bacteroidaceae | Bacteroides | Bacteroides uniformis |
| 17202 | 97_5082 | 30 | 11 | Bacteroidetes | Bacteroidia | Bacteroidales | Porphyromonadaceae | Parabacteroides | Parabacteroides merdae |
| 17203 | 130_6217 | 7 | 6 | Bacteroidetes | Bacteroidia | Bacteroidales | Bacteroidaceae | Bacteroides | Bacteroides caccae |
| 17206 | 115_1712 | 15 | 12 | Bacteroidetes | Bacteroidia | Bacteroidales | Porphyromonadaceae | Parabacteroides | Parabacteroides merdae |
| 17212 | 104_1117 | 15 | 12 | Bacteroidetes | Bacteroidia | Bacteroidales | Porphyromonadaceae | Parabacteroides | Parabacteroides merdae |
| 17213 | 101_585 | 40 | 17 | Bacteroidetes | Bacteroidia | Bacteroidales | Porphyromonadaceae | Parabacteroides | Parabacteroides merdae |
| 17218 | 60_4309 | 12 | 8 | Bacteroidetes | Bacteroidia | Bacteroidales | Porphyromonadaceae | Parabacteroides | Parabacteroides merdae |
| 17233 | 75_1650 | 10 | 9 | Bacteroidetes | Bacteroidia | Bacteroidales | Bacteroidaceae | Bacteroides | Bacteroides caccae |
| 17246 | 1_2237 | 7 | 5 | Bacteroidetes | Bacteroidia | Bacteroidales | Prevotellaceae | Prevotella | Prevotella copri |
| 17252 | 10_2232 | 110 | 34 | Bacteroidetes | Bacteroidia | Bacteroidales | Bacteroidaceae | Bacteroides | Bacteroides uniformis |
| 17253 | 24_5590 | 28 | 16 | Bacteroidetes | Bacteroidia | Bacteroidales | Bacteroidaceae | Bacteroides | Bacteroides massiliensis |
| 17254 | 113_2665 | 6 | 6 | Bacteroidetes | Bacteroidia | Bacteroidales | Bacteroidaceae | Bacteroides | Bacteroides massiliensis |
| 17269 | 4_509 | 10 | 8 | Bacteroidetes | Bacteroidia | Bacteroidales | Prevotellaceae | Prevotella | Prevotella copri |
| 17273 | 86_922 | 9 | 6 | Bacteroidetes | Bacteroidia | Bacteroidales | Bacteroidaceae | Bacteroides | Bacteroides caccae |
| 17299 | 71_4090 | 1889 | 10 | Bacteroidetes | Bacteroidia | Bacteroidales | Bacteroidaceae | Bacteroides | Bacteroides plebeius |
| 17306 | 58_4302 | 23 | 3 | Bacteroidetes | Bacteroidia | Bacteroidales | Bacteroidaceae | Bacteroides | Bacteroides plebeius |
| 17325 | 75_1330 | 25 | 11 | Bacteroidetes | Bacteroidia | Bacteroidales | Bacteroidaceae | Bacteroides | Bacteroides uniformis |
| 17327 | 75_3122 | 8 | 5 | Bacteroidetes | Bacteroidia | Bacteroidales | Bacteroidaceae | Bacteroides | Bacteroides eggerthii |
| 17328 | 91_841 | 48 | 7 | Bacteroidetes | Bacteroidia | Bacteroidales | Bacteroidaceae | Bacteroides | Bacteroides finegoldii |
| 17344 | 20_4215 | 72 | 3 | Bacteroidetes | Bacteroidia | Bacteroidales | Bacteroidaceae | Bacteroides | Bacteroides eggerthii |
| 17365 | 20_7241 | 7 | 5 | Bacteroidetes | Bacteroidia | Bacteroidales | Bacteroidaceae | Bacteroides | Bacteroides ovatus |
| 17375 | 20_7328 | 102 | 3 | Bacteroidetes | Bacteroidia | Bacteroidales | Bacteroidaceae | Bacteroides | Bacteroides eggerthii |
| 17394 | 32_5322 | 21 | 5 | Bacteroidetes | Bacteroidia | Bacteroidales | Rikenellaceae | Alistipes | Alistipes onderdonkii |
| 17398 | 63_6336 | 1605 | 57 | Bacteroidetes | Bacteroidia | Bacteroidales | Rikenellaceae | Alistipes | Alistipes sp. |
| 17423 | 123_28 | 11 | 6 | Bacteroidetes | Bacteroidia | Bacteroidales | Porphyromonadaceae | Barnesiella | Barnsiella intestinihominis |
| 17431 | 9_2195 | 9 | 5 | Bacteroidetes | Bacteroidia | Bacteroidales | Bacteroidaceae | Bacteroides | Bacteroides uniformis |
| 17437 | 100_11833 | 17 | 10 | Bacteroidetes | Bacteroidia | Bacteroidales | Bacteroidaceae | Bacteroides | Bacteroides sp. |
| 17474 | 89_6285 | 6 | 5 | Bacteroidetes | Bacteroidia | Bacteroidales | Bacteroidaceae | Bacteroides | Bacteroides ovatus |
| 17475 | 100_2672 | 37 | 4 | Bacteroidetes | Bacteroidia | Bacteroidales | Bacteroidaceae | Bacteroides | Bacteroides ovatus |
| 17483 | 100_5432 | 162 | 15 | Bacteroidetes | Bacteroidia | Bacteroidales | Bacteroidaceae | Bacteroides | Bacteroides sp. |
| 17485 | 100_9386 | 9 | 5 | Bacteroidetes | Bacteroidia | Bacteroidales | Prevotellaceae | Paraprevotella | Paraprevotella clara |
| 17487 | 131_8221 | 149 | 30 | Bacteroidetes | Bacteroidia | Bacteroidales | Bacteroidaceae | Bacteroides | Bacteroides uniformis |
| 17490 | 131_4213 | 27 | 1 | Bacteroidetes | Bacteroidia | Bacteroidales | Bacteroidaceae | Bacteroides | Bacteroides sp. |
| 17494 | 131_4423 | 195 | 14 | Bacteroidetes | Bacteroidia | Bacteroidales | Bacteroidaceae | Bacteroides | Bacteroides uniformis |
| 17527 | 102_134 | 28 | 6 | Bacteroidetes | Bacteroidia | Bacteroidales | Bacteroidaceae | Bacteroides | Bacteroides ovatus |
| 17531 | 79_6926 | 11 | 5 | Bacteroidetes | Bacteroidia | Bacteroidales | Bacteroidaceae | Bacteroides | Bacteroides ovatus |
| 17537 | 68_11729 | 20 | 5 | Bacteroidetes | Bacteroidia | Bacteroidales | Bacteroidaceae | Bacteroides | Bacteroides ovatus |
| 17538 | 91_4212 | 29 | 7 | Bacteroidetes | Bacteroidia | Bacteroidales | Bacteroidaceae | Bacteroides | Bacteroides ovatus |
| 17566 | 91_4943 | 11 | 5 | Bacteroidetes | Bacteroidia | Bacteroidales | Bacteroidaceae | Bacteroides | Bacteroides ovatus |
| 17574 | 68_9336 | 9 | 6 | Bacteroidetes | Bacteroidia | Bacteroidales | Bacteroidaceae | Bacteroides | Bacteroides thetaiotaomicron |
| 17576 | 131_9469 | 15 | 7 | Bacteroidetes | Bacteroidia | Bacteroidales | Bacteroidaceae | Bacteroides | Bacteroides uniformis |
| 17599 | 130_1197 | 93 | 27 | Bacteroidetes | Bacteroidia | Bacteroidales | Bacteroidaceae | Bacteroides | Bacteroides uniformis |
| 17619 | 114_5778 | 12 | 6 | Bacteroidetes | Bacteroidia | Bacteroidales | Prevotellaceae | Prevotella | Prevotella copri |
| 17630 | 60_840 | 153 | 13 | Bacteroidetes | Bacteroidia | Bacteroidales | Bacteroidaceae | Bacteroides | Bacteroides ovatus |
| 17637 | 43_2094 | 7 | 5 | Bacteroidetes | Bacteroidia | Bacteroidales | Prevotellaceae | Prevotella | Prevotella copri |
| 17640 | 123_3118 | 9 | 5 | Bacteroidetes | Bacteroidia | Bacteroidales | Prevotellaceae | Prevotella | Prevotella copri |
| 17641 | 58_384 | 12 | 7 | Bacteroidetes | Bacteroidia | Bacteroidales | Bacteroidaceae | Bacteroides | Bacteroides ovatus |
| 17648 | 5_1351 | 11 | 7 | Bacteroidetes | Bacteroidia | Bacteroidales | Porphyromonadaceae | Barnesiella | Barnesiella intestinihominis |
| 17672 | 82_8200 | 14 | 6 | Bacteroidetes | Bacteroidia | Bacteroidales | Bacteroidaceae | Bacteroides | Bacteroides ovatus |
| 17677 | 70_6738 | 16 | 10 | Bacteroidetes | Bacteroidia | Bacteroidales | Bacteroidaceae | Bacteroides | Bacteroides ovatus |
| 17682 | 122_2460 | 16 | 8 | Bacteroidetes | Bacteroidia | Bacteroidales | Bacteroidaceae | Bacteroides | Bacteroides thetaiotaomicron |
| 17686 | 112_5456 | 18 | 12 | Bacteroidetes | Bacteroidia | Bacteroidales | Prevotellaceae | Prevotella | Prevotella copri |
| 17708 | 79_8512 | 10 | 6 | Bacteroidetes | Bacteroidia | Bacteroidales | Bacteroidaceae | Bacteroides | Bacteroides ovatus |
| 17713 | 68_10524 | 10 | 6 | Bacteroidetes | Bacteroidia | Bacteroidales | Bacteroidaceae | Bacteroides | Bacteroides ovatus |
| 17714 | 68_12285 | 28 | 6 | Bacteroidetes | Bacteroidia | Bacteroidales | Bacteroidaceae | Bacteroides | Bacteroides ovatus |
| 17721 | 91_5404 | 63 | 6 | Bacteroidetes | Bacteroidia | Bacteroidales | Bacteroidaceae | Bacteroides | Bacteroides sp. |
| 17745 | 82_5242 | 11 | 5 | Bacteroidetes | Bacteroidia | Bacteroidales | Bacteroidaceae | Bacteroides | Bacteroides massiliensis |
| 17761 | 91_5599 | 33 | 5 | Bacteroidetes | Bacteroidia | Bacteroidales | Bacteroidaceae | Bacteroides | Bacteroides thetaiotaomicron |
| 17763 | 2_6506 | 18 | 7 | Bacteroidetes | Bacteroidia | Bacteroidales | Bacteroidaceae | Bacteroides | Bacteroides thetaiotaomicron |
| 17767 | 79_4026 | 23 | 7 | Bacteroidetes | Bacteroidia | Bacteroidales | Bacteroidaceae | Bacteroides | Bacteroides thetaiotaomicron |
| 17769 | 102_5039 | 38 | 6 | Bacteroidetes | Bacteroidia | Bacteroidales | Bacteroidaceae | Bacteroides | Bacteroides thetaiotaomicron |
| 17774 | 101_1037 | 14 | 7 | Bacteroidetes | Bacteroidia | Bacteroidales | Bacteroidaceae | Bacteroides | Bacteroides thetaiotaomicron |
| 17776 | 4_1667 | 6 | 5 | Bacteroidetes | Bacteroidia | Bacteroidales | Bacteroidaceae | Bacteroides | Bacteroides thetaiotaomicron |
| 17779 | 50_1615 | 6 | 5 | Bacteroidetes | Bacteroidia | Bacteroidales | Prevotellaceae | Prevotella | Prevotella copri |
| 17789 | 130_7432 | 23 | 10 | Bacteroidetes | Bacteroidia | Bacteroidales | Bacteroidaceae | Bacteroides | Bacteroides thetaiotaomicron |
| 17836 | 41_9626 | 12 | 11 | Bacteroidetes | Bacteroidia | Bacteroidales | Bacteroidaceae | Bacteroides | Bacteroides caccae |
| 17838 | 107_4159 | 17 | 7 | Bacteroidetes | Bacteroidia | Bacteroidales | Bacteroidaceae | Bacteroides | Bacteroides uniformis |
| 17839 | 120_2238 | 11 | 7 | Bacteroidetes | Bacteroidia | Bacteroidales | Bacteroidaceae | Bacteroides | Bacteroides sp. |
| 17842 | 100_6099 | 20 | 1 | Bacteroidetes | Bacteroidia | Bacteroidales | Bacteroidaceae | Bacteroides | Bacteroides sp. |
| 17864 | 45_7684 | 5 | 5 | Bacteroidetes | Bacteroidia | Bacteroidales | Bacteroidaceae | Bacteroides | Bacteroides caccae |
| 17884 | 101_4676 | 23 | 11 | Bacteroidetes | Bacteroidia | Bacteroidales | Bacteroidaceae | Bacteroides | Bacteroides uniformis |
| 17910 | 105_5593 | 16 | 10 | Bacteroidetes | Bacteroidia | Bacteroidales | Bacteroidaceae | Bacteroides | Bacteroides caccae |
| 17923 | 120_3400 | 25 | 6 | Bacteroidetes | Bacteroidia | Bacteroidales | Bacteroidaceae | Bacteroides | Bacteroides eggerthii |
| 17926 | 69_2459 | 12 | 8 | Bacteroidetes | Bacteroidia | Bacteroidales | Bacteroidaceae | Bacteroides | Bacteroides caccae |
| 17927 | 105_7168 | 8 | 7 | Bacteroidetes | Bacteroidia | Bacteroidales | Bacteroidaceae | Bacteroides | Bacteroides caccae |
| 17931 | 18_5477 | 14 | 9 | Bacteroidetes | Bacteroidia | Bacteroidales | Bacteroidaceae | Bacteroides | Bacteroides caccae |
| 17937 | 20_6858 | 10 | 8 | Bacteroidetes | Bacteroidia | Bacteroidales | Bacteroidaceae | Bacteroides | Bacteroides caccae |
| 17946 | 105_3578 | 24 | 3 | Bacteroidetes | Bacteroidia | Bacteroidales | Bacteroidaceae | Bacteroides | Bacteroides thetaiotaomicron |
| 17947 | 105_6527 | 163 | 12 | Bacteroidetes | Bacteroidia | Bacteroidales | Bacteroidaceae | Bacteroides | Bacteroides thetaiotaomicron |
| 17948 | 105_4303 | 37 | 10 | Bacteroidetes | Bacteroidia | Bacteroidales | Bacteroidaceae | Bacteroides | Bacteroides sp. |
| 17958 | 100_9558 | 27 | 6 | Bacteroidetes | Bacteroidia | Bacteroidales | Bacteroidaceae | Bacteroides | Bacteroides sp. |
| 17967 | 30_10274 | 164 | 10 | Bacteroidetes | Bacteroidia | Bacteroidales | Bacteroidaceae | Bacteroides | Bacteroides fragilis |
| 17968 | 65_8072 | 14 | 5 | Bacteroidetes | Bacteroidia | Bacteroidales | Bacteroidaceae | Bacteroides | Bacteroides fragilis |
| 17971 | 53_5904 | 47 | 7 | Bacteroidetes | Bacteroidia | Bacteroidales | Bacteroidaceae | Bacteroides | Bacteroides fragilis |
| 17991 | 55_6962 | 89 | 8 | Bacteroidetes | Bacteroidia | Bacteroidales | Bacteroidaceae | Bacteroides | Bacteroides uniformis |
| 18003 | 71_7298 | 1060 | 41 | Bacteroidetes | Bacteroidia | Bacteroidales | Bacteroidaceae | Bacteroides | Bacteroides massiliensis |
| 18006 | 1_96 | 16 | 12 | Bacteroidetes | Bacteroidia | Bacteroidales | Prevotellaceae | Prevotella | Prevotella copri |
| 18028 | 78_8803 | 43 | 3 | Bacteroidetes | Bacteroidia | Bacteroidales | Bacteroidaceae | Bacteroides | Bacteroides uniformis |
| 18040 | 96_8336 | 13 | 8 | Bacteroidetes | Bacteroidia | Bacteroidales | Bacteroidaceae | Bacteroides | Bacteroides sp. |
| 18041 | 60_1364 | 30 | 13 | Bacteroidetes | Bacteroidia | Bacteroidales | Bacteroidaceae | Bacteroides | Bacteroides sp. |
| 18046 | 124_5297 | 3615 | 66 | Bacteroidetes | Bacteroidia | Bacteroidales | Bacteroidaceae | Bacteroides | Bacteroides massiliensis |
| 18047 | 1_2047 | 1660 | 71 | Bacteroidetes | Bacteroidia | Bacteroidales | Bacteroidaceae | Bacteroides | Bacteroides massiliensis |
| 18051 | 121_944 | 28 | 15 | Bacteroidetes | Bacteroidia | Bacteroidales | Prevotellaceae | Prevotella | Prevotella copri |
| 18053 | 124_6773 | 111 | 9 | Bacteroidetes | Bacteroidia | Bacteroidales | Bacteroidaceae | Bacteroides | Bacteroides massiliensis |
| 18075 | 71_3474 | 392 | 36 | Bacteroidetes | Bacteroidia | Bacteroidales | Bacteroidaceae | Bacteroides | Bacteroides massiliensis |
| 18090 | 124_7327 | 13 | 11 | Bacteroidetes | Bacteroidia | Bacteroidales | Bacteroidaceae | Bacteroides | Bacteroides massiliensis |
| 18105 | 63_6487 | 367 | 17 | Bacteroidetes | Bacteroidia | Bacteroidales | Bacteroidaceae | Bacteroides | Bacteroides uniformis |
| 18107 | 20_3227 | 20 | 5 | Bacteroidetes | Bacteroidia | Bacteroidales | Bacteroidaceae | Bacteroides | Bacteroides eggerthii |
| 18109 | 23_423 | 25 | 10 | Bacteroidetes | Bacteroidia | Bacteroidales | Bacteroidaceae | Bacteroides | Bacteroides eggerthii |
| 18115 | 131_1410 | 18 | 9 | Bacteroidetes | Bacteroidia | Bacteroidales | Bacteroidaceae | Bacteroides | Bacteroides eggerthii |
| 18118 | 39_6353 | 35 | 12 | Bacteroidetes | Bacteroidia | Bacteroidales | Bacteroidaceae | Bacteroides | Bacteroides eggerthii |
| 18119 | 20_3367 | 19 | 11 | Bacteroidetes | Bacteroidia | Bacteroidales | Bacteroidaceae | Bacteroides | Bacteroides eggerthii |
| 18124 | 63_6104 | 92 | 14 | Bacteroidetes | Bacteroidia | Bacteroidales | Bacteroidaceae | Bacteroides | Bacteroides eggerthii |
| 18132 | 130_7063 | 6 | 6 | Bacteroidetes | Bacteroidia | Bacteroidales | Bacteroidaceae | Bacteroides | Bacteroides eggerthii |
| 18136 | 63_6352 | 11 | 6 | Bacteroidetes | Bacteroidia | Bacteroidales | Bacteroidaceae | Bacteroides | Bacteroides uniformis |
| 18151 | 131_3507 | 41 | 10 | Bacteroidetes | Bacteroidia | Bacteroidales | Bacteroidaceae | Bacteroides | Bacteroides eggerthii |
| 18165 | 105_1518 | 115 | 8 | Bacteroidetes | Bacteroidia | Bacteroidales | Bacteroidaceae | Bacteroides | Bacteroides massiliensis |
| 18173 | 75_366 | 8 | 6 | Bacteroidetes | Bacteroidia | Bacteroidales | Bacteroidaceae | Bacteroides | Bacteroides uniformis |
| 18175 | 101_323 | 24 | 7 | Bacteroidetes | Bacteroidia | Bacteroidales | Bacteroidaceae | Bacteroides | Bacteroides sp. |
| 18190 | 63_6498 | 117 | 25 | Bacteroidetes | Bacteroidia | Bacteroidales | Bacteroidaceae | Bacteroides | Bacteroides uniformis |
| 18193 | 119_386 | 9 | 8 | Bacteroidetes | Bacteroidia | Bacteroidales | Porphyromonadaceae | Parabacteroides | Parabacteroides merdae |
| 18195 | 108_2397 | 34 | 1 | Bacteroidetes | Bacteroidia | Bacteroidales | Bacteroidaceae | Bacteroides | Bacteroides finegoldii |
| 18197 | 97_1119 | 10 | 5 | Bacteroidetes | Bacteroidia | Bacteroidales | Bacteroidaceae | Bacteroides | Bacteroides finegoldii |
| 18200 | 75_770 | 12 | 6 | Bacteroidetes | Bacteroidia | Bacteroidales | Bacteroidaceae | Bacteroides | Bacteroides finegoldii |
| 18216 | 24_7126 | 9 | 6 | Bacteroidetes | Bacteroidia | Bacteroidales | Bacteroidaceae | Bacteroides | Bacteroides sp. |
| 18232 | 42_6967 | 29 | 7 | Bacteroidetes | Bacteroidia | Bacteroidales | Bacteroidaceae | Bacteroides | Bacteroides fragilis |
| 18237 | 68_11671 | 32 | 11 | Bacteroidetes | Bacteroidia | Bacteroidales | Bacteroidaceae | Bacteroides | Bacteroides sp. |
| 18239 | 115_3602 | 18 | 6 | Bacteroidetes | Bacteroidia | Bacteroidales | Prevotellaceae | Prevotella | Prevotella copri |
| 18240 | 131_9068 | 16 | 7 | Bacteroidetes | Bacteroidia | Bacteroidales | Bacteroidaceae | Bacteroides | Bacteroides uniformis |
| 18241 | 99_6609 | 16 | 5 | Bacteroidetes | Bacteroidia | Bacteroidales | Bacteroidaceae | Bacteroides | Bacteroides uniformis |
| 18252 | 65_6963 | 24 | 7 | Bacteroidetes | Bacteroidia | Bacteroidales | Bacteroidaceae | Bacteroides | Bacteroides uniformis |
| 18259 | 42_2520 | 9 | 6 | Bacteroidetes | Bacteroidia | Bacteroidales | Bacteroidaceae | Bacteroides | Bacteroides uniformis |
| 18262 | 35_5042 | 11 | 8 | Bacteroidetes | Bacteroidia | Bacteroidales | Bacteroidaceae | Bacteroides | Bacteroides uniformis |
| 18286 | 96_9822 | 9 | 5 | Bacteroidetes | Bacteroidia | Bacteroidales | Bacteroidaceae | Bacteroides | Bacteroides massiliensis |
| 18296 | 1_1396 | 13 | 10 | Bacteroidetes | Bacteroidia | Bacteroidales | Prevotellaceae | Prevotella | Prevotella copri |
| 18317 | 63_1222 | 14 | 6 | Bacteroidetes | Bacteroidia | Bacteroidales | Bacteroidaceae | Bacteroides | Bacteroides eggerthii |
| 18319 | 40_2138 | 27 | 10 | Bacteroidetes | Bacteroidia | Bacteroidales | Bacteroidaceae | Bacteroides | Bacteroides uniformis |
| 18321 | 90_10107 | 16 | 6 | Bacteroidetes | Bacteroidia | Bacteroidales | Bacteroidaceae | Bacteroides | Bacteroides uniformis |
| 18323 | 91_8657 | 14 | 11 | Bacteroidetes | Bacteroidia | Bacteroidales | Porphyromonadaceae | Barnesiella | Barnesiella intestinihominis |
| 18328 | 90_5061 | 25 | 5 | Bacteroidetes | Bacteroidia | Bacteroidales | Bacteroidaceae | Bacteroides | Bacteroides uniformis |
| 18335 | 63_3467 | 12 | 7 | Bacteroidetes | Bacteroidia | Bacteroidales | Bacteroidaceae | Bacteroides | Bacteroides thetaiotaomicron |
| 18343 | 2_6722 | 9 | 6 | Bacteroidetes | Bacteroidia | Bacteroidales | Bacteroidaceae | Bacteroides | Bacteroides uniformis |
| 18352 | 113_4983 | 12 | 5 | Bacteroidetes | Bacteroidia | Bacteroidales | Bacteroidaceae | Bacteroides | Bacteroides uniformis |
| 18356 | 63_5474 | 12 | 10 | Bacteroidetes | Bacteroidia | Bacteroidales | Bacteroidaceae | Bacteroides | Bacteroides finegoldii |
| 18361 | 91_5197 | 14 | 9 | Bacteroidetes | Bacteroidia | Bacteroidales | Bacteroidaceae | Bacteroides | Bacteroides thetaiotaomicron |
| 18369 | 10_5462 | 110 | 13 | Bacteroidetes | Bacteroidia | Bacteroidales | Bacteroidaceae | Bacteroides | Bacteroides uniformis |
| 18370 | 90_8037 | 142 | 29 | Bacteroidetes | Bacteroidia | Bacteroidales | Bacteroidaceae | Bacteroides | Bacteroides uniformis |
| 18372 | 90_10200 | 223 | 15 | Bacteroidetes | Bacteroidia | Bacteroidales | Bacteroidaceae | Bacteroides | Bacteroides massiliensis |
| 18375 | 1_51 | 12 | 9 | Bacteroidetes | Bacteroidia | Bacteroidales | Prevotellaceae | Prevotella | Prevotella copri |
| 18378 | 40_3421 | 11 | 7 | Bacteroidetes | Bacteroidia | Bacteroidales | Bacteroidaceae | Bacteroides | Bacteroides uniformis |
| 18379 | 122_4304 | 15 | 8 | Bacteroidetes | Bacteroidia | Bacteroidales | Bacteroidaceae | Bacteroides | Bacteroides uniformis |
| 18395 | 63_1823 | 10 | 7 | Bacteroidetes | Bacteroidia | Bacteroidales | Bacteroidaceae | Bacteroides | Bacteroides uniformis |
| 18407 | 23_3624 | 8 | 5 | Bacteroidetes | Bacteroidia | Bacteroidales | Prevotellaceae | Prevotella | Prevotella copri |
| 18409 | 123_3894 | 87 | 13 | Bacteroidetes | Bacteroidia | Bacteroidales | Bacteroidaceae | Bacteroides | Bacteroides uniformis |
| 18439 | 68_12055 | 18 | 8 | Bacteroidetes | Bacteroidia | Bacteroidales | Bacteroidaceae | Bacteroides | Bacteroides ovatus |
| 18442 | 43_3129 | 5 | 5 | Bacteroidetes | Bacteroidia | Bacteroidales | Bacteroidaceae | Bacteroides | Bacteroides sp. |
| 18452 | 107_1849 | 32 | 5 | Bacteroidetes | Bacteroidia | Bacteroidales | Bacteroidaceae | Bacteroides | Bacteroides sp. |
| 18454 | 105_7116 | 11 | 7 | Bacteroidetes | Bacteroidia | Bacteroidales | Bacteroidaceae | Bacteroides | Bacteroides finegoldii |
| 18455 | 126_383 | 44 | 13 | Bacteroidetes | Bacteroidia | Bacteroidales | Bacteroidaceae | Bacteroides | Bacteroides sp. |
| 18464 | 18_2766 | 24 | 11 | Bacteroidetes | Bacteroidia | Bacteroidales | Bacteroidaceae | Bacteroides | Bacteroides sp. |
| 18476 | 24_7024 | 5 | 5 | Bacteroidetes | Bacteroidia | Bacteroidales | Bacteroidaceae | Bacteroides | Bacteroides caccae |
| 18479 | 4_2730 | 7 | 7 | Bacteroidetes | Bacteroidia | Bacteroidales | Bacteroidaceae | Bacteroides | Bacteroides finegoldii |
| 18482 | 119_3172 | 9 | 7 | Bacteroidetes | Bacteroidia | Bacteroidales | Bacteroidaceae | Bacteroides | Bacteroides uniformis |
| 18533 | 126_949 | 73 | 10 | Bacteroidetes | Bacteroidia | Bacteroidales | Bacteroidaceae | Bacteroides | Bacteroides massiliensis |
| 18572 | 131_4736 | 11 | 6 | Bacteroidetes | Bacteroidia | Bacteroidales | Bacteroidaceae | Bacteroides | Bacteroides uniformis |
| 18575 | 130_7510 | 5 | 5 | Bacteroidetes | Bacteroidia | Bacteroidales | Bacteroidaceae | Bacteroides | Bacteroides uniformis |
| 18596 | 123_1363 | 7 | 5 | Bacteroidetes | Bacteroidia | Bacteroidales | Prevotellaceae | Prevotella | Prevotella copri |
| 18627 | 39_5668 | 40 | 4 | Bacteroidetes | Bacteroidia | Bacteroidales | Bacteroidaceae | Bacteroides | Bacteroides eggerthii |
| 18655 | 120_1103 | 12 | 9 | Bacteroidetes | Bacteroidia | Bacteroidales | Porphyromonadaceae | Parabacteroides | Parabacteroides distasonis |
| 18657 | 63_1369 | 45 | 17 | Bacteroidetes | Bacteroidia | Bacteroidales | Porphyromonadaceae | Parabacteroides | Parabacteroides merdae |
| 18672 | 19_7168 | 6 | 6 | Bacteroidetes | Bacteroidia | Bacteroidales | Bacteroidaceae | Bacteroides | Bacteroides massiliensis |
| 18677 | 6_3807 | 8 | 7 | Bacteroidetes | Bacteroidia | Bacteroidales | Prevotellaceae | Prevotella | Prevotella copri |
| 18690 | 30_9569 | 187 | 21 | Bacteroidetes | Bacteroidia | Bacteroidales | Porphyromonadaceae | Parabacteroides | Parabacteroides distasonis |
| 18709 | 7_2465 | 20 | 13 | Bacteroidetes | Bacteroidia | Bacteroidales | Bacteroidaceae | Bacteroides | Bacteroides uniformis |
| 18722 | 100_978 | 19 | 5 | Bacteroidetes | Bacteroidia | Bacteroidales | Bacteroidaceae | Bacteroides | Bacteroides ovatus |
| 18746 | 120_639 | 1258 | 63 | Bacteroidetes | Bacteroidia | Bacteroidales | Porphyromonadaceae | Parabacteroides | Parabacteroides distasonis |
| 18749 | 50_1147 | 10 | 7 | Bacteroidetes | Bacteroidia | Bacteroidales | Bacteroidaceae | Bacteroides | Bacteroides massiliensis |
| 18766 | 4_1271 | 13 | 12 | Bacteroidetes | Bacteroidia | Bacteroidales | Bacteroidaceae | Bacteroides | Bacteroides massiliensis |
| 18770 | 60_6908 | 13 | 7 | Bacteroidetes | Bacteroidia | Bacteroidales | Bacteroidaceae | Bacteroides | Bacteroides uniformis |
| 18780 | 22_1315 | 6 | 5 | Bacteroidetes | Bacteroidia | Bacteroidales | Porphyromonadaceae | Barnesiella | Barnsiella intestinihominis |
| 18784 | 104_968 | 18 | 14 | Bacteroidetes | Bacteroidia | Bacteroidales | Prevotellaceae | Prevotella | Prevotella copri |
| 18791 | 41_4470 | 7 | 5 | Bacteroidetes | Bacteroidia | Bacteroidales | Bacteroidaceae | Bacteroides | Bacteroides eggerthii |
| 18796 | 108_4792 | 22 | 6 | Bacteroidetes | Bacteroidia | Bacteroidales | Bacteroidaceae | Bacteroides | Bacteroides thetaiotaomicron |
| 18800 | 63_2642 | 28 | 18 | Bacteroidetes | Bacteroidia | Bacteroidales | Bacteroidaceae | Bacteroides | Bacteroides uniformis |
| 18803 | 104_152 | 8 | 5 | Bacteroidetes | Bacteroidia | Bacteroidales | Prevotellaceae | Prevotella | Prevotella copri |
| 18831 | 122_2593 | 24 | 12 | Bacteroidetes | Bacteroidia | Bacteroidales | Porphyromonadaceae | Barnesiella | Barnsiella intestinihominis |
| 18833 | 122_1374 | 11 | 7 | Bacteroidetes | Bacteroidia | Bacteroidales | Bacteroidaceae | Bacteroides | Bacteroides thetaiotaomicron |
| 18848 | 63_5364 | 70 | 24 | Bacteroidetes | Bacteroidia | Bacteroidales | Bacteroidaceae | Bacteroides | Bacteroides caccae |
| 18855 | 107_2239 | 243 | 33 | Bacteroidetes | Bacteroidia | Bacteroidales | Bacteroidaceae | Bacteroides | Bacteroides vulgatus |
| 18869 | 112_3787 | 8 | 5 | Bacteroidetes | Bacteroidia | Bacteroidales | Prevotellaceae | Prevotella | Prevotella copri |
| 18872 | 33_4608 | 7 | 5 | Bacteroidetes | Bacteroidia | Bacteroidales | Prevotellaceae | Prevotella | Prevotella copri |
| 18876 | 4_549 | 24 | 13 | Bacteroidetes | Bacteroidia | Bacteroidales | Prevotellaceae | Prevotella | Prevotella copri |
| 18884 | 86_1571 | 6 | 5 | Bacteroidetes | Bacteroidia | Bacteroidales | Bacteroidaceae | Bacteroides | Bacteroides xylanisolvens |
| 18885 | 30_5116 | 22 | 10 | Bacteroidetes | Bacteroidia | Bacteroidales | Bacteroidaceae | Bacteroides | Bacteroides uniformis |
| 18890 | 20_8737 | 37 | 8 | Bacteroidetes | Bacteroidia | Bacteroidales | Bacteroidaceae | Bacteroides | Bacteroides vulgatus |
| 18896 | 16_2575 | 11 | 5 | Bacteroidetes | Bacteroidia | Bacteroidales | Bacteroidaceae | Bacteroides | Bacteroides vulgatus |
| 18906 | 7_1760 | 6 | 6 | Bacteroidetes | Bacteroidia | Bacteroidales | Bacteroidaceae | Bacteroides | Bacteroides caccae |
| 18917 | 7_5422 | 12 | 8 | Bacteroidetes | Bacteroidia | Bacteroidales | Bacteroidaceae | Bacteroides | Bacteroides caccae |
| 18918 | 3_359 | 12 | 7 | Bacteroidetes | Bacteroidia | Bacteroidales | Bacteroidaceae | Bacteroides | Bacteroides ovatus |
| 18924 | 65_3623 | 9630 | 86 | Bacteroidetes | Bacteroidia | Bacteroidales | Bacteroidaceae | Bacteroides | Bacteroides vulgatus |
| 18932 | 130_1777 | 5 | 5 | Bacteroidetes | Bacteroidia | Bacteroidales | Bacteroidaceae | Bacteroides | Bacteroides vulgatus |
| 18938 | 127_2871 | 6 | 5 | Bacteroidetes | Bacteroidia | Bacteroidales | Bacteroidaceae | Bacteroides | Bacteroides uniformis |
| 18969 | 76_1674 | 5 | 5 | Firmicutes | Clostridia | Clostridiales | Lachnospiraceae | Lachnospiraceae incertae sedis | Lachnospiraceae incertae sedis |
| 18979 | 130_3623 | 13 | 7 | Bacteroidetes | Bacteroidia | Bacteroidales | Bacteroidaceae | Bacteroides | Bacteroides uniformis |
| 18992 | 20_8610 | 80 | 14 | Bacteroidetes | Bacteroidia | Bacteroidales | Bacteroidaceae | Bacteroides | Bacteroides eggerthii |
| 18997 | 130_5317 | 21 | 8 | Bacteroidetes | Bacteroidia | Bacteroidales | Bacteroidaceae | Bacteroides | Bacteroides finegoldii |
| 18999 | 108_3240 | 26 | 5 | Bacteroidetes | Bacteroidia | Bacteroidales | Prevotellaceae | Prevotella | Prevotella copri |
| 19026 | 66_133 | 6 | 5 | Bacteroidetes | Bacteroidia | Bacteroidales | Prevotellaceae | Prevotella | Prevotella copri |
| 19040 | 7_4992 | 5 | 5 | Bacteroidetes | Bacteroidia | Bacteroidales | Porphyromonadaceae | Parabacteroides | Parabacteroides merdae |
| 19042 | 53_5784 | 57 | 6 | Bacteroidetes | Bacteroidia | Bacteroidales | Bacteroidaceae | Bacteroides | Bacteroides fragilis |
| 19046 | 65_5351 | 122 | 28 | Bacteroidetes | Bacteroidia | Bacteroidales | Bacteroidaceae | Bacteroides | Bacteroides vulgatus |
| 19050 | 76_5406 | 20 | 5 | Bacteroidetes | Bacteroidia | Bacteroidales | Bacteroidaceae | Bacteroides | Bacteroides fragilis |
| 19051 | 42_6658 | 35 | 15 | Bacteroidetes | Bacteroidia | Bacteroidales | Bacteroidaceae | Bacteroides | Bacteroides uniformis |
| 19052 | 123_2908 | 8 | 5 | Bacteroidetes | Bacteroidia | Bacteroidales | Prevotellaceae | Prevotella | Prevotella copri |
| 19053 | 12_1096 | 11 | 5 | Bacteroidetes | Bacteroidia | Bacteroidales | Bacteroidaceae | Bacteroides | Bacteroides uniformis |
| 19070 | 15_2612 | 9 | 6 | Bacteroidetes | Bacteroidia | Bacteroidales | Bacteroidaceae | Bacteroides | Bacteroides ovatus |
| 19080 | 123_6960 | 28 | 7 | Bacteroidetes | Bacteroidia | Bacteroidales | Bacteroidaceae | Bacteroides | Bacteroides vulgatus |
| 19083 | 12_2456 | 12 | 8 | Bacteroidetes | Bacteroidia | Bacteroidales | Bacteroidaceae | Bacteroides | Bacteroides uniformis |
| 19085 | 60_5443 | 17 | 8 | Bacteroidetes | Bacteroidia | Bacteroidales | Porphyromonadaceae | Parabacteroides | Parabacteroides merdae |
| 19093 | 19_1791 | 7 | 6 | Bacteroidetes | Bacteroidia | Bacteroidales | Bacteroidaceae | Bacteroides | Bacteroides xylanisolvens |
| 19224 | 70_3115 | 20 | 2 | Bacteroidetes | Bacteroidia | Bacteroidales | Prevotellaceae | Prevotella | Prevotella sp. |
| 19235 | 81_6834 | 37 | 5 | Bacteroidetes | Bacteroidia | Bacteroidales | Prevotellaceae | Prevotella | Prevotella sp. |
| 19278 | 20_1387 | 34 | 4 | Bacteroidetes | Bacteroidia | Bacteroidales | Bacteroidaceae | Bacteroides | Bacteroides eggerthii |
| 19310 | 29_4542 | 21 | 3 | Bacteroidetes | Bacteroidia | Bacteroidales | Bacteroidaceae | Bacteroides | Bacteroides eggerthii |
| 19315 | 39_5635 | 19 | 5 | Bacteroidetes | Bacteroidia | Bacteroidales | Prevotellaceae | Prevotella | Prevotella sp. |
| 19336 | 26_1426 | 43 | 7 | Bacteroidetes | Bacteroidia | Bacteroidales | Prevotellaceae | Prevotella | Prevotella copri |
| 19351 | 47_5668 | 30 | 7 | Bacteroidetes | Bacteroidia | Bacteroidales | Prevotellaceae | Prevotella | Prevotella ruminicola |
| 19360 | 1_1199 | 178 | 12 | Bacteroidetes | Bacteroidia | Bacteroidales | Prevotellaceae | Prevotella | Prevotella copri |
| 19368 | 104_507 | 7 | 6 | Bacteroidetes | Bacteroidia | Bacteroidales | Prevotellaceae | Prevotella | Prevotella copri |
| 19403 | 129_5529 | 103 | 5 | Bacteroidetes | Bacteroidia | Bacteroidales | Prevotellaceae | Prevotella | Prevotella copri |
| 19449 | 81_755 | 32 | 8 | Bacteroidetes | Bacteroidia | Bacteroidales | Prevotellaceae | Prevotella | Prevotella copri |
| 19460 | 25_2293 | 11 | 7 | Bacteroidetes | Bacteroidia | Bacteroidales | Prevotellaceae | Prevotella | Prevotella copri |
| 19479 | 104_1034 | 44 | 9 | Bacteroidetes | Bacteroidia | Bacteroidales | Prevotellaceae | Prevotella | Prevotella copri |
| 19481 | 104_134 | 111 | 11 | Bacteroidetes | Bacteroidia | Bacteroidales | Prevotellaceae | Prevotella | Prevotella copri |
| 19484 | 54_4855 | 6 | 5 | Bacteroidetes | Bacteroidia | Bacteroidales | Prevotellaceae | Prevotella | Prevotella copri |
| 19494 | 21_3312 | 10 | 5 | Bacteroidetes | Bacteroidia | Bacteroidales | Prevotellaceae | Prevotella | Prevotella copri |
| 19498 | 123_887 | 65 | 17 | Bacteroidetes | Bacteroidia | Bacteroidales | Prevotellaceae | Prevotella | Prevotella copri |
| 19523 | 123_3692 | 10 | 5 | Bacteroidetes | Bacteroidia | Bacteroidales | Prevotellaceae | Prevotella | Prevotella copri |
| 19524 | 104_131 | 25 | 7 | Bacteroidetes | Bacteroidia | Bacteroidales | Prevotellaceae | Prevotella | Prevotella copri |
| 19527 | 121_6217 | 45 | 7 | Bacteroidetes | Bacteroidia | Bacteroidales | Prevotellaceae | Prevotella | Prevotella copri |
| 19539 | 25_1356 | 10 | 7 | Bacteroidetes | Bacteroidia | Bacteroidales | Bacteroidaceae | Bacteroides | Bacteroides xylanisolvens |
| 19541 | 125_588 | 32 | 8 | Bacteroidetes | Bacteroidia | Bacteroidales | Prevotellaceae | Prevotella | Prevotella copri |
| 19546 | 125_5642 | 64 | 9 | Bacteroidetes | Bacteroidia | Bacteroidales | Prevotellaceae | Prevotella | Prevotella copri |
| 19547 | 35_161 | 8 | 6 | Bacteroidetes | Bacteroidia | Bacteroidales | Prevotellaceae | Prevotella | Prevotella copri |
| 19548 | 100_2993 | 8 | 5 | Bacteroidetes | Bacteroidia | Bacteroidales | Prevotellaceae | Prevotella | Prevotella copri |
| 19553 | 1_3196 | 30 | 6 | Bacteroidetes | Bacteroidia | Bacteroidales | Prevotellaceae | Prevotella | Prevotella sp. |
| 19554 | 123_7643 | 9 | 6 | Bacteroidetes | Bacteroidia | Bacteroidales | Porphyromonadaceae | Barnesiella | Barnsiella intestinihominis |
| 19560 | 121_6397 | 21 | 4 | Bacteroidetes | Bacteroidia | Bacteroidales | Prevotellaceae | Prevotella | Prevotella copri |
| 19561 | 112_6250 | 23 | 9 | Bacteroidetes | Bacteroidia | Bacteroidales | Prevotellaceae | Prevotella | Prevotella copri |
| 19565 | 33_4498 | 19 | 5 | Bacteroidetes | Bacteroidia | Bacteroidales | Prevotellaceae | Prevotella | Prevotella copri |
| 19566 | 31_1611 | 9 | 6 | Bacteroidetes | Bacteroidia | Bacteroidales | Prevotellaceae | Prevotella | Prevotella copri |
| 19567 | 104_732 | 78 | 13 | Bacteroidetes | Bacteroidia | Bacteroidales | Prevotellaceae | Prevotella | Prevotella copri |
| 19571 | 112_5280 | 23 | 4 | Bacteroidetes | Bacteroidia | Bacteroidales | Prevotellaceae | Prevotella | Prevotella copri |
| 19583 | 121_4066 | 11 | 5 | Bacteroidetes | Bacteroidia | Bacteroidales | Prevotellaceae | Prevotella | Prevotella copri |
| 19584 | 13_2517 | 9 | 5 | Bacteroidetes | Bacteroidia | Bacteroidales | Prevotellaceae | Prevotella | Prevotella copri |
| 19588 | 104_610 | 33 | 10 | Bacteroidetes | Bacteroidia | Bacteroidales | Prevotellaceae | Prevotella | Prevotella copri |
| 19591 | 23_2890 | 7 | 5 | Bacteroidetes | Bacteroidia | Bacteroidales | Bacteroidaceae | Bacteroides | Bacteroides thetaiotaomicron |
| 19594 | 112_1859 | 14 | 6 | Bacteroidetes | Bacteroidia | Bacteroidales | Prevotellaceae | Prevotella | Prevotella copri |
| 19596 | 24_1388 | 6 | 5 | Bacteroidetes | Bacteroidia | Bacteroidales | Prevotellaceae | Prevotella | Prevotella copri |
| 19597 | 123_3846 | 8 | 5 | Bacteroidetes | Bacteroidia | Bacteroidales | Prevotellaceae | Prevotella | Prevotella copri |
| 19603 | 4_2979 | 11 | 7 | Bacteroidetes | Bacteroidia | Bacteroidales | Prevotellaceae | Prevotella | Prevotella copri |
| 19606 | 4_1614 | 6 | 6 | Bacteroidetes | Bacteroidia | Bacteroidales | Prevotellaceae | Prevotella | Prevotella copri |
| 19609 | 121_6057 | 19 | 7 | Bacteroidetes | Bacteroidia | Bacteroidales | Prevotellaceae | Prevotella | Prevotella copri |
| 19614 | 123_6951 | 61 | 13 | Bacteroidetes | Bacteroidia | Bacteroidales | Prevotellaceae | Prevotella | Prevotella copri |
| 19624 | 24_1167 | 110 | 6 | Bacteroidetes | Bacteroidia | Bacteroidales | Prevotellaceae | Prevotella | Prevotella copri |
| 19625 | 123_4326 | 110 | 13 | Bacteroidetes | Bacteroidia | Bacteroidales | Prevotellaceae | Prevotella | Prevotella copri |
| 19627 | 1_837 | 18 | 5 | Bacteroidetes | Bacteroidia | Bacteroidales | Prevotellaceae | Prevotella | Prevotella copri |
| 19634 | 43_1894 | 5 | 5 | Bacteroidetes | Bacteroidia | Bacteroidales | Bacteroidaceae | Bacteroides | Bacteroides uniformis |
| 19653 | 104_1011 | 12 | 6 | Bacteroidetes | Bacteroidia | Bacteroidales | Prevotellaceae | Prevotella | Prevotella copri |
| 19654 | 23_3867 | 45 | 5 | Bacteroidetes | Bacteroidia | Bacteroidales | Prevotellaceae | Prevotella | Prevotella copri |
| 19661 | 125_1057 | 10 | 5 | Bacteroidetes | Bacteroidia | Bacteroidales | Prevotellaceae | Prevotella | Prevotella copri |
| 19665 | 23_799 | 16 | 5 | Bacteroidetes | Bacteroidia | Bacteroidales | Prevotellaceae | Prevotella | Prevotella copri |
| 19668 | 33_5490 | 55 | 11 | Bacteroidetes | Bacteroidia | Bacteroidales | Prevotellaceae | Prevotella | Prevotella copri |
| 19694 | 54_3400 | 8 | 5 | Bacteroidetes | Bacteroidia | Bacteroidales | Prevotellaceae | Prevotella | Prevotella copri |
| 19696 | 81_3817 | 84 | 10 | Bacteroidetes | Bacteroidia | Bacteroidales | Prevotellaceae | Prevotella | Prevotella copri |
| 19700 | 43_3037 | 7 | 5 | Bacteroidetes | Bacteroidia | Bacteroidales | Prevotellaceae | Prevotella | Prevotella copri |
| 19704 | 121_5538 | 13 | 5 | Bacteroidetes | Bacteroidia | Bacteroidales | Prevotellaceae | Prevotella | Prevotella copri |
| 19718 | 104_91 | 6 | 5 | Bacteroidetes | Bacteroidia | Bacteroidales | Prevotellaceae | Prevotella | Prevotella copri |
| 19727 | 1_107 | 35 | 6 | Bacteroidetes | Bacteroidia | Bacteroidales | Prevotellaceae | Prevotella | Prevotella sp. |
| 19742 | 81_6435 | 23 | 7 | Bacteroidetes | Bacteroidia | Bacteroidales | Prevotellaceae | Prevotella | Prevotella stercorea |
| 19743 | 43_2009 | 9 | 5 | Bacteroidetes | Bacteroidia | Bacteroidales | Prevotellaceae | Prevotella | Prevotella copri |
| 19768 | 104_911 | 23 | 8 | Bacteroidetes | Bacteroidia | Bacteroidales | Prevotellaceae | Prevotella | Prevotella copri |
| 19795 | 121_3262 | 9 | 6 | Bacteroidetes | Bacteroidia | Bacteroidales | Prevotellaceae | Prevotella | Prevotella copri |
| 19806 | 121_6377 | 11 | 5 | Bacteroidetes | Bacteroidia | Bacteroidales | Prevotellaceae | Prevotella | Prevotella copri |
| 19809 | 1_2216 | 5 | 5 | Bacteroidetes | Bacteroidia | Bacteroidales | Prevotellaceae | Prevotella | Prevotella copri |
| 19824 | 1_2739 | 14 | 6 | Bacteroidetes | Bacteroidia | Bacteroidales | Prevotellaceae | Prevotella | Prevotella copri |
| 19834 | 112_5715 | 41 | 8 | Bacteroidetes | Bacteroidia | Bacteroidales | Prevotellaceae | Prevotella | Prevotella copri |
| 19838 | 121_4481 | 54 | 8 | Bacteroidetes | Bacteroidia | Bacteroidales | Prevotellaceae | Prevotella | Prevotella copri |
| 19843 | 112_2901 | 44 | 11 | Bacteroidetes | Bacteroidia | Bacteroidales | Prevotellaceae | Prevotella | Prevotella copri |
| 19845 | 21_6073 | 14 | 5 | Bacteroidetes | Bacteroidia | Bacteroidales | Prevotellaceae | Prevotella | Prevotella copri |
| 19852 | 104_339 | 39 | 5 | Bacteroidetes | Bacteroidia | Bacteroidales | Prevotellaceae | Prevotella | Prevotella copri |
| 19854 | 25_270 | 28 | 8 | Bacteroidetes | Bacteroidia | Bacteroidales | Prevotellaceae | Prevotella | Prevotella copri |
| 19867 | 66_3940 | 12 | 7 | Bacteroidetes | Bacteroidia | Bacteroidales | Prevotellaceae | Prevotella | Prevotella copri |
| 19869 | 121_2663 | 17 | 7 | Bacteroidetes | Bacteroidia | Bacteroidales | Prevotellaceae | Prevotella | Prevotella copri |
| 19871 | 112_5517 | 19 | 9 | Bacteroidetes | Bacteroidia | Bacteroidales | Prevotellaceae | Prevotella | Prevotella copri |
| 19872 | 121_3923 | 13 | 8 | Bacteroidetes | Bacteroidia | Bacteroidales | Prevotellaceae | Prevotella | Prevotella copri |
| 19874 | 104_630 | 8 | 5 | Bacteroidetes | Bacteroidia | Bacteroidales | Prevotellaceae | Prevotella | Prevotella copri |
| 19878 | 112_5029 | 29 | 10 | Bacteroidetes | Bacteroidia | Bacteroidales | Prevotellaceae | Prevotella | Prevotella copri |
| 19910 | 107_990 | 86 | 9 | Bacteroidetes | Bacteroidia | Bacteroidales | Prevotellaceae | Prevotella | Prevotella copri |
| 19920 | 121_3014 | 5 | 5 | Bacteroidetes | Bacteroidia | Bacteroidales | Prevotellaceae | Prevotella | Prevotella stercorea |
| 19928 | 123_7110 | 15 | 5 | Bacteroidetes | Bacteroidia | Bacteroidales | Prevotellaceae | Prevotella | Prevotella copri |
| 19932 | 112_2185 | 28 | 7 | Bacteroidetes | Bacteroidia | Bacteroidales | Prevotellaceae | Prevotella | Prevotella copri |
| 19938 | 123_3056 | 30 | 6 | Bacteroidetes | Bacteroidia | Bacteroidales | Prevotellaceae | Prevotella | Prevotella copri |
| 19944 | 112_6147 | 25 | 8 | Bacteroidetes | Bacteroidia | Bacteroidales | Prevotellaceae | Prevotella | Prevotella copri |
| 19962 | 26_5503 | 17 | 9 | Bacteroidetes | Bacteroidia | Bacteroidales | Bacteroidaceae | Bacteroides | Bacteroides sp. |
| 19968 | 71_6161 | 142 | 6 | Bacteroidetes | Bacteroidia | Bacteroidales | Prevotellaceae | Prevotella | Prevotella copri |
| 19994 | 123_5023 | 12 | 8 | Bacteroidetes | Bacteroidia | Bacteroidales | Porphyromonadaceae | Barnesiella | Barnsiella intestinihominis |
| 20000 | 81_6957 | 8 | 5 | Bacteroidetes | Bacteroidia | Bacteroidales | Prevotellaceae | Prevotella | Prevotella copri |
| 20023 | 69_4511 | 117 | 8 | Bacteroidetes | Bacteroidia | Bacteroidales | Prevotellaceae | Paraprevotella | Paraprevotella xylaniphila |
| 20053 | 112_6057 | 9 | 5 | Bacteroidetes | Bacteroidia | Bacteroidales | Prevotellaceae | Prevotella | Prevotella copri |
| 20093 | 119_2959 | 39 | 5 | Bacteroidetes | Bacteroidia | Bacteroidales | Bacteroidaceae | Bacteroides | Bacteroides massiliensis |
| 20100 | 119_5762 | 7 | 5 | Bacteroidetes | Bacteroidia | Bacteroidales | Prevotellaceae | Paraprevotella | Paraprevotella clara |
| 20108 | 77_8303 | 11 | 5 | Bacteroidetes | Bacteroidia | Bacteroidales | Bacteroidaceae | Bacteroides | Bacteroides ovatus |
| 20118 | 50_370 | 6 | 5 | Bacteroidetes | Bacteroidia | Bacteroidales | Porphyromonadaceae | Parabacteroides | Parabacteroides merdae |
| 20164 | 100_10773 | 3836 | 8 | Verrucomicrobia | Verrucomicrobiae | Verrucomicrobiales | Akkermansiaceae | Akkermansia | Akkermansia muciniphila |
| 20174 | 96_2817 | 9 | 5 | Bacteroidetes | Bacteroidia | Bacteroidales | Bacteroidaceae | Bacteroides | Bacteroides sp. |
| 20204 | 91_258 | 7 | 5 | Bacteroidetes | Bacteroidia | Bacteroidales | Bacteroidaceae | Bacteroides | Bacteroides ovatus |
| 20205 | 104_1134 | 41 | 9 | Bacteroidetes | Bacteroidia | Bacteroidales | Prevotellaceae | Prevotella | Prevotella copri |
| 20228 | 112_1711 | 13 | 8 | Bacteroidetes | Bacteroidia | Bacteroidales | Prevotellaceae | Prevotella | Prevotella copri |
| 20232 | 108_5042 | 13 | 5 | Bacteroidetes | Bacteroidia | Bacteroidales | Prevotellaceae | Prevotella | Prevotella stercorea |
| 20275 | 1_1934 | 13 | 6 | Bacteroidetes | Bacteroidia | Bacteroidales | Prevotellaceae | Prevotella | Prevotella copri |
| 20283 | 37_6291 | 11 | 5 | Bacteroidetes | Bacteroidia | Bacteroidales | Bacteroidaceae | Bacteroides | Bacteroides sp. |
| 20321 | 8_1313 | 11 | 7 | Bacteroidetes | Bacteroidia | Bacteroidales | Bacteroidaceae | Bacteroides | Bacteroides sp. |
| 20368 | 9_57 | 59 | 6 | Bacteroidetes | Bacteroidia | Bacteroidales | Bacteroidaceae | Bacteroides | Bacteroides sp. |
| 20376 | 126_132 | 78 | 28 | Bacteroidetes | Bacteroidia | Bacteroidales | Porphyromonadaceae | Parabacteroides | Parabacteroides merdae |
| 20378 | 105_4655 | 8 | 6 | Bacteroidetes | Bacteroidia | Bacteroidales | Bacteroidaceae | Bacteroides | Bacteroides massiliensis |
| 20384 | 131_7281 | 32 | 2 | Bacteroidetes | Bacteroidia | Bacteroidales | Bacteroidaceae | Bacteroides | Bacteroides eggerthii |
| 20401 | 117_1172 | 25 | 11 | Firmicutes | Negativicutes | Selenomonadales | Veillonellaceae | Dialister | Dialister invisus |
| 20406 | 117_2399 | 36 | 11 | Firmicutes | Negativicutes | Selenomonadales | Veillonellaceae | Dialister | Dialister invisus |
| 20568 | 17_3205 | 16 | 8 | Bacteroidetes | Bacteroidia | Bacteroidales | Bacteroidaceae | Bacteroides | Bacteroides massiliensis |
| 20586 | 130_5084 | 9 | 8 | Firmicutes | Negativicutes | Selenomonadales | Veillonellaceae | Dialister | Dialister invisus |
| 20672 | 61_2713 | 9 | 5 | NULL | NULL | NULL | NULL | NULL | NULL |
| 20707 | 47_11291 | 31 | 9 | Firmicutes | Negativicutes | Selenomonadales | Veillonellaceae | Mitsuokella | Mitsuokella |
| 20724 | 61_3432 | 153 | 22 | Firmicutes | Clostridia | Clostridiales | Ruminococcaceae | Faecalibacterium | Faecalibacterium prausnitzii |
| 20760 | 49_1738 | 10 | 6 | Firmicutes | Negativicutes | Selenomonadales | Veillonellaceae | Dialister | Dialister invisus |
| 20826 | 5_216 | 401 | 53 | Firmicutes | Clostridia | Clostridiales | Ruminococcaceae | Faecalibacterium | Faecalibacterium prausnitzii |
| 20827 | 10_2630 | 24 | 12 | Bacteroidetes | Bacteroidia | Bacteroidales | Rikenellaceae | Alistipes | Alistipes sp. |
| 20841 | 115_2924 | 62 | 16 | Firmicutes | Clostridia | Clostridiales | Ruminococcaceae | Faecalibacterium | Faecalibacterium prausnitzii |
| 20843 | 37_4927 | 8 | 7 | Firmicutes | Negativicutes | Selenomonadales | Veillonellaceae | Dialister | Dialister invisus |
| 20845 | 130_6828 | 27 | 9 | Firmicutes | Negativicutes | Selenomonadales | Veillonellaceae | Dialister | Dialister invisus |
| 20912 | 18_3113 | 18 | 9 | Firmicutes | Negativicutes | Selenomonadales | Veillonellaceae | Dialister | Dialister invisus |
| 20918 | 24_513 | 258 | 40 | Firmicutes | Clostridia | Clostridiales | Lachnospiraceae | Lachnospiraceae incertae sedis | Lachnospiraceae incertae sedis |
| 20928 | 117_4601 | 8 | 6 | Firmicutes | Negativicutes | Selenomonadales | Veillonellaceae | Dialister | Dialister invisus |
| 20936 | 76_2344 | 109 | 27 | Firmicutes | Clostridia | Clostridiales | Lachnospiraceae | Blautia | Blautia |
| 20938 | 65_4226 | 1002 | 85 | Firmicutes | Clostridia | Clostridiales | Lachnospiraceae | Blautia | Blautia |
| 20939 | 88_1851 | 14 | 8 | Firmicutes | Clostridia | Clostridiales | Lachnospiraceae | Blautia | Blautia |
| 20952 | 117_2479 | 9 | 5 | Firmicutes | Negativicutes | Selenomonadales | Veillonellaceae | Dialister | Dialister invisus |
| 20956 | 8_2024 | 2545 | 114 | Firmicutes | Clostridia | Clostridiales | Lachnospiraceae | Blautia | Blautia |
| 20957 | 11_1603 | 508 | 66 | Firmicutes | Clostridia | Clostridiales | Lachnospiraceae | Blautia | Blautia |
| 20960 | 7_2207 | 9 | 7 | Firmicutes | Negativicutes | Selenomonadales | Veillonellaceae | Dialister | Dialister invisus |
| 20982 | 24_259 | 30 | 6 | NULL | NULL | NULL | NULL | NULL | NULL |
| 20991 | 49_240 | 392 | 57 | NULL | NULL | NULL | NULL | NULL | NULL |
| 21118 | 32_6635 | 79 | 10 | Bacteroidetes | Bacteroidia | Bacteroidales | Bacteroidaceae | Bacteroides | Bacteroides dorei |
| 21129 | 32_101 | 34 | 15 | Bacteroidetes | Bacteroidia | Bacteroidales | Bacteroidaceae | Bacteroides | Bacteroides massiliensis |
| 21144 | 39_1489 | 27 | 6 | Bacteroidetes | Bacteroidia | Bacteroidales | Porphyromonadaceae | Barnesiella | Barnsiella intestinihominis |
| 21191 | 28_882 | 21 | 7 | Bacteroidetes | Bacteroidia | Bacteroidales | Porphyromonadaceae | Butyricimonas | Butyricimonas virosa |
| 21193 | 20_8743 | 9 | 5 | Bacteroidetes | Bacteroidia | Bacteroidales | Porphyromonadaceae | Butyricimonas | Butyricimonas virosa |
| 21199 | 110_5320 | 27 | 2 | Bacteroidetes | Bacteroidia | Bacteroidales | Bacteroidaceae | Bacteroides | Bacteroides eggerthii |
| 21203 | 98_3217 | 225 | 3 | Bacteroidetes | Bacteroidia | Bacteroidales | Porphyromonadaceae | Barnesiella | Barnesiella sp. |
| 21215 | 113_5298 | 6 | 5 | Bacteroidetes | Bacteroidia | Bacteroidales | Porphyromonadaceae | Parabacteroides | Parabacteroides johnsonii |
| 21249 | 18_5421 | 24 | 11 | Bacteroidetes | Bacteroidia | Bacteroidales | Bacteroidaceae | Bacteroides | Bacteroides sp. |
| 21259 | 117_4312 | 16 | 12 | Bacteroidetes | Bacteroidia | Bacteroidales | Porphyromonadaceae | Parabacteroides | Parabacteroides merdae |
| 21262 | 125_534 | 12 | 5 | Bacteroidetes | Bacteroidia | Bacteroidales | Porphyromonadaceae | Parabacteroides | Parabacteroides merdae |
| 21263 | 2_3387 | 6 | 5 | Bacteroidetes | Bacteroidia | Bacteroidales | Porphyromonadaceae | Parabacteroides | Parabacteroides merdae |
| 21268 | 51_2384 | 50 | 10 | Bacteroidetes | Bacteroidia | Bacteroidales | Porphyromonadaceae | Parabacteroides | Parabacteroides merdae |
| 21269 | 10_5091 | 27 | 14 | Bacteroidetes | Bacteroidia | Bacteroidales | Bacteroidaceae | Bacteroides | Bacteroides uniformis |
| 21272 | 24_3196 | 20 | 14 | Bacteroidetes | Bacteroidia | Bacteroidales | Bacteroidaceae | Bacteroides | Bacteroides massiliensis |
| 21295 | 20_4500 | 17 | 7 | Bacteroidetes | Bacteroidia | Bacteroidales | Bacteroidaceae | Bacteroides | Bacteroides eggerthii |
| 21302 | 84_5646 | 7 | 5 | Bacteroidetes | Bacteroidia | Bacteroidales | Bacteroidaceae | Bacteroides | Bacteroides massiliensis |
| 21320 | 49_1027 | 6 | 5 | Bacteroidetes | Bacteroidia | Bacteroidales | Bacteroidaceae | Bacteroides | Bacteroides massiliensis |
| 21322 | 96_3943 | 18 | 11 | Bacteroidetes | Bacteroidia | Bacteroidales | Bacteroidaceae | Bacteroides | Bacteroides massiliensis |
| 21325 | 124_5529 | 24 | 16 | Bacteroidetes | Bacteroidia | Bacteroidales | Bacteroidaceae | Bacteroides | Bacteroides massiliensis |
| 21362 | 119_2657 | 19 | 5 | Bacteroidetes | Bacteroidia | Bacteroidales | Porphyromonadaceae | Odoribacter | Odoribacter splanchnicus |
| 21363 | 20_8533 | 7 | 5 | Bacteroidetes | Bacteroidia | Bacteroidales | Porphyromonadaceae | Odoribacter | Odoribacter splanchnicus |
| 21381 | 20_8658 | 36 | 3 | Bacteroidetes | Bacteroidia | Bacteroidales | Bacteroidaceae | Bacteroides | Bacteroides eggerthii |
| 21391 | 101_5274 | 148 | 17 | Bacteroidetes | Bacteroidia | Bacteroidales | Bacteroidaceae | Bacteroides | Bacteroides uniformis |
| 21421 | 20_7581 | 27 | 3 | Bacteroidetes | Bacteroidia | Bacteroidales | Bacteroidaceae | Bacteroides | Bacteroides eggerthii |
| 21435 | 19_4818 | 45 | 9 | Bacteroidetes | Bacteroidia | Bacteroidales | Rikenellaceae | Alistipes | Alistipes putredinis |
| 21438 | 20_4391 | 48 | 4 | Bacteroidetes | Bacteroidia | Bacteroidales | Bacteroidaceae | Bacteroides | Bacteroides eggerthii |
| 21445 | 6_2475 | 69 | 13 | Bacteroidetes | Bacteroidia | Bacteroidales | Rikenellaceae | Alistipes | Alistipes sp. |
| 21448 | 19_2519 | 11 | 8 | Bacteroidetes | Bacteroidia | Bacteroidales | Rikenellaceae | Alistipes | Alistipes indistinctus |
| 21452 | 115_1632 | 8 | 6 | Bacteroidetes | Bacteroidia | Bacteroidales | Bacteroidaceae | Bacteroides | Bacteroides uniformis |
| 21458 | 29_4824 | 46 | 11 | Bacteroidetes | Bacteroidia | Bacteroidales | Rikenellaceae | Alistipes | Alistipes finegoldii |
| 21461 | 37_3084 | 106 | 30 | Bacteroidetes | Bacteroidia | Bacteroidales | Rikenellaceae | Alistipes | Alistipes finegoldii |
| 21462 | 127_959 | 6 | 6 | Bacteroidetes | Bacteroidia | Bacteroidales | Rikenellaceae | Alistipes | Alistipes finegoldii |
| 21472 | 96_9953 | 16 | 5 | Bacteroidetes | Bacteroidia | Bacteroidales | Bacteroidaceae | Bacteroides | Bacteroides sp. |
| 21484 | 94_7969 | 30 | 3 | Bacteroidetes | Bacteroidia | Bacteroidales | Bacteroidaceae | Bacteroides | Bacteroides ovatus |
| 21544 | 68_3116 | 10 | 5 | Bacteroidetes | Bacteroidia | Bacteroidales | Bacteroidaceae | Bacteroides | Bacteroides thetaiotaomicron |
| 21552 | 102_2684 | 148 | 17 | Bacteroidetes | Bacteroidia | Bacteroidales | Bacteroidaceae | Bacteroides | Bacteroides ovatus |
| 21560 | 102_4821 | 38 | 8 | Bacteroidetes | Bacteroidia | Bacteroidales | Bacteroidaceae | Bacteroides | Bacteroides ovatus |
| 21590 | 102_1520 | 19 | 5 | Bacteroidetes | Bacteroidia | Bacteroidales | Bacteroidaceae | Bacteroides | Bacteroides thetaiotaomicron |
| 21611 | 100_11427 | 21 | 2 | Bacteroidetes | Bacteroidia | Bacteroidales | Bacteroidaceae | Bacteroides | Bacteroides sp. |
| 21625 | 68_6274 | 13 | 7 | Bacteroidetes | Bacteroidia | Bacteroidales | Bacteroidaceae | Bacteroides | Bacteroides ovatus |
| 21631 | 79_8657 | 29 | 15 | Bacteroidetes | Bacteroidia | Bacteroidales | Bacteroidaceae | Bacteroides | Bacteroides uniformis |
| 21646 | 90_5086 | 13 | 6 | Bacteroidetes | Bacteroidia | Bacteroidales | Bacteroidaceae | Bacteroides | Bacteroides uniformis |
| 21664 | 94_9841 | 21 | 7 | Bacteroidetes | Bacteroidia | Bacteroidales | Bacteroidaceae | Bacteroides | Bacteroides ovatus |
| 21665 | 82_11391 | 13 | 5 | Bacteroidetes | Bacteroidia | Bacteroidales | Bacteroidaceae | Bacteroides | Bacteroides ovatus |
| 21670 | 9_2922 | 20 | 5 | Bacteroidetes | Bacteroidia | Bacteroidales | Bacteroidaceae | Bacteroides | Bacteroides eggerthii |
| 21682 | 60_3274 | 130 | 12 | Bacteroidetes | Bacteroidia | Bacteroidales | Bacteroidaceae | Bacteroides | Bacteroides sp. |
| 21683 | 116_1081 | 54 | 9 | Bacteroidetes | Bacteroidia | Bacteroidales | Bacteroidaceae | Bacteroides | Bacteroides sp. |
| 21685 | 94_3376 | 8 | 6 | Bacteroidetes | Bacteroidia | Bacteroidales | Bacteroidaceae | Bacteroides | Bacteroides ovatus |
| 21692 | 82_640 | 38 | 6 | Bacteroidetes | Bacteroidia | Bacteroidales | Bacteroidaceae | Bacteroides | Bacteroides ovatus |
| 21695 | 7_80 | 16 | 8 | Bacteroidetes | Bacteroidia | Bacteroidales | Bacteroidaceae | Bacteroides | Bacteroides ovatus |
| 21706 | 105_6436 | 14 | 7 | Bacteroidetes | Bacteroidia | Bacteroidales | Bacteroidaceae | Bacteroides | Bacteroides ovatus |
| 21733 | 82_1720 | 33 | 3 | Bacteroidetes | Bacteroidia | Bacteroidales | Bacteroidaceae | Bacteroides | Bacteroides massiliensis |
| 21735 | 82_639 | 6 | 5 | Bacteroidetes | Bacteroidia | Bacteroidales | Bacteroidaceae | Bacteroides | Bacteroides caccae |
| 21737 | 79_8068 | 84 | 4 | Bacteroidetes | Bacteroidia | Bacteroidales | Bacteroidaceae | Bacteroides | Bacteroides ovatus |
| 21752 | 68_4989 | 10 | 8 | Bacteroidetes | Bacteroidia | Bacteroidales | Bacteroidaceae | Bacteroides | Bacteroides ovatus |
| 21757 | 68_5879 | 29 | 4 | Bacteroidetes | Bacteroidia | Bacteroidales | Bacteroidaceae | Bacteroides | Bacteroides sp. |
| 21766 | 56_1849 | 6 | 5 | Bacteroidetes | Bacteroidia | Bacteroidales | Bacteroidaceae | Bacteroides | Bacteroides ovatus |
| 21815 | 101_2474 | 9 | 5 | Bacteroidetes | Bacteroidia | Bacteroidales | Bacteroidaceae | Bacteroides | Bacteroides thetaiotaomicron |
| 21823 | 101_5012 | 22 | 9 | Bacteroidetes | Bacteroidia | Bacteroidales | Bacteroidaceae | Bacteroides | Bacteroides thetaiotaomicron |
| 21830 | 131_4791 | 23 | 6 | Bacteroidetes | Bacteroidia | Bacteroidales | Bacteroidaceae | Bacteroides | Bacteroides eggerthii |
| 21832 | 94_5228 | 6 | 5 | Bacteroidetes | Bacteroidia | Bacteroidales | Bacteroidaceae | Bacteroides | Bacteroides ovatus |
| 21835 | 63_2617 | 21 | 9 | Bacteroidetes | Bacteroidia | Bacteroidales | Bacteroidaceae | Bacteroides | Bacteroides thetaiotaomicron |
| 21888 | 100_3114 | 29 | 3 | Bacteroidetes | Bacteroidia | Bacteroidales | Bacteroidaceae | Bacteroides | Bacteroides sp. |
| 21892 | 100_6066 | 24 | 4 | Bacteroidetes | Bacteroidia | Bacteroidales | Bacteroidaceae | Bacteroides | Bacteroides sp. |
| 21896 | 107_4094 | 13 | 7 | Bacteroidetes | Bacteroidia | Bacteroidales | Bacteroidaceae | Bacteroides | Bacteroides sp. |
| 21907 | 122_2421 | 7 | 5 | Bacteroidetes | Bacteroidia | Bacteroidales | Bacteroidaceae | Bacteroides | Bacteroides intestinalis |
| 21908 | 101_380 | 7 | 5 | Bacteroidetes | Bacteroidia | Bacteroidales | Bacteroidaceae | Bacteroides | Bacteroides thetaiotaomicron |
| 21913 | 114_3044 | 11 | 6 | Bacteroidetes | Bacteroidia | Bacteroidales | Bacteroidaceae | Bacteroides | Bacteroides caccae |
| 21930 | 91_5386 | 29 | 14 | Bacteroidetes | Bacteroidia | Bacteroidales | Bacteroidaceae | Bacteroides | Bacteroides caccae |
| 21947 | 125_2238 | 6 | 5 | Bacteroidetes | Bacteroidia | Bacteroidales | Prevotellaceae | Prevotella | Prevotella copri |
| 21949 | 105_5636 | 16 | 5 | Bacteroidetes | Bacteroidia | Bacteroidales | Bacteroidaceae | Bacteroides | Bacteroides sp. |
| 21953 | 40_4181 | 16 | 11 | Bacteroidetes | Bacteroidia | Bacteroidales | Bacteroidaceae | Bacteroides | Bacteroides uniformis |
| 21956 | 105_917 | 45 | 8 | Bacteroidetes | Bacteroidia | Bacteroidales | Bacteroidaceae | Bacteroides | Bacteroides sp. |
| 21970 | 105_6301 | 19 | 7 | Bacteroidetes | Bacteroidia | Bacteroidales | Bacteroidaceae | Bacteroides | Bacteroides thetaiotaomicron |
| 21973 | 126_440 | 34 | 3 | Bacteroidetes | Bacteroidia | Bacteroidales | Bacteroidaceae | Bacteroides | Bacteroides sp. |
| 21974 | 105_6348 | 37 | 5 | Bacteroidetes | Bacteroidia | Bacteroidales | Bacteroidaceae | Bacteroides | Bacteroides sp. |
| 21996 | 20_3893 | 14 | 5 | Bacteroidetes | Bacteroidia | Bacteroidales | Bacteroidaceae | Bacteroides | Bacteroides sp. |
| 22001 | 6_2459 | 9 | 7 | Bacteroidetes | Bacteroidia | Bacteroidales | Prevotellaceae | Prevotella | Prevotella copri |
| 22006 | 71_3533 | 14 | 10 | Bacteroidetes | Bacteroidia | Bacteroidales | Prevotellaceae | Prevotella | Prevotella copri |
| 22008 | 63_4868 | 54 | 14 | Bacteroidetes | Bacteroidia | Bacteroidales | Bacteroidaceae | Bacteroides | Bacteroides eggerthii |
| 22015 | 4_4004 | 62 | 25 | Bacteroidetes | Bacteroidia | Bacteroidales | Bacteroidaceae | Bacteroides | Bacteroides uniformis |
| 22020 | 4_3557 | 11 | 7 | Bacteroidetes | Bacteroidia | Bacteroidales | Prevotellaceae | Prevotella | Prevotella copri |
| 22025 | 50_1181 | 5 | 5 | Bacteroidetes | Bacteroidia | Bacteroidales | Porphyromonadaceae | Parabacteroides | Parabacteroides merdae |
| 22030 | 27_2163 | 5 | 5 | Bacteroidetes | Bacteroidia | Bacteroidales | Bacteroidaceae | Bacteroides | Bacteroides caccae |
| 22074 | 30_10087 | 21 | 7 | Bacteroidetes | Bacteroidia | Bacteroidales | Bacteroidaceae | Bacteroides | Bacteroides fragilis |
| 22106 | 96_9706 | 13 | 6 | Bacteroidetes | Bacteroidia | Bacteroidales | Bacteroidaceae | Bacteroides | Bacteroides sp. |
| 22112 | 90_9456 | 284 | 37 | Bacteroidetes | Bacteroidia | Bacteroidales | Bacteroidaceae | Bacteroides | Bacteroides uniformis |
| 22127 | 67_8513 | 7 | 5 | Bacteroidetes | Bacteroidia | Bacteroidales | Bacteroidaceae | Bacteroides | Bacteroides uniformis |
| 22146 | 107_4511 | 13 | 5 | Bacteroidetes | Bacteroidia | Bacteroidales | Bacteroidaceae | Bacteroides | Bacteroides massiliensis |
| 22149 | 1_169 | 36 | 15 | Bacteroidetes | Bacteroidia | Bacteroidales | Bacteroidaceae | Bacteroides | Bacteroides massiliensis |
| 22152 | 96_4671 | 47 | 9 | Bacteroidetes | Bacteroidia | Bacteroidales | Bacteroidaceae | Bacteroides | Bacteroides massiliensis |
| 22153 | 20_3053 | 22 | 6 | Bacteroidetes | Bacteroidia | Bacteroidales | Bacteroidaceae | Bacteroides | Bacteroides eggerthii |
| 22177 | 60_1132 | 7 | 7 | Bacteroidetes | Bacteroidia | Bacteroidales | Bacteroidaceae | Bacteroides | Bacteroides massiliensis |
| 22182 | 124_987 | 20 | 8 | Bacteroidetes | Bacteroidia | Bacteroidales | Bacteroidaceae | Bacteroides | Bacteroides massiliensis |
| 22197 | 60_4189 | 28 | 16 | Bacteroidetes | Bacteroidia | Bacteroidales | Porphyromonadaceae | Parabacteroides | Parabacteroides merdae |
| 22209 | 124_1375 | 35 | 20 | Bacteroidetes | Bacteroidia | Bacteroidales | Bacteroidaceae | Bacteroides | Bacteroides massiliensis |
| 22220 | 69_70 | 16 | 10 | Bacteroidetes | Bacteroidia | Bacteroidales | Bacteroidaceae | Bacteroides | Bacteroides uniformis |
| 22230 | 50_3128 | 44 | 17 | Bacteroidetes | Bacteroidia | Bacteroidales | Bacteroidaceae | Bacteroides | Bacteroides massiliensis |
| 22248 | 39_862 | 19 | 5 | Bacteroidetes | Bacteroidia | Bacteroidales | Prevotellaceae | Prevotella | Prevotella sp. |
| 22255 | 40_2301 | 11 | 6 | Bacteroidetes | Bacteroidia | Bacteroidales | Bacteroidaceae | Bacteroides | Bacteroides eggerthii |
| 22269 | 20_4229 | 5 | 5 | Bacteroidetes | Bacteroidia | Bacteroidales | Bacteroidaceae | Bacteroides | Bacteroides eggerthii |
| 22305 | 39_1914 | 10 | 6 | Bacteroidetes | Bacteroidia | Bacteroidales | Bacteroidaceae | Bacteroides | Bacteroides eggerthii |
| 22309 | 29_812 | 20 | 2 | Bacteroidetes | Bacteroidia | Bacteroidales | Bacteroidaceae | Bacteroides | Bacteroides eggerthii |
| 22314 | 20_6735 | 7 | 7 | Bacteroidetes | Bacteroidia | Bacteroidales | Bacteroidaceae | Bacteroides | Bacteroides sp. |
| 22324 | 115_305 | 13 | 7 | Bacteroidetes | Bacteroidia | Bacteroidales | Bacteroidaceae | Bacteroides | Bacteroides sp. |
| 22333 | 109_1068 | 9 | 6 | Bacteroidetes | Bacteroidia | Bacteroidales | Bacteroidaceae | Bacteroides | Bacteroides uniformis |
| 22336 | 39_3940 | 12 | 6 | Bacteroidetes | Bacteroidia | Bacteroidales | Bacteroidaceae | Bacteroides | Bacteroides finegoldii |
| 22350 | 120_2778 | 6 | 6 | Bacteroidetes | Bacteroidia | Bacteroidales | Bacteroidaceae | Bacteroides | Bacteroides uniformis |
| 22361 | 109_2061 | 75 | 14 | Bacteroidetes | Bacteroidia | Bacteroidales | Bacteroidaceae | Bacteroides | Bacteroides eggerthii |
| 22383 | 76_2624 | 29 | 8 | Bacteroidetes | Bacteroidia | Bacteroidales | Bacteroidaceae | Bacteroides | Bacteroides uniformis |
| 22395 | 131_9624 | 20 | 11 | Bacteroidetes | Bacteroidia | Bacteroidales | Bacteroidaceae | Bacteroides | Bacteroides uniformis |
| 22399 | 111_8713 | 35 | 10 | Bacteroidetes | Bacteroidia | Bacteroidales | Bacteroidaceae | Bacteroides | Bacteroides uniformis |
| 22414 | 65_5665 | 43 | 17 | Bacteroidetes | Bacteroidia | Bacteroidales | Bacteroidaceae | Bacteroides | Bacteroides uniformis |
| 22416 | 111_13292 | 39 | 11 | Bacteroidetes | Bacteroidia | Bacteroidales | Bacteroidaceae | Bacteroides | Bacteroides uniformis |
| 22440 | 99_10408 | 6 | 5 | Bacteroidetes | Bacteroidia | Bacteroidales | Bacteroidaceae | Bacteroides | Bacteroides uniformis |
| 22446 | 109_14 | 21 | 13 | Bacteroidetes | Bacteroidia | Bacteroidales | Bacteroidaceae | Bacteroides | Bacteroides finegoldii |
| 22466 | 62_4688 | 23 | 5 | Bacteroidetes | Bacteroidia | Bacteroidales | Prevotellaceae | Prevotella | Prevotella shahii |
| 22468 | 86_3320 | 16 | 9 | Bacteroidetes | Bacteroidia | Bacteroidales | Bacteroidaceae | Bacteroides | Bacteroides eggerthii |
| 22474 | 101_6468 | 10 | 8 | Bacteroidetes | Bacteroidia | Bacteroidales | Bacteroidaceae | Bacteroides | Bacteroides uniformis |
| 22484 | 67_7929 | 31 | 14 | Bacteroidetes | Bacteroidia | Bacteroidales | Bacteroidaceae | Bacteroides | Bacteroides uniformis |
| 22491 | 39_2451 | 40 | 7 | Bacteroidetes | Bacteroidia | Bacteroidales | Prevotellaceae | Prevotella | Prevotella sp. |
| 22495 | 63_2999 | 50 | 24 | Bacteroidetes | Bacteroidia | Bacteroidales | Bacteroidaceae | Bacteroides | Bacteroides uniformis |
| 22503 | 1_3116 | 112 | 36 | Bacteroidetes | Bacteroidia | Bacteroidales | Porphyromonadaceae | Parabacteroides | Parabacteroides merdae |
| 22506 | 131_8640 | 13 | 6 | Bacteroidetes | Bacteroidia | Bacteroidales | Bacteroidaceae | Bacteroides | Bacteroides eggerthii |
| 22510 | 101_6346 | 17 | 6 | Bacteroidetes | Bacteroidia | Bacteroidales | Bacteroidaceae | Bacteroides | Bacteroides uniformis |
| 22515 | 130_1978 | 8 | 6 | Bacteroidetes | Bacteroidia | Bacteroidales | Bacteroidaceae | Bacteroides | Bacteroides thetaiotaomicron |
| 22522 | 75_1109 | 10 | 5 | Bacteroidetes | Bacteroidia | Bacteroidales | Bacteroidaceae | Bacteroides | Bacteroides eggerthii |
| 22524 | 9_6460 | 7 | 5 | Bacteroidetes | Bacteroidia | Bacteroidales | Porphyromonadaceae | Barnesiella | Barnesiella intestinihominis |
| 22526 | 20_6299 | 15 | 6 | Bacteroidetes | Bacteroidia | Bacteroidales | Bacteroidaceae | Bacteroides | Bacteroides uniformis |
| 22531 | 63_2846 | 39 | 17 | Bacteroidetes | Bacteroidia | Bacteroidales | Bacteroidaceae | Bacteroides | Bacteroides uniformis |
| 22532 | 111_11518 | 52 | 15 | Bacteroidetes | Bacteroidia | Bacteroidales | Bacteroidaceae | Bacteroides | Bacteroides uniformis |
| 22550 | 10_3029 | 9 | 6 | Bacteroidetes | Bacteroidia | Bacteroidales | Bacteroidaceae | Bacteroides | Bacteroides uniformis |
| 22554 | 107_4139 | 18 | 11 | Bacteroidetes | Bacteroidia | Bacteroidales | Bacteroidaceae | Bacteroides | Bacteroides uniformis |
| 22570 | 30_5209 | 11 | 7 | Bacteroidetes | Bacteroidia | Bacteroidales | Bacteroidaceae | Bacteroides | Bacteroides uniformis |
| 22611 | 19_4838 | 36 | 23 | Bacteroidetes | Bacteroidia | Bacteroidales | Porphyromonadaceae | Parabacteroides | Parabacteroides merdae |
| 22613 | 63_2801 | 9 | 6 | Bacteroidetes | Bacteroidia | Bacteroidales | Bacteroidaceae | Bacteroides | Bacteroides uniformis |
| 22645 | 120_3273 | 9 | 6 | Bacteroidetes | Bacteroidia | Bacteroidales | Bacteroidaceae | Bacteroides | Bacteroides eggerthii |
| 22695 | 39_2838 | 22 | 16 | Bacteroidetes | Bacteroidia | Bacteroidales | Bacteroidaceae | Bacteroides | Bacteroides uniformis |
| 22697 | 60_6761 | 7 | 6 | Bacteroidetes | Bacteroidia | Bacteroidales | Porphyromonadaceae | Parabacteroides | Parabacteroides merdae |
| 22716 | 105_6102 | 250 | 42 | Bacteroidetes | Bacteroidia | Bacteroidales | Bacteroidaceae | Bacteroides | Bacteroides massiliensis |
| 22735 | 100_1189 | 52 | 9 | Bacteroidetes | Bacteroidia | Bacteroidales | Porphyromonadaceae | Parabacteroides | Parabacteroides distasonis |
| 22740 | 20_1863 | 18 | 6 | Bacteroidetes | Bacteroidia | Bacteroidales | Bacteroidaceae | Bacteroides | Bacteroides eggerthii |
| 22742 | 96_3243 | 21 | 9 | Bacteroidetes | Bacteroidia | Bacteroidales | Bacteroidaceae | Bacteroides | Bacteroides sp. |
| 22746 | 79_3507 | 18 | 8 | Bacteroidetes | Bacteroidia | Bacteroidales | Bacteroidaceae | Bacteroides | Bacteroides sp. |
| 22772 | 115_2762 | 18 | 11 | Bacteroidetes | Bacteroidia | Bacteroidales | Bacteroidaceae | Bacteroides | Bacteroides sp. |
| 22793 | 60_3137 | 10 | 7 | Bacteroidetes | Bacteroidia | Bacteroidales | Bacteroidaceae | Bacteroides | Bacteroides sp. |
| 22797 | 40_4997 | 24 | 12 | Bacteroidetes | Bacteroidia | Bacteroidales | Bacteroidaceae | Bacteroides | Bacteroides eggerthii |
| 22847 | 56_10473 | 9 | 5 | Bacteroidetes | Bacteroidia | Bacteroidales | Bacteroidaceae | Bacteroides | Bacteroides sp. |
| 22860 | 105_1137 | 13 | 9 | Bacteroidetes | Bacteroidia | Bacteroidales | Bacteroidaceae | Bacteroides | Bacteroides massiliensis |
| 22889 | 26_807 | 36 | 16 | Bacteroidetes | Bacteroidia | Bacteroidales | Bacteroidaceae | Bacteroides | Bacteroides massiliensis |
| 22899 | 130_6620 | 11 | 5 | Bacteroidetes | Bacteroidia | Bacteroidales | Bacteroidaceae | Bacteroides | Bacteroides vulgatus |
| 22911 | 42_2257 | 15 | 12 | Bacteroidetes | Bacteroidia | Bacteroidales | Bacteroidaceae | Bacteroides | Bacteroides uniformis |
| 22917 | 19_3444 | 96 | 14 | Bacteroidetes | Bacteroidia | Bacteroidales | Porphyromonadaceae | Parabacteroides | Parabacteroides distasonis |
| 22922 | 38_6816 | 8 | 5 | Bacteroidetes | Bacteroidia | Bacteroidales | Porphyromonadaceae | Parabacteroides | Parabacteroides distasonis |
| 22944 | 17_90 | 8 | 7 | Bacteroidetes | Bacteroidia | Bacteroidales | Porphyromonadaceae | Parabacteroides | Parabacteroides distasonis |
| 22945 | 102_3207 | 20 | 8 | Bacteroidetes | Bacteroidia | Bacteroidales | Porphyromonadaceae | Parabacteroides | Parabacteroides distasonis |
| 23010 | 50_1589 | 10 | 5 | Bacteroidetes | Bacteroidia | Bacteroidales | Bacteroidaceae | Bacteroides | Bacteroides massiliensis |
| 23018 | 61_5715 | 16 | 10 | Bacteroidetes | Bacteroidia | Bacteroidales | Bacteroidaceae | Bacteroides | Bacteroides uniformis |
| 23025 | 69_4286 | 13 | 9 | Bacteroidetes | Bacteroidia | Bacteroidales | Bacteroidaceae | Bacteroides | Bacteroides uniformis |
| 23029 | 61_5141 | 29 | 12 | Bacteroidetes | Bacteroidia | Bacteroidales | Porphyromonadaceae | Parabacteroides | Parabacteroides distasonis |
| 23060 | 129_904 | 31 | 14 | Bacteroidetes | Bacteroidia | Bacteroidales | Porphyromonadaceae | Parabacteroides | Parabacteroides merdae |
| 23068 | 120_2848 | 8 | 6 | Bacteroidetes | Bacteroidia | Bacteroidales | Bacteroidaceae | Bacteroides | Bacteroides uniformis |
| 23072 | 109_2959 | 35 | 15 | Bacteroidetes | Bacteroidia | Bacteroidales | Bacteroidaceae | Bacteroides | Bacteroides eggerthii |
| 23093 | 11_2241 | 20 | 7 | Bacteroidetes | Bacteroidia | Bacteroidales | Bacteroidaceae | Bacteroides | Bacteroides eggerthii |
| 23103 | 126_938 | 15 | 5 | Bacteroidetes | Bacteroidia | Bacteroidales | Bacteroidaceae | Bacteroides | Bacteroides ovatus |
| 23106 | 2_1434 | 10 | 6 | Bacteroidetes | Bacteroidia | Bacteroidales | Bacteroidaceae | Bacteroides | Bacteroides stercorirosoris |
| 23108 | 113_2684 | 148 | 9 | Bacteroidetes | Bacteroidia | Bacteroidales | Bacteroidaceae | Bacteroides | Bacteroides intestinalis |
| 23110 | 32_3497 | 20 | 9 | Bacteroidetes | Bacteroidia | Bacteroidales | Bacteroidaceae | Bacteroides | Bacteroides uniformis |
| 23127 | 116_1113 | 55 | 22 | Bacteroidetes | Bacteroidia | Bacteroidales | Bacteroidaceae | Bacteroides | Bacteroides sp. |
| 23163 | 124_3227 | 31 | 17 | Bacteroidetes | Bacteroidia | Bacteroidales | Bacteroidaceae | Bacteroides | Bacteroides massiliensis |
| 23168 | 58_7370 | 2779 | 48 | Bacteroidetes | Bacteroidia | Bacteroidales | Bacteroidaceae | Bacteroides | Bacteroides vulgatus |
| 23175 | 128_5531 | 17 | 14 | Bacteroidetes | Bacteroidia | Bacteroidales | Bacteroidaceae | Bacteroides | Bacteroides vulgatus |
| 23181 | 124_3502 | 17 | 7 | Bacteroidetes | Bacteroidia | Bacteroidales | Bacteroidaceae | Bacteroides | Bacteroides vulgatus |
| 23185 | 60_2917 | 16 | 11 | Bacteroidetes | Bacteroidia | Bacteroidales | Porphyromonadaceae | Parabacteroides | Parabacteroides merdae |
| 23193 | 76_2539 | 36 | 7 | Bacteroidetes | Bacteroidia | Bacteroidales | Bacteroidaceae | Bacteroides | Bacteroides fragilis |
| 23196 | 56_5303 | 93 | 25 | Bacteroidetes | Bacteroidia | Bacteroidales | Bacteroidaceae | Bacteroides | Bacteroides sp. |
| 23206 | 56_5733 | 11 | 6 | Bacteroidetes | Bacteroidia | Bacteroidales | Bacteroidaceae | Bacteroides | Bacteroides ovatus |
| 23223 | 12_2539 | 108 | 24 | Bacteroidetes | Bacteroidia | Bacteroidales | Bacteroidaceae | Bacteroides | Bacteroides uniformis |
| 23227 | 76_1004 | 12 | 5 | Bacteroidetes | Bacteroidia | Bacteroidales | Bacteroidaceae | Bacteroides | Bacteroides fragilis |
| 23235 | 124_3214 | 156 | 33 | Bacteroidetes | Bacteroidia | Bacteroidales | Bacteroidaceae | Bacteroides | Bacteroides vulgatus |
| 23239 | 49_3557 | 169 | 36 | Bacteroidetes | Bacteroidia | Bacteroidales | Bacteroidaceae | Bacteroides | Bacteroides vulgatus |
| 23247 | 132_1171 | 6 | 5 | Bacteroidetes | Bacteroidia | Bacteroidales | Bacteroidaceae | Bacteroides | Bacteroides vulgatus |
| 23255 | 7_2542 | 13 | 8 | Bacteroidetes | Bacteroidia | Bacteroidales | Porphyromonadaceae | Parabacteroides | Parabacteroides merdae |
| 23256 | 123_3596 | 32 | 13 | Bacteroidetes | Bacteroidia | Bacteroidales | Bacteroidaceae | Bacteroides | Bacteroides vulgatus |
| 23268 | 115_2110 | 39 | 14 | Bacteroidetes | Bacteroidia | Bacteroidales | Bacteroidaceae | Bacteroides | Bacteroides uniformis |
| 23276 | 68_9976 | 29 | 13 | Bacteroidetes | Bacteroidia | Bacteroidales | Bacteroidaceae | Bacteroides | Bacteroides sp. |
| 23306 | 20_6385 | 33 | 15 | Bacteroidetes | Bacteroidia | Bacteroidales | Bacteroidaceae | Bacteroides | Bacteroides sp. |
| 23312 | 120_3316 | 44 | 10 | Bacteroidetes | Bacteroidia | Bacteroidales | Bacteroidaceae | Bacteroides | Bacteroides uniformis |
| 23317 | 86_2463 | 15 | 9 | Bacteroidetes | Bacteroidia | Bacteroidales | Bacteroidaceae | Bacteroides | Bacteroides eggerthii |
| 23318 | 107_1581 | 5 | 5 | Bacteroidetes | Bacteroidia | Bacteroidales | Porphyromonadaceae | Parabacteroides | Parabacteroides merdae |
| 23324 | 124_3285 | 13 | 6 | Bacteroidetes | Bacteroidia | Bacteroidales | Bacteroidaceae | Bacteroides | Bacteroides vulgatus |
| 23340 | 75_3039 | 77 | 20 | Bacteroidetes | Bacteroidia | Bacteroidales | Bacteroidaceae | Bacteroides | Bacteroides uniformis |
| 23356 | 76_2724 | 16 | 7 | Bacteroidetes | Bacteroidia | Bacteroidales | Bacteroidaceae | Bacteroides | Bacteroides uniformis |
| 23376 | 120_1833 | 9 | 5 | Bacteroidetes | Bacteroidia | Bacteroidales | Bacteroidaceae | Bacteroides | Bacteroides uniformis |
| 23377 | 60_6502 | 11 | 10 | Bacteroidetes | Bacteroidia | Bacteroidales | Porphyromonadaceae | Parabacteroides | Parabacteroides merdae |
| 23380 | 130_345 | 16 | 9 | Bacteroidetes | Bacteroidia | Bacteroidales | Bacteroidaceae | Bacteroides | Bacteroides caccae |
| 23388 | 17_1621 | 12 | 11 | Bacteroidetes | Bacteroidia | Bacteroidales | Bacteroidaceae | Bacteroides | Bacteroides vulgatus |
| 23402 | 97_4810 | 20 | 12 | Bacteroidetes | Bacteroidia | Bacteroidales | Bacteroidaceae | Bacteroides | Bacteroides vulgatus |
| 23504 | 81_783 | 43 | 3 | Bacteroidetes | Bacteroidia | Bacteroidales | Bacteroidaceae | Bacteroides | Bacteroides plebeius |
| 23511 | 35_3125 | 20 | 1 | Bacteroidetes | Bacteroidia | Bacteroidales | Prevotellaceae | Prevotella | Prevotella copri |
| 23522 | 21_2026 | 8 | 5 | Bacteroidetes | Bacteroidia | Bacteroidales | Prevotellaceae | Prevotella | Prevotella copri |
| 23539 | 20_7418 | 26 | 4 | Bacteroidetes | Bacteroidia | Bacteroidales | Bacteroidaceae | Bacteroides | Bacteroides eggerthii |
| 23551 | 9_3126 | 15 | 5 | Bacteroidetes | Bacteroidia | Bacteroidales | Bacteroidaceae | Bacteroides | Bacteroides vulgatus |
| 23554 | 39_5445 | 43 | 6 | Bacteroidetes | Bacteroidia | Bacteroidales | Bacteroidaceae | Bacteroides | Bacteroides uniformis |
| 23587 | 1_2992 | 8 | 6 | Bacteroidetes | Bacteroidia | Bacteroidales | Prevotellaceae | Prevotella | Prevotella copri |
| 23612 | 81_2692 | 21 | 5 | Bacteroidetes | Bacteroidia | Bacteroidales | Prevotellaceae | Prevotella | Prevotella copri |
| 23662 | 7_5303 | 9 | 8 | Bacteroidetes | Bacteroidia | Bacteroidales | Bacteroidaceae | Bacteroides | Bacteroides uniformis |
| 23739 | 129_3201 | 8 | 7 | Bacteroidetes | Bacteroidia | Bacteroidales | Porphyromonadaceae | Parabacteroides | Parabacteroides merdae |
| 23769 | 115_939 | 25 | 8 | Bacteroidetes | Bacteroidia | Bacteroidales | Prevotellaceae | Prevotella | Prevotella copri |
| 23770 | 128_429 | 9 | 8 | Bacteroidetes | Bacteroidia | Bacteroidales | Porphyromonadaceae | Parabacteroides | Parabacteroides merdae |
| 23775 | 121_1862 | 58 | 10 | Bacteroidetes | Bacteroidia | Bacteroidales | Prevotellaceae | Prevotella | Prevotella stercorea |
| 23784 | 121_2715 | 35 | 8 | Bacteroidetes | Bacteroidia | Bacteroidales | Prevotellaceae | Prevotella | Prevotella copri |
| 23785 | 121_5130 | 15 | 6 | Bacteroidetes | Bacteroidia | Bacteroidales | Prevotellaceae | Prevotella | Prevotella copri |
| 23787 | 8_169 | 5 | 5 | Bacteroidetes | Bacteroidia | Bacteroidales | Bacteroidaceae | Bacteroides | Bacteroides massiliensis |
| 23791 | 25_2843 | 10 | 5 | Bacteroidetes | Bacteroidia | Bacteroidales | Prevotellaceae | Prevotella | Prevotella copri |
| 23793 | 8_2508 | 8 | 6 | Bacteroidetes | Bacteroidia | Bacteroidales | Prevotellaceae | Prevotella | Prevotella copri |
| 23826 | 50_1470 | 5 | 5 | Bacteroidetes | Bacteroidia | Bacteroidales | Prevotellaceae | Prevotella | Prevotella copri |
| 23828 | 112_2293 | 8 | 5 | Bacteroidetes | Bacteroidia | Bacteroidales | Prevotellaceae | Prevotella | Prevotella copri |
| 23834 | 104_792 | 15 | 9 | Bacteroidetes | Bacteroidia | Bacteroidales | Prevotellaceae | Prevotella | Prevotella copri |
| 23835 | 1_1217 | 99 | 17 | Bacteroidetes | Bacteroidia | Bacteroidales | Prevotellaceae | Prevotella | Prevotella copri |
| 23841 | 123_3812 | 18 | 5 | Bacteroidetes | Bacteroidia | Bacteroidales | Prevotellaceae | Prevotella | Prevotella copri |
| 23844 | 104_914 | 305 | 17 | Bacteroidetes | Bacteroidia | Bacteroidales | Prevotellaceae | Prevotella | Prevotella copri |
| 23847 | 104_350 | 52 | 10 | Bacteroidetes | Bacteroidia | Bacteroidales | Prevotellaceae | Prevotella | Prevotella copri |
| 23859 | 4_1232 | 11 | 7 | Bacteroidetes | Bacteroidia | Bacteroidales | Bacteroidaceae | Bacteroides | Bacteroides sp. |
| 23862 | 121_4845 | 8 | 7 | Bacteroidetes | Bacteroidia | Bacteroidales | Bacteroidaceae | Bacteroides | Bacteroides sp. |
| 23874 | 112_1024 | 33 | 8 | Bacteroidetes | Bacteroidia | Bacteroidales | Prevotellaceae | Prevotella | Prevotella copri |
| 23879 | 114_7457 | 9 | 5 | Bacteroidetes | Bacteroidia | Bacteroidales | Prevotellaceae | Prevotella | Prevotella copri |
| 23886 | 123_2741 | 13 | 9 | Bacteroidetes | Bacteroidia | Bacteroidales | Prevotellaceae | Prevotella | Prevotella copri |
| 23896 | 54_6530 | 13 | 8 | Bacteroidetes | Bacteroidia | Bacteroidales | Prevotellaceae | Prevotella | Prevotella copri |
| 23904 | 1_3607 | 10 | 5 | Bacteroidetes | Bacteroidia | Bacteroidales | Prevotellaceae | Prevotella | Prevotella copri |
| 23935 | 23_3406 | 16 | 5 | Bacteroidetes | Bacteroidia | Bacteroidales | Prevotellaceae | Prevotella | Prevotella copri |
| 23952 | 123_1520 | 14 | 5 | Bacteroidetes | Bacteroidia | Bacteroidales | Prevotellaceae | Prevotella | Prevotella copri |
| 23962 | 21_3772 | 15 | 6 | Bacteroidetes | Bacteroidia | Bacteroidales | Prevotellaceae | Prevotella | Prevotella copri |
| 23964 | 1_3722 | 23 | 11 | Bacteroidetes | Bacteroidia | Bacteroidales | Bacteroidaceae | Bacteroides | Bacteroides massiliensis |
| 24016 | 121_2210 | 10 | 6 | Bacteroidetes | Bacteroidia | Bacteroidales | Prevotellaceae | Prevotella | Prevotella sp. |
| 24060 | 4_4030 | 18 | 8 | Bacteroidetes | Bacteroidia | Bacteroidales | Prevotellaceae | Prevotella | Prevotella copri |
| 24077 | 1_1661 | 44 | 8 | Bacteroidetes | Bacteroidia | Bacteroidales | Prevotellaceae | Prevotella | Prevotella copri |
| 24078 | 121_5585 | 23 | 7 | Bacteroidetes | Bacteroidia | Bacteroidales | Prevotellaceae | Prevotella | Prevotella copri |
| 24080 | 21_6444 | 96 | 8 | Bacteroidetes | Bacteroidia | Bacteroidales | Prevotellaceae | Prevotella | Prevotella copri |
| 24084 | 54_7103 | 33 | 5 | Bacteroidetes | Bacteroidia | Bacteroidales | Prevotellaceae | Prevotella | Prevotella copri |
| 24089 | 8_1632 | 23 | 6 | Bacteroidetes | Bacteroidia | Bacteroidales | Prevotellaceae | Prevotella | Prevotella copri |
| 24093 | 112_646 | 6 | 5 | Bacteroidetes | Bacteroidia | Bacteroidales | Bacteroidaceae | Bacteroides | Bacteroides caccae |
| 24094 | 121_4596 | 35 | 10 | Bacteroidetes | Bacteroidia | Bacteroidales | Prevotellaceae | Prevotella | Prevotella copri |
| 24115 | 112_2154 | 24 | 7 | Bacteroidetes | Bacteroidia | Bacteroidales | Prevotellaceae | Prevotella | Prevotella copri |
| 24131 | 121_3291 | 7 | 5 | Bacteroidetes | Bacteroidia | Bacteroidales | Prevotellaceae | Prevotella | Prevotella copri |
| 24138 | 23_2409 | 18 | 6 | Bacteroidetes | Bacteroidia | Bacteroidales | Prevotellaceae | Prevotella | Prevotella copri |
| 24145 | 107_1653 | 17 | 8 | Bacteroidetes | Bacteroidia | Bacteroidales | Prevotellaceae | Prevotella | Prevotella copri |
| 24193 | 17_2781 | 35 | 9 | Bacteroidetes | Bacteroidia | Bacteroidales | Prevotellaceae | Prevotella | Prevotella copri |
| 24214 | 54_1716 | 11 | 5 | Bacteroidetes | Bacteroidia | Bacteroidales | Prevotellaceae | Prevotella | Prevotella copri |
| 24240 | 101_8011 | 129 | 11 | Bacteroidetes | Bacteroidia | Bacteroidales | Porphyromonadaceae | Coprobacter | Coprobacter fastidiosus |
| 24281 | 100_10762 | 35 | 8 | Bacteroidetes | Bacteroidia | Bacteroidales | Bacteroidaceae | Bacteroides | Bacteroides sp. |
| 24362 | 96_8617 | 11 | 7 | Bacteroidetes | Bacteroidia | Bacteroidales | Bacteroidaceae | Bacteroides | Bacteroides uniformis |
| 24372 | 1_1031 | 12 | 7 | Bacteroidetes | Bacteroidia | Bacteroidales | Prevotellaceae | Prevotella | Prevotella copri |
| 24435 | 131_9277 | 33 | 11 | Bacteroidetes | Bacteroidia | Bacteroidales | Bacteroidaceae | Bacteroides | Bacteroides eggerthii |
| 24436 | 78_8805 | 33 | 1 | Bacteroidetes | Bacteroidia | Bacteroidales | Bacteroidaceae | Bacteroides | Bacteroides massiliensis |
| 24447 | 65_8197 | 10 | 8 | Bacteroidetes | Bacteroidia | Bacteroidales | Bacteroidaceae | Bacteroides | Bacteroides vulgatus |
| 24448 | 75_501 | 8 | 5 | Bacteroidetes | Bacteroidia | Bacteroidales | Bacteroidaceae | Bacteroides | Bacteroides eggerthii |
| 24542 | 100_3561 | 51 | 8 | Bacteroidetes | Bacteroidia | Bacteroidales | Bacteroidaceae | Bacteroides | Bacteroides ovatus |
| 24593 | 119_5912 | 51 | 20 | Bacteroidetes | Bacteroidia | Bacteroidales | Porphyromonadaceae | Parabacteroides | Parabacteroides distasonis |
| 24600 | 75_2772 | 8 | 5 | Bacteroidetes | Bacteroidia | Bacteroidales | Bacteroidaceae | Bacteroides | Bacteroides uniformis |
| 24621 | 9_1819 | 16 | 12 | Bacteroidetes | Bacteroidia | Bacteroidales | Bacteroidaceae | Bacteroides | Bacteroides massiliensis |
| 24624 | 20_2415 | 27 | 3 | Bacteroidetes | Bacteroidia | Bacteroidales | Bacteroidaceae | Bacteroides | Bacteroides eggerthii |
| 24629 | 43_2414 | 80 | 10 | Bacteroidetes | Bacteroidia | Bacteroidales | Prevotellaceae | Prevotella | Prevotella copri |
| 24639 | 124_5833 | 18 | 12 | Bacteroidetes | Bacteroidia | Bacteroidales | Bacteroidaceae | Bacteroides | Bacteroides massiliensis |
| 24655 | 117_4158 | 9 | 7 | Firmicutes | Negativicutes | Selenomonadales | Veillonellaceae | Dialister | Dialister invisus |
| 24674 | 106_577 | 5 | 5 | Firmicutes | Negativicutes | Selenomonadales | Veillonellaceae | Dialister | Dialister invisus |
| 24679 | 49_2347 | 32 | 10 | Firmicutes | Negativicutes | Selenomonadales | Veillonellaceae | Dialister | Dialister invisus |
| 24682 | 117_94 | 7 | 5 | Firmicutes | Negativicutes | Selenomonadales | Veillonellaceae | Dialister | Dialister invisus |
| 24699 | 117_1945 | 16 | 8 | Firmicutes | Negativicutes | Selenomonadales | Veillonellaceae | Dialister | Dialister invisus |
| 24734 | 43_4079 | 8 | 6 | Firmicutes | Negativicutes | Selenomonadales | Veillonellaceae | Megasphaera | Megasphaera elsdenii |
| 24761 | 66_2568 | 97 | 13 | Firmicutes | Erysipelotrichia | Erysipelotrichales | Erysipelotrichaceae | Catenibacterium | Catenibacterium mitsuokai |
| 24764 | 1_1747 | 33 | 8 | Firmicutes | Erysipelotrichia | Erysipelotrichales | Erysipelotrichaceae | Catenibacterium | Catenibacterium mitsuokai |
| 24836 | 128_3121 | 8 | 6 | Bacteroidetes | Bacteroidia | Bacteroidales | Porphyromonadaceae | Parabacteroides | Parabacteroides merdae |
| 24846 | 40_4461 | 9 | 5 | Firmicutes | Negativicutes | Selenomonadales | Veillonellaceae | Megasphaera | Megasphaera elsdenii |
| 24881 | 53_2747 | 6 | 5 | Firmicutes | Negativicutes | Selenomonadales | Veillonellaceae | Dialister | Dialister invisus |
| 24901 | 117_4849 | 19 | 8 | Firmicutes | Clostridia | Clostridiales | Lachnospiraceae | Roseburia | Eubacterium rectale |
| 24912 | 60_6510 | 11 | 6 | Firmicutes | Clostridia | Clostridiales | Lachnospiraceae | Roseburia | Roseburia faecis |
| 25017 | 54_4664 | 44 | 8 | Firmicutes | Negativicutes | Selenomonadales | Veillonellaceae | Mitsuokella | Mitsuokella |
| 25019 | 66_5083 | 15 | 7 | Firmicutes | Negativicutes | Selenomonadales | Veillonellaceae | Mitsuokella | Mitsuokella |
| 25065 | 107_4631 | 13 | 7 | Firmicutes | Clostridia | Clostridiales | Ruminococcaceae | Papillibacter | Papillibacter cinnamivorans |
| 25078 | 18_5851 | 34 | 4 | Firmicutes | Clostridia | Clostridiales | Ruminococcaceae | Papillibacter | Papillibacter cinnamivorans |
| 25080 | 1_2108 | 218 | 56 | Firmicutes | Clostridia | Clostridiales | Ruminococcaceae | Papillibacter | Papillibacter cinnamivorans |
| 25092 | 124_5536 | 7 | 5 | Bacteroidetes | Bacteroidia | Bacteroidales | Porphyromonadaceae | Parabacteroides | Parabacteroides distasonis |
| 25148 | 36_1882 | 10 | 6 | Firmicutes | Negativicutes | Selenomonadales | Veillonellaceae | Dialister | Dialister invisus |
| 25150 | 130_6664 | 627 | 68 | Firmicutes | Clostridia | Clostridiales | Ruminococcaceae | Faecalibacterium | Faecalibacterium prausnitzii |
| 25156 | 32_2308 | 551 | 14 | Firmicutes | Clostridia | Clostridiales | Ruminococcaceae | Faecalibacterium | Faecalibacterium prausnitzii |
| 25159 | 33_1821 | 13 | 8 | Firmicutes | Clostridia | Clostridiales | Ruminococcaceae | Faecalibacterium | Faecalibacterium prausnitzii |
| 25164 | 42_6360 | 423 | 36 | Firmicutes | Clostridia | Clostridiales | Ruminococcaceae | Faecalibacterium | Faecalibacterium prausnitzii |
| 25167 | 4_504 | 6 | 6 | Firmicutes | Clostridia | Clostridiales | Ruminococcaceae | Faecalibacterium | Faecalibacterium prausnitzii |
| 25168 | 32_6571 | 32 | 6 | Firmicutes | Clostridia | Clostridiales | Ruminococcaceae | Faecalibacterium | Faecalibacterium prausnitzii |
| 25169 | 32_6892 | 212 | 41 | Firmicutes | Clostridia | Clostridiales | Ruminococcaceae | Faecalibacterium | Faecalibacterium prausnitzii |
| 25173 | 55_3946 | 5 | 5 | Firmicutes | Negativicutes | Selenomonadales | Veillonellaceae | Dialister | Dialister invisus |
| 25176 | 32_7023 | 40 | 9 | Firmicutes | Clostridia | Clostridiales | Ruminococcaceae | Faecalibacterium | Faecalibacterium prausnitzii |
| 25189 | 37_2437 | 5 | 5 | Firmicutes | Negativicutes | Selenomonadales | Veillonellaceae | Dialister | Dialister invisus |
| 25203 | 58_4522 | 66 | 5 | Proteobacteria | Betaproteobacteria | Burkholderiales | Sutterellaceae | Sutterella | Sutterella stercoricanis |
| 25223 | 44_1236 | 15 | 5 | Bacteroidetes | Bacteroidia | Bacteroidales | Bacteroidaceae | Bacteroides | Bacteroides dorei |
| 25265 | 63_4195 | 8 | 5 | Firmicutes | Negativicutes | Selenomonadales | Veillonellaceae | Dialister | Dialister invisus |
| 25267 | 48_1359 | 7 | 5 | Firmicutes | Negativicutes | Selenomonadales | Veillonellaceae | Dialister | Dialister invisus |
| 25280 | 110_1008 | 74 | 9 | Bacteroidetes | Bacteroidia | Bacteroidales | Bacteroidaceae | Bacteroides | Bacteroides sp. |
| 25324 | 130_3182 | 15 | 8 | Firmicutes | Negativicutes | Selenomonadales | Veillonellaceae | Dialister | Dialister invisus |
| 25329 | 84_5355 | 384 | 66 | Firmicutes | Clostridia | Clostridiales | Lachnospiraceae | Blautia | Blautia |
| 25356 | 48_1485 | 16 | 6 | Firmicutes | Negativicutes | Selenomonadales | Veillonellaceae | Dialister | Dialister invisus |
| 25374 | 125_493 | 25 | 11 | Firmicutes | Clostridia | Clostridiales | Clostridiaceae | Clostridium | Clostridium sp. |
| 25402 | 18_6150 | 7 | 6 | Firmicutes | Negativicutes | Selenomonadales | Veillonellaceae | Dialister | Dialister invisus |
| 25433 | 90_3910 | 13 | 8 | Bacteroidetes | Bacteroidia | Bacteroidales | Bacteroidaceae | Bacteroides | Bacteroides uniformis |
| 25438 | 30_4788 | 10 | 6 | Bacteroidetes | Bacteroidia | Bacteroidales | Bacteroidaceae | Bacteroides | Bacteroides uniformis |
| 25442 | 65_1781 | 16 | 5 | Bacteroidetes | Bacteroidia | Bacteroidales | Bacteroidaceae | Bacteroides | Bacteroides vulgatus |
| 25448 | 114_5329 | 26 | 5 | Bacteroidetes | Bacteroidia | Bacteroidales | Bacteroidaceae | Bacteroides | Bacteroides uniformis |
| 25470 | 48_4989 | 21 | 4 | Bacteroidetes | Bacteroidia | Bacteroidales | Prevotellaceae | Prevotella | Prevotella copri |
| 25476 | 71_8151 | 2230 | 22 | Proteobacteria | Gammaproteobacteria | Enterobacteriales | Enterobacteriaceae | Escherichia/Shigella | Escherichia/Shigella |
| 25487 | 47_4278 | 44 | 2 | Proteobacteria | Gammaproteobacteria | Enterobacteriales | Enterobacteriaceae | Escherichia/Shigella | Escherichia/Shigella |
| 25514 | 71_6526 | 15 | 10 | Proteobacteria | Deltaproteobacteria | Desulfovibrionales | Desulfovibrionaceae | Desulfovibrio | Desulfovibrio vulgaris |
| 25520 | 60_5001 | 12 | 8 | Bacteroidetes | Bacteroidia | Bacteroidales | Bacteroidaceae | Bacteroides | Bacteroides sp. |
| 25526 | 19_1364 | 237 | 40 | Bacteroidetes | Bacteroidia | Bacteroidales | Porphyromonadaceae | Odoribacter | Odoribacter splanchnicus |
| 25531 | 102_5587 | 28 | 14 | Bacteroidetes | Bacteroidia | Bacteroidales | Bacteroidaceae | Bacteroides | Bacteroides uniformis |
| 25559 | 51_1104 | 15 | 5 | Bacteroidetes | Bacteroidia | Bacteroidales | Prevotellaceae | Prevotella | Prevotella sp. |
| 25565 | 51_1409 | 9 | 5 | Bacteroidetes | Bacteroidia | Bacteroidales | Porphyromonadaceae | Barnesiella | Barnsiella intestinihominis |
| 25566 | 26_839 | 9 | 6 | Bacteroidetes | Bacteroidia | Bacteroidales | Bacteroidaceae | Bacteroides | Bacteroides sp. |
| 25580 | 39_824 | 11 | 7 | Bacteroidetes | Bacteroidia | Bacteroidales | Bacteroidaceae | Bacteroides | Bacteroides sp. |
| 25626 | 38_6760 | 53 | 14 | Bacteroidetes | Bacteroidia | Bacteroidales | Porphyromonadaceae | Butyricimonas | Butyricimonas virosa |
| 25640 | 98_2679 | 618 | 7 | Bacteroidetes | Bacteroidia | Bacteroidales | Porphyromonadaceae | Barnesiella | Barnesiella |
| 25693 | 40_3258 | 22 | 12 | Bacteroidetes | Bacteroidia | Bacteroidales | Bacteroidaceae | Bacteroides | Bacteroides uniformis |
| 25716 | 69_4422 | 35 | 23 | Bacteroidetes | Bacteroidia | Bacteroidales | Porphyromonadaceae | Parabacteroides | Parabacteroides merdae |
| 25718 | 1_3262 | 19 | 13 | Bacteroidetes | Bacteroidia | Bacteroidales | Bacteroidaceae | Bacteroides | Bacteroides sp. |
| 25719 | 39_6344 | 10 | 5 | Bacteroidetes | Bacteroidia | Bacteroidales | Bacteroidaceae | Bacteroides | Bacteroides eggerthii |
| 25722 | 122_670 | 24 | 9 | Bacteroidetes | Bacteroidia | Bacteroidales | Bacteroidaceae | Bacteroides | Bacteroides dorei |
| 25725 | 19_5157 | 8 | 6 | Bacteroidetes | Bacteroidia | Bacteroidales | Porphyromonadaceae | Parabacteroides | Parabacteroides merdae |
| 25737 | 90_3968 | 12 | 6 | Bacteroidetes | Bacteroidia | Bacteroidales | Bacteroidaceae | Bacteroides | Bacteroides uniformis |
| 25741 | 19_3305 | 9 | 8 | Bacteroidetes | Bacteroidia | Bacteroidales | Bacteroidaceae | Bacteroides | Bacteroides uniformis |
| 25754 | 124_3409 | 5 | 5 | Bacteroidetes | Bacteroidia | Bacteroidales | Bacteroidaceae | Bacteroides | Bacteroides sp. |
| 25757 | 29_1763 | 7 | 5 | Bacteroidetes | Bacteroidia | Bacteroidales | Bacteroidaceae | Bacteroides | Bacteroides uniformis |
| 25777 | 10_6530 | 7 | 5 | Bacteroidetes | Bacteroidia | Bacteroidales | Bacteroidaceae | Bacteroides | Bacteroides uniformis |
| 25796 | 58_6896 | 21 | 5 | Bacteroidetes | Bacteroidia | Bacteroidales | Bacteroidaceae | Bacteroides | Bacteroides plebeius |
| 25798 | 81_3521 | 7 | 5 | Bacteroidetes | Bacteroidia | Bacteroidales | Bacteroidaceae | Bacteroides | Bacteroides plebeius |
| 25826 | 20_605 | 20 | 3 | Bacteroidetes | Bacteroidia | Bacteroidales | Bacteroidaceae | Bacteroides | Bacteroides eggerthii |
| 25830 | 48_6247 | 21 | 11 | Bacteroidetes | Bacteroidia | Bacteroidales | Prevotellaceae | Prevotella | Prevotella copri |
| 25834 | 20_8570 | 209 | 39 | Bacteroidetes | Bacteroidia | Bacteroidales | Porphyromonadaceae | Odoribacter | Odoribacter splanchnicus |
| 25854 | 71_3512 | 57 | 23 | Bacteroidetes | Bacteroidia | Bacteroidales | Bacteroidaceae | Bacteroides | Bacteroides vulgatus |
| 25857 | 69_3994 | 15 | 6 | Bacteroidetes | Bacteroidia | Bacteroidales | Bacteroidaceae | Bacteroides | Bacteroides uniformis |
| 25881 | 93_3072 | 67 | 8 | Bacteroidetes | Bacteroidia | Bacteroidales | Bacteroidaceae | Bacteroides | Bacteroides intestinalis |
| 25886 | 108_6945 | 21 | 1 | Bacteroidetes | Bacteroidia | Bacteroidales | Bacteroidaceae | Bacteroides | Bacteroides finegoldii |
| 25890 | 39_5639 | 31 | 3 | Bacteroidetes | Bacteroidia | Bacteroidales | Bacteroidaceae | Bacteroides | Bacteroides uniformis |
| 25898 | 79_4754 | 20 | 6 | Bacteroidetes | Bacteroidia | Bacteroidales | Bacteroidaceae | Bacteroides | Bacteroides sp. |
| 25901 | 108_7228 | 7 | 5 | Bacteroidetes | Bacteroidia | Bacteroidales | Prevotellaceae | Prevotella | Prevotella copri |
| 25913 | 40_1925 | 56 | 13 | Bacteroidetes | Bacteroidia | Bacteroidales | Bacteroidaceae | Bacteroides | Bacteroides uniformis |
| 25920 | 15_2724 | 11 | 9 | Bacteroidetes | Bacteroidia | Bacteroidales | Rikenellaceae | Alistipes | Alistipes finegoldii |
| 25927 | 26_5053 | 6 | 5 | Bacteroidetes | Bacteroidia | Bacteroidales | Rikenellaceae | Alistipes | Alistipes sp. |
| 25930 | 63_1011 | 54 | 21 | Bacteroidetes | Bacteroidia | Bacteroidales | Rikenellaceae | Alistipes | Alistipes sp. |
| 25931 | 63_2235 | 844 | 52 | Bacteroidetes | Bacteroidia | Bacteroidales | Rikenellaceae | Alistipes | Alistipes sp. |
| 25934 | 63_2362 | 11 | 9 | Bacteroidetes | Bacteroidia | Bacteroidales | Rikenellaceae | Alistipes | Alistipes sp. |
| 25953 | 75_2985 | 6 | 6 | Bacteroidetes | Bacteroidia | Bacteroidales | Bacteroidaceae | Bacteroides | Bacteroides eggerthii |
| 25966 | 6_1543 | 10 | 9 | Bacteroidetes | Bacteroidia | Bacteroidales | Rikenellaceae | Alistipes | Alistipes sp. |
| 25969 | 37_5904 | 24 | 15 | Bacteroidetes | Bacteroidia | Bacteroidales | Rikenellaceae | Alistipes | Alistipes finegoldii |
| 26045 | 56_94 | 22 | 5 | Bacteroidetes | Bacteroidia | Bacteroidales | Bacteroidaceae | Bacteroides | Bacteroides ovatus |
| 26054 | 15_2554 | 19 | 11 | Bacteroidetes | Bacteroidia | Bacteroidales | Bacteroidaceae | Bacteroides | Bacteroides ovatus |
| 26057 | 79_1384 | 56 | 5 | Bacteroidetes | Bacteroidia | Bacteroidales | Bacteroidaceae | Bacteroides | Bacteroides ovatus |
| 26062 | 15_741 | 10 | 5 | Bacteroidetes | Bacteroidia | Bacteroidales | Bacteroidaceae | Bacteroides | Bacteroides ovatus |
| 26101 | 123_6794 | 6 | 5 | Bacteroidetes | Bacteroidia | Bacteroidales | Prevotellaceae | Prevotella | Prevotella copri |
| 26116 | 100_9262 | 27 | 6 | Bacteroidetes | Bacteroidia | Bacteroidales | Bacteroidaceae | Bacteroides | Bacteroides ovatus |
| 26121 | 4_352 | 9 | 8 | Bacteroidetes | Bacteroidia | Bacteroidales | Prevotellaceae | Prevotella | Prevotella copri |
| 26122 | 107_3916 | 12 | 6 | Bacteroidetes | Bacteroidia | Bacteroidales | Prevotellaceae | Prevotella | Prevotella copri |
| 26135 | 60_5671 | 44 | 9 | Bacteroidetes | Bacteroidia | Bacteroidales | Bacteroidaceae | Bacteroides | Bacteroides massiliensis |
| 26142 | 20_307 | 33 | 9 | Bacteroidetes | Bacteroidia | Bacteroidales | Bacteroidaceae | Bacteroides | Bacteroides eggerthii |
| 26149 | 94_10371 | 18 | 9 | Bacteroidetes | Bacteroidia | Bacteroidales | Bacteroidaceae | Bacteroides | Bacteroides ovatus |
| 26179 | 105_731 | 27 | 10 | Bacteroidetes | Bacteroidia | Bacteroidales | Bacteroidaceae | Bacteroides | Bacteroides ovatus |
| 26181 | 7_2197 | 27 | 2 | Bacteroidetes | Bacteroidia | Bacteroidales | Bacteroidaceae | Bacteroides | Bacteroides ovatus |
| 26197 | 94_10992 | 55 | 4 | Bacteroidetes | Bacteroidia | Bacteroidales | Bacteroidaceae | Bacteroides | Bacteroides thetaiotaomicron |
| 26239 | 90_1173 | 12 | 6 | Bacteroidetes | Bacteroidia | Bacteroidales | Bacteroidaceae | Bacteroides | Bacteroides uniformis |
| 26314 | 73_1115 | 7 | 5 | Bacteroidetes | Bacteroidia | Bacteroidales | Porphyromonadaceae | Parabacteroides | Parabacteroides merdae |
| 26323 | 105_2427 | 31 | 9 | Bacteroidetes | Bacteroidia | Bacteroidales | Bacteroidaceae | Bacteroides | Bacteroides massiliensis |
| 26386 | 20_357 | 8 | 5 | Bacteroidetes | Bacteroidia | Bacteroidales | Bacteroidaceae | Bacteroides | Bacteroides caccae |
| 26430 | 105_6999 | 21 | 5 | Bacteroidetes | Bacteroidia | Bacteroidales | Bacteroidaceae | Bacteroides | Bacteroides thetaiotaomicron |
| 26435 | 39_2893 | 8 | 6 | Bacteroidetes | Bacteroidia | Bacteroidales | Bacteroidaceae | Bacteroides | Bacteroides eggerthii |
| 26454 | 130_5645 | 13 | 8 | Bacteroidetes | Bacteroidia | Bacteroidales | Bacteroidaceae | Bacteroides | Bacteroides uniformis |
| 26458 | 107_4423 | 10 | 5 | Bacteroidetes | Bacteroidia | Bacteroidales | Bacteroidaceae | Bacteroides | Bacteroides caccae |
| 26521 | 88_1768 | 10 | 5 | Bacteroidetes | Bacteroidia | Bacteroidales | Bacteroidaceae | Bacteroides | Bacteroides fragilis |
| 26535 | 105_3751 | 14 | 7 | Bacteroidetes | Bacteroidia | Bacteroidales | Bacteroidaceae | Bacteroides | Bacteroides massiliensis |
| 26541 | 105_6700 | 11 | 5 | Bacteroidetes | Bacteroidia | Bacteroidales | Bacteroidaceae | Bacteroides | Bacteroides sp. |
| 26544 | 49_1279 | 49 | 20 | Bacteroidetes | Bacteroidia | Bacteroidales | Bacteroidaceae | Bacteroides | Bacteroides massiliensis |
| 26546 | 60_3724 | 10 | 5 | Bacteroidetes | Bacteroidia | Bacteroidales | Bacteroidaceae | Bacteroides | Bacteroides uniformis |
| 26555 | 71_782 | 2594 | 66 | Bacteroidetes | Bacteroidia | Bacteroidales | Bacteroidaceae | Bacteroides | Bacteroides massiliensis |
| 26560 | 94_5530 | 50 | 5 | Bacteroidetes | Bacteroidia | Bacteroidales | Bacteroidaceae | Bacteroides | Bacteroides thetaiotaomicron |
| 26566 | 97_4140 | 6 | 6 | Bacteroidetes | Bacteroidia | Bacteroidales | Porphyromonadaceae | Barnesiella | Barnsiella intestinihominis |
| 26575 | 17_1646 | 7 | 6 | Bacteroidetes | Bacteroidia | Bacteroidales | Bacteroidaceae | Bacteroides | Bacteroides massiliensis |
| 26582 | 96_6011 | 110 | 26 | Bacteroidetes | Bacteroidia | Bacteroidales | Bacteroidaceae | Bacteroides | Bacteroides massiliensis |
| 26583 | 9_1397 | 9 | 5 | Bacteroidetes | Bacteroidia | Bacteroidales | Bacteroidaceae | Bacteroides | Bacteroides massiliensis |
| 26586 | 50_880 | 11 | 5 | Bacteroidetes | Bacteroidia | Bacteroidales | Bacteroidaceae | Bacteroides | Bacteroides uniformis |
| 26601 | 115_2180 | 257 | 26 | Bacteroidetes | Bacteroidia | Bacteroidales | Bacteroidaceae | Bacteroides | Bacteroides massiliensis |
| 26605 | 57_468 | 8 | 6 | Bacteroidetes | Bacteroidia | Bacteroidales | Bacteroidaceae | Bacteroides | Bacteroides massiliensis |
| 26623 | 107_646 | 7 | 5 | Bacteroidetes | Bacteroidia | Bacteroidales | Prevotellaceae | Prevotella | Prevotella copri |
| 26638 | 105_3727 | 68 | 15 | Bacteroidetes | Bacteroidia | Bacteroidales | Bacteroidaceae | Bacteroides | Bacteroides massiliensis |
| 26650 | 106_560 | 6 | 5 | Bacteroidetes | Bacteroidia | Bacteroidales | Bacteroidaceae | Bacteroides | Bacteroides sp. |
| 26703 | 39_1189 | 10 | 5 | Bacteroidetes | Bacteroidia | Bacteroidales | Bacteroidaceae | Bacteroides | Bacteroides eggerthii |
| 26708 | 20_2144 | 22 | 5 | Bacteroidetes | Bacteroidia | Bacteroidales | Bacteroidaceae | Bacteroides | Bacteroides eggerthii |
| 26711 | 100_3424 | 29 | 8 | Bacteroidetes | Bacteroidia | Bacteroidales | Bacteroidaceae | Bacteroides | Bacteroides eggerthii |
| 26727 | 20_4507 | 40 | 4 | Bacteroidetes | Bacteroidia | Bacteroidales | Bacteroidaceae | Bacteroides | Bacteroides sp. |
| 26741 | 63_2386 | 28 | 8 | Bacteroidetes | Bacteroidia | Bacteroidales | Bacteroidaceae | Bacteroides | Bacteroides eggerthii |
| 26750 | 97_842 | 31 | 6 | Bacteroidetes | Bacteroidia | Bacteroidales | Porphyromonadaceae | Barnesiella | Barnesiella intestinihominis |
| 26755 | 63_5252 | 35 | 10 | Bacteroidetes | Bacteroidia | Bacteroidales | Bacteroidaceae | Bacteroides | Bacteroides finegoldii |
| 26805 | 120_1407 | 17 | 7 | Bacteroidetes | Bacteroidia | Bacteroidales | Bacteroidaceae | Bacteroides | Bacteroides finegoldii |
| 26811 | 86_3230 | 17 | 6 | Bacteroidetes | Bacteroidia | Bacteroidales | Bacteroidaceae | Bacteroides | Bacteroides uniformis |
| 26812 | 9_5751 | 8 | 5 | Bacteroidetes | Bacteroidia | Bacteroidales | Porphyromonadaceae | Parabacteroides | Parabacteroides merdae |
| 26848 | 69_3722 | 20 | 6 | Bacteroidetes | Bacteroidia | Bacteroidales | Bacteroidaceae | Bacteroides | Bacteroides uniformis |
| 26864 | 131_9274 | 31 | 6 | Bacteroidetes | Bacteroidia | Bacteroidales | Bacteroidaceae | Bacteroides | Bacteroides uniformis |
| 26875 | 111_7021 | 8 | 5 | Bacteroidetes | Bacteroidia | Bacteroidales | Bacteroidaceae | Bacteroides | Bacteroides uniformis |
| 26884 | 131_7984 | 46 | 11 | Bacteroidetes | Bacteroidia | Bacteroidales | Bacteroidaceae | Bacteroides | Bacteroides uniformis |
| 26885 | 69_3809 | 18 | 14 | Bacteroidetes | Bacteroidia | Bacteroidales | Bacteroidaceae | Bacteroides | Bacteroides sp. |
| 26925 | 63_5122 | 10 | 5 | Bacteroidetes | Bacteroidia | Bacteroidales | Bacteroidaceae | Bacteroides | Bacteroides uniformis |
| 26941 | 101_2276 | 12 | 8 | Bacteroidetes | Bacteroidia | Bacteroidales | Porphyromonadaceae | Parabacteroides | Parabacteroides merdae |
| 26944 | 9_5236 | 9 | 5 | Bacteroidetes | Bacteroidia | Bacteroidales | Bacteroidaceae | Bacteroides | Bacteroides uniformis |
| 26951 | 129_1231 | 7 | 5 | Bacteroidetes | Bacteroidia | Bacteroidales | Bacteroidaceae | Bacteroides | Bacteroides uniformis |
| 26961 | 122_1945 | 13 | 6 | Bacteroidetes | Bacteroidia | Bacteroidales | Bacteroidaceae | Bacteroides | Bacteroides intestinalis |
| 26966 | 9_935 | 7 | 5 | Bacteroidetes | Bacteroidia | Bacteroidales | Prevotellaceae | Prevotella | Prevotella sp. |
| 26974 | 99_3892 | 6 | 5 | Bacteroidetes | Bacteroidia | Bacteroidales | Bacteroidaceae | Bacteroides | Bacteroides uniformis |
| 26976 | 86_3422 | 5 | 5 | Bacteroidetes | Bacteroidia | Bacteroidales | Bacteroidaceae | Bacteroides | Bacteroides uniformis |
| 26993 | 75_1754 | 11 | 6 | Bacteroidetes | Bacteroidia | Bacteroidales | Bacteroidaceae | Bacteroides | Bacteroides uniformis |
| 26998 | 19_7015 | 8 | 7 | Bacteroidetes | Bacteroidia | Bacteroidales | Bacteroidaceae | Bacteroides | Bacteroides sp. |
| 27013 | 120_1773 | 7 | 6 | Bacteroidetes | Bacteroidia | Bacteroidales | Bacteroidaceae | Bacteroides | Bacteroides eggerthii |
| 27018 | 7_2316 | 29 | 7 | Bacteroidetes | Bacteroidia | Bacteroidales | Prevotellaceae | Prevotella | Prevotella stercorea |
| 27031 | 90_9427 | 37 | 6 | Bacteroidetes | Bacteroidia | Bacteroidales | Bacteroidaceae | Bacteroides | Bacteroides massiliensis |
| 27040 | 19_6112 | 8 | 6 | Bacteroidetes | Bacteroidia | Bacteroidales | Bacteroidaceae | Bacteroides | Bacteroides uniformis |
| 27093 | 60_2960 | 12 | 9 | Bacteroidetes | Bacteroidia | Bacteroidales | Bacteroidaceae | Bacteroides | Bacteroides uniformis |
| 27094 | 60_1423 | 6 | 5 | Bacteroidetes | Bacteroidia | Bacteroidales | Bacteroidaceae | Bacteroides | Bacteroides sp. |
| 27099 | 9_1055 | 6 | 5 | Bacteroidetes | Bacteroidia | Bacteroidales | Prevotellaceae | Prevotella | Prevotella sp. |
| 27100 | 39_2443 | 21 | 13 | Bacteroidetes | Bacteroidia | Bacteroidales | Bacteroidaceae | Bacteroides | Bacteroides uniformis |
| 27106 | 24_5380 | 7 | 6 | Bacteroidetes | Bacteroidia | Bacteroidales | Bacteroidaceae | Bacteroides | Bacteroides uniformis |
| 27114 | 107_1886 | 8 | 5 | Bacteroidetes | Bacteroidia | Bacteroidales | Bacteroidaceae | Bacteroides | Bacteroides massiliensis |
| 27116 | 60_3575 | 10 | 5 | Bacteroidetes | Bacteroidia | Bacteroidales | Bacteroidaceae | Bacteroides | Bacteroides sp. |
| 27126 | 39_2621 | 19 | 12 | Bacteroidetes | Bacteroidia | Bacteroidales | Bacteroidaceae | Bacteroides | Bacteroides sp. |
| 27132 | 124_6583 | 9 | 6 | Bacteroidetes | Bacteroidia | Bacteroidales | Bacteroidaceae | Bacteroides | Bacteroides sp. |
| 27140 | 9_5616 | 13 | 5 | Bacteroidetes | Bacteroidia | Bacteroidales | Prevotellaceae | Prevotella | Prevotella sp. |
| 27141 | 116_1138 | 5 | 5 | Bacteroidetes | Bacteroidia | Bacteroidales | Bacteroidaceae | Bacteroides | Bacteroides sp. |
| 27205 | 102_5113 | 8 | 5 | Bacteroidetes | Bacteroidia | Bacteroidales | Bacteroidaceae | Bacteroides | Bacteroides sp. |
| 27208 | 105_6915 | 29 | 5 | Bacteroidetes | Bacteroidia | Bacteroidales | Bacteroidaceae | Bacteroides | Bacteroides thetaiotaomicron |
| 27233 | 105_2606 | 13 | 7 | Bacteroidetes | Bacteroidia | Bacteroidales | Bacteroidaceae | Bacteroides | Bacteroides massiliensis |
| 27243 | 20_923 | 6 | 5 | Bacteroidetes | Bacteroidia | Bacteroidales | Bacteroidaceae | Bacteroides | Bacteroides massiliensis |
| 27256 | 120_3391 | 5 | 5 | Bacteroidetes | Bacteroidia | Bacteroidales | Bacteroidaceae | Bacteroides | Bacteroides uniformis |
| 27261 | 131_4612 | 24 | 10 | Bacteroidetes | Bacteroidia | Bacteroidales | Bacteroidaceae | Bacteroides | Bacteroides uniformis |
| 27262 | 119_6011 | 16 | 11 | Bacteroidetes | Bacteroidia | Bacteroidales | Bacteroidaceae | Bacteroides | Bacteroides sp. |
| 27263 | 10_6638 | 27 | 9 | Bacteroidetes | Bacteroidia | Bacteroidales | Rikenellaceae | Alistipes | Alistipes sp. |
| 27264 | 60_7108 | 11 | 5 | Bacteroidetes | Bacteroidia | Bacteroidales | Bacteroidaceae | Bacteroides | Bacteroides massiliensis |
| 27267 | 39_2476 | 7 | 5 | Bacteroidetes | Bacteroidia | Bacteroidales | Bacteroidaceae | Bacteroides | Bacteroides sp. |
| 27274 | 40_2357 | 8 | 6 | Bacteroidetes | Bacteroidia | Bacteroidales | Bacteroidaceae | Bacteroides | Bacteroides uniformis |
| 27287 | 105_6073 | 63 | 17 | Bacteroidetes | Bacteroidia | Bacteroidales | Bacteroidaceae | Bacteroides | Bacteroides massiliensis |
| 27302 | 57_534 | 15 | 11 | Bacteroidetes | Bacteroidia | Bacteroidales | Bacteroidaceae | Bacteroides | Bacteroides uniformis |
| 27307 | 68_8915 | 7 | 5 | Bacteroidetes | Bacteroidia | Bacteroidales | Bacteroidaceae | Bacteroides | Bacteroides sp. |
| 27309 | 4_3574 | 9 | 7 | Bacteroidetes | Bacteroidia | Bacteroidales | Bacteroidaceae | Bacteroides | Bacteroides massiliensis |
| 27335 | 114_6989 | 51 | 13 | Bacteroidetes | Bacteroidia | Bacteroidales | Porphyromonadaceae | Parabacteroides | Parabacteroides distasonis |
| 27340 | 3_487 | 8 | 6 | Bacteroidetes | Bacteroidia | Bacteroidales | Bacteroidaceae | Bacteroides | Bacteroides sp. |
| 27352 | 42_2311 | 26 | 13 | Bacteroidetes | Bacteroidia | Bacteroidales | Bacteroidaceae | Bacteroides | Bacteroides vulgatus |
| 27353 | 65_7235 | 11 | 7 | Bacteroidetes | Bacteroidia | Bacteroidales | Bacteroidaceae | Bacteroides | Bacteroides uniformis |
| 27369 | 65_4718 | 120 | 19 | Bacteroidetes | Bacteroidia | Bacteroidales | Bacteroidaceae | Bacteroides | Bacteroides vulgatus |
| 27371 | 12_4196 | 14 | 9 | Bacteroidetes | Bacteroidia | Bacteroidales | Bacteroidaceae | Bacteroides | Bacteroides vulgatus |
| 27376 | 12_1008 | 33 | 18 | Bacteroidetes | Bacteroidia | Bacteroidales | Porphyromonadaceae | Parabacteroides | Parabacteroides distasonis |
| 27377 | 65_1308 | 465 | 60 | Bacteroidetes | Bacteroidia | Bacteroidales | Porphyromonadaceae | Parabacteroides | Parabacteroides distasonis |
| 27379 | 50_2228 | 14 | 10 | Bacteroidetes | Bacteroidia | Bacteroidales | Porphyromonadaceae | Parabacteroides | Parabacteroides distasonis |
| 27394 | 91_5443 | 71 | 16 | Bacteroidetes | Bacteroidia | Bacteroidales | Porphyromonadaceae | Parabacteroides | Parabacteroides distasonis |
| 27406 | 114_1474 | 28 | 8 | Bacteroidetes | Bacteroidia | Bacteroidales | Porphyromonadaceae | Parabacteroides | Parabacteroides distasonis |
| 27408 | 32_9 | 14 | 13 | Bacteroidetes | Bacteroidia | Bacteroidales | Porphyromonadaceae | Parabacteroides | Parabacteroides distasonis |
| 27410 | 44_2288 | 484 | 36 | Bacteroidetes | Bacteroidia | Bacteroidales | Porphyromonadaceae | Parabacteroides | Parabacteroides distasonis |
| 27418 | 9_1369 | 14 | 9 | Bacteroidetes | Bacteroidia | Bacteroidales | Bacteroidaceae | Bacteroides | Bacteroides uniformis |
| 27425 | 76_5342 | 14 | 7 | Bacteroidetes | Bacteroidia | Bacteroidales | Bacteroidaceae | Bacteroides | Bacteroides fragilis |
| 27428 | 114_7841 | 25 | 11 | Bacteroidetes | Bacteroidia | Bacteroidales | Porphyromonadaceae | Parabacteroides | Parabacteroides distasonis |
| 27432 | 76_4993 | 18 | 9 | Bacteroidetes | Bacteroidia | Bacteroidales | Bacteroidaceae | Bacteroides | Bacteroides uniformis |
| 27464 | 38_3176 | 6 | 5 | Bacteroidetes | Bacteroidia | Bacteroidales | Porphyromonadaceae | Parabacteroides | Parabacteroides distasonis |
| 27465 | 61_4440 | 62 | 16 | Bacteroidetes | Bacteroidia | Bacteroidales | Porphyromonadaceae | Parabacteroides | Parabacteroides distasonis |
| 27468 | 61_247 | 22 | 8 | Bacteroidetes | Bacteroidia | Bacteroidales | Porphyromonadaceae | Parabacteroides | Parabacteroides distasonis |
| 27473 | 80_5200 | 7 | 5 | Bacteroidetes | Bacteroidia | Bacteroidales | Bacteroidaceae | Bacteroides | Bacteroides uniformis |
| 27481 | 60_3145 | 8 | 6 | Bacteroidetes | Bacteroidia | Bacteroidales | Bacteroidaceae | Bacteroides | Bacteroides sp. |
| 27499 | 50_1569 | 10 | 5 | Bacteroidetes | Bacteroidia | Bacteroidales | Porphyromonadaceae | Parabacteroides | Parabacteroides distasonis |
| 27501 | 114_6841 | 52 | 13 | Bacteroidetes | Bacteroidia | Bacteroidales | Porphyromonadaceae | Parabacteroides | Parabacteroides distasonis |
| 27519 | 58_6114 | 66 | 2 | Bacteroidetes | Bacteroidia | Bacteroidales | Bacteroidaceae | Bacteroides | Bacteroides plebeius |
| 27527 | 40_397 | 33 | 11 | Bacteroidetes | Bacteroidia | Bacteroidales | Bacteroidaceae | Bacteroides | Bacteroides eggerthii |
| 27528 | 130_3578 | 9 | 6 | Bacteroidetes | Bacteroidia | Bacteroidales | Bacteroidaceae | Bacteroides | Bacteroides vulgatus |
| 27534 | 120_698 | 30 | 11 | Bacteroidetes | Bacteroidia | Bacteroidales | Bacteroidaceae | Bacteroides | Bacteroides uniformis |
| 27562 | 122_611 | 29 | 10 | Bacteroidetes | Bacteroidia | Bacteroidales | Bacteroidaceae | Bacteroides | Bacteroides uniformis |
| 27566 | 122_3064 | 7 | 5 | Bacteroidetes | Bacteroidia | Bacteroidales | Bacteroidaceae | Bacteroides | Bacteroides uniformis |
| 27569 | 67_2517 | 8 | 7 | Bacteroidetes | Bacteroidia | Bacteroidales | Bacteroidaceae | Bacteroides | Bacteroides uniformis |
| 27571 | 22_356 | 8 | 5 | Bacteroidetes | Bacteroidia | Bacteroidales | Bacteroidaceae | Bacteroides | Bacteroides uniformis |
| 27576 | 32_3060 | 20 | 7 | Bacteroidetes | Bacteroidia | Bacteroidales | Bacteroidaceae | Bacteroides | Bacteroides intestinalis |
| 27581 | 105_3289 | 5 | 5 | Bacteroidetes | Bacteroidia | Bacteroidales | Bacteroidaceae | Bacteroides | Bacteroides uniformis |
| 27604 | 122_599 | 7 | 5 | Bacteroidetes | Bacteroidia | Bacteroidales | Bacteroidaceae | Bacteroides | Bacteroides intestinalis |
| 27640 | 117_1586 | 37 | 5 | Bacteroidetes | Bacteroidia | Bacteroidales | Prevotellaceae | Prevotella | Prevotella stercorea |
| 27644 | 71_6932 | 192 | 46 | Bacteroidetes | Bacteroidia | Bacteroidales | Bacteroidaceae | Bacteroides | Bacteroides vulgatus |
| 27647 | 65_2995 | 11 | 5 | Bacteroidetes | Bacteroidia | Bacteroidales | Bacteroidaceae | Bacteroides | Bacteroides fragilis |
| 27661 | 75_2116 | 12 | 5 | Bacteroidetes | Bacteroidia | Bacteroidales | Bacteroidaceae | Bacteroides | Bacteroides eggerthii |
| 27663 | 12_4182 | 8 | 5 | Bacteroidetes | Bacteroidia | Bacteroidales | Porphyromonadaceae | Parabacteroides | Parabacteroides distasonis |
| 27673 | 123_4012 | 32 | 4 | Bacteroidetes | Bacteroidia | Bacteroidales | Bacteroidaceae | Bacteroides | Bacteroides uniformis |
| 27678 | 132_2984 | 16 | 9 | Bacteroidetes | Bacteroidia | Bacteroidales | Bacteroidaceae | Bacteroides | Bacteroides uniformis |
| 27679 | 114_7428 | 7 | 6 | Bacteroidetes | Bacteroidia | Bacteroidales | Bacteroidaceae | Bacteroides | Bacteroides thetaiotaomicron |
| 27697 | 41_8070 | 6 | 5 | Bacteroidetes | Bacteroidia | Bacteroidales | Bacteroidaceae | Bacteroides | Bacteroides vulgatus |
| 27699 | 12_1287 | 311 | 44 | Bacteroidetes | Bacteroidia | Bacteroidales | Bacteroidaceae | Bacteroides | Bacteroides vulgatus |
| 27702 | 1_2831 | 64 | 21 | Bacteroidetes | Bacteroidia | Bacteroidales | Bacteroidaceae | Bacteroides | Bacteroides vulgatus |
| 27720 | 108_6504 | 13 | 10 | Bacteroidetes | Bacteroidia | Bacteroidales | Bacteroidaceae | Bacteroides | Bacteroides sp. |
| 27722 | 57_489 | 11 | 10 | Bacteroidetes | Bacteroidia | Bacteroidales | Bacteroidaceae | Bacteroides | Bacteroides vulgatus |
| 27724 | 115_2429 | 6 | 5 | Bacteroidetes | Bacteroidia | Bacteroidales | Prevotellaceae | Prevotella | Prevotella copri |
| 27730 | 9_123 | 7 | 6 | Bacteroidetes | Bacteroidia | Bacteroidales | Prevotellaceae | Prevotella | Prevotella ruminicola |
| 27740 | 107_3552 | 20 | 13 | Bacteroidetes | Bacteroidia | Bacteroidales | Rikenellaceae | Alistipes | Alistipes putredinis |
| 27772 | 12_1681 | 9 | 5 | Bacteroidetes | Bacteroidia | Bacteroidales | Bacteroidaceae | Bacteroides | Bacteroides uniformis |
| 27775 | 7_3891 | 44 | 4 | Bacteroidetes | Bacteroidia | Bacteroidales | Prevotellaceae | Prevotella | Prevotella stercorea |
| 27792 | 42_5999 | 6 | 5 | Bacteroidetes | Bacteroidia | Bacteroidales | Bacteroidaceae | Bacteroides | Bacteroides vulgatus |
| 27809 | 130_3775 | 1963 | 69 | Bacteroidetes | Bacteroidia | Bacteroidales | Bacteroidaceae | Bacteroides | Bacteroides vulgatus |
| 27810 | 65_6727 | 55 | 18 | Bacteroidetes | Bacteroidia | Bacteroidales | Porphyromonadaceae | Parabacteroides | Parabacteroides distasonis |
| 27813 | 130_5275 | 7 | 6 | Bacteroidetes | Bacteroidia | Bacteroidales | Bacteroidaceae | Bacteroides | Bacteroides uniformis |
| 27816 | 65_5937 | 11 | 5 | Bacteroidetes | Bacteroidia | Bacteroidales | Bacteroidaceae | Bacteroides | Bacteroides uniformis |
| 27827 | 12_4541 | 8 | 5 | Bacteroidetes | Bacteroidia | Bacteroidales | Bacteroidaceae | Bacteroides | Bacteroides vulgatus |
| 27843 | 86_457 | 7 | 6 | Bacteroidetes | Bacteroidia | Bacteroidales | Bacteroidaceae | Bacteroides | Bacteroides eggerthii |
| 27862 | 123_1984 | 7 | 6 | Bacteroidetes | Bacteroidia | Bacteroidales | Bacteroidaceae | Bacteroides | Bacteroides sp. |
| 27874 | 12_2561 | 20 | 8 | Bacteroidetes | Bacteroidia | Bacteroidales | Bacteroidaceae | Bacteroides | Bacteroides vulgatus |
| 27894 | 108_3128 | 19 | 5 | Bacteroidetes | Bacteroidia | Bacteroidales | Bacteroidaceae | Bacteroides | Bacteroides thetaiotaomicron |
| 27905 | 97_2037 | 96 | 9 | Bacteroidetes | Bacteroidia | Bacteroidales | Rikenellaceae | Alistipes | Alistipes onderdonkii |
| 27908 | 73_6 | 8 | 8 | Bacteroidetes | Bacteroidia | Bacteroidales | Rikenellaceae | Alistipes | Alistipes onderdonkii |
| 27910 | 123_5770 | 19 | 8 | Bacteroidetes | Bacteroidia | Bacteroidales | Rikenellaceae | Alistipes | Alistipes putredinis |
| 27988 | 9_5760 | 17 | 7 | Bacteroidetes | Bacteroidia | Bacteroidales | Bacteroidaceae | Bacteroides | Bacteroides uniformis |
| 28058 | 69_4386 | 22 | 6 | Bacteroidetes | Bacteroidia | Bacteroidales | Prevotellaceae | Prevotella | Prevotella ruminicola |
| 28064 | 49_480 | 16 | 8 | Bacteroidetes | Bacteroidia | Bacteroidales | Bacteroidaceae | Bacteroides | Bacteroides uniformis |
| 28072 | 54_6910 | 31 | 8 | Bacteroidetes | Bacteroidia | Bacteroidales | Prevotellaceae | Prevotella | Prevotella ruminicola |
| 28083 | 81_974 | 12 | 7 | Bacteroidetes | Bacteroidia | Bacteroidales | Prevotellaceae | Prevotella | Prevotella copri |
| 28087 | 81_139 | 30 | 2 | Bacteroidetes | Bacteroidia | Bacteroidales | Prevotellaceae | Prevotella | Prevotella copri |
| 28088 | 104_712 | 193 | 15 | Bacteroidetes | Bacteroidia | Bacteroidales | Prevotellaceae | Prevotella | Prevotella copri |
| 28089 | 112_5518 | 27 | 5 | Bacteroidetes | Bacteroidia | Bacteroidales | Prevotellaceae | Prevotella | Prevotella copri |
| 28090 | 112_3458 | 31 | 10 | Bacteroidetes | Bacteroidia | Bacteroidales | Prevotellaceae | Prevotella | Prevotella copri |
| 28100 | 1_2055 | 63 | 9 | Bacteroidetes | Bacteroidia | Bacteroidales | Prevotellaceae | Prevotella | Prevotella copri |
| 28101 | 112_2997 | 80 | 9 | Bacteroidetes | Bacteroidia | Bacteroidales | Prevotellaceae | Prevotella | Prevotella copri |
| 28112 | 115_944 | 8 | 6 | Bacteroidetes | Bacteroidia | Bacteroidales | Bacteroidaceae | Bacteroides | Bacteroides uniformis |
| 28141 | 97_3835 | 9 | 5 | Bacteroidetes | Bacteroidia | Bacteroidales | Prevotellaceae | Prevotella | Prevotella stercorea |
| 28146 | 127_3065 | 25 | 8 | Bacteroidetes | Bacteroidia | Bacteroidales | Prevotellaceae | Prevotella | Prevotella stercorea |
| 28217 | 81_2809 | 29 | 3 | Bacteroidetes | Bacteroidia | Bacteroidales | Prevotellaceae | Prevotella | Prevotella copri |
| 28226 | 43_1950 | 11 | 5 | Bacteroidetes | Bacteroidia | Bacteroidales | Prevotellaceae | Prevotella | Prevotella copri |
| 28262 | 121_1823 | 131 | 7 | Bacteroidetes | Bacteroidia | Bacteroidales | Prevotellaceae | Prevotella | Prevotella copri |
| 28287 | 125_2477 | 11 | 7 | Bacteroidetes | Bacteroidia | Bacteroidales | Porphyromonadaceae | Parabacteroides | Parabacteroides merdae |
| 28293 | 8_1247 | 33 | 11 | Bacteroidetes | Bacteroidia | Bacteroidales | Bacteroidaceae | Bacteroides | Bacteroides uniformis |
| 28296 | 25_2824 | 10 | 8 | Bacteroidetes | Bacteroidia | Bacteroidales | Prevotellaceae | Prevotella | Prevotella copri |
| 28300 | 104_852 | 13 | 8 | Bacteroidetes | Bacteroidia | Bacteroidales | Prevotellaceae | Prevotella | Prevotella copri |
| 28332 | 1_1716 | 164 | 17 | Bacteroidetes | Bacteroidia | Bacteroidales | Prevotellaceae | Prevotella | Prevotella copri |
| 28342 | 1_3412 | 9 | 5 | Bacteroidetes | Bacteroidia | Bacteroidales | Prevotellaceae | Prevotella | Prevotella copri |
| 28344 | 121_994 | 21 | 7 | Bacteroidetes | Bacteroidia | Bacteroidales | Prevotellaceae | Prevotella | Prevotella copri |
| 28355 | 112_5720 | 17 | 5 | Bacteroidetes | Bacteroidia | Bacteroidales | Prevotellaceae | Prevotella | Prevotella copri |
| 28361 | 17_1581 | 14 | 9 | Bacteroidetes | Bacteroidia | Bacteroidales | Bacteroidaceae | Bacteroides | Bacteroides sp. |
| 28368 | 112_4075 | 24 | 11 | Bacteroidetes | Bacteroidia | Bacteroidales | Prevotellaceae | Prevotella | Prevotella copri |
| 28370 | 121_3269 | 30 | 9 | Bacteroidetes | Bacteroidia | Bacteroidales | Prevotellaceae | Prevotella | Prevotella copri |
| 28371 | 112_199 | 6 | 5 | Bacteroidetes | Bacteroidia | Bacteroidales | Prevotellaceae | Prevotella | Prevotella copri |
| 28372 | 23_2017 | 12 | 6 | Bacteroidetes | Bacteroidia | Bacteroidales | Prevotellaceae | Prevotella | Prevotella copri |
| 28380 | 23_1241 | 37 | 9 | Bacteroidetes | Bacteroidia | Bacteroidales | Prevotellaceae | Prevotella | Prevotella copri |
| 28385 | 115_4134 | 22 | 9 | Bacteroidetes | Bacteroidia | Bacteroidales | Prevotellaceae | Prevotella | Prevotella copri |
| 28386 | 121_1547 | 5 | 5 | Bacteroidetes | Bacteroidia | Bacteroidales | Prevotellaceae | Prevotella | Prevotella copri |
| 28390 | 33_3417 | 6 | 5 | Bacteroidetes | Bacteroidia | Bacteroidales | Prevotellaceae | Prevotella | Prevotella copri |
| 28396 | 121_3766 | 7 | 5 | Bacteroidetes | Bacteroidia | Bacteroidales | Prevotellaceae | Prevotella | Prevotella copri |
| 28399 | 1_2962 | 13 | 9 | Bacteroidetes | Bacteroidia | Bacteroidales | Prevotellaceae | Prevotella | Prevotella copri |
| 28400 | 115_1568 | 20 | 9 | Bacteroidetes | Bacteroidia | Bacteroidales | Bacteroidaceae | Bacteroides | Bacteroides uniformis |
| 28402 | 115_1206 | 12 | 7 | Bacteroidetes | Bacteroidia | Bacteroidales | Prevotellaceae | Prevotella | Prevotella copri |
| 28404 | 4_437 | 84 | 14 | Bacteroidetes | Bacteroidia | Bacteroidales | Prevotellaceae | Prevotella | Prevotella copri |
| 28419 | 115_3606 | 7 | 5 | Bacteroidetes | Bacteroidia | Bacteroidales | Bacteroidaceae | Bacteroides | Bacteroides sp. |
| 28421 | 115_90 | 26 | 10 | Bacteroidetes | Bacteroidia | Bacteroidales | Bacteroidaceae | Bacteroides | Bacteroides uniformis |
| 28422 | 81_1269 | 11 | 5 | Bacteroidetes | Bacteroidia | Bacteroidales | Prevotellaceae | Prevotella | Prevotella copri |
| 28431 | 19_3533 | 25 | 12 | Bacteroidetes | Bacteroidia | Bacteroidales | Bacteroidaceae | Bacteroides | Bacteroides uniformis |
| 28447 | 104_1079 | 18 | 9 | Bacteroidetes | Bacteroidia | Bacteroidales | Prevotellaceae | Prevotella | Prevotella copri |
| 28474 | 81_458 | 8 | 5 | Bacteroidetes | Bacteroidia | Bacteroidales | Prevotellaceae | Prevotella | Prevotella stercorea |
| 28487 | 104_1218 | 12 | 7 | Bacteroidetes | Bacteroidia | Bacteroidales | Porphyromonadaceae | Parabacteroides | Parabacteroides merdae |
| 28511 | 81_1742 | 6 | 5 | Bacteroidetes | Bacteroidia | Bacteroidales | Prevotellaceae | Prevotella | Prevotella stercorea |
| 28514 | 21_2887 | 8 | 5 | Bacteroidetes | Bacteroidia | Bacteroidales | Prevotellaceae | Prevotella | Prevotella copri |
| 28599 | 21_4482 | 24 | 6 | Bacteroidetes | Bacteroidia | Bacteroidales | Prevotellaceae | Prevotella | Prevotella copri |
| 28624 | 125_6270 | 16 | 12 | Bacteroidetes | Bacteroidia | Bacteroidales | Bacteroidaceae | Bacteroides | Bacteroides uniformis |
| 28629 | 1_410 | 36 | 4 | Bacteroidetes | Bacteroidia | Bacteroidales | Prevotellaceae | Prevotella | Prevotella copri |
| 28640 | 104_30 | 50 | 9 | Bacteroidetes | Bacteroidia | Bacteroidales | Prevotellaceae | Prevotella | Prevotella copri |
| 28653 | 121_1149 | 20 | 4 | Bacteroidetes | Bacteroidia | Bacteroidales | Prevotellaceae | Prevotella | Prevotella copri |
| 28669 | 112_926 | 18 | 5 | Bacteroidetes | Bacteroidia | Bacteroidales | Prevotellaceae | Prevotella | Prevotella copri |
| 28689 | 128_3019 | 31 | 5 | Bacteroidetes | Bacteroidia | Bacteroidales | Prevotellaceae | Prevotella | Prevotella copri |
| 28691 | 104_97 | 27 | 8 | Bacteroidetes | Bacteroidia | Bacteroidales | Prevotellaceae | Prevotella | Prevotella copri |
| 28783 | 122_169 | 17 | 6 | Bacteroidetes | Bacteroidia | Bacteroidales | Bacteroidaceae | Bacteroides | Bacteroides dorei |
| 28836 | 9_4376 | 27 | 12 | Bacteroidetes | Bacteroidia | Bacteroidales | Bacteroidaceae | Bacteroides | Bacteroides vulgatus |
| 28839 | 62_4879 | 7 | 5 | Bacteroidetes | Bacteroidia | Bacteroidales | Prevotellaceae | Prevotella | Prevotella stercorea |
| 28859 | 107_4565 | 14 | 8 | Bacteroidetes | Bacteroidia | Bacteroidales | Bacteroidaceae | Bacteroides | Bacteroides sp. |
| 28885 | 121_4926 | 38 | 8 | Bacteroidetes | Bacteroidia | Bacteroidales | Prevotellaceae | Prevotella | Prevotella copri |
| 28901 | 71_8535 | 5540 | 34 | Verrucomicrobia | Verrucomicrobiae | Verrucomicrobiales | Akkermansiaceae | Akkermansia | Akkermansia muciniphila |
| 28905 | 71_244 | 2325 | 30 | Verrucomicrobia | Verrucomicrobiae | Verrucomicrobiales | Akkermansiaceae | Akkermansia | Akkermansia muciniphila |
| 28908 | 124_7417 | 129 | 37 | Bacteroidetes | Bacteroidia | Bacteroidales | Rikenellaceae | Alistipes | Alistipes shahii |
| 28922 | 17_1678 | 6 | 6 | Bacteroidetes | Bacteroidia | Bacteroidales | Prevotellaceae | Prevotella | Prevotella copri |
| 28927 | 75_1505 | 24 | 8 | Bacteroidetes | Bacteroidia | Bacteroidales | Bacteroidaceae | Bacteroides | Bacteroides eggerthii |
| 28957 | 98_5404 | 28 | 2 | Bacteroidetes | Bacteroidia | Bacteroidales | Porphyromonadaceae | Barnesiella | Barnesiella sp. |
| 28959 | 122_2133 | 452 | 12 | Bacteroidetes | Bacteroidia | Bacteroidales | Bacteroidaceae | Bacteroides | Bacteroides intestinalis |
| 28998 | 17_3430 | 6 | 5 | Bacteroidetes | Bacteroidia | Bacteroidales | Rikenellaceae | Alistipes | Alistipes shahii |
| 29047 | 76_3952 | 6 | 5 | Bacteroidetes | Bacteroidia | Bacteroidales | Bacteroidaceae | Bacteroides | Bacteroides sp. |
| 29071 | 57_809 | 8 | 6 | Bacteroidetes | Bacteroidia | Bacteroidales | Prevotellaceae | Prevotella | Prevotella copri |
| 29075 | 12_1370 | 323 | 33 | Bacteroidetes | Bacteroidia | Bacteroidales | Porphyromonadaceae | Parabacteroides | Parabacteroides distasonis |
| 29079 | 104_757 | 7 | 6 | Bacteroidetes | Bacteroidia | Bacteroidales | Porphyromonadaceae | Parabacteroides | Parabacteroides merdae |
| 29141 | 1_2595 | 14 | 9 | Bacteroidetes | Bacteroidia | Bacteroidales | Porphyromonadaceae | Parabacteroides | Parabacteroides merdae |
| 29162 | 104_602 | 13 | 8 | Bacteroidetes | Bacteroidia | Bacteroidales | Prevotellaceae | Prevotella | Prevotella copri |
| 29176 | 82_2238 | 171 | 20 | Bacteroidetes | Bacteroidia | Bacteroidales | Bacteroidaceae | Bacteroides | Bacteroides massiliensis |
| 29186 | 20_5394 | 34 | 4 | Bacteroidetes | Bacteroidia | Bacteroidales | Bacteroidaceae | Bacteroides | Bacteroides sp. |
| 29190 | 45_7743 | 20 | 7 | Bacteroidetes | Bacteroidia | Bacteroidales | Prevotellaceae | Prevotella | Prevotella copri |
| 29191 | 47_9911 | 43 | 4 | Bacteroidetes | Bacteroidia | Bacteroidales | Prevotellaceae | Prevotella | Prevotella ruminicola |
| 29192 | 82_7501 | 22 | 4 | Bacteroidetes | Bacteroidia | Bacteroidales | Bacteroidaceae | Bacteroides | Bacteroides ovatus |
| 29210 | 125_6485 | 28 | 16 | Firmicutes | Clostridia | Clostridiales | Ruminococcaceae | Ruminococcus | Ruminococcus faecis |
| 29212 | 32_6837 | 9 | 6 | Firmicutes | Clostridia | Clostridiales | Lachnospiraceae | Blautia | Blautia |
| 29219 | 63_1571 | 7 | 5 | Firmicutes | Negativicutes | Selenomonadales | Veillonellaceae | Dialister | Dialister invisus |
| 29221 | 102_5339 | 38 | 5 | Firmicutes | Clostridia | Clostridiales | Lachnospiraceae | Lachnospiraceae incertae sedis | Lachnospiraceae incertae sedis |
| 29229 | 36_1680 | 9 | 6 | Firmicutes | Negativicutes | Selenomonadales | Veillonellaceae | Dialister | Dialister invisus |
| 29238 | 65_6357 | 6 | 6 | Firmicutes | Negativicutes | Selenomonadales | Veillonellaceae | Dialister | Dialister invisus |
| 29270 | 106_7089 | 18 | 7 | Firmicutes | Negativicutes | Selenomonadales | Veillonellaceae | Dialister | Dialister invisus |
| 29271 | 117_2426 | 6 | 5 | Firmicutes | Negativicutes | Selenomonadales | Veillonellaceae | Dialister | Dialister invisus |
| 29297 | 1_1186 | 161 | 19 | Firmicutes | Erysipelotrichia | Erysipelotrichales | Erysipelotrichaceae | Catenibacterium | Catenibacterium mitsuokai |
| 29307 | 5_1500 | 81 | 20 | Firmicutes | Erysipelotrichia | Erysipelotrichales | Erysipelotrichaceae | Catenibacterium | Catenibacterium mitsuokai |
| 29310 | 115_3847 | 9 | 7 | Firmicutes | Erysipelotrichia | Erysipelotrichales | Erysipelotrichaceae | Catenibacterium | Catenibacterium mitsuokai |
| 29321 | 1_3258 | 171 | 9 | Firmicutes | Erysipelotrichia | Erysipelotrichales | Erysipelotrichaceae | Catenibacterium | Catenibacterium mitsuokai |
| 29370 | 52_13598 | 145 | 20 | Firmicutes | Negativicutes | Selenomonadales | Veillonellaceae | Veillonella | Veillonella parvula |
| 29374 | 132_438 | 43 | 10 | Firmicutes | Negativicutes | Selenomonadales | Veillonellaceae | Veillonella | Veillonella dispar |
| 29395 | 49_2794 | 15 | 6 | Firmicutes | Clostridia | Clostridiales | Lachnospiraceae | Roseburia | Eubacterium rectale |
| 29509 | 84_1914 | 7 | 6 | Firmicutes | Clostridia | Clostridiales | Lachnospiraceae | Roseburia | Eubacterium rectale |
| 29528 | 121_6819 | 10 | 5 | Firmicutes | Negativicutes | Selenomonadales | Veillonellaceae | Mitsuokella | Mitsuokella |
| 29555 | 17_3118 | 192 | 16 | Firmicutes | Clostridia | Clostridiales | Ruminococcaceae | Ruminococcus | Ruminococcus albus |
| 29567 | 23_5153 | 8 | 5 | Bacteroidetes | Bacteroidia | Bacteroidales | Prevotellaceae | Prevotella | Prevotella copri |
| 29583 | 88_5502 | 41 | 16 | Firmicutes | Erysipelotrichia | Erysipelotrichales | Erysipelotrichaceae | Erysipelotrichaceae incertae sedis | Erysipelotrichaceae incertae sedis |
| 29590 | 60_6110 | 6 | 5 | Firmicutes | Clostridia | Clostridiales | unclassified Clostridiales | Flavonifractor | Flavonifractor |
| 29609 | 5_1297 | 163 | 7 | Firmicutes | Clostridia | Clostridiales | Lactobacillaceae | Lactobacillus | Lactobacillus kalixensis |
| 29671 | 128_1577 | 10 | 7 | Firmicutes | Negativicutes | Selenomonadales | Veillonellaceae | Dialister | Dialister invisus |
| 29679 | 27_2046 | 11 | 10 | Firmicutes | Clostridia | Clostridiales | Ruminococcaceae | Faecalibacterium | Faecalibacterium prausnitzii |
| 29689 | 117_1471 | 7 | 5 | Firmicutes | Negativicutes | Selenomonadales | Veillonellaceae | Dialister | Dialister invisus |
| 29696 | 53_1855 | 5 | 5 | Firmicutes | Clostridia | Clostridiales | Ruminococcaceae | Faecalibacterium | Faecalibacterium prausnitzii |
| 29750 | 130_7377 | 10 | 8 | Firmicutes | Negativicutes | Selenomonadales | Veillonellaceae | Dialister | Dialister invisus |
| 29759 | 117_4758 | 58 | 22 | Firmicutes | Clostridia | Clostridiales | Lachnospiraceae | Roseburia | Roseburia intestinalis |
| 29760 | 49_4319 | 6 | 5 | Firmicutes | Negativicutes | Selenomonadales | Veillonellaceae | Dialister | Dialister invisus |
| 29762 | 26_5031 | 735 | 41 | Firmicutes | Clostridia | Clostridiales | Lachnospiraceae | Coprococcus | Coprococcus sp. |
| 29770 | 60_6651 | 307 | 47 | Firmicutes | Clostridia | Clostridiales | Lachnospiraceae | Blautia | Blautia |
| 29778 | 59_6761 | 29 | 17 | Firmicutes | Negativicutes | Selenomonadales | Veillonellaceae | Dialister | Dialister invisus |
| 29792 | 65_2974 | 415 | 47 | Firmicutes | Clostridia | Clostridiales | Lachnospiraceae | Blautia | Blautia |
| 29796 | 65_2963 | 6 | 5 | Firmicutes | Negativicutes | Selenomonadales | Veillonellaceae | Dialister | Dialister invisus |
| 29836 | 65_3906 | 6 | 5 | Firmicutes | Negativicutes | Selenomonadales | Veillonellaceae | Dialister | Dialister invisus |
| 29865 | 131_9093 | 8 | 5 | Firmicutes | Negativicutes | Selenomonadales | Veillonellaceae | Dialister | Dialister invisus |
| 29901 | 2_2698 | 24 | 6 | Firmicutes | Clostridia | Clostridiales | Lachnospiraceae | Blautia | Blautia |
| 29932 | 68_10018 | 79 | 18 | Bacteroidetes | Bacteroidia | Bacteroidales | Bacteroidaceae | Bacteroides | Bacteroides sp. |
| 30000 | 131_3867 | 170 | 35 | Proteobacteria | Deltaproteobacteria | Desulfovibrionales | Desulfovibrionaceae | Desulfovibrio | Desulfovibrio piger |
| 30001 | 65_7333 | 25 | 14 | Proteobacteria | Deltaproteobacteria | Desulfovibrionales | Desulfovibrionaceae | Desulfovibrio | Desulfovibrio piger |
| 30023 | 78_4465 | 7 | 5 | Bacteroidetes | Bacteroidia | Bacteroidales | Bacteroidaceae | Bacteroides | Bacteroides uniformis |
| 30090 | 60_5477 | 18 | 5 | Bacteroidetes | Bacteroidia | Bacteroidales | Bacteroidaceae | Bacteroides | Bacteroides sp. |
| 30111 | 24_6997 | 31 | 8 | Bacteroidetes | Bacteroidia | Bacteroidales | Porphyromonadaceae | Butyricimonas | Butyricimonas virosa |
| 30113 | 20_8569 | 20 | 6 | Bacteroidetes | Bacteroidia | Bacteroidales | Porphyromonadaceae | Butyricimonas | Butyricimonas virosa |
| 30114 | 38_5610 | 51 | 14 | Bacteroidetes | Bacteroidia | Bacteroidales | Porphyromonadaceae | Butyricimonas | Butyricimonas virosa |
| 30115 | 96_8867 | 27 | 9 | Bacteroidetes | Bacteroidia | Bacteroidales | Porphyromonadaceae | Butyricimonas | Butyricimonas virosa |
| 30122 | 110_4178 | 266 | 8 | Bacteroidetes | Bacteroidia | Bacteroidales | Porphyromonadaceae | Barnesiella | Barnesiella sp. |
| 30124 | 98_4839 | 27 | 3 | Bacteroidetes | Bacteroidia | Bacteroidales | Porphyromonadaceae | Barnesiella | Barnesiella sp. |
| 30175 | 73_746 | 8 | 7 | Bacteroidetes | Bacteroidia | Bacteroidales | Bacteroidaceae | Bacteroides | Bacteroides massiliensis |
| 30191 | 122_1077 | 7 | 6 | Bacteroidetes | Bacteroidia | Bacteroidales | Bacteroidaceae | Bacteroides | Bacteroides massiliensis |
| 30212 | 113_4582 | 11 | 6 | Bacteroidetes | Bacteroidia | Bacteroidales | Bacteroidaceae | Bacteroides | Bacteroides uniformis |
| 30274 | 37_5082 | 26 | 6 | Bacteroidetes | Bacteroidia | Bacteroidales | Porphyromonadaceae | Parabacteroides | Parabacteroides merdae |
| 30300 | 108_3346 | 10 | 6 | Bacteroidetes | Bacteroidia | Bacteroidales | Prevotellaceae | Prevotella | Prevotella stercorea |
| 30319 | 58_8261 | 30 | 2 | Bacteroidetes | Bacteroidia | Bacteroidales | Bacteroidaceae | Bacteroides | Bacteroides plebeius |
| 30339 | 39_1174 | 10 | 9 | Bacteroidetes | Bacteroidia | Bacteroidales | Porphyromonadaceae | Odoribacter | Odoribacter splanchnicus |
| 30342 | 9_3029 | 5 | 5 | Bacteroidetes | Bacteroidia | Bacteroidales | Bacteroidaceae | Bacteroides | Bacteroides uniformis |
| 30343 | 9_5473 | 5 | 5 | Bacteroidetes | Bacteroidia | Bacteroidales | Bacteroidaceae | Bacteroides | Bacteroides sp. |
| 30357 | 26_4290 | 11 | 7 | Bacteroidetes | Bacteroidia | Bacteroidales | Rikenellaceae | Alistipes | Alistipes putredinis |
| 30449 | 97_7 | 8 | 7 | Bacteroidetes | Bacteroidia | Bacteroidales | Rikenellaceae | Alistipes | Alistipes sp. |
| 30455 | 125_2793 | 78 | 17 | Bacteroidetes | Bacteroidia | Bacteroidales | Rikenellaceae | Alistipes | Alistipes onderdonkii |
| 30457 | 63_2125 | 99 | 27 | Bacteroidetes | Bacteroidia | Bacteroidales | Rikenellaceae | Alistipes | Alistipes sp. |
| 30458 | 62_6246 | 6 | 5 | Bacteroidetes | Bacteroidia | Bacteroidales | Rikenellaceae | Alistipes | Alistipes onderdonkii |
| 30466 | 130_3453 | 5 | 5 | Bacteroidetes | Bacteroidia | Bacteroidales | Bacteroidaceae | Bacteroides | Bacteroides uniformis |
| 30479 | 49_4374 | 361 | 57 | Bacteroidetes | Bacteroidia | Bacteroidales | Rikenellaceae | Alistipes | Alistipes shahii |
| 30487 | 24_6539 | 28 | 15 | Bacteroidetes | Bacteroidia | Bacteroidales | Rikenellaceae | Alistipes | Alistipes shahii |
| 30503 | 50_2936 | 7 | 5 | Bacteroidetes | Bacteroidia | Bacteroidales | Rikenellaceae | Alistipes | Alistipes putredinis |
| 30516 | 90_3007 | 8 | 7 | Bacteroidetes | Bacteroidia | Bacteroidales | Bacteroidaceae | Bacteroides | Bacteroides uniformis |
| 30523 | 79_4936 | 5 | 5 | Bacteroidetes | Bacteroidia | Bacteroidales | Bacteroidaceae | Bacteroides | Bacteroides uniformis |
| 30527 | 131_8137 | 9 | 8 | Bacteroidetes | Bacteroidia | Bacteroidales | Bacteroidaceae | Bacteroides | Bacteroides eggerthii |
| 30654 | 60_1087 | 14 | 9 | Bacteroidetes | Bacteroidia | Bacteroidales | Porphyromonadaceae | Parabacteroides | Parabacteroides merdae |
| 30659 | 126_203 | 17 | 7 | Bacteroidetes | Bacteroidia | Bacteroidales | Bacteroidaceae | Bacteroides | Bacteroides massiliensis |
| 30662 | 49_1724 | 6 | 5 | Bacteroidetes | Bacteroidia | Bacteroidales | Bacteroidaceae | Bacteroides | Bacteroides uniformis |
| 30669 | 105_7114 | 56 | 3 | Bacteroidetes | Bacteroidia | Bacteroidales | Bacteroidaceae | Bacteroides | Bacteroides ovatus |
| 30670 | 82_10260 | 18 | 5 | Bacteroidetes | Bacteroidia | Bacteroidales | Bacteroidaceae | Bacteroides | Bacteroides massiliensis |
| 30701 | 105_1517 | 157 | 18 | Bacteroidetes | Bacteroidia | Bacteroidales | Bacteroidaceae | Bacteroides | Bacteroides ovatus |
| 30704 | 94_5549 | 43 | 4 | Bacteroidetes | Bacteroidia | Bacteroidales | Bacteroidaceae | Bacteroides | Bacteroides ovatus |
| 30724 | 60_590 | 11 | 5 | Bacteroidetes | Bacteroidia | Bacteroidales | Bacteroidaceae | Bacteroides | Bacteroides sp. |
| 30749 | 29_1749 | 17 | 5 | Bacteroidetes | Bacteroidia | Bacteroidales | Bacteroidaceae | Bacteroides | Bacteroides ovatus |
| 30780 | 60_4439 | 8 | 5 | Bacteroidetes | Bacteroidia | Bacteroidales | Bacteroidaceae | Bacteroides | Bacteroides sp. |
| 30801 | 122_4080 | 20 | 12 | Bacteroidetes | Bacteroidia | Bacteroidales | Bacteroidaceae | Bacteroides | Bacteroides massiliensis |
| 30812 | 120_2740 | 21 | 13 | Bacteroidetes | Bacteroidia | Bacteroidales | Bacteroidaceae | Bacteroides | Bacteroides uniformis |
| 30815 | 91_4655 | 214 | 17 | Bacteroidetes | Bacteroidia | Bacteroidales | Bacteroidaceae | Bacteroides | Bacteroides thetaiotaomicron |
| 30849 | 50_2583 | 12 | 5 | Bacteroidetes | Bacteroidia | Bacteroidales | Bacteroidaceae | Bacteroides | Bacteroides uniformis |
| 30851 | 102_1458 | 161 | 17 | Bacteroidetes | Bacteroidia | Bacteroidales | Bacteroidaceae | Bacteroides | Bacteroides salyersiae |
| 30872 | 101_9518 | 8 | 7 | Bacteroidetes | Bacteroidia | Bacteroidales | Bacteroidaceae | Bacteroides | Bacteroides intestinalis |
| 30875 | 107_4616 | 6 | 6 | Bacteroidetes | Bacteroidia | Bacteroidales | Prevotellaceae | Prevotella | Prevotella copri |
| 30891 | 9_6540 | 23 | 12 | Bacteroidetes | Bacteroidia | Bacteroidales | Bacteroidaceae | Bacteroides | Bacteroides massiliensis |
| 30905 | 101_4016 | 9 | 6 | Bacteroidetes | Bacteroidia | Bacteroidales | Bacteroidaceae | Bacteroides | Bacteroides ovatus |
| 30942 | 94_4453 | 26 | 3 | Bacteroidetes | Bacteroidia | Bacteroidales | Bacteroidaceae | Bacteroides | Bacteroides ovatus |
| 30963 | 105_5907 | 20 | 2 | Bacteroidetes | Bacteroidia | Bacteroidales | Bacteroidaceae | Bacteroides | Bacteroides caccae |
| 31023 | 42_6972 | 44 | 7 | Bacteroidetes | Bacteroidia | Bacteroidales | Bacteroidaceae | Bacteroides | Bacteroides uniformis |
| 31037 | 130_2236 | 24 | 12 | Bacteroidetes | Bacteroidia | Bacteroidales | Bacteroidaceae | Bacteroides | Bacteroides vulgatus |
| 31038 | 130_6356 | 15 | 9 | Bacteroidetes | Bacteroidia | Bacteroidales | Porphyromonadaceae | Parabacteroides | Parabacteroides merdae |
| 31052 | 71_4139 | 556 | 32 | Bacteroidetes | Bacteroidia | Bacteroidales | Bacteroidaceae | Bacteroides | Bacteroides massiliensis |
| 31066 | 105_6996 | 78 | 17 | Bacteroidetes | Bacteroidia | Bacteroidales | Bacteroidaceae | Bacteroides | Bacteroides massiliensis |
| 31074 | 60_3883 | 16 | 9 | Bacteroidetes | Bacteroidia | Bacteroidales | Porphyromonadaceae | Parabacteroides | Parabacteroides merdae |
| 31086 | 101_689 | 101 | 11 | Bacteroidetes | Bacteroidia | Bacteroidales | Bacteroidaceae | Bacteroides | Bacteroides massiliensis |
| 31111 | 107_3756 | 14 | 5 | Bacteroidetes | Bacteroidia | Bacteroidales | Bacteroidaceae | Bacteroides | Bacteroides massiliensis |
| 31119 | 107_4447 | 105 | 19 | Bacteroidetes | Bacteroidia | Bacteroidales | Bacteroidaceae | Bacteroides | Bacteroides massiliensis |
| 31121 | 90_2144 | 20 | 5 | Bacteroidetes | Bacteroidia | Bacteroidales | Bacteroidaceae | Bacteroides | Bacteroides uniformis |
| 31122 | 96_3542 | 53 | 5 | Bacteroidetes | Bacteroidia | Bacteroidales | Bacteroidaceae | Bacteroides | Bacteroides massiliensis |
| 31123 | 96_9472 | 62 | 8 | Bacteroidetes | Bacteroidia | Bacteroidales | Bacteroidaceae | Bacteroides | Bacteroides massiliensis |
| 31141 | 107_2171 | 41 | 10 | Bacteroidetes | Bacteroidia | Bacteroidales | Bacteroidaceae | Bacteroides | Bacteroides massiliensis |
| 31149 | 117_4707 | 12 | 5 | Bacteroidetes | Bacteroidia | Bacteroidales | Prevotellaceae | Prevotella | Prevotella stercorea |
| 31162 | 16_4136 | 55 | 14 | Bacteroidetes | Bacteroidia | Bacteroidales | Bacteroidaceae | Bacteroides | Bacteroides massiliensis |
| 31169 | 62_2308 | 5 | 5 | Bacteroidetes | Bacteroidia | Bacteroidales | Porphyromonadaceae | Parabacteroides | Parabacteroides merdae |
| 31178 | 124_6775 | 6 | 5 | Bacteroidetes | Bacteroidia | Bacteroidales | Bacteroidaceae | Bacteroides | Bacteroides massiliensis |
| 31192 | 26_4957 | 11 | 7 | Bacteroidetes | Bacteroidia | Bacteroidales | Bacteroidaceae | Bacteroides | Bacteroides massiliensis |
| 31203 | 105_5013 | 65 | 23 | Bacteroidetes | Bacteroidia | Bacteroidales | Bacteroidaceae | Bacteroides | Bacteroides massiliensis |
| 31207 | 4_1720 | 8 | 6 | Bacteroidetes | Bacteroidia | Bacteroidales | Bacteroidaceae | Bacteroides | Bacteroides sp. |
| 31217 | 39_5265 | 30 | 4 | Bacteroidetes | Bacteroidia | Bacteroidales | Bacteroidaceae | Bacteroides | Bacteroides sp. |
| 31239 | 9_3106 | 10 | 6 | Bacteroidetes | Bacteroidia | Bacteroidales | Porphyromonadaceae | Parabacteroides | Parabacteroides merdae |
| 31252 | 110_139 | 6 | 5 | Bacteroidetes | Bacteroidia | Bacteroidales | Bacteroidaceae | Bacteroides | Bacteroides eggerthii |
| 31267 | 63_574 | 10 | 7 | Bacteroidetes | Bacteroidia | Bacteroidales | Bacteroidaceae | Bacteroides | Bacteroides finegoldii |
| 31289 | 120_650 | 5 | 5 | Bacteroidetes | Bacteroidia | Bacteroidales | Bacteroidaceae | Bacteroides | Bacteroides uniformis |
| 31291 | 120_1783 | 9 | 6 | Bacteroidetes | Bacteroidia | Bacteroidales | Bacteroidaceae | Bacteroides | Bacteroides uniformis |
| 31312 | 20_3032 | 29 | 3 | Bacteroidetes | Bacteroidia | Bacteroidales | Bacteroidaceae | Bacteroides | Bacteroides eggerthii |
| 31324 | 111_103 | 6 | 5 | Bacteroidetes | Bacteroidia | Bacteroidales | Bacteroidaceae | Bacteroides | Bacteroides uniformis |
| 31372 | 7_1044 | 5 | 5 | Bacteroidetes | Bacteroidia | Bacteroidales | Bacteroidaceae | Bacteroides | Bacteroides finegoldii |
| 31430 | 80_5281 | 38 | 17 | Bacteroidetes | Bacteroidia | Bacteroidales | Bacteroidaceae | Bacteroides | Bacteroides uniformis |
| 31510 | 61_5878 | 7 | 5 | Bacteroidetes | Bacteroidia | Bacteroidales | Rikenellaceae | Alistipes | Alistipes shahii |
| 31512 | 12_4999 | 17 | 10 | Bacteroidetes | Bacteroidia | Bacteroidales | Porphyromonadaceae | Parabacteroides | Parabacteroides distasonis |
| 31517 | 122_3359 | 9 | 6 | Bacteroidetes | Bacteroidia | Bacteroidales | Bacteroidaceae | Bacteroides | Bacteroides sp. |
| 31521 | 60_5417 | 15 | 8 | Bacteroidetes | Bacteroidia | Bacteroidales | Bacteroidaceae | Bacteroides | Bacteroides sp. |
| 31530 | 101_4525 | 5 | 5 | Bacteroidetes | Bacteroidia | Bacteroidales | Bacteroidaceae | Bacteroides | Bacteroides uniformis |
| 31533 | 19_2381 | 20 | 10 | Bacteroidetes | Bacteroidia | Bacteroidales | Porphyromonadaceae | Parabacteroides | Parabacteroides distasonis |
| 31549 | 44_2826 | 113 | 16 | Bacteroidetes | Bacteroidia | Bacteroidales | Bacteroidaceae | Bacteroides | Bacteroides intestinalis |
| 31552 | 94_10803 | 20 | 5 | Bacteroidetes | Bacteroidia | Bacteroidales | Bacteroidaceae | Bacteroides | Bacteroides thetaiotaomicron |
| 31568 | 119_1687 | 22 | 13 | Bacteroidetes | Bacteroidia | Bacteroidales | Porphyromonadaceae | Parabacteroides | Parabacteroides merdae |
| 31575 | 60_3025 | 14 | 10 | Bacteroidetes | Bacteroidia | Bacteroidales | Bacteroidaceae | Bacteroides | Bacteroides uniformis |
| 31577 | 101_4810 | 77 | 26 | Bacteroidetes | Bacteroidia | Bacteroidales | Bacteroidaceae | Bacteroides | Bacteroides uniformis |
| 31602 | 130_3651 | 7 | 6 | Bacteroidetes | Bacteroidia | Bacteroidales | Bacteroidaceae | Bacteroides | Bacteroides uniformis |
| 31617 | 97_2479 | 13 | 9 | Bacteroidetes | Bacteroidia | Bacteroidales | Prevotellaceae | Prevotella | Prevotella stercorea |
| 31619 | 7_2870 | 13 | 6 | Bacteroidetes | Bacteroidia | Bacteroidales | Prevotellaceae | Prevotella | Prevotella stercorea |
| 31629 | 37_2679 | 15 | 7 | Bacteroidetes | Bacteroidia | Bacteroidales | Bacteroidaceae | Bacteroides | Bacteroides uniformis |
| 31646 | 105_5729 | 190 | 38 | Bacteroidetes | Bacteroidia | Bacteroidales | Bacteroidaceae | Bacteroides | Bacteroides massiliensis |
| 31657 | 130_203 | 7 | 5 | Bacteroidetes | Bacteroidia | Bacteroidales | Bacteroidaceae | Bacteroides | Bacteroides uniformis |
| 31666 | 66_4017 | 16 | 8 | Bacteroidetes | Bacteroidia | Bacteroidales | Bacteroidaceae | Bacteroides | Bacteroides uniformis |
| 31738 | 60_5656 | 7 | 6 | Bacteroidetes | Bacteroidia | Bacteroidales | Porphyromonadaceae | Parabacteroides | Parabacteroides merdae |
| 31845 | 96_9224 | 5 | 5 | Bacteroidetes | Bacteroidia | Bacteroidales | Bacteroidaceae | Bacteroides | Bacteroides uniformis |
| 31850 | 75_2918 | 9 | 7 | Bacteroidetes | Bacteroidia | Bacteroidales | Bacteroidaceae | Bacteroides | Bacteroides uniformis |
| 31897 | 119_4117 | 10 | 6 | Bacteroidetes | Bacteroidia | Bacteroidales | Porphyromonadaceae | Parabacteroides | Parabacteroides distasonis |
| 31908 | 124_7349 | 8 | 5 | Bacteroidetes | Bacteroidia | Bacteroidales | Bacteroidaceae | Bacteroides | Bacteroides massiliensis |
| 31934 | 119_4928 | 18 | 14 | Bacteroidetes | Bacteroidia | Bacteroidales | Porphyromonadaceae | Parabacteroides | Parabacteroides distasonis |
| 31936 | 10_1196 | 52 | 14 | Bacteroidetes | Bacteroidia | Bacteroidales | Porphyromonadaceae | Parabacteroides | Parabacteroides distasonis |
| 31945 | 69_4311 | 14 | 5 | Bacteroidetes | Bacteroidia | Bacteroidales | Porphyromonadaceae | Parabacteroides | Parabacteroides distasonis |
| 31950 | 63_578 | 61 | 18 | Bacteroidetes | Bacteroidia | Bacteroidales | Bacteroidaceae | Bacteroides | Bacteroides vulgatus |
| 31952 | 65_6081 | 79 | 22 | Bacteroidetes | Bacteroidia | Bacteroidales | Bacteroidaceae | Bacteroides | Bacteroides vulgatus |
| 31985 | 7_5385 | 7 | 6 | Bacteroidetes | Bacteroidia | Bacteroidales | Porphyromonadaceae | Parabacteroides | Parabacteroides distasonis |
| 31986 | 30_4929 | 6 | 6 | Bacteroidetes | Bacteroidia | Bacteroidales | Porphyromonadaceae | Parabacteroides | Parabacteroides distasonis |
| 32007 | 6_3966 | 6 | 5 | Bacteroidetes | Bacteroidia | Bacteroidales | Bacteroidaceae | Bacteroides | Bacteroides massiliensis |
| 32079 | 69_2481 | 14 | 8 | Bacteroidetes | Bacteroidia | Bacteroidales | Porphyromonadaceae | Parabacteroides | Parabacteroides distasonis |
| 32105 | 24_4181 | 13 | 7 | Bacteroidetes | Bacteroidia | Bacteroidales | Porphyromonadaceae | Parabacteroides | Parabacteroides merdae |
| 32108 | 50_563 | 14 | 8 | Bacteroidetes | Bacteroidia | Bacteroidales | Porphyromonadaceae | Parabacteroides | Parabacteroides distasonis |
| 32120 | 108_3402 | 12 | 7 | Bacteroidetes | Bacteroidia | Bacteroidales | Bacteroidaceae | Bacteroides | Bacteroides intestinalis |
| 32122 | 75_702 | 10 | 8 | Bacteroidetes | Bacteroidia | Bacteroidales | Bacteroidaceae | Bacteroides | Bacteroides eggerthii |
| 32144 | 122_3979 | 23 | 8 | Bacteroidetes | Bacteroidia | Bacteroidales | Bacteroidaceae | Bacteroides | Bacteroides dorei |
| 32159 | 41_8569 | 48 | 11 | Bacteroidetes | Bacteroidia | Bacteroidales | Bacteroidaceae | Bacteroides | Bacteroides vulgatus |
| 32241 | 12_2279 | 48 | 10 | Bacteroidetes | Bacteroidia | Bacteroidales | Porphyromonadaceae | Parabacteroides | Parabacteroides distasonis |
| 32258 | 91_9641 | 13 | 5 | Bacteroidetes | Bacteroidia | Bacteroidales | Bacteroidaceae | Bacteroides | Bacteroides sp. |
| 32276 | 129_5853 | 8 | 7 | Bacteroidetes | Bacteroidia | Bacteroidales | Bacteroidaceae | Bacteroides | Bacteroides vulgatus |
| 32298 | 107_562 | 47 | 20 | Bacteroidetes | Bacteroidia | Bacteroidales | Bacteroidaceae | Bacteroides | Bacteroides vulgatus |
| 32300 | 12_4560 | 55 | 11 | Bacteroidetes | Bacteroidia | Bacteroidales | Bacteroidaceae | Bacteroides | Bacteroides vulgatus |
| 32304 | 128_1606 | 6 | 5 | Bacteroidetes | Bacteroidia | Bacteroidales | Rikenellaceae | Alistipes | Alistipes putredinis |
| 32308 | 7_2422 | 128 | 10 | Bacteroidetes | Bacteroidia | Bacteroidales | Prevotellaceae | Prevotella | Prevotella stercorea |
| 32321 | 125_5049 | 6 | 5 | Firmicutes | Clostridia | Clostridiales | Lachnospiraceae | Roseburia | Eubacterium rectale |
| 32339 | 130_7397 | 911 | 67 | Bacteroidetes | Bacteroidia | Bacteroidales | Bacteroidaceae | Bacteroides | Bacteroides vulgatus |
| 32340 | 114_2836 | 6 | 5 | Bacteroidetes | Bacteroidia | Bacteroidales | Bacteroidaceae | Bacteroides | Bacteroides sp. |
| 32370 | 10_1917 | 8 | 5 | Bacteroidetes | Bacteroidia | Bacteroidales | Bacteroidaceae | Bacteroides | Bacteroides uniformis |
| 32397 | 75_1520 | 14 | 7 | Bacteroidetes | Bacteroidia | Bacteroidales | Rikenellaceae | Alistipes | Alistipes sp. |
| 32399 | 123_5870 | 591 | 31 | Bacteroidetes | Bacteroidia | Bacteroidales | Bacteroidaceae | Bacteroides | Bacteroides vulgatus |
| 32421 | 37_6004 | 6 | 5 | Bacteroidetes | Bacteroidia | Bacteroidales | Bacteroidaceae | Bacteroides | Bacteroides vulgatus |
| 32425 | 65_1475 | 21 | 10 | Bacteroidetes | Bacteroidia | Bacteroidales | Bacteroidaceae | Bacteroides | Bacteroides vulgatus |
| 32488 | 123_6877 | 9 | 5 | Bacteroidetes | Bacteroidia | Bacteroidales | Bacteroidaceae | Bacteroides | Bacteroides vulgatus |
| 32557 | 39_5830 | 7 | 5 | Bacteroidetes | Bacteroidia | Bacteroidales | Bacteroidaceae | Bacteroides | Bacteroides uniformis |
| 32574 | 1_1744 | 22 | 5 | Bacteroidetes | Bacteroidia | Bacteroidales | Prevotellaceae | Prevotella | Prevotella stercorea |
| 32596 | 81_1852 | 15 | 5 | Bacteroidetes | Bacteroidia | Bacteroidales | Prevotellaceae | Prevotella | Prevotella stercorea |
| 32712 | 59_4389 | 35 | 7 | Bacteroidetes | Bacteroidia | Bacteroidales | Prevotellaceae | Prevotella | Prevotella copri |
| 32715 | 1_691 | 41 | 10 | Bacteroidetes | Bacteroidia | Bacteroidales | Prevotellaceae | Prevotella | Prevotella copri |
| 32732 | 101_7965 | 57 | 12 | Bacteroidetes | Bacteroidia | Bacteroidales | Prevotellaceae | Prevotella | Prevotella stercorea |
| 32744 | 112_4246 | 12 | 5 | Bacteroidetes | Bacteroidia | Bacteroidales | Prevotellaceae | Prevotella | Prevotella copri |
| 32747 | 104_1502 | 80 | 10 | Bacteroidetes | Bacteroidia | Bacteroidales | Prevotellaceae | Prevotella | Prevotella copri |
| 32757 | 112_2885 | 28 | 3 | Bacteroidetes | Bacteroidia | Bacteroidales | Prevotellaceae | Prevotella | Prevotella copri |
| 32776 | 7_995 | 24 | 12 | Bacteroidetes | Bacteroidia | Bacteroidales | Bacteroidaceae | Bacteroides | Bacteroides uniformis |
| 32781 | 7_4362 | 7 | 5 | Bacteroidetes | Bacteroidia | Bacteroidales | Bacteroidaceae | Bacteroides | Bacteroides uniformis |
| 32792 | 49_288 | 10 | 5 | Bacteroidetes | Bacteroidia | Bacteroidales | Prevotellaceae | Prevotella | Prevotella stercorea |
| 32797 | 49_3814 | 488 | 14 | Bacteroidetes | Bacteroidia | Bacteroidales | Prevotellaceae | Prevotella | Prevotella stercorea |
| 32850 | 129_1332 | 46 | 3 | Bacteroidetes | Bacteroidia | Bacteroidales | Prevotellaceae | Prevotella | Prevotella stercorea |
| 32877 | 104_671 | 52 | 9 | Bacteroidetes | Bacteroidia | Bacteroidales | Prevotellaceae | Prevotella | Prevotella stercorea |
| 32920 | 43_361 | 11 | 6 | Bacteroidetes | Bacteroidia | Bacteroidales | Prevotellaceae | Prevotella | Prevotella copri |
| 32925 | 54_5484 | 5 | 5 | Bacteroidetes | Bacteroidia | Bacteroidales | Prevotellaceae | Prevotella | Prevotella copri |
| 32929 | 1_964 | 27 | 12 | Bacteroidetes | Bacteroidia | Bacteroidales | Porphyromonadaceae | Parabacteroides | Parabacteroides merdae |
| 32942 | 28_908 | 8 | 5 | Bacteroidetes | Bacteroidia | Bacteroidales | Prevotellaceae | Prevotella | Prevotella copri |
| 32944 | 112_4667 | 6 | 5 | Bacteroidetes | Bacteroidia | Bacteroidales | Prevotellaceae | Prevotella | Prevotella copri |
| 32965 | 123_2313 | 6 | 5 | Bacteroidetes | Bacteroidia | Bacteroidales | Prevotellaceae | Prevotella | Prevotella copri |
| 32966 | 112_2845 | 9 | 6 | Bacteroidetes | Bacteroidia | Bacteroidales | Prevotellaceae | Prevotella | Prevotella copri |
| 32991 | 123_2396 | 106 | 11 | Bacteroidetes | Bacteroidia | Bacteroidales | Prevotellaceae | Prevotella | Prevotella copri |
| 33014 | 23_5823 | 128 | 15 | Bacteroidetes | Bacteroidia | Bacteroidales | Prevotellaceae | Prevotella | Prevotella copri |
| 33028 | 4_2061 | 78 | 17 | Bacteroidetes | Bacteroidia | Bacteroidales | Prevotellaceae | Prevotella | Prevotella copri |
| 33030 | 123_4510 | 8 | 5 | Bacteroidetes | Bacteroidia | Bacteroidales | Prevotellaceae | Prevotella | Prevotella copri |
| 33069 | 104_615 | 8 | 5 | Bacteroidetes | Bacteroidia | Bacteroidales | Prevotellaceae | Prevotella | Prevotella stercorea |
| 33082 | 23_4035 | 17 | 7 | Bacteroidetes | Bacteroidia | Bacteroidales | Bacteroidaceae | Bacteroides | Bacteroides uniformis |
| 33175 | 121_3816 | 37 | 8 | Bacteroidetes | Bacteroidia | Bacteroidales | Prevotellaceae | Prevotella | Prevotella copri |
| 33176 | 66_4910 | 72 | 9 | Bacteroidetes | Bacteroidia | Bacteroidales | Prevotellaceae | Prevotella | Prevotella copri |
| 33234 | 121_1023 | 7 | 7 | Bacteroidetes | Bacteroidia | Bacteroidales | Prevotellaceae | Prevotella | Prevotella copri |
| 33273 | 107_3872 | 7 | 5 | Bacteroidetes | Bacteroidia | Bacteroidales | Prevotellaceae | Prevotella | Prevotella copri |
| 33340 | 101_7278 | 50 | 11 | Bacteroidetes | Bacteroidia | Bacteroidales | Porphyromonadaceae | Coprobacter | Coprobacter fastidiosus |
| 33356 | 9_3099 | 7 | 5 | Bacteroidetes | Bacteroidia | Bacteroidales | Bacteroidaceae | Bacteroides | Bacteroides uniformis |
| 33379 | 112_403 | 9 | 5 | Bacteroidetes | Bacteroidia | Bacteroidales | Prevotellaceae | Prevotella | Prevotella stercorea |
| 33434 | 112_2346 | 29 | 4 | Bacteroidetes | Bacteroidia | Bacteroidales | Prevotellaceae | Prevotella | Prevotella copri |
| 33437 | 1_612 | 8 | 5 | Bacteroidetes | Bacteroidia | Bacteroidales | Prevotellaceae | Prevotella | Prevotella copri |
| 33441 | 124_3065 | 58 | 8 | Firmicutes | Clostridia | Clostridiales | Peptococcaceae | Peptococcus | Peptococcus |
| 33444 | 71_3536 | 5737 | 36 | Verrucomicrobia | Verrucomicrobiae | Verrucomicrobiales | Akkermansiaceae | Akkermansia | Akkermansia muciniphila |
| 33445 | 71_1487 | 9 | 5 | Verrucomicrobia | Verrucomicrobiae | Verrucomicrobiales | Akkermansiaceae | Akkermansia | Akkermansia muciniphila |
| 33453 | 100_1350 | 42 | 3 | Verrucomicrobia | Verrucomicrobiae | Verrucomicrobiales | Akkermansiaceae | Akkermansia | Akkermansia muciniphila |
| 33454 | 100_1293 | 421 | 3 | Verrucomicrobia | Verrucomicrobiae | Verrucomicrobiales | Akkermansiaceae | Akkermansia | Akkermansia muciniphila |
| 33522 | 112_4978 | 42 | 14 | Bacteroidetes | Bacteroidia | Bacteroidales | Prevotellaceae | Prevotella | Prevotella copri |
| 33523 | 45_8003 | 16 | 7 | Bacteroidetes | Bacteroidia | Bacteroidales | Prevotellaceae | Prevotella | Prevotella copri |
| 33648 | 130_3778 | 12 | 6 | Bacteroidetes | Bacteroidia | Bacteroidales | Bacteroidaceae | Bacteroides | Bacteroides finegoldii |
| 33672 | 29_4310 | 76 | 16 | Bacteroidetes | Bacteroidia | Bacteroidales | Rikenellaceae | Alistipes | Alistipes finegoldii |
| 33681 | 24_7171 | 13 | 6 | Bacteroidetes | Bacteroidia | Bacteroidales | Porphyromonadaceae | Parabacteroides | Parabacteroides distasonis |
| 33712 | 42_6984 | 687 | 14 | Bacteroidetes | Bacteroidia | Bacteroidales | Bacteroidaceae | Bacteroides | Bacteroides vulgatus |
| 33717 | 123_6447 | 11 | 7 | Bacteroidetes | Bacteroidia | Bacteroidales | Bacteroidaceae | Bacteroides | Bacteroides vulgatus |
| 33721 | 2_4205 | 9 | 5 | Bacteroidetes | Bacteroidia | Bacteroidales | Bacteroidaceae | Bacteroides | Bacteroides intestinalis |
| 33731 | 119_6283 | 14 | 9 | Bacteroidetes | Bacteroidia | Bacteroidales | Porphyromonadaceae | Odoribacter | Odoribacter splanchnicus |
| 33758 | 113_2707 | 94 | 41 | Firmicutes | Clostridia | Clostridiales | Lachnospiraceae | Blautia | Blautia |
| 33778 | 121_6804 | 111 | 30 | Firmicutes | Clostridia | Clostridiales | Lachnospiraceae | Lachnospiraceae incertae sedis | Lachnospiraceae incertae sedis |
| 33782 | 25_2419 | 7 | 6 | Firmicutes | Negativicutes | Selenomonadales | Veillonellaceae | Dialister | Dialister invisus |
| 33784 | 89_9421 | 181 | 26 | Firmicutes | Clostridia | Clostridiales | Lachnospiraceae | Roseburia | Roseburia faecis |
| 33792 | 49_3857 | 31 | 8 | Firmicutes | Negativicutes | Selenomonadales | Veillonellaceae | Dialister | Dialister invisus |
| 33795 | 17_2502 | 10 | 7 | Firmicutes | Negativicutes | Selenomonadales | Veillonellaceae | Dialister | Dialister invisus |
| 33809 | 60_2769 | 24 | 12 | Firmicutes | Negativicutes | Selenomonadales | Veillonellaceae | Dialister | Dialister invisus |
| 33811 | 117_3551 | 5 | 5 | Firmicutes | Negativicutes | Selenomonadales | Veillonellaceae | Dialister | Dialister invisus |
| 33813 | 17_2052 | 14 | 7 | Firmicutes | Negativicutes | Selenomonadales | Veillonellaceae | Dialister | Dialister invisus |
| 33825 | 42_4499 | 12 | 7 | Bacteroidetes | Bacteroidia | Bacteroidales | Bacteroidaceae | Bacteroides | Bacteroides vulgatus |
| 33846 | 4_2006 | 42 | 8 | Firmicutes | Erysipelotrichia | Erysipelotrichales | Erysipelotrichaceae | Catenibacterium | Catenibacterium mitsuokai |
| 33858 | 66_4980 | 73 | 19 | Firmicutes | Erysipelotrichia | Erysipelotrichales | Erysipelotrichaceae | Catenibacterium | Catenibacterium mitsuokai |
| 33868 | 1_1686 | 38 | 8 | Firmicutes | Erysipelotrichia | Erysipelotrichales | Erysipelotrichaceae | Catenibacterium | Catenibacterium mitsuokai |
| 33944 | 47_2080 | 90 | 14 | Firmicutes | Negativicutes | Selenomonadales | Veillonellaceae | Veillonella | Veillonella parvula |
| 33945 | 132_1909 | 13 | 7 | Bacteroidetes | Bacteroidia | Bacteroidales | Bacteroidaceae | Bacteroides | Bacteroides vulgatus |
| 33998 | 49_1206 | 14 | 7 | Firmicutes | Clostridia | Clostridiales | Lachnospiraceae | Roseburia | Eubacterium rectale |
| 34012 | 30_7183 | 21 | 9 | Firmicutes | Negativicutes | Selenomonadales | Veillonellaceae | Megasphaera | Megasphaera elsdenii |
| 34018 | 59_6161 | 5 | 5 | Firmicutes | Negativicutes | Selenomonadales | Veillonellaceae | Dialister | Dialister invisus |
| 34026 | 128_1599 | 18 | 8 | Bacteroidetes | Bacteroidia | Bacteroidales | Bacteroidaceae | Bacteroides | Bacteroides uniformis |
| 34033 | 117_550 | 15 | 12 | Firmicutes | Clostridia | Clostridiales | Lachnospiraceae | Blautia | Blautia |
| 34043 | 106_7530 | 6 | 5 | Actinobacteria | Coriobacteriia | Coriobacteriales | Coriobacteriaceae | Collinsella | Collinsella aerofaciens |
| 34060 | 17_2621 | 9 | 6 | Bacteroidetes | Bacteroidia | Bacteroidales | Prevotellaceae | Prevotella | Prevotella copri |
| 34061 | 128_5143 | 31 | 3 | Firmicutes | Negativicutes | Selenomonadales | Veillonellaceae | Megasphaera | Megasphaera elsdenii |
| 34159 | 43_1506 | 10 | 6 | Firmicutes | Negativicutes | Selenomonadales | Veillonellaceae | Mitsuokella | Mitsuokella |
| 34169 | 25_1030 | 305 | 14 | Firmicutes | Negativicutes | Selenomonadales | Acidaminococcaceae | Acidaminococcus | Acidaminococcus fermentans |
| 34170 | 125_6224 | 43 | 9 | Firmicutes | Negativicutes | Selenomonadales | Acidaminococcaceae | Acidaminococcus | Acidaminococcus fermentans |
| 34182 | 124_3840 | 49 | 6 | Firmicutes | Clostridia | Clostridiales | Ruminococcaceae | Ruminococcus | Ruminococcus albus |
| 34205 | 76_4786 | 96 | 30 | Firmicutes | Erysipelotrichia | Erysipelotrichales | Erysipelotrichaceae | Erysipelotrichaceae incertae sedis | Erysipelotrichaceae incertae sedis |
| 34210 | 114_8116 | 49 | 21 | Firmicutes | Clostridia | Clostridiales | Oscillospiraceae | Oscillibacter | Oscillibacter |
| 34217 | 62_2880 | 53 | 15 | Firmicutes | Erysipelotrichia | Erysipelotrichales | Erysipelotrichaceae | Erysipelotrichaceae incertae sedis | Erysipelotrichaceae incertae sedis |
| 34220 | 96_845 | 54 | 8 | Firmicutes | Erysipelotrichia | Erysipelotrichales | Erysipelotrichaceae | Erysipelotrichaceae incertae sedis | Erysipelotrichaceae incertae sedis |
| 34223 | 24_6672 | 44 | 16 | Firmicutes | Erysipelotrichia | Erysipelotrichales | Erysipelotrichaceae | Erysipelotrichaceae incertae sedis | Erysipelotrichaceae incertae sedis |
| 34225 | 6_4564 | 9 | 6 | Firmicutes | Erysipelotrichia | Erysipelotrichales | Erysipelotrichaceae | Erysipelotrichaceae incertae sedis | Erysipelotrichaceae incertae sedis |
| 34232 | 57_549 | 12 | 9 | Firmicutes | Clostridia | Clostridiales | Ruminococcaceae | Papillibacter | Papillibacter cinnamivorans |
| 34233 | 71_4559 | 25 | 9 | Firmicutes | Clostridia | Clostridiales | Ruminococcaceae | Papillibacter | Papillibacter cinnamivorans |
| 34234 | 78_4627 | 6 | 6 | Firmicutes | Clostridia | Clostridiales | Ruminococcaceae | Papillibacter | Papillibacter cinnamivorans |
| 34243 | 125_6327 | 43 | 9 | Firmicutes | Bacilli | Lactobacillales | Streptococcaceae | Streptococcus | Streptococcus sp. |
| 34263 | 124_5394 | 10 | 6 | Bacteroidetes | Bacteroidia | Bacteroidales | Bacteroidaceae | Bacteroides | Bacteroides massiliensis |
| 34300 | 35_5288 | 17 | 10 | Firmicutes | Clostridia | Clostridiales | Ruminococcaceae | Faecalibacterium | Faecalibacterium prausnitzii |
| 34304 | 18_157 | 6 | 5 | Firmicutes | Negativicutes | Selenomonadales | Veillonellaceae | Dialister | Dialister invisus |
| 34307 | 43_2654 | 181 | 38 | Firmicutes | Clostridia | Clostridiales | Ruminococcaceae | Faecalibacterium | Faecalibacterium prausnitzii |
| 34310 | 128_3642 | 8 | 6 | Firmicutes | Negativicutes | Selenomonadales | Veillonellaceae | Dialister | Dialister invisus |
| 34318 | 49_2818 | 9 | 6 | Firmicutes | Negativicutes | Selenomonadales | Acidaminococcaceae | Acidaminococcus | Acidaminococcus intestini |
| 34343 | 131_7369 | 71 | 30 | Firmicutes | Clostridia | Clostridiales | Ruminococcaceae | incertae sedis | Ruminococcaceae incertae sedis |
| 34345 | 50_1549 | 15 | 9 | Proteobacteria | Betaproteobacteria | Burkholderiales | Oxalobacteraceae | Oxalobacter | Oxalobacter formigenes |
| 34405 | 97_2538 | 9 | 5 | Firmicutes | Clostridia | Clostridiales | Lachnospiraceae | Blautia | Blautia |
| 34410 | 48_4714 | 9 | 5 | Firmicutes | Negativicutes | Selenomonadales | Veillonellaceae | Dialister | Dialister invisus |
| 34414 | 48_5120 | 5 | 5 | Firmicutes | Negativicutes | Selenomonadales | Veillonellaceae | Dialister | Dialister invisus |
| 34421 | 60_3560 | 5 | 5 | Firmicutes | Clostridia | Clostridiales | Lachnospiraceae | Roseburia | Eubacterium rectale |
| 34426 | 40_3246 | 132 | 38 | Firmicutes | Clostridia | Clostridiales | Lachnospiraceae | Blautia | Blautia |
| 34433 | 60_6350 | 9 | 7 | Firmicutes | Negativicutes | Selenomonadales | Veillonellaceae | Dialister | Dialister invisus |
| 34445 | 111_10356 | 137 | 20 | Firmicutes | Clostridia | Clostridiales | Lachnospiraceae | Lachnospiraceae incertae sedis | Lachnospiraceae incertae sedis |
| 34446 | 73_1173 | 35 | 18 | Firmicutes | Clostridia | Clostridiales | Lachnospiraceae | Lachnospiraceae incertae sedis | Lachnospiraceae incertae sedis |
| 34459 | 55_3895 | 11 | 7 | Firmicutes | Clostridia | Clostridiales | Lachnospiraceae | Blautia | Blautia |
| 34463 | 9_3234 | 5 | 5 | Firmicutes | Clostridia | Clostridiales | Ruminococcaceae | Faecalibacterium | Faecalibacterium prausnitzii |
| 34469 | 84_3539 | 108 | 28 | Firmicutes | Clostridia | Clostridiales | Eubacteriaceae | Eubacterium | Eubacterium sp. |
| 34477 | 37_3247 | 5 | 5 | Firmicutes | Negativicutes | Selenomonadales | Veillonellaceae | Dialister | Dialister invisus |
| 34479 | 96_9127 | 13 | 10 | Firmicutes | Clostridia | Clostridiales | Eubacteriaceae | Eubacterium | Eubacterium sp. |
| 34550 | 61_5694 | 7 | 5 | NULL | NULL | NULL | NULL | NULL | NULL |
| 34589 | 62_5013 | 16 | 8 | Firmicutes | Clostridia | Clostridiales | Eubacteriaceae | Eubacterium | Eubacterium desmolans |
| 34651 | 124_7276 | 205 | 36 | Firmicutes | Erysipelotrichia | Erysipelotrichales | Erysipelotrichaceae | Erysipelotrichaceae incertae sedis | Erysipelotrichaceae incertae sedis |
| 34672 | 82_10772 | 14 | 6 | Bacteroidetes | Bacteroidia | Bacteroidales | Bacteroidaceae | Bacteroides | Bacteroides massiliensis |
| 34693 | 79_3165 | 13 | 6 | Bacteroidetes | Bacteroidia | Bacteroidales | Bacteroidaceae | Bacteroides | Bacteroides sp. |
| 34697 | 55_2048 | 51 | 10 | Proteobacteria | Gammaproteobacteria | Enterobacteriales | Enterobacteriaceae | Escherichia/Shigella | Escherichia/Shigella |
| 34720 | 55_2918 | 645 | 12 | Proteobacteria | Gammaproteobacteria | Enterobacteriales | Enterobacteriaceae | Escherichia/Shigella | Escherichia/Shigella |
| 34731 | 96_9797 | 11 | 9 | Proteobacteria | Deltaproteobacteria | Desulfovibrionales | Desulfovibrionaceae | Desulfovibrio | Desulfovibrio piger |
| 34761 | 22_7907 | 13 | 5 | Bacteroidetes | Bacteroidia | Bacteroidales | Bacteroidaceae | Bacteroides | Bacteroides intestinalis |
| 34763 | 122_907 | 6 | 5 | Bacteroidetes | Bacteroidia | Bacteroidales | Bacteroidaceae | Bacteroides | Bacteroides dorei |
| 34781 | 20_8666 | 10 | 7 | Bacteroidetes | Bacteroidia | Bacteroidales | Porphyromonadaceae | Odoribacter | Odoribacter splanchnicus |
| 34791 | 60_3158 | 18 | 6 | Bacteroidetes | Bacteroidia | Bacteroidales | Porphyromonadaceae | Parabacteroides | Parabacteroides merdae |
| 34812 | 47_5779 | 10 | 7 | Bacteroidetes | Bacteroidia | Bacteroidales | Porphyromonadaceae | Butyricimonas | Butyricimonas virosa |
| 34815 | 20_3887 | 14 | 6 | Bacteroidetes | Bacteroidia | Bacteroidales | Porphyromonadaceae | Butyricimonas | Butyricimonas virosa |
| 34816 | 2_5418 | 13 | 8 | Bacteroidetes | Bacteroidia | Bacteroidales | Porphyromonadaceae | Butyricimonas | Butyricimonas virosa |
| 34820 | 2_6220 | 12 | 5 | Bacteroidetes | Bacteroidia | Bacteroidales | Porphyromonadaceae | Butyricimonas | Butyricimonas virosa |
| 34877 | 101_5826 | 8 | 7 | Bacteroidetes | Bacteroidia | Bacteroidales | Bacteroidaceae | Bacteroides | Bacteroides massiliensis |
| 34895 | 4_3984 | 24 | 15 | Bacteroidetes | Bacteroidia | Bacteroidales | Bacteroidaceae | Bacteroides | Bacteroides vulgatus |
| 34896 | 120_1785 | 9 | 8 | Bacteroidetes | Bacteroidia | Bacteroidales | Bacteroidaceae | Bacteroides | Bacteroides vulgatus |
| 34897 | 44_1896 | 10 | 9 | Bacteroidetes | Bacteroidia | Bacteroidales | Porphyromonadaceae | Parabacteroides | Parabacteroides distasonis |
| 34903 | 44_3109 | 44 | 10 | Bacteroidetes | Bacteroidia | Bacteroidales | Bacteroidaceae | Bacteroides | Bacteroides dorei |
| 34913 | 69_3709 | 24 | 11 | Bacteroidetes | Bacteroidia | Bacteroidales | Porphyromonadaceae | Parabacteroides | Parabacteroides merdae |
| 34914 | 19_6094 | 11 | 6 | Bacteroidetes | Bacteroidia | Bacteroidales | Porphyromonadaceae | Parabacteroides | Parabacteroides merdae |
| 34920 | 101_4952 | 7 | 7 | Bacteroidetes | Bacteroidia | Bacteroidales | Porphyromonadaceae | Parabacteroides | Parabacteroides merdae |
| 34932 | 1_2259 | 37 | 9 | Bacteroidetes | Bacteroidia | Bacteroidales | Bacteroidaceae | Bacteroides | Bacteroides dorei |
| 34936 | 1_1103 | 181 | 43 | Bacteroidetes | Bacteroidia | Bacteroidales | Bacteroidaceae | Bacteroides | Bacteroides vulgatus |
| 34941 | 1_2787 | 9 | 5 | Bacteroidetes | Bacteroidia | Bacteroidales | Prevotellaceae | Prevotella | Prevotella stercorea |
| 34982 | 58_353 | 39 | 2 | Bacteroidetes | Bacteroidia | Bacteroidales | Bacteroidaceae | Bacteroides | Bacteroides plebeius |
| 35011 | 44_2486 | 42 | 14 | Bacteroidetes | Bacteroidia | Bacteroidales | Porphyromonadaceae | Odoribacter | Odoribacter splanchnicus |
| 35029 | 101_6865 | 9 | 5 | Bacteroidetes | Bacteroidia | Bacteroidales | Bacteroidaceae | Bacteroides | Bacteroides uniformis |
| 35036 | 32_3084 | 42 | 12 | Bacteroidetes | Bacteroidia | Bacteroidales | Bacteroidaceae | Bacteroides | Bacteroides intestinalis |
| 35037 | 79_1360 | 7 | 6 | Bacteroidetes | Bacteroidia | Bacteroidales | Bacteroidaceae | Bacteroides | Bacteroides ovatus |
| 35040 | 113_1354 | 11 | 6 | Bacteroidetes | Bacteroidia | Bacteroidales | Bacteroidaceae | Bacteroides | Bacteroides intestinalis |
| 35059 | 70_3360 | 62 | 6 | Bacteroidetes | Bacteroidia | Bacteroidales | Bacteroidaceae | Bacteroides | Bacteroides intestinalis |
| 35094 | 124_3620 | 155 | 23 | Bacteroidetes | Bacteroidia | Bacteroidales | Rikenellaceae | Alistipes | Alistipes finegoldii |
| 35095 | 16_6508 | 7 | 5 | Bacteroidetes | Bacteroidia | Bacteroidales | Rikenellaceae | Alistipes | Alistipes finegoldii |
| 35108 | 10_3282 | 10 | 5 | Bacteroidetes | Bacteroidia | Bacteroidales | Rikenellaceae | Alistipes | Alistipes sp. |
| 35113 | 130_3512 | 83 | 34 | Bacteroidetes | Bacteroidia | Bacteroidales | Rikenellaceae | Alistipes | Alistipes sp. |
| 35155 | 129_1547 | 29 | 9 | Bacteroidetes | Bacteroidia | Bacteroidales | Rikenellaceae | Alistipes | Alistipes onderdonkii |
| 35170 | 55_1260 | 9 | 6 | Bacteroidetes | Bacteroidia | Bacteroidales | Bacteroidaceae | Bacteroides | Bacteroides uniformis |
| 35227 | 68_7329 | 79 | 5 | Bacteroidetes | Bacteroidia | Bacteroidales | Bacteroidaceae | Bacteroides | Bacteroides ovatus |
| 35233 | 79_5199 | 41 | 5 | Bacteroidetes | Bacteroidia | Bacteroidales | Bacteroidaceae | Bacteroides | Bacteroides sp. |
| 35253 | 62_3560 | 13 | 10 | Bacteroidetes | Bacteroidia | Bacteroidales | Bacteroidaceae | Bacteroides | Bacteroides xylanisolvens |
| 35266 | 126_640 | 69 | 19 | Bacteroidetes | Bacteroidia | Bacteroidales | Bacteroidaceae | Bacteroides | Bacteroides massiliensis |
| 35277 | 58_4244 | 122 | 4 | Bacteroidetes | Bacteroidia | Bacteroidales | Bacteroidaceae | Bacteroides | Bacteroides plebeius |
| 35279 | 101_3587 | 21 | 11 | Bacteroidetes | Bacteroidia | Bacteroidales | Porphyromonadaceae | Parabacteroides | Parabacteroides merdae |
| 35369 | 105_5984 | 30 | 11 | Bacteroidetes | Bacteroidia | Bacteroidales | Bacteroidaceae | Bacteroides | Bacteroides massiliensis |
| 35385 | 81_4697 | 70 | 10 | Bacteroidetes | Bacteroidia | Bacteroidales | Bacteroidaceae | Bacteroides | Bacteroides thetaiotaomicron |
| 35386 | 101_2649 | 8 | 5 | Bacteroidetes | Bacteroidia | Bacteroidales | Porphyromonadaceae | Parabacteroides | Parabacteroides merdae |
| 35389 | 101_8341 | 19 | 15 | Bacteroidetes | Bacteroidia | Bacteroidales | Porphyromonadaceae | Parabacteroides | Parabacteroides merdae |
| 35403 | 102_604 | 5 | 5 | Bacteroidetes | Bacteroidia | Bacteroidales | Bacteroidaceae | Bacteroides | Bacteroides thetaiotaomicron |
| 35411 | 105_6284 | 19 | 5 | Bacteroidetes | Bacteroidia | Bacteroidales | Bacteroidaceae | Bacteroides | Bacteroides ovatus |
| 35415 | 107_540 | 44 | 18 | Bacteroidetes | Bacteroidia | Bacteroidales | Bacteroidaceae | Bacteroides | Bacteroides salyersiae |
| 35417 | 107_1405 | 13 | 7 | Bacteroidetes | Bacteroidia | Bacteroidales | Prevotellaceae | Prevotella | Prevotella copri |
| 35448 | 29_1791 | 5 | 5 | Bacteroidetes | Bacteroidia | Bacteroidales | Bacteroidaceae | Bacteroides | Bacteroides intestinalis |
| 35454 | 2_6810 | 11 | 5 | Bacteroidetes | Bacteroidia | Bacteroidales | Bacteroidaceae | Bacteroides | Bacteroides intestinalis |
| 35474 | 9_1893 | 76 | 12 | Bacteroidetes | Bacteroidia | Bacteroidales | Bacteroidaceae | Bacteroides | Bacteroides massiliensis |
| 35487 | 105_704 | 23 | 3 | Bacteroidetes | Bacteroidia | Bacteroidales | Bacteroidaceae | Bacteroides | Bacteroides massiliensis |
| 35498 | 124_4346 | 11 | 8 | Bacteroidetes | Bacteroidia | Bacteroidales | Bacteroidaceae | Bacteroides | Bacteroides caccae |
| 35503 | 82_9280 | 8 | 8 | Bacteroidetes | Bacteroidia | Bacteroidales | Bacteroidaceae | Bacteroides | Bacteroides caccae |
| 35507 | 75_2264 | 22 | 12 | Bacteroidetes | Bacteroidia | Bacteroidales | Bacteroidaceae | Bacteroides | Bacteroides vulgatus |
| 35508 | 106_3477 | 12 | 5 | Bacteroidetes | Bacteroidia | Bacteroidales | Prevotellaceae | Prevotella | Prevotella stercorea |
| 35512 | 120_2195 | 15 | 9 | Bacteroidetes | Bacteroidia | Bacteroidales | Porphyromonadaceae | Parabacteroides | Parabacteroides merdae |
| 35585 | 105_3784 | 47 | 17 | Bacteroidetes | Bacteroidia | Bacteroidales | Bacteroidaceae | Bacteroides | Bacteroides massiliensis |
| 35586 | 50_1580 | 6 | 5 | Bacteroidetes | Bacteroidia | Bacteroidales | Bacteroidaceae | Bacteroides | Bacteroides massiliensis |
| 35605 | 38_4476 | 12 | 9 | Bacteroidetes | Bacteroidia | Bacteroidales | Porphyromonadaceae | Parabacteroides | Parabacteroides merdae |
| 35608 | 9_2417 | 21 | 14 | Bacteroidetes | Bacteroidia | Bacteroidales | Porphyromonadaceae | Parabacteroides | Parabacteroides merdae |
| 35632 | 78_3852 | 26 | 7 | Bacteroidetes | Bacteroidia | Bacteroidales | Bacteroidaceae | Bacteroides | Bacteroides intestinalis |
| 35702 | 24_5029 | 5 | 5 | Bacteroidetes | Bacteroidia | Bacteroidales | Porphyromonadaceae | Parabacteroides | Parabacteroides distasonis |
| 35744 | 20_5545 | 34 | 3 | Bacteroidetes | Bacteroidia | Bacteroidales | Bacteroidaceae | Bacteroides | Bacteroides eggerthii |
| 35759 | 39_4501 | 8 | 7 | Bacteroidetes | Bacteroidia | Bacteroidales | Porphyromonadaceae | Parabacteroides | Parabacteroides merdae |
| 35783 | 63_6135 | 17 | 6 | Bacteroidetes | Bacteroidia | Bacteroidales | Rikenellaceae | Alistipes | Alistipes sp. |
| 35786 | 75_1215 | 7 | 6 | Bacteroidetes | Bacteroidia | Bacteroidales | Rikenellaceae | Alistipes | Alistipes sp. |
| 35834 | 130_6935 | 28 | 8 | Bacteroidetes | Bacteroidia | Bacteroidales | Bacteroidaceae | Bacteroides | Bacteroides finegoldii |
| 35839 | 108_4943 | 20 | 6 | Bacteroidetes | Bacteroidia | Bacteroidales | Prevotellaceae | Prevotella | Prevotella stercorea |
| 35851 | 130_6538 | 7 | 5 | Bacteroidetes | Bacteroidia | Bacteroidales | Rikenellaceae | Alistipes | Alistipes sp. |
| 35852 | 23_5657 | 8 | 6 | Bacteroidetes | Bacteroidia | Bacteroidales | Prevotellaceae | Prevotella | Prevotella copri |
| 35862 | 57_359 | 8 | 7 | Bacteroidetes | Bacteroidia | Bacteroidales | Porphyromonadaceae | Parabacteroides | Parabacteroides merdae |
| 35895 | 111_9104 | 31 | 3 | Firmicutes | Clostridia | Clostridiales | Lachnospiraceae | Lachnospiraceae incertae sedis | Lachnospiraceae incertae sedis |
| 35932 | 7_4188 | 8 | 8 | Bacteroidetes | Bacteroidia | Bacteroidales | Porphyromonadaceae | Parabacteroides | Parabacteroides merdae |
| 35935 | 120_3541 | 8 | 5 | Bacteroidetes | Bacteroidia | Bacteroidales | Porphyromonadaceae | Parabacteroides | Parabacteroides merdae |
| 35936 | 67_4487 | 5 | 5 | Bacteroidetes | Bacteroidia | Bacteroidales | Bacteroidaceae | Bacteroides | Bacteroides uniformis |
| 35941 | 96_9725 | 13 | 8 | Bacteroidetes | Bacteroidia | Bacteroidales | Bacteroidaceae | Bacteroides | Bacteroides massiliensis |
| 35962 | 39_176 | 7 | 6 | Bacteroidetes | Bacteroidia | Bacteroidales | Bacteroidaceae | Bacteroides | Bacteroides uniformis |
| 35969 | 7_4577 | 11 | 5 | Bacteroidetes | Bacteroidia | Bacteroidales | Prevotellaceae | Prevotella | Prevotella stercorea |
| 35972 | 65_2867 | 10 | 8 | Bacteroidetes | Bacteroidia | Bacteroidales | Bacteroidaceae | Bacteroides | Bacteroides uniformis |
| 35983 | 40_4088 | 9 | 5 | Bacteroidetes | Bacteroidia | Bacteroidales | Bacteroidaceae | Bacteroides | Bacteroides uniformis |
| 36009 | 10_5561 | 35 | 11 | Bacteroidetes | Bacteroidia | Bacteroidales | Rikenellaceae | Alistipes | Alistipes sp. |
| 36022 | 67_7112 | 33 | 12 | Bacteroidetes | Bacteroidia | Bacteroidales | Bacteroidaceae | Bacteroides | Bacteroides massiliensis |
| 36052 | 75_1245 | 9 | 8 | Bacteroidetes | Bacteroidia | Bacteroidales | Rikenellaceae | Alistipes | Alistipes sp. |
| 36099 | 129_6038 | 22 | 10 | Bacteroidetes | Bacteroidia | Bacteroidales | Prevotellaceae | Prevotella | Prevotella stercorea |
| 36138 | 106_4210 | 9 | 6 | Bacteroidetes | Bacteroidia | Bacteroidales | Prevotellaceae | Prevotella | Prevotella stercorea |
| 36183 | 32_2579 | 11 | 6 | Bacteroidetes | Bacteroidia | Bacteroidales | Bacteroidaceae | Bacteroides | Bacteroides dorei |
| 36185 | 115_2907 | 8 | 5 | Bacteroidetes | Bacteroidia | Bacteroidales | Porphyromonadaceae | Parabacteroides | Parabacteroides distasonis |
| 36203 | 17_1730 | 31 | 16 | Bacteroidetes | Bacteroidia | Bacteroidales | Porphyromonadaceae | Parabacteroides | Parabacteroides distasonis |
| 36206 | 30_4690 | 9 | 5 | Bacteroidetes | Bacteroidia | Bacteroidales | Porphyromonadaceae | Parabacteroides | Parabacteroides distasonis |
| 36208 | 65_8194 | 10 | 5 | Bacteroidetes | Bacteroidia | Bacteroidales | Porphyromonadaceae | Parabacteroides | Parabacteroides distasonis |
| 36209 | 76_2969 | 13 | 5 | Bacteroidetes | Bacteroidia | Bacteroidales | Porphyromonadaceae | Parabacteroides | Parabacteroides distasonis |
| 36223 | 129_5301 | 8 | 5 | Bacteroidetes | Bacteroidia | Bacteroidales | Prevotellaceae | Prevotella | Prevotella stercorea |
| 36227 | 65_8081 | 9 | 6 | Bacteroidetes | Bacteroidia | Bacteroidales | Bacteroidaceae | Bacteroides | Bacteroides vulgatus |
| 36232 | 12_4516 | 108 | 21 | Bacteroidetes | Bacteroidia | Bacteroidales | Bacteroidaceae | Bacteroides | Bacteroides vulgatus |
| 36245 | 122_2979 | 44 | 9 | Bacteroidetes | Bacteroidia | Bacteroidales | Bacteroidaceae | Bacteroides | Bacteroides dorei |
| 36248 | 125_4421 | 28 | 13 | Bacteroidetes | Bacteroidia | Bacteroidales | Bacteroidaceae | Bacteroides | Bacteroides vulgatus |
| 36259 | 123_4554 | 18 | 10 | Bacteroidetes | Bacteroidia | Bacteroidales | Bacteroidaceae | Bacteroides | Bacteroides vulgatus |
| 36266 | 111_7926 | 10 | 8 | Bacteroidetes | Bacteroidia | Bacteroidales | Porphyromonadaceae | Parabacteroides | Parabacteroides distasonis |
| 36269 | 12_4701 | 111 | 15 | Bacteroidetes | Bacteroidia | Bacteroidales | Bacteroidaceae | Bacteroides | Bacteroides vulgatus |
| 36270 | 42_6696 | 11 | 5 | Bacteroidetes | Bacteroidia | Bacteroidales | Bacteroidaceae | Bacteroides | Bacteroides vulgatus |
| 36272 | 22_2199 | 13 | 11 | Bacteroidetes | Bacteroidia | Bacteroidales | Porphyromonadaceae | Parabacteroides | Parabacteroides distasonis |
| 36297 | 50_1294 | 32 | 9 | Bacteroidetes | Bacteroidia | Bacteroidales | Porphyromonadaceae | Parabacteroides | Parabacteroides distasonis |
| 36349 | 105_1824 | 13 | 5 | Bacteroidetes | Bacteroidia | Bacteroidales | Bacteroidaceae | Bacteroides | Bacteroides ovatus |
| 36375 | 122_2945 | 10 | 6 | Bacteroidetes | Bacteroidia | Bacteroidales | Bacteroidaceae | Bacteroides | Bacteroides dorei |
| 36388 | 67_8017 | 84 | 12 | Bacteroidetes | Bacteroidia | Bacteroidales | Bacteroidaceae | Bacteroides | Bacteroides vulgatus |
| 36392 | 2_3441 | 86 | 10 | Bacteroidetes | Bacteroidia | Bacteroidales | Bacteroidaceae | Bacteroides | Bacteroides intestinalis |
| 36397 | 108_3746 | 28 | 7 | Bacteroidetes | Bacteroidia | Bacteroidales | Bacteroidaceae | Bacteroides | Bacteroides intestinalis |
| 36433 | 7_2365 | 8 | 5 | Bacteroidetes | Bacteroidia | Bacteroidales | Prevotellaceae | Prevotella | Prevotella stercorea |
| 36435 | 42_1947 | 31 | 13 | Bacteroidetes | Bacteroidia | Bacteroidales | Bacteroidaceae | Bacteroides | Bacteroides vulgatus |
| 36447 | 124_879 | 10014 | 91 | Bacteroidetes | Bacteroidia | Bacteroidales | Bacteroidaceae | Bacteroides | Bacteroides vulgatus |
| 36449 | 115_929 | 13 | 8 | Bacteroidetes | Bacteroidia | Bacteroidales | Porphyromonadaceae | Parabacteroides | Parabacteroides distasonis |
| 36460 | 23_5350 | 16 | 7 | Bacteroidetes | Bacteroidia | Bacteroidales | Bacteroidaceae | Bacteroides | Bacteroides salyersiae |
| 36466 | 130_5590 | 26 | 10 | Bacteroidetes | Bacteroidia | Bacteroidales | Bacteroidaceae | Bacteroides | Bacteroides vulgatus |
| 36477 | 37_1799 | 8 | 7 | Bacteroidetes | Bacteroidia | Bacteroidales | Bacteroidaceae | Bacteroides | Bacteroides vulgatus |
| 36481 | 12_4591 | 8199 | 90 | Bacteroidetes | Bacteroidia | Bacteroidales | Bacteroidaceae | Bacteroides | Bacteroides vulgatus |
| 36490 | 12_2549 | 221 | 36 | Bacteroidetes | Bacteroidia | Bacteroidales | Bacteroidaceae | Bacteroides | Bacteroides vulgatus |
| 36492 | 12_2441 | 142 | 21 | Bacteroidetes | Bacteroidia | Bacteroidales | Bacteroidaceae | Bacteroides | Bacteroides vulgatus |
| 36516 | 132_1665 | 8 | 6 | Bacteroidetes | Bacteroidia | Bacteroidales | Bacteroidaceae | Bacteroides | Bacteroides vulgatus |
| 36546 | 63_1048 | 9 | 5 | Bacteroidetes | Bacteroidia | Bacteroidales | Bacteroidaceae | Bacteroides | Bacteroides uniformis |
| 36558 | 107_260 | 12 | 9 | Bacteroidetes | Bacteroidia | Bacteroidales | Rikenellaceae | Alistipes | Alistipes putredinis |
| 36577 | 12_4271 | 13 | 7 | Bacteroidetes | Bacteroidia | Bacteroidales | Bacteroidaceae | Bacteroides | Bacteroides vulgatus |
| 36583 | 65_4516 | 11 | 6 | Bacteroidetes | Bacteroidia | Bacteroidales | Bacteroidaceae | Bacteroides | Bacteroides vulgatus |
| 36591 | 42_6859 | 7 | 5 | Bacteroidetes | Bacteroidia | Bacteroidales | Bacteroidaceae | Bacteroides | Bacteroides vulgatus |
| 36597 | 65_4251 | 12 | 6 | Bacteroidetes | Bacteroidia | Bacteroidales | Bacteroidaceae | Bacteroides | Bacteroides vulgatus |
| 36603 | 9_5076 | 39 | 17 | Bacteroidetes | Bacteroidia | Bacteroidales | Bacteroidaceae | Bacteroides | Bacteroides vulgatus |
| 36604 | 12_1937 | 286 | 23 | Bacteroidetes | Bacteroidia | Bacteroidales | Bacteroidaceae | Bacteroides | Bacteroides vulgatus |
| 36607 | 42_3437 | 281 | 27 | Bacteroidetes | Bacteroidia | Bacteroidales | Bacteroidaceae | Bacteroides | Bacteroides vulgatus |
| 36614 | 102_1345 | 15 | 9 | Bacteroidetes | Bacteroidia | Bacteroidales | Porphyromonadaceae | Parabacteroides | Parabacteroides distasonis |
| 36617 | 10_3127 | 16 | 11 | Bacteroidetes | Bacteroidia | Bacteroidales | Rikenellaceae | Alistipes | Alistipes sp. |
| 36627 | 4_2521 | 7 | 6 | Bacteroidetes | Bacteroidia | Bacteroidales | Bacteroidaceae | Bacteroides | Bacteroides vulgatus |
| 36628 | 63_6466 | 17 | 9 | Bacteroidetes | Bacteroidia | Bacteroidales | Bacteroidaceae | Bacteroides | Bacteroides vulgatus |
| 36635 | 108_6283 | 2648 | 57 | Bacteroidetes | Bacteroidia | Bacteroidales | Bacteroidaceae | Bacteroides | Bacteroides vulgatus |
| 36648 | 108_4672 | 8 | 6 | Bacteroidetes | Bacteroidia | Bacteroidales | Bacteroidaceae | Bacteroides | Bacteroides vulgatus |
| 36691 | 1_3114 | 12 | 7 | Bacteroidetes | Bacteroidia | Bacteroidales | Rikenellaceae | Alistipes | Alistipes shahii |
| 36752 | 51_1085 | 8 | 5 | Bacteroidetes | Bacteroidia | Bacteroidales | Prevotellaceae | Prevotella | Prevotella sp. |
| 36795 | 48_3391 | 37 | 3 | Bacteroidetes | Bacteroidia | Bacteroidales | Prevotellaceae | Prevotella | Prevotella copri |
| 36829 | 1_3403 | 31 | 8 | Bacteroidetes | Bacteroidia | Bacteroidales | Prevotellaceae | Prevotella | Prevotella copri |
| 36832 | 81_2749 | 8 | 5 | Bacteroidetes | Bacteroidia | Bacteroidales | Prevotellaceae | Prevotella | Prevotella copri |
| 36844 | 112_378 | 62 | 7 | Bacteroidetes | Bacteroidia | Bacteroidales | Prevotellaceae | Prevotella | Prevotella copri |
| 36851 | 1_3625 | 29 | 10 | Bacteroidetes | Bacteroidia | Bacteroidales | Prevotellaceae | Prevotella | Prevotella copri |
| 36854 | 1_2305 | 35 | 5 | Bacteroidetes | Bacteroidia | Bacteroidales | Prevotellaceae | Prevotella | Prevotella salivae |
| 36863 | 106_2669 | 17 | 6 | Bacteroidetes | Bacteroidia | Bacteroidales | Bacteroidaceae | Bacteroides | Bacteroides sp. |
| 36865 | 106_3751 | 317 | 8 | Bacteroidetes | Bacteroidia | Bacteroidales | Prevotellaceae | Prevotella | Prevotella stercorea |
| 36874 | 104_256 | 3375 | 37 | Bacteroidetes | Bacteroidia | Bacteroidales | Prevotellaceae | Prevotella | Prevotella stercorea |
| 36917 | 117_1599 | 7 | 5 | Bacteroidetes | Bacteroidia | Bacteroidales | Prevotellaceae | Prevotella | Prevotella stercorea |
| 37008 | 104_879 | 5 | 5 | Bacteroidetes | Bacteroidia | Bacteroidales | Prevotellaceae | Prevotella | Prevotella stercorea |
| 37014 | 4_3503 | 10 | 7 | Bacteroidetes | Bacteroidia | Bacteroidales | Bacteroidaceae | Bacteroides | Bacteroides vulgatus |
| 37021 | 115_3230 | 25 | 10 | Bacteroidetes | Bacteroidia | Bacteroidales | Prevotellaceae | Prevotella | Prevotella copri |
| 37046 | 121_6519 | 6 | 5 | Bacteroidetes | Bacteroidia | Bacteroidales | Prevotellaceae | Prevotella | Prevotella copri |
| 37047 | 104_1484 | 9 | 6 | Bacteroidetes | Bacteroidia | Bacteroidales | Prevotellaceae | Prevotella | Prevotella copri |
| 37076 | 115_1917 | 5 | 5 | Bacteroidetes | Bacteroidia | Bacteroidales | Prevotellaceae | Prevotella | Prevotella copri |
| 37101 | 59_4184 | 6 | 5 | Bacteroidetes | Bacteroidia | Bacteroidales | Prevotellaceae | Prevotella | Prevotella copri |
| 37103 | 123_7574 | 5 | 5 | Bacteroidetes | Bacteroidia | Bacteroidales | Prevotellaceae | Prevotella | Prevotella copri |
| 37104 | 123_7066 | 9 | 6 | Bacteroidetes | Bacteroidia | Bacteroidales | Prevotellaceae | Prevotella | Prevotella copri |
| 37156 | 123_3247 | 7 | 5 | Bacteroidetes | Bacteroidia | Bacteroidales | Prevotellaceae | Prevotella | Prevotella copri |
| 37175 | 81_1911 | 13 | 7 | Bacteroidetes | Bacteroidia | Bacteroidales | Prevotellaceae | Prevotella | Prevotella stercorea |
| 37209 | 54_6934 | 10 | 5 | Bacteroidetes | Bacteroidia | Bacteroidales | Prevotellaceae | Prevotella | Prevotella copri |
| 37235 | 81_391 | 20 | 5 | NULL | NULL | NULL | NULL | NULL | NULL |
| 37240 | 81_2008 | 12 | 5 | Bacteroidetes | Bacteroidia | Bacteroidales | Bacteroidaceae | Bacteroides | Bacteroides plebeius |
| 37249 | 48_5133 | 18 | 6 | Bacteroidetes | Bacteroidia | Bacteroidales | Prevotellaceae | Prevotella | Prevotella copri |
| 37292 | 104_1555 | 29 | 6 | Bacteroidetes | Bacteroidia | Bacteroidales | Prevotellaceae | Prevotella | Prevotella copri |
| 37395 | 48_3565 | 36 | 10 | Bacteroidetes | Bacteroidia | Bacteroidales | Prevotellaceae | Prevotella | Prevotella copri |
| 37415 | 4_1334 | 8 | 5 | Bacteroidetes | Bacteroidia | Bacteroidales | Prevotellaceae | Prevotella | Prevotella copri |
| 37454 | 108_7032 | 23 | 8 | Bacteroidetes | Bacteroidia | Bacteroidales | Bacteroidaceae | Bacteroides | Bacteroides vulgatus |
| 37473 | 107_4043 | 13 | 6 | Bacteroidetes | Bacteroidia | Bacteroidales | Bacteroidaceae | Bacteroides | Bacteroides vulgatus |
| 37486 | 119_4241 | 6 | 5 | Bacteroidetes | Bacteroidia | Bacteroidales | Bacteroidaceae | Bacteroides | Bacteroides sp. |
| 37494 | 54_3626 | 15 | 6 | Bacteroidetes | Bacteroidia | Bacteroidales | Prevotellaceae | Prevotella | Prevotella copri |
| 37495 | 112_2044 | 23 | 7 | Bacteroidetes | Bacteroidia | Bacteroidales | Prevotellaceae | Prevotella | Prevotella copri |
| 37546 | 9_5875 | 12 | 7 | Bacteroidetes | Bacteroidia | Bacteroidales | Porphyromonadaceae | Parabacteroides | Parabacteroides merdae |
| 37550 | 55_930 | 2755 | 16 | Proteobacteria | Gammaproteobacteria | Enterobacteriales | Enterobacteriaceae | Escherichia/Shigella | Escherichia/Shigella |
| 37553 | 120_874 | 6 | 5 | Bacteroidetes | Bacteroidia | Bacteroidales | Bacteroidaceae | Bacteroides | Bacteroides finegoldii |
| 37557 | 25_2652 | 382 | 23 | Firmicutes | Erysipelotrichia | Erysipelotrichales | Erysipelotrichaceae | Catenibacterium | Catenibacterium mitsuokai |
| 37560 | 49_2823 | 6 | 5 | NULL | NULL | NULL | NULL | NULL | NULL |
| 37561 | 122_2167 | 53 | 15 | Bacteroidetes | Bacteroidia | Bacteroidales | Porphyromonadaceae | Odoribacter | Odoribacter splanchnicus |
| 37571 | 105_6039 | 66 | 11 | Bacteroidetes | Bacteroidia | Bacteroidales | Bacteroidaceae | Bacteroides | Bacteroides vulgatus |
| 37574 | 42_6492 | 100 | 12 | Bacteroidetes | Bacteroidia | Bacteroidales | Bacteroidaceae | Bacteroides | Bacteroides vulgatus |
| 37609 | 9_5982 | 63 | 3 | Bacteroidetes | Bacteroidia | Bacteroidales | Prevotellaceae | Prevotella | Prevotella copri |
| 37643 | 86_3156 | 13 | 6 | Firmicutes | Negativicutes | Selenomonadales | Veillonellaceae | Dialister | Dialister invisus |
| 37745 | 41_8815 | 11 | 6 | Bacteroidetes | Bacteroidia | Bacteroidales | Bacteroidaceae | Bacteroides | Bacteroides dorei |
| 37756 | 115_432 | 29 | 18 | Bacteroidetes | Bacteroidia | Bacteroidales | Bacteroidaceae | Bacteroides | Bacteroides vulgatus |
| 37773 | 106_4059 | 579 | 11 | Bacteroidetes | Bacteroidia | Bacteroidales | Prevotellaceae | Prevotella | Prevotella stercorea |
| 37774 | 62_1781 | 45 | 8 | Bacteroidetes | Bacteroidia | Bacteroidales | Bacteroidaceae | Bacteroides | Bacteroides massiliensis |
| 37789 | 102_2769 | 66 | 28 | Firmicutes | Clostridia | Clostridiales | Ruminococcaceae | Ruminococcus | Ruminococcus faecis |
| 37790 | 66_2557 | 35 | 21 | Firmicutes | Clostridia | Clostridiales | Lachnospiraceae | Coprococcus | Coprococcus comes |
| 37807 | 66_1121 | 146 | 43 | Firmicutes | Clostridia | Clostridiales | Lachnospiraceae | Blautia | Blautia |
| 37816 | 123_7625 | 82 | 23 | Firmicutes | Clostridia | Clostridiales | Lachnospiraceae | Roseburia | Roseburia inulinivorans |
| 37817 | 97_4909 | 6 | 5 | Firmicutes | Clostridia | Clostridiales | Lachnospiraceae | Roseburia | Roseburia intestinalis |
| 37818 | 132_1459 | 8 | 6 | Firmicutes | Clostridia | Clostridiales | Lachnospiraceae | Roseburia | Roseburia intestinalis |
| 37827 | 58_6608 | 744 | 72 | Firmicutes | Clostridia | Clostridiales | Lachnospiraceae | Roseburia | Roseburia intestinalis |
| 37828 | 105_3686 | 156 | 31 | Firmicutes | Clostridia | Clostridiales | Lachnospiraceae | Roseburia | Roseburia inulinivorans |
| 37834 | 105_3058 | 1015 | 66 | Firmicutes | Clostridia | Clostridiales | Lachnospiraceae | Roseburia | Roseburia inulinivorans |
| 37865 | 60_3204 | 6 | 5 | Firmicutes | Negativicutes | Selenomonadales | Veillonellaceae | Dialister | Dialister invisus |
| 37876 | 17_3285 | 6 | 5 | Firmicutes | Negativicutes | Selenomonadales | Veillonellaceae | Dialister | Dialister invisus |
| 37931 | 5_628 | 30 | 7 | Firmicutes | Erysipelotrichia | Erysipelotrichales | Erysipelotrichaceae | Catenibacterium | Catenibacterium mitsuokai |
| 37937 | 25_660 | 59 | 19 | Firmicutes | Erysipelotrichia | Erysipelotrichales | Erysipelotrichaceae | Catenibacterium | Catenibacterium mitsuokai |
| 37940 | 1_3705 | 901 | 34 | Firmicutes | Erysipelotrichia | Erysipelotrichales | Erysipelotrichaceae | Catenibacterium | Catenibacterium mitsuokai |
| 37942 | 66_1751 | 105 | 15 | Firmicutes | Erysipelotrichia | Erysipelotrichales | Erysipelotrichaceae | Catenibacterium | Catenibacterium mitsuokai |
| 37943 | 4_1327 | 9 | 6 | Firmicutes | Erysipelotrichia | Erysipelotrichales | Erysipelotrichaceae | Catenibacterium | Catenibacterium mitsuokai |
| 37951 | 1_248 | 244 | 22 | Firmicutes | Erysipelotrichia | Erysipelotrichales | Erysipelotrichaceae | Catenibacterium | Catenibacterium mitsuokai |
| 37961 | 1_1360 | 29 | 6 | Firmicutes | Erysipelotrichia | Erysipelotrichales | Erysipelotrichaceae | Catenibacterium | Catenibacterium mitsuokai |
| 37967 | 1_1390 | 21 | 6 | Firmicutes | Erysipelotrichia | Erysipelotrichales | Erysipelotrichaceae | Catenibacterium | Catenibacterium mitsuokai |
| 38032 | 49_3787 | 11 | 6 | Firmicutes | Negativicutes | Selenomonadales | Veillonellaceae | Dialister | Dialister invisus |
| 38065 | 48_2359 | 5 | 5 | Firmicutes | Clostridia | Clostridiales | Ruminococcaceae | Faecalibacterium | Faecalibacterium prausnitzii |
| 38075 | 128_2346 | 31 | 2 | Firmicutes | Negativicutes | Selenomonadales | Veillonellaceae | Megasphaera | Megasphaera elsdenii |
| 38097 | 117_3238 | 10 | 6 | Firmicutes | Negativicutes | Selenomonadales | Veillonellaceae | Dialister | Dialister invisus |
| 38120 | 128_2528 | 9 | 7 | Firmicutes | Negativicutes | Selenomonadales | Veillonellaceae | Dialister | Dialister invisus |
| 38142 | 62_6208 | 298 | 37 | Firmicutes | Clostridia | Clostridiales | Ruminococcaceae | Ruminococcus | Ruminococcus |
| 38196 | 37_1329 | 11 | 5 | Firmicutes | Clostridia | Clostridiales | Lachnospiraceae | Roseburia | Eubacterium rectale |
| 38211 | 49_1353 | 12 | 9 | Bacteroidetes | Bacteroidia | Bacteroidales | Bacteroidaceae | Bacteroides | Bacteroides vulgatus |
| 38246 | 96_4760 | 14 | 5 | NULL | NULL | NULL | NULL | NULL | NULL |
| 38258 | 122_2015 | 334 | 28 | Firmicutes | Clostridia | Clostridiales | Ruminococcaceae | Ruminococcus | Ruminococcus albus |
| 38295 | 123_7255 | 41 | 18 | Firmicutes | Clostridia | Clostridiales | Oscillospiraceae | Oscillibacter | Oscillibacter |
| 38296 | 57_529 | 110 | 41 | Firmicutes | Clostridia | Clostridiales | Oscillospiraceae | Oscillibacter | Oscillibacter |
| 38318 | 62_2561 | 219 | 37 | Firmicutes | Erysipelotrichia | Erysipelotrichales | Erysipelotrichaceae | Erysipelotrichaceae incertae sedis | Erysipelotrichaceae incertae sedis |
| 38373 | 97_3991 | 9 | 6 | NULL | NULL | NULL | NULL | NULL | NULL |
| 38404 | 33_3323 | 27 | 8 | Bacteroidetes | Bacteroidia | Bacteroidales | Prevotellaceae | Prevotella | Prevotella copri |
| 38441 | 73_942 | 12 | 9 | Firmicutes | Clostridia | Clostridiales | Ruminococcaceae | Faecalibacterium | Faecalibacterium prausnitzii |
| 38464 | 55_2425 | 10 | 6 | Proteobacteria | Gammaproteobacteria | Enterobacteriales | Enterobacteriaceae | Escherichia/Shigella | Escherichia/Shigella |
| 38470 | 76_2047 | 45 | 26 | Firmicutes | Clostridia | Clostridiales | Ruminococcaceae | incertae sedis | Ruminococcaceae incertae sedis |
| 38474 | 5_417 | 32 | 17 | Proteobacteria | Betaproteobacteria | Burkholderiales | Oxalobacteraceae | Oxalobacter | Oxalobacter formigenes |
| 38506 | 119_5959 | 17 | 12 | Firmicutes | Clostridia | Clostridiales | Lachnospiraceae | Blautia | Blautia |
| 38521 | 15_5544 | 52 | 12 | Firmicutes | Clostridia | Clostridiales | Lachnospiraceae | Lachnospiraceae incertae sedis | Lachnospiraceae incertae sedis |
| 38531 | 55_4637 | 123 | 36 | Firmicutes | Clostridia | Clostridiales | Lachnospiraceae | Blautia | Blautia |
| 38560 | 25_442 | 62 | 30 | Firmicutes | Clostridia | Clostridiales | Lachnospiraceae | Roseburia | Eubacterium rectale |
| 38586 | 84_4616 | 189 | 56 | Firmicutes | Clostridia | Clostridiales | Lachnospiraceae | Blautia | Blautia |
| 38587 | 65_6204 | 57 | 26 | Firmicutes | Clostridia | Clostridiales | Lachnospiraceae | Roseburia | Eubacterium rectale |
| 38596 | 102_6052 | 270 | 22 | Firmicutes | Clostridia | Clostridiales | Lachnospiraceae | Blautia | Blautia |
| 38607 | 49_4038 | 7 | 5 | Firmicutes | Negativicutes | Selenomonadales | Veillonellaceae | Dialister | Dialister invisus |
| 38644 | 11_940 | 13 | 8 | Firmicutes | Clostridia | Clostridiales | Lachnospiraceae | Blautia | Blautia |
| 38646 | 73_615 | 20 | 13 | Firmicutes | Clostridia | Clostridiales | Ruminococcaceae | Faecalibacterium | Faecalibacterium prausnitzii |
| 38726 | 124_6903 | 19 | 12 | Firmicutes | Clostridia | Clostridiales | Eubacteriaceae | Eubacterium | Eubacterium desmolans |
| 38727 | 97_3517 | 79 | 34 | Firmicutes | Clostridia | Clostridiales | Eubacteriaceae | Eubacterium | Eubacterium desmolans |
| 38729 | 18_6528 | 33 | 14 | Firmicutes | Clostridia | Clostridiales | Eubacteriaceae | Eubacterium | Eubacterium desmolans |
| 38730 | 101_5344 | 36 | 19 | Firmicutes | Clostridia | Clostridiales | Eubacteriaceae | Eubacterium | Eubacterium desmolans |
| 38738 | 115_1188 | 9 | 5 | Firmicutes | Clostridia | Clostridiales | Lachnospiraceae | Blautia | Blautia |
| 38819 | 55_7101 | 23 | 7 | Proteobacteria | Gammaproteobacteria | Enterobacteriales | Enterobacteriaceae | Escherichia/Shigella | Escherichia/Shigella |
| 38823 | 41_3355 | 8 | 5 | Proteobacteria | Gammaproteobacteria | Enterobacteriales | Enterobacteriaceae | Escherichia/Shigella | Escherichia/Shigella |
| 38824 | 55_6486 | 52 | 10 | Proteobacteria | Gammaproteobacteria | Enterobacteriales | Enterobacteriaceae | Escherichia/Shigella | Escherichia/Shigella |
| 38826 | 71_614 | 283 | 17 | Proteobacteria | Gammaproteobacteria | Enterobacteriales | Enterobacteriaceae | Escherichia/Shigella | Escherichia/Shigella |
| 38863 | 107_1859 | 52 | 20 | Proteobacteria | Deltaproteobacteria | Desulfovibrionales | Desulfovibrionaceae | Desulfovibrio | Desulfovibrio |
| 38874 | 102_3323 | 11 | 5 | Bacteroidetes | Bacteroidia | Bacteroidales | Porphyromonadaceae | Parabacteroides | Parabacteroides distasonis |
| 38914 | 123_3389 | 104 | 14 | Bacteroidetes | Bacteroidia | Bacteroidales | Bacteroidaceae | Bacteroides | Bacteroides vulgatus |
| 38966 | 126_501 | 14 | 5 | Bacteroidetes | Bacteroidia | Bacteroidales | Porphyromonadaceae | Butyricimonas | Butyricimonas virosa |
| 38979 | 113_2086 | 7 | 6 | Bacteroidetes | Bacteroidia | Bacteroidales | Porphyromonadaceae | Parabacteroides | Parabacteroides merdae |
| 39025 | 50_344 | 18 | 13 | Bacteroidetes | Bacteroidia | Bacteroidales | Porphyromonadaceae | Parabacteroides | Parabacteroides merdae |
| 39031 | 21_6465 | 11 | 10 | Bacteroidetes | Bacteroidia | Bacteroidales | Porphyromonadaceae | Parabacteroides | Parabacteroides merdae |
| 39036 | 21_6708 | 7 | 6 | Bacteroidetes | Bacteroidia | Bacteroidales | Porphyromonadaceae | Parabacteroides | Parabacteroides distasonis |
| 39037 | 119_5902 | 19 | 9 | Bacteroidetes | Bacteroidia | Bacteroidales | Porphyromonadaceae | Parabacteroides | Parabacteroides merdae |
| 39038 | 55_6565 | 18 | 13 | Bacteroidetes | Bacteroidia | Bacteroidales | Porphyromonadaceae | Parabacteroides | Parabacteroides merdae |
| 39041 | 38_6841 | 12 | 10 | Bacteroidetes | Bacteroidia | Bacteroidales | Porphyromonadaceae | Parabacteroides | Parabacteroides merdae |
| 39095 | 58_4025 | 58 | 3 | Bacteroidetes | Bacteroidia | Bacteroidales | Bacteroidaceae | Bacteroides | Bacteroides vulgatus |
| 39106 | 50_1204 | 9 | 7 | Bacteroidetes | Bacteroidia | Bacteroidales | Porphyromonadaceae | Parabacteroides | Parabacteroides distasonis |
| 39107 | 26_2175 | 11 | 6 | Bacteroidetes | Bacteroidia | Bacteroidales | Porphyromonadaceae | Odoribacter | Odoribacter splanchnicus |
| 39110 | 26_2739 | 151 | 36 | Bacteroidetes | Bacteroidia | Bacteroidales | Porphyromonadaceae | Odoribacter | Odoribacter splanchnicus |
| 39117 | 129_5311 | 6 | 6 | Bacteroidetes | Bacteroidia | Bacteroidales | Bacteroidaceae | Bacteroides | Bacteroides uniformis |
| 39125 | 107_3006 | 15 | 8 | Bacteroidetes | Bacteroidia | Bacteroidales | Bacteroidaceae | Bacteroides | Bacteroides caccae |
| 39127 | 61_2052 | 12 | 10 | Bacteroidetes | Bacteroidia | Bacteroidales | Porphyromonadaceae | Butyricimonas | Butyricimonas virosa |
| 39136 | 113_699 | 32 | 8 | Bacteroidetes | Bacteroidia | Bacteroidales | Bacteroidaceae | Bacteroides | Bacteroides intestinalis |
| 39139 | 32_5373 | 31 | 11 | Bacteroidetes | Bacteroidia | Bacteroidales | Bacteroidaceae | Bacteroides | Bacteroides uniformis |
| 39140 | 32_3320 | 67 | 8 | Bacteroidetes | Bacteroidia | Bacteroidales | Bacteroidaceae | Bacteroides | Bacteroides intestinalis |
| 39142 | 101_666 | 12 | 6 | Bacteroidetes | Bacteroidia | Bacteroidales | Bacteroidaceae | Bacteroides | Bacteroides uniformis |
| 39145 | 91_1324 | 31 | 5 | Bacteroidetes | Bacteroidia | Bacteroidales | Bacteroidaceae | Bacteroides | Bacteroides intestinalis |
| 39146 | 32_6832 | 199 | 11 | Bacteroidetes | Bacteroidia | Bacteroidales | Bacteroidaceae | Bacteroides | Bacteroides intestinalis |
| 39170 | 80_1324 | 40 | 4 | Bacteroidetes | Bacteroidia | Bacteroidales | Bacteroidaceae | Bacteroides | Bacteroides intestinalis |
| 39177 | 8_908 | 9 | 6 | Bacteroidetes | Bacteroidia | Bacteroidales | Rikenellaceae | Alistipes | Alistipes finegoldii |
| 39181 | 61_5622 | 17 | 8 | Bacteroidetes | Bacteroidia | Bacteroidales | Rikenellaceae | Alistipes | Alistipes shahii |
| 39189 | 26_2706 | 37 | 13 | Bacteroidetes | Bacteroidia | Bacteroidales | Rikenellaceae | Alistipes | Alistipes finegoldii |
| 39198 | 39_3230 | 6 | 5 | Bacteroidetes | Bacteroidia | Bacteroidales | Rikenellaceae | Alistipes | Alistipes shahii |
| 39208 | 62_5961 | 25 | 13 | Bacteroidetes | Bacteroidia | Bacteroidales | Rikenellaceae | Alistipes | Alistipes shahii |
| 39210 | 63_2788 | 12 | 8 | Bacteroidetes | Bacteroidia | Bacteroidales | Bacteroidaceae | Bacteroides | Bacteroides eggerthii |
| 39212 | 97_2541 | 7 | 5 | Bacteroidetes | Bacteroidia | Bacteroidales | Bacteroidaceae | Bacteroides | Bacteroides massiliensis |
| 39221 | 84_4347 | 6 | 5 | Bacteroidetes | Bacteroidia | Bacteroidales | Rikenellaceae | Alistipes | Alistipes finegoldii |
| 39243 | 50_1312 | 8 | 5 | Bacteroidetes | Bacteroidia | Bacteroidales | Rikenellaceae | Alistipes | Alistipes sp. |
| 39273 | 16_4885 | 5 | 5 | Bacteroidetes | Bacteroidia | Bacteroidales | Rikenellaceae | Alistipes | Alistipes finegoldii |
| 39278 | 50_1517 | 7 | 5 | Bacteroidetes | Bacteroidia | Bacteroidales | Rikenellaceae | Alistipes | Alistipes putredinis |
| 39287 | 17_1767 | 19 | 11 | Bacteroidetes | Bacteroidia | Bacteroidales | Rikenellaceae | Alistipes | Alistipes onderdonkii |
| 39397 | 58_7878 | 27 | 2 | Bacteroidetes | Bacteroidia | Bacteroidales | Bacteroidaceae | Bacteroides | Bacteroides plebeius |
| 39403 | 82_3946 | 9 | 5 | Bacteroidetes | Bacteroidia | Bacteroidales | Bacteroidaceae | Bacteroides | Bacteroides massiliensis |
| 39410 | 105_3593 | 9 | 5 | Bacteroidetes | Bacteroidia | Bacteroidales | Bacteroidaceae | Bacteroides | Bacteroides dorei |
| 39416 | 113_1603 | 19 | 11 | Bacteroidetes | Bacteroidia | Bacteroidales | Bacteroidaceae | Bacteroides | Bacteroides ovatus |
| 39440 | 7_405 | 17 | 6 | Bacteroidetes | Bacteroidia | Bacteroidales | Prevotellaceae | Prevotella | Prevotella stercorea |
| 39513 | 131_8277 | 18 | 8 | Bacteroidetes | Bacteroidia | Bacteroidales | Bacteroidaceae | Bacteroides | Bacteroides uniformis |
| 39544 | 4_1556 | 16 | 6 | Bacteroidetes | Bacteroidia | Bacteroidales | Bacteroidaceae | Bacteroides | Bacteroides salyersiae |
| 39603 | 105_3697 | 10 | 7 | Bacteroidetes | Bacteroidia | Bacteroidales | Bacteroidaceae | Bacteroides | Bacteroides caccae |
| 39604 | 9_5483 | 8 | 6 | Bacteroidetes | Bacteroidia | Bacteroidales | Bacteroidaceae | Bacteroides | Bacteroides sp. |
| 39671 | 12_4572 | 14 | 5 | Bacteroidetes | Bacteroidia | Bacteroidales | Bacteroidaceae | Bacteroides | Bacteroides fragilis |
| 39677 | 22_6117 | 13 | 8 | Bacteroidetes | Bacteroidia | Bacteroidales | Bacteroidaceae | Bacteroides | Bacteroides nordii |
| 39699 | 67_8340 | 16 | 6 | Bacteroidetes | Bacteroidia | Bacteroidales | Bacteroidaceae | Bacteroides | Bacteroides massiliensis |
| 39720 | 122_454 | 12 | 7 | Bacteroidetes | Bacteroidia | Bacteroidales | Bacteroidaceae | Bacteroides | Bacteroides massiliensis |
| 39735 | 61_5538 | 11 | 5 | Bacteroidetes | Bacteroidia | Bacteroidales | Bacteroidaceae | Bacteroides | Bacteroides intestinalis |
| 39736 | 107_4145 | 17 | 8 | Bacteroidetes | Bacteroidia | Bacteroidales | Bacteroidaceae | Bacteroides | Bacteroides salyersiae |
| 39746 | 96_4660 | 52 | 8 | Bacteroidetes | Bacteroidia | Bacteroidales | Bacteroidaceae | Bacteroides | Bacteroides massiliensis |
| 39776 | 19_5697 | 18 | 12 | Bacteroidetes | Bacteroidia | Bacteroidales | Porphyromonadaceae | Parabacteroides | Parabacteroides distasonis |
| 39814 | 105_948 | 14 | 10 | Bacteroidetes | Bacteroidia | Bacteroidales | Bacteroidaceae | Bacteroides | Bacteroides massiliensis |
| 39816 | 24_4225 | 17 | 10 | Bacteroidetes | Bacteroidia | Bacteroidales | Bacteroidaceae | Bacteroides | Bacteroides massiliensis |
| 39844 | 20_4237 | 43 | 4 | Bacteroidetes | Bacteroidia | Bacteroidales | Bacteroidaceae | Bacteroides | Bacteroides sp. |
| 39873 | 20_8700 | 26 | 3 | Bacteroidetes | Bacteroidia | Bacteroidales | Bacteroidaceae | Bacteroides | Bacteroides eggerthii |
| 39875 | 20_6834 | 28 | 3 | Bacteroidetes | Bacteroidia | Bacteroidales | Bacteroidaceae | Bacteroides | Bacteroides eggerthii |
| 39886 | 110_1752 | 5 | 5 | Bacteroidetes | Bacteroidia | Bacteroidales | Bacteroidaceae | Bacteroides | Bacteroides eggerthii |
| 39896 | 120_3090 | 7 | 6 | Bacteroidetes | Bacteroidia | Bacteroidales | Bacteroidaceae | Bacteroides | Bacteroides uniformis |
| 39921 | 63_2516 | 10 | 7 | Bacteroidetes | Bacteroidia | Bacteroidales | Bacteroidaceae | Bacteroides | Bacteroides eggerthii |
| 39964 | 108_1324 | 88 | 26 | Bacteroidetes | Bacteroidia | Bacteroidales | Bacteroidaceae | Bacteroides | Bacteroides vulgatus |
| 39971 | 124_3206 | 27 | 13 | Bacteroidetes | Bacteroidia | Bacteroidales | Bacteroidaceae | Bacteroides | Bacteroides massiliensis |
| 39980 | 65_7587 | 13 | 7 | Bacteroidetes | Bacteroidia | Bacteroidales | Porphyromonadaceae | Parabacteroides | Parabacteroides distasonis |
| 40015 | 97_4633 | 10 | 5 | Bacteroidetes | Bacteroidia | Bacteroidales | Porphyromonadaceae | Odoribacter | Odoribacter splanchnicus |
| 40018 | 10_5395 | 21 | 5 | Bacteroidetes | Bacteroidia | Bacteroidales | Bacteroidaceae | Bacteroides | Bacteroides uniformis |
| 40027 | 12_3663 | 10 | 7 | Bacteroidetes | Bacteroidia | Bacteroidales | Porphyromonadaceae | Parabacteroides | Parabacteroides distasonis |
| 40064 | 19_6441 | 8 | 6 | Bacteroidetes | Bacteroidia | Bacteroidales | Porphyromonadaceae | Parabacteroides | Parabacteroides distasonis |
| 40091 | 62_4460 | 8 | 6 | Bacteroidetes | Bacteroidia | Bacteroidales | Bacteroidaceae | Bacteroides | Bacteroides uniformis |
| 40092 | 97_3790 | 23 | 7 | Bacteroidetes | Bacteroidia | Bacteroidales | Bacteroidaceae | Bacteroides | Bacteroides uniformis |
| 40098 | 78_3869 | 47 | 14 | Bacteroidetes | Bacteroidia | Bacteroidales | Bacteroidaceae | Bacteroides | Bacteroides intestinalis |
| 40106 | 122_1515 | 34 | 8 | Bacteroidetes | Bacteroidia | Bacteroidales | Bacteroidaceae | Bacteroides | Bacteroides massiliensis |
| 40157 | 20_284 | 11 | 5 | Bacteroidetes | Bacteroidia | Bacteroidales | Bacteroidaceae | Bacteroides | Bacteroides intestinalis |
| 40170 | 50_3006 | 16 | 10 | Bacteroidetes | Bacteroidia | Bacteroidales | Porphyromonadaceae | Parabacteroides | Parabacteroides distasonis |
| 40216 | 100_6946 | 7 | 5 | Bacteroidetes | Bacteroidia | Bacteroidales | Porphyromonadaceae | Parabacteroides | Parabacteroides distasonis |
| 40229 | 17_876 | 5 | 5 | Bacteroidetes | Bacteroidia | Bacteroidales | Rikenellaceae | Alistipes | Alistipes sp. |
| 40292 | 69_51 | 7 | 5 | Bacteroidetes | Bacteroidia | Bacteroidales | Porphyromonadaceae | Parabacteroides | Parabacteroides distasonis |
| 40305 | 24_3879 | 6 | 5 | Bacteroidetes | Bacteroidia | Bacteroidales | Bacteroidaceae | Bacteroides | Bacteroides vulgatus |
| 40306 | 129_4409 | 11 | 7 | Bacteroidetes | Bacteroidia | Bacteroidales | Bacteroidaceae | Bacteroides | Bacteroides vulgatus |
| 40317 | 65_5804 | 8 | 5 | Bacteroidetes | Bacteroidia | Bacteroidales | Bacteroidaceae | Bacteroides | Bacteroides vulgatus |
| 40319 | 12_526 | 34 | 10 | Bacteroidetes | Bacteroidia | Bacteroidales | Bacteroidaceae | Bacteroides | Bacteroides vulgatus |
| 40343 | 51_2529 | 11 | 6 | Bacteroidetes | Bacteroidia | Bacteroidales | Porphyromonadaceae | Parabacteroides | Parabacteroides distasonis |
| 40347 | 29_2510 | 7 | 7 | Bacteroidetes | Bacteroidia | Bacteroidales | Porphyromonadaceae | Parabacteroides | Parabacteroides distasonis |
| 40366 | 123_7653 | 145 | 19 | Bacteroidetes | Bacteroidia | Bacteroidales | Bacteroidaceae | Bacteroides | Bacteroides vulgatus |
| 40401 | 84_2814 | 55 | 17 | Bacteroidetes | Bacteroidia | Bacteroidales | Porphyromonadaceae | Parabacteroides | Parabacteroides distasonis |
| 40424 | 2_6979 | 888 | 12 | Bacteroidetes | Bacteroidia | Bacteroidales | Bacteroidaceae | Bacteroides | Bacteroides dorei |
| 40449 | 58_2440 | 39 | 3 | Bacteroidetes | Bacteroidia | Bacteroidales | Bacteroidaceae | Bacteroides | Bacteroides plebeius |
| 40452 | 58_7 | 42 | 2 | Bacteroidetes | Bacteroidia | Bacteroidales | Bacteroidaceae | Bacteroides | Bacteroides plebeius |
| 40453 | 40_2330 | 29 | 14 | Bacteroidetes | Bacteroidia | Bacteroidales | Rikenellaceae | Alistipes | Alistipes sp. |
| 40456 | 67_7794 | 8631 | 42 | Bacteroidetes | Bacteroidia | Bacteroidales | Bacteroidaceae | Bacteroides | Bacteroides dorei |
| 40469 | 55_2327 | 902 | 29 | Bacteroidetes | Bacteroidia | Bacteroidales | Bacteroidaceae | Bacteroides | Bacteroides dorei |
| 40474 | 67_8992 | 34 | 6 | Bacteroidetes | Bacteroidia | Bacteroidales | Bacteroidaceae | Bacteroides | Bacteroides dorei |
| 40475 | 55_5020 | 8 | 5 | Bacteroidetes | Bacteroidia | Bacteroidales | Bacteroidaceae | Bacteroides | Bacteroides dorei |
| 40484 | 113_2433 | 28 | 7 | Bacteroidetes | Bacteroidia | Bacteroidales | Bacteroidaceae | Bacteroides | Bacteroides intestinalis |
| 40532 | 24_6979 | 7 | 7 | Bacteroidetes | Bacteroidia | Bacteroidales | Porphyromonadaceae | Parabacteroides | Parabacteroides distasonis |
| 40547 | 65_6703 | 57 | 16 | Bacteroidetes | Bacteroidia | Bacteroidales | Porphyromonadaceae | Parabacteroides | Parabacteroides distasonis |
| 40559 | 114_3554 | 13 | 10 | Bacteroidetes | Bacteroidia | Bacteroidales | Bacteroidaceae | Bacteroides | Bacteroides vulgatus |
| 40560 | 41_8740 | 21 | 5 | Proteobacteria | Gammaproteobacteria | Enterobacteriales | Enterobacteriaceae | Escherichia/Shigella | Escherichia/Shigella |
| 40567 | 53_2434 | 45 | 8 | Bacteroidetes | Bacteroidia | Bacteroidales | Porphyromonadaceae | Parabacteroides | Parabacteroides distasonis |
| 40572 | 29_2013 | 22 | 8 | Bacteroidetes | Bacteroidia | Bacteroidales | Bacteroidaceae | Bacteroides | Bacteroides intestinalis |
| 40578 | 123_2953 | 210 | 31 | Bacteroidetes | Bacteroidia | Bacteroidales | Bacteroidaceae | Bacteroides | Bacteroides vulgatus |
| 40584 | 17_3140 | 38 | 13 | Bacteroidetes | Bacteroidia | Bacteroidales | Bacteroidaceae | Bacteroides | Bacteroides dorei |
| 40587 | 42_2341 | 104 | 32 | Bacteroidetes | Bacteroidia | Bacteroidales | Bacteroidaceae | Bacteroides | Bacteroides vulgatus |
| 40590 | 128_2633 | 10 | 8 | Bacteroidetes | Bacteroidia | Bacteroidales | Bacteroidaceae | Bacteroides | Bacteroides vulgatus |
| 40603 | 33_5643 | 8 | 7 | Bacteroidetes | Bacteroidia | Bacteroidales | Porphyromonadaceae | Odoribacter | Odoribacter splanchnicus |
| 40630 | 63_272 | 6 | 5 | Bacteroidetes | Bacteroidia | Bacteroidales | Bacteroidaceae | Bacteroides | Bacteroides eggerthii |
| 40642 | 68_6832 | 11 | 7 | Bacteroidetes | Bacteroidia | Bacteroidales | Bacteroidaceae | Bacteroides | Bacteroides vulgatus |
| 40672 | 108_6745 | 15 | 6 | Bacteroidetes | Bacteroidia | Bacteroidales | Prevotellaceae | Prevotella | Prevotella stercorea |
| 40678 | 42_3560 | 9 | 5 | Bacteroidetes | Bacteroidia | Bacteroidales | Porphyromonadaceae | Parabacteroides | Parabacteroides distasonis |
| 40680 | 30_9803 | 19 | 6 | Bacteroidetes | Bacteroidia | Bacteroidales | Bacteroidaceae | Bacteroides | Bacteroides vulgatus |
| 40688 | 65_3458 | 78 | 18 | Bacteroidetes | Bacteroidia | Bacteroidales | Bacteroidaceae | Bacteroides | Bacteroides vulgatus |
| 40702 | 12_1162 | 8 | 7 | Bacteroidetes | Bacteroidia | Bacteroidales | Bacteroidaceae | Bacteroides | Bacteroides vulgatus |
| 40711 | 130_6242 | 28 | 17 | Bacteroidetes | Bacteroidia | Bacteroidales | Bacteroidaceae | Bacteroides | Bacteroides vulgatus |
| 40713 | 108_6843 | 40 | 15 | Bacteroidetes | Bacteroidia | Bacteroidales | Bacteroidaceae | Bacteroides | Bacteroides vulgatus |
| 40720 | 20_8386 | 222 | 12 | Bacteroidetes | Bacteroidia | Bacteroidales | Bacteroidaceae | Bacteroides | Bacteroides vulgatus |
| 40739 | 7_4986 | 14 | 13 | Bacteroidetes | Bacteroidia | Bacteroidales | Bacteroidaceae | Bacteroides | Bacteroides vulgatus |
| 40759 | 108_7063 | 6 | 5 | Bacteroidetes | Bacteroidia | Bacteroidales | Rikenellaceae | Alistipes | Alistipes sp. |
| 40814 | 9_5225 | 26 | 7 | Bacteroidetes | Bacteroidia | Bacteroidales | Bacteroidaceae | Bacteroides | Bacteroides vulgatus |
| 40890 | 81_3152 | 18 | 5 | Bacteroidetes | Bacteroidia | Bacteroidales | Prevotellaceae | Prevotella | Prevotella copri |
| 40893 | 112_642 | 15 | 9 | Bacteroidetes | Bacteroidia | Bacteroidales | Prevotellaceae | Prevotella | Prevotella sp. |
| 40909 | 112_5246 | 54 | 11 | Bacteroidetes | Bacteroidia | Bacteroidales | Prevotellaceae | Prevotella | Prevotella stercorea |
| 40915 | 1_3226 | 9 | 6 | Bacteroidetes | Bacteroidia | Bacteroidales | Prevotellaceae | Prevotella | Prevotella copri |
| 40925 | 7_1398 | 37 | 6 | Bacteroidetes | Bacteroidia | Bacteroidales | Prevotellaceae | Prevotella | Prevotella stercorea |
| 40926 | 49_2704 | 713 | 16 | Bacteroidetes | Bacteroidia | Bacteroidales | Prevotellaceae | Prevotella | Prevotella stercorea |
| 40946 | 49_3589 | 22 | 9 | Bacteroidetes | Bacteroidia | Bacteroidales | Bacteroidaceae | Bacteroides | Bacteroides sp. |
| 40959 | 7_4704 | 14 | 6 | Bacteroidetes | Bacteroidia | Bacteroidales | Prevotellaceae | Prevotella | Prevotella stercorea |
| 40961 | 25_79 | 5 | 5 | Bacteroidetes | Bacteroidia | Bacteroidales | Prevotellaceae | Prevotella | Prevotella stercorea |
| 40966 | 106_6511 | 1080 | 18 | Bacteroidetes | Bacteroidia | Bacteroidales | Prevotellaceae | Prevotella | Prevotella stercorea |
| 40968 | 49_3505 | 13 | 8 | Bacteroidetes | Bacteroidia | Bacteroidales | Prevotellaceae | Prevotella | Prevotella stercorea |
| 40974 | 7_2863 | 44 | 11 | Bacteroidetes | Bacteroidia | Bacteroidales | Prevotellaceae | Prevotella | Prevotella stercorea |
| 40975 | 106_1159 | 8 | 6 | Bacteroidetes | Bacteroidia | Bacteroidales | Bacteroidaceae | Bacteroides | Bacteroides sp. |
| 41006 | 129_4933 | 40 | 3 | Bacteroidetes | Bacteroidia | Bacteroidales | Prevotellaceae | Prevotella | Prevotella stercorea |
| 41021 | 129_2192 | 21 | 4 | Bacteroidetes | Bacteroidia | Bacteroidales | Prevotellaceae | Prevotella | Prevotella stercorea |
| 41040 | 125_3192 | 22 | 3 | Bacteroidetes | Bacteroidia | Bacteroidales | Prevotellaceae | Prevotella | Prevotella stercorea |
| 41068 | 123_6642 | 11 | 6 | Bacteroidetes | Bacteroidia | Bacteroidales | Prevotellaceae | Prevotella | Prevotella copri |
| 41099 | 112_4480 | 41 | 7 | Bacteroidetes | Bacteroidia | Bacteroidales | Prevotellaceae | Prevotella | Prevotella stercorea |
| 41128 | 112_3586 | 13 | 7 | Bacteroidetes | Bacteroidia | Bacteroidales | Prevotellaceae | Prevotella | Prevotella copri |
| 41137 | 23_1846 | 28 | 7 | Bacteroidetes | Bacteroidia | Bacteroidales | Prevotellaceae | Prevotella | Prevotella copri |
| 41142 | 123_2931 | 10 | 5 | Bacteroidetes | Bacteroidia | Bacteroidales | Prevotellaceae | Prevotella | Prevotella copri |
| 41144 | 21_7205 | 10 | 5 | Bacteroidetes | Bacteroidia | Bacteroidales | Prevotellaceae | Prevotella | Prevotella copri |
| 41174 | 115_2505 | 8 | 7 | Bacteroidetes | Bacteroidia | Bacteroidales | Prevotellaceae | Prevotella | Prevotella copri |
| 41178 | 115_2418 | 10 | 9 | Bacteroidetes | Bacteroidia | Bacteroidales | Porphyromonadaceae | Parabacteroides | Parabacteroides distasonis |
| 41181 | 123_4468 | 19 | 7 | Bacteroidetes | Bacteroidia | Bacteroidales | Bacteroidaceae | Bacteroides | Bacteroides uniformis |
| 41183 | 66_3254 | 6 | 5 | Bacteroidetes | Bacteroidia | Bacteroidales | Bacteroidaceae | Bacteroides | Bacteroides finegoldii |
| 41192 | 33_31 | 11 | 5 | Bacteroidetes | Bacteroidia | Bacteroidales | Prevotellaceae | Prevotella | Prevotella copri |
| 41199 | 123_7216 | 13 | 6 | Bacteroidetes | Bacteroidia | Bacteroidales | Prevotellaceae | Prevotella | Prevotella copri |
| 41205 | 125_2519 | 26 | 8 | Bacteroidetes | Bacteroidia | Bacteroidales | Prevotellaceae | Prevotella | Prevotella copri |
| 41207 | 123_2249 | 8 | 6 | Bacteroidetes | Bacteroidia | Bacteroidales | Prevotellaceae | Prevotella | Prevotella copri |
| 41222 | 115_402 | 16 | 6 | Bacteroidetes | Bacteroidia | Bacteroidales | Prevotellaceae | Prevotella | Prevotella copri |
| 41232 | 26_1421 | 11 | 8 | Bacteroidetes | Bacteroidia | Bacteroidales | Rikenellaceae | Alistipes | Alistipes sp. |
| 41245 | 17_3180 | 9 | 6 | Bacteroidetes | Bacteroidia | Bacteroidales | Rikenellaceae | Alistipes | Alistipes sp. |
| 41256 | 123_3823 | 84 | 21 | Bacteroidetes | Bacteroidia | Bacteroidales | Prevotellaceae | Prevotella | Prevotella copri |
| 41277 | 81_6261 | 52 | 7 | Bacteroidetes | Bacteroidia | Bacteroidales | Bacteroidaceae | Bacteroides | Bacteroides plebeius |
| 41304 | 104_1519 | 32 | 6 | Bacteroidetes | Bacteroidia | Bacteroidales | Prevotellaceae | Prevotella | Prevotella copri |
| 41317 | 69_178 | 10 | 7 | Bacteroidetes | Bacteroidia | Bacteroidales | Bacteroidaceae | Bacteroides | Bacteroides salyersiae |
| 41319 | 4_214 | 23 | 11 | Bacteroidetes | Bacteroidia | Bacteroidales | Bacteroidaceae | Bacteroides | Bacteroides massiliensis |
| 41327 | 21_6647 | 18 | 5 | Bacteroidetes | Bacteroidia | Bacteroidales | Prevotellaceae | Prevotella | Prevotella copri |
| 41330 | 21_7297 | 11 | 6 | Bacteroidetes | Bacteroidia | Bacteroidales | Prevotellaceae | Prevotella | Prevotella copri |
| 41340 | 104_714 | 5 | 5 | Bacteroidetes | Bacteroidia | Bacteroidales | Prevotellaceae | Prevotella | Prevotella copri |
| 41505 | 124_6677 | 22 | 2 | Firmicutes | Clostridia | Clostridiales | Peptococcaceae | Peptococcus | Peptococcus sp. |
| 41518 | 111_240 | 36 | 9 | Bacteroidetes | Bacteroidia | Bacteroidales | Bacteroidaceae | Bacteroides | Bacteroides uniformis |
| 41570 | 65_1532 | 12 | 7 | Bacteroidetes | Bacteroidia | Bacteroidales | Bacteroidaceae | Bacteroides | Bacteroides vulgatus |
| 41574 | 65_3589 | 16 | 9 | Bacteroidetes | Bacteroidia | Bacteroidales | Bacteroidaceae | Bacteroides | Bacteroides vulgatus |
| 41603 | 123_12 | 5 | 5 | Bacteroidetes | Bacteroidia | Bacteroidales | Prevotellaceae | Prevotella | Prevotella copri |
| 41643 | 66_2662 | 19 | 5 | Bacteroidetes | Bacteroidia | Bacteroidales | Prevotellaceae | Prevotella | Prevotella copri |
| 41677 | 102_4112 | 59 | 16 | Bacteroidetes | Bacteroidia | Bacteroidales | Bacteroidaceae | Bacteroides | Bacteroides vulgatus |
| 41683 | 115_2144 | 54 | 16 | Firmicutes | Erysipelotrichia | Erysipelotrichales | Erysipelotrichaceae | Catenibacterium | Catenibacterium mitsuokai |
| 41687 | 101_4303 | 579 | 17 | Bacteroidetes | Bacteroidia | Bacteroidales | Bacteroidaceae | Bacteroides | Bacteroides intestinalis |
| 41715 | 115_1485 | 15 | 11 | Bacteroidetes | Bacteroidia | Bacteroidales | Rikenellaceae | Alistipes | Alistipes sp. |
| 41746 | 47_2050 | 11 | 5 | Proteobacteria | Gammaproteobacteria | Enterobacteriales | Enterobacteriaceae | Escherichia/Shigella | Escherichia/Shigella |
| 41747 | 67_3953 | 71 | 6 | Proteobacteria | Gammaproteobacteria | Enterobacteriales | Enterobacteriaceae | Escherichia/Shigella | Escherichia/Shigella |
| 41756 | 41_9867 | 141 | 48 | Firmicutes | Clostridia | Clostridiales | Lachnospiraceae | Blautia | Blautia |
| 41759 | 65_4510 | 89 | 27 | Bacteroidetes | Bacteroidia | Bacteroidales | Bacteroidaceae | Bacteroides | Bacteroides vulgatus |
| 41767 | 7_1596 | 16 | 6 | Bacteroidetes | Bacteroidia | Bacteroidales | Prevotellaceae | Prevotella | Prevotella stercorea |
| 41769 | 27_747 | 59 | 1 | Bacteroidetes | Bacteroidia | Bacteroidales | Rikenellaceae | Alistipes | Alistipes onderdonkii |
| 41773 | 79_6481 | 31 | 10 | Bacteroidetes | Bacteroidia | Bacteroidales | Bacteroidaceae | Bacteroides | Bacteroides vulgatus |
| 41809 | 123_3769 | 555 | 78 | Firmicutes | Clostridia | Clostridiales | Lachnospiraceae | Blautia | Blautia |
| 41812 | 21_4016 | 16 | 6 | Firmicutes | Clostridia | Clostridiales | Lachnospiraceae | Lachnospiraceae incertae sedis | Lachnospiraceae incertae sedis |
| 41826 | 117_4901 | 50 | 24 | Firmicutes | Clostridia | Clostridiales | Lachnospiraceae | Roseburia | Roseburia intestinalis |
| 41829 | 42_6846 | 19 | 12 | Firmicutes | Clostridia | Clostridiales | Lachnospiraceae | Roseburia | Roseburia intestinalis |
| 41834 | 58_8249 | 10 | 5 | Firmicutes | Clostridia | Clostridiales | Lachnospiraceae | Roseburia | Roseburia inulinivorans |
| 41842 | 65_5073 | 83 | 36 | Firmicutes | Clostridia | Clostridiales | Lachnospiraceae | Roseburia | Roseburia intestinalis |
| 41843 | 32_3256 | 11 | 8 | Firmicutes | Clostridia | Clostridiales | Lachnospiraceae | Blautia | Blautia |
| 41886 | 49_7 | 11 | 6 | Firmicutes | Negativicutes | Selenomonadales | Veillonellaceae | Dialister | Dialister invisus |
| 41897 | 26_2238 | 33 | 19 | Firmicutes | Clostridia | Clostridiales | Ruminococcaceae | Papillibacter | Papillibacter cinnamivorans |
| 41923 | 5_1425 | 14 | 6 | Firmicutes | Erysipelotrichia | Erysipelotrichales | Erysipelotrichaceae | Catenibacterium | Catenibacterium mitsuokai |
| 41941 | 1_1379 | 14 | 9 | Firmicutes | Erysipelotrichia | Erysipelotrichales | Erysipelotrichaceae | Catenibacterium | Catenibacterium mitsuokai |
| 41950 | 1_378 | 6 | 6 | Proteobacteria | Betaproteobacteria | Burkholderiales | Sutterellaceae | Sutterella | Sutterella stercoricanis |
| 41953 | 1_2638 | 14 | 6 | Firmicutes | Erysipelotrichia | Erysipelotrichales | Erysipelotrichaceae | Catenibacterium | Catenibacterium mitsuokai |
| 41955 | 1_3450 | 26 | 7 | Firmicutes | Erysipelotrichia | Erysipelotrichales | Erysipelotrichaceae | Catenibacterium | Catenibacterium mitsuokai |
| 41976 | 1_1011 | 14 | 8 | Firmicutes | Negativicutes | Selenomonadales | Veillonellaceae | Megasphaera | Megasphaera elsdenii |
| 41987 | 1_2207 | 88 | 5 | Firmicutes | Negativicutes | Selenomonadales | Veillonellaceae | Megasphaera | Megasphaera elsdenii |
| 42067 | 53_2846 | 7 | 7 | Firmicutes | Clostridia | Clostridiales | Lachnospiraceae | Blautia | Blautia |
| 42092 | 112_3529 | 37 | 6 | Firmicutes | Negativicutes | Selenomonadales | Veillonellaceae | Dialister | Dialister invisus |
| 42103 | 128_1819 | 36 | 11 | Bacteroidetes | Bacteroidia | Bacteroidales | Prevotellaceae | Prevotella | Prevotella copri |
| 42122 | 18_2715 | 7 | 5 | Actinobacteria | Coriobacteriia | Coriobacteriales | Coriobacteriaceae | Collinsella | Collinsella aerofaciens |
| 42278 | 8_515 | 22 | 5 | Firmicutes | Clostridia | Clostridiales | Ruminococcaceae | Faecalibacterium | Faecalibacterium prausnitzii |
| 42288 | 124_3352 | 454 | 30 | NULL | NULL | NULL | NULL | NULL | NULL |
| 42298 | 73_182 | 27 | 12 | NULL | NULL | NULL | NULL | NULL | NULL |
| 42323 | 31_5247 | 5 | 5 | Bacteroidetes | Bacteroidia | Bacteroidales | Prevotellaceae | Prevotella | Prevotella copri |
| 42342 | 99_8162 | 21 | 10 | Firmicutes | Erysipelotrichia | Erysipelotrichales | Erysipelotrichaceae | Erysipelotrichaceae incertae sedis | Erysipelotrichaceae incertae sedis |
| 42344 | 124_7173 | 6 | 5 | Firmicutes | Clostridia | Clostridiales | Oscillospiraceae | Oscillibacter | Oscillibacter |
| 42354 | 69_2305 | 25 | 7 | Firmicutes | Erysipelotrichia | Erysipelotrichales | Erysipelotrichaceae | Erysipelotrichaceae incertae sedis | Erysipelotrichaceae incertae sedis |
| 42361 | 67_2590 | 15 | 5 | Firmicutes | Erysipelotrichia | Erysipelotrichales | Erysipelotrichaceae | Erysipelotrichaceae incertae sedis | Erysipelotrichaceae incertae sedis |
| 42362 | 13_3119 | 9 | 9 | Firmicutes | Clostridia | Clostridiales | Oscillospiraceae | Oscillibacter | Oscillibacter |
| 42365 | 96_9489 | 18 | 5 | Firmicutes | Erysipelotrichia | Erysipelotrichales | Erysipelotrichaceae | Erysipelotrichaceae incertae sedis | Erysipelotrichaceae incertae sedis |
| 42372 | 6_4412 | 29 | 16 | Firmicutes | Erysipelotrichia | Erysipelotrichales | Erysipelotrichaceae | Erysipelotrichaceae incertae sedis | Erysipelotrichaceae incertae sedis |
| 42377 | 48_186 | 37 | 17 | Firmicutes | Clostridia | Clostridiales | Ruminococcaceae | Papillibacter | Papillibacter cinnamivorans |
| 42378 | 26_2623 | 12 | 8 | Firmicutes | Clostridia | Clostridiales | Ruminococcaceae | Papillibacter | Papillibacter cinnamivorans |
| 42401 | 47_5475 | 8 | 8 | Firmicutes | Clostridia | Clostridiales | Lachnospiraceae | Blautia | Blautia |
| 42428 | 66_1774 | 15 | 9 | Firmicutes | Erysipelotrichia | Erysipelotrichales | Erysipelotrichaceae | Holdemanella | Eubacterium biforme |
| 42461 | 12_4944 | 384 | 67 | Firmicutes | Bacilli | Lactobacillales | Streptococcaceae | Streptococcus | Streptococcus salivarius |
| 42499 | 32_5260 | 9 | 7 | Firmicutes | Clostridia | Clostridiales | Ruminococcaceae | Faecalibacterium | Faecalibacterium prausnitzii |
| 42503 | 42_4769 | 234 | 27 | Firmicutes | Clostridia | Clostridiales | Ruminococcaceae | Faecalibacterium | Faecalibacterium prausnitzii |
| 42509 | 32_1242 | 54 | 7 | Firmicutes | Clostridia | Clostridiales | Ruminococcaceae | Faecalibacterium | Faecalibacterium prausnitzii |
| 42545 | 12_4386 | 7 | 7 | Firmicutes | Clostridia | Clostridiales | Lachnospiraceae | Roseburia | Roseburia intestinalis |
| 42572 | 53_5803 | 12 | 10 | Firmicutes | Clostridia | Clostridiales | Lachnospiraceae | Roseburia | Eubacterium rectale |
| 42578 | 86_1523 | 30 | 8 | Firmicutes | Clostridia | Clostridiales | Clostridiales Family XIII | Clostridiales Family XIII incertae sedis | Clostridiales Family XIII incertae sedis |
| 42605 | 130_7421 | 2728 | 88 | Firmicutes | Clostridia | Clostridiales | Lachnospiraceae | Lachnospiraceae incertae sedis | Lachnospiraceae incertae sedis |
| 42606 | 119_3699 | 6 | 6 | Firmicutes | Clostridia | Clostridiales | Lachnospiraceae | Lachnospiraceae incertae sedis | Lachnospiraceae incertae sedis |
| 42625 | 55_6331 | 32 | 22 | Firmicutes | Clostridia | Clostridiales | Lachnospiraceae | Blautia | Blautia |
| 42636 | 89_2625 | 59 | 11 | Bacteroidetes | Bacteroidia | Bacteroidales | Bacteroidaceae | Bacteroides | Bacteroides ovatus |
| 42651 | 71_5362 | 62 | 22 | Firmicutes | Clostridia | Clostridiales | Lachnospiraceae | Lachnospiraceae incertae sedis | Lachnospiraceae incertae sedis |
| 42660 | 97_1983 | 37 | 21 | Firmicutes | Clostridia | Clostridiales | Lachnospiraceae | Lachnospiraceae incertae sedis | Lachnospiraceae incertae sedis |
| 42685 | 127_2264 | 11 | 8 | Firmicutes | Clostridia | Clostridiales | Lachnospiraceae | Roseburia | Eubacterium rectale |
| 42691 | 70_3232 | 8 | 5 | Firmicutes | Clostridia | Clostridiales | Lachnospiraceae | Dorea | Dorea formicigenerans |
| 42694 | 91_10145 | 14 | 9 | Bacteroidetes | Bacteroidia | Bacteroidales | Bacteroidaceae | Bacteroides | Bacteroides ovatus |
| 42700 | 26_3578 | 382 | 59 | Firmicutes | Clostridia | Clostridiales | Lachnospiraceae | Dorea | Dorea formicigenerans |
| 42711 | 11_2060 | 6 | 5 | Firmicutes | Clostridia | Clostridiales | Lachnospiraceae | Lachnospiraceae incertae sedis | Lachnospiraceae incertae sedis |
| 42712 | 124_7204 | 55 | 36 | Firmicutes | Clostridia | Clostridiales | Lachnospiraceae | Lachnospiraceae incertae sedis | Lachnospiraceae incertae sedis |
| 42740 | 42_3293 | 36 | 21 | Firmicutes | Clostridia | Clostridiales | Lachnospiraceae | Blautia | Blautia |
| 42743 | 98_2248 | 132 | 38 | Firmicutes | Clostridia | Clostridiales | Lachnospiraceae | Blautia | Blautia |
| 42762 | 26_4816 | 10 | 6 | NULL | NULL | NULL | NULL | NULL | NULL |
| 42769 | 17_2034 | 56 | 11 | NULL | NULL | NULL | NULL | NULL | NULL |
| 42827 | 65_7964 | 27 | 15 | Firmicutes | Clostridia | Clostridiales | Eubacteriaceae | Eubacterium | Eubacterium desmolans |
| 42829 | 55_6118 | 27 | 13 | Firmicutes | Clostridia | Clostridiales | Eubacteriaceae | Eubacterium | Eubacterium desmolans |
| 42831 | 12_2190 | 24 | 10 | Firmicutes | Clostridia | Clostridiales | Eubacteriaceae | Eubacterium | Eubacterium desmolans |
| 42832 | 10_6531 | 55 | 29 | Firmicutes | Clostridia | Clostridiales | Eubacteriaceae | Eubacterium | Eubacterium desmolans |
| 42833 | 9_4760 | 7 | 5 | Firmicutes | Clostridia | Clostridiales | Eubacteriaceae | Eubacterium | Eubacterium desmolans |
| 42834 | 127_1386 | 7 | 5 | Firmicutes | Clostridia | Clostridiales | Eubacteriaceae | Eubacterium | Eubacterium desmolans |
| 42837 | 44_3222 | 5 | 5 | Firmicutes | Clostridia | Clostridiales | Clostridiaceae | Clostridium | Clostridium sp. |
| 42839 | 127_97 | 34 | 17 | Firmicutes | Clostridia | Clostridiales | Eubacteriaceae | Eubacterium | Eubacterium desmolans |
| 42855 | 115_3896 | 13 | 6 | Firmicutes | Clostridia | Clostridiales | Lachnospiraceae | Blautia | Blautia |
| 42911 | 129_1328 | 124 | 25 | Bacteroidetes | Bacteroidia | Bacteroidales | Bacteroidaceae | Bacteroides | Bacteroides vulgatus |
| 42914 | 123_7134 | 8 | 6 | Bacteroidetes | Bacteroidia | Bacteroidales | Porphyromonadaceae | Odoribacter | Odoribacter splanchnicus |
| 42935 | 41_8909 | 20 | 4 | Bacteroidetes | Bacteroidia | Bacteroidales | Bacteroidaceae | Bacteroides | Bacteroides massiliensis |
| 42951 | 52_3841 | 16 | 10 | Proteobacteria | Gammaproteobacteria | Enterobacteriales | Enterobacteriaceae | Escherichia/Shigella | Escherichia/Shigella |
| 42952 | 47_5865 | 134 | 12 | Proteobacteria | Gammaproteobacteria | Enterobacteriales | Enterobacteriaceae | Escherichia/Shigella | Escherichia/Shigella |
| 42956 | 91_10911 | 24 | 7 | Proteobacteria | Gammaproteobacteria | Enterobacteriales | Enterobacteriaceae | Escherichia/Shigella | Escherichia/Shigella |
| 42957 | 47_4512 | 26 | 6 | Proteobacteria | Gammaproteobacteria | Enterobacteriales | Enterobacteriaceae | Escherichia/Shigella | Escherichia/Shigella |
| 42961 | 55_3177 | 213 | 18 | Proteobacteria | Gammaproteobacteria | Enterobacteriales | Enterobacteriaceae | Escherichia/Shigella | Escherichia/Shigella |
| 43060 | 49_2733 | 16 | 7 | Bacteroidetes | Bacteroidia | Bacteroidales | Porphyromonadaceae | Parabacteroides | Parabacteroides merdae |
| 43064 | 60_431 | 20 | 4 | Bacteroidetes | Bacteroidia | Bacteroidales | Bacteroidaceae | Bacteroides | Bacteroides massiliensis |
| 43135 | 22_1338 | 13 | 9 | Bacteroidetes | Bacteroidia | Bacteroidales | Porphyromonadaceae | Parabacteroides | Parabacteroides merdae |
| 43146 | 69_345 | 6 | 5 | Bacteroidetes | Bacteroidia | Bacteroidales | Porphyromonadaceae | Parabacteroides | Parabacteroides merdae |
| 43162 | 63_2015 | 12 | 9 | Bacteroidetes | Bacteroidia | Bacteroidales | Bacteroidaceae | Bacteroides | Bacteroides vulgatus |
| 43167 | 67_2148 | 18 | 8 | Bacteroidetes | Bacteroidia | Bacteroidales | Bacteroidaceae | Bacteroides | Bacteroides intestinalis |
| 43184 | 113_2301 | 12 | 6 | Bacteroidetes | Bacteroidia | Bacteroidales | Bacteroidaceae | Bacteroides | Bacteroides intestinalis |
| 43190 | 38_2022 | 9 | 7 | Bacteroidetes | Bacteroidia | Bacteroidales | Porphyromonadaceae | Odoribacter | Odoribacter splanchnicus |
| 43210 | 58_2932 | 30 | 1 | Bacteroidetes | Bacteroidia | Bacteroidales | Bacteroidaceae | Bacteroides | Bacteroides vulgatus |
| 43215 | 58_4948 | 38 | 2 | Bacteroidetes | Bacteroidia | Bacteroidales | Bacteroidaceae | Bacteroides | Bacteroides vulgatus |
| 43272 | 122_3585 | 18 | 7 | Bacteroidetes | Bacteroidia | Bacteroidales | Bacteroidaceae | Bacteroides | Bacteroides intestinalis |
| 43277 | 67_4561 | 95 | 9 | Bacteroidetes | Bacteroidia | Bacteroidales | Bacteroidaceae | Bacteroides | Bacteroides intestinalis |
| 43282 | 122_190 | 19 | 6 | Bacteroidetes | Bacteroidia | Bacteroidales | Bacteroidaceae | Bacteroides | Bacteroides intestinalis |
[truncated: 434,958 more chars]
