# Supplementary material for: Individual-specific changes in the human gut microbiota after challenge with enterotoxigenic Escherichia coli and subsequent ciprofloxacin treatment
Source: BMC Genomics. 2016 Jun 8;17:440. doi: 10.1186/s12864-016-2777-0 (PMC4898365; doi:10.1186/s12864-016-2777-0)
Supplement: Additional file 5: Table S1. — Quantitative measurements of E. coli presence in the samples (quantitative culture, qPCR, 16S rRNA gene). (DOCX 27 kb) [file 12864_2016_2777_MOESM5_ESM.docx]

Table s1

| SampleID | SubjectID | Weight | DAY | Fraction5Col | TotalEcoli | QualCulture | QuantCulture | Log10QuantCulture | LTQuantityPCR | Log10QPCR | Log1016S | Diarrhea | AnyDayDiarrhea | NextDayDiarrhea |
| --- | --- | --- | --- | --- | --- | --- | --- | --- | --- | --- | --- | --- | --- | --- |
| 31 | P03 | 0.31 | 0 | 0 | 20000 | 0 | 0 | 0 | 409.05 | 2.611776397 | 0 | 0 | 0 | 0 |
| 52 | P03 | 0.41 | 1 | 0 | 30000 | 0 | 0 | 0 | 23.5938864 | 1.372799484 | 0 | 0 | 0 | 0 |
| 80 | P03 | 0.32 | 2 |  | 0 | 0 | 0 | 0 | 2.694729169 | 0.430515123 | 0.365523 | 0 | 0 | 0 |
| 109 | P03 | 0.29 | 3 |  | 0 | 0 | 0 | 0 | 1.4881156 | 0.172636669 | 0 | 0 | 0 | 0 |
| 137 | P03 | 0.31 | 4 |  | 0 | 0 | 0 | 0 | 0 | 0 | 0 | 0 | 0 | NA |
| 32 | P04 | 0.26 | 0 | 0 | 1500000 | 0 | 0 | 0 | 0 | 0 | 0 | 0 | 1 | 0 |
| 53 | P04 | 0.78 | 1 | 0 | 12000000 | 0 | 0 | 0 | 0 | 0 | 0.279841 | 0 | 1 | 0 |
| 81 | P04 | 0.25 | 2 | 1 | 8400000 | 1 | 8400000 | 6.924279286 | 21910.3 | 4.340648324 | 0.783834 | 0 | 1 | 1 |
| 110 | P04 | 0.23 | 3 | 1 | 19000000 | 1 | 19000000 | 7.278753601 | 28752.41882 | 4.458674386 | 0.905412 | 1 | 1 | 1 |
| 138 | P04 | 0.57 | 4 | 1 | 1E+09 | 1 | 1000000000 | 9 | 57208139.03 | 7.75745782 | 3.603 | 1 | 1 | NA |
| 36 | P11 | 0.32 | 0 | 0 | 330000 | 0 | 0 | 0 | 0 | 0 | 0.742321 | 0 | 1 | 0 |
| 58 | P11 | 0.26 | 1 | 0.8 | 10000000 | 1 | 8100000 | 6.908485019 | 20912.10442 | 4.320397739 | 0.7687 | 0 | 1 | 1 |
| 86 | P11 | 0.23 | 2 | 0.8 | 120000000 | 1 | 95000000 | 7.977723605 | 2300797.124 | 6.361878326 | 1.97994 | 1 | 1 | 1 |
| 115 | P11 | 0.98 | 3 | 0.8 | 960000000 | 1 | 760000000 | 8.880813592 | 618125334.1 | 8.791076544 | 3.560527 | 1 | 1 | 1 |
| 37 | P13 | 0.27 | 0 |  | 0 | 0 | 0 | 0 | 337.55 | 2.528338112 | 0.630784 | 0 | 0 | 0 |
| 60 | P13 | 0.37 | 1 |  | 0 | 0 | 0 | 0 | 0 | 0 | 0 | 0 | 0 | 0 |
| 88 | P13 | 0.29 | 2 |  | 0 | 0 | 0 | 0 | 0 | 0 | NA | 0 | 0 | 0 |
| 117 | P13 | 0.32 | 3 |  | 0 | 0 | 0 | 0 | 4944157.259 | 6.694092276 | NA | 0 | 0 | 0 |
| 145 | P13 | 0.23 | 4 |  | 0 | 0 | 0 | 0 | 0 | 0 | 0 | 0 | 0 | NA |
| 62 | P16 |  | 1 |  |  |  |  | NA | 0 | 0 | 0 | 0 | 0 | 1 |
| 90 | P16 |  | 2 |  |  | 1 | 1200000000 | 9.079181246 | 4275838.007 | 6.631021243 | 3.292597 | 1 | 1 | 1 |
| 119 | P16 |  | 3 |  |  |  |  | NA | 338252229 | 8.529240667 | 4.443979 | 1 | 1 | 1 |
| 39 | P17 | 0.29 | 0 |  | 0 | 0 | 0 | 0 | 0 | 0 | 0 | 0 | 1 | 0 |
| 63 | P17 | 0.4 | 1 | 1 | 100000 | 1 | 100000 | 5 | 0 | 0 | NA | 0 | 1 | 0 |
| 91 | P17 | 0.22 | 2 | 1 | 20000 | 1 | 20000 | 4.301029996 | 4794.72 | 3.680763251 | 0 | 0 | 1 | 1 |
| 120 | P17 | 0.53 | 3 | 0.8 | 800000000 | 1 | 640000000 | 8.806179974 | 9097327.893 | 6.958913848 | 2.486285 | 1 | 1 | 1 |
| 148 | P17 | 0.18 | 4 | 0.8 | 1E+09 | 1 | 800000000 | 8.903089987 | 34408585.88 | 7.536666824 | 2.965396 | 1 | 1 | NA |
| 41 | P22 | 0.42 | 0 |  | 0 | 0 | 0 | 0 | 0 | 0 | 0 | 0 | 0 | 0 |
| 66 | P22 | 0.21 | 1 |  |  | 1 | 5000 | 3.698970004 | 32.1387228 | 1.507028614 | 0.647818 | 0 | 0 | 0 |
| 93 | P22 | 0.31 | 2 |  |  | 1 | 5000 | 3.698970004 | 0.077674493 | 0 | 0 | 0 | 0 | 0 |
| 122 | P22 | 0.39 | 3 |  | 0 | 0 | 0 | 0 | 0.165911983 | 0 | 0 | 0 | 0 | 0 |
| 151 | P22 | 0.23 | 4 |  | 0 | 0 | 0 | 0 | 1.147459878 | 0.059737509 | 0 | 0 | 0 | NA |
| 45 | P29 | 0.3 | 0 |  | 0 | 0 | 0 | 0 | 0 | 0 | 0 | 0 | 0 | 0 |
| 70 | P29 | 0.49 | 1 |  | 0 | 0 | 0 | 0 | 0 | 0 | 0 | 0 | 0 | 0 |
| 97 | P29 | 0.29 | 2 |  | 0 | 0 | 0 | 0 | 0.11287 | 0 | 0 | 0 | 0 | 0 |
| 126 | P29 | 0.2 | 3 |  | 0 | 0 | 0 | 0 | 1.863799 | 0.270399074 | 0 | 0 | 0 | 0 |
| 155 | P29 | 0.29 | 4 |  |  | 1 | 5000 | 3.698970004 | 1566.195999 | 3.19484611 | 0 | 0 | 0 | NA |
| 46 | P30 | 0.3 | 0 | 0 | 1100000 | 0 | 0 | 0 | 0 | 0 | 1.463081 | 0 | 0 | 0 |
| 71 | P30 | 0.22 | 1 | 1 | 30000 | 1 | 30000 | 4.477121255 | 115.0189051 | 2.060769229 | 0 | 0 | 0 | 0 |
| 98 | P30 | 0.39 | 2 | 1 | 1100000 | 1 | 1100000 | 6.041392685 | 2588.539839 | 3.413054853 | 0 | 0 | 0 | 0 |
| 127 | P30 | 0.26 | 3 | 1 | 6200000 | 1 | 6200000 | 6.792391689 | 1168787984 | 9.067735738 | 0 | 0 | 0 | 0 |
| 156 | P30 | 0.28 | 4 | 1 | 7900000 | 1 | 7900000 | 6.897627091 | 3710980.991 | 6.56948873 | 0.735182 | 0 | 0 | NA |
| 101 | P33 |  | 2 |  |  | 0 | 0 | 0 | 0 | 0 | 0 | 0 | 0 | NA |
| 159 | P33 |  | 4 |  |  | 0 | 0 | 0 | 603.165509 | 2.780436499 | 0 | 0 | 0 | NA |
| 105 | P38 |  | 2 |  |  | 1 | 3100000000 | 9.491361694 | 9844757.52 | 6.993205024 | 4.054131 | 1 | 1 | 1 |
| 77 | P41 | 0.58 | 1 | 0.6 | 460000 | 1 | 280000 | 5.447158031 | 109.31 | 2.038659894 | 0 | 0 | 0 | 0 |
| 106 | P41 | 0.27 | 2 | 0.6 | 1800000 | 1 | 1100000 | 6.041392685 | 0.54868418 | 0 | 0 | 0 | 0 | 0 |
| 134 | P41 | 0.34 | 3 | 0 | 2100000 | 0 | 0 | 0 | 17.66921596 | 1.247217279 | 0.363512 | 0 | 0 | 0 |
| 164 | P41 | 0.12 | 4 | 0.6 | 2500000 | 1 | 1500000 | 6.176091259 | 3.10606329 | 0.492210301 | 0 | 0 | 0 | NA |
