# Supplementary material for: Individual-specific changes in the human gut microbiota after challenge with enterotoxigenic Escherichia coli and subsequent ciprofloxacin treatment
Source: BMC Genomics. 2016 Jun 8;17:440. doi: 10.1186/s12864-016-2777-0 (PMC4898365; doi:10.1186/s12864-016-2777-0)
Supplement: Additional file 7: Table S3. — Differentially abundant OTUs across multiple conditions. (DOCX 344 kb) [file 12864_2016_2777_MOESM7_ESM.docx]

Table s3A

| Number of patients | Number of reads in Case samples | Number of reads in Controls | Number of positive Case samples | Number of positive Controls | OTU | fold change | 2.5 confint | 97.5 confint | agree |
| --- | --- | --- | --- | --- | --- | --- | --- | --- | --- |
| 5 | 220 | 42 | 11 | 9 | 7161 | 1.95559527 | 0.288481 | 3.62270954 | TRUE |
| 3 | 45 | 0 | 7 | 0 | 7560 | 0.95503011 | 0.11780228 | 1.79225795 | TRUE |
| 2 | 89 | 0 | 6 | 0 | 8020 | 1.34342634 | 0.21075359 | 2.4760991 | TRUE |
| 2 | 12 | 0 | 5 | 0 | 9033 | 0.65379488 | 0.18546926 | 1.1221205 | TRUE |
| 5 | 17 | 3 | 5 | 3 | 10387 | 0.56074582 | 0.11785653 | 1.00363512 | TRUE |
| 4 | 13 | 5 | 6 | 2 | 10918 | 0.62563663 | 0.16310927 | 1.088164 | TRUE |
| 5 | 46 | 4 | 8 | 4 | 11052 | 1.10173386 | 0.25834953 | 1.94511818 | TRUE |
| 3 | 17 | 0 | 6 | 0 | 11299 | 0.73181732 | 0.11947977 | 1.34415487 | TRUE |
| 4 | 5 | 1 | 4 | 1 | 12412 | 0.33507901 | 0.11898421 | 0.5511738 | TRUE |
| 4 | 11 | 3 | 4 | 2 | 14616 | 0.48212875 | 0.06199346 | 0.90226405 | TRUE |
| 2 | 9 | 0 | 5 | 0 | 14645 | 0.51828238 | 0.11060304 | 0.92596173 | TRUE |
| 3 | 10 | 1 | 4 | 1 | 18831 | 0.47865762 | 0.14429276 | 0.81302248 | TRUE |
| 3 | 10 | 1 | 4 | 1 | 21552 | 0.40247501 | 0.02642813 | 0.77852189 | TRUE |
| 4 | 7 | 1 | 6 | 1 | 25920 | 0.49250811 | 0.26638091 | 0.71863531 | TRUE |
| 4 | 7 | 3 | 6 | 3 | 27408 | 0.39189131 | 0.05331347 | 0.73046914 | TRUE |
| 4 | 4 | 2 | 4 | 2 | 27908 | 0.31130418 | 0.05909281 | 0.56351556 | TRUE |
| 2 | 113 | 0 | 8 | 0 | 29555 | 1.45079659 | 0.12760944 | 2.77398374 | TRUE |
| 7 | 44 | 35 | 10 | 9 | 30000 | 1.17663763 | 0.32719494 | 2.02608033 | TRUE |
| 4 | 61 | 21 | 8 | 5 | 31052 | 1.20846514 | 0.09675773 | 2.32017255 | TRUE |
| 7 | 12 | 11 | 7 | 6 | 34210 | 0.63462467 | 0.11530764 | 1.1539417 | TRUE |
| 3 | 0 | 8 | 0 | 5 | 34895 | -0.3206486 | -0.6312942 | -0.0100031 | TRUE |
| 3 | 21 | 1 | 5 | 1 | 37561 | 0.68781606 | 0.04305776 | 1.33257435 | TRUE |
| 4 | 0 | 7 | 0 | 6 | 39031 | -0.2504333 | -0.4955516 | -0.0053149 | TRUE |
| 3 | 7 | 2 | 4 | 1 | 39677 | 0.36577383 | 0.0563726 | 0.67517506 | TRUE |
| 6 | 3648 | 56 | 12 | 5 | 40456 | 3.9620957 | 1.23741358 | 6.68677781 | TRUE |
| 3 | 0 | 7 | 0 | 5 | 40968 | -0.267287 | -0.5302811 | -0.0042929 | TRUE |
| 6 | 9 | 7 | 6 | 5 | 41897 | 0.47570282 | 0.0572268 | 0.89417883 | TRUE |
| 7 | 13 | 7 | 6 | 4 | 42660 | 0.48668526 | 0.03468948 | 0.93868104 | TRUE |
| 5 | 7 | 7 | 4 | 4 | 42829 | 0.44064062 | 0.03564005 | 0.84564119 | TRUE |
| 8 | 5 | 539 | 4 | 29 | 44063 | -2.299595 | -4.0924329 | -0.506757 | TRUE |
| 4 | 5 | 1 | 4 | 1 | 44709 | 0.34359557 | 0.14292861 | 0.54426253 | TRUE |
| 3 | 13 | 2 | 5 | 2 | 44850 | 0.50354995 | 0.05974117 | 0.94735873 | TRUE |
| 6 | 7 | 5 | 6 | 3 | 45956 | 0.40838767 | 0.0995061 | 0.71726925 | TRUE |
| 4 | 8 | 1 | 7 | 1 | 46540 | 0.5630085 | 0.33702609 | 0.78899092 | TRUE |
| 5 | 0 | 22 | 0 | 9 | 49233 | -0.5282392 | -1.0533477 | -0.0031307 | TRUE |
| 5 | 26 | 2 | 6 | 2 | 49383 | 0.86344072 | 0.18256727 | 1.54431416 | TRUE |
| 4 | 91 | 8 | 10 | 5 | 50418 | 1.48416096 | 0.41654185 | 2.55178006 | TRUE |
| 4 | 0 | 8 | 0 | 6 | 50611 | -0.3224727 | -0.5804318 | -0.0645135 | TRUE |
| 5 | 0 | 12 | 0 | 10 | 50630 | -0.3299817 | -0.6550911 | -0.0048722 | TRUE |
| 3 | 46 | 0 | 6 | 0 | 54652 | 1.08759449 | 0.08673628 | 2.0884527 | TRUE |
| 5 | 19 | 3 | 6 | 3 | 55756 | 0.7616852 | 0.30103895 | 1.22233144 | TRUE |
| 4 | 4 | 2 | 3 | 2 | 55767 | 0.26934993 | 0.02885492 | 0.50984494 | TRUE |
| 7 | 10 | 7 | 6 | 5 | 56166 | 0.47188879 | 0.01952566 | 0.92425192 | TRUE |
| 3 | 43 | 0 | 7 | 0 | 57771 | 0.95305491 | 0.05974829 | 1.84636153 | TRUE |
| 9 | 8 | 249 | 3 | 26 | 58092 | -1.5939697 | -3.1192121 | -0.0687272 | TRUE |
| 10 | 6 | 76 | 4 | 23 | 61406 | -0.8158752 | -1.6265876 | -0.0051628 | TRUE |
| 5 | 16 | 2 | 4 | 2 | 61607 | 0.73604681 | 0.06479894 | 1.40729469 | TRUE |
| 7 | 15 | 22 | 9 | 9 | 61938 | 0.67797911 | 0.07673026 | 1.27922795 | TRUE |
| 6 | 6 | 4 | 5 | 3 | 62899 | 0.35142547 | 0.06643456 | 0.63641638 | TRUE |
| 11 | 493 | 95 | 12 | 17 | 64548 | 1.78520363 | 0.10562134 | 3.46478591 | TRUE |
| 10 | 54 | 23 | 9 | 12 | 66329 | 0.97506106 | 0.11968736 | 1.83043477 | TRUE |
| 5 | 3 | 3 | 3 | 3 | 67095 | 0.25288436 | 0.049085 | 0.45668371 | TRUE |
| 3 | 11 | 0 | 5 | 0 | 71962 | 0.52083404 | 0.09937508 | 0.942293 | TRUE |
| 4 | 0 | 42 | 0 | 10 | 72729 | -0.7302543 | -1.456844 | -0.0036645 | TRUE |
| 4 | 9 | 3 | 6 | 2 | 73541 | 0.52295548 | 0.07177415 | 0.97413681 | TRUE |
| 4 | 7 | 1 | 4 | 1 | 75130 | 0.38946379 | 0.13438606 | 0.64454153 | TRUE |
| 6 | 1 | 299 | 1 | 21 | 78889 | -1.8648939 | -3.5218664 | -0.2079214 | TRUE |
| 6 | 7 | 2 | 5 | 2 | 79546 | 0.37438995 | 0.10863799 | 0.64014192 | TRUE |
| 5 | 4 | 2 | 3 | 2 | 81205 | 0.22550437 | 0.00046762 | 0.45054112 | TRUE |
| 6 | 2 | 255 | 2 | 20 | 83479 | -1.7574361 | -3.3275391 | -0.1873332 | TRUE |
| 5 | 0 | 17 | 0 | 8 | 88862 | -0.4759604 | -0.8715758 | -0.080345 | TRUE |
| 3 | 6 | 1 | 4 | 1 | 91937 | 0.35427617 | 0.01898173 | 0.68957061 | TRUE |
| 3 | 12 | 4 | 4 | 2 | 101155 | 0.42039715 | 0.00155062 | 0.83924368 | TRUE |
| 4 | 14 | 3 | 6 | 2 | 104573 | 0.54650108 | 0.0309364 | 1.06206577 | TRUE |
| 7 | 13 | 10 | 6 | 6 | 110534 | 0.69976687 | 0.20879511 | 1.19073863 | TRUE |
| 4 | 7 | 1 | 4 | 1 | 111895 | 0.38970265 | 0.11590159 | 0.66350371 | TRUE |
| 4 | 8 | 2 | 6 | 2 | 111921 | 0.55414526 | 0.06650469 | 1.04178583 | TRUE |
| 3 | 26 | 1 | 5 | 1 | 114917 | 0.81952409 | 0.00097144 | 1.63807674 | TRUE |
| 2 | 8 | 0 | 6 | 0 | 115896 | 0.42214794 | 0.04974854 | 0.79454735 | TRUE |
| 2 | 8 | 0 | 5 | 0 | 120438 | 0.4339122 | 0.0999878 | 0.76783659 | TRUE |
| 3 | 23 | 3 | 4 | 1 | 122796 | 0.67835428 | 0.00987673 | 1.34683184 | TRUE |
| 8 | 13 | 19 | 6 | 5 | 122798 | 0.52934109 | 0.0208178 | 1.03786438 | TRUE |
| 3 | 8 | 1 | 4 | 1 | 124371 | 0.41409948 | 0.05937915 | 0.76881981 | TRUE |
| 3 | 7 | 0 | 7 | 0 | 124610 | 0.48474791 | 0.1338996 | 0.83559623 | TRUE |
| 5 | 9 | 3 | 4 | 2 | 126801 | 0.40554899 | 0.06847016 | 0.74262783 | TRUE |
| 4 | 6 | 1 | 4 | 1 | 128385 | 0.31731465 | 0.07211706 | 0.56251224 | TRUE |
| 3 | 18 | 0 | 6 | 0 | 128929 | 0.71380612 | 0.04694115 | 1.3806711 | TRUE |
| 4 | 14 | 6 | 3 | 4 | 133358 | 0.50526411 | 0.01686171 | 0.99366651 | TRUE |
| 5 | 5 | 2 | 4 | 2 | 146025 | 0.27088112 | 0.03131463 | 0.51044761 | TRUE |
| 4 | 1 | 10 | 1 | 5 | 3 | -0.0699768 | -0.4907636 | 0.35080994 | FALSE |
| 7 | 2 | 8 | 1 | 8 | 65 | -0.116074 | -0.3953569 | 0.16320885 | FALSE |
| 7 | 14 | 8 | 6 | 7 | 84 | 0.37304369 | -0.1365304 | 0.8826178 | FALSE |
| 4 | 1 | 8 | 1 | 5 | 364 | -0.2145048 | -0.5944039 | 0.16539423 | FALSE |
| 3 | 1 | 39 | 1 | 6 | 540 | -0.4135225 | -1.4555682 | 0.6285231 | FALSE |
| 4 | 0 | 36 | 0 | 9 | 2768 | -0.388601 | -1.134096 | 0.35689398 | FALSE |
| 4 | 34 | 11 | 3 | 5 | 2794 | 0.50510088 | -0.666881 | 1.67708275 | FALSE |
| 5 | 5 | 45 | 2 | 8 | 2831 | -0.5064358 | -1.684544 | 0.67167245 | FALSE |
| 6 | 482 | 1689 | 5 | 17 | 3084 | -0.12685 | -3.2700223 | 3.01632222 | FALSE |
| 3 | 1 | 11 | 1 | 4 | 3092 | -0.0419656 | -0.45199 | 0.36805872 | FALSE |
| 9 | 9 | 132 | 5 | 12 | 3115 | 0.20857834 | -0.5654413 | 0.98259801 | FALSE |
| 5 | 25 | 73 | 3 | 8 | 3194 | 0.24393731 | -1.0193061 | 1.5071807 | FALSE |
| 5 | 452 | 1559 | 4 | 18 | 3200 | -0.3962743 | -3.5482404 | 2.7556918 | FALSE |
| 4 | 5 | 72 | 3 | 7 | 3209 | -0.0140385 | -1.0154855 | 0.98740859 | FALSE |
| 3 | 2 | 34 | 1 | 6 | 3239 | -0.1677178 | -1.0283 | 0.69286433 | FALSE |
| 4 | 0 | 17 | 0 | 10 | 3296 | -0.3215584 | -0.8545664 | 0.21144954 | FALSE |
| 4 | 0 | 149 | 0 | 14 | 3321 | -1.112373 | -2.4937903 | 0.26904437 | FALSE |
| 4 | 0 | 62 | 0 | 11 | 3376 | -0.6951195 | -1.670623 | 0.28038393 | FALSE |
| 5 | 343 | 2290 | 4 | 14 | 3380 | -0.1451168 | -3.2115519 | 2.92131829 | FALSE |
| 5 | 12 | 100 | 3 | 13 | 3381 | -0.1424173 | -1.3414466 | 1.05661207 | FALSE |
| 5 | 46 | 209 | 4 | 15 | 3399 | -0.1349019 | -1.8908812 | 1.6210773 | FALSE |
| 2 | 0 | 38 | 0 | 7 | 3403 | -0.4808974 | -1.4369796 | 0.47518485 | FALSE |
| 3 | 2 | 14 | 2 | 5 | 3409 | 0.02190908 | -0.5234186 | 0.56723678 | FALSE |
| 4 | 1 | 49 | 1 | 6 | 3415 | -0.0761562 | -0.8198115 | 0.66749915 | FALSE |
| 2 | 1 | 433 | 1 | 6 | 3420 | -0.5147478 | -2.2451791 | 1.21568345 | FALSE |
| 2 | 1 | 75 | 1 | 4 | 3421 | -0.1066848 | -1.2095991 | 0.99622954 | FALSE |
| 7 | 14 | 358 | 2 | 22 | 3442 | -0.8354447 | -2.7727843 | 1.10189479 | FALSE |
| 6 | 3 | 155 | 1 | 23 | 3467 | -1.2298455 | -2.5625933 | 0.10290233 | FALSE |
| 6 | 3 | 48 | 2 | 5 | 3482 | -0.2667284 | -0.933877 | 0.40042027 | FALSE |
| 5 | 180 | 705 | 4 | 19 | 3546 | -0.592904 | -3.1827384 | 1.99693043 | FALSE |
| 3 | 3 | 15 | 1 | 5 | 3589 | -0.0320369 | -0.5891394 | 0.52506568 | FALSE |
| 5 | 3 | 41 | 2 | 8 | 3599 | -0.0330084 | -0.8519082 | 0.78589139 | FALSE |
| 2 | 0 | 17 | 0 | 5 | 3609 | -0.0949694 | -0.6457144 | 0.45577569 | FALSE |
| 2 | 1 | 13 | 1 | 4 | 3631 | -0.0280184 | -0.5363874 | 0.48035052 | FALSE |
| 2 | 1 | 36 | 1 | 4 | 3635 | 0.0015654 | -0.8114112 | 0.81454194 | FALSE |
| 3 | 1 | 17 | 1 | 5 | 3636 | -0.0509125 | -0.6353627 | 0.53353768 | FALSE |
| 3 | 0 | 46 | 0 | 7 | 3784 | -0.2173957 | -0.9427064 | 0.50791499 | FALSE |
| 5 | 1 | 20 | 1 | 10 | 3785 | -0.2419305 | -0.7989213 | 0.31506025 | FALSE |
| 3 | 8 | 57 | 4 | 3 | 3786 | -0.1852194 | -1.4710125 | 1.10057364 | FALSE |
| 5 | 78 | 264 | 4 | 17 | 3858 | -0.2355458 | -2.3068907 | 1.8357991 | FALSE |
| 1 | 0 | 58 | 0 | 5 | 3877 | -0.2390852 | -1.21673 | 0.73855971 | FALSE |
| 4 | 2 | 4 | 2 | 4 | 3902 | 0.07324331 | -0.2454172 | 0.39190379 | FALSE |
| 3 | 15 | 40 | 4 | 3 | 4060 | 0.01523563 | -1.3322633 | 1.36273452 | FALSE |
| 2 | 0 | 14 | 0 | 6 | 4118 | -0.3479796 | -0.9306069 | 0.23464769 | FALSE |
| 2 | 2 | 9 | 2 | 3 | 4139 | 0.01640468 | -0.4339835 | 0.46679283 | FALSE |
| 6 | 2 | 100 | 2 | 15 | 4191 | -0.692609 | -1.9947169 | 0.60949892 | FALSE |
| 8 | 45 | 156 | 7 | 21 | 4192 | -0.0051046 | -1.5973494 | 1.58714015 | FALSE |
| 5 | 1 | 8 | 1 | 6 | 4203 | -0.1196551 | -0.4996238 | 0.26031353 | FALSE |
| 5 | 1 | 36 | 1 | 13 | 4257 | -0.4411668 | -1.1495026 | 0.26716894 | FALSE |
| 4 | 0 | 10 | 0 | 6 | 4308 | -0.1186729 | -0.4901464 | 0.25280064 | FALSE |
| 2 | 0 | 49 | 0 | 10 | 4343 | -0.5682342 | -1.5446826 | 0.40821419 | FALSE |
| 4 | 3 | 45 | 2 | 12 | 4344 | -0.4178173 | -1.2530146 | 0.41737997 | FALSE |
| 5 | 47 | 509 | 5 | 10 | 4366 | 0.49469802 | -1.1943036 | 2.18369968 | FALSE |
| 2 | 2 | 49 | 2 | 4 | 4487 | -0.0226208 | -0.94378 | 0.89853836 | FALSE |
| 4 | 6 | 42 | 2 | 10 | 4493 | -0.1379377 | -1.0001681 | 0.72429273 | FALSE |
| 3 | 1 | 9 | 1 | 5 | 4516 | -0.0311032 | -0.3959886 | 0.33378215 | FALSE |
| 1 | 0 | 65 | 0 | 5 | 4524 | -0.268316 | -1.3088345 | 0.77220255 | FALSE |
| 1 | 0 | 100 | 0 | 6 | 4525 | -0.3373982 | -1.4180255 | 0.74322904 | FALSE |
| 8 | 18 | 111 | 6 | 15 | 4577 | -0.0325351 | -1.2713481 | 1.20627787 | FALSE |
| 3 | 3 | 3 | 2 | 3 | 4675 | 0.12950585 | -0.1438191 | 0.40283079 | FALSE |
| 3 | 10 | 4 | 4 | 3 | 4678 | 0.40939405 | -0.104534 | 0.92332207 | FALSE |
| 3 | 65 | 664 | 4 | 11 | 4792 | -0.1151483 | -2.4663205 | 2.23602394 | FALSE |
| 2 | 7 | 5 | 4 | 3 | 4833 | 0.1925877 | -0.3610774 | 0.74625279 | FALSE |
| 4 | 1 | 11 | 1 | 5 | 4840 | -0.0533062 | -0.4742603 | 0.36764785 | FALSE |
| 1 | 0 | 69 | 0 | 5 | 4879 | -0.3391954 | -1.3644398 | 0.68604901 | FALSE |
| 3 | 2 | 16 | 1 | 5 | 4885 | 0.08284417 | -0.5052452 | 0.67093356 | FALSE |
| 6 | 14 | 63 | 4 | 9 | 4927 | 0.00106692 | -1.3411452 | 1.34327902 | FALSE |
| 4 | 3 | 5 | 1 | 4 | 4955 | 0.1490935 | -0.1461738 | 0.44436082 | FALSE |
| 4 | 0 | 12 | 0 | 6 | 4958 | -0.0535593 | -0.3931786 | 0.28606005 | FALSE |
| 2 | 1 | 17 | 1 | 5 | 4967 | -0.0634887 | -0.6745869 | 0.54760946 | FALSE |
| 1 | 0 | 25 | 0 | 5 | 4971 | -0.1862768 | -0.879261 | 0.50670742 | FALSE |
| 1 | 0 | 24 | 0 | 6 | 5129 | -0.2849609 | -1.0006806 | 0.43075875 | FALSE |
| 3 | 83 | 380 | 4 | 11 | 5170 | -0.0069815 | -2.162147 | 2.14818395 | FALSE |
| 3 | 0 | 10 | 0 | 6 | 5197 | -0.1599339 | -0.5210164 | 0.20114866 | FALSE |
| 3 | 4 | 5 | 2 | 4 | 5219 | 0.14751559 | -0.2276834 | 0.52271453 | FALSE |
| 1 | 0 | 38 | 0 | 5 | 5236 | -0.1106969 | -0.8696976 | 0.6483037 | FALSE |
| 8 | 21 | 14 | 5 | 9 | 5273 | 0.2980582 | -0.3335668 | 0.92968323 | FALSE |
| 5 | 1 | 189 | 1 | 13 | 5284 | -0.5851991 | -2.0117813 | 0.84138306 | FALSE |
| 4 | 3 | 8 | 2 | 4 | 5286 | 0.12470912 | -0.2389718 | 0.48839002 | FALSE |
| 5 | 4 | 77 | 3 | 4 | 5299 | -0.2107969 | -1.7497945 | 1.32820081 | FALSE |
| 3 | 2 | 10 | 2 | 5 | 5341 | 0.04713419 | -0.4606014 | 0.55486978 | FALSE |
| 2 | 2 | 10 | 2 | 4 | 5342 | 0.07807483 | -0.3920551 | 0.54820477 | FALSE |
| 5 | 2 | 24 | 2 | 9 | 5409 | -0.1694796 | -0.8422882 | 0.50332898 | FALSE |
| 3 | 3 | 9 | 2 | 3 | 5545 | 0.11098525 | -0.2998265 | 0.52179701 | FALSE |
| 3 | 0 | 82 | 0 | 8 | 5715 | -0.3270776 | -1.3953487 | 0.74119346 | FALSE |
| 10 | 341 | 298 | 11 | 23 | 5747 | 0.9234068 | -1.3133455 | 3.16015912 | FALSE |
| 5 | 6 | 42 | 5 | 10 | 5754 | -0.1231945 | -1.1140335 | 0.86764458 | FALSE |
| 3 | 0 | 70 | 0 | 8 | 5755 | -0.4242552 | -1.4484585 | 0.59994805 | FALSE |
| 3 | 1 | 23 | 1 | 5 | 5758 | -0.3105716 | -1.2025382 | 0.58139496 | FALSE |
| 8 | 128 | 63 | 8 | 7 | 5762 | 1.0955381 | -0.3060839 | 2.49716013 | FALSE |
| 5 | 82 | 164 | 8 | 7 | 5828 | 0.92168463 | -1.0625111 | 2.9058804 | FALSE |
| 8 | 248 | 68 | 10 | 17 | 5872 | 1.57659905 | -0.052885 | 3.20608305 | FALSE |
| 2 | 0 | 7 | 0 | 5 | 5958 | -0.1933919 | -0.5374356 | 0.15065173 | FALSE |
| 8 | 66 | 725 | 5 | 21 | 5978 | -0.7727766 | -2.4920962 | 0.94654299 | FALSE |
| 1 | 0 | 1172 | 0 | 6 | 6007 | -0.9536764 | -3.3874351 | 1.48008234 | FALSE |
| 1 | 0 | 59 | 0 | 5 | 6080 | -0.5112207 | -1.506888 | 0.48444657 | FALSE |
| 2 | 0 | 11 | 0 | 5 | 6171 | -0.1418669 | -0.5307529 | 0.24701914 | FALSE |
| 2 | 0 | 100 | 0 | 7 | 6277 | -0.0907443 | -1.110403 | 0.92891432 | FALSE |
| 5 | 2 | 4 | 2 | 4 | 6308 | 0.01083244 | -0.2252041 | 0.246869 | FALSE |
| 5 | 3 | 5 | 2 | 5 | 6309 | 0.05782575 | -0.2828277 | 0.39847919 | FALSE |
| 6 | 17 | 22 | 5 | 9 | 6329 | 0.22250172 | -0.6143124 | 1.05931588 | FALSE |
| 4 | 22 | 14 | 3 | 3 | 6333 | 0.46879332 | -0.0931198 | 1.03070643 | FALSE |
| 4 | 8 | 15 | 3 | 7 | 6341 | 0.04351338 | -0.6498223 | 0.73684905 | FALSE |
| 5 | 0 | 23 | 0 | 14 | 6362 | -0.4512735 | -0.9735563 | 0.07100933 | FALSE |
| 2 | 30 | 1178 | 2 | 5 | 6405 | 0.07683098 | -2.5541675 | 2.70782943 | FALSE |
| 8 | 79 | 217 | 4 | 14 | 6421 | -0.3262464 | -1.5311949 | 0.87870203 | FALSE |
| 2 | 5 | 909 | 3 | 6 | 6438 | -0.5805617 | -2.4678417 | 1.30671838 | FALSE |
| 6 | 243 | 204 | 11 | 12 | 6472 | 1.43222356 | -0.6130286 | 3.47747567 | FALSE |
| 4 | 101 | 83 | 7 | 5 | 6474 | 0.94407246 | -0.7901182 | 2.67826315 | FALSE |
| 2 | 0 | 114 | 0 | 7 | 6532 | -0.9517412 | -2.5679313 | 0.66444891 | FALSE |
| 6 | 1 | 45 | 1 | 12 | 6550 | -0.5745991 | -1.3918066 | 0.24260833 | FALSE |
| 5 | 1 | 13 | 1 | 10 | 6554 | -0.2646898 | -0.7440261 | 0.2146464 | FALSE |
| 4 | 11 | 18 | 5 | 5 | 6564 | 0.49345721 | -0.3104745 | 1.29738891 | FALSE |
| 4 | 1 | 9 | 1 | 5 | 6588 | -0.1968851 | -0.7181238 | 0.32435354 | FALSE |
| 3 | 1 | 11 | 1 | 5 | 6770 | 0.05841038 | -0.4171773 | 0.53399803 | FALSE |
| 5 | 19 | 218 | 3 | 10 | 6778 | 0.35578722 | -1.2120854 | 1.92365983 | FALSE |
| 2 | 0 | 9 | 0 | 5 | 6925 | -0.2586912 | -0.8106283 | 0.29324594 | FALSE |
| 3 | 1 | 12 | 1 | 5 | 6945 | -0.0924287 | -0.5875507 | 0.40269325 | FALSE |
| 2 | 0 | 14 | 0 | 5 | 6984 | -0.0870168 | -0.5714469 | 0.39741325 | FALSE |
| 3 | 1 | 13 | 1 | 6 | 7023 | 0.00249282 | -0.4889482 | 0.49393384 | FALSE |
| 8 | 23 | 44 | 7 | 16 | 7071 | 0.1058636 | -0.7277714 | 0.9394986 | FALSE |
| 4 | 7 | 11 | 2 | 6 | 7114 | 0.59123582 | -0.3469862 | 1.5294578 | FALSE |
| 6 | 41 | 46 | 8 | 7 | 7154 | 0.77484431 | -0.4583832 | 2.00807184 | FALSE |
| 4 | 1 | 5 | 1 | 5 | 7179 | -0.0093658 | -0.2845698 | 0.2658383 | FALSE |
| 11 | 107 | 672 | 8 | 26 | 7183 | -1.0702969 | -3.0702134 | 0.92961959 | FALSE |
| 6 | 48 | 15 | 8 | 7 | 7258 | 0.8085161 | -0.125581 | 1.74261323 | FALSE |
| 4 | 143 | 26 | 6 | 5 | 7270 | 1.08251541 | -0.4021573 | 2.56718808 | FALSE |
| 5 | 27 | 155 | 3 | 15 | 7294 | -0.5475374 | -2.2321562 | 1.13708133 | FALSE |
| 3 | 0 | 9 | 0 | 5 | 7347 | -0.226237 | -0.6232089 | 0.17073485 | FALSE |
| 2 | 0 | 9 | 0 | 5 | 7388 | -0.1115381 | -0.5201952 | 0.29711899 | FALSE |
| 2 | 0 | 35 | 0 | 6 | 7471 | -0.4842125 | -1.4475623 | 0.47913739 | FALSE |
| 3 | 2 | 83 | 2 | 5 | 7488 | -0.1751578 | -0.9634937 | 0.61317808 | FALSE |
| 2 | 0 | 12 | 0 | 5 | 7495 | -0.2329705 | -0.7480284 | 0.28208736 | FALSE |
| 2 | 0 | 90 | 0 | 7 | 7848 | -0.1267291 | -1.2636925 | 1.01023434 | FALSE |
| 2 | 0 | 39 | 0 | 5 | 7894 | -0.2060127 | -1.0505242 | 0.63849883 | FALSE |
| 3 | 6 | 15 | 2 | 6 | 7936 | 0.18246882 | -0.4305998 | 0.79553746 | FALSE |
| 3 | 0 | 40 | 0 | 9 | 8063 | -0.3122808 | -1.1174682 | 0.49290664 | FALSE |
| 4 | 1 | 67 | 1 | 10 | 8109 | -0.772151 | -1.779973 | 0.235671 | FALSE |
| 11 | 112 | 557 | 10 | 28 | 8138 | -0.6321714 | -2.6883536 | 1.4240108 | FALSE |
| 4 | 2 | 4 | 2 | 4 | 8141 | -0.0020169 | -0.2429685 | 0.23893478 | FALSE |
| 2 | 0 | 20 | 0 | 7 | 8259 | -0.2961609 | -1.1052031 | 0.51288119 | FALSE |
| 3 | 2 | 20 | 1 | 5 | 8282 | -0.1711898 | -0.7190284 | 0.3766489 | FALSE |
| 3 | 34 | 1339 | 1 | 9 | 8314 | -0.5618843 | -3.3325734 | 2.20880485 | FALSE |
| 2 | 0 | 96 | 0 | 6 | 8322 | -0.4217768 | -1.3132474 | 0.46969368 | FALSE |
| 5 | 1 | 5 | 1 | 5 | 8371 | -0.0639617 | -0.2936314 | 0.16570807 | FALSE |
| 11 | 95 | 2260 | 11 | 33 | 8377 | -1.8853245 | -4.4451015 | 0.67445248 | FALSE |
| 6 | 3 | 51 | 2 | 12 | 8378 | -0.5405056 | -1.7014886 | 0.62047737 | FALSE |
| 9 | 35 | 227 | 7 | 24 | 8421 | -0.9196041 | -2.5989612 | 0.75975301 | FALSE |
| 5 | 16 | 8 | 5 | 5 | 8448 | 0.46758976 | -0.1354578 | 1.0706373 | FALSE |
| 3 | 2 | 4 | 1 | 4 | 8489 | 0.09417112 | -0.1917643 | 0.3801065 | FALSE |
| 6 | 73 | 109 | 7 | 13 | 8497 | 0.47129707 | -1.1959725 | 2.13856665 | FALSE |
| 1 | 0 | 9 | 0 | 5 | 8510 | -0.1128716 | -0.5474466 | 0.3217034 | FALSE |
| 1 | 0 | 16 | 0 | 5 | 8521 | -0.1831268 | -0.7817792 | 0.41552547 | FALSE |
| 4 | 9 | 95 | 3 | 12 | 8529 | -0.360135 | -1.6307098 | 0.91043981 | FALSE |
| 2 | 0 | 46 | 0 | 7 | 8530 | -0.2457126 | -1.2331845 | 0.74175928 | FALSE |
| 1 | 0 | 157 | 0 | 5 | 8538 | -0.5059382 | -1.4933788 | 0.48150239 | FALSE |
| 1 | 0 | 15 | 0 | 6 | 8549 | -0.1774616 | -0.7740424 | 0.41911923 | FALSE |
| 4 | 143 | 8 | 6 | 3 | 8569 | 1.43018511 | -0.062939 | 2.92330919 | FALSE |
| 1 | 0 | 133 | 0 | 6 | 8595 | -0.5681297 | -2.0009721 | 0.86471264 | FALSE |
| 1 | 0 | 282 | 0 | 6 | 8597 | -0.5474363 | -2.2037261 | 1.10885355 | FALSE |
| 3 | 2 | 9 | 2 | 3 | 8649 | 0.04657921 | -0.3995212 | 0.49267961 | FALSE |
| 5 | 2 | 16 | 2 | 11 | 8671 | -0.2044513 | -0.6743334 | 0.26543081 | FALSE |
| 1 | 0 | 315 | 0 | 6 | 8691 | -0.5737138 | -2.3583795 | 1.21095181 | FALSE |
| 2 | 5 | 13 | 3 | 4 | 8713 | 0.14729222 | -0.4474402 | 0.74202464 | FALSE |
| 4 | 4 | 9 | 3 | 5 | 8902 | 0.11967252 | -0.3322938 | 0.57163887 | FALSE |
| 2 | 2 | 12 | 1 | 5 | 8924 | 0.01338903 | -0.5219347 | 0.54871276 | FALSE |
| 8 | 14 | 25 | 8 | 14 | 9008 | 0.32818147 | -0.2991258 | 0.95548874 | FALSE |
| 5 | 3 | 63 | 2 | 12 | 9018 | -0.2090192 | -1.1831663 | 0.7651279 | FALSE |
| 6 | 12 | 67 | 4 | 12 | 9057 | 0.12842218 | -1.1081731 | 1.36501741 | FALSE |
| 11 | 56 | 301 | 9 | 30 | 9183 | -0.6905543 | -2.2345418 | 0.85343312 | FALSE |
| 3 | 16 | 8 | 3 | 3 | 9359 | 0.30286519 | -0.3670521 | 0.9727825 | FALSE |
| 1 | 0 | 91 | 0 | 5 | 9394 | -0.5100299 | -1.8633566 | 0.84329672 | FALSE |
| 5 | 40 | 2501 | 4 | 10 | 9421 | -0.8255641 | -4.048106 | 2.39697788 | FALSE |
| 2 | 0 | 123 | 0 | 7 | 9435 | -0.9670345 | -2.6486918 | 0.71462284 | FALSE |
| 9 | 17 | 1058 | 4 | 18 | 9458 | -1.4966085 | -4.0761284 | 1.08291141 | FALSE |
| 6 | 0 | 20 | 0 | 12 | 9461 | -0.4135177 | -0.9767391 | 0.14970368 | FALSE |
| 4 | 0 | 10 | 0 | 7 | 9482 | -0.2006818 | -0.5418617 | 0.14049812 | FALSE |
| 12 | 524 | 4480 | 14 | 34 | 9494 | -1.8652419 | -4.3786022 | 0.64811847 | FALSE |
| 6 | 8 | 17 | 5 | 6 | 9498 | 0.21719371 | -0.3818283 | 0.81621576 | FALSE |
| 3 | 1 | 5 | 1 | 5 | 9537 | -0.0268956 | -0.3226729 | 0.26888175 | FALSE |
| 4 | 4 | 92 | 1 | 11 | 9539 | -0.171416 | -1.2896242 | 0.94679221 | FALSE |
| 7 | 47 | 448 | 6 | 23 | 9552 | -0.8168373 | -2.8060572 | 1.17238259 | FALSE |
| 6 | 2 | 6 | 2 | 4 | 9566 | 0.0508326 | -0.2246666 | 0.32633184 | FALSE |
| 1 | 0 | 39 | 0 | 6 | 9682 | -0.2218483 | -1.0995431 | 0.6558466 | FALSE |
| 1 | 0 | 11 | 0 | 5 | 9705 | -0.1426323 | -0.6286736 | 0.34340914 | FALSE |
| 2 | 0 | 1899 | 0 | 7 | 9715 | -1.0645156 | -3.7245135 | 1.59548235 | FALSE |
| 3 | 0 | 63 | 0 | 10 | 9725 | -0.3632984 | -1.3750501 | 0.64845334 | FALSE |
| 4 | 33 | 20 | 3 | 6 | 9760 | 0.15103468 | -0.7872157 | 1.08928507 | FALSE |
| 1 | 0 | 49 | 0 | 5 | 9767 | -0.2118889 | -1.1402808 | 0.716503 | FALSE |
| 5 | 495 | 45 | 7 | 5 | 9794 | 1.65731635 | -0.4963534 | 3.81098612 | FALSE |
| 2 | 38 | 0 | 5 | 0 | 9798 | 0.7603259 | -0.0123867 | 1.53303848 | FALSE |
| 3 | 1 | 537 | 1 | 7 | 9805 | -0.5375339 | -2.5896958 | 1.51462801 | FALSE |
| 2 | 7 | 20 | 2 | 5 | 9806 | 0.24288848 | -0.6538414 | 1.13961838 | FALSE |
| 2 | 0 | 79 | 0 | 6 | 9814 | -0.1831528 | -1.2455888 | 0.87928328 | FALSE |
| 2 | 1 | 9 | 1 | 4 | 9815 | -0.0302291 | -0.4805293 | 0.4200711 | FALSE |
| 1 | 0 | 23 | 0 | 6 | 9817 | -0.2023881 | -0.9132809 | 0.50850474 | FALSE |
| 2 | 1 | 123 | 1 | 6 | 9830 | -0.196027 | -1.5448743 | 1.1528202 | FALSE |
| 5 | 7 | 2 | 3 | 2 | 9836 | 0.29516036 | -0.0011409 | 0.59146161 | FALSE |
| 1 | 0 | 42 | 0 | 5 | 9853 | -0.261768 | -1.1896549 | 0.66611885 | FALSE |
| 5 | 25 | 18 | 6 | 3 | 9857 | 0.55426906 | -0.2603194 | 1.36885749 | FALSE |
| 1 | 0 | 101 | 0 | 6 | 9867 | -0.4175539 | -1.6214503 | 0.78634251 | FALSE |
| 1 | 0 | 20 | 0 | 5 | 9874 | -0.1421235 | -0.7965685 | 0.51232156 | FALSE |
| 3 | 2 | 16 | 1 | 5 | 9960 | -0.1753222 | -0.7219238 | 0.37127935 | FALSE |
| 2 | 3 | 23 | 1 | 5 | 10053 | 0.03283413 | -0.6604246 | 0.72609281 | FALSE |
| 2 | 0 | 40 | 0 | 6 | 10175 | -0.1834355 | -1.0164391 | 0.649568 | FALSE |
| 7 | 123 | 96 | 7 | 12 | 10328 | 0.67376639 | -0.9259902 | 2.27352298 | FALSE |
| 8 | 6 | 78 | 4 | 17 | 10341 | -0.3470736 | -1.3375221 | 0.64337486 | FALSE |
| 3 | 39 | 108 | 4 | 6 | 10373 | 0.29545196 | -1.2313315 | 1.82223539 | FALSE |
| 2 | 2 | 21 | 1 | 6 | 10389 | 0.16207016 | -0.6460023 | 0.97014258 | FALSE |
| 7 | 7 | 28 | 3 | 11 | 10490 | -0.1517718 | -0.765407 | 0.46186335 | FALSE |
| 5 | 1 | 6 | 1 | 5 | 10499 | -0.0418314 | -0.2747335 | 0.19107077 | FALSE |
| 5 | 1 | 7 | 1 | 7 | 10503 | -0.085999 | -0.3586117 | 0.18661379 | FALSE |
| 7 | 8 | 19 | 5 | 9 | 10515 | 0.03561063 | -0.4838982 | 0.55511949 | FALSE |
| 3 | 0 | 7 | 0 | 5 | 10522 | -0.2424479 | -0.5015317 | 0.016636 | FALSE |
| 6 | 95 | 97 | 6 | 11 | 10549 | 0.63037139 | -1.1477675 | 2.4085103 | FALSE |
| 1 | 0 | 114 | 0 | 5 | 10669 | -0.4296236 | -1.7570538 | 0.89780667 | FALSE |
| 2 | 0 | 11 | 0 | 5 | 10714 | -0.0907117 | -0.5367211 | 0.35529774 | FALSE |
| 4 | 8 | 10 | 3 | 5 | 10716 | 0.30969572 | -0.3166749 | 0.93606636 | FALSE |
| 4 | 4 | 10 | 3 | 5 | 10717 | 0.1034208 | -0.4469316 | 0.65377324 | FALSE |
| 5 | 16 | 14 | 4 | 6 | 10718 | 0.39766989 | -0.2920573 | 1.08739708 | FALSE |
| 2 | 18 | 2 | 3 | 2 | 10742 | 0.41340744 | -0.2223244 | 1.04913924 | FALSE |
| 8 | 27 | 133 | 6 | 20 | 10770 | -0.264044 | -1.4662566 | 0.93816861 | FALSE |
| 7 | 8 | 28 | 4 | 8 | 10771 | 0.00716475 | -0.612328 | 0.62665746 | FALSE |
| 2 | 0 | 14 | 0 | 6 | 10787 | -0.2393203 | -0.7619117 | 0.28327105 | FALSE |
| 4 | 2 | 16 | 2 | 6 | 10791 | -0.042451 | -0.5559568 | 0.47105472 | FALSE |
| 4 | 7 | 30 | 2 | 6 | 10808 | 0.01893638 | -0.6954925 | 0.73336524 | FALSE |
| 2 | 0 | 23 | 0 | 6 | 10825 | -0.4042736 | -1.3503157 | 0.54176837 | FALSE |
| 3 | 18 | 712 | 1 | 9 | 10836 | -0.4615563 | -2.8240966 | 1.90098394 | FALSE |
| 1 | 0 | 13 | 0 | 5 | 10853 | -0.231504 | -0.7694508 | 0.30644284 | FALSE |
| 6 | 0 | 163 | 0 | 15 | 10854 | -1.0354535 | -2.658479 | 0.58757189 | FALSE |
| 3 | 0 | 11 | 0 | 5 | 10927 | -0.3156997 | -0.9760252 | 0.34462582 | FALSE |
| 5 | 1 | 19 | 1 | 8 | 10945 | -0.2093871 | -0.7153751 | 0.29660101 | FALSE |
| 6 | 7 | 71 | 4 | 13 | 10956 | -0.2102552 | -1.2316108 | 0.81110047 | FALSE |
| 6 | 23 | 672 | 5 | 16 | 10958 | -0.5132814 | -2.8161082 | 1.78954533 | FALSE |
| 4 | 0 | 69 | 0 | 14 | 10995 | -0.6920757 | -1.6669231 | 0.28277166 | FALSE |
| 4 | 8 | 8 | 3 | 5 | 10996 | 0.10075956 | -0.5111364 | 0.71265553 | FALSE |
| 6 | 23 | 167 | 5 | 14 | 11016 | -0.6917436 | -2.417449 | 1.03396177 | FALSE |
| 4 | 6 | 28 | 2 | 8 | 11057 | -0.0189175 | -1.0493525 | 1.01151738 | FALSE |
| 3 | 0 | 7 | 0 | 5 | 11060 | -0.1717492 | -0.5089222 | 0.16542386 | FALSE |
| 4 | 10 | 32 | 3 | 9 | 11082 | -0.2405972 | -1.2719739 | 0.79077956 | FALSE |
| 4 | 11 | 15 | 4 | 4 | 11083 | 0.19637689 | -0.5522392 | 0.944993 | FALSE |
| 4 | 12 | 27 | 3 | 9 | 11086 | -0.0499036 | -0.9717977 | 0.87199043 | FALSE |
| 10 | 48 | 1204 | 6 | 30 | 11103 | -2.2305995 | -4.5257691 | 0.06457015 | FALSE |
| 3 | 9 | 15 | 2 | 5 | 11115 | -0.0726397 | -0.8251195 | 0.67984011 | FALSE |
| 4 | 11 | 4 | 2 | 3 | 11132 | 0.32703602 | -0.2338195 | 0.88789149 | FALSE |
| 1 | 0 | 23 | 0 | 5 | 11149 | -0.1694498 | -0.8887209 | 0.54982143 | FALSE |
| 1 | 0 | 50 | 0 | 6 | 11174 | -0.3753553 | -1.3730252 | 0.62231468 | FALSE |
| 1 | 0 | 330 | 0 | 6 | 11180 | -0.7387345 | -2.5800642 | 1.10259532 | FALSE |
| 2 | 0 | 15 | 0 | 6 | 11189 | -0.173185 | -0.7467925 | 0.4004224 | FALSE |
| 4 | 1 | 44 | 1 | 9 | 11191 | -0.390247 | -1.2462911 | 0.46579716 | FALSE |
| 1 | 0 | 78 | 0 | 6 | 11226 | -0.3098361 | -1.4887297 | 0.86905746 | FALSE |
| 1 | 0 | 25 | 0 | 6 | 11228 | -0.2179482 | -0.9428476 | 0.50695117 | FALSE |
| 2 | 1 | 864 | 1 | 6 | 11229 | -0.5668832 | -2.809569 | 1.67580248 | FALSE |
| 6 | 117 | 355 | 5 | 12 | 11232 | -0.0498515 | -2.3801646 | 2.28046166 | FALSE |
| 3 | 5 | 214 | 1 | 6 | 11234 | -0.0508114 | -1.6503545 | 1.54873162 | FALSE |
| 2 | 5 | 21 | 2 | 4 | 11235 | -0.0477106 | -0.8416701 | 0.74624888 | FALSE |
| 2 | 1 | 11 | 1 | 5 | 11240 | -0.0119699 | -0.5382452 | 0.51430535 | FALSE |
| 2 | 5 | 49 | 1 | 6 | 11263 | -0.2192232 | -1.3419535 | 0.90350706 | FALSE |
| 2 | 5 | 11 | 1 | 4 | 11279 | 0.19826492 | -0.4026926 | 0.79922244 | FALSE |
| 2 | 7 | 88 | 1 | 6 | 11285 | 0.04905604 | -1.2630434 | 1.36115552 | FALSE |
| 1 | 0 | 33 | 0 | 5 | 11287 | -0.1478426 | -0.9819466 | 0.68626127 | FALSE |
| 2 | 0 | 103 | 0 | 7 | 11305 | -0.4273809 | -1.6661716 | 0.8114098 | FALSE |
| 1 | 0 | 131 | 0 | 6 | 11306 | -0.6233084 | -2.0996147 | 0.8529979 | FALSE |
| 1 | 0 | 57 | 0 | 6 | 11313 | -0.4920802 | -1.5050449 | 0.52088445 | FALSE |
| 2 | 3 | 21 | 2 | 3 | 11324 | -0.1663454 | -0.7455057 | 0.41281498 | FALSE |
| 3 | 17 | 4 | 3 | 2 | 11340 | 0.5020171 | -0.1813768 | 1.18541104 | FALSE |
| 2 | 0 | 10 | 0 | 5 | 11349 | -0.1440402 | -0.5641147 | 0.27603436 | FALSE |
| 3 | 2 | 5 | 1 | 4 | 11394 | -0.0293484 | -0.3430562 | 0.28435948 | FALSE |
| 5 | 1 | 11 | 1 | 9 | 11498 | -0.1770026 | -0.5847158 | 0.23071048 | FALSE |
| 2 | 27 | 26 | 4 | 5 | 11526 | 0.42553637 | -0.6349128 | 1.48598552 | FALSE |
| 10 | 177 | 338 | 13 | 25 | 11865 | 0.50150379 | -1.2993731 | 2.30238069 | FALSE |
| 5 | 10 | 12 | 4 | 5 | 11868 | 0.13850143 | -0.5182756 | 0.79527842 | FALSE |
| 10 | 35 | 240 | 7 | 20 | 11919 | -0.1996363 | -1.5824219 | 1.18314936 | FALSE |
| 4 | 9 | 4 | 3 | 4 | 12053 | 0.35020253 | -0.0235501 | 0.72395516 | FALSE |
| 5 | 1 | 6 | 1 | 5 | 12085 | -0.1054029 | -0.4007156 | 0.18990976 | FALSE |
| 2 | 0 | 10 | 0 | 5 | 12099 | -0.3119602 | -0.7420871 | 0.11816668 | FALSE |
| 3 | 0 | 10 | 0 | 5 | 12109 | -0.290822 | -0.8594535 | 0.27780962 | FALSE |
| 9 | 7 | 16 | 5 | 11 | 12112 | 0.13712485 | -0.3725358 | 0.64678554 | FALSE |
| 6 | 7 | 63 | 4 | 13 | 12369 | -0.5100758 | -1.748068 | 0.72791641 | FALSE |
| 2 | 31 | 35 | 4 | 6 | 12442 | 0.28567231 | -0.8949055 | 1.46625009 | FALSE |
| 8 | 16 | 57 | 5 | 14 | 12446 | -0.0753162 | -0.9512691 | 0.80063671 | FALSE |
| 2 | 0 | 88 | 0 | 6 | 12458 | -0.5868389 | -1.9344734 | 0.76079559 | FALSE |
| 2 | 0 | 14 | 0 | 6 | 12481 | -0.0883149 | -0.6178246 | 0.44119491 | FALSE |
| 3 | 1 | 11 | 1 | 5 | 12482 | -0.2909665 | -0.7432631 | 0.16133011 | FALSE |
| 2 | 6 | 3 | 3 | 2 | 12484 | 0.10451225 | -0.3173025 | 0.52632699 | FALSE |
| 3 | 1 | 7 | 1 | 4 | 12504 | -0.0646682 | -0.4069509 | 0.27761438 | FALSE |
| 2 | 0 | 13 | 0 | 6 | 12511 | -0.308393 | -0.828654 | 0.21186802 | FALSE |
| 2 | 0 | 9 | 0 | 5 | 12531 | -0.1529858 | -0.5610524 | 0.25508083 | FALSE |
| 3 | 3 | 44 | 1 | 6 | 12532 | -0.2347071 | -1.142209 | 0.67279471 | FALSE |
| 2 | 0 | 41 | 0 | 6 | 12536 | -0.6036479 | -1.7305881 | 0.52329228 | FALSE |
| 4 | 0 | 67 | 0 | 6 | 12537 | -0.5132052 | -1.2980143 | 0.27160393 | FALSE |
| 3 | 1 | 33 | 1 | 7 | 12547 | -0.3374784 | -1.295388 | 0.62043112 | FALSE |
| 3 | 0 | 18 | 0 | 5 | 12549 | -0.253058 | -0.7269523 | 0.2208362 | FALSE |
| 4 | 0 | 8 | 0 | 7 | 12578 | -0.1969679 | -0.5406069 | 0.14667112 | FALSE |
| 2 | 0 | 36 | 0 | 7 | 12580 | -0.5598946 | -1.6221025 | 0.50231335 | FALSE |
| 4 | 3 | 7 | 3 | 4 | 12605 | 0.12903129 | -0.2700673 | 0.52812991 | FALSE |
| 2 | 0 | 16 | 0 | 7 | 12615 | -0.0954545 | -0.5975222 | 0.40661325 | FALSE |
| 2 | 117 | 6 | 3 | 3 | 12668 | 0.84508245 | -0.8914886 | 2.58165354 | FALSE |
| 3 | 4 | 216 | 3 | 5 | 12685 | 0.16079168 | -1.0756495 | 1.39723289 | FALSE |
| 4 | 4 | 101 | 2 | 8 | 12691 | -0.0209361 | -1.0625017 | 1.02062941 | FALSE |
| 3 | 0 | 17 | 0 | 8 | 12732 | -0.2203642 | -0.7397295 | 0.29900112 | FALSE |
| 3 | 2 | 70 | 1 | 8 | 12737 | -0.4377503 | -1.7871701 | 0.91166948 | FALSE |
| 3 | 0 | 14 | 0 | 6 | 12745 | -0.2420862 | -0.7443741 | 0.26020168 | FALSE |
| 4 | 1 | 29 | 1 | 7 | 12765 | -0.3379187 | -0.9753677 | 0.29953028 | FALSE |
| 4 | 16 | 101 | 3 | 9 | 12791 | -0.4203524 | -1.921068 | 1.0803632 | FALSE |
| 5 | 6 | 6 | 3 | 5 | 12792 | 0.19114329 | -0.1490561 | 0.53134265 | FALSE |
| 2 | 0 | 11 | 0 | 5 | 12817 | -0.0746151 | -0.5316788 | 0.38244869 | FALSE |
| 4 | 3 | 56 | 2 | 8 | 12838 | -0.4314886 | -1.6316773 | 0.76870018 | FALSE |
| 4 | 5 | 2 | 5 | 2 | 12860 | 0.22310051 | -0.1124703 | 0.55867128 | FALSE |
| 5 | 15 | 476 | 1 | 6 | 12904 | -0.7709381 | -2.0095525 | 0.46767629 | FALSE |
| 4 | 15 | 8 | 3 | 5 | 12910 | 0.31075161 | -0.359207 | 0.9807102 | FALSE |
| 5 | 0 | 17 | 0 | 9 | 12919 | -0.3154212 | -0.7656738 | 0.13483144 | FALSE |
| 5 | 6 | 8 | 3 | 7 | 12971 | 0.07795216 | -0.3745156 | 0.53041988 | FALSE |
| 3 | 1 | 6 | 1 | 4 | 12984 | 0.0322563 | -0.2780056 | 0.34251822 | FALSE |
| 5 | 3 | 5 | 2 | 4 | 12986 | 0.04125434 | -0.2279532 | 0.31046187 | FALSE |
| 6 | 51 | 116 | 8 | 11 | 12987 | 0.64519657 | -0.7921416 | 2.08253475 | FALSE |
| 1 | 0 | 10 | 0 | 6 | 12991 | -0.1529673 | -0.6178868 | 0.31195226 | FALSE |
| 1 | 0 | 13 | 0 | 6 | 12995 | -0.1707423 | -0.7196599 | 0.37817525 | FALSE |
| 1 | 0 | 39 | 0 | 6 | 13001 | -0.3711739 | -1.3208824 | 0.57853459 | FALSE |
| 1 | 0 | 21 | 0 | 5 | 13018 | -0.1521545 | -0.7896433 | 0.48533434 | FALSE |
| 1 | 0 | 9 | 0 | 5 | 13047 | -0.2234572 | -0.6587488 | 0.21183446 | FALSE |
| 1 | 0 | 9 | 0 | 5 | 13053 | -0.1497838 | -0.581273 | 0.28170537 | FALSE |
| 1 | 0 | 13 | 0 | 5 | 13055 | -0.2021568 | -0.7523048 | 0.34799109 | FALSE |
| 2 | 0 | 213 | 0 | 7 | 13056 | -0.7350475 | -2.3793599 | 0.909265 | FALSE |
| 1 | 0 | 19 | 0 | 5 | 13063 | -0.2117609 | -0.8490347 | 0.42551298 | FALSE |
| 3 | 0 | 89 | 0 | 8 | 13138 | -0.5875264 | -1.47834 | 0.30328712 | FALSE |
| 3 | 0 | 123 | 0 | 8 | 13142 | -0.7226709 | -1.8557626 | 0.41042075 | FALSE |
| 5 | 11 | 20 | 4 | 11 | 13153 | -0.0110826 | -0.7337116 | 0.71154634 | FALSE |
| 5 | 57 | 189 | 6 | 10 | 13154 | 0.16643912 | -1.7344805 | 2.06735869 | FALSE |
| 1 | 0 | 25 | 0 | 5 | 13155 | -0.2609079 | -1.0104196 | 0.48860378 | FALSE |
| 1 | 0 | 31 | 0 | 5 | 13158 | -0.1675457 | -0.9706562 | 0.63556471 | FALSE |
| 6 | 176 | 269 | 9 | 14 | 13160 | 0.78869615 | -1.3692365 | 2.94662884 | FALSE |
| 3 | 3 | 323 | 1 | 7 | 13161 | -0.4191085 | -2.2217492 | 1.3835322 | FALSE |
| 7 | 66 | 1108 | 6 | 16 | 13164 | -0.9418035 | -3.7294488 | 1.84584188 | FALSE |
| 1 | 0 | 18 | 0 | 5 | 13166 | -0.1751374 | -0.8066822 | 0.45640743 | FALSE |
| 2 | 2 | 60 | 1 | 5 | 13170 | -0.0164466 | -1.0750189 | 1.04212559 | FALSE |
| 2 | 5 | 35 | 1 | 4 | 13172 | -0.2232626 | -1.1022422 | 0.65571707 | FALSE |
| 1 | 0 | 19 | 0 | 5 | 13180 | -0.1421219 | -0.7651675 | 0.48092373 | FALSE |
| 6 | 48 | 216 | 8 | 11 | 13194 | 0.28532205 | -1.4765524 | 2.04719645 | FALSE |
| 6 | 68 | 56 | 9 | 9 | 13225 | 1.02114333 | -0.4601846 | 2.50247123 | FALSE |
| 2 | 14 | 289 | 2 | 6 | 13244 | 0.15026926 | -1.7302591 | 2.03079766 | FALSE |
| 1 | 0 | 27 | 0 | 5 | 13246 | -0.2004035 | -0.9621269 | 0.56131988 | FALSE |
| 1 | 0 | 75 | 0 | 6 | 13250 | -0.3399623 | -1.4970632 | 0.81713866 | FALSE |
| 2 | 3 | 26 | 1 | 5 | 13262 | 0.0386425 | -0.7654571 | 0.84274207 | FALSE |
| 2 | 1 | 38 | 1 | 4 | 13265 | 0.00727994 | -0.8517433 | 0.8663032 | FALSE |
| 1 | 0 | 35 | 0 | 5 | 13271 | -0.2347588 | -1.0610103 | 0.59149268 | FALSE |
| 2 | 9 | 331 | 1 | 6 | 13302 | -0.3504863 | -2.2533524 | 1.55237984 | FALSE |
| 1 | 0 | 203 | 0 | 6 | 13321 | -0.5159211 | -2.1031392 | 1.07129701 | FALSE |
| 3 | 1 | 785 | 1 | 9 | 13322 | -0.958639 | -3.0910973 | 1.1738192 | FALSE |
| 1 | 0 | 130 | 0 | 6 | 13331 | -0.5317635 | -1.8327783 | 0.76925125 | FALSE |
| 1 | 0 | 15 | 0 | 5 | 13335 | -0.1291394 | -0.7020122 | 0.4437334 | FALSE |
| 1 | 0 | 37 | 0 | 5 | 13337 | -0.2690493 | -1.1285742 | 0.59047561 | FALSE |
| 2 | 7 | 67 | 2 | 4 | 13354 | -0.1894604 | -1.2258078 | 0.84688708 | FALSE |
| 2 | 16 | 24 | 1 | 5 | 13355 | 0.01091261 | -0.8204121 | 0.84223732 | FALSE |
| 1 | 0 | 7 | 0 | 5 | 13369 | -0.0928859 | -0.4678638 | 0.28209211 | FALSE |
| 1 | 0 | 13 | 0 | 5 | 13375 | -0.1240407 | -0.6502988 | 0.4022174 | FALSE |
| 1 | 0 | 72 | 0 | 5 | 13382 | -0.4428731 | -1.4932033 | 0.6074571 | FALSE |
| 2 | 0 | 16 | 0 | 6 | 13513 | -0.4100537 | -1.0730453 | 0.25293789 | FALSE |
| 6 | 266 | 442 | 7 | 15 | 13594 | 0.16057001 | -2.3438117 | 2.66495172 | FALSE |
| 3 | 1 | 6 | 1 | 5 | 14002 | -0.093926 | -0.4394068 | 0.25155477 | FALSE |
| 3 | 1 | 5 | 1 | 4 | 14037 | 8.78E-05 | -0.2582812 | 0.25845687 | FALSE |
| 5 | 0 | 6 | 0 | 5 | 14061 | -0.1688334 | -0.4026869 | 0.06502021 | FALSE |
| 6 | 7 | 31 | 4 | 10 | 14095 | 0.34785861 | -0.5581911 | 1.25390833 | FALSE |
| 11 | 21 | 141 | 7 | 20 | 14109 | -0.0810799 | -1.4857664 | 1.32360652 | FALSE |
| 8 | 9 | 23 | 8 | 8 | 14118 | 0.26805341 | -0.345081 | 0.88118784 | FALSE |
| 4 | 5 | 5 | 3 | 2 | 14172 | 0.25540838 | -0.1153899 | 0.62620669 | FALSE |
| 2 | 1 | 34 | 1 | 5 | 14254 | -0.325718 | -1.2039549 | 0.55251888 | FALSE |
| 7 | 10 | 24 | 3 | 12 | 14258 | 0.08327897 | -0.5252567 | 0.69181468 | FALSE |
| 5 | 1 | 6 | 1 | 6 | 14280 | 0.00254195 | -0.2282597 | 0.23334357 | FALSE |
| 5 | 2 | 6 | 2 | 6 | 14284 | 0.07326464 | -0.2515436 | 0.39807293 | FALSE |
| 4 | 2 | 11 | 2 | 4 | 14398 | -0.1846253 | -0.8585151 | 0.48926458 | FALSE |
| 6 | 19 | 133 | 4 | 10 | 14399 | -0.2850284 | -1.6990607 | 1.12900386 | FALSE |
| 3 | 4 | 6 | 3 | 3 | 14480 | 0.03258315 | -0.3684202 | 0.43358652 | FALSE |
| 2 | 0 | 6 | 0 | 6 | 14592 | -0.1098885 | -0.3770117 | 0.15723479 | FALSE |
| 8 | 9 | 38 | 6 | 11 | 14706 | -0.314716 | -1.3072938 | 0.67786177 | FALSE |
| 3 | 6 | 11 | 3 | 3 | 14709 | 0.14792686 | -0.3778329 | 0.67368661 | FALSE |
| 3 | 1 | 5 | 1 | 4 | 14777 | -0.0965465 | -0.358468 | 0.16537496 | FALSE |
| 3 | 27 | 167 | 3 | 4 | 14794 | -0.2411997 | -1.3644745 | 0.88207518 | FALSE |
| 3 | 6 | 134 | 1 | 7 | 14798 | -0.5720648 | -1.9905218 | 0.84639221 | FALSE |
| 3 | 6 | 67 | 2 | 8 | 14799 | -0.5837782 | -1.7956991 | 0.62814261 | FALSE |
| 1 | 0 | 39 | 0 | 5 | 14800 | -0.3704797 | -1.3497414 | 0.60878205 | FALSE |
| 4 | 3 | 9 | 1 | 5 | 14804 | -0.0037665 | -0.399276 | 0.39174298 | FALSE |
| 6 | 2 | 25 | 1 | 14 | 14808 | -0.5304063 | -1.1558147 | 0.095002 | FALSE |
| 3 | 1 | 11 | 1 | 5 | 14855 | -0.3146706 | -0.7796411 | 0.15029985 | FALSE |
| 3 | 3 | 6 | 3 | 3 | 14869 | 0.12968667 | -0.2306361 | 0.4900094 | FALSE |
| 3 | 1 | 11 | 1 | 4 | 14884 | -0.0902034 | -0.5202578 | 0.33985095 | FALSE |
| 2 | 0 | 9 | 0 | 5 | 14885 | -0.2702456 | -0.6950184 | 0.15452727 | FALSE |
| 3 | 1 | 12 | 1 | 5 | 14902 | -0.1338424 | -0.6447363 | 0.37705158 | FALSE |
| 4 | 0 | 8 | 0 | 6 | 14957 | -0.1721262 | -0.4709293 | 0.1266768 | FALSE |
| 4 | 0 | 42 | 0 | 9 | 14975 | -0.6382296 | -1.6020894 | 0.32563011 | FALSE |
| 4 | 0 | 13 | 0 | 8 | 14994 | -0.1928721 | -0.6284429 | 0.24269865 | FALSE |
| 5 | 3 | 18 | 1 | 6 | 15029 | -0.1741604 | -0.777427 | 0.4291063 | FALSE |
| 3 | 1 | 115 | 1 | 7 | 15034 | -0.8120868 | -2.3315364 | 0.70736284 | FALSE |
| 7 | 2 | 48 | 2 | 13 | 15045 | -0.6338046 | -1.6827243 | 0.41511503 | FALSE |
| 9 | 16 | 164 | 5 | 23 | 15047 | -0.8738592 | -2.3557483 | 0.60802983 | FALSE |
| 3 | 0 | 166 | 0 | 10 | 15064 | -1.2266013 | -3.0830798 | 0.62987724 | FALSE |
| 5 | 15 | 36 | 5 | 12 | 15073 | -0.1220245 | -1.0994786 | 0.85542959 | FALSE |
| 1 | 0 | 21 | 0 | 5 | 15076 | -0.2357126 | -0.8675071 | 0.39608192 | FALSE |
| 3 | 1 | 43 | 1 | 7 | 15093 | -0.5417884 | -1.6441441 | 0.5605673 | FALSE |
| 5 | 1 | 26 | 1 | 13 | 15099 | -0.2850648 | -0.8985182 | 0.3283886 | FALSE |
| 3 | 18 | 3 | 6 | 2 | 15112 | 0.55383607 | -0.2592731 | 1.36694527 | FALSE |
| 3 | 0 | 9 | 0 | 8 | 15131 | -0.3354572 | -0.6876835 | 0.01676921 | FALSE |
| 1 | 0 | 19 | 0 | 5 | 15132 | -0.2208109 | -0.8544926 | 0.4128709 | FALSE |
| 5 | 1 | 68 | 1 | 12 | 15143 | -0.4315491 | -1.4612309 | 0.59813263 | FALSE |
| 5 | 14 | 26 | 2 | 13 | 15149 | 0.00979902 | -0.7820395 | 0.80163756 | FALSE |
| 2 | 0 | 116 | 0 | 7 | 15159 | -0.5858144 | -1.7360956 | 0.56446683 | FALSE |
| 5 | 3 | 5 | 1 | 5 | 15233 | 0.08248335 | -0.1872901 | 0.35225677 | FALSE |
| 5 | 2 | 7 | 2 | 6 | 15263 | -0.1656074 | -0.4780834 | 0.1468686 | FALSE |
| 4 | 85 | 72 | 6 | 7 | 15308 | 0.5863125 | -1.2944838 | 2.46710881 | FALSE |
| 7 | 5 | 10 | 2 | 9 | 15313 | -0.0468175 | -0.3808206 | 0.28718555 | FALSE |
| 6 | 4 | 8 | 3 | 6 | 15340 | 0.11936834 | -0.204405 | 0.44314172 | FALSE |
| 5 | 38 | 6 | 4 | 5 | 15389 | 0.43525947 | -0.6521729 | 1.52269186 | FALSE |
| 3 | 1 | 8 | 1 | 5 | 15395 | -0.1003878 | -0.4398871 | 0.23911154 | FALSE |
| 2 | 1 | 7 | 1 | 4 | 15411 | 0.01151956 | -0.382988 | 0.4060271 | FALSE |
| 4 | 1 | 10 | 1 | 6 | 15417 | -0.0259055 | -0.412217 | 0.36040602 | FALSE |
| 5 | 0 | 39 | 0 | 11 | 15440 | -0.7293478 | -1.6182639 | 0.15956831 | FALSE |
| 1 | 0 | 9 | 0 | 5 | 15452 | -0.1912768 | -0.6313941 | 0.24884047 | FALSE |
| 4 | 3 | 14 | 2 | 4 | 15473 | 0.09755295 | -0.4382368 | 0.63334269 | FALSE |
| 2 | 0 | 8 | 0 | 5 | 15475 | -0.1302874 | -0.5120401 | 0.25146532 | FALSE |
| 5 | 3 | 10 | 2 | 5 | 15477 | -0.0214067 | -0.4147465 | 0.37193321 | FALSE |
| 4 | 5 | 3 | 3 | 2 | 15540 | 0.23942673 | -0.0721929 | 0.55104633 | FALSE |
| 5 | 3 | 3 | 2 | 3 | 15565 | 0.08816968 | -0.1359809 | 0.31232025 | FALSE |
| 1 | 0 | 8 | 0 | 5 | 15569 | -0.1125677 | -0.5224231 | 0.29728769 | FALSE |
| 1 | 0 | 23 | 0 | 5 | 15572 | -0.1685899 | -0.8829663 | 0.54578656 | FALSE |
| 1 | 0 | 57 | 0 | 6 | 15580 | -0.232523 | -1.269771 | 0.80472505 | FALSE |
| 1 | 0 | 9 | 0 | 5 | 15613 | -0.148956 | -0.5922931 | 0.29438111 | FALSE |
| 1 | 0 | 11 | 0 | 6 | 15617 | -0.1980229 | -0.6993643 | 0.30331845 | FALSE |
| 1 | 0 | 10 | 0 | 6 | 15700 | -0.1772751 | -0.649974 | 0.29542381 | FALSE |
| 1 | 0 | 17 | 0 | 5 | 15793 | -0.1622768 | -0.7789675 | 0.45441385 | FALSE |
| 4 | 78 | 11 | 7 | 2 | 15811 | 1.1808857 | -0.1262197 | 2.48799113 | FALSE |
| 1 | 0 | 7 | 0 | 5 | 15817 | -0.102668 | -0.4778436 | 0.2725077 | FALSE |
| 5 | 11 | 17 | 3 | 5 | 15829 | 0.11600476 | -0.4263414 | 0.6583509 | FALSE |
| 1 | 0 | 36 | 0 | 5 | 15832 | -0.1938527 | -1.0720734 | 0.68436793 | FALSE |
| 6 | 6 | 195 | 3 | 12 | 15837 | -0.6963296 | -2.1786236 | 0.7859644 | FALSE |
| 5 | 4 | 5 | 3 | 5 | 15843 | 0.06662389 | -0.2505345 | 0.38378224 | FALSE |
| 3 | 3 | 10 | 1 | 6 | 15848 | -0.1091324 | -0.5989807 | 0.3807159 | FALSE |
| 6 | 8 | 60 | 3 | 8 | 15853 | -0.1110723 | -1.26198 | 1.03983542 | FALSE |
| 1 | 0 | 252 | 0 | 6 | 15856 | -0.5774732 | -2.1713891 | 1.01644278 | FALSE |
| 4 | 4 | 28 | 3 | 6 | 15873 | -0.0451387 | -0.8468909 | 0.75661354 | FALSE |
| 2 | 16 | 0 | 5 | 0 | 15878 | 0.54552662 | -0.1831239 | 1.27417716 | FALSE |
| 2 | 1 | 30 | 1 | 5 | 15963 | -0.0517008 | -0.8400051 | 0.73660356 | FALSE |
| 1 | 0 | 7 | 0 | 5 | 16007 | -0.2078457 | -0.5790901 | 0.1633988 | FALSE |
| 2 | 0 | 14 | 0 | 6 | 16009 | -0.1924803 | -0.6648456 | 0.27988506 | FALSE |
| 1 | 0 | 22 | 0 | 5 | 16013 | -0.2042169 | -0.865025 | 0.45659127 | FALSE |
| 1 | 0 | 42 | 0 | 5 | 16021 | -0.3889872 | -1.2108018 | 0.43282729 | FALSE |
| 1 | 0 | 11 | 0 | 5 | 16023 | -0.1600581 | -0.6448541 | 0.32473792 | FALSE |
| 2 | 2 | 37 | 1 | 4 | 16026 | 0.00712417 | -0.8029851 | 0.81723348 | FALSE |
| 3 | 4 | 3 | 3 | 2 | 16054 | 0.13929033 | -0.1905673 | 0.46914797 | FALSE |
| 1 | 0 | 14 | 0 | 5 | 16059 | -0.1676722 | -0.7113925 | 0.37604811 | FALSE |
| 1 | 0 | 9 | 0 | 5 | 16119 | -0.1929702 | -0.6316721 | 0.24573184 | FALSE |
| 4 | 4 | 54 | 2 | 7 | 16265 | -0.261239 | -1.1804485 | 0.65797056 | FALSE |
| 4 | 0 | 10 | 0 | 7 | 16365 | -0.1870905 | -0.5685364 | 0.19435539 | FALSE |
| 2 | 1 | 6 | 1 | 4 | 16774 | 0.02244364 | -0.3288102 | 0.37369745 | FALSE |
| 3 | 0 | 16 | 0 | 7 | 16813 | -0.4257813 | -0.8965982 | 0.04503547 | FALSE |
| 4 | 3 | 6 | 3 | 4 | 16841 | 0.02590926 | -0.3196121 | 0.37143057 | FALSE |
| 6 | 4 | 326 | 1 | 19 | 16842 | -1.2402101 | -3.0846876 | 0.60426742 | FALSE |
| 5 | 2 | 65 | 2 | 11 | 16849 | -0.3271045 | -1.346986 | 0.69277707 | FALSE |
| 9 | 7 | 296 | 5 | 18 | 16887 | -0.0391876 | -1.6807188 | 1.6023436 | FALSE |
| 4 | 8 | 15 | 3 | 5 | 16930 | 0.22878938 | -0.3934481 | 0.85102689 | FALSE |
| 4 | 0 | 28 | 0 | 7 | 16941 | -0.2627027 | -0.9358662 | 0.41046082 | FALSE |
| 4 | 4 | 6 | 2 | 4 | 17054 | 0.03634852 | -0.3283405 | 0.40103749 | FALSE |
| 3 | 3 | 3 | 3 | 3 | 17069 | 0.08839113 | -0.2090492 | 0.38583147 | FALSE |
| 5 | 2 | 11 | 2 | 8 | 17168 | -0.0066447 | -0.3955049 | 0.38221549 | FALSE |
| 4 | 0 | 12 | 0 | 8 | 17188 | -0.2436259 | -0.7874726 | 0.30022086 | FALSE |
| 3 | 0 | 16 | 0 | 7 | 17198 | -0.3616306 | -1.0157417 | 0.29248058 | FALSE |
| 2 | 0 | 13 | 0 | 6 | 17202 | -0.2552809 | -0.7491356 | 0.23857386 | FALSE |
| 6 | 1 | 9 | 1 | 7 | 17206 | -0.0576918 | -0.3729895 | 0.25760595 | FALSE |
| 4 | 1 | 14 | 1 | 7 | 17213 | -0.080364 | -0.6059062 | 0.44517818 | FALSE |
| 8 | 8 | 55 | 4 | 15 | 17252 | -0.521506 | -1.6621999 | 0.61918788 | FALSE |
| 5 | 1 | 9 | 1 | 6 | 17253 | -0.0582973 | -0.359505 | 0.24291044 | FALSE |
| 3 | 0 | 17 | 0 | 8 | 17325 | -0.4050088 | -1.0371109 | 0.22709332 | FALSE |
| 10 | 187 | 970 | 12 | 18 | 17398 | 0.55801638 | -2.1128697 | 3.22890248 | FALSE |
| 5 | 11 | 15 | 6 | 6 | 17487 | 0.34968722 | -0.3337885 | 1.03316292 | FALSE |
| 6 | 1 | 28 | 1 | 10 | 17599 | -0.4665546 | -1.3322796 | 0.3991705 | FALSE |
| 3 | 8 | 2 | 4 | 1 | 17686 | 0.37636009 | -0.1459561 | 0.89867633 | FALSE |
| 4 | 3 | 3 | 3 | 3 | 17884 | 0.13883543 | -0.1481475 | 0.42581837 | FALSE |
| 3 | 0 | 7 | 0 | 5 | 17937 | -0.2339672 | -0.5081124 | 0.04017805 | FALSE |
| 2 | 10 | 9 | 2 | 3 | 17947 | 0.17931458 | -0.4148882 | 0.77351733 | FALSE |
| 3 | 2 | 4 | 2 | 3 | 17948 | 0.00435304 | -0.2681895 | 0.27689559 | FALSE |
| 3 | 7 | 132 | 2 | 7 | 17967 | -0.6462874 | -2.1990539 | 0.90647911 | FALSE |
| 2 | 0 | 37 | 0 | 6 | 17971 | -0.4899384 | -1.4376638 | 0.4577871 | FALSE |
| 6 | 137 | 54 | 8 | 10 | 18003 | 1.50228276 | -0.0201863 | 3.0247518 | FALSE |
| 3 | 0 | 6 | 0 | 6 | 18006 | -0.2325533 | -0.468623 | 0.00351645 | FALSE |
| 4 | 0 | 21 | 0 | 9 | 18041 | -0.2574922 | -0.7805199 | 0.26553552 | FALSE |
| 8 | 113 | 1039 | 7 | 26 | 18046 | -0.9350764 | -3.5581398 | 1.68798695 | FALSE |
| 9 | 64 | 403 | 11 | 24 | 18047 | -0.4402456 | -2.4340465 | 1.5535553 | FALSE |
| 3 | 1 | 13 | 1 | 6 | 18051 | -0.2805186 | -0.8264411 | 0.26540392 | FALSE |
| 7 | 49 | 64 | 9 | 7 | 18075 | 0.74516217 | -0.4077983 | 1.89812262 | FALSE |
| 3 | 3 | 171 | 1 | 8 | 18105 | -0.7239585 | -2.674984 | 1.22706693 | FALSE |
| 3 | 1 | 18 | 1 | 5 | 18109 | -0.2318232 | -0.7681464 | 0.30450003 | FALSE |
| 3 | 2 | 9 | 1 | 4 | 18115 | 0.03262153 | -0.4574054 | 0.52264846 | FALSE |
| 2 | 0 | 13 | 0 | 7 | 18118 | -0.3958749 | -0.956869 | 0.16511912 | FALSE |
| 2 | 0 | 9 | 0 | 7 | 18119 | -0.2985286 | -0.7298116 | 0.13275434 | FALSE |
| 2 | 0 | 48 | 0 | 8 | 18124 | -0.5869368 | -1.7178491 | 0.54397543 | FALSE |
| 2 | 0 | 23 | 0 | 5 | 18151 | -0.3179801 | -0.9408589 | 0.30489882 | FALSE |
| 4 | 0 | 61 | 0 | 13 | 18190 | -0.8702677 | -2.189264 | 0.4487286 | FALSE |
| 3 | 0 | 6 | 0 | 5 | 18193 | -0.2371349 | -0.5816663 | 0.10739653 | FALSE |
| 1 | 0 | 22 | 0 | 5 | 18232 | -0.3323909 | -1.10495 | 0.44016827 | FALSE |
| 2 | 0 | 18 | 0 | 6 | 18237 | -0.3241833 | -0.9140025 | 0.26563591 | FALSE |
| 2 | 0 | 9 | 0 | 6 | 18259 | -0.1984581 | -0.6451441 | 0.24822793 | FALSE |
| 4 | 2 | 7 | 2 | 4 | 18296 | -0.0378962 | -0.3718966 | 0.29610414 | FALSE |
| 2 | 0 | 10 | 0 | 6 | 18319 | -0.2979708 | -0.8255897 | 0.22964811 | FALSE |
| 3 | 1 | 7 | 1 | 5 | 18323 | -0.0682312 | -0.4312243 | 0.2947619 | FALSE |
| 4 | 0 | 7 | 0 | 6 | 18356 | -0.0889333 | -0.3852558 | 0.20738917 | FALSE |
| 2 | 0 | 49 | 0 | 7 | 18369 | -0.7104999 | -1.8138595 | 0.39285981 | FALSE |
| 6 | 2 | 14 | 2 | 9 | 18370 | -0.167661 | -0.6003789 | 0.26505682 | FALSE |
| 4 | 10 | 18 | 2 | 5 | 18372 | 0.18435489 | -0.4182491 | 0.78695894 | FALSE |
| 3 | 0 | 10 | 0 | 7 | 18375 | -0.2726116 | -0.6331142 | 0.08789101 | FALSE |
| 3 | 6 | 2 | 3 | 2 | 18379 | 0.25647573 | -0.1370474 | 0.64999891 | FALSE |
| 3 | 23 | 10 | 4 | 4 | 18409 | 0.48397629 | -0.5707958 | 1.53874838 | FALSE |
| 5 | 4 | 8 | 1 | 7 | 18455 | -0.064313 | -0.4580206 | 0.3293947 | FALSE |
| 3 | 1 | 14 | 1 | 5 | 18464 | -0.0369126 | -0.5376963 | 0.46387115 | FALSE |
| 3 | 0 | 9 | 0 | 6 | 18655 | -0.1668706 | -0.5177807 | 0.18403959 | FALSE |
| 5 | 1 | 29 | 1 | 10 | 18657 | -0.2661318 | -0.993379 | 0.46111534 | FALSE |
| 6 | 13 | 43 | 5 | 12 | 18690 | -0.2386432 | -1.2140903 | 0.73680389 | FALSE |
| 5 | 4 | 10 | 4 | 4 | 18709 | 0.07284605 | -0.3124374 | 0.45812949 | FALSE |
| 9 | 50 | 772 | 10 | 29 | 18746 | -1.4085159 | -3.3636053 | 0.5465734 | FALSE |
| 3 | 2 | 7 | 2 | 4 | 18749 | -0.0179983 | -0.4149211 | 0.37892452 | FALSE |
| 4 | 4 | 6 | 4 | 5 | 18766 | 0.06789971 | -0.3076635 | 0.44346292 | FALSE |
| 4 | 0 | 14 | 0 | 11 | 18800 | -0.3992235 | -0.8856426 | 0.08719565 | FALSE |
| 4 | 2 | 12 | 2 | 6 | 18848 | -0.2386809 | -0.7105947 | 0.2332329 | FALSE |
| 6 | 4 | 98 | 1 | 18 | 18855 | -1.0874855 | -2.3255914 | 0.15062047 | FALSE |
| 3 | 1 | 7 | 1 | 4 | 18876 | -0.1559867 | -0.5707276 | 0.25875417 | FALSE |
| 2 | 0 | 15 | 0 | 5 | 18885 | -0.4083652 | -0.9623977 | 0.14566729 | FALSE |
| 1 | 0 | 34 | 0 | 6 | 18890 | -0.344179 | -1.2196481 | 0.53129009 | FALSE |
| 11 | 521 | 4390 | 7 | 33 | 18924 | -2.1411489 | -4.7691866 | 0.48688873 | FALSE |
| 3 | 3 | 49 | 1 | 6 | 18992 | -0.2339652 | -1.2228484 | 0.75491794 | FALSE |
| 1 | 0 | 34 | 0 | 5 | 19042 | -0.3653004 | -1.3192959 | 0.58869499 | FALSE |
| 7 | 3 | 60 | 2 | 10 | 19046 | -0.29681 | -1.0392236 | 0.44560348 | FALSE |
| 5 | 0 | 24 | 0 | 10 | 19051 | -0.6768323 | -1.3661435 | 0.01247895 | FALSE |
| 4 | 2 | 8 | 2 | 3 | 19085 | 0.05477646 | -0.2808729 | 0.39042583 | FALSE |
| 1 | 0 | 19 | 0 | 5 | 19315 | -0.2455758 | -0.8938917 | 0.40274002 | FALSE |
| 1 | 0 | 73 | 0 | 5 | 19360 | -0.2657902 | -1.3998278 | 0.86824752 | FALSE |
| 1 | 0 | 22 | 0 | 5 | 19479 | -0.1593548 | -0.8283139 | 0.50960426 | FALSE |
| 4 | 17 | 4 | 4 | 3 | 19498 | 0.47754916 | -0.2144845 | 1.16958279 | FALSE |
| 1 | 0 | 40 | 0 | 5 | 19527 | -0.2150287 | -1.0500558 | 0.61999845 | FALSE |
| 1 | 0 | 30 | 0 | 6 | 19553 | -0.2383946 | -1.0723533 | 0.59556412 | FALSE |
| 4 | 7 | 12 | 3 | 3 | 19567 | 0.34717052 | -0.2414804 | 0.93582149 | FALSE |
| 1 | 0 | 16 | 0 | 6 | 19588 | -0.2303437 | -0.8262922 | 0.36560473 | FALSE |
| 4 | 4 | 23 | 2 | 4 | 19614 | -0.1505785 | -0.9325879 | 0.63143085 | FALSE |
| 3 | 39 | 4 | 3 | 3 | 19625 | 0.56166142 | -0.4997749 | 1.62309776 | FALSE |
| 1 | 0 | 18 | 0 | 5 | 19627 | -0.210204 | -0.8387915 | 0.41838359 | FALSE |
| 1 | 0 | 35 | 0 | 6 | 19727 | -0.2131047 | -1.0774513 | 0.651242 | FALSE |
| 1 | 0 | 17 | 0 | 5 | 19768 | -0.1343364 | -0.7075084 | 0.43883556 | FALSE |
| 1 | 0 | 14 | 0 | 6 | 19824 | -0.1774894 | -0.7463304 | 0.39135158 | FALSE |
| 3 | 1 | 16 | 1 | 6 | 19843 | -0.1881161 | -0.7443875 | 0.36815539 | FALSE |
| 1 | 0 | 10 | 0 | 5 | 19867 | -0.1487621 | -0.6086192 | 0.31109498 | FALSE |
| 2 | 0 | 7 | 0 | 6 | 19872 | -0.1592694 | -0.5079286 | 0.18938979 | FALSE |
| 1 | 0 | 56 | 0 | 5 | 19910 | -0.3788914 | -1.3454333 | 0.58765043 | FALSE |
| 1 | 0 | 11 | 0 | 5 | 19944 | -0.1988522 | -0.6931205 | 0.29541598 | FALSE |
| 2 | 2 | 20 | 1 | 4 | 20205 | -0.0265697 | -0.6882485 | 0.63510905 | FALSE |
| 1 | 0 | 59 | 0 | 6 | 20368 | -0.4442455 | -1.471325 | 0.5828341 | FALSE |
| 8 | 2 | 45 | 2 | 13 | 20376 | -0.437398 | -1.2267034 | 0.35190745 | FALSE |
| 1 | 0 | 13 | 0 | 5 | 20401 | -0.0889333 | -0.6113234 | 0.43345669 | FALSE |
| 2 | 4 | 25 | 2 | 4 | 20406 | 0.00728483 | -0.7353967 | 0.74996637 | FALSE |
| 3 | 3 | 9 | 2 | 4 | 20568 | 0.05680406 | -0.3786895 | 0.49229762 | FALSE |
| 3 | 0 | 6 | 0 | 5 | 20586 | -0.081661 | -0.3547212 | 0.1913993 | FALSE |
| 2 | 1 | 20 | 1 | 4 | 20707 | -0.0075896 | -0.6164037 | 0.60122453 | FALSE |
| 4 | 24 | 56 | 5 | 7 | 20724 | 0.4889487 | -0.731827 | 1.70972438 | FALSE |
| 2 | 2 | 8 | 2 | 4 | 20760 | 0.07638176 | -0.3614913 | 0.51425482 | FALSE |
| 12 | 95 | 163 | 11 | 20 | 20826 | 0.37283443 | -1.0214411 | 1.76710993 | FALSE |
| 5 | 7 | 12 | 3 | 6 | 20827 | -0.032833 | -0.7229959 | 0.65732982 | FALSE |
| 4 | 5 | 38 | 4 | 5 | 20841 | -0.1855552 | -1.3231471 | 0.95203661 | FALSE |
| 3 | 5 | 4 | 3 | 3 | 20845 | 0.25306655 | -0.1212502 | 0.62738329 | FALSE |
| 2 | 2 | 8 | 1 | 4 | 20912 | 0.0628063 | -0.3743553 | 0.49996788 | FALSE |
| 7 | 27 | 38 | 8 | 9 | 20918 | 0.55020976 | -0.4747697 | 1.57518921 | FALSE |
| 6 | 7 | 14 | 4 | 5 | 20936 | 0.29854873 | -0.2817331 | 0.87883058 | FALSE |
| 12 | 53 | 288 | 10 | 26 | 20938 | -0.1415127 | -1.7291832 | 1.4461579 | FALSE |
| 12 | 271 | 727 | 13 | 35 | 20956 | 0.02923467 | -1.5583322 | 1.61680149 | FALSE |
| 9 | 91 | 205 | 10 | 19 | 20957 | 0.71544423 | -1.0948937 | 2.52578219 | FALSE |
| 2 | 3 | 4 | 2 | 3 | 20960 | 0.08265073 | -0.2535521 | 0.41885359 | FALSE |
| 2 | 21 | 6 | 3 | 2 | 20982 | 0.43400194 | -0.3699893 | 1.2379932 | FALSE |
| 9 | 36 | 164 | 9 | 20 | 20991 | 0.03420843 | -1.4054506 | 1.47386748 | FALSE |
| 2 | 41 | 0 | 5 | 0 | 21118 | 0.89524465 | -0.0420905 | 1.83257985 | FALSE |
| 4 | 13 | 11 | 5 | 6 | 21129 | 0.36434951 | -0.2325858 | 0.96128477 | FALSE |
| 1 | 0 | 23 | 0 | 5 | 21144 | -0.2169323 | -0.8797649 | 0.44590034 | FALSE |
| 1 | 0 | 19 | 0 | 5 | 21191 | -0.0995825 | -0.758045 | 0.55888006 | FALSE |
| 4 | 0 | 18 | 0 | 8 | 21249 | -0.192218 | -0.7045957 | 0.32015971 | FALSE |
| 5 | 1 | 6 | 1 | 4 | 21259 | -0.0563854 | -0.2903976 | 0.17762676 | FALSE |
| 2 | 0 | 21 | 0 | 6 | 21268 | -0.4167976 | -1.0252153 | 0.19162016 | FALSE |
| 5 | 1 | 12 | 1 | 6 | 21269 | -0.1011215 | -0.5521098 | 0.34986668 | FALSE |
| 4 | 1 | 9 | 1 | 6 | 21272 | -0.1367682 | -0.4880362 | 0.21449971 | FALSE |
| 6 | 4 | 10 | 3 | 8 | 21325 | -0.1019406 | -0.5435187 | 0.33963743 | FALSE |
| 2 | 28 | 10 | 4 | 4 | 21391 | 0.59024555 | -0.4288487 | 1.60933979 | FALSE |
| 1 | 0 | 30 | 0 | 6 | 21435 | -0.2162398 | -1.0110809 | 0.5786012 | FALSE |
| 5 | 5 | 55 | 3 | 6 | 21445 | -0.0590567 | -0.7681172 | 0.65000381 | FALSE |
| 2 | 5 | 5 | 4 | 3 | 21448 | 0.25819022 | -0.1884004 | 0.70478088 | FALSE |
| 2 | 5 | 31 | 2 | 5 | 21458 | -0.0865846 | -0.9590076 | 0.7858383 | FALSE |
| 7 | 43 | 33 | 9 | 10 | 21461 | 0.7069074 | -0.3407122 | 1.75452698 | FALSE |
| 4 | 1 | 7 | 1 | 5 | 21953 | -0.2189888 | -0.6796615 | 0.24168391 | FALSE |
| 4 | 1 | 42 | 1 | 6 | 22008 | -0.313468 | -1.144645 | 0.51770913 | FALSE |
| 5 | 2 | 25 | 2 | 9 | 22015 | -0.3825897 | -1.2323773 | 0.46719785 | FALSE |
| 2 | 0 | 14 | 0 | 6 | 22074 | -0.2970193 | -0.9145446 | 0.32050598 | FALSE |
| 7 | 10 | 56 | 5 | 16 | 22112 | -0.0068314 | -1.0271285 | 1.01346572 | FALSE |
| 5 | 4 | 8 | 2 | 4 | 22149 | -0.0131951 | -0.36922 | 0.3428297 | FALSE |
| 2 | 0 | 15 | 0 | 5 | 22152 | -0.1269328 | -0.646396 | 0.39253035 | FALSE |
| 2 | 0 | 20 | 0 | 5 | 22153 | -0.3656595 | -0.9656322 | 0.23431331 | FALSE |
| 4 | 3 | 13 | 1 | 9 | 22197 | -0.025357 | -0.4886777 | 0.43796362 | FALSE |
| 5 | 1 | 12 | 1 | 10 | 22209 | -0.2427302 | -0.5959384 | 0.11047802 | FALSE |
| 2 | 0 | 7 | 0 | 6 | 22220 | -0.1680539 | -0.5026777 | 0.16656993 | FALSE |
| 4 | 3 | 13 | 2 | 6 | 22230 | 0.13767743 | -0.3585234 | 0.63387828 | FALSE |
| 2 | 0 | 9 | 0 | 5 | 22305 | -0.1836074 | -0.5902921 | 0.22307727 | FALSE |
| 1 | 0 | 12 | 0 | 6 | 22336 | -0.2150646 | -0.7178721 | 0.28774288 | FALSE |
| 3 | 1 | 48 | 1 | 7 | 22361 | -0.4607493 | -1.4674312 | 0.54593256 | FALSE |
| 2 | 3 | 18 | 1 | 4 | 22383 | -0.0940298 | -0.7809068 | 0.59284715 | FALSE |
| 5 | 0 | 34 | 0 | 11 | 22414 | -0.4231627 | -1.1980238 | 0.35169834 | FALSE |
| 3 | 0 | 12 | 0 | 7 | 22446 | -0.2968649 | -0.8959193 | 0.30218943 | FALSE |
| 4 | 1 | 5 | 1 | 4 | 22474 | -0.0087517 | -0.2404995 | 0.2229961 | FALSE |
| 1 | 0 | 39 | 0 | 6 | 22491 | -0.2894055 | -1.1610427 | 0.5822317 | FALSE |
| 9 | 3 | 37 | 2 | 15 | 22495 | -0.5678623 | -1.247248 | 0.11152332 | FALSE |
| 9 | 12 | 47 | 5 | 17 | 22503 | -0.1933822 | -1.1269932 | 0.74022888 | FALSE |
| 5 | 1 | 7 | 1 | 6 | 22531 | 0.01369319 | -0.2666467 | 0.29403308 | FALSE |
| 2 | 0 | 7 | 0 | 5 | 22550 | -0.1948619 | -0.5697152 | 0.17999141 | FALSE |
| 3 | 0 | 10 | 0 | 6 | 22554 | -0.1590559 | -0.5620389 | 0.24392703 | FALSE |
| 7 | 4 | 12 | 3 | 10 | 22611 | 0.02502485 | -0.3672891 | 0.41733877 | FALSE |
| 5 | 1 | 14 | 1 | 10 | 22695 | -0.2740412 | -0.7205699 | 0.17248748 | FALSE |
| 7 | 9 | 68 | 6 | 18 | 22716 | -0.3734664 | -1.3554207 | 0.60848794 | FALSE |
| 3 | 2 | 16 | 1 | 5 | 22740 | -0.1964044 | -0.7278601 | 0.33505144 | FALSE |
| 2 | 0 | 9 | 0 | 5 | 22772 | -0.2179307 | -0.6267445 | 0.19088298 | FALSE |
| 2 | 0 | 9 | 0 | 6 | 22793 | -0.122345 | -0.4982466 | 0.25355659 | FALSE |
| 2 | 0 | 15 | 0 | 7 | 22797 | -0.3139201 | -0.9555593 | 0.3277191 | FALSE |
| 5 | 7 | 17 | 2 | 7 | 22889 | 0.06139394 | -0.5380406 | 0.66082845 | FALSE |
| 4 | 0 | 13 | 0 | 10 | 22911 | -0.2491501 | -0.680808 | 0.18250777 | FALSE |
| 2 | 0 | 90 | 0 | 10 | 22917 | -0.2677943 | -1.4462519 | 0.91066335 | FALSE |
| 5 | 1 | 10 | 1 | 5 | 23018 | -0.0600901 | -0.3843671 | 0.26418701 | FALSE |
| 2 | 0 | 8 | 0 | 6 | 23025 | -0.095933 | -0.434606 | 0.24273996 | FALSE |
| 2 | 0 | 23 | 0 | 7 | 23029 | -0.1770128 | -0.8732586 | 0.519233 | FALSE |
| 5 | 13 | 10 | 3 | 6 | 23060 | 0.0727951 | -0.58046 | 0.72605022 | FALSE |
| 4 | 4 | 16 | 4 | 5 | 23072 | 0.09955799 | -0.5386314 | 0.73774737 | FALSE |
| 3 | 5 | 6 | 1 | 5 | 23093 | 0.28417138 | -0.5337265 | 1.10206927 | FALSE |
| 2 | 44 | 1 | 4 | 1 | 23108 | 0.61503026 | -0.5179881 | 1.74804865 | FALSE |
| 3 | 10 | 3 | 3 | 2 | 23110 | 0.35947724 | -0.0959844 | 0.81493886 | FALSE |
| 6 | 6 | 14 | 3 | 8 | 23127 | -0.0983254 | -0.5963993 | 0.39974847 | FALSE |
| 6 | 10 | 8 | 2 | 8 | 23163 | 0.11037073 | -0.3923657 | 0.61310714 | FALSE |
| 9 | 14 | 1664 | 4 | 18 | 23168 | -1.7272589 | -3.8710866 | 0.41656873 | FALSE |
| 3 | 0 | 7 | 0 | 6 | 23175 | -0.1298361 | -0.5328256 | 0.27315349 | FALSE |
| 3 | 0 | 8 | 0 | 5 | 23185 | -0.1620746 | -0.4853835 | 0.16123436 | FALSE |
| 2 | 0 | 26 | 0 | 6 | 23193 | -0.4399817 | -1.2837058 | 0.40374233 | FALSE |
| 5 | 2 | 30 | 2 | 9 | 23196 | -0.129787 | -0.829785 | 0.57021089 | FALSE |
| 6 | 0 | 69 | 0 | 14 | 23223 | -0.8991008 | -1.9593007 | 0.16109907 | FALSE |
| 8 | 45 | 31 | 6 | 15 | 23235 | 0.45789194 | -0.4575838 | 1.37336769 | FALSE |
| 7 | 13 | 24 | 4 | 11 | 23239 | 0.10196028 | -0.8102818 | 1.01420234 | FALSE |
| 3 | 0 | 21 | 0 | 8 | 23268 | -0.4678514 | -1.2098054 | 0.27410272 | FALSE |
| 3 | 2 | 4 | 1 | 4 | 23276 | 0.02073743 | -0.2689045 | 0.3103794 | FALSE |
| 4 | 1 | 17 | 1 | 7 | 23306 | -0.1370995 | -0.627945 | 0.35374589 | FALSE |
| 3 | 0 | 18 | 0 | 5 | 23312 | -0.2894564 | -1.0400082 | 0.46109542 | FALSE |
| 2 | 0 | 11 | 0 | 5 | 23317 | -0.1978241 | -0.6448655 | 0.24921733 | FALSE |
| 4 | 1 | 29 | 1 | 8 | 23340 | -0.5526897 | -1.5728241 | 0.46744478 | FALSE |
| 4 | 2 | 4 | 2 | 3 | 23377 | 0.10276008 | -0.1507647 | 0.35628485 | FALSE |
| 4 | 2 | 5 | 2 | 4 | 23388 | -0.0005664 | -0.2795801 | 0.2784474 | FALSE |
| 4 | 3 | 6 | 2 | 4 | 23402 | 0.05997517 | -0.2628507 | 0.382801 | FALSE |
| 1 | 0 | 15 | 0 | 5 | 23551 | -0.211019 | -0.7890565 | 0.36701841 | FALSE |
| 1 | 0 | 42 | 0 | 5 | 23554 | -0.3003956 | -1.1828447 | 0.58205343 | FALSE |
| 2 | 0 | 23 | 0 | 6 | 23775 | -0.1999681 | -0.8852512 | 0.48531498 | FALSE |
| 2 | 0 | 18 | 0 | 5 | 23784 | -0.1619742 | -0.7724136 | 0.44846512 | FALSE |
| 4 | 1 | 85 | 1 | 8 | 23835 | -0.3847901 | -1.5207543 | 0.75117413 | FALSE |
| 2 | 4 | 40 | 2 | 6 | 23844 | 0.08469962 | -0.859775 | 1.02917424 | FALSE |
| 2 | 3 | 26 | 1 | 4 | 23847 | 0.07156261 | -0.6853228 | 0.82844801 | FALSE |
| 1 | 0 | 10 | 0 | 5 | 23904 | -0.1512194 | -0.6151864 | 0.31274751 | FALSE |
| 2 | 1 | 13 | 1 | 4 | 23962 | -0.0135383 | -0.5442946 | 0.51721796 | FALSE |
| 3 | 0 | 9 | 0 | 5 | 23964 | -0.2994552 | -0.641978 | 0.04306761 | FALSE |
| 1 | 0 | 37 | 0 | 6 | 24077 | -0.2244134 | -1.064405 | 0.61557829 | FALSE |
| 1 | 0 | 33 | 0 | 5 | 24084 | -0.1492281 | -0.9718976 | 0.67344141 | FALSE |
| 2 | 4 | 13 | 2 | 3 | 24138 | -0.0191363 | -0.5695157 | 0.53124305 | FALSE |
| 1 | 0 | 11 | 0 | 5 | 24214 | -0.1093352 | -0.5906068 | 0.37193648 | FALSE |
| 1 | 0 | 9 | 0 | 5 | 24372 | -0.1552863 | -0.5873486 | 0.27677601 | FALSE |
| 2 | 0 | 16 | 0 | 6 | 24435 | -0.3758102 | -0.962581 | 0.21096055 | FALSE |
| 2 | 2 | 4 | 2 | 4 | 24447 | 0.06113192 | -0.2935005 | 0.41576434 | FALSE |
| 5 | 16 | 17 | 6 | 8 | 24593 | 0.2957078 | -0.4842955 | 1.07571113 | FALSE |
| 4 | 1 | 9 | 1 | 6 | 24621 | -0.15969 | -0.5223778 | 0.20299785 | FALSE |
| 3 | 1 | 75 | 1 | 5 | 24629 | -0.1000571 | -1.2055552 | 1.00544104 | FALSE |
| 2 | 3 | 22 | 1 | 4 | 24679 | -0.02448 | -0.7339332 | 0.68497321 | FALSE |
| 2 | 0 | 12 | 0 | 6 | 24699 | -0.142758 | -0.6226657 | 0.33714969 | FALSE |
| 2 | 0 | 83 | 0 | 7 | 24761 | -0.4961071 | -1.7067523 | 0.71453801 | FALSE |
| 1 | 0 | 12 | 0 | 6 | 24764 | -0.2436662 | -0.7655254 | 0.27819295 | FALSE |
| 2 | 0 | 6 | 0 | 5 | 24881 | -0.0703817 | -0.3514754 | 0.21071203 | FALSE |
| 3 | 2 | 14 | 2 | 5 | 24901 | 0.0375703 | -0.4873994 | 0.56254001 | FALSE |
| 2 | 1 | 32 | 1 | 4 | 25017 | -0.046272 | -0.7881475 | 0.69560361 | FALSE |
| 9 | 19 | 45 | 9 | 15 | 25080 | 0.55419286 | -0.3053877 | 1.41377339 | FALSE |
| 11 | 124 | 204 | 12 | 26 | 25150 | 0.72161889 | -0.6451933 | 2.0884311 | FALSE |
| 4 | 469 | 5 | 6 | 2 | 25156 | 1.37526592 | -0.505806 | 3.25633784 | FALSE |
| 7 | 13 | 337 | 4 | 17 | 25164 | -0.803206 | -2.7425072 | 1.13609524 | FALSE |
| 10 | 87 | 67 | 10 | 17 | 25169 | 0.64644652 | -0.4739639 | 1.76685691 | FALSE |
| 2 | 19 | 14 | 2 | 3 | 25176 | 0.42873985 | -0.2453899 | 1.10286955 | FALSE |
| 3 | 1 | 7 | 1 | 4 | 25265 | 0.0015016 | -0.335667 | 0.33867022 | FALSE |
| 12 | 29 | 137 | 8 | 19 | 25329 | -0.4310005 | -1.6474339 | 0.78543296 | FALSE |
| 2 | 0 | 9 | 0 | 5 | 25438 | -0.0856107 | -0.4390349 | 0.26781344 | FALSE |
| 3 | 2 | 9 | 2 | 4 | 25514 | 0.04809279 | -0.3581344 | 0.45432 | FALSE |
| 4 | 0 | 10 | 0 | 6 | 25520 | -0.2035268 | -0.5551537 | 0.14810009 | FALSE |
| 7 | 17 | 126 | 5 | 17 | 25526 | -0.4780414 | -1.8211823 | 0.86509945 | FALSE |
| 4 | 3 | 15 | 3 | 4 | 25531 | 0.07180487 | -0.4497742 | 0.59338393 | FALSE |
| 3 | 1 | 26 | 1 | 6 | 25626 | -0.1374626 | -0.8612264 | 0.58630108 | FALSE |
| 4 | 2 | 11 | 1 | 5 | 25693 | -0.0588546 | -0.8360289 | 0.71831977 | FALSE |
| 6 | 4 | 14 | 3 | 9 | 25716 | -0.0972217 | -0.6097707 | 0.41532725 | FALSE |
| 3 | 0 | 11 | 0 | 7 | 25718 | -0.1722381 | -0.5395475 | 0.19507132 | FALSE |
| 4 | 2 | 5 | 1 | 5 | 25741 | -0.0085471 | -0.2824512 | 0.26535705 | FALSE |
| 4 | 3 | 5 | 3 | 4 | 25830 | 0.06683028 | -0.2480669 | 0.38172747 | FALSE |
| 6 | 12 | 141 | 6 | 17 | 25834 | -0.4738698 | -1.9075338 | 0.9597942 | FALSE |
| 5 | 8 | 14 | 3 | 8 | 25854 | 0.21802063 | -0.3483569 | 0.78439818 | FALSE |
| 4 | 1 | 39 | 1 | 5 | 25913 | -0.4491007 | -1.6246812 | 0.72647982 | FALSE |
| 4 | 3 | 3 | 3 | 2 | 25927 | 0.17772362 | -0.0499475 | 0.40539474 | FALSE |
| 7 | 10 | 30 | 6 | 8 | 25930 | 0.05266797 | -0.8827344 | 0.98807038 | FALSE |
| 9 | 75 | 450 | 9 | 19 | 25931 | 0.01682386 | -2.1452396 | 2.17888734 | FALSE |
| 5 | 3 | 6 | 3 | 5 | 25966 | 0.05825564 | -0.2871863 | 0.40369757 | FALSE |
| 4 | 10 | 3 | 6 | 2 | 25969 | 0.43330226 | -0.0565821 | 0.92318657 | FALSE |
| 3 | 0 | 10 | 0 | 5 | 26142 | -0.2205142 | -0.6178169 | 0.17678853 | FALSE |
| 2 | 0 | 6 | 0 | 5 | 26435 | -0.1302166 | -0.4418871 | 0.18145397 | FALSE |
| 4 | 0 | 25 | 0 | 8 | 26544 | -0.2491961 | -0.9072255 | 0.40883331 | FALSE |
| 8 | 189 | 285 | 9 | 23 | 26555 | 0.7008486 | -1.4995656 | 2.90126283 | FALSE |
| 6 | 8 | 15 | 3 | 9 | 26582 | 0.11910218 | -0.4237519 | 0.66195627 | FALSE |
| 5 | 0 | 104 | 0 | 15 | 26601 | -0.79529 | -1.9472214 | 0.35664145 | FALSE |
| 3 | 1 | 7 | 1 | 5 | 26605 | -0.1683916 | -0.558038 | 0.22125474 | FALSE |
| 3 | 6 | 14 | 3 | 4 | 26638 | 0.01904412 | -0.6698923 | 0.70798057 | FALSE |
| 1 | 0 | 22 | 0 | 5 | 26708 | -0.2850919 | -0.9642653 | 0.39408143 | FALSE |
| 2 | 0 | 23 | 0 | 6 | 26741 | -0.3199898 | -0.9475133 | 0.30753369 | FALSE |
| 2 | 1 | 29 | 1 | 4 | 26750 | -0.1966586 | -0.9243622 | 0.53104506 | FALSE |
| 2 | 0 | 18 | 0 | 6 | 26755 | -0.2933866 | -0.9748704 | 0.38809724 | FALSE |
| 3 | 0 | 8 | 0 | 5 | 26812 | -0.3236289 | -0.6985985 | 0.0513406 | FALSE |
| 2 | 0 | 22 | 0 | 5 | 26884 | -0.4892009 | -1.3965025 | 0.41810078 | FALSE |
| 4 | 0 | 6 | 0 | 6 | 26885 | -0.1931245 | -0.4239518 | 0.0377027 | FALSE |
| 3 | 0 | 10 | 0 | 5 | 26925 | -0.1268386 | -0.5240861 | 0.27040883 | FALSE |
| 3 | 3 | 5 | 3 | 2 | 26941 | 0.09488634 | -0.3346387 | 0.52441136 | FALSE |
| 3 | 0 | 7 | 0 | 6 | 26998 | -0.1229484 | -0.3910446 | 0.14514793 | FALSE |
| 5 | 1 | 12 | 1 | 8 | 27100 | -0.0418976 | -0.4433623 | 0.35956698 | FALSE |
| 2 | 0 | 13 | 0 | 6 | 27126 | -0.2869501 | -0.7298006 | 0.15590031 | FALSE |
| 1 | 0 | 13 | 0 | 5 | 27140 | -0.2159936 | -0.7386599 | 0.30667261 | FALSE |
| 2 | 0 | 9 | 0 | 7 | 27262 | -0.0939845 | -0.4896798 | 0.30171072 | FALSE |
| 3 | 0 | 23 | 0 | 6 | 27263 | -0.3426738 | -1.1130105 | 0.42766286 | FALSE |
| 2 | 3 | 4 | 3 | 2 | 27267 | 0.12030665 | -0.2207155 | 0.46132883 | FALSE |
| 3 | 0 | 6 | 0 | 5 | 27274 | -0.1680553 | -0.4750589 | 0.13894821 | FALSE |
| 3 | 2 | 6 | 1 | 5 | 27287 | -0.1662095 | -0.4856836 | 0.15326459 | FALSE |
| 4 | 6 | 6 | 2 | 5 | 27335 | 0.06571895 | -0.329863 | 0.46130086 | FALSE |
| 4 | 1 | 5 | 1 | 4 | 27340 | -0.0747977 | -0.3133405 | 0.16374511 | FALSE |
| 4 | 0 | 19 | 0 | 9 | 27352 | -0.4072974 | -1.0213021 | 0.20670734 | FALSE |
| 3 | 0 | 11 | 0 | 7 | 27353 | -0.2593241 | -0.7264063 | 0.20775821 | FALSE |
| 5 | 0 | 97 | 0 | 11 | 27369 | -0.7825778 | -2.1394586 | 0.57430293 | FALSE |
| 3 | 0 | 10 | 0 | 6 | 27371 | -0.2560308 | -0.6946112 | 0.18254959 | FALSE |
| 6 | 1 | 28 | 1 | 13 | 27376 | -0.4060159 | -1.0007493 | 0.18871756 | FALSE |
| 10 | 53 | 244 | 8 | 26 | 27377 | -0.5085837 | -2.1753548 | 1.1581874 | FALSE |
| 2 | 0 | 6 | 0 | 5 | 27379 | -0.0600915 | -0.3388411 | 0.21865806 | FALSE |
| 3 | 8 | 6 | 3 | 4 | 27394 | 0.24837306 | -0.2765828 | 0.77332893 | FALSE |
| 5 | 72 | 27 | 10 | 8 | 27410 | 1.10540508 | -0.181588 | 2.39239812 | FALSE |
| 3 | 1 | 10 | 1 | 5 | 27418 | -0.1133048 | -0.5840973 | 0.35748776 | FALSE |
| 3 | 2 | 10 | 1 | 5 | 27425 | -0.0795106 | -0.5812158 | 0.42219457 | FALSE |
| 3 | 1 | 12 | 1 | 4 | 27432 | -0.0734014 | -0.5084668 | 0.36166393 | FALSE |
| 3 | 0 | 50 | 0 | 11 | 27465 | -0.3579077 | -1.3447246 | 0.62890918 | FALSE |
| 2 | 0 | 16 | 0 | 5 | 27468 | -0.0176668 | -0.5427147 | 0.5073811 | FALSE |
| 3 | 3 | 5 | 3 | 4 | 27501 | 0.11076644 | -0.2769533 | 0.49848623 | FALSE |
| 2 | 0 | 14 | 0 | 6 | 27527 | -0.3843594 | -1.0513847 | 0.2826659 | FALSE |
| 2 | 1 | 15 | 1 | 4 | 27534 | -0.2093078 | -0.8486174 | 0.43000178 | FALSE |
| 6 | 10 | 60 | 2 | 15 | 27644 | -0.4316285 | -1.5247003 | 0.6614433 | FALSE |
| 7 | 7 | 138 | 3 | 15 | 27699 | -0.3952302 | -1.486833 | 0.69637262 | FALSE |
| 5 | 11 | 13 | 3 | 6 | 27702 | 0.10556524 | -0.4143075 | 0.625438 | FALSE |
| 3 | 0 | 8 | 0 | 7 | 27722 | -0.2295334 | -0.6207099 | 0.16164311 | FALSE |
| 1 | 0 | 6 | 0 | 5 | 27730 | -0.1398072 | -0.4827161 | 0.20310163 | FALSE |
| 4 | 1 | 9 | 1 | 5 | 27740 | -0.1541587 | -0.6108477 | 0.3025303 | FALSE |
| 9 | 140 | 532 | 4 | 30 | 27809 | -1.186199 | -3.4361577 | 1.06375978 | FALSE |
| 7 | 3 | 39 | 2 | 10 | 27810 | -0.4334396 | -1.3608811 | 0.49400181 | FALSE |
| 2 | 0 | 11 | 0 | 5 | 27874 | -0.2175167 | -0.7017351 | 0.2667017 | FALSE |
| 1 | 0 | 74 | 0 | 6 | 27905 | -0.488854 | -1.6747611 | 0.69705307 | FALSE |
| 2 | 3 | 11 | 2 | 4 | 27910 | 0.03878561 | -0.4386537 | 0.5162249 | FALSE |
| 1 | 0 | 14 | 0 | 6 | 27988 | -0.2335235 | -0.7927665 | 0.32571962 | FALSE |
| 1 | 0 | 24 | 0 | 6 | 28072 | -0.2136298 | -0.9338377 | 0.5065781 | FALSE |
| 2 | 1 | 67 | 1 | 6 | 28088 | -0.2245491 | -1.3564874 | 0.90738925 | FALSE |
| 1 | 0 | 58 | 0 | 5 | 28100 | -0.2298317 | -1.2531185 | 0.79345502 | FALSE |
| 1 | 0 | 47 | 0 | 6 | 28101 | -0.241335 | -1.2068031 | 0.7241331 | FALSE |
| 1 | 0 | 79 | 0 | 5 | 28262 | -0.1935369 | -1.3133646 | 0.92629084 | FALSE |
| 2 | 0 | 25 | 0 | 7 | 28293 | -0.4942044 | -1.1598702 | 0.17146146 | FALSE |
| 3 | 3 | 3 | 3 | 2 | 28296 | 0.12278657 | -0.1673006 | 0.41287374 | FALSE |
| 4 | 6 | 77 | 3 | 7 | 28332 | -0.1220346 | -1.2525778 | 1.00850851 | FALSE |
| 1 | 0 | 9 | 0 | 5 | 28342 | -0.1258411 | -0.5472214 | 0.29553927 | FALSE |
| 3 | 5 | 3 | 3 | 2 | 28361 | 0.20714662 | -0.1204348 | 0.53472806 | FALSE |
| 2 | 0 | 10 | 0 | 6 | 28368 | -0.1489698 | -0.5736922 | 0.27575256 | FALSE |
| 2 | 0 | 9 | 0 | 6 | 28399 | -0.1599207 | -0.5530574 | 0.23321594 | FALSE |
| 3 | 2 | 5 | 1 | 4 | 28400 | -0.0180827 | -0.381513 | 0.34534765 | FALSE |
| 4 | 2 | 49 | 2 | 4 | 28404 | -0.2963497 | -1.3294993 | 0.73679983 | FALSE |
| 4 | 1 | 12 | 1 | 5 | 28421 | -0.0952788 | -0.5246962 | 0.33413865 | FALSE |
| 4 | 1 | 15 | 1 | 8 | 28431 | -0.2479842 | -0.7524848 | 0.25651634 | FALSE |
| 1 | 0 | 23 | 0 | 5 | 28599 | -0.2617766 | -0.9529323 | 0.42937903 | FALSE |
| 4 | 2 | 7 | 1 | 5 | 28624 | -0.0263319 | -0.3289665 | 0.27630266 | FALSE |
| 2 | 1 | 28 | 1 | 4 | 28640 | -0.1183427 | -0.8128277 | 0.57614239 | FALSE |
| 3 | 18 | 111 | 4 | 9 | 28901 | -0.1627749 | -1.6220902 | 1.29654045 | FALSE |
| 4 | 5 | 340 | 4 | 10 | 28905 | -0.9167904 | -3.0482743 | 1.21469357 | FALSE |
| 6 | 42 | 33 | 9 | 11 | 28908 | 0.57212328 | -0.6435641 | 1.78781067 | FALSE |
| 2 | 73 | 11 | 3 | 3 | 28959 | 0.74879344 | -0.4214437 | 1.91903061 | FALSE |
| 8 | 33 | 25 | 7 | 10 | 29075 | 0.68283274 | -0.3792263 | 1.74489181 | FALSE |
| 2 | 1 | 7 | 1 | 4 | 29141 | -0.1132528 | -0.4960089 | 0.26950334 | FALSE |
| 5 | 4 | 14 | 3 | 6 | 29176 | 0.1481991 | -0.3441879 | 0.64058608 | FALSE |
| 5 | 4 | 5 | 3 | 4 | 29210 | 0.13253619 | -0.1532522 | 0.41832456 | FALSE |
| 3 | 1 | 7 | 1 | 4 | 29229 | 0.02657062 | -0.3093048 | 0.36244607 | FALSE |
| 3 | 2 | 108 | 2 | 8 | 29297 | -0.608163 | -2.0014823 | 0.78515631 | FALSE |
| 4 | 6 | 25 | 3 | 7 | 29307 | 0.06539848 | -0.7552447 | 0.88604163 | FALSE |
| 1 | 0 | 118 | 0 | 6 | 29321 | -0.6811931 | -2.0974688 | 0.73508266 | FALSE |
| 3 | 0 | 31 | 0 | 8 | 29370 | -0.2050011 | -0.9724476 | 0.56244551 | FALSE |
| 2 | 3 | 4 | 2 | 4 | 29671 | 0.03186131 | -0.3395386 | 0.40326127 | FALSE |
| 4 | 2 | 5 | 1 | 5 | 29679 | -0.0134038 | -0.285027 | 0.25821949 | FALSE |
| 3 | 0 | 33 | 0 | 9 | 29759 | -0.2412771 | -0.9021232 | 0.41956888 | FALSE |
| 8 | 458 | 34 | 7 | 9 | 29762 | 1.47058435 | -0.4199446 | 3.36111335 | FALSE |
| 9 | 4 | 93 | 3 | 19 | 29770 | -0.4457134 | -1.2876316 | 0.3962048 | FALSE |
| 2 | 7 | 8 | 3 | 4 | 29778 | 0.25932975 | -0.2974872 | 0.81614672 | FALSE |
| 8 | 7 | 147 | 5 | 20 | 29792 | -0.9264141 | -2.1452179 | 0.29238959 | FALSE |
| 5 | 1 | 13 | 1 | 7 | 29932 | -0.1263223 | -0.557814 | 0.3051693 | FALSE |
| 4 | 5 | 5 | 3 | 3 | 30001 | 0.30291666 | -0.0738565 | 0.67968983 | FALSE |
| 2 | 0 | 18 | 0 | 5 | 30111 | -0.3080896 | -1.0624713 | 0.44629205 | FALSE |
| 1 | 0 | 19 | 0 | 5 | 30113 | -0.284576 | -0.925159 | 0.35600702 | FALSE |
| 3 | 4 | 34 | 3 | 7 | 30114 | -0.0596371 | -0.8960189 | 0.7767448 | FALSE |
| 1 | 0 | 25 | 0 | 5 | 30274 | -0.1243415 | -0.8173622 | 0.5686792 | FALSE |
| 3 | 1 | 7 | 1 | 6 | 30339 | -0.0951574 | -0.4671333 | 0.27681857 | FALSE |
| 2 | 2 | 4 | 2 | 3 | 30449 | -0.0815818 | -0.5262113 | 0.36304771 | FALSE |
| 3 | 41 | 1 | 6 | 1 | 30455 | 0.98297155 | -0.0680663 | 2.03400941 | FALSE |
| 6 | 9 | 56 | 4 | 9 | 30457 | -0.486539 | -1.8196952 | 0.84661708 | FALSE |
| 10 | 60 | 224 | 14 | 21 | 30479 | 0.84108521 | -0.6377846 | 2.31995501 | FALSE |
| 6 | 3 | 20 | 3 | 7 | 30487 | -0.1081838 | -0.6998735 | 0.48350601 | FALSE |
| 2 | 1 | 6 | 1 | 4 | 30654 | -0.0103606 | -0.3654718 | 0.34475057 | FALSE |
| 3 | 1 | 8 | 1 | 4 | 30701 | 0.02170628 | -0.3610739 | 0.40448646 | FALSE |
| 1 | 0 | 17 | 0 | 5 | 30749 | -0.3321446 | -0.9579453 | 0.29365617 | FALSE |
| 3 | 7 | 4 | 2 | 4 | 30801 | 0.18124185 | -0.2158785 | 0.57836223 | FALSE |
| 3 | 0 | 10 | 0 | 6 | 30812 | -0.2572611 | -0.7629162 | 0.24839401 | FALSE |
| 3 | 6 | 6 | 2 | 4 | 30815 | 0.17432037 | -0.2957438 | 0.64438452 | FALSE |
| 3 | 2 | 74 | 1 | 8 | 30851 | -0.2267844 | -1.3683347 | 0.91476592 | FALSE |
| 2 | 0 | 36 | 0 | 6 | 31023 | -0.40151 | -1.2087872 | 0.40576726 | FALSE |
| 5 | 0 | 17 | 0 | 8 | 31037 | -0.3802343 | -0.9444755 | 0.18400703 | FALSE |
| 3 | 2 | 7 | 1 | 4 | 31038 | -0.0181108 | -0.3792911 | 0.34306946 | FALSE |
| 3 | 0 | 9 | 0 | 6 | 31074 | -0.1571568 | -0.53525 | 0.22093642 | FALSE |
| 5 | 5 | 13 | 4 | 6 | 31119 | 0.21542881 | -0.2846932 | 0.71555079 | FALSE |
| 4 | 0 | 20 | 0 | 8 | 31162 | -0.3135179 | -0.8123661 | 0.18533027 | FALSE |
| 5 | 4 | 8 | 3 | 6 | 31203 | 0.06631923 | -0.346477 | 0.47911542 | FALSE |
| 2 | 0 | 7 | 0 | 5 | 31207 | -0.200013 | -0.5280254 | 0.12799947 | FALSE |
| 2 | 0 | 6 | 0 | 5 | 31239 | -0.1993311 | -0.6014827 | 0.20282059 | FALSE |
| 2 | 0 | 8 | 0 | 5 | 31267 | -0.1438997 | -0.5332619 | 0.24546258 | FALSE |
| 4 | 5 | 7 | 2 | 6 | 31430 | 0.11101602 | -0.3011785 | 0.52321056 | FALSE |
| 3 | 0 | 7 | 0 | 5 | 31510 | -0.0224188 | -0.2995097 | 0.25467217 | FALSE |
| 3 | 0 | 13 | 0 | 7 | 31512 | -0.1942755 | -0.6054141 | 0.21686305 | FALSE |
| 3 | 0 | 9 | 0 | 5 | 31521 | -0.1438257 | -0.4961327 | 0.20848133 | FALSE |
| 3 | 1 | 14 | 1 | 5 | 31533 | -0.1084233 | -0.6241383 | 0.40729166 | FALSE |
| 4 | 35 | 11 | 5 | 4 | 31549 | 0.8087158 | -0.2171424 | 1.83457403 | FALSE |
| 4 | 2 | 6 | 1 | 4 | 31568 | -0.1089024 | -0.4650207 | 0.24721577 | FALSE |
| 4 | 1 | 8 | 1 | 5 | 31575 | -0.0119636 | -0.3330432 | 0.30911593 | FALSE |
| 5 | 3 | 29 | 3 | 9 | 31577 | -0.1406324 | -1.033824 | 0.75255908 | FALSE |
| 1 | 0 | 12 | 0 | 5 | 31629 | -0.1416357 | -0.6681716 | 0.38490008 | FALSE |
| 6 | 8 | 41 | 5 | 16 | 31646 | -0.1817992 | -1.1012661 | 0.73766764 | FALSE |
| 4 | 1 | 5 | 1 | 5 | 31934 | 0.002313 | -0.2471396 | 0.2517656 | FALSE |
| 3 | 0 | 39 | 0 | 9 | 31936 | -0.4337762 | -1.3460469 | 0.47849445 | FALSE |
| 4 | 0 | 43 | 0 | 10 | 31950 | -0.5837552 | -1.5116014 | 0.34409101 | FALSE |
| 5 | 0 | 56 | 0 | 14 | 31952 | -0.6502907 | -1.6802748 | 0.37969347 | FALSE |
| 3 | 0 | 11 | 0 | 5 | 32079 | -0.1027346 | -0.5092366 | 0.30376739 | FALSE |
| 3 | 0 | 6 | 0 | 5 | 32122 | -0.1461594 | -0.5344153 | 0.2420965 | FALSE |
| 2 | 3 | 10 | 2 | 3 | 32241 | 0.06378574 | -0.4780883 | 0.60565975 | FALSE |
| 5 | 4 | 8 | 2 | 5 | 32298 | -0.0264602 | -0.3862308 | 0.33331044 | FALSE |
| 10 | 64 | 222 | 8 | 26 | 32339 | -0.1730637 | -1.4891951 | 1.14306775 | FALSE |
| 8 | 4 | 244 | 2 | 12 | 32399 | -0.7442297 | -2.3507029 | 0.86224344 | FALSE |
| 1 | 0 | 7 | 0 | 5 | 32557 | -0.1641221 | -0.5367707 | 0.2085265 | FALSE |
| 1 | 0 | 22 | 0 | 5 | 32574 | -0.22864 | -0.9439808 | 0.48670076 | FALSE |
| 2 | 0 | 20 | 0 | 7 | 32715 | -0.2950175 | -0.9263112 | 0.33627625 | FALSE |
| 1 | 0 | 55 | 0 | 5 | 32747 | -0.2684814 | -1.2570426 | 0.72007988 | FALSE |
| 3 | 0 | 15 | 0 | 7 | 32776 | -0.347025 | -0.7916278 | 0.09757776 | FALSE |
| 2 | 0 | 40 | 0 | 7 | 32797 | -0.4825134 | -1.3626449 | 0.39761804 | FALSE |
| 3 | 0 | 15 | 0 | 6 | 32929 | -0.4293981 | -0.8881199 | 0.02932377 | FALSE |
| 4 | 18 | 5 | 4 | 4 | 33014 | 0.49426761 | -0.0862684 | 1.07480359 | FALSE |
| 4 | 5 | 43 | 3 | 6 | 33028 | -0.1754982 | -1.2189994 | 0.86800296 | FALSE |
| 1 | 0 | 17 | 0 | 5 | 33175 | -0.1909425 | -0.7600047 | 0.37811963 | FALSE |
| 1 | 0 | 64 | 0 | 6 | 33176 | -0.4219841 | -1.5372335 | 0.69326534 | FALSE |
| 2 | 25 | 1 | 4 | 1 | 33340 | 0.49676898 | -0.3871637 | 1.38070164 | FALSE |
| 1 | 0 | 8 | 0 | 5 | 33437 | -0.1031752 | -0.5125278 | 0.30617735 | FALSE |
| 2 | 2 | 8 | 2 | 3 | 33441 | -0.0161737 | -0.5870028 | 0.55465531 | FALSE |
| 4 | 6 | 130 | 3 | 8 | 33444 | -0.6488874 | -2.2803833 | 0.98260857 | FALSE |
| 5 | 11 | 12 | 2 | 5 | 33522 | 0.1452161 | -0.3835239 | 0.67395607 | FALSE |
| 4 | 2 | 66 | 1 | 10 | 33672 | -0.62811 | -1.7172536 | 0.4610335 | FALSE |
| 2 | 0 | 13 | 0 | 6 | 33681 | -0.1138819 | -0.5875029 | 0.35973909 | FALSE |
| 2 | 2 | 552 | 1 | 5 | 33712 | -0.8428959 | -3.1252633 | 1.43947158 | FALSE |
| 4 | 4 | 7 | 3 | 4 | 33731 | 0.1266186 | -0.2947939 | 0.54803106 | FALSE |
| 7 | 6 | 25 | 4 | 10 | 33758 | -0.2633155 | -0.9532305 | 0.42659937 | FALSE |
| 4 | 2 | 6 | 2 | 5 | 33778 | -0.0635685 | -0.383865 | 0.25672796 | FALSE |
| 4 | 1 | 14 | 1 | 7 | 33784 | -0.1338955 | -0.5661056 | 0.29831471 | FALSE |
| 2 | 3 | 25 | 3 | 3 | 33792 | 0.11128873 | -0.5354857 | 0.75806317 | FALSE |
| 2 | 2 | 5 | 2 | 3 | 33795 | 0.08836145 | -0.2458133 | 0.42253619 | FALSE |
| 3 | 8 | 7 | 3 | 4 | 33809 | 0.2001653 | -0.3094158 | 0.70974643 | FALSE |
| 1 | 0 | 10 | 0 | 5 | 33825 | -0.2387774 | -0.7626537 | 0.28509884 | FALSE |
| 2 | 0 | 40 | 0 | 7 | 33846 | -0.3628262 | -1.2613403 | 0.53568795 | FALSE |
| 4 | 12 | 39 | 4 | 8 | 33858 | 0.03342603 | -0.981328 | 1.04818006 | FALSE |
| 1 | 0 | 33 | 0 | 6 | 33868 | -0.319655 | -1.2012699 | 0.56196 | FALSE |
| 3 | 3 | 12 | 2 | 4 | 34012 | -0.2186112 | -0.9518505 | 0.51462815 | FALSE |
| 3 | 0 | 7 | 0 | 6 | 34033 | -0.160251 | -0.4495152 | 0.12901319 | FALSE |
| 3 | 9 | 45 | 3 | 4 | 34169 | 0.4277814 | -0.5891824 | 1.44474518 | FALSE |
| 2 | 4 | 5 | 1 | 4 | 34170 | 0.14989185 | -0.305051 | 0.60483473 | FALSE |
| 4 | 0 | 18 | 0 | 9 | 34205 | -0.3413641 | -0.8928117 | 0.21008357 | FALSE |
| 4 | 2 | 11 | 2 | 7 | 34217 | -0.0767121 | -0.5948839 | 0.4414596 | FALSE |
| 2 | 1 | 21 | 1 | 4 | 34220 | 0.0408 | -0.5977929 | 0.67939292 | FALSE |
| 4 | 2 | 14 | 2 | 6 | 34223 | -0.1445021 | -0.7060524 | 0.41704829 | FALSE |
| 3 | 4 | 4 | 3 | 2 | 34225 | 0.14283814 | -0.1788743 | 0.46455063 | FALSE |
| 2 | 12 | 14 | 2 | 4 | 34243 | 0.38940758 | -0.6013091 | 1.38012429 | FALSE |
| 4 | 2 | 6 | 2 | 5 | 34300 | 0.06704119 | -0.3221505 | 0.45623283 | FALSE |
| 3 | 1 | 5 | 1 | 4 | 34304 | -0.0495042 | -0.3343259 | 0.23531761 | FALSE |
| 9 | 65 | 53 | 9 | 15 | 34307 | 0.59835038 | -0.5127308 | 1.70943155 | FALSE |
| 2 | 0 | 8 | 0 | 5 | 34318 | -0.1517705 | -0.4905587 | 0.18701766 | FALSE |
| 9 | 4 | 25 | 3 | 12 | 34343 | -0.0011184 | -0.4971347 | 0.494898 | FALSE |
| 4 | 4 | 9 | 2 | 5 | 34345 | 0.13626716 | -0.3107175 | 0.58325177 | FALSE |
| 10 | 7 | 51 | 3 | 17 | 34426 | 0.00767802 | -0.9624066 | 0.97776266 | FALSE |
| 4 | 1 | 6 | 1 | 6 | 34445 | -0.1329823 | -0.4429672 | 0.17700266 | FALSE |
| 5 | 20 | 38 | 5 | 11 | 34469 | 0.38614784 | -0.6763118 | 1.44860743 | FALSE |
| 8 | 17 | 67 | 6 | 14 | 34651 | 0.27497882 | -0.9242681 | 1.47422575 | FALSE |
| 1 | 0 | 15 | 0 | 5 | 34791 | -0.1149417 | -0.6881153 | 0.45823192 | FALSE |
| 4 | 4 | 7 | 2 | 5 | 34816 | -0.0151873 | -0.4169034 | 0.38652883 | FALSE |
| 2 | 31 | 0 | 5 | 0 | 34903 | 0.75917455 | -0.0905325 | 1.60888156 | FALSE |
| 4 | 2 | 10 | 1 | 5 | 34913 | -0.0440243 | -0.4379868 | 0.34993833 | FALSE |
| 9 | 3 | 81 | 3 | 19 | 34936 | -0.9573417 | -1.9405433 | 0.02585982 | FALSE |
| 3 | 10 | 1 | 4 | 1 | 35011 | 0.43949871 | -0.0219006 | 0.90089802 | FALSE |
| 2 | 14 | 1 | 4 | 1 | 35036 | 0.43576359 | -0.2331698 | 1.104697 | FALSE |
| 3 | 0 | 115 | 0 | 16 | 35094 | -1.062621 | -2.495587 | 0.37034497 | FALSE |
| 6 | 6 | 42 | 3 | 12 | 35113 | -0.2610341 | -1.1724914 | 0.65042316 | FALSE |
| 2 | 0 | 26 | 0 | 7 | 35155 | -0.3141784 | -1.0339324 | 0.4055756 | FALSE |
| 3 | 0 | 7 | 0 | 5 | 35170 | -0.1911086 | -0.4709614 | 0.08874428 | FALSE |
| 3 | 0 | 12 | 0 | 9 | 35253 | -0.3712608 | -0.7506226 | 0.00810099 | FALSE |
| 5 | 6 | 13 | 4 | 6 | 35266 | 0.03640791 | -0.4214439 | 0.49425976 | FALSE |
| 5 | 2 | 7 | 2 | 3 | 35369 | 0.01930252 | -0.2800205 | 0.31862555 | FALSE |
| 6 | 2 | 8 | 2 | 7 | 35389 | -0.088114 | -0.409978 | 0.23374992 | FALSE |
| 2 | 0 | 26 | 0 | 8 | 35415 | -0.3623824 | -1.1213511 | 0.39658641 | FALSE |
| 4 | 1 | 7 | 1 | 6 | 35474 | -0.1217106 | -0.4212339 | 0.17781265 | FALSE |
| 5 | 2 | 8 | 2 | 5 | 35507 | -0.0854613 | -0.4502473 | 0.2793248 | FALSE |
| 3 | 2 | 5 | 2 | 4 | 35605 | 0.08384776 | -0.2308078 | 0.39850332 | FALSE |
| 4 | 3 | 6 | 2 | 5 | 35608 | 0.08240643 | -0.2279998 | 0.39281267 | FALSE |
| 4 | 1 | 5 | 1 | 5 | 35932 | -0.187251 | -0.4601788 | 0.08567687 | FALSE |
| 4 | 1 | 28 | 1 | 5 | 36009 | -0.4041964 | -1.3844545 | 0.5760617 | FALSE |
| 5 | 4 | 16 | 2 | 9 | 36203 | -0.0635898 | -0.5909406 | 0.4637611 | FALSE |
| 2 | 0 | 8 | 0 | 5 | 36227 | -0.1345681 | -0.5408964 | 0.27176018 | FALSE |
| 6 | 7 | 51 | 3 | 12 | 36232 | -0.1795609 | -1.1550483 | 0.79592644 | FALSE |
| 3 | 1 | 7 | 1 | 6 | 36248 | -0.1357072 | -0.5473754 | 0.27596106 | FALSE |
| 4 | 2 | 4 | 2 | 3 | 36259 | 0.08022098 | -0.1724499 | 0.3328919 | FALSE |
| 5 | 2 | 7 | 1 | 6 | 36266 | -0.0925136 | -0.3992987 | 0.21427158 | FALSE |
| 6 | 6 | 63 | 3 | 8 | 36269 | -0.4328854 | -1.6857 | 0.81992923 | FALSE |
| 4 | 4 | 3 | 3 | 3 | 36272 | 0.1883735 | -0.1355292 | 0.51227615 | FALSE |
| 1 | 0 | 27 | 0 | 6 | 36297 | -0.1896507 | -0.96584 | 0.5865386 | FALSE |
| 2 | 29 | 1 | 4 | 1 | 36392 | 0.57141535 | -0.3550809 | 1.49791157 | FALSE |
| 6 | 1 | 22 | 1 | 8 | 36435 | -0.3619907 | -1.0142828 | 0.29030148 | FALSE |
| 10 | 257 | 4664 | 7 | 31 | 36447 | -2.6175395 | -5.336037 | 0.10095787 | FALSE |
| 4 | 1 | 7 | 1 | 5 | 36449 | -0.0260158 | -0.314968 | 0.26293645 | FALSE |
| 1 | 0 | 19 | 0 | 5 | 36466 | -0.2926142 | -0.9243127 | 0.3390844 | FALSE |
| 11 | 241 | 3782 | 8 | 34 | 36481 | -2.3229637 | -4.9287873 | 0.28285995 | FALSE |
| 6 | 15 | 97 | 4 | 12 | 36490 | -0.1839953 | -1.6975518 | 1.32956124 | FALSE |
| 4 | 0 | 108 | 0 | 10 | 36492 | -0.7656154 | -2.072849 | 0.54161819 | FALSE |
| 3 | 2 | 4 | 2 | 3 | 36558 | 0.10136382 | -0.1652076 | 0.36793523 | FALSE |
| 1 | 0 | 10 | 0 | 5 | 36577 | -0.2158672 | -0.7428413 | 0.31110699 | FALSE |
| 2 | 1 | 9 | 1 | 4 | 36583 | -0.0322752 | -0.5000222 | 0.43547185 | FALSE |
| 1 | 0 | 11 | 0 | 5 | 36597 | -0.2213074 | -0.7818483 | 0.33923352 | FALSE |
| 5 | 1 | 19 | 1 | 8 | 36603 | -0.1418398 | -0.6480364 | 0.36435671 | FALSE |
| 5 | 0 | 115 | 0 | 8 | 36604 | -0.6899691 | -1.7469561 | 0.367018 | FALSE |
| 7 | 5 | 166 | 1 | 11 | 36607 | -0.7459469 | -2.06972 | 0.57782624 | FALSE |
| 3 | 5 | 3 | 3 | 2 | 36617 | 0.10397569 | -0.275344 | 0.48329534 | FALSE |
| 4 | 2 | 5 | 1 | 5 | 36628 | 0.00801445 | -0.283195 | 0.2992239 | FALSE |
| 9 | 214 | 379 | 4 | 21 | 36635 | -0.3155488 | -2.86312 | 2.23202243 | FALSE |
| 3 | 6 | 3 | 2 | 3 | 36691 | 0.13633454 | -0.2365745 | 0.50924361 | FALSE |
| 1 | 0 | 8 | 0 | 5 | 36752 | -0.1511919 | -0.5502095 | 0.24782572 | FALSE |
| 1 | 0 | 22 | 0 | 5 | 36829 | -0.2252706 | -0.886049 | 0.43550781 | FALSE |
| 1 | 0 | 35 | 0 | 5 | 36854 | -0.2693313 | -1.1389463 | 0.60028378 | FALSE |
| 4 | 2 | 601 | 1 | 17 | 36874 | -1.23698 | -3.0351151 | 0.56115508 | FALSE |
| 3 | 0 | 13 | 0 | 5 | 37021 | -0.3313174 | -0.8649041 | 0.20226933 | FALSE |
| 4 | 76 | 204 | 3 | 8 | 37557 | 0.39416592 | -1.7254159 | 2.5137477 | FALSE |
| 3 | 5 | 64 | 2 | 6 | 37574 | -0.2778831 | -1.5869766 | 1.03121031 | FALSE |
| 4 | 2 | 16 | 2 | 6 | 37756 | 0.01640001 | -0.5819229 | 0.61472287 | FALSE |
| 3 | 1 | 35 | 1 | 5 | 37774 | -0.3074495 | -1.0284167 | 0.4135176 | FALSE |
| 6 | 5 | 4 | 4 | 4 | 37789 | 0.26203215 | -0.0734757 | 0.59754001 | FALSE |
| 6 | 5 | 10 | 3 | 8 | 37790 | 0.19061915 | -0.2766535 | 0.65789185 | FALSE |
| 7 | 15 | 58 | 7 | 11 | 37807 | 0.088074 | -0.9503137 | 1.12646172 | FALSE |
| 3 | 3 | 12 | 2 | 4 | 37816 | -0.0188918 | -0.4878143 | 0.45003078 | FALSE |
| 10 | 22 | 196 | 7 | 22 | 37827 | -0.1837486 | -1.7210986 | 1.35360142 | FALSE |
| 5 | 18 | 8 | 5 | 6 | 37828 | 0.3523585 | -0.3654038 | 1.07012085 | FALSE |
| 9 | 36 | 92 | 7 | 21 | 37834 | 0.289014 | -0.934923 | 1.51295099 | FALSE |
| 4 | 12 | 25 | 4 | 5 | 37937 | 0.2825945 | -0.779171 | 1.34435996 | FALSE |
| 5 | 108 | 341 | 6 | 11 | 37940 | 0.40037342 | -2.167646 | 2.96839288 | FALSE |
| 3 | 5 | 12 | 2 | 7 | 37942 | 0.12088723 | -0.5836015 | 0.82537595 | FALSE |
| 3 | 23 | 93 | 2 | 9 | 37951 | -0.2181642 | -1.928704 | 1.4923757 | FALSE |
| 1 | 0 | 11 | 0 | 5 | 37967 | -0.2271075 | -0.7251594 | 0.2709444 | FALSE |
| 5 | 4 | 86 | 3 | 16 | 38142 | -0.1299491 | -1.3209944 | 1.06109617 | FALSE |
| 3 | 4 | 5 | 2 | 4 | 38211 | 0.22513099 | -0.1757288 | 0.62599076 | FALSE |
| 6 | 53 | 98 | 9 | 9 | 38258 | 0.61229605 | -1.1331608 | 2.35775287 | FALSE |
| 3 | 3 | 14 | 1 | 5 | 38295 | 0.10042953 | -0.4694854 | 0.6703445 | FALSE |
| 8 | 13 | 45 | 4 | 20 | 38296 | 0.01737172 | -0.8100793 | 0.84482277 | FALSE |
| 6 | 32 | 95 | 4 | 15 | 38318 | 0.15888854 | -1.2079353 | 1.52571233 | FALSE |
| 5 | 5 | 10 | 2 | 3 | 38404 | 0.0524654 | -0.3484585 | 0.45338933 | FALSE |
| 3 | 3 | 3 | 3 | 2 | 38441 | 0.13176389 | -0.1588275 | 0.42235528 | FALSE |
| 7 | 3 | 14 | 2 | 8 | 38470 | 0.01621948 | -0.364422 | 0.396861 | FALSE |
| 5 | 4 | 6 | 4 | 5 | 38474 | 0.04014984 | -0.326777 | 0.40707669 | FALSE |
| 3 | 0 | 9 | 0 | 5 | 38506 | -0.1793171 | -0.5168948 | 0.15826064 | FALSE |
| 11 | 7 | 62 | 4 | 14 | 38531 | -0.2573238 | -1.0318917 | 0.51724417 | FALSE |
| 6 | 12 | 22 | 5 | 10 | 38560 | 0.31433452 | -0.4614037 | 1.09007272 | FALSE |
| 11 | 18 | 69 | 8 | 17 | 38586 | -0.0685787 | -1.053897 | 0.91673969 | FALSE |
| 9 | 10 | 21 | 5 | 10 | 38587 | 0.01342491 | -0.5558275 | 0.58267728 | FALSE |
| 4 | 3 | 8 | 1 | 5 | 38596 | 0.08333205 | -0.2935136 | 0.46017772 | FALSE |
| 5 | 6 | 4 | 2 | 4 | 38644 | 0.37442469 | -0.2950076 | 1.04385701 | FALSE |
| 5 | 4 | 10 | 2 | 5 | 38646 | 0.00153665 | -0.4214826 | 0.42455585 | FALSE |
| 4 | 5 | 5 | 3 | 3 | 38726 | 0.23592138 | -0.0797466 | 0.55158931 | FALSE |
| 6 | 8 | 31 | 5 | 13 | 38727 | 0.00191933 | -0.7196768 | 0.72351551 | FALSE |
| 5 | 6 | 15 | 3 | 7 | 38729 | 0.14245765 | -0.4041934 | 0.68910872 | FALSE |
| 6 | 2 | 16 | 2 | 7 | 38730 | 0.02433556 | -0.4342212 | 0.4828923 | FALSE |
| 4 | 10 | 11 | 4 | 4 | 38863 | 0.46712762 | -0.1818308 | 1.11608603 | FALSE |
| 4 | 11 | 8 | 3 | 5 | 38914 | 0.27341963 | -0.2342434 | 0.78108266 | FALSE |
| 4 | 2 | 8 | 2 | 5 | 39025 | 0.04208747 | -0.3297752 | 0.41395013 | FALSE |
| 6 | 1 | 8 | 1 | 6 | 39038 | -0.0878549 | -0.3882992 | 0.21258948 | FALSE |
| 3 | 0 | 6 | 0 | 6 | 39041 | -0.1161494 | -0.391794 | 0.15949522 | FALSE |
| 8 | 23 | 77 | 7 | 14 | 39110 | 0.19220224 | -1.0685766 | 1.45298103 | FALSE |
| 2 | 0 | 8 | 0 | 6 | 39127 | -0.1384576 | -0.5082992 | 0.23138413 | FALSE |
| 2 | 14 | 1 | 4 | 1 | 39139 | 0.38950851 | -0.2409591 | 1.0199761 | FALSE |
| 2 | 135 | 11 | 4 | 2 | 39146 | 0.91236237 | -0.4854692 | 2.31019391 | FALSE |
| 4 | 0 | 15 | 0 | 7 | 39181 | -0.2949495 | -0.7053435 | 0.11544449 | FALSE |
| 2 | 6 | 7 | 2 | 5 | 39189 | 0.14745946 | -0.3473218 | 0.6422407 | FALSE |
| 6 | 5 | 17 | 3 | 8 | 39208 | 0.07128761 | -0.4477145 | 0.59028975 | FALSE |
| 3 | 0 | 10 | 0 | 6 | 39210 | -0.2990248 | -0.773319 | 0.17526931 | FALSE |
| 3 | 1 | 7 | 1 | 4 | 39243 | -0.0468413 | -0.3574563 | 0.26377369 | FALSE |
| 4 | 10 | 5 | 4 | 5 | 39287 | 0.32692746 | -0.2525431 | 0.90639802 | FALSE |
| 3 | 1 | 6 | 1 | 4 | 39604 | -0.0574177 | -0.3528332 | 0.23799791 | FALSE |
| 3 | 0 | 12 | 0 | 8 | 39776 | -0.1842168 | -0.619687 | 0.25125338 | FALSE |
| 3 | 2 | 7 | 2 | 5 | 39816 | 0.01365584 | -0.3573496 | 0.38466127 | FALSE |
| 4 | 0 | 39 | 0 | 12 | 39964 | -0.5656009 | -1.3353103 | 0.20410854 | FALSE |
| 2 | 0 | 10 | 0 | 5 | 39980 | -0.190576 | -0.6498272 | 0.26867524 | FALSE |
| 1 | 0 | 20 | 0 | 5 | 40092 | -0.1879042 | -0.8372471 | 0.46143882 | FALSE |
| 2 | 10 | 3 | 4 | 2 | 40098 | 0.38525002 | -0.1957683 | 0.96626834 | FALSE |
| 4 | 0 | 9 | 0 | 7 | 40170 | -0.1348466 | -0.4506349 | 0.18094165 | FALSE |
| 2 | 0 | 8 | 0 | 5 | 40317 | -0.1907137 | -0.601575 | 0.22014772 | FALSE |
| 3 | 0 | 30 | 0 | 7 | 40319 | -0.4159662 | -1.2994447 | 0.46751231 | FALSE |
| 7 | 5 | 65 | 3 | 9 | 40366 | -0.4669441 | -1.6720105 | 0.73812239 | FALSE |
| 3 | 0 | 41 | 0 | 10 | 40401 | -0.2054012 | -1.0677343 | 0.65693195 | FALSE |
| 2 | 508 | 1 | 4 | 1 | 40424 | 1.35199112 | -0.781068 | 3.48505023 | FALSE |
| 5 | 4 | 14 | 3 | 4 | 40453 | 0.05876136 | -0.7636613 | 0.88118401 | FALSE |
| 5 | 296 | 14 | 8 | 3 | 40469 | 1.81968234 | -0.0187125 | 3.65807718 | FALSE |
| 4 | 7 | 17 | 3 | 6 | 40547 | 0.0086779 | -0.7814907 | 0.79884648 | FALSE |
| 4 | 0 | 7 | 0 | 5 | 40559 | -0.0519718 | -0.2988422 | 0.19489863 | FALSE |
| 2 | 1 | 35 | 1 | 5 | 40567 | -0.4339205 | -1.3998141 | 0.53197316 | FALSE |
| 6 | 16 | 32 | 2 | 11 | 40578 | -0.1517108 | -1.0972042 | 0.79378254 | FALSE |
| 3 | 9 | 3 | 4 | 2 | 40584 | 0.3383281 | -0.2740203 | 0.95067654 | FALSE |
| 8 | 2 | 64 | 2 | 13 | 40587 | -0.5723393 | -1.5933687 | 0.44868998 | FALSE |
| 2 | 0 | 19 | 0 | 6 | 40680 | -0.3556908 | -1.0597133 | 0.3483316 | FALSE |
| 4 | 5 | 42 | 1 | 7 | 40688 | -0.2068271 | -1.1929104 | 0.77925623 | FALSE |
| 4 | 2 | 5 | 2 | 4 | 40711 | 0.02842788 | -0.2862283 | 0.34308402 | FALSE |
| 4 | 0 | 15 | 0 | 9 | 40713 | -0.1632507 | -0.65183 | 0.32532867 | FALSE |
| 4 | 1 | 5 | 1 | 5 | 40739 | -0.1151869 | -0.4057729 | 0.17539912 | FALSE |
| 1 | 0 | 23 | 0 | 6 | 40814 | -0.3146476 | -1.0419053 | 0.41261004 | FALSE |
| 1 | 0 | 21 | 0 | 5 | 40909 | -0.1830206 | -0.8694473 | 0.50340618 | FALSE |
| 1 | 0 | 8 | 0 | 5 | 40915 | -0.1124269 | -0.5185076 | 0.29365387 | FALSE |
| 2 | 0 | 135 | 0 | 8 | 40926 | -0.5049829 | -1.6213103 | 0.61134454 | FALSE |
| 2 | 0 | 97 | 0 | 9 | 40966 | -0.709932 | -1.7986607 | 0.37879663 | FALSE |
| 1 | 0 | 32 | 0 | 5 | 41099 | -0.1843411 | -1.0164828 | 0.64780056 | FALSE |
| 1 | 0 | 10 | 0 | 5 | 41144 | -0.1024665 | -0.5607033 | 0.35577027 | FALSE |
| 4 | 12 | 9 | 4 | 5 | 41256 | 0.2269556 | -0.5257059 | 0.97961708 | FALSE |
| 3 | 2 | 9 | 2 | 5 | 41319 | -0.1262431 | -0.6111027 | 0.35861654 | FALSE |
| 3 | 0 | 30 | 0 | 6 | 41677 | -0.3995977 | -1.208527 | 0.40933165 | FALSE |
| 4 | 7 | 13 | 4 | 5 | 41683 | 0.19462488 | -0.5758371 | 0.96508684 | FALSE |
| 2 | 143 | 18 | 4 | 3 | 41687 | 0.95289709 | -0.5436415 | 2.44943565 | FALSE |
| 5 | 7 | 6 | 5 | 5 | 41715 | 0.28653455 | -0.0658922 | 0.63896128 | FALSE |
| 9 | 11 | 52 | 7 | 15 | 41756 | -0.1621332 | -1.1604317 | 0.83616535 | FALSE |
| 5 | 2 | 50 | 2 | 10 | 41759 | -0.3020002 | -1.339192 | 0.73519173 | FALSE |
| 11 | 51 | 133 | 12 | 22 | 41809 | 0.50804683 | -0.5452368 | 1.56133042 | FALSE |
| 3 | 6 | 9 | 3 | 6 | 41826 | 0.27257682 | -0.3042477 | 0.8494013 | FALSE |
| 5 | 2 | 16 | 2 | 9 | 41842 | -0.0910288 | -0.6067191 | 0.42466156 | FALSE |
| 3 | 1 | 6 | 1 | 4 | 41843 | -0.0850573 | -0.4061068 | 0.2359923 | FALSE |
| 3 | 1 | 10 | 1 | 6 | 41941 | -0.1578639 | -0.5826144 | 0.26688661 | FALSE |
| 1 | 0 | 18 | 0 | 5 | 41955 | -0.2616719 | -0.9091485 | 0.3858047 | FALSE |
| 1 | 0 | 9 | 0 | 5 | 41976 | -0.1088876 | -0.5468992 | 0.32912406 | FALSE |
| 1 | 0 | 88 | 0 | 5 | 41987 | -0.2511288 | -1.3268181 | 0.82456048 | FALSE |
| 2 | 7 | 2 | 3 | 2 | 42103 | 0.19377154 | -0.2519964 | 0.63953943 | FALSE |
| 5 | 16 | 169 | 3 | 13 | 42288 | -0.2511022 | -2.0492593 | 1.54705486 | FALSE |
| 2 | 1 | 7 | 1 | 4 | 42298 | -0.0409647 | -0.439773 | 0.35784354 | FALSE |
| 3 | 6 | 4 | 3 | 3 | 42372 | 0.12414116 | -0.296239 | 0.54452135 | FALSE |
| 4 | 3 | 5 | 3 | 3 | 42377 | 0.17956121 | -0.1174207 | 0.47654309 | FALSE |
| 2 | 2 | 4 | 2 | 3 | 42378 | -0.007473 | -0.3657895 | 0.35084361 | FALSE |
| 3 | 2 | 10 | 1 | 6 | 42428 | 0.02130614 | -0.4390006 | 0.48161289 | FALSE |
| 11 | 8 | 176 | 4 | 25 | 42461 | -0.9219125 | -2.2124501 | 0.36862509 | FALSE |
| 3 | 3 | 4 | 1 | 4 | 42499 | -0.0104318 | -0.3122367 | 0.29137304 | FALSE |
| 5 | 0 | 175 | 0 | 17 | 42503 | -1.1247056 | -2.5460941 | 0.29668294 | FALSE |
| 4 | 2 | 5 | 2 | 4 | 42572 | -0.0172639 | -0.2881088 | 0.25358101 | FALSE |
| 2 | 0 | 11 | 0 | 5 | 42578 | -0.191031 | -0.6178497 | 0.23578767 | FALSE |
| 11 | 208 | 520 | 12 | 25 | 42605 | 0.1325143 | -2.0023054 | 2.26733405 | FALSE |
| 7 | 1 | 14 | 1 | 10 | 42625 | -0.1666258 | -0.5301799 | 0.1969283 | FALSE |
| 5 | 2 | 37 | 2 | 10 | 42651 | -0.0919873 | -0.8894887 | 0.70551421 | FALSE |
| 9 | 32 | 107 | 9 | 23 | 42700 | 0.32910083 | -0.8555759 | 1.51377753 | FALSE |
| 7 | 5 | 12 | 3 | 9 | 42712 | 0.05173072 | -0.3343381 | 0.43779959 | FALSE |
| 8 | 5 | 13 | 4 | 8 | 42740 | 0.04000395 | -0.4629002 | 0.54290807 | FALSE |
| 8 | 13 | 48 | 4 | 12 | 42743 | -0.2843043 | -1.2276593 | 0.65905068 | FALSE |
| 3 | 44 | 3 | 4 | 3 | 42769 | 0.64866997 | -0.4627674 | 1.76010738 | FALSE |
| 5 | 5 | 6 | 3 | 4 | 42827 | 0.20384778 | -0.1446003 | 0.55229588 | FALSE |
| 2 | 0 | 12 | 0 | 6 | 42831 | -0.1890503 | -0.6286644 | 0.25056373 | FALSE |
| 8 | 5 | 13 | 4 | 9 | 42832 | 0.15278898 | -0.2766929 | 0.58227084 | FALSE |
| 4 | 9 | 8 | 5 | 4 | 42839 | 0.4440737 | -0.0828168 | 0.97096417 | FALSE |
| 7 | 3 | 86 | 1 | 14 | 42911 | -0.720576 | -1.9119297 | 0.47077774 | FALSE |
| 2 | 1 | 11 | 1 | 4 | 43060 | 0.04898098 | -0.4579155 | 0.55587749 | FALSE |
| 4 | 1 | 10 | 1 | 6 | 43135 | -0.0713192 | -0.4907014 | 0.34806301 | FALSE |
| 4 | 0 | 9 | 0 | 6 | 43162 | -0.21406 | -0.5282175 | 0.10009745 | FALSE |
| 3 | 99 | 14 | 5 | 4 | 43289 | 1.18465912 | -0.2434476 | 2.61276583 | FALSE |
| 6 | 8 | 10 | 4 | 7 | 43432 | 0.16072201 | -0.3113434 | 0.63278737 | FALSE |
| 3 | 4 | 5 | 3 | 5 | 43448 | 0.1228751 | -0.255386 | 0.50113616 | FALSE |
| 3 | 0 | 10 | 0 | 5 | 43661 | -0.1812063 | -0.5087394 | 0.14632678 | FALSE |
| 3 | 2 | 5 | 2 | 3 | 43888 | 0.04505985 | -0.2964174 | 0.38653711 | FALSE |
| 4 | 0 | 6 | 0 | 5 | 44025 | -0.1785397 | -0.434882 | 0.07780251 | FALSE |
| 2 | 0 | 8 | 0 | 5 | 44156 | -0.1240992 | -0.4613295 | 0.21313109 | FALSE |
| 8 | 8 | 13 | 5 | 9 | 44325 | 0.08013689 | -0.437638 | 0.59791176 | FALSE |
| 4 | 2 | 29 | 2 | 6 | 44348 | -0.3181298 | -1.18571 | 0.54945033 | FALSE |
| 4 | 0 | 13 | 0 | 8 | 44360 | -0.2843943 | -0.6973924 | 0.12860384 | FALSE |
| 2 | 0 | 15 | 0 | 6 | 44393 | -0.2104715 | -0.836707 | 0.415764 | FALSE |
| 1 | 0 | 12 | 0 | 5 | 44398 | -0.2901799 | -0.8753668 | 0.29500706 | FALSE |
| 2 | 0 | 16 | 0 | 5 | 44449 | -0.0327527 | -0.5800482 | 0.51454272 | FALSE |
| 3 | 54 | 1 | 5 | 1 | 44466 | 0.99992833 | -0.1420677 | 2.14192435 | FALSE |
| 2 | 27 | 0 | 5 | 0 | 44520 | 0.61889766 | -0.1672258 | 1.4050211 | FALSE |
| 4 | 1 | 6 | 1 | 6 | 44580 | -0.0856453 | -0.4590401 | 0.28774941 | FALSE |
| 7 | 23 | 89 | 2 | 15 | 44591 | -0.258249 | -1.6083327 | 1.09183468 | FALSE |
| 6 | 0 | 32 | 0 | 12 | 44595 | -0.6047657 | -1.4144192 | 0.20488777 | FALSE |
| 4 | 1 | 8 | 1 | 5 | 44608 | -0.1300755 | -0.5002219 | 0.24007082 | FALSE |
| 5 | 4 | 15 | 3 | 6 | 44624 | -0.087856 | -0.6606029 | 0.48489093 | FALSE |
| 10 | 66 | 902 | 7 | 28 | 44633 | -1.262225 | -3.3499098 | 0.82545983 | FALSE |
| 4 | 0 | 12 | 0 | 7 | 44635 | -0.3051604 | -0.7172006 | 0.10687974 | FALSE |
| 9 | 7 | 41 | 4 | 14 | 44636 | -0.045442 | -0.8288421 | 0.73795802 | FALSE |
| 3 | 0 | 20 | 0 | 5 | 44645 | -0.207469 | -0.6870951 | 0.27215711 | FALSE |
| 3 | 2 | 6 | 2 | 3 | 44703 | -0.0336261 | -0.3678887 | 0.30063648 | FALSE |
| 6 | 1 | 17 | 1 | 8 | 44721 | -0.3017569 | -0.8705071 | 0.26699332 | FALSE |
| 5 | 4 | 18 | 1 | 7 | 44723 | -0.148916 | -0.6836779 | 0.38584591 | FALSE |
| 9 | 57 | 107 | 5 | 22 | 44740 | -0.0702605 | -1.3658754 | 1.22535446 | FALSE |
| 4 | 3 | 7 | 2 | 4 | 44795 | -0.0134932 | -0.3744208 | 0.34743431 | FALSE |
| 5 | 2 | 67 | 1 | 9 | 44809 | -0.6789618 | -1.8624541 | 0.50453058 | FALSE |
| 3 | 0 | 35 | 0 | 8 | 44813 | -0.5751935 | -1.4612114 | 0.31082442 | FALSE |
| 1 | 0 | 12 | 0 | 5 | 44922 | -0.2121619 | -0.695258 | 0.27093425 | FALSE |
| 3 | 0 | 6 | 0 | 5 | 44965 | -0.1606009 | -0.4215314 | 0.1003295 | FALSE |
| 2 | 0 | 33 | 0 | 6 | 45078 | -0.4144528 | -1.0603257 | 0.23142002 | FALSE |
| 5 | 12 | 23 | 4 | 8 | 45175 | 0.09170204 | -0.7410106 | 0.92441471 | FALSE |
| 4 | 4 | 55 | 3 | 10 | 45196 | -0.5769201 | -1.6989564 | 0.54511621 | FALSE |
| 3 | 1 | 12 | 1 | 4 | 45201 | -0.1998263 | -0.6379802 | 0.23832761 | FALSE |
| 3 | 1 | 7 | 1 | 4 | 45290 | -0.1591685 | -0.5728194 | 0.25448236 | FALSE |
| 4 | 3 | 4 | 3 | 2 | 45314 | 0.10952975 | -0.1527472 | 0.37180669 | FALSE |
| 1 | 0 | 42 | 0 | 6 | 45438 | -0.3174109 | -1.2907446 | 0.6559228 | FALSE |
| 1 | 0 | 12 | 0 | 5 | 45467 | -0.1020126 | -0.6127711 | 0.40874595 | FALSE |
| 4 | 4 | 7 | 1 | 4 | 45478 | 0.1256716 | -0.2776794 | 0.52902263 | FALSE |
| 2 | 0 | 59 | 0 | 9 | 45730 | -0.710845 | -1.5774756 | 0.1557856 | FALSE |
| 4 | 5 | 11 | 4 | 3 | 45732 | 0.06685901 | -0.4629205 | 0.59663852 | FALSE |
| 3 | 3 | 32 | 1 | 5 | 45742 | -0.1766553 | -0.7390038 | 0.3856933 | FALSE |
| 6 | 5 | 4 | 4 | 4 | 45743 | 0.09315601 | -0.2289878 | 0.4152998 | FALSE |
| 8 | 3 | 26 | 3 | 11 | 45938 | -0.1831437 | -0.7092628 | 0.34297541 | FALSE |
| 4 | 2 | 8 | 2 | 6 | 45954 | -0.0452911 | -0.4461228 | 0.35554069 | FALSE |
| 10 | 64 | 121 | 12 | 19 | 45955 | 1.14199194 | -0.227961 | 2.51194483 | FALSE |
| 9 | 45 | 39 | 9 | 11 | 46005 | 0.80776677 | -0.1762219 | 1.79175546 | FALSE |
| 4 | 0 | 7 | 0 | 6 | 46006 | -0.1603969 | -0.4053093 | 0.08451539 | FALSE |
| 5 | 3 | 8 | 2 | 4 | 46010 | 0.03674922 | -0.3005401 | 0.37403849 | FALSE |
| 6 | 10 | 17 | 4 | 8 | 46012 | 0.30566139 | -0.3329004 | 0.94422314 | FALSE |
| 9 | 4 | 55 | 4 | 17 | 46030 | -0.3919249 | -1.1226071 | 0.33875721 | FALSE |
| 5 | 5 | 32 | 4 | 9 | 46067 | -0.0514103 | -0.8544089 | 0.75158841 | FALSE |
| 2 | 40 | 5 | 3 | 4 | 46069 | 0.67758986 | -0.1525015 | 1.5076812 | FALSE |
| 4 | 7 | 7 | 3 | 4 | 46089 | 0.31964474 | -0.1773323 | 0.81662176 | FALSE |
| 3 | 1 | 8 | 1 | 5 | 46146 | -0.0982182 | -0.4918389 | 0.29540261 | FALSE |
| 3 | 1 | 12 | 1 | 6 | 46155 | -0.1304869 | -0.6133495 | 0.35237577 | FALSE |
| 1 | 0 | 22 | 0 | 5 | 46176 | -0.3327754 | -0.9715366 | 0.30598578 | FALSE |
| 5 | 2 | 76 | 2 | 13 | 46267 | -0.7834914 | -1.9865025 | 0.41951971 | FALSE |
| 2 | 47 | 15 | 2 | 4 | 46474 | 0.67875209 | -0.6816994 | 2.03920359 | FALSE |
| 3 | 1 | 27 | 1 | 6 | 46512 | 0.02745103 | -0.7276934 | 0.78259542 | FALSE |
| 3 | 12 | 132 | 2 | 7 | 46520 | 0.18961222 | -1.2308216 | 1.61004608 | FALSE |
| 4 | 11 | 8 | 6 | 4 | 46538 | 0.51155932 | -0.0307281 | 1.05384669 | FALSE |
| 6 | 4 | 11 | 2 | 7 | 46600 | 0.12706552 | -0.3275297 | 0.58166077 | FALSE |
| 3 | 1 | 6 | 1 | 5 | 46618 | -0.1131793 | -0.4401241 | 0.21376556 | FALSE |
| 6 | 5 | 14 | 3 | 6 | 46632 | 0.06092035 | -0.3851287 | 0.50696945 | FALSE |
| 5 | 73 | 1195 | 5 | 15 | 46673 | -0.1837729 | -3.2128684 | 2.84532262 | FALSE |
| 4 | 174 | 453 | 5 | 11 | 46694 | 0.47167372 | -2.0032708 | 2.94661825 | FALSE |
| 3 | 521 | 1 | 8 | 1 | 46783 | 1.62112392 | -0.2933802 | 3.53562804 | FALSE |
| 5 | 4 | 7 | 4 | 5 | 46824 | 0.07941955 | -0.3220213 | 0.48086044 | FALSE |
| 9 | 55 | 137 | 9 | 20 | 46879 | 0.69226538 | -0.6820741 | 2.0666049 | FALSE |
| 4 | 1 | 42 | 1 | 10 | 46933 | -0.3192658 | -1.0972025 | 0.45867088 | FALSE |
| 11 | 11 | 215 | 5 | 26 | 46950 | -0.4974464 | -1.8331319 | 0.83823913 | FALSE |
| 5 | 1 | 14 | 1 | 6 | 46953 | -0.0912799 | -0.5522794 | 0.36971956 | FALSE |
| 3 | 0 | 9 | 0 | 6 | 46968 | -0.0858002 | -0.4270601 | 0.25545975 | FALSE |
| 7 | 4 | 20 | 3 | 9 | 46969 | 0.02710712 | -0.4437515 | 0.49796569 | FALSE |
| 3 | 2 | 6 | 2 | 5 | 47006 | -0.0220651 | -0.3595681 | 0.31543781 | FALSE |
| 10 | 58 | 129 | 14 | 19 | 47042 | 1.10721734 | -0.1433891 | 2.35782383 | FALSE |
| 3 | 0 | 8 | 0 | 5 | 47045 | -0.1564616 | -0.4621324 | 0.14920909 | FALSE |
| 7 | 8 | 48 | 2 | 13 | 47049 | 0.0142641 | -0.9565034 | 0.98503157 | FALSE |
| 10 | 14 | 51 | 8 | 15 | 47063 | -0.1568502 | -1.1303272 | 0.81662679 | FALSE |
| 5 | 4 | 11 | 3 | 8 | 47093 | -0.0146332 | -0.5646436 | 0.5353772 | FALSE |
| 11 | 48 | 117 | 12 | 21 | 47099 | 0.13955501 | -0.9935229 | 1.27263296 | FALSE |
| 2 | 1 | 5 | 1 | 4 | 47115 | -0.0222265 | -0.3424676 | 0.29801468 | FALSE |
| 3 | 0 | 6 | 0 | 5 | 47186 | -0.0793787 | -0.3586092 | 0.19985167 | FALSE |
| 2 | 6 | 11 | 3 | 3 | 47194 | -0.0176682 | -0.8089688 | 0.77363231 | FALSE |
| 4 | 3 | 11 | 2 | 4 | 47196 | 0.04508084 | -0.3829568 | 0.47311852 | FALSE |
| 4 | 0 | 8 | 0 | 6 | 47564 | -0.2622651 | -0.6227202 | 0.09819006 | FALSE |
| 2 | 8 | 5 | 3 | 4 | 47799 | 0.1966325 | -0.3123494 | 0.70561442 | FALSE |
| 6 | 5 | 11 | 4 | 8 | 47873 | -0.0802686 | -0.5710743 | 0.41053714 | FALSE |
| 5 | 3 | 6 | 3 | 4 | 48808 | 0.05120251 | -0.256087 | 0.35849203 | FALSE |
| 5 | 2 | 15 | 1 | 7 | 48829 | -0.1084038 | -0.5373712 | 0.32056352 | FALSE |
| 2 | 5 | 2 | 3 | 2 | 49097 | 0.1946252 | -0.1811723 | 0.57042273 | FALSE |
| 7 | 1 | 162 | 1 | 17 | 49175 | -1.4156928 | -2.8765357 | 0.0451501 | FALSE |
| 5 | 2 | 4 | 2 | 3 | 49200 | 0.0698796 | -0.1670551 | 0.30681428 | FALSE |
| 3 | 1 | 7 | 1 | 4 | 49214 | -0.0746678 | -0.4453423 | 0.29600667 | FALSE |
| 4 | 7 | 19 | 3 | 7 | 49222 | 0.01174973 | -0.8621793 | 0.88567873 | FALSE |
| 6 | 0 | 66 | 0 | 13 | 49227 | -0.5606261 | -1.4907515 | 0.36949924 | FALSE |
| 4 | 1 | 8 | 1 | 6 | 49243 | -0.0994442 | -0.4517529 | 0.25286453 | FALSE |
| 5 | 0 | 220 | 0 | 11 | 49250 | -0.6853691 | -2.1568558 | 0.78611759 | FALSE |
| 2 | 0 | 24 | 0 | 6 | 49251 | -0.3772497 | -1.1623979 | 0.40789848 | FALSE |
| 3 | 0 | 15 | 0 | 5 | 49271 | -0.28798 | -0.7858165 | 0.20985659 | FALSE |
| 1 | 0 | 8 | 0 | 5 | 49363 | -0.1270936 | -0.5662184 | 0.31203114 | FALSE |
| 3 | 0 | 24 | 0 | 7 | 49364 | -0.2911501 | -1.0902505 | 0.5079503 | FALSE |
| 7 | 6 | 36 | 3 | 11 | 49395 | -0.2128118 | -1.201286 | 0.77566249 | FALSE |
| 2 | 1 | 10 | 1 | 5 | 49423 | -0.0509746 | -0.5755092 | 0.47356003 | FALSE |
| 1 | 0 | 9 | 0 | 5 | 49874 | -0.2182353 | -0.6578088 | 0.22133823 | FALSE |
| 4 | 8 | 13 | 5 | 4 | 49957 | 0.12358958 | -0.6008152 | 0.84799438 | FALSE |
| 1 | 0 | 24 | 0 | 5 | 50028 | -0.1842503 | -0.8795548 | 0.51105425 | FALSE |
| 2 | 0 | 24 | 0 | 5 | 50349 | -0.4478419 | -1.3540642 | 0.45838038 | FALSE |
| 6 | 0 | 80 | 0 | 14 | 50408 | -0.9958701 | -2.1528298 | 0.16108966 | FALSE |
| 8 | 15 | 61 | 8 | 18 | 50455 | 0.1340312 | -0.8040425 | 1.07210485 | FALSE |
| 3 | 0 | 19 | 0 | 7 | 50606 | -0.4077386 | -1.0621702 | 0.2466929 | FALSE |
| 5 | 5 | 10 | 4 | 5 | 50635 | 0.31382208 | -0.1723888 | 0.80003292 | FALSE |
| 5 | 1 | 13 | 1 | 5 | 50660 | -0.2527594 | -0.6362482 | 0.1307294 | FALSE |
| 5 | 3 | 19 | 1 | 11 | 50663 | -0.2888009 | -0.8556413 | 0.27803957 | FALSE |
| 11 | 33 | 65 | 12 | 16 | 50667 | 0.89668218 | -0.1018014 | 1.89516573 | FALSE |
| 4 | 3 | 27 | 2 | 8 | 50688 | -0.2383219 | -0.9499764 | 0.47333263 | FALSE |
| 7 | 3 | 28 | 2 | 13 | 50703 | -0.3138092 | -0.9297265 | 0.302108 | FALSE |
| 7 | 8 | 18 | 4 | 13 | 50707 | -0.0391522 | -0.6773335 | 0.59902909 | FALSE |
| 9 | 55 | 95 | 9 | 18 | 50776 | 0.45777956 | -1.0369071 | 1.95246617 | FALSE |
| 4 | 5 | 10 | 3 | 5 | 50782 | 0.14990444 | -0.3521933 | 0.65200223 | FALSE |
| 2 | 1 | 6 | 1 | 4 | 50881 | -0.007308 | -0.3675153 | 0.35289926 | FALSE |
| 2 | 0 | 14 | 0 | 6 | 50997 | -0.1361612 | -0.6436397 | 0.37131739 | FALSE |
| 3 | 0 | 20 | 0 | 9 | 51015 | -0.4002305 | -1.0581555 | 0.25769448 | FALSE |
| 2 | 0 | 21 | 0 | 6 | 51313 | -0.0582768 | -0.626201 | 0.50964747 | FALSE |
| 2 | 12 | 10 | 2 | 4 | 51350 | 0.15344675 | -0.5689192 | 0.87581271 | FALSE |
| 5 | 13 | 3 | 4 | 3 | 51353 | 0.4532623 | -0.0023604 | 0.908885 | FALSE |
| 2 | 0 | 96 | 0 | 7 | 51366 | -0.4044216 | -1.5666547 | 0.75781142 | FALSE |
| 3 | 1 | 5 | 1 | 4 | 51409 | 0.05747526 | -0.2303696 | 0.34532012 | FALSE |
| 4 | 1 | 55 | 1 | 9 | 51442 | -0.3353366 | -1.3140198 | 0.64334666 | FALSE |
| 7 | 2 | 15 | 2 | 7 | 51479 | 0.06684802 | -0.3666301 | 0.50032619 | FALSE |
| 3 | 5 | 11 | 2 | 4 | 51507 | 0.13645856 | -0.4182811 | 0.69119826 | FALSE |
| 5 | 3 | 107 | 3 | 8 | 51542 | -0.1663702 | -1.4217673 | 1.08902691 | FALSE |
| 5 | 11 | 342 | 4 | 12 | 51555 | -0.1924098 | -2.2324255 | 1.84760597 | FALSE |
| 5 | 58 | 229 | 4 | 15 | 51558 | 0.08650386 | -1.7653571 | 1.93836482 | FALSE |
| 3 | 148 | 187 | 4 | 12 | 51580 | 0.55681966 | -1.4860488 | 2.59968813 | FALSE |
| 10 | 58 | 67 | 10 | 17 | 51665 | 0.68892636 | -0.3702919 | 1.74814457 | FALSE |
| 7 | 21 | 8 | 6 | 6 | 51667 | 0.32682537 | -0.2914359 | 0.9450866 | FALSE |
| 5 | 1 | 7 | 1 | 5 | 51679 | -0.1495468 | -0.4245952 | 0.12550154 | FALSE |
| 3 | 0 | 7 | 0 | 5 | 51685 | -0.153084 | -0.4727232 | 0.16655514 | FALSE |
| 8 | 76 | 47 | 9 | 13 | 51691 | 0.77055702 | -0.4825288 | 2.02364285 | FALSE |
| 5 | 3 | 7 | 2 | 4 | 51713 | 0.12978703 | -0.1842083 | 0.44378236 | FALSE |
| 5 | 9 | 6 | 4 | 5 | 51814 | 0.23451562 | -0.1690377 | 0.63806897 | FALSE |
| 6 | 6 | 24 | 5 | 5 | 51824 | 0.17160238 | -0.5018355 | 0.84504025 | FALSE |
| 4 | 2 | 14 | 1 | 6 | 51826 | -0.0609216 | -0.56143 | 0.43958691 | FALSE |
| 7 | 9 | 12 | 6 | 9 | 51831 | 0.44838949 | -0.067631 | 0.96440995 | FALSE |
| 8 | 10 | 32 | 3 | 11 | 51887 | -0.0650658 | -0.8434827 | 0.7133511 | FALSE |
| 6 | 1 | 37 | 1 | 12 | 51893 | -0.3418864 | -0.9729404 | 0.28916757 | FALSE |
| 9 | 11 | 19 | 5 | 12 | 51920 | 0.08164409 | -0.3998963 | 0.56318447 | FALSE |
| 9 | 6 | 11 | 6 | 8 | 51929 | 0.13404086 | -0.2096031 | 0.47768486 | FALSE |
| 7 | 7 | 27 | 3 | 8 | 51937 | 0.07436364 | -0.5510223 | 0.69974962 | FALSE |
| 6 | 2 | 8 | 2 | 6 | 51946 | -0.015972 | -0.3158834 | 0.28393941 | FALSE |
| 2 | 2 | 6 | 2 | 4 | 51973 | -0.0634882 | -0.4435207 | 0.31654433 | FALSE |
| 12 | 21 | 55 | 7 | 18 | 51991 | 0.05796069 | -0.8219132 | 0.93783459 | FALSE |
| 7 | 6 | 21 | 4 | 11 | 51994 | -0.1235275 | -0.7944711 | 0.54741615 | FALSE |
| 2 | 0 | 10 | 0 | 5 | 52018 | -0.2004774 | -0.6615723 | 0.26061746 | FALSE |
| 10 | 11 | 43 | 5 | 19 | 52032 | -0.1520849 | -0.8946213 | 0.59045144 | FALSE |
| 6 | 8 | 9 | 3 | 6 | 52069 | 0.24465593 | -0.1702743 | 0.65958614 | FALSE |
| 12 | 124 | 586 | 13 | 31 | 52116 | -0.2181494 | -1.8392863 | 1.40298757 | FALSE |
| 7 | 6 | 7 | 5 | 6 | 52129 | 0.15347609 | -0.2012112 | 0.50816338 | FALSE |
| 5 | 2 | 7 | 2 | 4 | 52132 | -0.001841 | -0.3053474 | 0.30166537 | FALSE |
| 8 | 7 | 39 | 4 | 13 | 52230 | 0.01146204 | -0.7419557 | 0.76487978 | FALSE |
| 5 | 3 | 5 | 3 | 3 | 52362 | 0.17423421 | -0.1118188 | 0.46028725 | FALSE |
| 5 | 0 | 23 | 0 | 12 | 52622 | -0.4971982 | -1.0391736 | 0.04477716 | FALSE |
| 4 | 0 | 15 | 0 | 7 | 52629 | -0.2544522 | -0.7781349 | 0.26923037 | FALSE |
| 5 | 3 | 12 | 2 | 7 | 52758 | -0.2651386 | -0.7503192 | 0.22004199 | FALSE |
| 6 | 16 | 13 | 6 | 9 | 52776 | 0.3901355 | -0.238031 | 1.01830204 | FALSE |
| 3 | 2 | 6 | 2 | 4 | 52993 | 0.05686834 | -0.3223459 | 0.43608257 | FALSE |
| 3 | 0 | 6 | 0 | 5 | 53066 | -0.1047268 | -0.4050824 | 0.19562868 | FALSE |
| 3 | 26 | 2 | 5 | 2 | 53177 | 0.57652756 | -0.2108664 | 1.36392149 | FALSE |
| 4 | 0 | 15 | 0 | 8 | 53259 | -0.3831952 | -0.8509602 | 0.08456973 | FALSE |
| 4 | 2 | 75 | 1 | 8 | 53292 | -0.5142221 | -1.7362893 | 0.70784518 | FALSE |
| 7 | 1 | 43 | 1 | 13 | 53323 | -0.6679154 | -1.5439637 | 0.20813293 | FALSE |
| 6 | 3 | 21 | 1 | 11 | 53412 | -0.1922548 | -0.7780104 | 0.39350084 | FALSE |
| 3 | 2 | 6 | 2 | 4 | 53474 | -0.0038151 | -0.4073945 | 0.39976425 | FALSE |
| 3 | 4 | 3 | 3 | 3 | 53480 | 0.06513544 | -0.2784074 | 0.40867832 | FALSE |
| 3 | 1 | 6 | 1 | 4 | 53482 | -0.0597688 | -0.406202 | 0.28666437 | FALSE |
| 2 | 0 | 11 | 0 | 5 | 53493 | -0.351794 | -0.8673051 | 0.16371713 | FALSE |
| 4 | 6 | 10 | 5 | 3 | 53579 | 0.24042841 | -0.4181817 | 0.89903857 | FALSE |
| 3 | 6 | 24 | 1 | 7 | 53616 | -0.2167734 | -1.0772821 | 0.64373529 | FALSE |
| 3 | 0 | 6 | 0 | 5 | 53625 | -0.1801236 | -0.470571 | 0.11032381 | FALSE |
| 6 | 5 | 35 | 2 | 13 | 53649 | -0.1417033 | -1.0977362 | 0.8143297 | FALSE |
| 2 | 0 | 8 | 0 | 5 | 53656 | -0.2644168 | -0.6779327 | 0.14909902 | FALSE |
| 4 | 2 | 29 | 1 | 6 | 53665 | -0.3017853 | -1.0876227 | 0.48405212 | FALSE |
| 3 | 0 | 19 | 0 | 5 | 53668 | -0.320986 | -0.9115319 | 0.26955984 | FALSE |
| 1 | 0 | 32 | 0 | 5 | 53704 | -0.3155017 | -1.1191388 | 0.48813543 | FALSE |
| 3 | 1 | 16 | 1 | 5 | 53757 | -0.2344784 | -0.848438 | 0.37948113 | FALSE |
| 7 | 5 | 12 | 4 | 7 | 53774 | -0.0329068 | -0.5401486 | 0.47433489 | FALSE |
| 4 | 0 | 17 | 0 | 5 | 53775 | -0.2877029 | -0.9330986 | 0.35769266 | FALSE |
| 4 | 1 | 17 | 1 | 9 | 53777 | -0.3096479 | -0.8898825 | 0.27058673 | FALSE |
| 5 | 1 | 60 | 1 | 7 | 53788 | -0.3214336 | -1.3440041 | 0.70113698 | FALSE |
| 3 | 0 | 59 | 0 | 7 | 53989 | -0.5735005 | -1.2864758 | 0.13947475 | FALSE |
| 2 | 0 | 7 | 0 | 5 | 54205 | -0.2142305 | -0.5882149 | 0.15975387 | FALSE |
| 3 | 1 | 8 | 1 | 4 | 54572 | -0.1249098 | -0.4619278 | 0.21210829 | FALSE |
| 2 | 0 | 63 | 0 | 6 | 54587 | -0.4732332 | -1.6424623 | 0.69599602 | FALSE |
| 3 | 4 | 4 | 2 | 3 | 54690 | 0.19676473 | -0.1223617 | 0.51589113 | FALSE |
| 2 | 0 | 16 | 0 | 5 | 54779 | -0.2737836 | -0.8715125 | 0.32394521 | FALSE |
| 6 | 28 | 39 | 2 | 9 | 54828 | -0.0386218 | -0.910167 | 0.83292341 | FALSE |
| 11 | 30 | 82 | 13 | 19 | 54845 | 0.53098355 | -0.6106562 | 1.67262328 | FALSE |
| 9 | 19 | 66 | 9 | 14 | 54861 | 0.15998569 | -0.8684955 | 1.18846692 | FALSE |
| 6 | 5 | 10 | 4 | 7 | 54940 | 0.16830998 | -0.2107608 | 0.54738075 | FALSE |
| 3 | 2 | 5 | 2 | 3 | 54954 | -0.0120551 | -0.3024756 | 0.27836536 | FALSE |
| 2 | 11 | 19 | 2 | 4 | 54960 | 0.19659061 | -0.5771922 | 0.97037345 | FALSE |
| 4 | 1 | 6 | 1 | 4 | 55013 | -0.0980372 | -0.372748 | 0.1766737 | FALSE |
| 8 | 28 | 55 | 5 | 18 | 55018 | 0.15002706 | -0.8230181 | 1.12307225 | FALSE |
| 4 | 1 | 8 | 1 | 5 | 55019 | -0.1141941 | -0.4714307 | 0.24304244 | FALSE |
| 5 | 1 | 11 | 1 | 5 | 55028 | -0.0856461 | -0.4318758 | 0.26058359 | FALSE |
| 4 | 1 | 5 | 1 | 4 | 55048 | -0.0204935 | -0.2579924 | 0.21700545 | FALSE |
| 4 | 2 | 5 | 2 | 4 | 55076 | 0.04201195 | -0.2385713 | 0.32259521 | FALSE |
| 4 | 3 | 7 | 2 | 4 | 55104 | 0.18269762 | -0.3827796 | 0.74817489 | FALSE |
| 2 | 3 | 60 | 3 | 5 | 55170 | -0.4136338 | -1.6715175 | 0.84424997 | FALSE |
| 3 | 4 | 3 | 2 | 3 | 55216 | 0.25981815 | -0.2310236 | 0.75065986 | FALSE |
| 4 | 2 | 11 | 2 | 5 | 55253 | 0.04793254 | -0.3544932 | 0.45035825 | FALSE |
| 5 | 2 | 26 | 1 | 8 | 55300 | 0.10228586 | -0.6039061 | 0.80847786 | FALSE |
| 3 | 1 | 10 | 1 | 4 | 55304 | -0.0061693 | -0.3709828 | 0.35864415 | FALSE |
| 2 | 6 | 2 | 3 | 2 | 55425 | 0.28714421 | -0.1125253 | 0.68681373 | FALSE |
| 5 | 2 | 6 | 2 | 4 | 55667 | -0.014928 | -0.293872 | 0.26401607 | FALSE |
| 5 | 4 | 7 | 2 | 5 | 55841 | 0.16653368 | -0.2036719 | 0.53673932 | FALSE |
| 3 | 2 | 31 | 1 | 4 | 55875 | 0.0577206 | -0.5318375 | 0.64727865 | FALSE |
| 4 | 2 | 40 | 2 | 11 | 55886 | -0.1078323 | -0.9076455 | 0.69198102 | FALSE |
| 2 | 2 | 5 | 2 | 4 | 55953 | 0.04321436 | -0.3024118 | 0.38884055 | FALSE |
| 4 | 4 | 3 | 2 | 3 | 55995 | 0.18626038 | -0.0825408 | 0.4550616 | FALSE |
| 3 | 2 | 4 | 2 | 4 | 56142 | 0.0318982 | -0.2422622 | 0.30605858 | FALSE |
| 4 | 3 | 13 | 1 | 6 | 56156 | -0.131974 | -0.5864589 | 0.32251085 | FALSE |
| 5 | 3 | 7 | 2 | 6 | 56163 | 0.00442607 | -0.3188416 | 0.32769375 | FALSE |
| 3 | 0 | 7 | 0 | 6 | 56194 | -0.1594303 | -0.4233996 | 0.10453896 | FALSE |
| 3 | 2 | 5 | 2 | 3 | 56209 | 0.01580911 | -0.3862742 | 0.41789246 | FALSE |
| 3 | 0 | 17 | 0 | 10 | 56218 | -0.2643867 | -0.7360149 | 0.20724144 | FALSE |
| 2 | 4 | 3 | 3 | 3 | 56233 | 0.16494844 | -0.1917523 | 0.52164914 | FALSE |
| 2 | 0 | 7 | 0 | 5 | 56427 | -0.0897353 | -0.4158903 | 0.23641969 | FALSE |
| 4 | 1 | 8 | 1 | 6 | 56462 | -0.0728155 | -0.4605822 | 0.31495121 | FALSE |
| 6 | 3 | 14 | 2 | 6 | 56560 | -0.0410181 | -0.4881175 | 0.40608136 | FALSE |
| 9 | 48 | 92 | 6 | 16 | 56586 | 0.09136998 | -1.3377647 | 1.52050471 | FALSE |
| 4 | 1 | 7 | 1 | 6 | 56592 | -0.0219427 | -0.3491601 | 0.30527465 | FALSE |
| 3 | 12 | 3 | 3 | 2 | 56604 | 0.27930877 | -0.2936211 | 0.85223864 | FALSE |
| 3 | 0 | 10 | 0 | 6 | 56606 | -0.2956767 | -0.8093978 | 0.2180443 | FALSE |
| 6 | 10 | 12 | 5 | 8 | 56610 | 0.09448605 | -0.4129555 | 0.60192764 | FALSE |
| 6 | 8 | 14 | 4 | 11 | 56611 | 0.02057219 | -0.5597004 | 0.60084474 | FALSE |
| 5 | 3 | 11 | 2 | 6 | 56637 | 0.02974719 | -0.5323836 | 0.59187798 | FALSE |
| 9 | 12 | 35 | 6 | 14 | 56710 | 0.30860268 | -0.3745679 | 0.99177331 | FALSE |
| 11 | 6 | 29 | 5 | 11 | 56711 | -0.167617 | -0.7059824 | 0.3707484 | FALSE |
| 5 | 0 | 24 | 0 | 11 | 56712 | -0.4337178 | -1.0200381 | 0.15260253 | FALSE |
| 4 | 4 | 7 | 2 | 6 | 56724 | 0.15656428 | -0.3030603 | 0.61618886 | FALSE |
| 2 | 0 | 7 | 0 | 5 | 56748 | -0.1276741 | -0.4964862 | 0.24113798 | FALSE |
| 6 | 10 | 22 | 5 | 6 | 56755 | 0.36196623 | -0.2138604 | 0.9377929 | FALSE |
| 7 | 6 | 23 | 5 | 13 | 56767 | -0.1393798 | -0.7569101 | 0.47815054 | FALSE |
| 2 | 0 | 35 | 0 | 6 | 56813 | -0.1511604 | -0.9659594 | 0.66363852 | FALSE |
| 4 | 0 | 6 | 0 | 6 | 56820 | -0.1848756 | -0.4051004 | 0.03534919 | FALSE |
| 6 | 2 | 14 | 2 | 8 | 56825 | -0.1642858 | -0.6211632 | 0.29259165 | FALSE |
| 6 | 6 | 11 | 4 | 6 | 56833 | 0.19023278 | -0.3024978 | 0.68296333 | FALSE |
| 4 | 4 | 5 | 1 | 5 | 56843 | 0.32765011 | -0.3741933 | 1.02949354 | FALSE |
| 6 | 2 | 8 | 2 | 6 | 56848 | -0.1318669 | -0.4501854 | 0.1864516 | FALSE |
| 4 | 1 | 9 | 1 | 7 | 56866 | 0.0041667 | -0.3405389 | 0.34887229 | FALSE |
| 4 | 2 | 4 | 2 | 4 | 56873 | 0.00478482 | -0.2719504 | 0.28152008 | FALSE |
| 4 | 2 | 27 | 1 | 8 | 56890 | -0.2594831 | -1.0561218 | 0.5371556 | FALSE |
| 3 | 0 | 36 | 0 | 7 | 56989 | -0.2748556 | -1.1162904 | 0.56657919 | FALSE |
| 2 | 31 | 0 | 5 | 0 | 57205 | 0.6437746 | -0.1341908 | 1.42174002 | FALSE |
| 4 | 0 | 53 | 0 | 12 | 57220 | -0.8935632 | -1.9989498 | 0.21182342 | FALSE |
| 4 | 2 | 144 | 1 | 10 | 57386 | -0.9049776 | -2.4404413 | 0.63048605 | FALSE |
| 2 | 114 | 0 | 5 | 0 | 57536 | 1.12554038 | -0.3479605 | 2.59904127 | FALSE |
| 3 | 0 | 11 | 0 | 5 | 57594 | -0.3194725 | -0.9621105 | 0.32316553 | FALSE |
| 3 | 1 | 6 | 1 | 4 | 57599 | -0.0602268 | -0.3768241 | 0.25637048 | FALSE |
| 5 | 3 | 5 | 3 | 3 | 57616 | 0.11108664 | -0.176803 | 0.39897631 | FALSE |
| 8 | 24 | 42 | 9 | 12 | 57638 | 0.46795179 | -0.3887252 | 1.32462879 | FALSE |
| 1 | 0 | 11 | 0 | 5 | 57667 | -0.2026867 | -0.6741582 | 0.26878469 | FALSE |
| 3 | 1 | 5 | 1 | 4 | 57677 | -0.0756696 | -0.3524329 | 0.2010937 | FALSE |
| 6 | 9 | 16 | 5 | 7 | 57699 | 0.16355007 | -0.4053713 | 0.73247144 | FALSE |
| 3 | 8 | 1 | 4 | 1 | 57760 | 0.33642625 | -0.0612157 | 0.7340682 | FALSE |
| 4 | 15 | 18 | 4 | 6 | 57778 | 0.19751571 | -0.6234189 | 1.01845027 | FALSE |
| 4 | 0 | 9 | 0 | 6 | 57966 | -0.1758163 | -0.5883638 | 0.23673123 | FALSE |
| 3 | 0 | 17 | 0 | 6 | 58055 | -0.2123833 | -0.7822709 | 0.35750435 | FALSE |
| 7 | 3 | 110 | 2 | 15 | 58060 | -0.9691626 | -2.2915183 | 0.35319314 | FALSE |
| 5 | 1 | 39 | 1 | 9 | 58068 | -0.4595728 | -1.3980558 | 0.47891018 | FALSE |
| 6 | 1 | 106 | 1 | 11 | 58112 | -0.9875651 | -2.456631 | 0.48150075 | FALSE |
| 5 | 0 | 201 | 0 | 19 | 58125 | -1.568941 | -3.3177009 | 0.17981896 | FALSE |
| 3 | 9 | 2 | 3 | 2 | 58164 | 0.26468204 | -0.3369981 | 0.86636221 | FALSE |
| 6 | 5 | 23 | 3 | 10 | 58172 | -0.1170131 | -0.7957451 | 0.56171897 | FALSE |
| 4 | 0 | 11 | 0 | 7 | 58173 | -0.1034813 | -0.4611017 | 0.25413909 | FALSE |
| 3 | 0 | 15 | 0 | 6 | 58218 | -0.24855 | -0.8452959 | 0.34819603 | FALSE |
| 7 | 1 | 68 | 1 | 16 | 58394 | -0.6467551 | -1.6040187 | 0.31050859 | FALSE |
| 2 | 0 | 27 | 0 | 7 | 58427 | -0.4606227 | -1.2697504 | 0.34850499 | FALSE |
| 6 | 1 | 141 | 1 | 13 | 58437 | -0.6816837 | -1.9991805 | 0.63581314 | FALSE |
| 5 | 1 | 7 | 1 | 5 | 58444 | -0.020271 | -0.2988175 | 0.25827545 | FALSE |
| 3 | 1 | 5 | 1 | 4 | 58493 | -0.0788096 | -0.3784745 | 0.22085538 | FALSE |
| 4 | 1 | 5 | 1 | 5 | 58518 | -0.0938589 | -0.3678864 | 0.18016855 | FALSE |
| 1 | 0 | 7 | 0 | 5 | 58524 | -0.1823105 | -0.5986183 | 0.23399731 | FALSE |
| 3 | 0 | 22 | 0 | 7 | 58538 | -0.392251 | -1.1569899 | 0.37248795 | FALSE |
| 2 | 0 | 9 | 0 | 5 | 58539 | -0.2218993 | -0.6732965 | 0.22949788 | FALSE |
| 3 | 4 | 3 | 2 | 3 | 58554 | 0.12985784 | -0.1838512 | 0.44356685 | FALSE |
| 3 | 3 | 3 | 2 | 3 | 58558 | 0.07495133 | -0.2010924 | 0.35099506 | FALSE |
| 2 | 0 | 18 | 0 | 6 | 58570 | -0.3255495 | -0.9857579 | 0.33465895 | FALSE |
| 3 | 0 | 35 | 0 | 8 | 58611 | -0.4622411 | -1.24364 | 0.31915778 | FALSE |
| 3 | 0 | 12 | 0 | 5 | 58805 | -0.1825297 | -0.5776695 | 0.21261022 | FALSE |
| 3 | 3 | 4 | 2 | 3 | 58994 | 0.0975677 | -0.2617984 | 0.4569338 | FALSE |
| 1 | 0 | 17 | 0 | 5 | 59080 | -0.1497856 | -0.757023 | 0.4574519 | FALSE |
| 1 | 0 | 41 | 0 | 6 | 59117 | -0.2574547 | -1.1918596 | 0.67695021 | FALSE |
| 1 | 0 | 73 | 0 | 5 | 59119 | -0.3541004 | -1.5208997 | 0.81269884 | FALSE |
| 2 | 0 | 10 | 0 | 5 | 59121 | -0.2875066 | -0.7244591 | 0.14944592 | FALSE |
| 2 | 24 | 3 | 3 | 2 | 59161 | 0.56359916 | -0.3313266 | 1.45852496 | FALSE |
| 4 | 3 | 9 | 3 | 4 | 59261 | -0.0114694 | -0.4963031 | 0.47336437 | FALSE |
| 3 | 23 | 32 | 6 | 5 | 59462 | 0.54706132 | -0.5026836 | 1.59680626 | FALSE |
| 5 | 3 | 4 | 3 | 3 | 59503 | 0.13142263 | -0.1586033 | 0.42144854 | FALSE |
| 3 | 0 | 18 | 0 | 9 | 59541 | -0.184737 | -0.7041585 | 0.33468441 | FALSE |
| 10 | 14 | 41 | 5 | 14 | 59551 | 0.1498503 | -0.5487243 | 0.84842492 | FALSE |
| 5 | 24 | 35 | 6 | 11 | 59554 | 0.23163345 | -0.904228 | 1.36749485 | FALSE |
| 3 | 20 | 19 | 4 | 4 | 59559 | 0.36179885 | -0.4312446 | 1.1548423 | FALSE |
| 6 | 1 | 17 | 1 | 10 | 59592 | -0.3187482 | -0.768026 | 0.13052959 | FALSE |
| 3 | 1 | 11 | 1 | 6 | 59594 | -0.0121956 | -0.4408371 | 0.41644579 | FALSE |
| 7 | 8 | 24 | 5 | 10 | 59613 | 0.17786202 | -0.5275937 | 0.88331775 | FALSE |
| 2 | 6 | 11 | 2 | 4 | 59635 | 0.10631431 | -0.443792 | 0.6564206 | FALSE |
| 10 | 9 | 72 | 6 | 15 | 59657 | -0.417134 | -1.3864722 | 0.55220414 | FALSE |
| 9 | 25 | 43 | 9 | 13 | 59668 | 0.50241761 | -0.3785187 | 1.38335388 | FALSE |
| 4 | 1 | 16 | 1 | 6 | 59752 | -0.3208252 | -0.7581619 | 0.11651158 | FALSE |
| 5 | 1 | 22 | 1 | 5 | 59892 | -0.0830081 | -0.6130013 | 0.44698504 | FALSE |
| 4 | 0 | 11 | 0 | 8 | 59908 | -0.1955607 | -0.5266232 | 0.13550182 | FALSE |
| 7 | 3 | 25 | 3 | 11 | 59935 | -0.1969176 | -0.8355737 | 0.44173859 | FALSE |
| 11 | 108 | 515 | 12 | 26 | 59937 | 0.61141885 | -1.2786317 | 2.50146938 | FALSE |
| 3 | 1 | 12 | 1 | 4 | 60003 | -0.0201623 | -0.4204508 | 0.38012615 | FALSE |
| 6 | 27 | 33 | 8 | 8 | 60007 | 0.77108574 | -0.1821483 | 1.72431974 | FALSE |
| 3 | 7 | 6 | 3 | 5 | 60008 | 0.09842749 | -0.412077 | 0.60893201 | FALSE |
| 5 | 10 | 15 | 3 | 6 | 60011 | 0.37560394 | -0.3970908 | 1.14829866 | FALSE |
| 6 | 12 | 42 | 4 | 12 | 60012 | -0.0163717 | -0.9278044 | 0.89506108 | FALSE |
| 8 | 21 | 16 | 7 | 5 | 60014 | 0.55670968 | -0.1751586 | 1.28857798 | FALSE |
| 5 | 99 | 56 | 5 | 9 | 60065 | 0.98548215 | -1.1014955 | 3.0724598 | FALSE |
| 5 | 3 | 9 | 2 | 6 | 60091 | 0.14847471 | -0.3346736 | 0.63162304 | FALSE |
| 2 | 3 | 6 | 2 | 4 | 60112 | 0.14913345 | -0.3467449 | 0.64501183 | FALSE |
| 3 | 0 | 10 | 0 | 6 | 60194 | -0.0368139 | -0.3724318 | 0.29880402 | FALSE |
| 2 | 0 | 9 | 0 | 6 | 60307 | -0.0861586 | -0.4644544 | 0.2921372 | FALSE |
| 3 | 2 | 5 | 1 | 5 | 60546 | 0.01234406 | -0.2972347 | 0.32192286 | FALSE |
| 3 | 17 | 25 | 2 | 5 | 60635 | 0.67243403 | -0.7000372 | 2.04490524 | FALSE |
| 2 | 0 | 6 | 0 | 5 | 60648 | -0.0985852 | -0.4053549 | 0.20818462 | FALSE |
| 7 | 20 | 190 | 6 | 20 | 60864 | -0.4415565 | -1.811322 | 0.92820903 | FALSE |
| 3 | 2 | 4 | 2 | 3 | 60920 | 0.03792436 | -0.2539402 | 0.32978897 | FALSE |
| 5 | 4 | 3 | 3 | 3 | 60941 | 0.22097345 | -0.0224587 | 0.46440557 | FALSE |
| 5 | 1 | 13 | 1 | 10 | 60991 | -0.2517235 | -0.6449895 | 0.14154248 | FALSE |
| 8 | 26 | 53 | 8 | 11 | 61045 | 0.22830678 | -0.8476534 | 1.30426695 | FALSE |
| 3 | 1 | 40 | 1 | 8 | 61076 | -0.2896112 | -1.0822247 | 0.50300231 | FALSE |
| 4 | 3 | 4 | 2 | 3 | 61125 | 0.0451854 | -0.2147942 | 0.30516502 | FALSE |
| 10 | 6 | 21 | 4 | 10 | 61152 | 0.07328519 | -0.4309669 | 0.57753729 | FALSE |
| 4 | 3 | 6 | 3 | 5 | 61273 | 0.08877701 | -0.2461688 | 0.42372281 | FALSE |
| 2 | 0 | 8 | 0 | 6 | 61414 | -0.0761918 | -0.4254225 | 0.27303898 | FALSE |
| 8 | 73 | 73 | 9 | 15 | 61447 | 1.07683931 | -0.0879674 | 2.24164603 | FALSE |
| 6 | 8 | 8 | 6 | 7 | 61448 | 0.30874786 | -0.1580431 | 0.7755388 | FALSE |
| 4 | 3 | 6 | 3 | 5 | 61521 | -0.0531757 | -0.4271504 | 0.32079891 | FALSE |
| 3 | 11 | 38 | 4 | 6 | 61526 | 0.37287034 | -0.8360472 | 1.5817879 | FALSE |
| 11 | 58 | 256 | 11 | 23 | 61548 | 0.30698123 | -1.3235522 | 1.93751469 | FALSE |
| 6 | 10 | 16 | 5 | 6 | 61555 | 0.39346416 | -0.1796229 | 0.96655119 | FALSE |
| 5 | 6 | 2 | 3 | 2 | 61569 | 0.26756684 | -0.0105135 | 0.54564713 | FALSE |
| 3 | 0 | 42 | 0 | 6 | 61611 | -0.2680189 | -1.090774 | 0.55473631 | FALSE |
| 4 | 1 | 11 | 1 | 4 | 61619 | -0.0128412 | -0.3972831 | 0.37160075 | FALSE |
| 6 | 2 | 7 | 2 | 5 | 61621 | 0.00915186 | -0.2780202 | 0.29632394 | FALSE |
| 4 | 6 | 6 | 2 | 3 | 61650 | 0.2634629 | -0.3545735 | 0.88149932 | FALSE |
| 5 | 4 | 2 | 3 | 2 | 61677 | 0.13016631 | -0.0941378 | 0.35447046 | FALSE |
| 11 | 13 | 30 | 7 | 13 | 61680 | 0.17793858 | -0.6248564 | 0.98073352 | FALSE |
| 8 | 5 | 8 | 4 | 5 | 61727 | 0.15604108 | -0.1858689 | 0.49795107 | FALSE |
| 6 | 1 | 13 | 1 | 8 | 61755 | -0.1133438 | -0.5011136 | 0.27442589 | FALSE |
| 9 | 5 | 52 | 3 | 16 | 61760 | -0.3103377 | -1.0092193 | 0.3885438 | FALSE |
| 4 | 4 | 4 | 2 | 3 | 61825 | 0.16844121 | -0.1142897 | 0.45117218 | FALSE |
| 7 | 8 | 6 | 5 | 6 | 61861 | 0.24062054 | -0.1097253 | 0.59096638 | FALSE |
| 10 | 12 | 61 | 6 | 16 | 61892 | -0.1771692 | -1.0746487 | 0.72031033 | FALSE |
| 4 | 4 | 3 | 3 | 3 | 61945 | 0.15846673 | -0.0928851 | 0.40981859 | FALSE |
| 4 | 1 | 6 | 1 | 5 | 61960 | -0.0505852 | -0.3209668 | 0.21979646 | FALSE |
| 8 | 4 | 34 | 4 | 16 | 61982 | -0.3494266 | -1.0365414 | 0.33768809 | FALSE |
| 12 | 62 | 161 | 11 | 25 | 61989 | 0.38719863 | -0.7645171 | 1.53891442 | FALSE |
| 4 | 3 | 8 | 3 | 7 | 61999 | -0.0075335 | -0.3881234 | 0.37305637 | FALSE |
| 5 | 0 | 8 | 0 | 7 | 62009 | -0.2527932 | -0.5068274 | 0.00124096 | FALSE |
| 10 | 6 | 25 | 4 | 14 | 62018 | -0.1345633 | -0.7864635 | 0.51733701 | FALSE |
| 8 | 29 | 11 | 4 | 9 | 62028 | 0.81502268 | -0.4572737 | 2.0873191 | FALSE |
| 9 | 68 | 55 | 7 | 11 | 62128 | 0.68460548 | -0.5921804 | 1.96139138 | FALSE |
| 3 | 0 | 8 | 0 | 7 | 62282 | -0.1021271 | -0.443796 | 0.23954175 | FALSE |
| 2 | 1 | 5 | 1 | 4 | 62417 | 0.03864192 | -0.3463297 | 0.42361359 | FALSE |
| 6 | 7 | 19 | 5 | 8 | 62479 | 0.066378 | -0.5782803 | 0.71103627 | FALSE |
| 7 | 5 | 12 | 4 | 7 | 62486 | 0.07430971 | -0.3333214 | 0.48194086 | FALSE |
| 4 | 4 | 3 | 2 | 3 | 62499 | 0.13528435 | -0.1203537 | 0.39092237 | FALSE |
| 3 | 0 | 6 | 0 | 5 | 62869 | -0.1659565 | -0.5584688 | 0.2265558 | FALSE |
| 4 | 1 | 6 | 1 | 5 | 62901 | -0.0497713 | -0.3377401 | 0.23819752 | FALSE |
| 4 | 2 | 6 | 2 | 5 | 62906 | -0.1060315 | -0.482965 | 0.27090209 | FALSE |
| 2 | 0 | 37 | 0 | 7 | 62933 | -0.2907656 | -1.2282599 | 0.64672877 | FALSE |
| 3 | 32 | 16 | 3 | 5 | 62996 | 0.46496526 | -0.6622039 | 1.59213442 | FALSE |
| 3 | 6 | 2 | 3 | 2 | 63127 | 0.31626802 | -0.0525364 | 0.68507244 | FALSE |
| 5 | 3 | 32 | 1 | 10 | 63138 | -0.3254757 | -1.0764696 | 0.42551821 | FALSE |
| 6 | 2 | 84 | 2 | 16 | 63166 | -0.6256449 | -1.8921944 | 0.64090463 | FALSE |
| 3 | 2 | 117 | 1 | 8 | 63274 | -0.7012234 | -2.0896017 | 0.68715484 | FALSE |
| 3 | 0 | 11 | 0 | 6 | 63312 | -0.1704276 | -0.6002453 | 0.25939015 | FALSE |
| 5 | 1 | 69 | 1 | 11 | 63320 | -0.7382877 | -1.9974683 | 0.5208929 | FALSE |
| 2 | 0 | 21 | 0 | 5 | 63563 | -0.3092851 | -0.873338 | 0.25476779 | FALSE |
| 4 | 0 | 7 | 0 | 6 | 63579 | -0.1724777 | -0.4635891 | 0.11863368 | FALSE |
| 2 | 2 | 103 | 1 | 6 | 63752 | -0.2440652 | -1.5944728 | 1.10634242 | FALSE |
| 7 | 85 | 53 | 6 | 8 | 64060 | 0.53792299 | -1.0062296 | 2.08207557 | FALSE |
| 2 | 1 | 5 | 1 | 4 | 64142 | -0.09264 | -0.4139677 | 0.22868763 | FALSE |
| 3 | 3 | 23 | 3 | 2 | 64157 | -0.139084 | -0.7746507 | 0.49648277 | FALSE |
| 4 | 1 | 28 | 1 | 10 | 64291 | -0.355217 | -1.0498489 | 0.33941484 | FALSE |
| 4 | 2 | 6 | 1 | 4 | 64411 | -0.0978242 | -0.4333191 | 0.23767067 | FALSE |
| 3 | 1 | 14 | 1 | 7 | 64421 | -0.242487 | -0.8400819 | 0.35510782 | FALSE |
| 8 | 32 | 55 | 8 | 14 | 64500 | 0.42764232 | -0.5410881 | 1.3963727 | FALSE |
| 9 | 49 | 49 | 7 | 17 | 64517 | 0.71734499 | -0.4901074 | 1.92479742 | FALSE |
| 8 | 14 | 12 | 6 | 9 | 64563 | 0.3563268 | -0.1406676 | 0.85332125 | FALSE |
| 7 | 13 | 29 | 3 | 10 | 64609 | -0.0748204 | -0.8419695 | 0.69232879 | FALSE |
| 9 | 8 | 92 | 4 | 13 | 64628 | -0.2260275 | -1.2305242 | 0.77846918 | FALSE |
| 7 | 7 | 7 | 5 | 7 | 64635 | 0.2357877 | -0.0861991 | 0.55777446 | FALSE |
| 5 | 5 | 3 | 4 | 3 | 64674 | 0.25415849 | -0.0282134 | 0.53653042 | FALSE |
| 7 | 6 | 5 | 4 | 4 | 64677 | 0.20554773 | -0.1086976 | 0.51979307 | FALSE |
| 6 | 3 | 6 | 3 | 3 | 64681 | -0.0167958 | -0.3043094 | 0.27071775 | FALSE |
| 3 | 7 | 8 | 4 | 3 | 64710 | 0.26004239 | -0.2309271 | 0.75101191 | FALSE |
| 5 | 2 | 14 | 2 | 5 | 64794 | -0.1877813 | -0.5872164 | 0.21165386 | FALSE |
| 4 | 0 | 20 | 0 | 8 | 64991 | -0.3129532 | -0.8684847 | 0.24257836 | FALSE |
| 4 | 2 | 4 | 1 | 4 | 64996 | 0.09633934 | -0.1371285 | 0.32980723 | FALSE |
| 11 | 101 | 495 | 12 | 25 | 65042 | 0.72595565 | -1.11391 | 2.56582132 | FALSE |
| 2 | 1 | 9 | 1 | 4 | 65050 | -0.0487148 | -0.48872 | 0.39129047 | FALSE |
| 6 | 6 | 16 | 3 | 9 | 65067 | -0.0240642 | -0.5813354 | 0.53320702 | FALSE |
| 3 | 0 | 7 | 0 | 5 | 65089 | -0.0507841 | -0.3277719 | 0.22620367 | FALSE |
| 6 | 3 | 47 | 3 | 14 | 65094 | -0.4572861 | -1.3765795 | 0.46200731 | FALSE |
| 4 | 0 | 11 | 0 | 7 | 65134 | -0.2680058 | -0.6343417 | 0.0983301 | FALSE |
| 6 | 6 | 46 | 3 | 11 | 65175 | -0.139222 | -1.0076285 | 0.72918459 | FALSE |
| 9 | 24 | 78 | 9 | 18 | 65177 | 0.3534243 | -0.6096537 | 1.31650229 | FALSE |
| 8 | 14 | 31 | 4 | 11 | 65184 | -0.037648 | -0.7675145 | 0.69221857 | FALSE |
| 2 | 15 | 95 | 4 | 5 | 65269 | -0.1837036 | -1.767071 | 1.39966369 | FALSE |
| 2 | 3 | 12 | 1 | 5 | 65270 | -0.035126 | -0.6314903 | 0.56123836 | FALSE |
| 5 | 2 | 7 | 2 | 5 | 65283 | -0.0358677 | -0.3541834 | 0.282448 | FALSE |
| 3 | 3 | 90 | 2 | 9 | 65457 | -0.0815498 | -1.3623401 | 1.19924047 | FALSE |
| 3 | 1 | 27 | 1 | 7 | 65460 | -0.0150312 | -0.6974035 | 0.66734107 | FALSE |
| 8 | 25 | 33 | 11 | 11 | 65576 | 0.65234279 | -0.2669871 | 1.57167273 | FALSE |
| 4 | 0 | 17 | 0 | 9 | 65835 | -0.3483517 | -0.869255 | 0.17255154 | FALSE |
| 1 | 0 | 26 | 0 | 5 | 65986 | -0.1088701 | -0.8079476 | 0.59020732 | FALSE |
| 3 | 0 | 17 | 0 | 10 | 66070 | -0.2277222 | -0.734942 | 0.27949771 | FALSE |
| 5 | 2 | 6 | 2 | 3 | 66073 | 0.15837158 | -0.1093986 | 0.42614173 | FALSE |
| 5 | 2 | 6 | 2 | 4 | 66222 | 0.05106165 | -0.2247212 | 0.32684454 | FALSE |
| 11 | 231 | 176 | 13 | 20 | 66330 | 1.26752507 | -0.4102892 | 2.94533931 | FALSE |
| 4 | 0 | 9 | 0 | 6 | 66656 | -0.2160631 | -0.5062754 | 0.07414912 | FALSE |
| 5 | 2 | 4 | 2 | 4 | 66683 | -0.0044207 | -0.2298494 | 0.22100808 | FALSE |
| 6 | 7 | 7 | 3 | 5 | 66818 | 0.18107287 | -0.1890416 | 0.5511873 | FALSE |
| 4 | 5 | 8 | 4 | 4 | 66943 | 0.17159388 | -0.2944258 | 0.63761359 | FALSE |
| 3 | 0 | 7 | 0 | 5 | 67019 | -0.081301 | -0.396944 | 0.23434189 | FALSE |
| 3 | 1 | 5 | 1 | 4 | 67022 | -0.0051987 | -0.2677963 | 0.25739887 | FALSE |
| 4 | 2 | 9 | 2 | 4 | 67100 | -0.1124894 | -0.5881839 | 0.36320502 | FALSE |
| 4 | 5 | 3 | 4 | 3 | 67145 | 0.14104182 | -0.2089489 | 0.49103257 | FALSE |
| 7 | 12 | 13 | 4 | 7 | 67160 | 0.28836833 | -0.1778279 | 0.7545646 | FALSE |
| 5 | 18 | 16 | 7 | 8 | 67203 | 0.3704868 | -0.4609277 | 1.20190124 | FALSE |
| 6 | 3 | 13 | 1 | 8 | 67259 | -0.1112904 | -0.5377455 | 0.31516461 | FALSE |
| 4 | 9 | 2 | 4 | 2 | 67395 | 0.27553995 | -0.1632035 | 0.71428338 | FALSE |
| 4 | 6 | 9 | 2 | 4 | 67415 | 0.0827511 | -0.3628335 | 0.52833574 | FALSE |
| 9 | 14 | 12 | 5 | 5 | 67451 | 0.41565491 | -0.0843385 | 0.91564838 | FALSE |
| 5 | 0 | 16 | 0 | 9 | 67502 | -0.228862 | -0.6349711 | 0.17724714 | FALSE |
| 5 | 4 | 2 | 3 | 2 | 67585 | 0.21912419 | -0.0109043 | 0.44915265 | FALSE |
| 3 | 2 | 4 | 2 | 3 | 67641 | 0.11714147 | -0.2118566 | 0.44613952 | FALSE |
| 10 | 42 | 38 | 11 | 19 | 67703 | 0.6354269 | -0.2164015 | 1.48725531 | FALSE |
| 7 | 6 | 13 | 4 | 6 | 67759 | 0.12386274 | -0.309071 | 0.55679649 | FALSE |
| 11 | 17 | 45 | 6 | 18 | 67760 | 0.0249074 | -0.8216089 | 0.87142367 | FALSE |
| 5 | 7 | 10 | 3 | 5 | 67765 | 0.10371596 | -0.3296476 | 0.53707953 | FALSE |
| 3 | 0 | 9 | 0 | 5 | 67772 | -0.207088 | -0.605131 | 0.19095488 | FALSE |
| 3 | 1 | 6 | 1 | 4 | 67779 | -0.2172772 | -0.6395306 | 0.20497608 | FALSE |
| 7 | 6 | 14 | 5 | 5 | 67809 | 0.34920376 | -0.1593811 | 0.85778861 | FALSE |
| 8 | 3 | 18 | 2 | 8 | 67877 | -0.2669283 | -0.7479763 | 0.2141198 | FALSE |
| 5 | 10 | 10 | 3 | 5 | 67911 | 0.32718009 | -0.2007061 | 0.85506626 | FALSE |
| 3 | 1 | 7 | 1 | 4 | 67923 | -0.0405972 | -0.3999462 | 0.31875189 | FALSE |
| 3 | 1 | 5 | 1 | 4 | 67991 | -0.0758072 | -0.3445482 | 0.19293382 | FALSE |
| 3 | 0 | 38 | 0 | 10 | 68029 | -0.3910658 | -1.1929988 | 0.41086719 | FALSE |
| 3 | 0 | 14 | 0 | 5 | 68137 | -0.126503 | -0.6285782 | 0.3755723 | FALSE |
| 6 | 8 | 14 | 3 | 9 | 68830 | 0.11991845 | -0.4683243 | 0.70816119 | FALSE |
| 5 | 4 | 9 | 3 | 6 | 68963 | 0.06640171 | -0.3454151 | 0.47821856 | FALSE |
| 6 | 13 | 9 | 6 | 8 | 68978 | 0.3565415 | -0.1815222 | 0.89460523 | FALSE |
| 6 | 10 | 8 | 3 | 8 | 68993 | 0.12291232 | -0.466655 | 0.71247963 | FALSE |
| 5 | 3 | 18 | 1 | 7 | 69027 | -0.3245681 | -0.8710025 | 0.22186629 | FALSE |
| 7 | 16 | 49 | 5 | 14 | 69066 | -0.1504549 | -1.1695585 | 0.8686487 | FALSE |
| 6 | 4 | 9 | 3 | 5 | 69133 | 0.12010004 | -0.250801 | 0.49100104 | FALSE |
| 4 | 1 | 6 | 1 | 5 | 69190 | -0.0227086 | -0.2859244 | 0.24050727 | FALSE |
| 7 | 5 | 51 | 3 | 14 | 69216 | -0.3524773 | -1.3258716 | 0.62091697 | FALSE |
| 7 | 7 | 38 | 5 | 15 | 69218 | -0.0618846 | -0.9301126 | 0.80634336 | FALSE |
| 6 | 0 | 73 | 0 | 12 | 69297 | -0.8696229 | -1.9414806 | 0.20223486 | FALSE |
| 5 | 0 | 20 | 0 | 9 | 69313 | -0.3711922 | -0.9587049 | 0.21632048 | FALSE |
| 2 | 0 | 8 | 0 | 6 | 69315 | -0.3290343 | -0.7825832 | 0.12451457 | FALSE |
| 3 | 1 | 5 | 1 | 4 | 69327 | -0.1751212 | -0.4735139 | 0.12327147 | FALSE |
| 3 | 0 | 15 | 0 | 7 | 69353 | -0.3262086 | -0.8988466 | 0.24642939 | FALSE |
| 2 | 0 | 14 | 0 | 5 | 69583 | -0.1882438 | -0.7391853 | 0.36269765 | FALSE |
| 1 | 0 | 9 | 0 | 5 | 69755 | -0.1925992 | -0.6283635 | 0.24316511 | FALSE |
| 2 | 2 | 31 | 1 | 5 | 69969 | -0.1414907 | -1.0187568 | 0.73577537 | FALSE |
| 5 | 44 | 53 | 4 | 9 | 69976 | 0.27623165 | -1.1755064 | 1.72796965 | FALSE |
| 4 | 20 | 2 | 3 | 2 | 70080 | 0.59715867 | -0.0266017 | 1.220919 | FALSE |
| 1 | 0 | 28 | 0 | 6 | 70108 | -0.2522654 | -1.0662273 | 0.5616965 | FALSE |
| 4 | 3 | 22 | 2 | 5 | 70144 | -0.2203712 | -0.901239 | 0.46049668 | FALSE |
| 1 | 0 | 12 | 0 | 5 | 70145 | -0.1253003 | -0.62733 | 0.37672943 | FALSE |
| 1 | 0 | 10 | 0 | 5 | 70147 | -0.1161541 | -0.5745483 | 0.34224019 | FALSE |
| 6 | 3 | 3 | 3 | 3 | 70582 | 0.14473933 | -0.0752722 | 0.3647509 | FALSE |
| 4 | 14 | 5 | 3 | 5 | 70624 | 0.23899785 | -0.3739485 | 0.85194422 | FALSE |
| 5 | 0 | 23 | 0 | 8 | 70665 | -0.31068 | -0.7706066 | 0.14924658 | FALSE |
| 5 | 4 | 8 | 3 | 5 | 70696 | 0.04218751 | -0.3780421 | 0.46241708 | FALSE |
| 4 | 1 | 5 | 1 | 4 | 70744 | -0.0835262 | -0.3231625 | 0.1561102 | FALSE |
| 3 | 0 | 6 | 0 | 5 | 71143 | -0.1638301 | -0.4338144 | 0.10615429 | FALSE |
| 3 | 8 | 47 | 4 | 5 | 71179 | 0.18352641 | -0.7712234 | 1.13827621 | FALSE |
| 5 | 5 | 18 | 2 | 9 | 71199 | -0.08392 | -0.6840447 | 0.51620463 | FALSE |
| 5 | 48 | 91 | 7 | 7 | 71205 | 0.59972472 | -0.9981641 | 2.19761355 | FALSE |
| 4 | 0 | 6 | 0 | 5 | 71207 | -0.0816549 | -0.3091762 | 0.14586652 | FALSE |
| 4 | 0 | 31 | 0 | 7 | 71281 | -0.2405643 | -0.9225073 | 0.44137876 | FALSE |
| 8 | 22 | 73 | 8 | 12 | 71364 | 0.51348522 | -0.5736218 | 1.60059221 | FALSE |
| 5 | 4 | 11 | 3 | 4 | 71368 | 0.07747267 | -0.3408427 | 0.49578801 | FALSE |
| 5 | 3 | 3 | 3 | 3 | 71389 | 0.11424891 | -0.1080273 | 0.33652514 | FALSE |
| 7 | 40 | 58 | 8 | 11 | 72203 | 0.93698566 | -0.1924646 | 2.06643588 | FALSE |
| 3 | 0 | 12 | 0 | 6 | 72207 | -0.1322975 | -0.5246803 | 0.26008523 | FALSE |
| 3 | 2 | 6 | 2 | 4 | 72238 | 0.20960104 | -0.2353345 | 0.65453655 | FALSE |
| 10 | 128 | 100 | 13 | 21 | 72282 | 0.71653303 | -0.5964654 | 2.02953145 | FALSE |
| 4 | 4 | 4 | 2 | 4 | 72344 | 0.08533911 | -0.2761091 | 0.44678737 | FALSE |
| 2 | 1 | 13 | 1 | 4 | 72367 | -0.0292954 | -0.5531815 | 0.49459059 | FALSE |
| 10 | 9 | 28 | 7 | 12 | 72373 | 0.08864399 | -0.5413602 | 0.7186482 | FALSE |
| 4 | 1 | 5 | 1 | 4 | 72374 | -0.0239939 | -0.2697755 | 0.22178771 | FALSE |
| 4 | 5 | 4 | 3 | 3 | 72379 | 0.24191125 | -0.0493961 | 0.53321855 | FALSE |
| 5 | 26 | 21 | 4 | 8 | 72397 | 0.50100007 | -0.4802259 | 1.48222603 | FALSE |
| 10 | 75 | 72 | 13 | 17 | 72416 | 0.99028312 | -0.1757839 | 2.15635013 | FALSE |
| 3 | 32 | 2 | 3 | 2 | 72446 | 0.58688199 | -0.1566626 | 1.33042661 | FALSE |
| 6 | 8 | 26 | 5 | 8 | 72469 | 0.01514442 | -0.759034 | 0.7893228 | FALSE |
| 3 | 3 | 17 | 2 | 4 | 72474 | -0.1755797 | -0.9631879 | 0.61202844 | FALSE |
| 2 | 164 | 0 | 6 | 0 | 72475 | 1.13926277 | -0.2140558 | 2.49258128 | FALSE |
| 3 | 9 | 3 | 3 | 3 | 72496 | 0.22314411 | -0.2127439 | 0.65903212 | FALSE |
| 5 | 4 | 5 | 2 | 4 | 72542 | 0.16457083 | -0.1306916 | 0.45983325 | FALSE |
| 3 | 0 | 12 | 0 | 7 | 72610 | -0.2198934 | -0.6594589 | 0.21967216 | FALSE |
| 5 | 6 | 9 | 4 | 8 | 72754 | 0.26705501 | -0.1896509 | 0.7237609 | FALSE |
| 3 | 3 | 6 | 2 | 3 | 72758 | 0.11185662 | -0.2234636 | 0.44717683 | FALSE |
| 3 | 3 | 17 | 2 | 3 | 72964 | -0.0046057 | -0.7583073 | 0.74909578 | FALSE |
| 5 | 3 | 6 | 3 | 4 | 73089 | 0.0987049 | -0.2037339 | 0.40114365 | FALSE |
| 5 | 4 | 9 | 2 | 4 | 73114 | 0.06333328 | -0.3342881 | 0.46095472 | FALSE |
| 10 | 16 | 36 | 7 | 13 | 73115 | 0.26231183 | -0.4732233 | 0.99784697 | FALSE |
| 5 | 0 | 10 | 0 | 7 | 73121 | -0.1607942 | -0.5063422 | 0.18475391 | FALSE |
| 5 | 0 | 14 | 0 | 7 | 73157 | -0.1990686 | -0.5801248 | 0.1819876 | FALSE |
| 3 | 4 | 8 | 2 | 4 | 73161 | 0.00755768 | -0.4322769 | 0.44739221 | FALSE |
| 2 | 4 | 7 | 2 | 4 | 73258 | 0.04518791 | -0.4156478 | 0.50602359 | FALSE |
| 8 | 5 | 7 | 4 | 5 | 73389 | 0.17330223 | -0.1530354 | 0.49963985 | FALSE |
| 7 | 4 | 17 | 1 | 9 | 73516 | -0.1134144 | -0.5769978 | 0.35016904 | FALSE |
| 3 | 0 | 6 | 0 | 5 | 73734 | -0.0610239 | -0.3142737 | 0.19222589 | FALSE |
| 3 | 3 | 4 | 3 | 3 | 73785 | 0.13916605 | -0.1755532 | 0.45388527 | FALSE |
| 4 | 2 | 6 | 2 | 3 | 73787 | 0.10184077 | -0.1976594 | 0.4013409 | FALSE |
| 4 | 10 | 8 | 3 | 5 | 73892 | 0.13625329 | -0.4008418 | 0.67334834 | FALSE |
| 7 | 20 | 11 | 6 | 7 | 73915 | 0.44136583 | -0.3005123 | 1.18324393 | FALSE |
| 4 | 1 | 6 | 1 | 4 | 73954 | -0.1072289 | -0.4053858 | 0.19092796 | FALSE |
| 4 | 1 | 11 | 1 | 8 | 73963 | -0.0699085 | -0.5018929 | 0.36207589 | FALSE |
| 8 | 6 | 9 | 5 | 6 | 73973 | 0.09449055 | -0.2461544 | 0.43513551 | FALSE |
| 7 | 5 | 7 | 5 | 4 | 73975 | 0.11231764 | -0.1874077 | 0.41204302 | FALSE |
| 6 | 2 | 8 | 2 | 6 | 73976 | 0.00812064 | -0.2942744 | 0.31051564 | FALSE |
| 5 | 7 | 8 | 5 | 3 | 73980 | 0.18625465 | -0.3675954 | 0.74010464 | FALSE |
| 5 | 1 | 8 | 1 | 6 | 73983 | -0.1667082 | -0.4866698 | 0.15325335 | FALSE |
| 4 | 1 | 8 | 1 | 7 | 73989 | -0.1263863 | -0.5356092 | 0.2828366 | FALSE |
| 5 | 5 | 2 | 3 | 2 | 74050 | 0.42859367 | -0.1111323 | 0.96831966 | FALSE |
| 4 | 2 | 4 | 2 | 4 | 74147 | 0.07064896 | -0.1775287 | 0.31882661 | FALSE |
| 5 | 5 | 2 | 4 | 2 | 74158 | 0.2656931 | -0.0947044 | 0.62609064 | FALSE |
| 4 | 4 | 4 | 2 | 4 | 74198 | 0.07368324 | -0.2346849 | 0.3820514 | FALSE |
| 5 | 5 | 4 | 3 | 3 | 74227 | 0.14499042 | -0.1818526 | 0.4718334 | FALSE |
| 7 | 4 | 11 | 3 | 4 | 74288 | 0.15538285 | -0.2334871 | 0.54425284 | FALSE |
| 2 | 15 | 2 | 4 | 1 | 74400 | 0.36371883 | -0.3172839 | 1.04472154 | FALSE |
| 4 | 9 | 9 | 1 | 5 | 74406 | 0.328675 | -0.6470833 | 1.30443334 | FALSE |
| 2 | 16 | 3 | 4 | 3 | 74408 | 0.43947847 | -0.2952463 | 1.17420328 | FALSE |
| 5 | 12 | 7 | 2 | 4 | 74574 | 0.6008922 | -0.4460165 | 1.64780091 | FALSE |
| 3 | 0 | 21 | 0 | 10 | 74669 | -0.3796647 | -0.9710539 | 0.21172451 | FALSE |
| 5 | 8 | 6 | 2 | 3 | 75148 | 0.12747747 | -0.2577714 | 0.51272631 | FALSE |
| 3 | 17 | 18 | 3 | 5 | 75149 | 0.02811941 | -0.8149605 | 0.87119935 | FALSE |
| 5 | 6 | 5 | 3 | 3 | 75172 | 0.19365648 | -0.1381437 | 0.52545669 | FALSE |
| 3 | 13 | 12 | 2 | 6 | 75240 | 0.03718032 | -0.6347235 | 0.70908417 | FALSE |
| 6 | 2 | 12 | 2 | 5 | 75302 | -0.1113889 | -0.579831 | 0.35705323 | FALSE |
| 4 | 3 | 6 | 2 | 3 | 75360 | 0.10587979 | -0.2036751 | 0.41543466 | FALSE |
| 3 | 1 | 11 | 1 | 4 | 75403 | -0.0334784 | -0.4541562 | 0.38719936 | FALSE |
| 3 | 1 | 37 | 1 | 6 | 75436 | -0.3830572 | -1.3387757 | 0.57266127 | FALSE |
| 5 | 1 | 17 | 1 | 9 | 75438 | -0.4420247 | -1.0504565 | 0.16640712 | FALSE |
| 3 | 0 | 29 | 0 | 9 | 75490 | -0.5029527 | -1.2528457 | 0.2469404 | FALSE |
| 4 | 0 | 12 | 0 | 10 | 75510 | -0.3457423 | -0.7513551 | 0.05987046 | FALSE |
| 3 | 0 | 28 | 0 | 12 | 75550 | -0.6932967 | -1.5253328 | 0.13873944 | FALSE |
| 4 | 1 | 6 | 1 | 5 | 75615 | -0.0448195 | -0.3439754 | 0.2543364 | FALSE |
| 2 | 21 | 0 | 5 | 0 | 75629 | 0.61962303 | -0.1568256 | 1.39607164 | FALSE |
| 3 | 8 | 5 | 3 | 2 | 76072 | 0.37184757 | -0.1603136 | 0.90400877 | FALSE |
| 4 | 6 | 5 | 4 | 3 | 76133 | 0.23667685 | -0.1653069 | 0.63866062 | FALSE |
| 3 | 0 | 11 | 0 | 6 | 76359 | -0.1509262 | -0.5775863 | 0.27573388 | FALSE |
| 4 | 1 | 6 | 1 | 6 | 76385 | -0.0770209 | -0.3897715 | 0.23572973 | FALSE |
| 3 | 0 | 21 | 0 | 7 | 76536 | -0.6086532 | -1.3582624 | 0.14095592 | FALSE |
| 3 | 3 | 3 | 2 | 3 | 76652 | 0.10999528 | -0.1709206 | 0.39091112 | FALSE |
| 3 | 1 | 12 | 1 | 6 | 76962 | -0.0032854 | -0.4643355 | 0.45776467 | FALSE |
| 4 | 4 | 12 | 3 | 5 | 77580 | 0.04481799 | -0.4203468 | 0.50998281 | FALSE |
| 2 | 0 | 32 | 0 | 6 | 78221 | -0.1024233 | -0.8090486 | 0.60420199 | FALSE |
| 6 | 2 | 8 | 1 | 6 | 78401 | -0.1013718 | -0.4113558 | 0.20861211 | FALSE |
| 9 | 72 | 181 | 10 | 20 | 78514 | 0.68417643 | -0.8794991 | 2.24785195 | FALSE |
| 4 | 0 | 60 | 0 | 11 | 78886 | -0.7383051 | -1.7389685 | 0.26235833 | FALSE |
| 2 | 0 | 6 | 0 | 5 | 79241 | -0.0582331 | -0.366998 | 0.25053168 | FALSE |
| 2 | 0 | 8 | 0 | 5 | 80435 | -0.1148393 | -0.4507305 | 0.22105194 | FALSE |
| 3 | 0 | 6 | 0 | 5 | 80511 | -0.0961873 | -0.376794 | 0.18441948 | FALSE |
| 4 | 2 | 6 | 2 | 5 | 80568 | -0.0636307 | -0.3923465 | 0.26508503 | FALSE |
| 3 | 2 | 4 | 2 | 4 | 80639 | 0.0903014 | -0.1894583 | 0.37006106 | FALSE |
| 6 | 4 | 4 | 4 | 3 | 80903 | 0.14912239 | -0.1069829 | 0.40522774 | FALSE |
| 4 | 1 | 8 | 1 | 6 | 81234 | -0.0395191 | -0.4343543 | 0.35531614 | FALSE |
| 3 | 5 | 12 | 2 | 5 | 81322 | 0.09746385 | -0.5046606 | 0.69958831 | FALSE |
| 4 | 1 | 10 | 1 | 6 | 81634 | -0.1267771 | -0.5886464 | 0.33509229 | FALSE |
| 5 | 7 | 6 | 4 | 5 | 81636 | 0.37721223 | -0.339718 | 1.09414244 | FALSE |
| 4 | 1 | 13 | 1 | 5 | 81674 | -0.0574795 | -0.5191103 | 0.4041513 | FALSE |
| 1 | 0 | 19 | 0 | 5 | 81914 | -0.092873 | -0.7087318 | 0.52298579 | FALSE |
| 2 | 3 | 6 | 3 | 2 | 81965 | 0.07616318 | -0.3456563 | 0.49798262 | FALSE |
| 5 | 2 | 4 | 2 | 3 | 82252 | 0.03311965 | -0.2046359 | 0.27087516 | FALSE |
| 5 | 1 | 8 | 1 | 5 | 82480 | -0.0961757 | -0.4163227 | 0.2239713 | FALSE |
| 4 | 15 | 122 | 2 | 8 | 82525 | -0.6589946 | -2.2866888 | 0.96869949 | FALSE |
| 4 | 4 | 111 | 1 | 10 | 82538 | -0.6918935 | -2.1045866 | 0.72079968 | FALSE |
| 3 | 0 | 7 | 0 | 6 | 82596 | -0.1542683 | -0.4353524 | 0.12681578 | FALSE |
| 4 | 1 | 6 | 1 | 4 | 82597 | -0.0696519 | -0.3747858 | 0.23548189 | FALSE |
| 3 | 1 | 8 | 1 | 5 | 82613 | -0.0981411 | -0.5197506 | 0.32346849 | FALSE |
| 3 | 0 | 15 | 0 | 8 | 82646 | -0.1866005 | -0.6405639 | 0.26736293 | FALSE |
| 3 | 4 | 2 | 3 | 2 | 82943 | 0.29689311 | -0.093288 | 0.68707424 | FALSE |
| 2 | 5 | 214 | 1 | 6 | 83000 | -0.301003 | -2.0397206 | 1.43771455 | FALSE |
| 1 | 0 | 35 | 0 | 5 | 83141 | -0.1617152 | -1.0199906 | 0.6965602 | FALSE |
| 2 | 38 | 15 | 3 | 3 | 83183 | 0.51550068 | -0.7796016 | 1.81060292 | FALSE |
| 5 | 0 | 32 | 0 | 11 | 83367 | -0.6199268 | -1.3033015 | 0.06344791 | FALSE |
| 3 | 0 | 24 | 0 | 7 | 83720 | -0.3785556 | -1.1515741 | 0.39446287 | FALSE |
| 3 | 0 | 8 | 0 | 6 | 83822 | -0.1464248 | -0.4620257 | 0.16917611 | FALSE |
| 4 | 4 | 7 | 2 | 3 | 84295 | 0.06932857 | -0.2876991 | 0.42635623 | FALSE |
| 6 | 3 | 20 | 3 | 10 | 84348 | -0.119746 | -0.7695094 | 0.53001743 | FALSE |
| 12 | 48 | 181 | 12 | 17 | 84372 | 1.10760414 | -0.3043129 | 2.51952115 | FALSE |
| 5 | 3 | 6 | 2 | 3 | 84376 | 0.02161335 | -0.3097821 | 0.35300881 | FALSE |
| 8 | 25 | 51 | 10 | 12 | 84644 | 0.83735403 | -0.2516972 | 1.92640528 | FALSE |
| 5 | 15 | 16 | 7 | 8 | 85096 | 0.44883727 | -0.3091504 | 1.20682489 | FALSE |
| 1 | 0 | 26 | 0 | 5 | 85114 | -0.1346277 | -0.8459207 | 0.57666538 | FALSE |
| 1 | 0 | 88 | 0 | 5 | 85120 | -0.1944546 | -1.2461421 | 0.85723278 | FALSE |
| 1 | 0 | 8 | 0 | 5 | 85236 | -0.1353189 | -0.532126 | 0.26148819 | FALSE |
| 2 | 0 | 9 | 0 | 5 | 85275 | -0.1343768 | -0.5318118 | 0.26305827 | FALSE |
| 8 | 45 | 114 | 5 | 15 | 85491 | 0.17776631 | -1.1261446 | 1.48167722 | FALSE |
| 9 | 223 | 445 | 11 | 19 | 85535 | 1.1773717 | -1.1481602 | 3.50290361 | FALSE |
| 2 | 1 | 25 | 1 | 5 | 85618 | -0.1791657 | -0.8555733 | 0.49724189 | FALSE |
| 3 | 0 | 13 | 0 | 5 | 85786 | -0.0948121 | -0.4826444 | 0.29302027 | FALSE |
| 6 | 13 | 29 | 4 | 11 | 85802 | 0.261953 | -0.486396 | 1.01030205 | FALSE |
| 4 | 0 | 42 | 0 | 12 | 85804 | -0.7930447 | -1.5991247 | 0.01303533 | FALSE |
| 2 | 6 | 1 | 4 | 1 | 85830 | 0.2428069 | -0.1842479 | 0.66986166 | FALSE |
| 3 | 2 | 4 | 1 | 4 | 86204 | 0.01630887 | -0.2556435 | 0.28826122 | FALSE |
| 4 | 1 | 5 | 1 | 4 | 86396 | 0.01548655 | -0.2236142 | 0.25458731 | FALSE |
| 4 | 0 | 16 | 0 | 9 | 86473 | -0.3785209 | -0.8906641 | 0.13362223 | FALSE |
| 3 | 3 | 4 | 1 | 4 | 87003 | 0.07610894 | -0.2650133 | 0.41723116 | FALSE |
| 4 | 4 | 5 | 3 | 3 | 87994 | 0.19833216 | -0.1135626 | 0.51022687 | FALSE |
| 2 | 11 | 14 | 4 | 4 | 88011 | 0.2790224 | -0.4629733 | 1.02101813 | FALSE |
| 4 | 1 | 326 | 1 | 13 | 88275 | -1.0565938 | -3.0625885 | 0.94940091 | FALSE |
| 2 | 0 | 6 | 0 | 5 | 88289 | -0.0574109 | -0.3371254 | 0.22230353 | FALSE |
| 3 | 286 | 331 | 5 | 3 | 88325 | 0.67735762 | -2.1593292 | 3.51404442 | FALSE |
| 2 | 58 | 17 | 4 | 3 | 88327 | 0.45120057 | -0.9762437 | 1.8786448 | FALSE |
| 1 | 0 | 6 | 0 | 5 | 88484 | -0.1241745 | -0.4638038 | 0.21545478 | FALSE |
| 4 | 4 | 3 | 3 | 3 | 88555 | 0.19861248 | -0.2026314 | 0.59985632 | FALSE |
| 5 | 2 | 17 | 2 | 7 | 88717 | -0.1422637 | -0.8558425 | 0.57131509 | FALSE |
| 5 | 0 | 8 | 0 | 6 | 88821 | -0.2172011 | -0.4755397 | 0.04113743 | FALSE |
| 2 | 0 | 6 | 0 | 5 | 88953 | -0.2035345 | -0.5592756 | 0.15220655 | FALSE |
| 2 | 4 | 3 | 3 | 2 | 88958 | 0.17203735 | -0.3147517 | 0.65882637 | FALSE |
| 2 | 0 | 14 | 0 | 5 | 89010 | -0.3078697 | -0.8764768 | 0.26073736 | FALSE |
| 3 | 0 | 8 | 0 | 5 | 89069 | -0.1646122 | -0.6323718 | 0.30314741 | FALSE |
| 7 | 5 | 26 | 3 | 14 | 89279 | -0.1097409 | -0.7367046 | 0.51722284 | FALSE |
| 6 | 1 | 11 | 1 | 8 | 89287 | -0.2367706 | -0.5669775 | 0.09343623 | FALSE |
| 5 | 5 | 25 | 3 | 7 | 89434 | -0.0660792 | -0.7107594 | 0.57860102 | FALSE |
| 2 | 0 | 75 | 0 | 6 | 89586 | -0.5521579 | -1.3914426 | 0.28712675 | FALSE |
| 3 | 1 | 19 | 1 | 4 | 90724 | -0.0739539 | -0.6405851 | 0.49267721 | FALSE |
| 4 | 6 | 12 | 4 | 6 | 90748 | 0.16132352 | -0.3855244 | 0.7081714 | FALSE |
| 5 | 13 | 5 | 3 | 4 | 90901 | 0.46948806 | -0.3828508 | 1.32182696 | FALSE |
| 8 | 10 | 93 | 4 | 15 | 90991 | -0.2968752 | -1.362256 | 0.76850559 | FALSE |
| 4 | 1 | 8 | 1 | 7 | 91116 | -0.1545981 | -0.5592015 | 0.25000528 | FALSE |
| 2 | 5 | 3 | 3 | 2 | 91146 | 0.11145133 | -0.3543045 | 0.57720718 | FALSE |
| 2 | 9 | 2 | 3 | 2 | 91150 | 0.16388459 | -0.334204 | 0.66197321 | FALSE |
| 3 | 0 | 7 | 0 | 5 | 91160 | -0.0891421 | -0.3833905 | 0.20510637 | FALSE |
| 7 | 7 | 23 | 4 | 9 | 91663 | 0.15573287 | -0.4226911 | 0.73415688 | FALSE |
| 5 | 7 | 5 | 4 | 4 | 91696 | 0.29030446 | -0.0848678 | 0.66547675 | FALSE |
| 4 | 8 | 23 | 2 | 7 | 91744 | -0.0501072 | -0.7708695 | 0.67065502 | FALSE |
| 3 | 1 | 7 | 1 | 4 | 91773 | -0.0082144 | -0.3518428 | 0.33541392 | FALSE |
| 4 | 0 | 15 | 0 | 8 | 91918 | -0.3036225 | -0.7702498 | 0.16300477 | FALSE |
| 5 | 2 | 5 | 2 | 3 | 92216 | 0.05099978 | -0.2106138 | 0.31261339 | FALSE |
| 3 | 0 | 6 | 0 | 6 | 92260 | -0.0991038 | -0.3937664 | 0.19555879 | FALSE |
| 3 | 5 | 2 | 4 | 2 | 92280 | 0.19131373 | -0.2094638 | 0.59209122 | FALSE |
| 4 | 2 | 5 | 2 | 4 | 92333 | -0.0503624 | -0.3250529 | 0.22432803 | FALSE |
| 3 | 0 | 9 | 0 | 5 | 92666 | -0.1236643 | -0.450176 | 0.20284736 | FALSE |
| 2 | 0 | 9 | 0 | 5 | 92694 | -0.2348945 | -0.6848806 | 0.21509162 | FALSE |
| 3 | 2 | 4 | 1 | 4 | 93286 | -0.0055246 | -0.2777357 | 0.26668642 | FALSE |
| 3 | 0 | 7 | 0 | 5 | 93428 | -0.1665829 | -0.5042655 | 0.17109961 | FALSE |
| 4 | 2 | 4 | 2 | 3 | 93431 | 0.07858959 | -0.1843987 | 0.34157791 | FALSE |
| 2 | 0 | 31 | 0 | 10 | 93572 | -0.244494 | -1.0122506 | 0.52326253 | FALSE |
| 3 | 0 | 25 | 0 | 5 | 93575 | -0.3198211 | -0.8556523 | 0.21601003 | FALSE |
| 2 | 0 | 16 | 0 | 5 | 93724 | -0.2600586 | -0.7746995 | 0.25458235 | FALSE |
| 4 | 4 | 2 | 3 | 2 | 93913 | 0.17294188 | -0.0511366 | 0.39702037 | FALSE |
| 10 | 835 | 887 | 12 | 21 | 93915 | 1.48141242 | -1.4338191 | 4.39664393 | FALSE |
| 2 | 0 | 43 | 0 | 7 | 94053 | -0.5692815 | -1.592574 | 0.45401105 | FALSE |
| 2 | 0 | 21 | 0 | 6 | 94055 | -0.2749158 | -0.9964639 | 0.44663224 | FALSE |
| 3 | 1 | 20 | 1 | 6 | 94112 | -0.1899672 | -1.0070045 | 0.62707014 | FALSE |
| 1 | 0 | 6 | 0 | 5 | 94455 | -0.0933517 | -0.432097 | 0.24539365 | FALSE |
| 1 | 0 | 12 | 0 | 5 | 94458 | -0.1281346 | -0.6427089 | 0.3864397 | FALSE |
| 3 | 5 | 4 | 2 | 3 | 94595 | 0.09797368 | -0.3209541 | 0.51690143 | FALSE |
| 2 | 0 | 70 | 0 | 5 | 94961 | -0.5962478 | -1.3407001 | 0.14820453 | FALSE |
| 4 | 1 | 6 | 1 | 6 | 95409 | -0.0773282 | -0.3747136 | 0.22005717 | FALSE |
| 5 | 3 | 5 | 3 | 4 | 95443 | 0.16145514 | -0.130851 | 0.4537613 | FALSE |
| 1 | 0 | 10 | 0 | 5 | 95939 | -0.026488 | -0.4726279 | 0.41965197 | FALSE |
| 3 | 0 | 18 | 0 | 6 | 96108 | -0.3691867 | -0.8456655 | 0.10729205 | FALSE |
| 2 | 5 | 6 | 3 | 2 | 96304 | 0.0502206 | -0.5008729 | 0.60131409 | FALSE |
| 9 | 4 | 89 | 2 | 14 | 96323 | -0.9126782 | -2.1013301 | 0.27597381 | FALSE |
| 4 | 5 | 7 | 3 | 7 | 96495 | 0.08227016 | -0.4100437 | 0.57458403 | FALSE |
| 5 | 5 | 43 | 2 | 10 | 96996 | -0.12962 | -1.0728829 | 0.81364286 | FALSE |
| 8 | 3 | 50 | 3 | 14 | 97167 | -0.5217574 | -1.5203282 | 0.47681332 | FALSE |
| 2 | 6 | 2 | 4 | 1 | 97236 | 0.19868673 | -0.2393393 | 0.63671278 | FALSE |
| 3 | 0 | 70 | 0 | 12 | 97362 | -0.4748861 | -1.6179533 | 0.66818112 | FALSE |
| 2 | 0 | 21 | 0 | 6 | 97371 | -0.1271397 | -0.8006464 | 0.54636705 | FALSE |
| 3 | 27 | 7 | 5 | 2 | 97404 | 0.83270927 | -0.2022904 | 1.86770898 | FALSE |
| 3 | 0 | 24 | 0 | 8 | 97426 | -0.3252063 | -1.048117 | 0.39770447 | FALSE |
| 3 | 0 | 13 | 0 | 8 | 97511 | -0.1858273 | -0.6610018 | 0.28934718 | FALSE |
| 4 | 3 | 5 | 2 | 4 | 97663 | 0.04499946 | -0.2581831 | 0.34818198 | FALSE |
| 4 | 6 | 4 | 4 | 3 | 97674 | 0.1941246 | -0.2134509 | 0.6017001 | FALSE |
| 6 | 33 | 53 | 5 | 9 | 97681 | 0.57807012 | -0.6787664 | 1.83490666 | FALSE |
| 3 | 1 | 23 | 1 | 6 | 97748 | -0.3591041 | -1.1464046 | 0.4281963 | FALSE |
| 1 | 0 | 6 | 0 | 5 | 97750 | -0.1514001 | -0.5307305 | 0.22793039 | FALSE |
| 5 | 2 | 7 | 2 | 6 | 97960 | -0.0926468 | -0.4492759 | 0.26398228 | FALSE |
| 1 | 0 | 527 | 0 | 6 | 97996 | -0.7767467 | -2.878192 | 1.32469866 | FALSE |
| 1 | 0 | 125 | 0 | 6 | 98061 | -0.4421826 | -1.8534831 | 0.96911796 | FALSE |
| 2 | 0 | 16 | 0 | 5 | 98571 | -0.4342422 | -1.0320704 | 0.163586 | FALSE |
| 3 | 1 | 12 | 1 | 5 | 98644 | -0.1743922 | -0.7154534 | 0.36666904 | FALSE |
| 3 | 108 | 29 | 4 | 6 | 98747 | 0.91775975 | -0.6686246 | 2.50414416 | FALSE |
| 4 | 4 | 3 | 3 | 3 | 98748 | 0.14513567 | -0.1814433 | 0.47171462 | FALSE |
| 5 | 15 | 79 | 5 | 12 | 98852 | -0.0180027 | -1.2552345 | 1.21922898 | FALSE |
| 1 | 0 | 7 | 0 | 5 | 98872 | -0.1271772 | -0.4993133 | 0.24495896 | FALSE |
| 4 | 13 | 46 | 2 | 9 | 99020 | 0.11939207 | -0.851292 | 1.0900761 | FALSE |
| 4 | 76 | 474 | 3 | 11 | 99457 | 0.13837253 | -2.1043285 | 2.38107355 | FALSE |
| 4 | 1 | 9 | 1 | 6 | 99600 | -0.1232552 | -0.5032711 | 0.25676078 | FALSE |
| 2 | 29 | 0 | 5 | 0 | 99790 | 0.68560869 | -0.1755806 | 1.546798 | FALSE |
| 2 | 11 | 2 | 3 | 2 | 99811 | 0.30694874 | -0.1691649 | 0.78306237 | FALSE |
| 4 | 0 | 17 | 0 | 8 | 99904 | -0.4050537 | -0.8852002 | 0.07509288 | FALSE |
| 4 | 0 | 7 | 0 | 6 | 100295 | -0.0683125 | -0.3281807 | 0.1915558 | FALSE |
| 2 | 0 | 9 | 0 | 5 | 100508 | -0.0505065 | -0.4102205 | 0.30920751 | FALSE |
| 2 | 0 | 7 | 0 | 5 | 100720 | -0.0969082 | -0.4263012 | 0.23248473 | FALSE |
| 3 | 0 | 38 | 0 | 8 | 100806 | -0.3445335 | -1.0596927 | 0.37062573 | FALSE |
| 3 | 1 | 8 | 1 | 4 | 101694 | 0.01436619 | -0.3379346 | 0.36666702 | FALSE |
| 2 | 7 | 2 | 3 | 2 | 101794 | 0.15618904 | -0.276632 | 0.5890101 | FALSE |
| 6 | 4 | 11 | 3 | 6 | 101805 | 0.14817749 | -0.3090863 | 0.60544131 | FALSE |
| 5 | 161 | 111 | 6 | 11 | 102142 | 0.97016819 | -0.9671619 | 2.90749826 | FALSE |
| 4 | 2 | 9 | 2 | 5 | 102470 | -0.1046429 | -0.4532661 | 0.24398035 | FALSE |
| 2 | 0 | 8 | 0 | 5 | 102481 | -0.0726846 | -0.436462 | 0.29109288 | FALSE |
| 4 | 0 | 10 | 0 | 6 | 102559 | -0.1153274 | -0.4120092 | 0.18135442 | FALSE |
| 3 | 1 | 5 | 1 | 4 | 103149 | 0.08473608 | -0.2871597 | 0.45663182 | FALSE |
| 3 | 5 | 1 | 4 | 1 | 103289 | 0.23079209 | -0.1487277 | 0.61031187 | FALSE |
| 5 | 1 | 11 | 1 | 7 | 103409 | -0.2419242 | -0.707891 | 0.22404264 | FALSE |
| 3 | 0 | 8 | 0 | 6 | 103412 | -0.1845233 | -0.5651608 | 0.19611419 | FALSE |
| 2 | 0 | 6 | 0 | 5 | 103709 | -0.1794629 | -0.5196312 | 0.1607054 | FALSE |
| 3 | 1 | 13 | 1 | 6 | 104051 | -0.0816195 | -0.5845916 | 0.4213526 | FALSE |
| 4 | 2 | 25 | 2 | 6 | 104076 | -0.2172377 | -0.9112879 | 0.47681256 | FALSE |
| 5 | 4 | 5 | 4 | 4 | 104142 | 0.18783594 | -0.1143409 | 0.49001276 | FALSE |
| 4 | 2 | 11 | 1 | 4 | 104182 | -0.0842227 | -0.4866281 | 0.31818281 | FALSE |
| 4 | 0 | 96 | 0 | 14 | 104240 | -0.6236904 | -1.6135296 | 0.36614881 | FALSE |
| 2 | 0 | 25 | 0 | 5 | 104855 | -0.3968184 | -1.0011527 | 0.207516 | FALSE |
| 5 | 2 | 6 | 2 | 6 | 104995 | 0.00438435 | -0.2816332 | 0.29040188 | FALSE |
| 3 | 1 | 6 | 1 | 5 | 104997 | -0.0579438 | -0.3799622 | 0.26407467 | FALSE |
| 3 | 0 | 11 | 0 | 5 | 105193 | -0.091527 | -0.520383 | 0.33732891 | FALSE |
| 7 | 15 | 112 | 6 | 13 | 105481 | -0.0497521 | -1.445698 | 1.34619379 | FALSE |
| 3 | 36 | 6 | 4 | 4 | 105484 | 0.71157655 | -0.3308087 | 1.75396177 | FALSE |
| 10 | 4 | 20 | 3 | 10 | 105501 | -0.1044311 | -0.6023492 | 0.39348711 | FALSE |
| 5 | 3 | 13 | 2 | 5 | 105554 | 0.11072978 | -0.4213912 | 0.64285081 | FALSE |
| 4 | 0 | 13 | 0 | 9 | 105565 | -0.3213494 | -0.7959794 | 0.15328056 | FALSE |
| 4 | 8 | 11 | 2 | 6 | 105642 | 0.23451614 | -0.2663747 | 0.73540701 | FALSE |
| 3 | 1 | 6 | 1 | 5 | 106175 | -0.0293088 | -0.3620245 | 0.30340696 | FALSE |
| 3 | 2 | 10 | 2 | 6 | 106549 | -0.0326087 | -0.4906276 | 0.42541015 | FALSE |
| 3 | 5 | 2 | 3 | 2 | 106572 | 0.21351358 | -0.1323845 | 0.5594117 | FALSE |
| 6 | 2 | 10 | 2 | 6 | 106574 | -0.1545406 | -0.485729 | 0.17664789 | FALSE |
| 7 | 4 | 6 | 2 | 6 | 106579 | 0.0813557 | -0.2175798 | 0.3802912 | FALSE |
| 3 | 0 | 7 | 0 | 5 | 106716 | -0.138353 | -0.4236116 | 0.14690556 | FALSE |
| 4 | 2 | 10 | 2 | 4 | 106731 | -0.0903617 | -0.5241949 | 0.34347145 | FALSE |
| 6 | 10 | 9 | 1 | 6 | 106982 | -0.0351177 | -0.4724403 | 0.4022049 | FALSE |
| 3 | 1 | 13 | 1 | 5 | 107067 | -0.0470138 | -0.5397747 | 0.44574697 | FALSE |
| 4 | 5 | 4 | 2 | 3 | 107134 | 0.18074485 | -0.1340789 | 0.49556863 | FALSE |
| 3 | 1 | 5 | 1 | 4 | 107201 | 0.00678215 | -0.2724686 | 0.28603292 | FALSE |
| 2 | 0 | 10 | 0 | 6 | 107202 | -0.285984 | -0.7857261 | 0.21375806 | FALSE |
| 2 | 9 | 1 | 4 | 1 | 107206 | 0.29428197 | -0.2438013 | 0.83236527 | FALSE |
| 5 | 1 | 9 | 1 | 7 | 107251 | -0.2473009 | -0.596126 | 0.10152421 | FALSE |
| 4 | 1 | 6 | 1 | 5 | 107473 | -0.1439746 | -0.4145921 | 0.12664296 | FALSE |
| 7 | 5 | 40 | 2 | 9 | 108039 | -0.4274604 | -1.5386678 | 0.68374702 | FALSE |
| 5 | 5 | 9 | 3 | 5 | 108054 | 0.10260377 | -0.3644462 | 0.56965374 | FALSE |
| 3 | 1 | 193 | 1 | 6 | 108125 | -0.8376397 | -2.6082948 | 0.93301538 | FALSE |
| 4 | 4 | 19 | 2 | 7 | 108265 | -0.1365351 | -0.8522729 | 0.57920273 | FALSE |
| 5 | 52 | 138 | 8 | 8 | 108636 | 1.08922088 | -0.3315729 | 2.51001464 | FALSE |
| 3 | 3 | 6 | 3 | 4 | 109113 | 0.12006551 | -0.2723232 | 0.51245423 | FALSE |
| 6 | 5 | 5 | 4 | 3 | 110237 | 0.21980489 | -0.0853984 | 0.52500819 | FALSE |
| 5 | 1 | 16 | 1 | 8 | 110255 | -0.3220753 | -0.9300587 | 0.28590811 | FALSE |
| 4 | 7 | 2 | 3 | 2 | 110324 | 0.28621813 | -0.0096028 | 0.58203906 | FALSE |
| 6 | 4 | 9 | 3 | 6 | 110349 | 0.05213447 | -0.292074 | 0.39634291 | FALSE |
| 3 | 1 | 8 | 1 | 4 | 110379 | -0.2069131 | -0.5837628 | 0.16993661 | FALSE |
| 3 | 2 | 18 | 1 | 6 | 110539 | -0.1525021 | -0.8625829 | 0.55757865 | FALSE |
| 3 | 0 | 17 | 0 | 5 | 110599 | -0.2638539 | -0.7725807 | 0.24487289 | FALSE |
| 4 | 5 | 10 | 2 | 4 | 110807 | 0.20399067 | -0.2172694 | 0.62525073 | FALSE |
| 2 | 1 | 10 | 1 | 4 | 110942 | -0.0944217 | -0.6051386 | 0.41629527 | FALSE |
| 3 | 0 | 6 | 0 | 5 | 111096 | -0.2108521 | -0.503398 | 0.08169387 | FALSE |
| 9 | 4 | 59 | 3 | 16 | 111231 | -0.7523174 | -1.8239132 | 0.31927847 | FALSE |
| 4 | 1 | 6 | 1 | 5 | 111310 | -0.0986685 | -0.3675746 | 0.17023767 | FALSE |
| 3 | 0 | 7 | 0 | 5 | 111569 | -0.1661779 | -0.4759199 | 0.14356409 | FALSE |
| 3 | 3 | 8 | 2 | 4 | 111592 | -0.130726 | -0.7260427 | 0.46459073 | FALSE |
| 2 | 1 | 6 | 1 | 4 | 111784 | 0.01406985 | -0.3931581 | 0.42129782 | FALSE |
| 2 | 0 | 14 | 0 | 6 | 111791 | -0.0770055 | -0.5608684 | 0.40685727 | FALSE |
| 5 | 2 | 7 | 2 | 6 | 111840 | -0.0538349 | -0.408826 | 0.30115629 | FALSE |
| 3 | 42 | 14 | 2 | 8 | 111871 | 0.54541504 | -0.9547922 | 2.04562227 | FALSE |
| 4 | 5 | 11 | 4 | 6 | 111873 | 0.26539592 | -0.2178476 | 0.74863942 | FALSE |
| 3 | 0 | 16 | 0 | 5 | 111903 | -0.1140876 | -0.6140558 | 0.38588058 | FALSE |
| 2 | 0 | 10 | 0 | 7 | 112350 | -0.1982264 | -0.6172144 | 0.22076151 | FALSE |
| 2 | 0 | 55 | 0 | 7 | 112445 | -0.582272 | -1.7499534 | 0.58540946 | FALSE |
| 1 | 0 | 27 | 0 | 5 | 112452 | -0.4135355 | -1.2958939 | 0.46882284 | FALSE |
| 3 | 1 | 10 | 1 | 5 | 112561 | -0.1211681 | -0.6298595 | 0.38752325 | FALSE |
| 3 | 5 | 2 | 3 | 2 | 113472 | 0.19748622 | -0.1643842 | 0.55935661 | FALSE |
| 4 | 4 | 6 | 3 | 5 | 113867 | 0.12355002 | -0.263674 | 0.51077406 | FALSE |
| 3 | 4 | 2 | 3 | 2 | 114134 | 0.22253685 | -0.0667996 | 0.5118733 | FALSE |
| 1 | 0 | 13 | 0 | 5 | 114160 | -0.1823086 | -0.7082006 | 0.34358332 | FALSE |
| 5 | 4 | 18 | 2 | 6 | 114181 | 0.05581566 | -0.4623034 | 0.57393475 | FALSE |
| 6 | 44 | 224 | 3 | 16 | 114186 | -0.0770228 | -1.9489036 | 1.79485807 | FALSE |
| 4 | 0 | 267 | 0 | 14 | 114188 | -0.8246919 | -2.3911769 | 0.74179315 | FALSE |
| 4 | 2 | 8 | 2 | 5 | 114439 | 0.01670543 | -0.3421564 | 0.37556722 | FALSE |
| 4 | 0 | 9 | 0 | 6 | 114453 | -0.2277531 | -0.5720317 | 0.11652542 | FALSE |
| 8 | 19 | 29 | 7 | 15 | 114465 | 0.08263768 | -0.8081735 | 0.97344884 | FALSE |
| 2 | 6 | 3 | 3 | 2 | 114526 | 0.16768386 | -0.2781121 | 0.6134798 | FALSE |
| 4 | 0 | 8 | 0 | 6 | 114533 | -0.1994619 | -0.5327268 | 0.13380291 | FALSE |
| 4 | 4 | 4 | 3 | 4 | 114544 | 0.09618576 | -0.2120563 | 0.40442785 | FALSE |
| 4 | 4 | 2 | 3 | 2 | 114565 | 0.17596397 | -0.0543414 | 0.4062693 | FALSE |
| 3 | 4 | 25 | 2 | 8 | 114583 | -0.160471 | -0.9565109 | 0.63556884 | FALSE |
| 4 | 2 | 5 | 2 | 5 | 114595 | 0.04030333 | -0.2640523 | 0.34465896 | FALSE |
| 3 | 1 | 8 | 1 | 4 | 114684 | -0.0379429 | -0.4089601 | 0.33307426 | FALSE |
| 1 | 0 | 7 | 0 | 5 | 114718 | -0.1694942 | -0.5848248 | 0.24583643 | FALSE |
| 4 | 0 | 6 | 0 | 5 | 114770 | -0.1288086 | -0.3624874 | 0.10487031 | FALSE |
| 2 | 0 | 10 | 0 | 5 | 114801 | -0.0820482 | -0.464352 | 0.30025556 | FALSE |
| 4 | 1 | 11 | 1 | 5 | 114861 | -0.1062636 | -0.4836841 | 0.27115686 | FALSE |
| 3 | 10 | 8 | 3 | 4 | 114887 | 0.32345461 | -0.2443524 | 0.89126165 | FALSE |
| 3 | 1 | 9 | 1 | 5 | 115032 | -0.0903613 | -0.538523 | 0.35780035 | FALSE |
| 3 | 0 | 21 | 0 | 12 | 115077 | -0.3637062 | -0.9696239 | 0.24221146 | FALSE |
| 3 | 0 | 12 | 0 | 9 | 115078 | -0.2590056 | -0.6876836 | 0.16967247 | FALSE |
| 4 | 1 | 10 | 1 | 5 | 115258 | -0.2458326 | -0.6688479 | 0.17718267 | FALSE |
| 2 | 8 | 2 | 3 | 2 | 115378 | 0.23267962 | -0.2591487 | 0.72450794 | FALSE |
| 7 | 6 | 6 | 3 | 5 | 115525 | 0.36575089 | -0.2065987 | 0.9381005 | FALSE |
| 7 | 7 | 11 | 4 | 8 | 115526 | 0.08281718 | -0.3560355 | 0.52166986 | FALSE |
| 2 | 12 | 2 | 4 | 2 | 115539 | 0.39897959 | -0.2542737 | 1.05223291 | FALSE |
| 2 | 9 | 2 | 4 | 1 | 115591 | 0.31504245 | -0.2492916 | 0.87937648 | FALSE |
| 5 | 3 | 4 | 2 | 3 | 115601 | 0.1353421 | -0.1953048 | 0.46598898 | FALSE |
| 4 | 2 | 4 | 1 | 4 | 115955 | 0.00100772 | -0.2344226 | 0.23643805 | FALSE |
| 2 | 0 | 19 | 0 | 5 | 116233 | -0.2725221 | -0.8461489 | 0.30110483 | FALSE |
| 4 | 2 | 8 | 2 | 6 | 116381 | -0.0720822 | -0.509953 | 0.36578869 | FALSE |
| 5 | 6 | 5 | 4 | 5 | 116413 | 0.18587414 | -0.281459 | 0.65320733 | FALSE |
| 3 | 0 | 6 | 0 | 5 | 116435 | -0.1824014 | -0.504989 | 0.14018616 | FALSE |
| 3 | 0 | 6 | 0 | 5 | 116608 | -0.151592 | -0.4148431 | 0.11165903 | FALSE |
| 4 | 2 | 4 | 2 | 4 | 117221 | 0.05491426 | -0.2029031 | 0.31273166 | FALSE |
| 4 | 1 | 21 | 1 | 10 | 117421 | -0.4056048 | -1.0200689 | 0.20885932 | FALSE |
| 5 | 3 | 7 | 1 | 4 | 117442 | -0.0112504 | -0.3425943 | 0.32009344 | FALSE |
| 5 | 2 | 7 | 2 | 5 | 117567 | 0.08964625 | -0.2220876 | 0.40138013 | FALSE |
| 6 | 5 | 16 | 2 | 11 | 118157 | -0.1085601 | -0.5828103 | 0.36569011 | FALSE |
| 5 | 28 | 11 | 6 | 4 | 118250 | 0.66544019 | -0.1196937 | 1.45057411 | FALSE |
| 4 | 1 | 6 | 1 | 5 | 118370 | -0.0043841 | -0.3091783 | 0.3004101 | FALSE |
| 1 | 0 | 11 | 0 | 5 | 118489 | -0.1843334 | -0.6776311 | 0.30896425 | FALSE |
| 4 | 2 | 6 | 2 | 5 | 118524 | 0.10335037 | -0.2108565 | 0.41755721 | FALSE |
| 5 | 6 | 35 | 3 | 9 | 118529 | 0.06667185 | -0.8413665 | 0.97471016 | FALSE |
| 4 | 1 | 11 | 1 | 6 | 118779 | -0.1826799 | -0.6861936 | 0.3208338 | FALSE |
| 3 | 4 | 4 | 2 | 4 | 118840 | 0.0789298 | -0.3485355 | 0.50639507 | FALSE |
| 6 | 7 | 8 | 4 | 6 | 119042 | 0.18686892 | -0.1923322 | 0.56607002 | FALSE |
| 2 | 0 | 23 | 0 | 6 | 119071 | -0.2226332 | -0.9120582 | 0.46679184 | FALSE |
| 1 | 0 | 26 | 0 | 5 | 119199 | -0.1726488 | -0.9124384 | 0.56714073 | FALSE |
| 3 | 0 | 11 | 0 | 5 | 119477 | -0.3397477 | -0.7639009 | 0.08440552 | FALSE |
| 3 | 0 | 9 | 0 | 7 | 119856 | -0.2519547 | -0.6500005 | 0.14609107 | FALSE |
| 6 | 77 | 58 | 7 | 10 | 120513 | 1.56481874 | -0.0869641 | 3.21660155 | FALSE |
| 2 | 0 | 7 | 0 | 5 | 120814 | -0.1624042 | -0.4722018 | 0.14739347 | FALSE |
| 3 | 2 | 4 | 2 | 3 | 121484 | 0.11033448 | -0.1808426 | 0.40151156 | FALSE |
| 6 | 23 | 271 | 4 | 16 | 121901 | -0.357491 | -2.2850888 | 1.57010688 | FALSE |
| 4 | 2 | 5 | 2 | 3 | 122124 | -0.010707 | -0.2692184 | 0.2478044 | FALSE |
| 1 | 0 | 24 | 0 | 5 | 122383 | -0.1403116 | -0.7889446 | 0.50832145 | FALSE |
| 2 | 1 | 12 | 1 | 4 | 122394 | -0.0946443 | -0.6272047 | 0.43791612 | FALSE |
| 2 | 0 | 9 | 0 | 5 | 122520 | -0.2017633 | -0.5838921 | 0.18036553 | FALSE |
| 5 | 6 | 116 | 3 | 15 | 122896 | -0.3938579 | -1.6558923 | 0.86817641 | FALSE |
| 2 | 0 | 22 | 0 | 6 | 122916 | -0.1620248 | -0.7775203 | 0.45347077 | FALSE |
| 4 | 4 | 2 | 3 | 2 | 123149 | 0.22861145 | -0.0013938 | 0.45861665 | FALSE |
| 4 | 1 | 6 | 1 | 4 | 123189 | -0.0216323 | -0.307205 | 0.26394046 | FALSE |
| 4 | 7 | 4 | 2 | 3 | 123258 | 0.13915731 | -0.1996723 | 0.47798697 | FALSE |
| 3 | 1 | 5 | 1 | 5 | 123272 | -0.0807923 | -0.4164862 | 0.25490155 | FALSE |
| 6 | 9 | 10 | 5 | 7 | 123277 | 0.20425807 | -0.3248991 | 0.73341522 | FALSE |
| 1 | 0 | 27 | 0 | 5 | 123280 | -0.3535535 | -1.2373797 | 0.53027263 | FALSE |
| 5 | 7 | 7 | 4 | 3 | 123307 | 0.20248281 | -0.3091533 | 0.71411888 | FALSE |
| 2 | 0 | 33 | 0 | 7 | 123327 | -0.4169483 | -1.1740837 | 0.34018711 | FALSE |
| 2 | 6 | 10 | 3 | 3 | 123340 | 0.07840372 | -0.5638983 | 0.72070571 | FALSE |
| 5 | 2 | 7 | 2 | 4 | 123348 | -0.0316076 | -0.3262421 | 0.26302683 | FALSE |
| 5 | 1 | 15 | 1 | 6 | 123349 | -0.1132806 | -0.5728791 | 0.34631795 | FALSE |
| 5 | 23 | 11 | 5 | 6 | 123350 | 0.31524799 | -0.5952804 | 1.2257764 | FALSE |
| 3 | 1 | 6 | 1 | 4 | 123357 | -0.1583962 | -0.4748481 | 0.15805569 | FALSE |
| 3 | 1 | 14 | 1 | 4 | 123370 | -0.0988407 | -0.6290372 | 0.43135585 | FALSE |
| 2 | 0 | 9 | 0 | 5 | 123507 | -0.1423813 | -0.5617768 | 0.27701407 | FALSE |
| 4 | 6 | 7 | 3 | 6 | 123528 | 0.11237469 | -0.327446 | 0.55219536 | FALSE |
| 3 | 0 | 8 | 0 | 5 | 123862 | -0.1742898 | -0.5032747 | 0.15469514 | FALSE |
| 4 | 7 | 2 | 4 | 2 | 124095 | 0.2932311 | -0.0750448 | 0.66150698 | FALSE |
| 7 | 11 | 28 | 4 | 13 | 124252 | 0.07040717 | -0.7784806 | 0.91929496 | FALSE |
| 2 | 5 | 10 | 2 | 3 | 124458 | -0.0387963 | -0.7947265 | 0.71713393 | FALSE |
| 2 | 7 | 28 | 3 | 4 | 124526 | 0.29977616 | -0.5188529 | 1.11840526 | FALSE |
| 1 | 0 | 14 | 0 | 5 | 124558 | -0.0730627 | -0.6210415 | 0.47491609 | FALSE |
| 1 | 0 | 10 | 0 | 5 | 124823 | -0.1931876 | -0.6463771 | 0.260002 | FALSE |
| 4 | 5 | 5 | 3 | 2 | 126122 | 0.20852464 | -0.1295597 | 0.54660896 | FALSE |
| 3 | 0 | 7 | 0 | 5 | 126207 | -0.2218694 | -0.5241262 | 0.08038735 | FALSE |
| 3 | 0 | 12 | 0 | 6 | 126377 | -0.0515521 | -0.4587957 | 0.35569145 | FALSE |
| 2 | 6 | 3 | 3 | 2 | 126809 | 0.20656594 | -0.2414543 | 0.65458614 | FALSE |
| 2 | 0 | 12 | 0 | 6 | 127297 | -0.2274703 | -0.688931 | 0.23399029 | FALSE |
| 6 | 4 | 7 | 2 | 6 | 127323 | 0.05907492 | -0.2703029 | 0.38845272 | FALSE |
| 7 | 8 | 19 | 5 | 8 | 127389 | 0.16779356 | -0.406369 | 0.74195613 | FALSE |
| 2 | 0 | 7 | 0 | 6 | 127413 | -0.0557589 | -0.399593 | 0.28807511 | FALSE |
| 3 | 1 | 6 | 1 | 4 | 127502 | 0.01398066 | -0.3129786 | 0.34093992 | FALSE |
| 2 | 3 | 6 | 2 | 3 | 127573 | 0.01224932 | -0.4638601 | 0.48835872 | FALSE |
| 4 | 2 | 7 | 2 | 3 | 128265 | 0.05539879 | -0.2545883 | 0.36538584 | FALSE |
| 4 | 2 | 6 | 2 | 3 | 128632 | 0.04598514 | -0.3858482 | 0.4778185 | FALSE |
| 5 | 3 | 15 | 3 | 4 | 128714 | -0.1184664 | -0.7872524 | 0.55031967 | FALSE |
| 2 | 2 | 16 | 2 | 5 | 128869 | -0.0092182 | -0.6132766 | 0.59484015 | FALSE |
| 2 | 22 | 0 | 5 | 0 | 128905 | 0.6325808 | -0.1820605 | 1.44722214 | FALSE |
| 5 | 2 | 8 | 2 | 6 | 129127 | 0.11760032 | -0.1937633 | 0.42896393 | FALSE |
| 4 | 1 | 12 | 1 | 6 | 129759 | -0.1918907 | -0.670164 | 0.28638268 | FALSE |
| 3 | 0 | 19 | 0 | 6 | 129843 | -0.1593481 | -0.7518742 | 0.43317796 | FALSE |
| 3 | 6 | 46 | 2 | 9 | 130711 | 0.04855216 | -0.8673695 | 0.96447384 | FALSE |
| 3 | 0 | 10 | 0 | 6 | 130725 | -0.1415954 | -0.4933248 | 0.21013406 | FALSE |
| 3 | 1 | 8 | 1 | 5 | 131415 | -0.0482166 | -0.4023606 | 0.30592727 | FALSE |
| 5 | 7 | 4 | 6 | 2 | 131780 | 0.31452152 | -0.0330144 | 0.66205749 | FALSE |
| 5 | 7 | 4 | 4 | 2 | 132038 | 0.22641988 | -0.1054565 | 0.5582963 | FALSE |
| 4 | 3 | 4 | 2 | 4 | 132042 | 0.11541735 | -0.1790507 | 0.40988546 | FALSE |
| 4 | 1 | 6 | 1 | 6 | 132052 | -0.0029188 | -0.3314395 | 0.325602 | FALSE |
| 1 | 0 | 52 | 0 | 5 | 132418 | -0.5142562 | -1.4656737 | 0.43716124 | FALSE |
| 4 | 3 | 4 | 2 | 4 | 132620 | 0.06986966 | -0.2069144 | 0.34665372 | FALSE |
| 7 | 4 | 22 | 3 | 12 | 133020 | -0.3304348 | -0.8308162 | 0.16994669 | FALSE |
| 3 | 0 | 9 | 0 | 5 | 133211 | -0.2003295 | -0.5854546 | 0.18479551 | FALSE |
| 5 | 25 | 27 | 6 | 9 | 133482 | 0.38713863 | -0.5956825 | 1.36995975 | FALSE |
| 3 | 0 | 37 | 0 | 9 | 133695 | -0.4112574 | -1.2457027 | 0.4231879 | FALSE |
| 3 | 2 | 4 | 2 | 3 | 133981 | 0.01136938 | -0.2561994 | 0.2789382 | FALSE |
| 4 | 3 | 3 | 2 | 3 | 134210 | 0.11555056 | -0.2476574 | 0.47875852 | FALSE |
| 7 | 3 | 9 | 3 | 8 | 134450 | 0.15937414 | -0.1758959 | 0.49464417 | FALSE |
| 1 | 0 | 9 | 0 | 5 | 134454 | -0.1409111 | -0.5767806 | 0.29495851 | FALSE |
| 2 | 0 | 12 | 0 | 6 | 135324 | -0.3229869 | -0.8090685 | 0.16309476 | FALSE |
| 6 | 5 | 7 | 5 | 6 | 136001 | 0.1677292 | -0.2101506 | 0.54560896 | FALSE |
| 6 | 3 | 6 | 3 | 6 | 136004 | 0.05294421 | -0.2388985 | 0.34478697 | FALSE |
| 4 | 0 | 11 | 0 | 6 | 136569 | -0.2755995 | -0.6654129 | 0.11421396 | FALSE |
| 3 | 0 | 7 | 0 | 6 | 136655 | -0.2090142 | -0.5319507 | 0.11392219 | FALSE |
| 4 | 0 | 8 | 0 | 7 | 136698 | -0.2080984 | -0.4850543 | 0.06885741 | FALSE |
| 2 | 6 | 0 | 5 | 0 | 136958 | 0.30753379 | -0.057765 | 0.67283259 | FALSE |
| 4 | 2 | 6 | 1 | 6 | 138061 | -0.0868898 | -0.369719 | 0.19593939 | FALSE |
| 5 | 16 | 75 | 3 | 12 | 138358 | 0.15986428 | -0.9584928 | 1.27822134 | FALSE |
| 4 | 1 | 6 | 1 | 5 | 138385 | -0.0536756 | -0.3461534 | 0.23880216 | FALSE |
| 5 | 4 | 3 | 2 | 3 | 138569 | 0.19884333 | -0.0541725 | 0.45185919 | FALSE |
| 5 | 3 | 8 | 2 | 6 | 138985 | 0.11329116 | -0.3332155 | 0.55979777 | FALSE |
| 2 | 1 | 15 | 1 | 6 | 139240 | -0.1545203 | -0.748737 | 0.43969636 | FALSE |
| 2 | 0 | 25 | 0 | 9 | 139867 | -0.1918421 | -0.8841194 | 0.50043512 | FALSE |
| 2 | 2 | 7 | 1 | 4 | 139942 | 0.12030406 | -0.2801586 | 0.52076673 | FALSE |
| 4 | 6 | 12 | 4 | 6 | 140215 | 0.08942887 | -0.505534 | 0.68439175 | FALSE |
| 4 | 0 | 21 | 0 | 9 | 140667 | -0.2646205 | -0.8104093 | 0.28116829 | FALSE |
| 3 | 2 | 11 | 2 | 4 | 140957 | 0.06977918 | -0.4209605 | 0.56051886 | FALSE |
| 4 | 6 | 6 | 3 | 2 | 141175 | 0.18984921 | -0.2194165 | 0.59911489 | FALSE |
| 4 | 1 | 21 | 1 | 6 | 141177 | -0.2733909 | -0.9100227 | 0.36324098 | FALSE |
| 4 | 0 | 16 | 0 | 7 | 141183 | -0.270037 | -0.8304521 | 0.29037815 | FALSE |
| 4 | 14 | 29 | 1 | 11 | 141955 | 0.03844661 | -0.7540429 | 0.83093614 | FALSE |
| 4 | 2 | 26 | 2 | 8 | 141962 | -0.2035585 | -0.8961092 | 0.48899225 | FALSE |
| 2 | 0 | 6 | 0 | 5 | 142153 | -0.1121621 | -0.3927302 | 0.16840603 | FALSE |
| 4 | 1 | 6 | 1 | 4 | 142168 | -0.0519511 | -0.3808578 | 0.27695567 | FALSE |
| 3 | 0 | 9 | 0 | 5 | 144243 | -0.2761801 | -0.6518032 | 0.09944301 | FALSE |
| 2 | 1 | 6 | 1 | 4 | 144938 | -0.0971639 | -0.4830105 | 0.28868265 | FALSE |
| 5 | 13 | 17 | 3 | 5 | 146118 | 0.38909802 | -0.3592879 | 1.13748392 | FALSE |
| 6 | 5 | 8 | 4 | 4 | 146120 | 0.17233156 | -0.1982826 | 0.54294567 | FALSE |
| 4 | 1 | 8 | 1 | 6 | 146318 | -0.1250196 | -0.4585999 | 0.20856069 | FALSE |
| 4 | 17 | 21 | 6 | 6 | 146322 | 0.45645663 | -0.3734505 | 1.28636379 | FALSE |
| 6 | 9 | 15 | 4 | 7 | 146551 | 0.12431722 | -0.4758835 | 0.72451794 | FALSE |
| 6 | 8 | 12 | 4 | 10 | 146552 | 0.022631 | -0.6285421 | 0.67380411 | FALSE |
| 3 | 8 | 5 | 3 | 2 | 146553 | 0.24117643 | -0.1376949 | 0.62004778 | FALSE |
| 3 | 12 | 2 | 4 | 2 | 146554 | 0.45103556 | -0.0151183 | 0.9171894 | FALSE |
| 3 | 1 | 5 | 1 | 4 | 146788 | -0.0284839 | -0.3390524 | 0.2820846 | FALSE |
| 9 | 15 | 22 | 6 | 9 | 148073 | 0.71332934 | -0.1193381 | 1.54599675 | FALSE |
| 5 | 6 | 6 | 2 | 6 | 148078 | 0.38472942 | -0.169085 | 0.93854387 | FALSE |

Table S3B

| Number of patients | Number of reads in Case samples | Number of reads in Controls | Number of positive Case samples | Number of positive Controls | OTU | fold change | 2.5 confint | 97.5 confint | agree |
| --- | --- | --- | --- | --- | --- | --- | --- | --- | --- |
| 8 | 5 | 539 | 4 | 29 | 44063 | -2.299595 | -4.0924329 | -0.506757 | TRUE |
| 6 | 1 | 299 | 1 | 21 | 78889 | -1.8648939 | -3.5218664 | -0.2079214 | TRUE |
| 6 | 2 | 255 | 2 | 20 | 83479 | -1.7574361 | -3.3275391 | -0.1873332 | TRUE |
| 9 | 8 | 249 | 3 | 26 | 58092 | -1.5939697 | -3.1192121 | -0.0687272 | TRUE |
| 10 | 6 | 76 | 4 | 23 | 61406 | -0.8158752 | -1.6265876 | -0.0051628 | TRUE |
| 4 | 0 | 42 | 0 | 10 | 72729 | -0.7302543 | -1.456844 | -0.0036645 | TRUE |
| 5 | 0 | 22 | 0 | 9 | 49233 | -0.5282392 | -1.0533477 | -0.0031307 | TRUE |
| 5 | 0 | 17 | 0 | 8 | 88862 | -0.4759604 | -0.8715758 | -0.080345 | TRUE |
| 5 | 0 | 12 | 0 | 10 | 50630 | -0.3299817 | -0.6550911 | -0.0048722 | TRUE |
| 4 | 0 | 8 | 0 | 6 | 50611 | -0.3224727 | -0.5804318 | -0.0645135 | TRUE |
| 3 | 0 | 8 | 0 | 5 | 34895 | -0.3206486 | -0.6312942 | -0.0100031 | TRUE |
| 3 | 0 | 7 | 0 | 5 | 40968 | -0.267287 | -0.5302811 | -0.0042929 | TRUE |
| 4 | 0 | 7 | 0 | 6 | 39031 | -0.2504333 | -0.4955516 | -0.0053149 | TRUE |
| 5 | 4 | 2 | 3 | 2 | 81205 | 0.22550437 | 0.00046762 | 0.45054112 | TRUE |
| 5 | 3 | 3 | 3 | 3 | 67095 | 0.25288436 | 0.049085 | 0.45668371 | TRUE |
| 4 | 4 | 2 | 3 | 2 | 55767 | 0.26934993 | 0.02885492 | 0.50984494 | TRUE |
| 5 | 5 | 2 | 4 | 2 | 146025 | 0.27088112 | 0.03131463 | 0.51044761 | TRUE |
| 4 | 4 | 2 | 4 | 2 | 27908 | 0.31130418 | 0.05909281 | 0.56351556 | TRUE |
| 4 | 6 | 1 | 4 | 1 | 128385 | 0.31731465 | 0.07211706 | 0.56251224 | TRUE |
| 4 | 5 | 1 | 4 | 1 | 12412 | 0.33507901 | 0.11898421 | 0.5511738 | TRUE |
| 4 | 5 | 1 | 4 | 1 | 44709 | 0.34359557 | 0.14292861 | 0.54426253 | TRUE |
| 6 | 6 | 4 | 5 | 3 | 62899 | 0.35142547 | 0.06643456 | 0.63641638 | TRUE |
| 3 | 6 | 1 | 4 | 1 | 91937 | 0.35427617 | 0.01898173 | 0.68957061 | TRUE |
| 3 | 7 | 2 | 4 | 1 | 39677 | 0.36577383 | 0.0563726 | 0.67517506 | TRUE |
| 6 | 7 | 2 | 5 | 2 | 79546 | 0.37438995 | 0.10863799 | 0.64014192 | TRUE |
| 4 | 7 | 1 | 4 | 1 | 75130 | 0.38946379 | 0.13438606 | 0.64454153 | TRUE |
| 4 | 7 | 1 | 4 | 1 | 111895 | 0.38970265 | 0.11590159 | 0.66350371 | TRUE |
| 4 | 7 | 3 | 6 | 3 | 27408 | 0.39189131 | 0.05331347 | 0.73046914 | TRUE |
| 3 | 10 | 1 | 4 | 1 | 21552 | 0.40247501 | 0.02642813 | 0.77852189 | TRUE |
| 5 | 9 | 3 | 4 | 2 | 126801 | 0.40554899 | 0.06847016 | 0.74262783 | TRUE |
| 6 | 7 | 5 | 6 | 3 | 45956 | 0.40838767 | 0.0995061 | 0.71726925 | TRUE |
| 3 | 8 | 1 | 4 | 1 | 124371 | 0.41409948 | 0.05937915 | 0.76881981 | TRUE |
| 3 | 12 | 4 | 4 | 2 | 101155 | 0.42039715 | 0.00155062 | 0.83924368 | TRUE |
| 2 | 8 | 0 | 6 | 0 | 115896 | 0.42214794 | 0.04974854 | 0.79454735 | TRUE |
| 2 | 8 | 0 | 5 | 0 | 120438 | 0.4339122 | 0.0999878 | 0.76783659 | TRUE |
| 5 | 7 | 7 | 4 | 4 | 42829 | 0.44064062 | 0.03564005 | 0.84564119 | TRUE |
| 7 | 10 | 7 | 6 | 5 | 56166 | 0.47188879 | 0.01952566 | 0.92425192 | TRUE |
| 6 | 9 | 7 | 6 | 5 | 41897 | 0.47570282 | 0.0572268 | 0.89417883 | TRUE |
| 3 | 10 | 1 | 4 | 1 | 18831 | 0.47865762 | 0.14429276 | 0.81302248 | TRUE |
| 4 | 11 | 3 | 4 | 2 | 14616 | 0.48212875 | 0.06199346 | 0.90226405 | TRUE |
| 3 | 7 | 0 | 7 | 0 | 124610 | 0.48474791 | 0.1338996 | 0.83559623 | TRUE |
| 7 | 13 | 7 | 6 | 4 | 42660 | 0.48668526 | 0.03468948 | 0.93868104 | TRUE |
| 4 | 7 | 1 | 6 | 1 | 25920 | 0.49250811 | 0.26638091 | 0.71863531 | TRUE |
| 3 | 13 | 2 | 5 | 2 | 44850 | 0.50354995 | 0.05974117 | 0.94735873 | TRUE |
| 4 | 14 | 6 | 3 | 4 | 133358 | 0.50526411 | 0.01686171 | 0.99366651 | TRUE |
| 2 | 9 | 0 | 5 | 0 | 14645 | 0.51828238 | 0.11060304 | 0.92596173 | TRUE |
| 3 | 11 | 0 | 5 | 0 | 71962 | 0.52083404 | 0.09937508 | 0.942293 | TRUE |
| 4 | 9 | 3 | 6 | 2 | 73541 | 0.52295548 | 0.07177415 | 0.97413681 | TRUE |
| 8 | 13 | 19 | 6 | 5 | 122798 | 0.52934109 | 0.0208178 | 1.03786438 | TRUE |
| 4 | 14 | 3 | 6 | 2 | 104573 | 0.54650108 | 0.0309364 | 1.06206577 | TRUE |
| 4 | 8 | 2 | 6 | 2 | 111921 | 0.55414526 | 0.06650469 | 1.04178583 | TRUE |
| 5 | 17 | 3 | 5 | 3 | 10387 | 0.56074582 | 0.11785653 | 1.00363512 | TRUE |
| 4 | 8 | 1 | 7 | 1 | 46540 | 0.5630085 | 0.33702609 | 0.78899092 | TRUE |
| 4 | 13 | 5 | 6 | 2 | 10918 | 0.62563663 | 0.16310927 | 1.088164 | TRUE |
| 7 | 12 | 11 | 7 | 6 | 34210 | 0.63462467 | 0.11530764 | 1.1539417 | TRUE |
| 2 | 12 | 0 | 5 | 0 | 9033 | 0.65379488 | 0.18546926 | 1.1221205 | TRUE |
| 7 | 15 | 22 | 9 | 9 | 61938 | 0.67797911 | 0.07673026 | 1.27922795 | TRUE |
| 3 | 23 | 3 | 4 | 1 | 122796 | 0.67835428 | 0.00987673 | 1.34683184 | TRUE |
| 3 | 21 | 1 | 5 | 1 | 37561 | 0.68781606 | 0.04305776 | 1.33257435 | TRUE |
| 7 | 13 | 10 | 6 | 6 | 110534 | 0.69976687 | 0.20879511 | 1.19073863 | TRUE |
| 3 | 18 | 0 | 6 | 0 | 128929 | 0.71380612 | 0.04694115 | 1.3806711 | TRUE |
| 3 | 17 | 0 | 6 | 0 | 11299 | 0.73181732 | 0.11947977 | 1.34415487 | TRUE |
| 5 | 16 | 2 | 4 | 2 | 61607 | 0.73604681 | 0.06479894 | 1.40729469 | TRUE |
| 5 | 19 | 3 | 6 | 3 | 55756 | 0.7616852 | 0.30103895 | 1.22233144 | TRUE |
| 3 | 26 | 1 | 5 | 1 | 114917 | 0.81952409 | 0.00097144 | 1.63807674 | TRUE |
| 5 | 26 | 2 | 6 | 2 | 49383 | 0.86344072 | 0.18256727 | 1.54431416 | TRUE |
| 3 | 43 | 0 | 7 | 0 | 57771 | 0.95305491 | 0.05974829 | 1.84636153 | TRUE |
| 3 | 45 | 0 | 7 | 0 | 7560 | 0.95503011 | 0.11780228 | 1.79225795 | TRUE |
| 10 | 54 | 23 | 9 | 12 | 66329 | 0.97506106 | 0.11968736 | 1.83043477 | TRUE |
| 3 | 46 | 0 | 6 | 0 | 54652 | 1.08759449 | 0.08673628 | 2.0884527 | TRUE |
| 5 | 46 | 4 | 8 | 4 | 11052 | 1.10173386 | 0.25834953 | 1.94511818 | TRUE |
| 7 | 44 | 35 | 10 | 9 | 30000 | 1.17663763 | 0.32719494 | 2.02608033 | TRUE |
| 4 | 61 | 21 | 8 | 5 | 31052 | 1.20846514 | 0.09675773 | 2.32017255 | TRUE |
| 2 | 89 | 0 | 6 | 0 | 8020 | 1.34342634 | 0.21075359 | 2.4760991 | TRUE |
| 2 | 113 | 0 | 8 | 0 | 29555 | 1.45079659 | 0.12760944 | 2.77398374 | TRUE |
| 4 | 91 | 8 | 10 | 5 | 50418 | 1.48416096 | 0.41654185 | 2.55178006 | TRUE |
| 11 | 493 | 95 | 12 | 17 | 64548 | 1.78520363 | 0.10562134 | 3.46478591 | TRUE |
| 5 | 220 | 42 | 11 | 9 | 7161 | 1.95559527 | 0.288481 | 3.62270954 | TRUE |
| 6 | 3648 | 56 | 12 | 5 | 40456 | 3.9620957 | 1.23741358 | 6.68677781 | TRUE |
